# Individual factors in the relationship between stress and resilience in mental health psychology practitioners during the COVID-19 pandemic

**DOI:** 10.1177/13591053211059393

**Published:** 2021-12-07

**Authors:** Constantina Panourgia, Agata Wezyk, Annita Ventouris, Amanda Comoretto, Zoe Taylor, Ala Yankouskaya

**Affiliations:** 1 Bournemouth University, UK; 2 University of West London, UK; 3 Université Catholique de Lyon (UCly), France

**Keywords:** burnout, COVID-19, optimism, resilience, secondary traumatic stress

## Abstract

Utilising an online survey, this study aimed to investigate the concurrent effects of pre-pandemic and COVID-19 stress on resilience in Mental Health Psychology Practitioners (MHPPs) (*n* *=* 325), focussing on the mediation effects of specific individual factors. Optimism, burnout and secondary traumatic stress, but not coping strategies, self-efficacy, compassion satisfaction, or self-compassion, mediated both the relationship between pre-pandemic stress and resilience and COVID-19 stress and resilience. Increased job demands caused by the pandemic, the nature and duration of COVID-19 stress may explain this finding. Training and supervision practices can help MHPPs deal with job demands under circumstances of general and extreme stress.

Although the erroneous belief that Mental Health Psychology Practitioners (MHPPs) should not be affected by their work has prevailed for years (Pope, 1994), research indicates that MHPPs are susceptible to occupational risks. Dealing with the adverse life events of others (Lamb and Cogan, 2016) and specific role-demands related to the nature of therapy are among the factors contributing to MHPPs experiencing high levels of work-related stress and anxiety. Unsurprisingly, during the COVID-19 pandemic mental health care demands have increased (Pierce et al., 2020; Wang et al., 2020a). The pandemic has brought dramatic changes in everyday life, which is now structured around high levels of unpredictability, and has altered the way societies function on a collective level. These changes have exposed MHPPs to additional work stress, potentially impacting their wellbeing in a negative way; as a result, the quality of care and service-user outcomes are likely to be affected, as suggested by diverse research findings (e.g. Delgadillo et al., 2018).

A pandemic like COVID-19 is considered among situations that may lead to collective trauma – the psychological upheaval that is shared by a group of people who all experience an event (Aydin, 2017). Collective traumas, which often have long-term effects, pose further challenges for MHPPs to practise their profession, as they are exposed to the same disaster as their patients. Therefore, the clients’ stories, fears and experiences can interact with the professionals’ own stress levels and concerns (Pulido, 2007). While MHPPs have been active in advising clients and the general public during the pandemic, their own needs might have not been addressed, as a result of the nature of their work. In an attempt to formally acknowledge the impact of the pandemic on the wellbeing of psychologists working within the UK, the British Psychological Society developed and shared a set of resources to support and contextualise the wellbeing related impact of COVID-19 on psychologists (British Psychological Society [BPS], 2020). Although it is widely recognised that there will be an increased demand for mental health services in the foreseeable future, research on the effects of the pandemic on frontline workers has mainly focussed on medical professionals and not so much on MHPPs.

It is likely that in such situations MHPPs are expected to demonstrate competencies like integrity and, above all, resilience. Resilience is a dynamic, interactive process which is defined in terms of successful adaptation to the environment in the face of major threat, traumatic experiences or severe adversity (Masten, 2007). In investigating the factors contributing to the amelioration of negative outcomes associated with risk, research has identified various models of resilience. As the main aim of this study was to examine the role of personal characteristics in mediating the relationship between stress and resilience in MHPPs, we adopted the protective model of resilience. This hypothesises that an interactive process between stress and personal qualities modifies the effects of risk and predicts adjustment, changing the outcomes of stress (Garmezy et al., 1984).

Furthermore, the present study adopted the transactional theory of stress (Lazarus and Folkman, 1984), which defines stress as a dynamic interaction between the individual and the environment, to produce an appraisal of a situation or event that subsequently determines coping strategies and results in various negative/positive outcomes. Therefore, according to the model, individuals will make primary appraisals when confronted with stressors and evaluate their relevance, and secondary appraisals when evaluating their own resources to deal with those stressors. Lazarus and Folkman (1984) argue that the variability in how people respond to stressful experiences can be explained by individual differences that can affect cognitive appraisals and coping strategies.

Rooted in the above transactional theory, the job demand resource model (Demerouti et al., 2001) divides working conditions into two categories, reflecting both negative and positive aspects – job demands and job resources. According to this model, when negative aspects are high and positives are low, workers experience burnout and stress. On the other hand, when positive aspects outnumber the negative ones, motivation and engagement are encouraged (Demerouti et al., 2001). This model is useful in understanding how MHPPs experienced stress before the pandemic and how the conditions created by it have affected the way they cope.

Drawing on these theories, the individual factors of self-efficacy and optimism are explored below, especially in relation to how they might link to the ways stress (both pre-pandemic and COVID-19 stress) influences the development of resilience in individuals.

## Self-efficacy and optimism

The relationship between self-efficacy and stress has been widely investigated, with various studies reporting a negative correlation between high general self-efficacy and stress and anxiety (Wang et al., 2001). The underlying premise of self-efficacy is self-regulation of behaviour by cognitive, affective and motivational processes (Wilde and Hsu, 2019). According to this definition, people’s beliefs in their ability to solve problems are positively related to the likelihood of initiating instrumental actions to reach targeted goals. A high level of personal self-efficacy is associated with a positive self-concept and self-appraisal of personal control (Rodriguez and Loos-Sant’Ana, 2015). The latter is a key concept in transactional theory of stress (Lazarus and Folkman, 1984), and has strong links to resilience, especially considering that one of the ways resilience manifests itself in individuals is related to sustained competence under threat (Masten, 2007). Furthermore, secondary appraisal involves assessing one’s own skills in relation to the demands of the situation. Being convinced that one can successfully deal with a situation can change the primary appraisal and reduce the level of stress (Lazarus and Folkman, 1984).

Moreover, self-efficacy can be developed through experiences of mastery and the anticipation of competent performance (Johnston-Wilder et al., 2016). Thus, when faced with stressors, MHPPs with positive self-efficacy are expected to be equipped and prepared for effective action by virtue of their self-confidence and, consequently, demonstrate resilience.

Optimism, the belief that future events will have positive outcomes, has been widely linked to positive outcomes (Carver et al., 2010; Dantzer et al., 2018; Lai, 1995) and has been studied both as a learned skill and a personality trait (e.g. Peterson and Vaidya, 2001). There is a great deal of evidence that optimism enables the individual to set goals, make commitments, cope with adversity and pain, and recover from trauma or stress (Esteve et al., 2018; Fischer and Leitenberg, 1986; Smith, 1983; Tiger, 1979). Consequently, in line with Carver and Scheier (2009), optimism may allow MHPPs to maintain higher levels of wellbeing and mental health during times of stress, rendering them less vulnerable to depression and anxiety. This also links to the transactional model of stress (Lazarus and Folkman, 1984), according to which positive beliefs are a crucial resource for coping. Optimism can predict approach coping with stress as it alters the individual’s cognitive appraisal process. This means that individuals can engage in active, constructive coping, by reframing or reinterpreting adverse experiences (Carver et al., 2010; Nes and Segerstrom, 2006). Hence, the psychological distress experienced by MHPPs is expected to be reduced, as optimists tend to demonstrate more resilience in the face of adversity (Synder and Lopez, 2002).

## Coping strategies

Considering the nature of MHPPs’ work and the fact that resilience arises from the operation of adaptational systems, we tested the mediating role of coping strategies in the relationship between stress and resilience.

Coping is a crucial part of the stress process (Montero-Marin et al., 2014) and it refers to cognitive and behavioural efforts to manage situations perceived as stressful (Folkman and Lazarus, 1986). Throughout the literature, there are several distinctions of coping strategies, including the *focus* of coping strategies, categorising them into approach and avoidant strategies (Carver et al., 1989). Approach coping focuses on active efforts to resolve a stressful situation; on the other hand, avoidant coping is characterised by avoidance of direct confrontation with the stressor. Generally, approach coping has been positively associated with adjustment, better psychological outcomes and lower risk of burnout (Moos, 2002; Thompson et al., 2016). It has been related to resilient individuals, as they usually engage in active coping strategies, such as planning and problem solving (Feder et al., 2009; Li and Nishikawa, 2012). Therefore, active coping strategies have the potential to influence the relationship between stress and resilience in MHPPs. These strategies, in comparison to avoidant coping strategies, have typically been associated with greater ability to deal with stressors and increased wellbeing (Southwick et al., 2005).

## Self-compassion

However, in our effort to gain a deeper understanding of how MHPPs experience stressful situations, we could not ignore the role of self-compassion and professional quality of life, potentially important resources in coping with job-related stress, especially among this group of professionals.

Self-compassion is a learnable skill which involves three components: self-kindness, common humanity and mindfulness (Neff, 2003a, 2003b). These have been suggested to positively influence psychological wellbeing (Neff, 2003b), coping (Neely et al., 2009) and resilience (Gilbert and Procter, 2006) whereas a negative relationship has been identified between self-compassion and psychopathology (MacBeth and Gumley, 2012) and compassion fatigue (Gilbert, 2005).

Self-compassion is viewed as an essential tool in psychological treatment (Figley, 2002) and it is recognised as beneficial for the quality of psychological treatment and the therapists’ well-being and self-care (Raab, 2014). The relationship between self-compassion and stress in mental health professionals is well supported in the literature. Counsellors who practise self-compassion are more capable of managing work stress and challenges; also, cultivation of self-compassion promotes job satisfaction, personal growth, well-being and prevents burnout (Patsiopoulos and Buchanan, 2011). Evidence supports that more self-compassionate student counsellors and student psychotherapists report better well-being and lower compassion fatigue and burnout (Beaumont et al., 2016). Similar results were reported by therapists who participated in Self Compassion interventions (Boellinghaus et al., 2013). Likewise, female trainee psychotherapists who participated in Mindfulness Training noticed benefits in their clinical practice and viewed the training as a way to decrease their stress and develop personally (Dorian and Killebrew, 2014). Furthermore, a study on the effect of Mindfulness-Based Cognitive Therapy on trainee clinical psychologists not only noted significant decreases in stress but in anxiety and rumination too (Rimes and Wingrove, 2011). Taken together, the above evidence suggests that self-compassion encourages the development of several psychological strengths and facilitates resilience by influencing individuals’ reactions to stress.

## Professional quality of life

MHPPs may experience stress related to the responsibilities of their role, which usually exposes them to others’ traumatic experiences (McCann and Pearlman, 1990; Posluns and Gall, 2019). Their role can be emotionally rewarding, as it focuses on helping others. This may lead to compassion satisfaction, that is, positive feelings derived from being able to help (Hansen et al., 2018). However, it may also result in compassion fatigue which involves feelings related to burnout such as exhaustion, hopelessness, or frustration, as well as secondary traumatic stress: sleep problems, intrusions and avoidance symptoms due to being exposed to another person’s trauma (Figley, 1995).

The above positive and negative outcomes contribute to the professional quality of life (Stamm, 2010), as the contentment attained from professional work is critical to the mental health and overall quality of life, especially among mental health practitioners. For example, compassion satisfaction has the potential to reduce compassion fatigue and increase the chance of professionals finding fulfilment in their work (Stamm, 2002). Therefore, it might reduce stress levels and enhance these professionals’ coping resources and strategies.

Compassion satisfaction and compassion fatigue have been studied as an indicator of resilience particularly on health care professions such as nurses (Ang et al., 2018; Flanders et al., 2020), human service providers (Hiles Howard et al., 2015) and medical doctors (McKinley et al., 2020). Very limited research has explored the above relationship in mental health professions. For example, in a sample of disaster behavioural health and emergency preparedness responders, compassion satisfaction was positively associated with resilience whereas compassion fatigue and burnout were negatively associated with resilience (Burnett and Wahl, 2015). A study in New Zealand found that counsellors with low levels of resilience were more likely to experience secondary traumatic stress compared to counsellors with high levels of resilience (Temitope and Williams, 2015). Nevertheless, most evidence is coming from studies evaluating the effectiveness of resilience training or other interventions particularly on health care professions.

## Rationale

MHPPs are likely to be at high risk of experiencing work-related stress because of the nature of their profession which regularly exposes them to trauma. Although the effect of stress on resilience among mental health professionals is well documented (Lee et al., 2019), understanding the effect of significant stress, such as the stress caused by the current COVID-19 pandemic, on the resilience of this group of professionals becomes even more imperative and has yet to be thoroughly explored. Nevertheless, while investigating the effect of stress related with such a crisis, we could not ignore the effects of pre-pandemic perceived stress as they are highly correlated and their synergistic effect has not been assessed by current studies focussing on the effects of COVID-19. Moreover, previous findings have tested resilience as a mediator or predictor variable (e.g. Hiles Howard et al., 2015; Litam et al., 2021) but we treated resilience as an outcome aiming to identify what factors can enhance and cultivate resilience development. This would allow us to make meaningful suggestions for practice. Therefore, based on the protective model of resilience that focuses on the interactive process between stress and personal qualities (Garmezy et al., 1984) and considering the gaps in the relevant research literature, the present study aimed to capture how MHPPs, coped during the pandemic by testing:

1. The concurrent role of pre-pandemic perceived stress and COVID-19 stress in MHPPs’ resilience.
2. The mediating role of individual factors that play a crucial part in the stress process (self-efficacy, optimism, coping strategies) and in coping with job-related stress (self-compassion and professional quality of life) in the relationship between pre-pandemic perceived stress and resilience and COVID-19 stress and resilience.

The inclusion of these specific individual features was informed by the findings of semi-structured interviews with MHPPs conducted before the current investigation, which revealed that personal qualities assisted these professionals in coping with stress and demonstrating resilience. In our effort to understand the relationship between stress and resilience in MHPPs, we focussed on the individual characteristics of self-efficacy and optimism. These are strongly linked to cognitive appraisals, namely the individual’s ability to appraise a stressor as threatening or non-threatening, and decide whether they have the resources to cope with the stressor in an effective way (Lazarus and Folkman, 1984). Moreover, we explored the mediating role of coping strategies in the relationship between stress and resilience, as resilience arises from the operation of adaptational systems, such as coping. Our mediation model also included variables that are particularly important for this specific group of professionals (i.e. self-compassion and professional quality of life) during the COVID-19 pandemic (Litam et al., 2021).

In line with the transactional model of stress (Lazarus and Folkman, 1984) and the job demand resource model (Demerouti et al., 2001), we expected that both perceived pre-pandemic stress and COVID-19 stress will be negatively associated with resilience in MHPPs. According to Lazarus and Folkman (1984), perceptions of stress determine individuals’ responses to stress. Also, as demonstrated by the qualitative data we collected, during COVID-19 MHPPs experienced high job demands which exceeded their existing job resources; according to Demerouti et al. (2001), this kind of excessive demands could lead to higher risk of negative outcomes.

Undoubtedly, COVID-19 changed the nature of MHPPs’ job. Face to face therapy sessions were replaced by tele-therapy, access to resources became limited, whilst their clients experienced additional distress and mental health issues. These changes, the increased workload and job demands, which often lead to experiences of secondary traumatisation, have affected MHPPs wellbeing in a negative manner.

We also hypothesised that the relationship between COVID-19 stress and resilience will be mediated by fewer factors in comparison to the relationship between pre-pandemic stress and resilience. This assumption was based on the nature and duration of stress caused by COVID-19, which is certainly different than stress experienced prior the pandemic. Arguably, the pandemic-related stress differed in terms of controllability and predictability from the stress experienced prior to the pandemic; these characteristics can determine not only the effects of stress, but also how stress is explained (Anisman and Merali, 1999). In terms of duration, it is argued that chronic and persistent stressors, such as COVID-19, have more deleterious effects on the individual. It is argued that uninterrupted and prolonged stressors can both impede positive outcomes and increase negative ones because prolonged exposure to stress requires continuous demands on neurochemical systems, overwhelming the adaptive capacities of the organism (McEwen, 2002). Therefore, different personal characteristics could explain such a relationship compared to the relationship between pre-pandemic stress and resilience.

To conclude, gaining insight into the experiences of MHPPs during this complex historical time holds the potential to assist in the development of programmes, policies and practices which can support this population in dealing with any additional stressors their role entails. In turn, this can potentially contribute to increased motivation, empowerment and personal fulfilment, leading to more positive outcomes for the service users.

## Method

### Sample and procedure

The sample size^1^ was determined using power analysis based on Cohen’s technique (1988) (see Supplemental material for details). Data were collected from 409 participants via an online survey but individuals who completed 50% or less of the survey were omitted from the sample, therefore resulting in an analytical sample of 325 participants. Informed consent was obtained electronically for all participants, and ethics approval was given by the Ethics Committee at Bournemouth University.

We collected data from counsellors, psychologists and psychotherapists who were practising (either face-to-face or online) during the first lockdown in the UK. The link to our anonymous online survey was advertised via social media and professional bodies.

### Measures

*Pre-pandemic* *Perceived Stress* was measured retrospectively with a 10-item self-report questionnaire (Cohen et al., 1983) which, in line with Lazarus and Folkman model (1984), evaluates how unpredictable, uncontrollable and overloaded respondents feel or think their lives are. Participants were asked to rate how often they experienced specific feelings and thoughts in the month before the pandemic on a 5-point Likert scale (0 = never, 4 = very often). In this sample, Cronbach alpha was 0.88.

C*OVID-19 stress* was measured using a version of the Responses to Stress Questionnaire (RSQ; Connor-Smith et al., 2000), a scale that assesses individuals’ involuntary stress reactions to the COVID-19 pandemic (RSQ – COVID-19, Coiro et al., under review). In this study, we used the first part of this scale (14 items). Participants were asked to rate, on a 4-point Likert scale (1 = not at all, 4 = very), how stressful they found the listed stressors over the lockdown period. In this sample Cronbach alpha was 0.83.

*Coping* was measured using the 28-item Brief COPE questionnaire (Carver, 1997). Participants were asked to rate how much they used each coping strategy when under stress during the lockdown on a 4-point Likert scale (1 = I haven’t been doing this at all, 4 = I’ve been doing this a lot). In the analysis we included the subscales of approach and avoidant coping strategies; alpha coefficient was 0.80 for the approach coping subscale and 0.66 for the avoidant coping subscale.

*Self-Compassion* was measured using the Self-Compassion Scale-Short Form (SCS-SF; Raes et al., 2011) which explores the ways individuals respond to failures, feelings of inadequacy, or suffering. Participants were asked to rate 12 statements on a 5-point Likert scale (1 = almost never, 5 = almost always). In this sample Cronbach alpha was 0.87.

*Professional quality of life* was assessed using the Professional Quality of Life Scale version 5 (ProQoL-5; Stamm, 2010) that assesses the two main dimensions of professional quality of life: (a) Compassion Satisfaction (CS) and (b) Compassion Fatigue (CF). Moreover, CF encompasses (1) Secondary Traumatic Stress (STS) and (2) Burnout (BU). Participants were asked to read 30 statements and select the number that represented how ‘frequently they experienced these things in the last 30 days’ on a 5-point Likert scale (1 = never, 5= very often). In this study Cronbach alpha was 0.88 for the CS scale, 0.76 for the STS scale and, 0.75 for the BU scale.

The 10-item Revised Life Orientation Test (LOT-R; Scheier et al., 1994) was utilised to measure *optimism*, by assessing individual differences about positive outcome expectancies. Participants were asked to indicate how much they agreed with each statement on a 5-point Likert scale (1 = strongly disagree, 5 = strongly agree). In our sample internal consistency was 0.85.

*Self-Efficacy* was assessed with the 10-item Generalised Self-Efficacy (GSE) measure (Schwarzer and Jerusalem, 1995). Participants were asked to rate how much they could cope with different statements using a 4-point Likert scale (1 = not at all true, 4 = exactly true). Cronbach alpha for the current study was 0.87.

*Resilience* during the pandemic was assessed using the Connor-Davidson Resilience Scale (CD-RISC; Connor and Davidson, 2003), a 25-item questionnaire that measures capacity to adjust and cope with adversity. Participants were asked to respond to the statements on a 5-point Likert scale (0 = not at all true, 4 = true nearly all the time). Cronbach alpha in the present study was 0.89.

(More details about the measures can be provided by the authors).

## Results

### Socio-demographic characteristics

Our sample consisted mainly of females (80.62%), aged 25–79 years (*M =* 53.17; *SD* = 11.53). About one third of the sample (33.23%; *N* = 108) had a Master’s degree and the years of their work experience as a mental health psychology practitioners ranged from 0.1 to 50 (*M* = 11.96; *SD* = 9.28). The majority of participants were self-employed (75.39%; *N* = 245), worked in private practice (60.62%; *N* = 197) and were in personal psychotherapy or professional supervision (89.23%; *N* = 290). As far as their personal situation was concerned, at the time of data collection most of respondents (75.01%; *N =* 244) were in a relationship (marriage, civil partnership or co-habitation) and lived with one person (42.46%; *N* = 138). Furthermore, most of our participants (64.92%, *N =* 211) lived with no children during the lockdown, and only 65 (20.00%) of them reported having other caring duties such as looking after older parents (a more detailed description of our sample can be found in Supplemental material).

### Mediation analysis

We tested the parallel mediating role of self-efficacy (SE), optimism (LOT), approach (AP) and avoidant (AV) coping strategies, self-compassion (SC), compassion satisfaction (CS), burnout (BU) and secondary traumatic stress (STS), in the relationship between pre-pandemic stress (PSS) and COVID-19 stress (RSQ) and resilience (RES). All calculations were performed in JASP software version 0.13.1 (JASP Team, 2020).^2^

Prior to testing the mediation effects, we ran a series of analyses to determine if mediation was appropriate. First, exploratory correlation analyses indicated medium to strong correlations between all variables excluding associations between AP and PSS (*r* = −0.03, *BF*_10_ = 0.08, 95% CI [0.08, −0.14]); AP and RSQ (*r* = 0.09, *BF*_10_ = 0.22, 95% [0.19, −0.02]), RSQ and CS (*r* = −0.17, *BF*_10_ = 6.76, 95% CI [−0.06, −0.27]), AV and CS (*r* = 0.10, *BF*_10_ = 0.35, 95% CI [0.01, −0.21]) (see details in Supplemental material). Second, we performed a series of regressions analyses to test the relationship among the independent variables, possible mediators and the outcome variable.

### The relationship between pre-pandemic stress (PSS), COVID-19 stress (RSQ) and resilience

Both the PSS and RSQ were associated with the resilience scores (*b* = −0.46, *t*(323) = −9.32, *p* < 0.001; β = −0.26, *t*(323) = −4.92, *p* < 0.001). The Bayesian model selection with the JZS priors (Ly et al., 2016) indicated that a model including both PSS and RSQ as predictors of resilience yielded a higher Bayes Factor (*BF*_10_ = 3.18e+15) compared to models with either predictors (*BF*_10_ = 2.23e+15, *BF*_10_ = 9.5e+3), explaining 22.4% of variance in the resilience scores. The Bayesian model-averaged analysis showed that a one-unit increase in RSQ added about 0.18 units in decreasing resilience. A one-unit increase in PSS added about 0.76 units in decreasing resilience. Therefore, this analysis showed that both PSS and RSQ were associated with resilience scores (see details in Supplemental material).

### The relationship between PSS, RSQ and possible mediators

Separate multiple mediation analyses were performed to test whether PSS and RSQ were associated with possible mediators (AV, AP, SE, LOT, SC, CS, BU and STS). The results of these analyses are summarised in Table 1 (see the full analysis in Supplemental material).

**Table 1.** The relationship between PSS and possible mediators results.

Possible mediators	Predictors		BFincl (PSS; RSQ)
	Pre-pandemic stress	Covid-19 stress	
Avoidance coping	β = 0.25, *t*(323) = 4.84, *p* < 0.001	β = 0.35, *t*(323) = 6.67, *p* < 0.001	14924.35; 1.71e+8
Approach coping	β = −0.06, *t*(323) = −1.07, *p* = 0.28	β = 0.11, *t*(323) = 1.80, *p* = 0.070	0.16; 0.28
Self-efficacy	β = −0.42, *t*(323) = 8.09, *p* < 0.001	β = −0.14, *t*(323) = −2.65, *p* = 0.009	1.31e+12; 6.52
Optimism	β = −0.19, *t*(323) = −3.83, *p* < 0.001	β = −0.39, *t*(323) = −7.49, *p* < 0.001	2.39e+10; 244.39
Self-compassion	β = −0.49, *t*(323) = −9.72, *p* < 0.001	β = −0.12, *t*(323) = −2.41, *p* = 0.02	1.31e+17; 3.34
Burnout	β = 0.37, *t*(323) = 7.25, *p* < 0.001	β = 0.25, *t*(323) = 4.86, *p* < 0.001	5.39e+9; 1.5e+4
Secondary traumatic stress	β = 0.33, *t*(323) = 6.25, *p* < 0.001	β = 0.22, *t*(323) = 4.15, *p* < 0.001	1.67e+7; 865.02
Compassion satisfaction	β = −0.34, *t*(323) = −6.08, *p* < 0.001	β = −0.50, *t*(323) = −0.94, *p* = 0.35	1.93e+7; 0.41

Here we use inclusion Bayes factors which answer the question: Are the observed data more probable under models with a particular effect, than they are under models without that particular effect?

The results in Table 1 indicate that PSS and RSQ were not associated with the AP variable. There was also no evidence that RSQ was associated with CS and weak evidence for the relationship between RSQ and CS.

### The relationship between resilience and possible mediators

A multiple regression was conducted to test whether the possible mediators were associated with the resilience scores. Using enter method it was found that, overall, the mediators accounted for 64.7% of the variance in resilience (*F*(8, 323) = 74.98, *p* < 0.001). The results outcome of this analysis are presented in Table 2. The results in Table 2 indicate weak to medium evidence for the relationship between AV and STS, and resilience scores.

**Table 2.** The relationship between possible mediators (predictors) and resilience (outcome).

Predictors	Parameters	BFincl
Avoidance coping	β = −0.08, *t*(323) = −1.82, *p* = 0.070	3.42
Approach coping	β = 0.15, *t*(323) = 3.85, *p* < 0.001	361.18
Self-efficacy	β = 0.35, *t*(323) = 8.29, *p* < 0.001	9.50e+12
Optimism	β = 0.13, *t*(323) = 2.93, *p* = 0.004	44.16
Self-compassion	β = 0.17, *t*(323) = 3.74, *p* < 0.001	781.19
Burnout	β = −0.20, *t*(323) = −3.50, *p* < 0.001	125.09
Secondary traumatic stress	β = 0.09, *t*(323) = 2.35, *p* = 0.020	7.78
Compassion satisfaction	β = 0.16, *t*(323) = 3.28, *p* < 0.001	94.87

Taken together, the results of the preliminary assessment of the relationship between resilience, stress and potential mediators suggested that both coping strategies (AV and AP) were unlikely to mediate the relationship between PSS, RSQ and resilience and thus they were omitted from the mediation model.

### Mediation model

**Figure 1.**
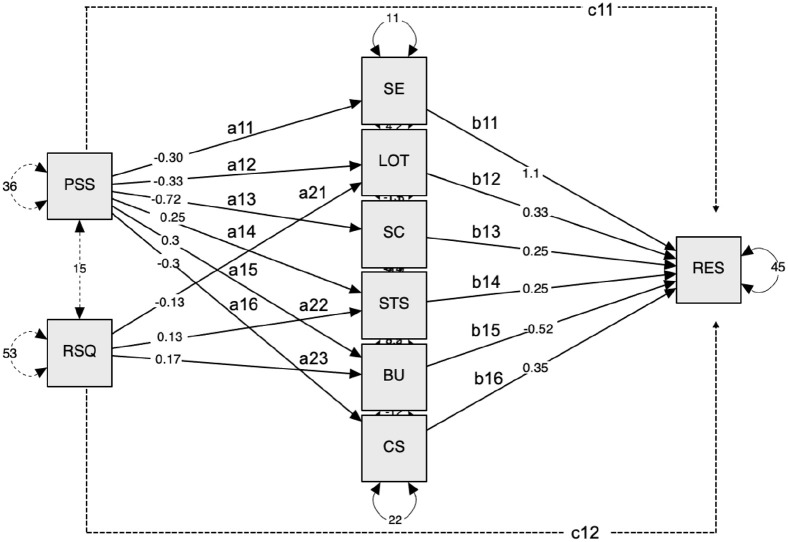
Mediation model. PSS (perceived stress) and RSQ (COVID-related stress) are independent variables; SE (self-efficacy), LOT (optimism), SC (self-compassion), STS (secondary traumatic stress), BU (burnout), CS (compassion satisfaction) are mediators; the RES (resilience) is the dependent variable; a11–a16 and a21–a23 represent the effects of the independent variables on the mediators; b11–b16 represent the effects of the mediators on the dependent variable; c11 and c12 represent the direct effects of PSS and RSQ on the dependent variable. Plain lines outline the main hypothesis tested in each model.

Our initial model included PSS and RSQ as independent variables, resilience as an outcome variable and six variables (self-efficacy, optimism, self-compassion, secondary traumatic stress, burnout and compassion satisfaction) as mediators. Using parallel mediation, we tested each proposed mediator while accounting for the shared variance between them. The path diagram of the mediation model (Figure 1) includes the standardised estimates for the causal paths for the indirect and direct effects. Only statistically significant paths were included in the final model. After introducing indirect paths through the mediators, both direct effects (from PSS to resilience and from RSQ to resilience) were close to zero and non-significant (Table 3).

**Table 3.** Summary of mediation analysis.

Pre-pandemic stress – resilience				
Indirect effects	Est^a^	*z*	*p*	95% CI^b^
Self-efficacy (a11 *×* b11)	−0.32	−6.45	<0.001	[−0.41, −0.22]
Optimism (a12 × b12)	−0.11	−3.12	0.002	[−0.18, −0.04]
Self-compassion (a13 × b13)	−0.18	−4.01	<0.001	[−0.27, −0.09]
Secondary traumatic stress (a14 × b14)	0.06	2.26	0.02	[0.01, 0.12]
Burnout (a15 × b15)	−0.16	−3.60	<0.001	[−0.25, −0.08]
Compassion satisfaction (a16 × b16)	−0.10	−2.99	0.003	[−0.17, −0.04]
Total indirect effect (a1–6 × b1–6)	−0.81	−9.72	<0.001	[−0.98, −0.65]
Total effect (indirect + direct) (a1–6 × b1–6) + c11	−0.83	−8.77	<0.001	[−1.02, −0.65]
Direct effect (c11)	−0.02	−0.19	0.84	[−0.18, 0.15]
Covid-19 stress – resilience				
Indirect effects				
Optimism (a21 × b12)	−0.03	−2.21	0.027	[−0.06, −0.03]
Secondary traumatic stress (a41 × b14)	0.03	2.05	0.040	[0.001, 0.06]
Burnout (a51 × b15)	−0.07	−3.15	0.002	[−0.11, −0.03]
Total indirect effect (a21b12 + a41 × b14 + a51 × b15)	−0.07	−2.63	0.009	[−0.12, −0.02]
Total effect (a21b12 + a41 × b14 + a51 × b15 + c12)	−0.03	−0.48	0.630	[−0.14, 0.09]
Direct effect (c12)	0.04	0.70	0.490	[−0.07, 0.15]

a Est the standardised estimates for the causal paths for the effects.

b 95% CI does not include zero (bias corrected, based on 1000 bootstrap resamples).

The multiple mediator model was fitted using SEM where residuals associated with the mediators were permitted to covary. The model showed a reasonably good model fit according to multiple SEM fit statistics and indices (Root Mean Square Error of Approximation (RMSEA) = 0.049, 95% CI [0.001, 0.10]); Comparative fit index (CFI) = 0.993; Tucker-Lewis index (TLI) = 0.977 (rule of thumb guidelines are that CFI ⩾ 0.95, TLI ⩾ 0.95 and RMSEA ⩽ 0.05 represent a good fitting model) (detailed analyses of the model diagnostics are presented in Supplemental material). The model fit metrics suggested that our theoretically motivated model of the covariance among variables provides a good approximation of the data obtained in this study.

Figure 1 indicates that LOT, STS and BU mediated the relationship between either the PSS and RSQ and resilience. To test the strengths of these mediating effects, we calculated contrasts comparing specific indirect effects of PSS, RSQ on resilience via LOT, STS and BU. The results that are summarised in Table 4 showed that a bias-corrected bootstrapped confidence interval was below zero for the contrasts at LOT and BU, but not at STS. This suggests stronger mediating effects of LOT and BU for the relationship between PSS and resilience compared to the relationship between RSQ and resilience.

**Table 4.** Summary of contrasts analysis.

Mediator	Contrast	Est^a^	*z-*value	*p*	95% CI^b^
Optimisim	(a12 × b12–a21 × b12)	−0.08	−2.19	0.028	[−0.17, −0.02]
Secondary traumatic stress	(a14 × b14–a22 × b14)	0.03	1.57	0.120	[−0.03, 0.07]
Burnout	(a15 × b15–a23 × b15)	−0.10	2.38	0.020	[−0.17, −0.03]

a The standard errors of the parameter estimates were computed using 1000 nonparametric bootstrap samples.

b Bias-corrected and accelerated CI bootstrapped confidence interval with 1000 samples.

## Discussion

This study investigated the relationship between both pre-pandemic stress and COVID-19 stress and resilience, and examined the mediation effects of self-efficacy, optimism, coping strategies, self-compassion and professional quality of life on the relationship between stress and resilience in MHPPs. Three interesting findings emerged. First, in line with the transactional model of stress (Lazarus and Folkman, 1984) and the job demand resource model (Demerouti et al., 2001), both pre-pandemic and COVID-19 stress were negatively linked to resilience. In other words, negative perceptions of stress and increased job demands which outnumbered job resources during the pandemic were negatively associated with MHPPs’ adjustment.

Second, consistently with previous findings (Chiang et al., 2018), it was revealed that avoidant coping was not associated with resilience. Interestingly, both pre-pandemic and COVID-19 stress were not correlated with approach coping. This may be explained by the fact that planning or problem-solving strategies might not be effective due to the uncertainty and limited situational control associated with the unpredictability of COVID-19. This is consistent with research in healthcare workers during COVID-19 demonstrating how problem-solving skills were impaired (Korkmaz et al., 2020). It is also in line with the argument that the lack of control, one of the main elements of COVID-19 (Fu et al., 2020), can inhibit coping and self-regulatory processes (Biggs et al., 2017; Cheng and McCarthy, 2018; Lazarus and Folkman, 1984; Park et al., 2004). A point that needs to be considered is that the effectiveness of coping depends on the context and appraisal; as the COVID-19 pandemic has been an unprecedented situation, in which people have likely experienced uncertainty and lack of control, avoidance coping might had a more functional and adaptive role than expected. Avoidant coping behaviours can be adaptive if a situation is perceived as uncontrollable, as it can act as an effective emotion-focussed coping strategy (Hofmann and Hay, 2018). Furthermore, avoidant coping not being associated with resilience most likely reflects the fact that participants were not adequately prepared or supported to cope with such a crisis (BPS, 2020).

Third, COVID-19 stress was not associated with compassion satisfaction. This may suggest that unprecedented situations, such as COVID-19, are not usually linked to MHPPs’ satisfaction with their abilities to take care of suffering patients possibly because of their belief that their work has a certain degree of social value (Roney and Acri, 2018). However, it should be acknowledged that this investigation only looked at the short-term effects of COVID-19 stress on compassion satisfaction.

In the mediation model only indirect effects were significant, suggesting full mediation. As expected, we found that pre-pandemic stress, in contrast to COVID-19 stress, was mediated by a wider number of factors. Only three variables (optimism, burnout and STS) mediated both the relationship between pre-pandemic stress and resilience and the relationship between COVID-19 stress and resilience. In other words, while certain individual features explained the effect of perceptions about pre-pandemic stress on resilience, they did not affect the relationship between COVID-19 stress and resilience. We could argue that the nature of the stress generated by the pandemic, in contrast to general pre-pandemic stress, could not be processed through the lens of past experiences (Rosen et al., 2020); this may explain the fact that it was not mediated by a wider number of individual factors. Moreover, stress caused by COVID-19 was characterised by uncontrollability, unpredictability and chronicity, characteristics that can determine the effects of stress and thus are crucial in the assessment and understanding of stress (Anisman and Merali, 1999). Previous studies have illustrated that chronic and persistent stressors, such as COVID-19, can have more detrimental effects on the individual than intermittent stressors (e.g. Schoon et al., 2002). Thus, their effects may be explained by different individual resources.

Most importantly, the above finding suggests that optimism, burnout and secondary traumatic stress represent individual variables crucial to understand how MHPPs may be affected by and adapt when exposed to general stress and to a crisis, such as COVID-19. Therefore, prevention strategies should focus on these particular factors.

Optimists tend to perceive stressful events as learning opportunities (Scheier and Carver, 1993) and interpret them in a less threatening way (Arslan et al., 2009), and this attitude provides them with the right confidence level to confront difficulties (Brissette et al., 2002). Learned optimism (Seligman, 2011) has received a lot of attention, not only in research but also in military training strategies (Reivich et al., 2011). This study proposes that learned optimism should be incorporated into the training of MHPPs, for example, they can be trained on how to identify their pessimistic explanatory style and reconstruct their appraisal skills when faced with general stress or unprecedented situations; this would encourage resilience development in this group of professionals. However, it needs to be acknowledged that the nature of a prolonged collective trauma, such as the COVID-19 pandemic, can sometimes make it difficult for individuals to create positive expectancies about the future, when there is confusion, anger and mental distress at a societal level, resulting from the prolonged isolation and disruptions in daily life (Wang et al., 2020b).

On the other hand, our study identified that the two aspects of compassion fatigue, burnout and secondary traumatic stress, are the factors that hinder the development of resilience in MHPPs when dealing with general stress and exceptionally stressful circumstances such as COVID-19. During the first lockdown in the UK demands for mental health care increased massively, resulting in higher exposure to trauma and higher requirements for empathy. These are linked to compassion fatigue symptoms in psychotherapists (Rupert et al., 2015) and consequently, to impaired wellbeing (Galvin and Smith, 2015). Burnout is characterised by exhaustion, hopelessness or frustration, and secondary traumatic stress involves sleep problems, intrusions and avoidance symptoms due to human beings being exposed to another person’s trauma (Stamm, 2010). The occurrence of these symptoms not only suggests that individuals’ capacity to cope has been exceeded, but it may also challenge specific resources, further limiting their ability to show resilience when faced with extremely stressful situations.

These findings highlight the importance of training and supervision practices that allow MHPPs to reflect on their capacity to deal with their workload and job demands under circumstances of general and extreme stress. These procedures would assist professionals in identifying early signs of burnout and secondary traumatic stress (Rupert et al., 2015). They also suggest that, during stressful periods or unprecedented situations, mental health organisations need to monitor their employees’ workload, professional experiences, emotions, beliefs and stress (Rupert and Kent, 2007) in a more attentive way to prevent compassion fatigue.

The conclusions of this study should be evaluated in the light of its limitations. First, although we modelled effects in line with theory and evidence, we assumed a causal path of associations via cross-sectional data. Longitudinal data are needed to exclude other possibilities about the direction of the identified relations. Second, we solely relied on self-reported data and thus the possibility of common source biases must be acknowledged. Thirdly, our mediation model included only specific individual factors; future studies would benefit by the inclusion of other individual factors (i.e. gender, working experience), and processes found within the family system and the community which play a significant role in coping and resilience (Garmezy et al., 1984; Luthar et al., 2000; Masten, 2001). Moreover, we used a convenience sample and the possibility of underrepresentation or overrepresentation of the MHPPs’ population in the UK should be acknowledged. Besides, our study included practitioners who were practising either face-to-face or online during the first lockdown. We should acknowledge that the anxieties, fears and stress of the practitioners who were in direct contact with patients and with COVID-19 cases were entirely different from those practising online and this needs to be taken into consideration when designing interventions to prevent stress and mitigate negative outcomes.

Future research should also consider the way that practitioners experienced online therapy. With the outbreak of COVID-19 MHPPs experienced a sudden transition from face-to-face to online therapy. Research has explored the consequences of such a radical change in psychological treatments among MHPPs; there is evidence that the remote psychological treatment was challenging for the MHPPs due to the technological and usability problems, lack of technological and logistical support, and difficulties in communicating and bonding with their clients (Feijt et al., 2020). Additionally, it should be noted that the majority of our sample had no child or caregiving responsibilities during the first lockdown. Arguably, this may have affected the levels of the stress they experienced during the lockdown, the way they coped with it and subsequently, their resilience (Cheng et al., 2021). Finally, the majority of the participants were self-employed, operating private practices and likely facing increased job and financial insecurity during the lockdown; these factors need to be considered, as they can have detrimental effects to wellbeing and mental health (Llosa et al., 2018). In fact, a study conducted by the National Centre for Social Research (NatCen, 2020), at the beginning of the coronavirus pandemic, suggested that UK workers who faced increased job and financial insecurity have suffered increased mental distress. Considering the above, it cannot be claimed that the findings of this study can be generalised to the wider population of psychologists, psychotherapists and counsellors in the UK.

Despite these limitations, however, it is important not to lose sight of the study’s strengths. The inclusion of specific individual factors in our model was informed by the findings of semi-structured interviews we conducted before the present study. Moreover, data collection started in June 2020, when a lockdown was ongoing in the UK and this enabled us to accurately capture MHPPs’ experiences. Additionally, this study added to the knowledge about the relationship between stress and resilience by highlighting the strong association among three particular personal factors (optimism, burnout and secondary traumatic stress) and resilience in a group of professionals that played and continues to play a significant role in fighting off the negative effects of COVID-19.

Finally, when contrasting the effects of optimism, burnout and STS we found stronger mediating effects of optimism and burnout for the relationship between pre-pandemic stress and resilience compared to the relationship between COVID-19 stress and resilience. COVID-19 is most likely not experienced in the same way as general, pre-pandemic stress because it is characterised by a cumulation of stress-linked repercussions and its effect, according to the allostatic load model (McEwen, 1998), can overwhelm the individual’s adaptive capacities.

In conclusion, our research can have widespread implications for prevention strategies, both within and outside the COVID-19 context. Our findings demonstrated that strategies allowing the development of ‘learned optimism’, as well as detecting burnout and STS symptoms, can reduce the detrimental effects of stress on MHPPs’ resilience levels. Future research should extend these findings by exploring time trajectories and investigating the effect of family and social support in the relationship between both general stress and stress related to unprecedented situations and resilience in MHPPs.

## Supplemental Material

sj-docx-1-hpq-10.1177_13591053211059393 – Supplemental material for Individual factors in the relationship between stress and resilience in mental health psychology practitioners during the COVID-19 pandemic Click here for additional data file. Supplemental material, sj-docx-1-hpq-10.1177_13591053211059393 for Individual factors in the relationship between stress and resilience in mental health psychology practitioners during the COVID-19 pandemic by Constantina Panourgia, Agata Wezyk, Annita Ventouris, Amanda Comoretto, Zoe Taylor and Ala Yankouskaya in Journal of Health Psychology

sj-docx-2-hpq-10.1177_13591053211059393 – Supplemental material for Individual factors in the relationship between stress and resilience in mental health psychology practitioners during the COVID-19 pandemic Click here for additional data file. Supplemental material, sj-docx-2-hpq-10.1177_13591053211059393 for Individual factors in the relationship between stress and resilience in mental health psychology practitioners during the COVID-19 pandemic by Constantina Panourgia, Agata Wezyk, Annita Ventouris, Amanda Comoretto, Zoe Taylor and Ala Yankouskaya in Journal of Health Psychology

sj-html-4-hpq-10.1177_13591053211059393 – Supplemental material for Individual factors in the relationship between stress and resilience in mental health psychology practitioners during the COVID-19 pandemic Click here for additional data file. Supplemental material, sj-html-4-hpq-10.1177_13591053211059393 for Individual factors in the relationship between stress and resilience in mental health psychology practitioners during the COVID-19 pandemic by Constantina Panourgia, Agata Wezyk, Annita Ventouris, Amanda Comoretto, Zoe Taylor and Ala Yankouskaya in Journal of Health Psychology

sj-jasp-3-hpq-10.1177_13591053211059393 – Supplemental material for Individual factors in the relationship between stress and resilience in mental health psychology practitioners during the COVID-19 pandemic Click here for additional data file. Supplemental material, sj-jasp-3-hpq-10.1177_13591053211059393 for Individual factors in the relationship between stress and resilience in mental health psychology practitioners during the COVID-19 pandemic by Constantina Panourgia, Agata Wezyk, Annita Ventouris, Amanda Comoretto, Zoe Taylor and Ala Yankouskaya in Journal of Health Psychology

sj-pdf-5-hpq-10.1177_13591053211059393 – Supplemental material for Individual factors in the relationship between stress and resilience in mental health psychology practitioners during the COVID-19 pandemic Click here for additional data file. Supplemental material, sj-pdf-5-hpq-10.1177_13591053211059393 for Individual factors in the relationship between stress and resilience in mental health psychology practitioners during the COVID-19 pandemic by Constantina Panourgia, Agata Wezyk, Annita Ventouris, Amanda Comoretto, Zoe Taylor and Ala Yankouskaya in Journal of Health Psychology

sj-txt-6-hpq-10.1177_13591053211059393 – Supplemental material for Individual factors in the relationship between stress and resilience in mental health psychology practitioners during the COVID-19 pandemic Click here for additional data file. Supplemental material, sj-txt-6-hpq-10.1177_13591053211059393 for Individual factors in the relationship between stress and resilience in mental health psychology practitioners during the COVID-19 pandemic by Constantina Panourgia, Agata Wezyk, Annita Ventouris, Amanda Comoretto, Zoe Taylor and Ala Yankouskaya in Journal of Health Psychology

## Notes

1. The estimated sample size needed for this study was 159 participants, based on α level of 0.05, power of 0.80, relatively small effect size (0.10) and eight predictors in regression analysis. The convenience sample of the study was tripled to account for attrition rates and ensure reliable effect size in the remaining data.

2. JASP implements R-scripting and Lavaan syntaxis for structural equation modelling (SEM) of mediation effects with multiple mediators (Preacher and Hayes, 2008; VanderWeele and Vansteelandt, 2014).
