# Supplementary material for: Individual factors in the relationship between stress and resilience in mental health psychology practitioners during the COVID-19 pandemic
Source: J Health Psychol. 2021 Dec 7;27(11):2613–31. doi: 10.1177/13591053211059393 (PMC9483698; doi:10.1177/13591053211059393)
Supplement: sj-docx-1-hpq-10.1177_13591053211059393 – Supplemental material for Individual factors in the relationship between stress and resilience in mental health psychology practitioners during the COVID-19 pandemic [file sj-docx-1-hpq-10.1177_13591053211059393.docx]

**Supplementary Material**

**The Role of Individual Factors in the Relationship Between Stress and Resilience in Mental Health Psychology Practitioners During Covid-19**

**Socio-demographic Characteristics: A more detailed description**

The sample consisted of 262 (80.62%) females and 57 (17.54%) males; 6 participants (1.85%) chose not to reveal their gender. Three hundred and nine participants reported their age which ranged from 25 to 79 years (*M* = 53.17; *SD* =11.53). About one third of the sample (33.23%; *N* = 108) had a Master’s degree, 20.92 % (*N* = 68) had a Bachelor’s degree, 14.77% (*N* = 48) had a Doctoral degree, 10.15% (*N* = 33) had a professional degree, and 19.39% (*N* = 63) had other qualifications; the remaining five respondents (1.54%) did not reveal their education level. Their years of experience as a mental health psychology practitioner ranged from 0.1 to 50 (*M* = 11.96; *SD* = 9.28). The majority of participants (75.39%; *N* = 245) were self-employed, while 79 (24.31%) of them worked in paid employment; one person did not answer the question about his/her current employment status. One hundred ninety-seven (60.62%) respondents worked in private practice, another 57 (17.54%) worked in the public sector, and 71 (21.85%) worked in both private and public sectors. A vast majority of them (89.23%; *N* = 290) were in personal psychotherapy or professional supervision.

As far as their personal situation was concerned, at the time of data collection most of respondents (75.01%; *N* = 244) remained in a relationship (marriage, civil partnership, or co-habitation), 11.39% (*N* = 37) were divorced or in the process of separating, 9.85% (*N* = 32) were single, 3.39% (*N* =11) were widowed, and one person (0.31%) did not state his/her marital status. Forty-one (12.62%) respondents lived alone during the lockdown, 138 (42.46%) lived with one person, 122 (37.54%) with 2 or 3 people, and 23 (7.08%) with 4 to 7 people. The majority of participants (64.92%, *N* = 211) lived with no children during the lockdown, 97 (28.85%) lived with 1-2 children, another 16 (4.92%) lived with 3 to 5 children, and one person did not reveal the number of children living with him/her during the lockdown. Sixty-five (20.00%) respondents reported having other caring duties such as looking after older parents.

**Determining Sample Size**

The sample size was calculated using G*Power version 3.1.9.6.

The full regression model for the present study is displayed below:

***RESIL = b_0_*** ***+ b_1_(AV) + b_2_(AP) + b_3_(LOT) + b_4_(SC) + b_5_(CS) + b_6_(BU) + b_7_(STS) + b_8_(SE)***

We believe, from previous research, that the R^2^ for the full-model with eight continuous predictor variables will be will be about 0.5. It is plausible to assume that each of these predictors will add about 0.1 (variance explained by special effect) to the R^2^ when it is added last to the model. The residual variance is defined as 1 – (R^2^ of the full-model), and in this case is 1 – 0.5 = 0.5.  The total number of variables (predictors) is 8 and the number being tested (df) is one.

We ran calculations with power equal to 0.7, 0.8 and 0.9 with steps of 0.01 (Figure S1).

**Figure S1**

*Required Sample Size Depending on Power Calculation*

This gives us a range of sample sizes ranging from 132 (Power = 0.7) to 236 (Power = 0.95).

**Exploratory correlation analysis**

Prior testing our mediation model, we assessed associations between variables in the present study . The results are displayed in Table S1.

| **Table S1**  *Bayesian and Pearson Correlations* | | | | | | | | | | | | | | | | | | | | | | | | | |
| --- | --- | --- | --- | --- | --- | --- | --- | --- | --- | --- | --- | --- | --- | --- | --- | --- | --- | --- | --- | --- | --- | --- | --- | --- | --- |
| **Variable** | |  | | **RESIL** | | **PSS** | | **RSQ** | | **SE** | | **LOT** | | **AV** | | **AP** | | **SC** | | **CS** | | **BU** | | **STS** | |
| 1. RESIL |  | Pearson's r |  | — |  |  |  |  |  |  |  |  |  |  |  |  |  |  |  |  |  |  |  |  |  |
|  |  | BF₁₀ |  | — |  |  |  |  |  |  |  |  |  |  |  |  |  |  |  |  |  |  |  |  |  |
|  |  | Upper 95% CI |  | — |  |  |  |  |  |  |  |  |  |  |  |  |  |  |  |  |  |  |  |  |  |
|  |  | Lower 95% CI |  | — |  |  |  |  |  |  |  |  |  |  |  |  |  |  |  |  |  |  |  |  |  |
| 2. PSS |  | Pearson's r |  | -0.460 | *** | — |  |  |  |  |  |  |  |  |  |  |  |  |  |  |  |  |  |  |  |
|  |  | BF₁₀ |  | 2.688e +15 |  | — |  |  |  |  |  |  |  |  |  |  |  |  |  |  |  |  |  |  |  |
|  |  | Upper 95% CI |  | -0.368 |  | — |  |  |  |  |  |  |  |  |  |  |  |  |  |  |  |  |  |  |  |
|  |  | Lower 95% CI |  | -0.540 |  | — |  |  |  |  |  |  |  |  |  |  |  |  |  |  |  |  |  |  |  |
| 3. RSQ |  | Pearson's r |  | -0.264 | *** | 0.344 | *** | — |  |  |  |  |  |  |  |  |  |  |  |  |  |  |  |  |  |
|  |  | BF₁₀ |  | 7510.235 |  | 4.188e  +7 |  | — |  |  |  |  |  |  |  |  |  |  |  |  |  |  |  |  |  |
|  |  | Upper 95% CI |  | -0.159 |  | 0.435 |  | — |  |  |  |  |  |  |  |  |  |  |  |  |  |  |  |  |  |
|  |  | Lower 95% CI |  | -0.361 |  | 0.243 |  | — |  |  |  |  |  |  |  |  |  |  |  |  |  |  |  |  |  |
| 4. SE |  | Pearson's r |  | 0.657 | *** | -0.467 | *** | -0.282 | *** | — |  |  |  |  |  |  |  |  |  |  |  |  |  |  |  |
|  |  | BF₁₀ |  | 1.553e +38 |  | 1.070e +16 |  | 41349.019 |  | — |  |  |  |  |  |  |  |  |  |  |  |  |  |  |  |
|  |  | Upper 95% CI |  | 0.713 |  | -0.376 |  | -0.178 |  | — |  |  |  |  |  |  |  |  |  |  |  |  |  |  |  |
|  |  | Lower 95% CI |  | 0.588 |  | -0.547 |  | -0.378 |  | — |  |  |  |  |  |  |  |  |  |  |  |  |  |  |  |
| 5. LOT |  | Pearson's r |  | 0.563 | *** | -0.455 | *** | -0.331 | *** | 0.468 | *** | — |  |  |  |  |  |  |  |  |  |  |  |  |  |
|  |  | BF₁₀ |  | 2.480e +25 |  | 1.070e +15 |  | 8.403e  +6 |  | 1.117e +16 |  | — |  |  |  |  |  |  |  |  |  |  |  |  |  |
|  |  | Upper 95% CI |  | 0.631 |  | -0.363 |  | -0.229 |  | 0.547 |  | — |  |  |  |  |  |  |  |  |  |  |  |  |  |
|  |  | Lower 95% CI |  | 0.482 |  | -0.536 |  | -0.423 |  | 0.376 |  | — |  |  |  |  |  |  |  |  |  |  |  |  |  |
| 6. AV |  | Pearson's r |  | -0.305 | *** | 0.367 | *** | 0.430 | *** | -0.334 | *** | -0.275 | *** | — |  |  |  |  |  |  |  |  |  |  |  |
|  |  | BF₁₀ |  | 434967.892 |  | 8.325e  +8 |  | 1.092e +13 |  | 1.138e  +7 |  | 20576.463 |  | — |  |  |  |  |  |  |  |  |  |  |  |
|  |  | Upper 95% CI |  | -0.202 |  | 0.456 |  | 0.513 |  | -0.232 |  | -0.170 |  | — |  |  |  |  |  |  |  |  |  |  |  |
|  |  | Lower 95% CI |  | -0.399 |  | 0.268 |  | 0.335 |  | -0.426 |  | -0.371 |  | — |  |  |  |  |  |  |  |  |  |  |  |
| 7. AP |  | Pearson's r |  | 0.351 | *** | -0.027 |  | 0.085 |  | 0.177 | * | 0.255 | *** | 0.222 | *** | — |  |  |  |  |  |  |  |  |  |
|  |  | BF₁₀ |  | 9.824e  +7 |  | 0.078 |  | 0.220 |  | 11.167 |  | 3354.762 |  | 220.786 |  | — |  |  |  |  |  |  |  |  |  |
|  |  | Upper 95% CI |  | 0.442 |  | 0.082 |  | 0.191 |  | 0.279 |  | 0.353 |  | 0.321 |  | — |  |  |  |  |  |  |  |  |  |
|  |  | Lower 95% CI |  | 0.251 |  | -0.135 |  | -0.024 |  | 0.069 |  | 0.150 |  | 0.115 |  | — |  |  |  |  |  |  |  |  |  |
| 8. SC |  | Pearson's r |  | 0.596 | *** | -0.527 | *** | -0.288 | *** | 0.469 | *** | 0.547 | *** | -0.403 | *** | 0.227 | *** | — |  |  |  |  |  |  |  |
|  |  | BF₁₀ |  | 2.501e +29 |  | 3.673e +21 |  | 73985.916 |  | 1.390e +16 |  | 4.674e +23 |  | 1.413e +11 |  | 326.333 |  | — |  |  |  |  |  |  |  |
|  |  | Upper 95% CI |  | 0.660 |  | -0.442 |  | -0.184 |  | 0.548 |  | 0.618 |  | -0.306 |  | 0.326 |  | — |  |  |  |  |  |  |  |
|  |  | Lower 95% CI |  | 0.519 |  | -0.600 |  | -0.383 |  | 0.378 |  | 0.464 |  | -0.489 |  | 0.120 |  | — |  |  |  |  |  |  |  |
| 9. CS |  | Pearson's r |  | 0.565 | *** | -0.355 | *** | -0.168 |  | 0.408 | *** | 0.334 | *** | -0.101 |  | 0.272 | *** | 0.383 | *** | — |  |  |  |  |  |
|  |  | BF₁₀ |  | 4.548e +25 |  | 1.614e  +8 |  | 6.755 |  | 3.336e +11 |  | 1.277e  +7 |  | 0.354 |  | 14667.284 |  | 8.144e  +9 |  | — |  |  |  |  |  |
|  |  | Upper 95% CI |  | 0.633 |  | -0.254 |  | -0.060 |  | 0.493 |  | 0.426 |  | 0.009 |  | 0.368 |  | 0.471 |  | — |  |  |  |  |  |
|  |  | Lower 95% CI |  | 0.484 |  | -0.445 |  | -0.270 |  | 0.311 |  | 0.233 |  | -0.206 |  | 0.167 |  | 0.285 |  | — |  |  |  |  |  |
| 10. BU |  | Pearson's r |  | -0.621 | *** | 0.455 | *** | 0.375 | *** | -0.451 | *** | -0.494 | *** | 0.325 | *** | -0.249 | *** | -0.528 | *** | -0.666 | *** | — |  |  |  |
|  |  | BF₁₀ |  | 6.272e +32 |  | 1.061e +15 |  | 2.548e  +9 |  | 4.522e +14 |  | 2.041e +18 |  | 4.099e  +6 |  | 1923.237 |  | 4.419e +21 |  | 4.543e +39 |  | — |  |  |  |
|  |  | Upper 95% CI |  | -0.547 |  | 0.536 |  | 0.463 |  | -0.358 |  | -0.405 |  | 0.418 |  | -0.143 |  | -0.443 |  | -0.598 |  | — |  |  |  |
|  |  | Lower 95% CI |  | -0.682 |  | 0.363 |  | 0.276 |  | -0.531 |  | -0.570 |  | 0.223 |  | -0.347 |  | -0.601 |  | -0.721 |  | — |  |  |  |
| 11. STS |  | Pearson's r |  | -0.239 | *** | 0.406 | *** | 0.333 | *** | -0.321 | *** | -0.230 | *** | 0.361 | *** | 4.978e -4 |  | -0.305 | *** | -0.159 |  | 0.468 | *** | — |  |
|  |  | BF₁₀ |  | 858.561 |  | 2.407e +11 |  | 1.053e  +7 |  | 2.745e  +6 |  | 431.443 |  | 3.755e  +8 |  | 0.070 |  | 464121.384 |  | 4.219 |  | 1.292e +16 |  | — |  |
|  |  | Upper 95% CI |  | -0.133 |  | 0.491 |  | 0.425 |  | -0.219 |  | -0.124 |  | 0.451 |  | 0.109 |  | -0.202 |  | -0.051 |  | 0.547 |  | — |  |
|  |  | Lower 95% CI |  | -0.337 |  | 0.309 |  | 0.231 |  | -0.414 |  | -0.329 |  | 0.262 |  | -0.108 |  | -0.399 |  | -0.262 |  | 0.377 |  | — |  |
|  | | | | | | | | | | | | | | | | | | | | | | | | | |

**Regression Model. RSQ and PSS as predictors for Resilience**

A model with PSQ and PSS as predictors could explain 22% of variance in Resilience (See summary of the regression model in Table S2.)

| **Table S2**  *Model Summary* | | | | | | | | | |  |  |  |  |  |  |  |  |  |
| --- | --- | --- | --- | --- | --- | --- | --- | --- | --- | --- | --- | --- | --- | --- | --- | --- | --- | --- |
| Model | | R | | R² | | Adjusted R² | | RMSE | |  |  |  |  |  |  |  |  |  |
| H₀ |  | 0.000 |  | 0.000 |  | 0.000 |  | 11.215 |  |  |  |  |  |  |  |  |  |  |
| H₁ |  | 0.474 |  | 0.224 |  | 0.220 |  | 9.907 |  |  |  |  |  |  |  |  |  |  |
|  | | | | | | | | | |  |  |  |  |  |  |  |  |  |
| ANOVA | | | | | | | | | |  |  |  |  |  |  |  |  |  |
| Model | | |  | | | | Sum of Squares | | | | df | | Mean Square | | F | | p | |
| H₁ | |  | Regression | | |  | 9144.634 | | |  | 2 |  | 4572.317 |  | 46.586 |  | < .001 |  |
|  | |  | Residual | | |  | 31603.594 | | |  | 322 |  | 98.148 |  |  |  |  |  |
|  | |  | Total | | |  | 40748.228 | | |  | 324 |  |  |  |  |  |  |  |
|  | | | | | | | | | | | | | | | | | | |
| *Note.*  The intercept model is omitted, as no meaningful information can be shown. | | | | | | | | | | | | | | | | | | |
| \| Coefficients \| \| \| \| \| \| \| \| \| \| \| \| \| \| \| \| \| \| \| --- \| --- \| --- \| --- \| --- \| --- \| --- \| --- \| --- \| --- \| --- \| --- \| --- \| --- \| --- \| --- \| --- \| --- \| \|  \| \| \| \| \| \| \| \| \| \| \| \| \| \| 95% CI \| \| \| \| \| Model \| \|  \| \| Unstandardized \| \| Standard Error \| \| Standardized \| \| t \| \| p \| \| Lower \| \| Upper \| \| \| H₀ \|  \| (Intercept) \|  \| 0.004 \|  \| 0.622 \|  \|  \|  \| 0.006 \|  \| 0.995 \|  \| -1.220 \|  \| 1.228 \|  \| \| H₁ \|  \| (Intercept) \|  \| 0.007 \|  \| 0.550 \|  \|  \|  \| 0.013 \|  \| 0.990 \|  \| -1.074 \|  \| 1.088 \|  \| \|  \|  \| RSQ \|  \| -0.184 \|  \| 0.080 \|  \| -0.120 \|  \| -2.292 \|  \| 0.023 \|  \| -0.342 \|  \| -0.026 \|  \| \|  \|  \| PSS \|  \| -0.779 \|  \| 0.097 \|  \| -0.419 \|  \| -8.016 \|  \| < .001 \|  \| -0.970 \|  \| -0.588 \|  \| \|  \| \| \| \| \| \| \| \| \| \| \| \| \| \| \| \| \| \| | | | | | | | | | | | | | | | | | | |

However, the coefficient of determination R^2^ is not a good measure for model comparison because it does not penalize models for complexity: when additional predictors are added to a model, R^2^ can only increase. Therefore, R^2^ will always favour the most complex model. This makes R^2^ unsuitable for model selection, unless models have the same number of predictors. Therefore, we will use Bayesian Inference for model selection.

As we have no previous studies that could indicate us prior believes, we will assign default priors (i.e., the relative plausibility of models before seeing the data) . The change from prior to posterior odds (i.e., the relative plausibility of models after seeing the data) is given by the Bayes factor (e.g., Jeffreys, 1961; Kass & Raftery, 1995), which indicates the models’ relative predictive performance for the data at hand (i.e., the ratio of marginal likelihoods). Using default priors:  we used the Jeffreys–Zellner–Siow (JZS) prior. The JZS prior fulfils several desiderata (Rouder & Morey, 2012;  Ly et al., 2016). Moreover, the sample size for our data is 324, which is relatively big. In the case of the big dataset, the influence of the prior is relatively small. As we have only 2 predictors, we will compare three possible models (PSS), (RSQ) and (PSS+RSQ) with the null model (see summary in Table S3).

| **Table S3**  *Model Comparison* | | | | | | | | | | | |
| --- | --- | --- | --- | --- | --- | --- | --- | --- | --- | --- | --- |
| Models | | P(M) | | P(M\|data) | | BF _M_ | | BF _10_ | | R² | |
| Null model |  | 0.250 |  | 1.849e -16 |  | 5.547e -16 |  | 1.000 |  | 0.000 |  |
| RSQ + PSS |  | 0.250 |  | 0.587 |  | 4.266 |  | 3.175e +15 |  | 0.224 |  |
| PSS |  | 0.250 |  | 0.413 |  | 2.110 |  | 2.233e +15 |  | 0.212 |  |
| RSQ |  | 0.250 |  | 1.757e -12 |  | 5.270e -12 |  | 9501.186 |  | 0.070 |  |
|  | | | | | | | | | | | |

The Bayes factor BF_10_ (H1) for model with both predictors is large and indicates that the data are 3.15e+15 times more likely under (RSQ + PSS) model compared to the null model (H0). However, the Bayes factor for two other models with only one predictor (PSS or RSQ) is also large (BF10 >100) that indicated strong evidence in favour of the H1 hypothesis. Therefore, the model comparison statistics are not informative in this case. We will then asses the Posterior Summary table (Table S4) which quantifies the relevance of individual predictors.

| **Table S4**  *Posterior Summaries of Coefficients* | | | | | | | | | | | | | | | |
| --- | --- | --- | --- | --- | --- | --- | --- | --- | --- | --- | --- | --- | --- | --- | --- |
|  | | | | | | | | | | | | 95% Credible Interval | | | |
| Coefficient | | Mean | | SD | | P(incl) | | P(incl\|data) | | BF _inclusion_ | | Lower | | Upper | |
| Intercept |  | 0.004 |  | 0.550 |  | 1.000 |  | 1.000 |  | 1.000 |  | -1.077 |  | 1.085 |  |
| RSQ |  | -0.180 |  | 0.079 |  | 0.500 |  | 0.587 |  | 1.422 |  | -0.336 |  | -0.024 |  |
| PSS |  | -0.762 |  | 0.096 |  | 0.500 |  | 1.000 |  | 5.692e +11 |  | -0.951 |  | -0.573 |  |
|  | | | | | | | | | | | | | | | |

*Note*.Mean and SD - represent the respective posterior mean and standard deviation of the parameter after model averaging

P (incl) denotes the prior inclusion probability

P (incl | data) denotes the posterior inclusion probability

BFinclusion - the change from prior to posterior inclusion odds

a 95% central credible interval (CI) for the parameters

The results indicate that each of these predictors are relevant for predicting resilience, as indicated by the fact that the posterior inclusion probabilities for PSS is =1.00 and for RSQ = 0.74. Although, the posterior inclusion probability for RSQ is not as high as we would expect*, the relevance of this predictor is evident because the data increased the inclusion probability from 0.5 to 0.74 (see Inclusion Probabilities plot below. The dashed line represents the prior inclusion probabilities ).

*Inclusion Probabilities*


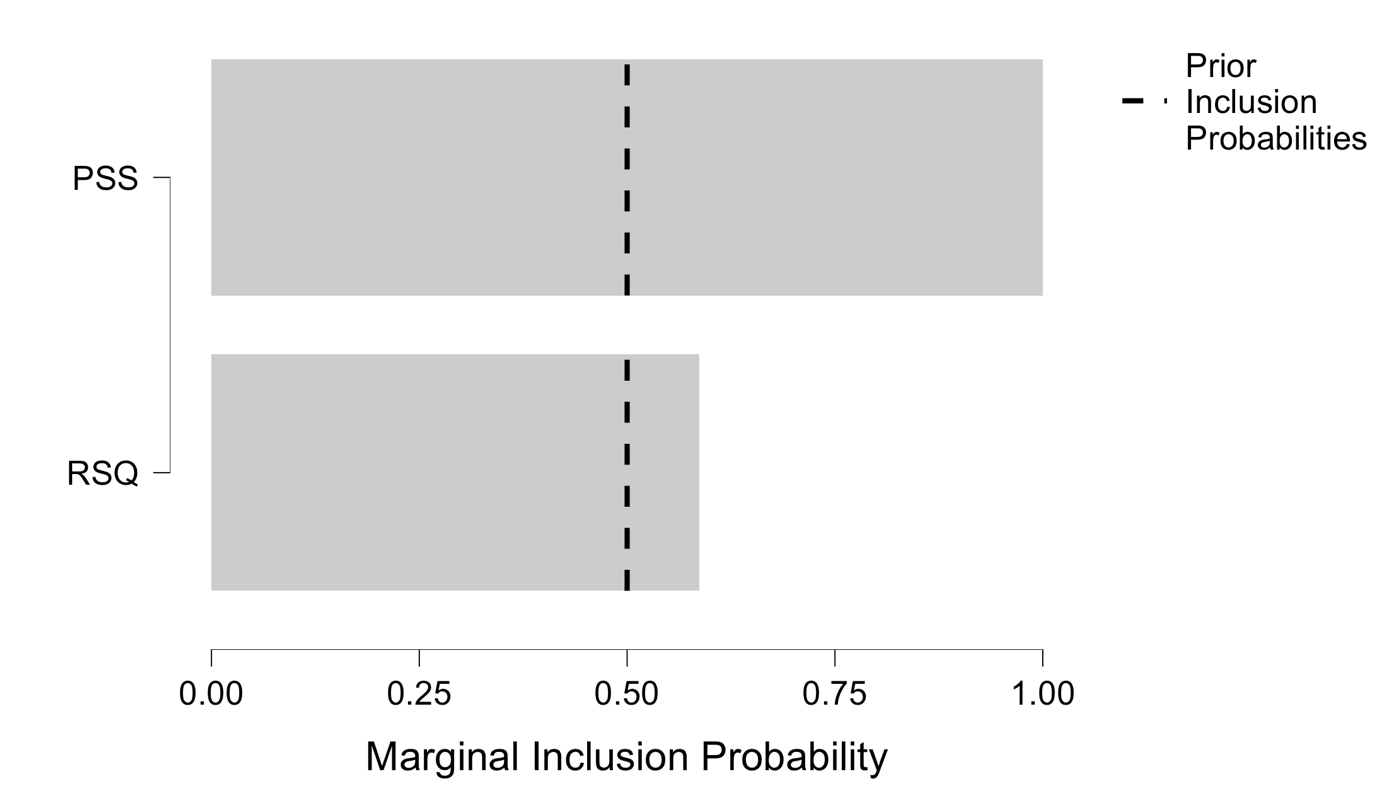


*The inclusion Bayes factor quantifies how much the observed data are more probable under models that include a particular predictor relative to the models that do not contain that particular predictor.  In case of RSQ, across all the candidate models, the model with the RSQ variable is, on average, about only 1.42 times more likely than the model without the RSQ variable.

The Q-Q plot (below) shows that the standardized residuals fit fairly well along the diagonal suggesting that both assumptions or normality and linearity have also not been violated.

*Q-Q Plot*


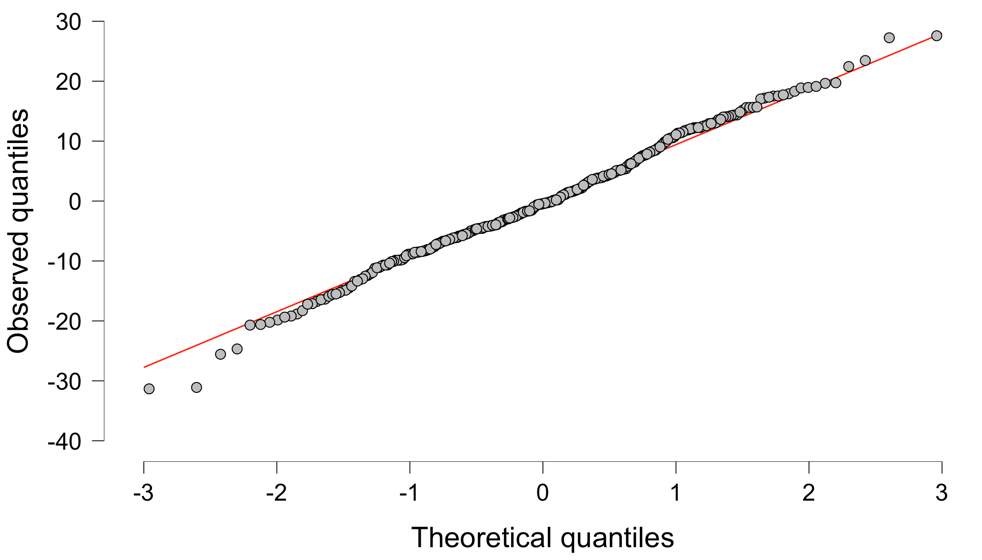


### Marginal Posterior Distributions

The complete model-averaged posteriors are be visualized below. The pike at zero in RSQ plot corresponds to the absence of an effect, and its height reflects the predictor’s posterior exclusion probability. The horizontal bars depicted a 95% credible interval for each predictor. Following recommendations by van Doorn at al (2019), we further investigated the robustness of the results against the choice of prior by using wide and ultrawide priors. Therefore, we repeated this analysis using scale of 1/4 and 1/2 but the result did not change in a meaningful way in both cases.

 Intercept RSQ


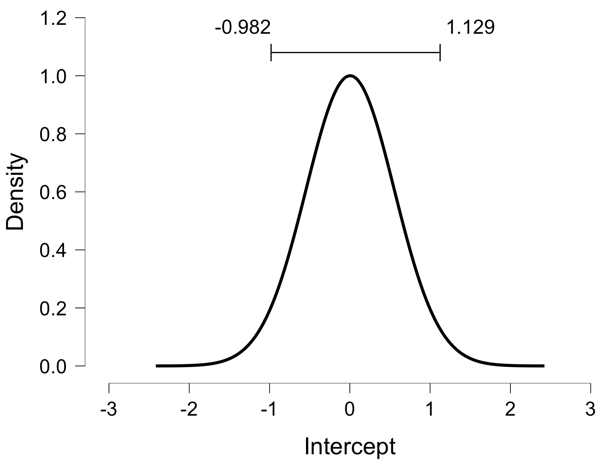

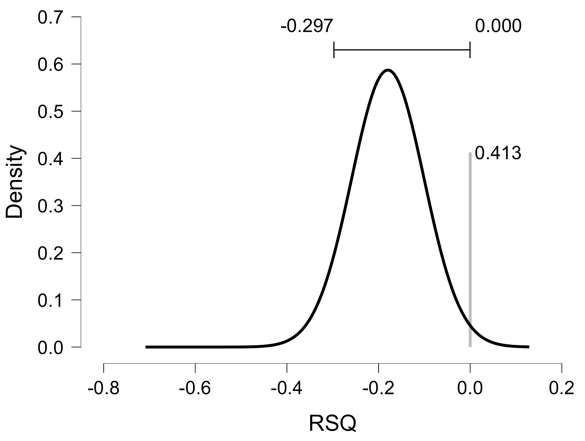


#### PSS


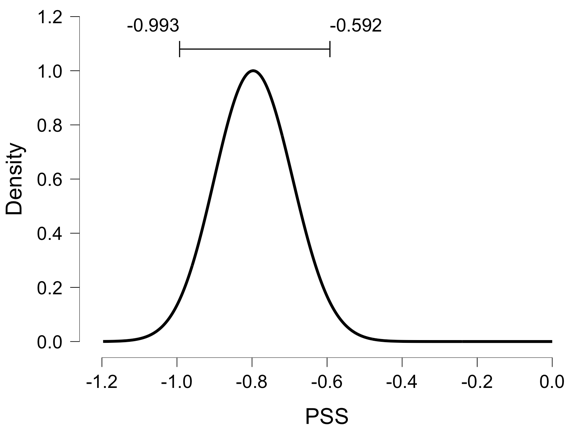


To summarize, the Bayesian model-averaged analysis showed that a one-unit increase in RSQ adds about 0.18 units in decreasing resilience. A one-unit increase in PSS adds about 0.76 units in decreasing resilience. Therefore, this analysis shows the evidence of the effects of both PSS and RSQ in predicting the level of resilience.

**The relationship between PSS, RSQ and possible mediators**

We next tested the relationship between either predictors (RSQ and PSS) and potential mediators (i.e., AV, AP, SE, LOT, SC, STS, BU, CS) We, first, tested whether RSQ and PSS could predict AV, AP, SE, LOT, SC, STS, BU, CS using a multiple regression analysis to gather an idea which independent variables will create the best prediction equation. We performed regression analyses with each of possible mediators as a dependent variable and RSQ and PSS as predictors. Each analysis we supplemented with Bayesian inferences to gather evidence of each prediction.

In summary, the analyses below showed that:

1. RSQ and PSS are not reliable predictors of AP
2. RSQ is not reliable predictors of SC and CS while PSS could reliably predict these variables

| *Model Summary - SE* | | | | | | | | | | | | | | | | | | | | | | | | | | |
| --- | --- | --- | --- | --- | --- | --- | --- | --- | --- | --- | --- | --- | --- | --- | --- | --- | --- | --- | --- | --- | --- | --- | --- | --- | --- | --- |
|  | | | | | | | | | | | | | | | | | | | | Durbin-Watson | | | | | | |
| Model | | R | | R² | | Adjusted R² | | RMSE | | R² Change | | F Change | | df1 | | df2 | | p | | Autocorrelation | | | Statistic | | p | |
| H₀ |  | 0.000 |  | 0.000 |  | 0.000 |  | 3.728 |  | 0.000 |  |  |  | 0 |  | 324 |  |  |  | -0.049 | |  | 2.098 |  | 0.374 |  |
| H₁ |  | 0.485 |  | 0.235 |  | 0.230 |  | 3.270 |  | 0.235 |  | 49.503 |  | 2 |  | 322 |  | < .001 |  | -0.059 | |  | 2.116 |  | 0.296 |  |
|  | | | | | | | | | | | | | | | | | | | | | | | | | | |
| ANOVA | | | | | | | | | | | | | | | | | | | | | | | | | | |
| Model | | |  | | | | Sum of Squares | | | | | | df | | Mean Square | | | | | | | F | | p | |  |
| H₁ | |  | Regression | | |  | 1058.871 | | | | |  | 2 |  | 529.436 | | | | |  | | 49.503 |  | < .001 |  |  |
|  | |  | Residual | | |  | 3443.818 | | | | |  | 322 |  | 10.695 | | | | |  | |  |  |  |  |  |
|  | |  | Total | | |  | 4502.689 | | | | |  | 324 |  |  | | | | |  | |  |  |  |  |  |
|  | | | | | | | | | | | | | | | | | | | | | | | | | |  |
| *Note.*  The intercept model is omitted, as no meaningful information can be shown. | | | | | | | | | | | | | | | | | | | | | | | | | |  |
| \| Coefficients \| \| \| \| \| \| \| \| \| \| \| \| \| \| \| \| \| \| \| \| \| \| \| --- \| --- \| --- \| --- \| --- \| --- \| --- \| --- \| --- \| --- \| --- \| --- \| --- \| --- \| --- \| --- \| --- \| --- \| --- \| --- \| --- \| --- \| \|  \| \| \| \| \| \| \| \| \| \| \| \| \| \| 95% CI \| \| \| \| Collinearity Statistics \| \| \| \| \| Model \| \|  \| \| Unstandardized \| \| Standard Error \| \| Standardized \| \| t \| \| p \| \| Lower \| \| Upper \| \| Tolerance \| \| VIF \| \| \| H₀ \|  \| (Intercept) \|  \| 0.018 \|  \| 0.207 \|  \|  \|  \| 0.089 \|  \| 0.929 \|  \| -0.388 \|  \| 0.425 \|  \|  \|  \|  \|  \| \| H₁ \|  \| (Intercept) \|  \| 0.020 \|  \| 0.181 \|  \|  \|  \| 0.108 \|  \| 0.914 \|  \| -0.337 \|  \| 0.376 \|  \|  \|  \|  \|  \| \|  \|  \| PSS \|  \| -0.260 \|  \| 0.032 \|  \| -0.420 \|  \| -8.095 \|  \| < .001 \|  \| -0.323 \|  \| -0.197 \|  \| 0.882 \|  \| 1.134 \|  \| \|  \|  \| RSQ \|  \| -0.070 \|  \| 0.026 \|  \| -0.137 \|  \| -2.647 \|  \| 0.009 \|  \| -0.122 \|  \| -0.018 \|  \| 0.882 \|  \| 1.134 \|  \| \|  \| \| \| \| \| \| \| \| \| \| \| \| \| \| \| \| \| \| \| \| \| \| | | | | | | | | | | | | | | | | | | | | | | | | | |  |
|  | | | | | | | | | | | | | | | | | | | | |  |  |  |  |  |  |

| Model Summary - AV | | | | | | | | | |  |  |  |  |  |  |  |  |  |
| --- | --- | --- | --- | --- | --- | --- | --- | --- | --- | --- | --- | --- | --- | --- | --- | --- | --- | --- |
| Model | | R | | R² | | Adjusted R² | | RMSE | |  |  |  |  |  |  |  |  |  |
| H₀ |  | 0.000 |  | 0.000 |  | 0.000 |  | 4.084 |  |  |  |  |  |  |  |  |  |  |
| H₁ |  | 0.490 |  | 0.240 |  | 0.236 |  | 3.570 |  |  |  |  |  |  |  |  |  |  |
|  | | | | | | | | | |  |  |  |  |  |  |  |  |  |
| ANOVA | | | | | | | | | |  |  |  |  |  |  |  |  |  |
| Model | | |  | | | | Sum of Squares | | | | df | | Mean Square | | F | | p | |
| H₁ | |  | Regression | | |  | 1294.350 | | |  | 2 |  | 647.175 |  | 50.766 |  | < .001 |  |
|  | |  | Residual | | |  | 4092.206 | | |  | 321 |  | 12.748 |  |  |  |  |  |
|  | |  | Total | | |  | 5386.556 | | |  | 323 |  |  |  |  |  |  |  |
|  | | | | | | | | | | | | | | | | | | |
| *Note.*  The intercept model is omitted, as no meaningful information can be shown.   \| Coefficients \| \| \| \| \| \| \| \| \| \| \| \| \| \| \| \| \| \| \| \| \| \| \| --- \| --- \| --- \| --- \| --- \| --- \| --- \| --- \| --- \| --- \| --- \| --- \| --- \| --- \| --- \| --- \| --- \| --- \| --- \| --- \| --- \| --- \| \|  \| \| \| \| \| \| \| \| \| \| \| \| \| \| 95% CI \| \| \| \| Collinearity Statistics \| \| \| \| \| Model \| \|  \| \| Unstandardized \| \| Standard Error \| \| Standardized \| \| t \| \| p \| \| Lower \| \| Upper \| \| Tolerance \| \| VIF \| \| \| H₀ \|  \| (Intercept) \|  \| 0.004 \|  \| 0.227 \|  \|  \|  \| 0.016 \|  \| 0.987 \|  \| -0.443 \|  \| 0.450 \|  \|  \|  \|  \|  \| \| H₁ \|  \| (Intercept) \|  \| -0.010 \|  \| 0.198 \|  \|  \|  \| -0.052 \|  \| 0.959 \|  \| -0.400 \|  \| 0.380 \|  \|  \|  \|  \|  \| \|  \|  \| PSS \|  \| 0.170 \|  \| 0.035 \|  \| 0.250 \|  \| 4.844 \|  \| < .001 \|  \| 0.101 \|  \| 0.239 \|  \| 0.885 \|  \| 1.130 \|  \| \|  \|  \| RSQ \|  \| 0.193 \|  \| 0.029 \|  \| 0.345 \|  \| 6.670 \|  \| < .001 \|  \| 0.136 \|  \| 0.250 \|  \| 0.885 \|  \| 1.130 \|  \| \|  \| \| \| \| \| \| \| \| \| \| \| \| \| \| \| \| \| \| \| \| \| \| | | | | | | | | | | | | | | | | | | |

| Model Summary - AP | | | | | | | | | |  |  |  |  |  |  |  |  |  |  |  |  |  |  |  |
| --- | --- | --- | --- | --- | --- | --- | --- | --- | --- | --- | --- | --- | --- | --- | --- | --- | --- | --- | --- | --- | --- | --- | --- | --- |
| Model | | R | | R² | | Adjusted R² | | RMSE | |  |  |  |  |  |  |  |  |  |  |  |  |  |  |  |
| H₀ |  | 0.000 |  | 0.000 |  | 0.000 |  | 5.845 |  |  |  |  |  |  |  |  |  |  |  |  |  |  |  |  |
| H₁ |  | 0.103 |  | 0.011 |  | 0.005 |  | 5.831 |  |  |  |  |  |  |  |  |  |  |  |  |  |  |  |  |
|  | | | | | | | | | |  |  |  |  |  |  |  |  |  |  |  |  |  |  |  |
| ANOVA | | | | | | | | | |  |  |  |  |  |  |  |  |  |  |  |  |  |  |  |
| Model | | |  | | | | Sum of Squares | | | | df | | Mean Square | | | F | | p | |  |  |  |  |  |
| H₁ | |  | Regression | | |  | 118.189 | | |  | 2 |  | 59.094 | |  | 1.738 |  | 0.178 |  |  |  |  |  |  |
|  | |  | Residual | | |  | 10915.836 | | |  | 321 |  | 34.006 | |  |  |  |  |  |  |  |  |  |  |
|  | |  | Total | | |  | 11034.025 | | |  | 323 |  |  | |  |  |  |  |  |  |  |  |  |  |
|  | | | | | | | | | | | | | | | | | | | |  |  |  |  |  |
| Coefficients | | | | | | | | | | | | | | | | | | | |  |  |  |  |  |
|  | | | | | | | | | | | | | | | | | | | | | 95% CI | | | |
| Model | | |  | | Unstandardized | | | | Standard Error | | | Standardized | | | | t | | | p | | Lower | | Upper | |
| H₀ | |  | (Intercept) |  | 0.004 | | |  | 0.325 | |  |  | |  | | 0.013 | |  | 0.990 |  | -0.635 |  | 0.643 |  |
| H₁ | |  | (Intercept) |  | 0.002 | | |  | 0.324 | |  |  | |  | | 0.006 | |  | 0.995 |  | -0.636 |  | 0.639 |  |
|  | |  | PSS |  | -0.061 | | |  | 0.057 | |  | -0.063 | |  | | -1.072 | |  | 0.284 |  | -0.174 |  | 0.051 |  |
|  | |  | RSQ |  | 0.085 | | |  | 0.047 | |  | 0.106 | |  | | 1.798 | |  | 0.073 |  | -0.008 |  | 0.178 |  |
|  | | | | | | | | | | | | | | | | | | | | | | | | |

| Model Summary - LOT | | | | | | | | | |  |  |  |  |  |  |  |  |  |
| --- | --- | --- | --- | --- | --- | --- | --- | --- | --- | --- | --- | --- | --- | --- | --- | --- | --- | --- |
| Model | | R | | R² | | Adjusted R² | | RMSE | |  |  |  |  |  |  |  |  |  |
| H₀ |  | 0.000 |  | 0.000 |  | 0.000 |  | 4.931 |  |  |  |  |  |  |  |  |  |  |
| H₁ |  | 0.492 |  | 0.242 |  | 0.237 |  | 4.307 |  |  |  |  |  |  |  |  |  |  |
|  | | | | | | | | | |  |  |  |  |  |  |  |  |  |
| ANOVA | | | | | | | | | |  |  |  |  |  |  |  |  |  |
| Model | | |  | | | | Sum of Squares | | | | df | | Mean Square | | F | | p | |
| H₁ | |  | Regression | | |  | 1903.710 | | |  | 2 |  | 951.855 |  | 51.310 |  | < .001 |  |
|  | |  | Residual | | |  | 5973.478 | | |  | 322 |  | 18.551 |  |  |  |  |  |
|  | |  | Total | | |  | 7877.188 | | |  | 324 |  |  |  |  |  |  |  |
|  | | | | | | | | | | | | | | | | | | |
| *Note.*  The intercept model is omitted, as no meaningful information can be shown. | | | | | | | | | | | | | | | | | | |

| Coefficients | | | | | | | | | | | | | | | | | | | | | |
| --- | --- | --- | --- | --- | --- | --- | --- | --- | --- | --- | --- | --- | --- | --- | --- | --- | --- | --- | --- | --- | --- |
|  | | | | | | | | | | | | | | 95% CI | | | | Collinearity Statistics | | | |
| Model | |  | | Unstandardized | | Standard Error | | Standardized | | t | | p | | Lower | | Upper | | Tolerance | | VIF | |
| H₀ |  | (Intercept) |  | 0.006 |  | 0.274 |  |  |  | 0.022 |  | 0.982 |  | -0.532 |  | 0.544 |  |  |  |  |  |
| H₁ |  | (Intercept) |  | 0.008 |  | 0.239 |  |  |  | 0.033 |  | 0.974 |  | -0.462 |  | 0.478 |  |  |  |  |  |
|  |  | RSQ |  | -0.133 |  | 0.035 |  | -0.198 |  | -3.825 |  | < .001 |  | -0.202 |  | -0.065 |  | 0.882 |  | 1.134 |  |
|  |  | PSS |  | -0.317 |  | 0.042 |  | -0.387 |  | -7.492 |  | < .001 |  | -0.400 |  | -0.233 |  | 0.882 |  | 1.134 |  |
|  | | | | | | | | | | | | | | | | | | | | | |

| Model Summary - SC | | | | | | | | | |
| --- | --- | --- | --- | --- | --- | --- | --- | --- | --- |
| Model | | R | | R² | | Adjusted R² | | RMSE | |
| H₀ |  | 0.000 |  | 0.000 |  | 0.000 |  | 8.891 |  |
| H₁ |  | 0.539 |  | 0.291 |  | 0.287 |  | 7.510 |  |
|  | | | | | | | | | |

| ANOVA | | | | | | | | | | | | | |
| --- | --- | --- | --- | --- | --- | --- | --- | --- | --- | --- | --- | --- | --- |
| Model | |  | | Sum of Squares | | df | | Mean Square | | F | | p | |
| H₁ |  | Regression |  | 7453.696 |  | 2 |  | 3726.848 |  | 66.082 |  | < .001 |  |
|  |  | Residual |  | 18159.892 |  | 322 |  | 56.397 |  |  |  |  |  |
|  |  | Total |  | 25613.588 |  | 324 |  |  |  |  |  |  |  |
|  | | | | | | | | | | | | | |
| *Note.*  The intercept model is omitted, as no meaningful information can be shown. | | | | | | | | | | | | | |

| Coefficients | | | | | | | | | | | | | | | | | | | | | |
| --- | --- | --- | --- | --- | --- | --- | --- | --- | --- | --- | --- | --- | --- | --- | --- | --- | --- | --- | --- | --- | --- |
|  | | | | | | | | | | | | | | 95% CI | | | | Collinearity Statistics | | | |
| Model | |  | | Unstandardized | | Standard Error | | Standardized | | t | | p | | Lower | | Upper | | Tolerance | | VIF | |
| H₀ |  | (Intercept) |  | 0.006 |  | 0.493 |  |  |  | 0.012 |  | 0.990 |  | -0.964 |  | 0.976 |  |  |  |  |  |
| H₁ |  | (Intercept) |  | 0.009 |  | 0.417 |  |  |  | 0.021 |  | 0.983 |  | -0.811 |  | 0.828 |  |  |  |  |  |
|  |  | PSS |  | -0.716 |  | 0.074 |  | -0.486 |  | -9.723 |  | < .001 |  | -0.861 |  | -0.571 |  | 0.882 |  | 1.134 |  |
|  |  | RSQ |  | -0.147 |  | 0.061 |  | -0.121 |  | -2.414 |  | 0.016 |  | -0.266 |  | -0.027 |  | 0.882 |  | 1.134 |  |
|  | | | | | | | | | | | | | | | | | | | | | |

| Model Summary - CS | | | | | | | | | |
| --- | --- | --- | --- | --- | --- | --- | --- | --- | --- |
| Model | | R | | R² | | Adjusted R² | | RMSE | |
| H₀ |  | 0.000 |  | 0.000 |  | 0.000 |  | 5.033 |  |
| H₁ |  | 0.358 |  | 0.128 |  | 0.123 |  | 4.714 |  |
|  | | | | | | | | | |

| ANOVA | | | | | | | | | | | | | |
| --- | --- | --- | --- | --- | --- | --- | --- | --- | --- | --- | --- | --- | --- |
| Model | |  | | Sum of Squares | | df | | Mean Square | | F | | p | |
| H₁ |  | Regression |  | 1051.307 |  | 2 |  | 525.654 |  | 23.653 |  | < .001 |  |
|  |  | Residual |  | 7156.096 |  | 322 |  | 22.224 |  |  |  |  |  |
|  |  | Total |  | 8207.403 |  | 324 |  |  |  |  |  |  |  |
|  | | | | | | | | | | | | | |
| *Note.*  The intercept model is omitted, as no meaningful information can be shown. | | | | | | | | | | | | | |

| Coefficients | | | | | | | | | | | | | | | | | | | | | |
| --- | --- | --- | --- | --- | --- | --- | --- | --- | --- | --- | --- | --- | --- | --- | --- | --- | --- | --- | --- | --- | --- |
|  | | | | | | | | | | | | | | 95% CI | | | | Collinearity Statistics | | | |
| Model | |  | | Unstandardized | | Standard Error | | Standardized | | t | | p | | Lower | | Upper | | Tolerance | | VIF | |
| H₀ |  | (Intercept) |  | 0.005 |  | 0.279 |  |  |  | 0.019 |  | 0.985 |  | -0.544 |  | 0.555 |  |  |  |  |  |
| H₁ |  | (Intercept) |  | 0.006 |  | 0.261 |  |  |  | 0.024 |  | 0.981 |  | -0.508 |  | 0.521 |  |  |  |  |  |
|  |  | PSS |  | -0.281 |  | 0.046 |  | -0.337 |  | -6.076 |  | < .001 |  | -0.372 |  | -0.190 |  | 0.882 |  | 1.134 |  |
|  |  | RSQ |  | -0.036 |  | 0.038 |  | -0.052 |  | -0.935 |  | 0.350 |  | -0.111 |  | 0.039 |  | 0.882 |  | 1.134 |  |
|  | | | | | | | | | | | | | | | | | | | | | |

| Model Summary - BU | | | | | | | | | |
| --- | --- | --- | --- | --- | --- | --- | --- | --- | --- |
| Model | | R | | R² | | Adjusted R² | | RMSE | |
| H₀ |  | 0.000 |  | 0.000 |  | 0.000 |  | 4.915 |  |
| H₁ |  | 0.511 |  | 0.261 |  | 0.257 |  | 4.237 |  |
|  | | | | | | | | | |

| ANOVA | | | | | | | | | | | | | |
| --- | --- | --- | --- | --- | --- | --- | --- | --- | --- | --- | --- | --- | --- |
| Model | |  | | Sum of Squares | | df | | Mean Square | | F | | p | |
| H₁ |  | Regression |  | 2044.953 |  | 2 |  | 1022.476 |  | 56.947 |  | < .001 |  |
|  |  | Residual |  | 5781.435 |  | 322 |  | 17.955 |  |  |  |  |  |
|  |  | Total |  | 7826.388 |  | 324 |  |  |  |  |  |  |  |
|  | | | | | | | | | | | | | |
| *Note.*  The intercept model is omitted, as no meaningful information can be shown. | | | | | | | | | | | | | |

| Coefficients | | | | | | | | | | | | | | | | | | | | | |
| --- | --- | --- | --- | --- | --- | --- | --- | --- | --- | --- | --- | --- | --- | --- | --- | --- | --- | --- | --- | --- | --- |
|  | | | | | | | | | | | | | | 95% CI | | | | Collinearity Statistics | | | |
| Model | |  | | Unstandardized | | Standard Error | | Standardized | | t | | p | | Lower | | Upper | | Tolerance | | VIF | |
| H₀ |  | (Intercept) |  | 0.004 |  | 0.273 |  |  |  | 0.014 |  | 0.989 |  | -0.532 |  | 0.540 |  |  |  |  |  |
| H₁ |  | (Intercept) |  | 0.002 |  | 0.235 |  |  |  | 0.008 |  | 0.993 |  | -0.460 |  | 0.464 |  |  |  |  |  |
|  |  | PSS |  | 0.302 |  | 0.042 |  | 0.370 |  | 7.252 |  | < .001 |  | 0.220 |  | 0.383 |  | 0.882 |  | 1.134 |  |
|  |  | RSQ |  | 0.167 |  | 0.034 |  | 0.248 |  | 4.856 |  | < .001 |  | 0.099 |  | 0.234 |  | 0.882 |  | 1.134 |  |
|  | | | | | | | | | | | | | | | | | | | | | |
|  | | | | | | | | | | | | | | | | | | | | | |

| Model Summary - STS | | | | | | | | | |
| --- | --- | --- | --- | --- | --- | --- | --- | --- | --- |
| Model | | R | | R² | | Adjusted R² | | RMSE | |
| H₀ |  | 0.000 |  | 0.000 |  | 0.000 |  | 4.501 |  |
| H₁ |  | 0.455 |  | 0.207 |  | 0.202 |  | 4.021 |  |
|  | | | | | | | | | |

| ANOVA | | | | | | | | | | | | | |
| --- | --- | --- | --- | --- | --- | --- | --- | --- | --- | --- | --- | --- | --- |
| Model | |  | | Sum of Squares | | df | | Mean Square | | F | | p | |
| H₁ |  | Regression |  | 1358.678 |  | 2 |  | 679.339 |  | 42.016 |  | < .001 |  |
|  |  | Residual |  | 5206.232 |  | 322 |  | 16.168 |  |  |  |  |  |
|  |  | Total |  | 6564.911 |  | 324 |  |  |  |  |  |  |  |
|  | | | | | | | | | | | | | |
| *Note.*  The intercept model is omitted, as no meaningful information can be shown. | | | | | | | | | | | | | |

| Coefficients | | | | | | | | | | | | | | | | | | | | | |
| --- | --- | --- | --- | --- | --- | --- | --- | --- | --- | --- | --- | --- | --- | --- | --- | --- | --- | --- | --- | --- | --- |
|  | | | | | | | | | | | | | | 95% CI | | | | Collinearity Statistics | | | |
| Model | |  | | Unstandardized | | Standard Error | | Standardized | | t | | p | | Lower | | Upper | | Tolerance | | VIF | |
| H₀ |  | (Intercept) |  | 0.008 |  | 0.250 |  |  |  | 0.031 |  | 0.975 |  | -0.484 |  | 0.499 |  |  |  |  |  |
| H₁ |  | (Intercept) |  | 0.006 |  | 0.223 |  |  |  | 0.028 |  | 0.978 |  | -0.433 |  | 0.445 |  |  |  |  |  |
|  |  | PSS |  | 0.247 |  | 0.039 |  | 0.330 |  | 6.251 |  | < .001 |  | 0.169 |  | 0.324 |  | 0.882 |  | 1.134 |  |
|  |  | RSQ |  | 0.135 |  | 0.033 |  | 0.219 |  | 4.146 |  | < .001 |  | 0.071 |  | 0.199 |  | 0.882 |  | 1.134 |  |
|  | | | | | | | | | | | | | | | | | | | | | |

**The relationship between resilience and possible mediators**

We tested the relationship between potential mediators and resilience using a multiple regression analysis.

| Model Summary - RESIL | | | | | | | | | | | | | | | |
| --- | --- | --- | --- | --- | --- | --- | --- | --- | --- | --- | --- | --- | --- | --- | --- |
|  | | | | | | | | | | Durbin-Watson | | | | | |
| Model | | R | | R² | | Adjusted R² | | RMSE | | Autocorrelation | | Statistic | | p | |
| H₀ |  | 0.000 |  | 0.000 |  | 0.000 |  | 11.171 |  | 0.045 |  | 1.908 |  | 0.407 |  |
| H₁ |  | 0.810 |  | 0.656 |  | 0.647 |  | 6.638 |  | -0.077 |  | 2.151 |  | 0.174 |  |
|  | | | | | | | | | | | | | | | |

The Model summary shows that the potential mediators accounts for 63.6% of resilience variance

| ANOVA | | | | | | | | | | | | | |
| --- | --- | --- | --- | --- | --- | --- | --- | --- | --- | --- | --- | --- | --- |
| Model | |  | | Sum of Squares | | df | | Mean Square | | F | | p | |
| H₁ |  | Regression |  | 26429.220 |  | 8 |  | 3303.653 |  | 74.976 |  | < .001 |  |
|  |  | Residual |  | 13879.752 |  | 315 |  | 44.063 |  |  |  |  |  |
|  |  | Total |  | 40308.972 |  | 323 |  |  |  |  |  |  |  |
|  | | | | | | | | | | | | | |
| *Note.*  The intercept model is omitted, as no meaningful information can be shown. | | | | | | | | | | | | | |

| Coefficients | | | | | | | | | | | | | | | | | | | | | |
| --- | --- | --- | --- | --- | --- | --- | --- | --- | --- | --- | --- | --- | --- | --- | --- | --- | --- | --- | --- | --- | --- |
|  | | | | | | | | | | | | | | 95% CI | | | | Collinearity Statistics | | | |
| Model | |  | | Unstandardized | | Standard Error | | Standardized | | t | | p | | Lower | | Upper | | Tolerance | | VIF | |
| H₀ |  | (Intercept) |  | -0.061 |  | 0.621 |  |  |  | -0.098 |  | 0.922 |  | -1.282 |  | 1.160 |  |  |  |  |  |
| H₁ |  | (Intercept) |  | -0.040 |  | 0.369 |  |  |  | -0.109 |  | 0.913 |  | -0.766 |  | 0.685 |  |  |  |  |  |
|  |  | SE |  | 1.040 |  | 0.125 |  | 0.345 |  | 8.298 |  | < .001 |  | 0.793 |  | 1.286 |  | 0.631 |  | 1.585 |  |
|  |  | LOT |  | 0.284 |  | 0.097 |  | 0.126 |  | 2.932 |  | 0.004 |  | 0.094 |  | 0.475 |  | 0.595 |  | 1.680 |  |
|  |  | SC |  | 0.211 |  | 0.056 |  | 0.168 |  | 3.740 |  | < .001 |  | 0.100 |  | 0.322 |  | 0.542 |  | 1.845 |  |
|  |  | STS |  | 0.234 |  | 0.099 |  | 0.094 |  | 2.352 |  | 0.019 |  | 0.038 |  | 0.429 |  | 0.681 |  | 1.468 |  |
|  |  | BU |  | -0.444 |  | 0.127 |  | -0.196 |  | -3.504 |  | < .001 |  | -0.694 |  | -0.195 |  | 0.351 |  | 2.849 |  |
|  |  | CS |  | 0.345 |  | 0.105 |  | 0.155 |  | 3.284 |  | 0.001 |  | 0.138 |  | 0.551 |  | 0.490 |  | 2.039 |  |
|  |  | AV |  | -0.205 |  | 0.113 |  | -0.075 |  | -1.815 |  | 0.070 |  | -0.427 |  | 0.017 |  | 0.643 |  | 1.556 |  |
|  |  | AP |  | 0.279 |  | 0.072 |  | 0.146 |  | 3.851 |  | < .001 |  | 0.136 |  | 0.421 |  | 0.763 |  | 1.311 |  |
|  | | | | | | | | | | | | | | | | | | | | | |

| Descriptives | | | | | | | | | |
| --- | --- | --- | --- | --- | --- | --- | --- | --- | --- |
|  | | N | | Mean | | SD | | SE | |
| RESIL |  | 324 |  | -0.061 |  | 11.171 |  | 0.621 |  |
| SE |  | 324 |  | -0.004 |  | 3.711 |  | 0.206 |  |
| LOT |  | 324 |  | -0.002 |  | 4.936 |  | 0.274 |  |
| SC |  | 324 |  | -0.021 |  | 8.891 |  | 0.494 |  |
| STS |  | 324 |  | -1.235e -4 |  | 4.506 |  | 0.250 |  |
| BU |  | 324 |  | 0.015 |  | 4.918 |  | 0.273 |  |
| CS |  | 324 |  | -0.014 |  | 5.028 |  | 0.279 |  |
| AV |  | 324 |  | 0.004 |  | 4.084 |  | 0.227 |  |
| AP |  | 324 |  | 0.004 |  | 5.845 |  | 0.325 |  |
|  | | | | | | | | | |

## SE, LOT, SC, STS, BU, CS, AV, AP as predictors of RESIL. Bayesian inference

| Model Comparison - RESIL | | | | | | | | | | | |
| --- | --- | --- | --- | --- | --- | --- | --- | --- | --- | --- | --- |
| Models | | P(M) | | P(M\|data) | | BF _M_ | | BF _10_ | | R² | |
| Null model |  | 0.111 |  | 7.536e -66 |  | 6.029e -65 |  | 1.000 |  | 0.000 |  |
| AV + AP + SE + LOT + SC + BU + STS + CS |  | 0.111 |  | 0.695 |  | 18.225 |  | 9.221e +64 |  | 0.656 |  |
| AP + SE + LOT + SC + BU + STS + CS |  | 0.014 |  | 0.158 |  | 13.343 |  | 1.679e +65 |  | 0.652 |  |
| AV + AP + SE + LOT + SC + BU + CS |  | 0.014 |  | 0.053 |  | 3.982 |  | 5.637e +64 |  | 0.650 |  |
| AP + SE + LOT + SC + BU + CS |  | 0.004 |  | 0.050 |  | 13.334 |  | 1.874e +65 |  | 0.647 |  |
| AV + AP + SE + SC + BU + STS + CS |  | 0.014 |  | 0.012 |  | 0.868 |  | 1.283e +64 |  | 0.646 |  |
| AP + SE + SC + BU + STS + CS |  | 0.004 |  | 0.005 |  | 1.347 |  | 1.983e +64 |  | 0.642 |  |
| AP + SE + LOT + SC + BU + STS |  | 0.004 |  | 0.005 |  | 1.166 |  | 1.718e +64 |  | 0.642 |  |
| AV + AP + SE + LOT + SC + BU + STS |  | 0.014 |  | 0.004 |  | 0.301 |  | 4.484e +63 |  | 0.644 |  |
| AV + AP + SE + LOT + SC + CS |  | 0.004 |  | 0.003 |  | 0.860 |  | 1.268e +64 |  | 0.641 |  |
|  | | | | | | | | | | | |
| Note.  Table displays only a subset of models; to see all models, select "No" under "Limit No. Models Shown". | | | | | | | | | | | |

### Posterior Summary

| Posterior Summaries of Coefficients | | | | | | | | | | | | | | | |
| --- | --- | --- | --- | --- | --- | --- | --- | --- | --- | --- | --- | --- | --- | --- | --- |
|  | | | | | | | | | | | | 95% Credible Interval | | | |
| Coefficient | | Mean | | SD | | P(incl) | | P(incl\|data) | | BF _inclusion_ | | Lower | | Upper | |
| Intercept |  | -0.061 |  | 0.370 |  | 1.000 |  | 1.000 |  | 1.000 |  | -0.823 |  | 0.613 |  |
| AV |  | -0.154 |  | 0.130 |  | 0.500 |  | 0.774 |  | 3.418 |  | -0.404 |  | 0.006 |  |
| AP |  | 0.266 |  | 0.075 |  | 0.500 |  | 0.997 |  | 361.183 |  | 0.123 |  | 0.428 |  |
| SE |  | 1.031 |  | 0.127 |  | 0.500 |  | 1.000 |  | 9.503e +12 |  | 0.791 |  | 1.275 |  |
| LOT |  | 0.278 |  | 0.105 |  | 0.500 |  | 0.978 |  | 44.161 |  | 0.077 |  | 0.498 |  |
| SC |  | 0.215 |  | 0.058 |  | 0.500 |  | 0.999 |  | 781.193 |  | 0.102 |  | 0.324 |  |
| BU |  | -0.434 |  | 0.137 |  | 0.500 |  | 0.992 |  | 125.099 |  | -0.720 |  | -0.166 |  |
| STS |  | 0.201 |  | 0.118 |  | 0.500 |  | 0.886 |  | 7.781 |  | 0.000 |  | 0.400 |  |
| CS |  | 0.338 |  | 0.112 |  | 0.500 |  | 0.990 |  | 94.866 |  | 0.109 |  | 0.562 |  |
|  | | | | | | | | | | | | | | | |

#### Posterior Coefficients with 95% Credible Interval


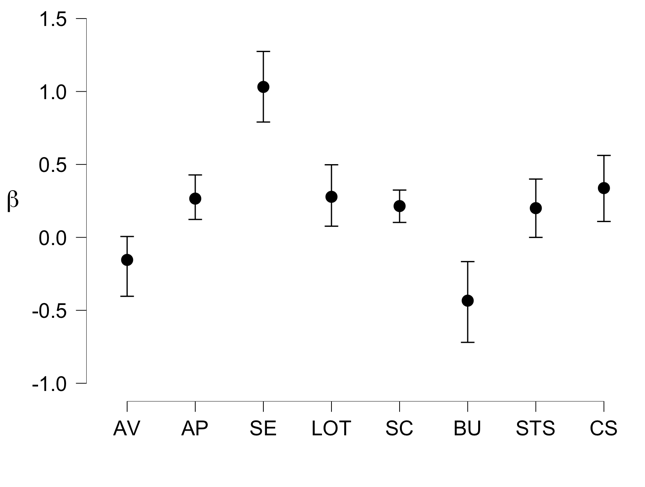


### Residuals vs Fitted


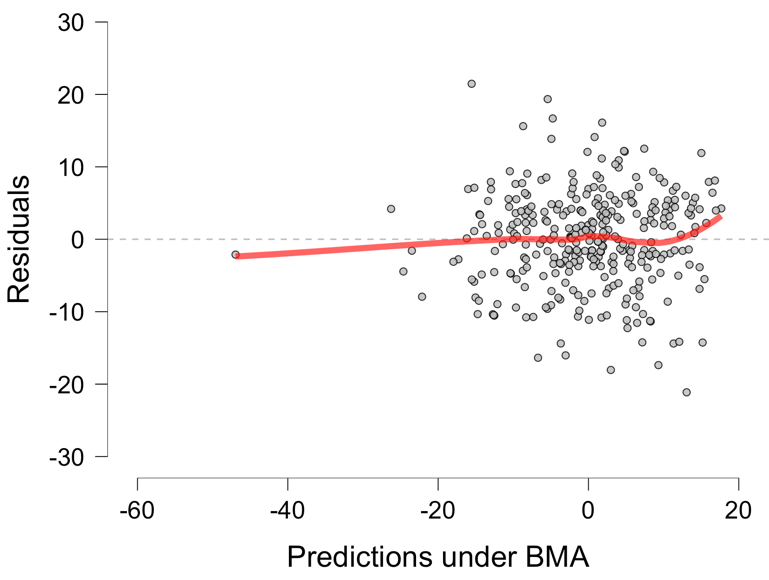


### Inclusion Probabilities


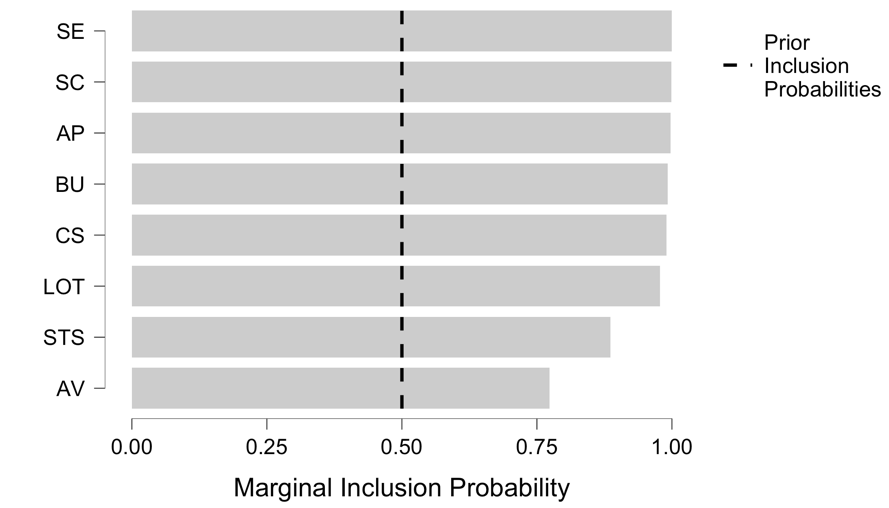


### Q-Q Plot


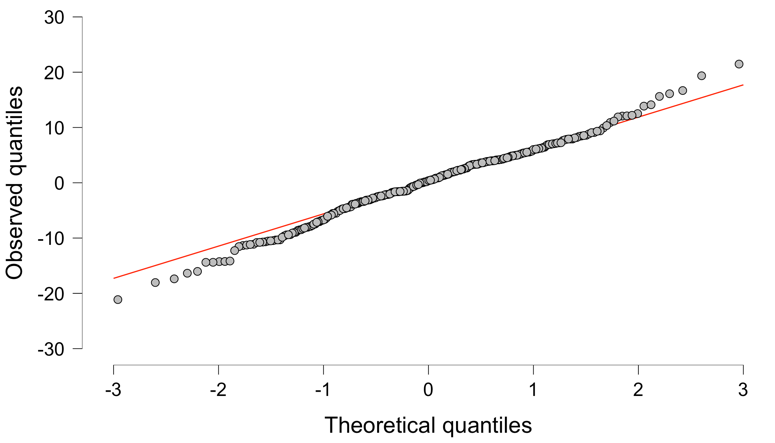


## **Mediation Analysis**

### Parameter estimates

| **Direct effects** | | | | | | | | | | | | | | | | | |
| --- | --- | --- | --- | --- | --- | --- | --- | --- | --- | --- | --- | --- | --- | --- | --- | --- | --- |
|  | | | | | | | | | | | | | | **95% Confidence Interval** | | | |
|  | |  | |  | | **Estimate** | | **Std. Error** | | **z-value** | | **p** | | **Lower** | | **Upper** | |
| PSS |  | → |  | RESIL |  | -0.016 |  | 0.081 |  | -0.202 |  | 0.840 |  | -0.183 |  | 0.142 |  |
| RSQ |  | → |  | RESIL |  | 0.040 |  | 0.058 |  | 0.689 |  | 0.491 |  | -0.070 |  | 0.150 |  |
|  | | | | | | | | | | | | | | | | | |
| Note.  Delta method standard errors, bias-corrected percentile bootstrap confidence intervals, ML estimator. | | | | | | | | | | | | | | | | | |

| **Indirect effects** | | | | | | | | | | | | | | | | | | | | | |
| --- | --- | --- | --- | --- | --- | --- | --- | --- | --- | --- | --- | --- | --- | --- | --- | --- | --- | --- | --- | --- | --- |
|  | | | | | | | | | | | | | | | | | | **95% Confidence Interval** | | | |
|  | |  | |  | |  | |  | | **Estimate** | | **Std. Error** | | **z-value** | | **p** | | **Lower** | | **Upper** | |
| PSS |  | → |  | SE |  | → |  | RESIL |  | -0.284 |  | 0.048 |  | -5.940 |  | < .001 |  | -0.410 |  | -0.187 |  |
| PSS |  | → |  | LOT |  | → |  | RESIL |  | -0.106 |  | 0.034 |  | -3.090 |  | 0.002 |  | -0.198 |  | -0.042 |  |
| PSS |  | → |  | SC |  | → |  | RESIL |  | -0.178 |  | 0.044 |  | -4.009 |  | < .001 |  | -0.285 |  | -0.094 |  |
| PSS |  | → |  | CS |  | → |  | RESIL |  | -0.099 |  | 0.034 |  | -2.927 |  | 0.003 |  | -0.184 |  | -0.044 |  |
| PSS |  | → |  | BU |  | → |  | RESIL |  | -0.156 |  | 0.044 |  | -3.542 |  | < .001 |  | -0.255 |  | -0.077 |  |
| PSS |  | → |  | STS |  | → |  | RESIL |  | 0.061 |  | 0.027 |  | 2.253 |  | 0.024 |  | 0.014 |  | 0.117 |  |
| RSQ |  | → |  | SE |  | → |  | RESIL |  | -0.077 |  | 0.030 |  | -2.543 |  | 0.011 |  | -0.140 |  | -0.025 |  |
| RSQ |  | → |  | LOT |  | → |  | RESIL |  | -0.045 |  | 0.018 |  | -2.541 |  | 0.011 |  | -0.095 |  | -0.017 |  |
| RSQ |  | → |  | SC |  | → |  | RESIL |  | -0.037 |  | 0.017 |  | -2.124 |  | 0.034 |  | -0.081 |  | -0.010 |  |
| RSQ |  | → |  | CS |  | → |  | RESIL |  | -0.013 |  | 0.014 |  | -0.904 |  | 0.366 |  | -0.042 |  | 0.009 |  |
| RSQ |  | → |  | BU |  | → |  | RESIL |  | -0.086 |  | 0.028 |  | -3.118 |  | 0.002 |  | -0.151 |  | -0.043 |  |
| RSQ |  | → |  | STS |  | → |  | RESIL |  | 0.033 |  | 0.016 |  | 2.088 |  | 0.037 |  | 0.006 |  | 0.074 |  |
|  | | | | | | | | | | | | | | | | | | | | | |
| Note.  Delta method standard errors, bias-corrected percentile bootstrap confidence intervals, ML estimator. | | | | | | | | | | | | | | | | | | | | | |

| **Total effects** | | | | | | | | | | | | | | | | | |
| --- | --- | --- | --- | --- | --- | --- | --- | --- | --- | --- | --- | --- | --- | --- | --- | --- | --- |
|  | | | | | | | | | | | | | | **95% Confidence Interval** | | | |
|  | |  | |  | | **Estimate** | | **Std. Error** | | **z-value** | | **p** | | **Lower** | | **Upper** | |
| PSS |  | → |  | RESIL |  | -0.779 |  | 0.097 |  | -8.053 |  | < .001 |  | -1.044 |  | -0.541 |  |
| RSQ |  | → |  | RESIL |  | -0.184 |  | 0.080 |  | -2.303 |  | 0.021 |  | -0.334 |  | -0.037 |  |
|  | | | | | | | | | | | | | | | | | |
| Note.  Delta method standard errors, bias-corrected percentile bootstrap confidence intervals, ML estimator. | | | | | | | | | | | | | | | | | |

| **Total indirect effects** | | | | | | | | | | | | | | | | | |
| --- | --- | --- | --- | --- | --- | --- | --- | --- | --- | --- | --- | --- | --- | --- | --- | --- | --- |
|  | | | | | | | | | | | | | | **95% Confidence Interval** | | | |
|  | |  | |  | | **Estimate** | | **Std. Error** | | **z-value** | | **p** | | **Lower** | | **Upper** | |
| PSS |  | → |  | RESIL |  | -0.763 |  | 0.085 |  | -8.949 |  | < .001 |  | -1.007 |  | -0.557 |  |
| RSQ |  | → |  | RESIL |  | -0.224 |  | 0.062 |  | -3.612 |  | < .001 |  | -0.359 |  | -0.119 |  |
|  | | | | | | | | | | | | | | | | | |
| Note.  Delta method standard errors, bias-corrected percentile bootstrap confidence intervals, ML estimator. | | | | | | | | | | | | | | | | | |

Mediation analysis final model (best fit)

| Chi Square Test Statistic (unscaled) | | | | | | | | | | | |
| --- | --- | --- | --- | --- | --- | --- | --- | --- | --- | --- | --- |
|  | | df | | AIC | | BIC | | χ² | | p | |
| Model |  | 5.000 |  | 13248.232 |  | 13388.234 |  | 8.884 |  | 0.114 |  |
|  | | | | | | | | | | | |

| **Parameter Estimates** | | | | | | | | | | | | | | | | | | | | | | | | | | | | |  |  |
| --- | --- | --- | --- | --- | --- | --- | --- | --- | --- | --- | --- | --- | --- | --- | --- | --- | --- | --- | --- | --- | --- | --- | --- | --- | --- | --- | --- | --- | --- | --- |
|  |  | | |  | | **label** | | **est** | | **se** | | **z** | | **p** | | **CI (lower)** | | **CI (upper)** | | **std (lv)** | | **std (all)** | | **std (nox)** | | **group** | | | |  |
| RESIL |  | ~ |  | | SE |  | b11 |  | 1.094 |  | 0.135 |  | 8.122 |  | < .001 |  | 0.806 |  | 1.353 |  | 1.094 |  | 0.364 |  | 0.364 |  |  |  | | |
| RESIL |  | ~ |  | | LOT |  | b12 |  | 0.335 |  | 0.112 |  | 3.001 |  | 0.003 |  | 0.123 |  | 0.554 |  | 0.335 |  | 0.146 |  | 0.146 |  |  |  | | |
| RESIL |  | ~ |  | | SC |  | b13 |  | 0.249 |  | 0.061 |  | 4.090 |  | < .001 |  | 0.123 |  | 0.362 |  | 0.249 |  | 0.197 |  | 0.197 |  |  |  | | |
| RESIL |  | ~ |  | | STS |  | b14 |  | 0.246 |  | 0.099 |  | 2.494 |  | 0.013 |  | 0.049 |  | 0.437 |  | 0.246 |  | 0.099 |  | 0.099 |  |  |  | | |
| RESIL |  | ~ |  | | BU |  | b15 |  | -0.519 |  | 0.121 |  | -4.275 |  | < .001 |  | -0.779 |  | -0.278 |  | -0.519 |  | -0.226 |  | -0.226 |  |  |  | | |
| RESIL |  | ~ |  | | CS |  | b16 |  | 0.351 |  | 0.103 |  | 3.412 |  | < .001 |  | 0.154 |  | 0.558 |  | 0.351 |  | 0.158 |  | 0.158 |  |  |  | | |
| RESIL |  | ~ |  | | PSS |  | c11 |  | -0.016 |  | 0.077 |  | -0.210 |  | 0.833 |  | -0.177 |  | 0.137 |  | -0.016 |  | -0.009 |  | -0.001 |  |  |  | | |
| RESIL |  | ~ |  | | RSQ |  | c12 |  | 0.040 |  | 0.058 |  | 0.685 |  | 0.493 |  | -0.070 |  | 0.153 |  | 0.040 |  | 0.026 |  | 0.004 |  |  |  | | |
| SE |  | ~ |  | | PSS |  | a11 |  | -0.289 |  | 0.037 |  | -7.767 |  | < .001 |  | -0.366 |  | -0.214 |  | -0.289 |  | -0.468 |  | -0.078 |  |  |  | | |
| LOT |  | ~ |  | | PSS |  | a21 |  | -0.334 |  | 0.048 |  | -6.957 |  | < .001 |  | -0.427 |  | -0.235 |  | -0.334 |  | -0.412 |  | -0.068 |  |  |  | | |
| LOT |  | ~ |  | | RSQ |  | a22 |  | -0.091 |  | 0.028 |  | -3.190 |  | 0.001 |  | -0.144 |  | -0.035 |  | -0.091 |  | -0.135 |  | -0.019 |  |  |  | | |
| SC |  | ~ |  | | PSS |  | a31 |  | -0.720 |  | 0.073 |  | -9.903 |  | < .001 |  | -0.864 |  | -0.581 |  | -0.720 |  | -0.489 |  | -0.081 |  |  |  | | |
| STS |  | ~ |  | | PSS |  | a41 |  | 0.251 |  | 0.043 |  | 5.890 |  | < .001 |  | 0.171 |  | 0.342 |  | 0.251 |  | 0.337 |  | 0.056 |  |  |  | | |
| STS |  | ~ |  | | RSQ |  | a42 |  | 0.125 |  | 0.035 |  | 3.618 |  | < .001 |  | 0.058 |  | 0.197 |  | 0.125 |  | 0.204 |  | 0.028 |  |  |  | | |
| BU |  | ~ |  | | PSS |  | a51 |  | 0.317 |  | 0.046 |  | 6.887 |  | < .001 |  | 0.227 |  | 0.411 |  | 0.317 |  | 0.393 |  | 0.065 |  |  |  | | |
| BU |  | ~ |  | | RSQ |  | a52 |  | 0.130 |  | 0.028 |  | 4.630 |  | < .001 |  | 0.073 |  | 0.184 |  | 0.130 |  | 0.196 |  | 0.027 |  |  |  | | |
| CS |  | ~ |  | | PSS |  | a61 |  | -0.296 |  | 0.051 |  | -5.769 |  | < .001 |  | -0.397 |  | -0.200 |  | -0.296 |  | -0.355 |  | -0.059 |  |  |  | | |
| SE |  | ~~ |  | | LOT |  |  |  | 4.353 |  | 0.851 |  | 5.116 |  | < .001 |  | 2.647 |  | 6.084 |  | 4.353 |  | 0.308 |  | 0.308 |  |  |  | | |
| SE |  | ~~ |  | | SC |  |  |  | 6.956 |  | 1.498 |  | 4.643 |  | < .001 |  | 3.935 |  | 9.737 |  | 6.956 |  | 0.282 |  | 0.282 |  |  |  | | |
| LOT |  | ~~ |  | | SC |  |  |  | 12.725 |  | 2.343 |  | 5.432 |  | < .001 |  | 8.263 |  | 17.643 |  | 12.725 |  | 0.395 |  | 0.395 |  |  |  | | |
| SE |  | ~~ |  | | BU |  |  |  | -3.896 |  | 0.864 |  | -4.510 |  | < .001 |  | -5.607 |  | -2.272 |  | -3.896 |  | -0.281 |  | -0.281 |  |  |  | | |
| LOT |  | ~~ |  | | BU |  |  |  | -5.892 |  | 1.226 |  | -4.805 |  | < .001 |  | -8.427 |  | -3.524 |  | -5.892 |  | -0.325 |  | -0.325 |  |  |  | | |
| SC |  | ~~ |  | | BU |  |  |  | -10.710 |  | 2.147 |  | -4.987 |  | < .001 |  | -15.219 |  | -6.617 |  | -10.710 |  | -0.339 |  | -0.339 |  |  |  | | |
| STS |  | ~~ |  | | BU |  |  |  | 5.148 |  | 1.044 |  | 4.932 |  | < .001 |  | 3.161 |  | 7.260 |  | 5.148 |  | 0.305 |  | 0.305 |  |  |  | | |
| SE |  | ~~ |  | | CS |  |  |  | 4.514 |  | 1.047 |  | 4.310 |  | < .001 |  | 2.400 |  | 6.527 |  | 4.514 |  | 0.292 |  | 0.292 |  |  |  | | |
| LOT |  | ~~ |  | | CS |  |  |  | 4.127 |  | 1.269 |  | 3.252 |  | 0.001 |  | 1.710 |  | 6.603 |  | 4.127 |  | 0.204 |  | 0.204 |  |  |  | | |
| SC |  | ~~ |  | | CS |  |  |  | 8.687 |  | 2.257 |  | 3.848 |  | < .001 |  | 4.340 |  | 13.224 |  | 8.687 |  | 0.247 |  | 0.247 |  |  |  | | |
| BU |  | ~~ |  | | CS |  |  |  | -12.177 |  | 1.595 |  | -7.636 |  | < .001 |  | -15.402 |  | -9.085 |  | -12.177 |  | -0.614 |  | -0.614 |  |  |  | | |
| SE |  | ~~ |  | | STS |  |  |  | -1.759 |  | 0.665 |  | -2.645 |  | 0.008 |  | -3.101 |  | -0.435 |  | -1.759 |  | -0.134 |  | -0.134 |  |  |  | | |
| SC |  | ~ |  | | STS |  |  |  | -0.189 |  | 0.087 |  | -2.179 |  | 0.029 |  | -0.358 |  | -0.009 |  | -0.189 |  | -0.095 |  | -0.095 |  |  |  | | |
| RESIL |  | ~~ |  | | RESIL |  |  |  | 44.757 |  | 3.616 |  | 12.378 |  | < .001 |  | 36.568 |  | 50.771 |  | 44.757 |  | 0.358 |  | 0.358 |  |  |  | | |
| SE |  | ~~ |  | | SE |  |  |  | 10.820 |  | 0.799 |  | 13.546 |  | < .001 |  | 9.253 |  | 12.352 |  | 10.820 |  | 0.781 |  | 0.781 |  |  |  | | |
| LOT |  | ~~ |  | | LOT |  |  |  | 18.466 |  | 1.521 |  | 12.140 |  | < .001 |  | 15.449 |  | 21.345 |  | 18.466 |  | 0.773 |  | 0.773 |  |  |  | | |
| SC |  | ~~ |  | | SC |  |  |  | 56.113 |  | 4.607 |  | 12.179 |  | < .001 |  | 47.463 |  | 64.934 |  | 56.113 |  | 0.714 |  | 0.714 |  |  |  | | |
| STS |  | ~~ |  | | STS |  |  |  | 16.024 |  | 1.461 |  | 10.964 |  | < .001 |  | 13.317 |  | 18.960 |  | 16.024 |  | 0.798 |  | 0.798 |  |  |  | | |
| BU |  | ~~ |  | | BU |  |  |  | 17.805 |  | 1.502 |  | 11.857 |  | < .001 |  | 14.543 |  | 20.687 |  | 17.805 |  | 0.755 |  | 0.755 |  |  |  | | |
| CS |  | ~~ |  | | CS |  |  |  | 22.079 |  | 2.023 |  | 10.914 |  | < .001 |  | 18.243 |  | 26.324 |  | 22.079 |  | 0.874 |  | 0.874 |  |  |  | | |
| PSS |  | ~~ |  | | PSS |  |  |  | 36.253 |  | 0.000 |  |  |  |  |  | 36.253 |  | 36.253 |  | 36.253 |  | 1.000 |  | 36.253 |  |  |  | | |
| PSS |  | ~~ |  | | RSQ |  |  |  | 15.112 |  | 0.000 |  |  |  |  |  | 15.112 |  | 15.112 |  | 15.112 |  | 0.344 |  | 15.112 |  |  |  | | |
| RSQ |  | ~~ |  | | RSQ |  |  |  | 53.229 |  | 0.000 |  |  |  |  |  | 53.229 |  | 53.229 |  | 53.229 |  | 1.000 |  | 53.229 |  |  |  | | |
| ind_x1_m1_y1 |  | := |  | | a11*b11 |  | ind_x1_m1_y1 |  | -0.316 |  | 0.055 |  | -5.733 |  | < .001 |  | -0.437 |  | -0.209 |  | -0.316 |  | -0.170 |  | -0.028 |  |  |  | | |
| ind_x1_m2_y1 |  | := |  | | a21*b12 |  | ind_x1_m2_y1 |  | -0.112 |  | 0.041 |  | -2.751 |  | 0.006 |  | -0.200 |  | -0.039 |  | -0.112 |  | -0.060 |  | -0.010 |  |  |  | | |
| ind_x1_m3_y1 |  | := |  | | a31*b13 |  | ind_x1_m3_y1 |  | -0.179 |  | 0.048 |  | -3.719 |  | < .001 |  | -0.279 |  | -0.086 |  | -0.179 |  | -0.097 |  | -0.016 |  |  |  | | |
| ind_x1_m4_y1 |  | := |  | | a41*b14 |  | ind_x1_m4_y1 |  | 0.062 |  | 0.026 |  | 2.348 |  | 0.019 |  | 0.013 |  | 0.118 |  | 0.062 |  | 0.033 |  | 0.006 |  |  |  | | |
| ind_x1_m5_y1 |  | := |  | | a51*b15 |  | ind_x1_m5_y1 |  | -0.164 |  | 0.046 |  | -3.579 |  | < .001 |  | -0.269 |  | -0.087 |  | -0.164 |  | -0.089 |  | -0.015 |  |  |  | | |
| ind_x1_m6_y1 |  | := |  | | a61*b16 |  | ind_x1_m6_y1 |  | -0.104 |  | 0.035 |  | -2.932 |  | 0.003 |  | -0.182 |  | -0.041 |  | -0.104 |  | -0.056 |  | -0.009 |  |  |  | | |
| ind_x1_y1 |  | := |  | | ind_x1_m1_y1+ind_x1_m2_y1+ind_x1_m3_y1+ind_x1_m4_y1+ind_x1_m5_y1+ind_x1_m6_y1 |  | ind_x1_y1 |  | -0.814 |  | 0.100 |  | -8.122 |  | < .001 |  | -1.021 |  | -0.622 |  | -0.814 |  | -0.438 |  | -0.073 |  |  |  | | |
| tot_x1_y1 |  | := |  | | ind_x1_y1+c11 |  | tot_x1_y1 |  | -0.830 |  | 0.111 |  | -7.497 |  | < .001 |  | -1.052 |  | -0.615 |  | -0.830 |  | -0.447 |  | -0.074 |  |  |  | | |
| ind_x2_m2_y1 |  | := |  | | a22*b12 |  | ind_x2_m2_y1 |  | -0.030 |  | 0.014 |  | -2.232 |  | 0.026 |  | -0.057 |  | -0.007 |  | -0.030 |  | -0.020 |  | -0.003 |  |  |  | | |
| ind_x2_m4_y1 |  | := |  | | a42*b14 |  | ind_x2_m4_y1 |  | 0.031 |  | 0.016 |  | 1.873 |  | 0.061 |  | 0.005 |  | 0.067 |  | 0.031 |  | 0.020 |  | 0.003 |  |  |  | | |
| ind_x2_m5_y1 |  | := |  | | a52*b15 |  | ind_x2_m5_y1 |  | -0.068 |  | 0.022 |  | -3.038 |  | 0.002 |  | -0.117 |  | -0.030 |  | -0.068 |  | -0.044 |  | -0.006 |  |  |  | | |
| ind_x2_y1 |  | := |  | | ind_x2_m2_y1+ind_x2_m4_y1+ind_x2_m5_y1 |  | ind_x2_y1 |  | -0.067 |  | 0.025 |  | -2.665 |  | 0.008 |  | -0.117 |  | -0.022 |  | -0.067 |  | -0.044 |  | -0.006 |  |  |  | | |
| tot_x2_y1 |  | := |  | | ind_x2_y1+c12 |  | tot_x2_y1 |  | -0.027 |  | 0.057 |  | -0.480 |  | 0.631 |  | -0.138 |  | 0.085 |  | -0.027 |  | -0.018 |  | -0.002 |  |  |  | | |
|  | | | | | | | | | | | | | | | | | | | | | | | | | | | | |  |  |

| User model versus baseline model | | | |
| --- | --- | --- | --- |
|  | | Model | |
| Comparative Fit Index (CFI) |  | 0.997 |  |
| Tucker-Lewis Index (TLI) |  | 0.977 |  |
| Bentler-Bonett Non-normed Fit Index (NNFI) |  | 0.977 |  |
| Bentler-Bonett Normed Fit Index (NFI) |  | 0.993 |  |
| Parsimony Normed Fit Index (PNFI) |  | 0.142 |  |
| Bollen's Relative Fit Index (RFI) |  | 0.949 |  |
| Bollen's Incremental Fit Index (IFI) |  | 0.997 |  |
| Relative Noncentrality Index (RNI) |  | 0.997 |  |
|  | | | |
|  | | | |

| Root Mean Square Error of Approximation | | | |
| --- | --- | --- | --- |
|  | | Model | |
| RMSEA |  | 0.049 |  |
| Upper 90% CI |  | 0.100 |  |
| Lower 90% CI |  | 0.000 |  |
| p-value RMSEA <= 0.05 |  | 0.445 |  |
|  | | | |

| R-Squared | | | |
| --- | --- | --- | --- |
| Variable | | R² | |
| RESIL |  | 0.642 |  |
| SE |  | 0.219 |  |
| LOT |  | 0.227 |  |
| SC |  | 0.286 |  |
| STS |  | 0.202 |  |
| BU |  | 0.245 |  |
| CS |  | 0.126 |  |
|  | | | |

Individual R^2^: indicate that the covariates involved this proportion of variation in the mediator

| Covariances (lower triangle) / correlations (upper triangle) | | | | | | | | | | | | | | | | | | | | | |
| --- | --- | --- | --- | --- | --- | --- | --- | --- | --- | --- | --- | --- | --- | --- | --- | --- | --- | --- | --- | --- | --- |
|  | |  | | RESIL | | SE | | LOT | | SC | | STS | | BU | | CS | | PSS | | RSQ | |
| RESIL |  | observed |  | 125.379 |  | 0.657 |  | 0.563 |  | 0.596 |  | -0.239 |  | -0.621 |  | 0.565 |  | -0.460 |  | -0.264 |  |
|  |  | fitted |  | 124.888 |  | 0.655 |  | 0.555 |  | 0.594 |  | -0.220 |  | -0.610 |  | 0.565 |  | -0.461 |  | -0.178 |  |
|  |  | residual |  | 0.491 |  | 0.002 |  | 0.008 |  | 0.002 |  | -0.019 |  | -0.011 |  | 8.770e -5 |  | 9.033e -4 |  | -0.086 |  |
| SE |  | observed |  | 27.373 |  | 13.854 |  | 0.468 |  | 0.469 |  | -0.321 |  | -0.451 |  | 0.408 |  | -0.467 |  | -0.282 |  |
|  |  | fitted |  | 27.248 |  | 13.847 |  | 0.454 |  | 0.468 |  | -0.296 |  | -0.431 |  | 0.407 |  | -0.468 |  | -0.161 |  |
|  |  | residual |  | 0.125 |  | 0.007 |  | 0.014 |  | 0.001 |  | -0.026 |  | -0.020 |  | 6.649e -4 |  | 1.171e -4 |  | -0.121 |  |
| LOT |  | observed |  | 31.026 |  | 8.570 |  | 24.238 |  | 0.547 |  | -0.230 |  | -0.494 |  | 0.334 |  | -0.455 |  | -0.331 |  |
|  |  | fitted |  | 30.302 |  | 8.252 |  | 23.874 |  | 0.538 |  | -0.211 |  | -0.483 |  | 0.331 |  | -0.459 |  | -0.277 |  |
|  |  | residual |  | 0.723 |  | 0.318 |  | 0.364 |  | 0.009 |  | -0.019 |  | -0.011 |  | 0.004 |  | 0.003 |  | -0.054 |  |
| SC |  | observed |  | 59.218 |  | 15.491 |  | 23.924 |  | 78.811 |  | -0.305 |  | -0.528 |  | 0.383 |  | -0.527 |  | -0.288 |  |
|  |  | fitted |  | 58.859 |  | 15.435 |  | 23.319 |  | 78.633 |  | -0.294 |  | -0.517 |  | 0.382 |  | -0.528 |  | -0.199 |  |
|  |  | residual |  | 0.359 |  | 0.056 |  | 0.605 |  | 0.178 |  | -0.011 |  | -0.011 |  | 0.001 |  | 5.978e -4 |  | -0.089 |  |
| STS |  | observed |  | -12.016 |  | -5.375 |  | -5.092 |  | -12.185 |  | 20.200 |  | 0.468 |  | -0.159 |  | 0.406 |  | 0.333 |  |
|  |  | fitted |  | -11.001 |  | -4.932 |  | -4.620 |  | -11.702 |  | 20.085 |  | 0.459 |  | -0.144 |  | 0.407 |  | 0.320 |  |
|  |  | residual |  | -1.015 |  | -0.443 |  | -0.472 |  | -0.483 |  | 0.115 |  | 0.010 |  | -0.015 |  | -0.001 |  | 0.013 |  |
| BU |  | observed |  | -34.121 |  | -8.229 |  | -11.926 |  | -23.011 |  | 10.331 |  | 24.081 |  | -0.666 |  | 0.455 |  | 0.375 |  |
|  |  | fitted |  | -33.100 |  | -7.782 |  | -11.452 |  | -22.285 |  | 9.986 |  | 23.591 |  | -0.662 |  | 0.460 |  | 0.331 |  |
|  |  | residual |  | -1.021 |  | -0.447 |  | -0.474 |  | -0.726 |  | 0.345 |  | 0.490 |  | -0.004 |  | -0.005 |  | 0.044 |  |
| CS |  | observed |  | 31.798 |  | 7.629 |  | 8.272 |  | 17.094 |  | -3.590 |  | -16.417 |  | 25.254 |  | -0.355 |  | -0.168 |  |
|  |  | fitted |  | 31.731 |  | 7.614 |  | 8.120 |  | 17.030 |  | -3.249 |  | -16.157 |  | 25.254 |  | -0.355 |  | -0.122 |  |
|  |  | residual |  | 0.067 |  | 0.014 |  | 0.152 |  | 0.064 |  | -0.340 |  | -0.260 |  | 2.960e -5 |  | 2.155e -7 |  | -0.046 |  |
| PSS |  | observed |  | -31.025 |  | -10.476 |  | -13.494 |  | -28.192 |  | 10.980 |  | 13.449 |  | -10.729 |  | 36.253 |  | 0.344 |  |
|  |  | fitted |  | -31.025 |  | -10.476 |  | -13.494 |  | -28.192 |  | 10.980 |  | 13.449 |  | -10.729 |  | 36.253 |  | 0.344 |  |
|  |  | residual |  | 6.155e -7 |  | 1.550e -7 |  | 1.796e -7 |  | -1.649e -7 |  | 7.254e -9 |  | -1.691e -7 |  | 2.327e -7 |  | 0.000 |  | 0.000 |  |
| RSQ |  | observed |  | -21.562 |  | -7.657 |  | -11.885 |  | -18.641 |  | 10.911 |  | 13.426 |  | -6.147 |  | 15.112 |  | 53.229 |  |
|  |  | fitted |  | -14.494 |  | -4.367 |  | -9.879 |  | -12.861 |  | 10.452 |  | 11.721 |  | -4.472 |  | 15.112 |  | 53.229 |  |
|  |  | residual |  | -7.068 |  | -3.290 |  | -2.005 |  | -5.780 |  | 0.460 |  | 1.705 |  | -1.675 |  | 0.000 |  | 0.000 |  |
|  | | | | | | | | | | | | | | | | | | | | | |

The goal of structural equation modelling here is to test whether our theoretically motivated model of the covariance among variables provides a good approximation of the data. More specifically, we are trying to test how well a parsimonious model (composed of measurement and/or structural components) reproduces the observed covariance matrix. Formally, we are seeking to develop a model whose *model-implied covariance matrix* approaches the *sample (observed) covariance matrix*.

The covariance matrix above provide evidence of a good fit.

We also computed residual covariance matrix (using R syntaxis) resid(NewModel9, "cor"):

$type

[1] "cor.bollen"

$cov

   RESIL SE   LOT  SC   STS  BU   CS   PSS  RSQ

RESIL 0.000

SE   0.001 0.000

LOT  0.008 0.013 0.000

SC   0.002 0.001 0.009 0.000

STS  -0.019 -0.026 -0.018 -0.011 0.000

BU  -0.011 -0.020 -0.010 -0.011 0.011 0.000

CS   0.000 0.001 0.003 0.001 -0.017 -0.004 0.000

PSS  0.001 0.000 0.003 0.001 -0.001 -0.005 0.000 0.000

RSQ  -0.083 -0.117 -0.053 -0.088 0.013 0.042 -0.043 0.000 0.000

The residual covariance matrix indicates that we do not have significantly unpredicted (positive value >.1) or significantly overpredicted (negative value >.1).

| Modification Indices | | | | | | | | | | | | | | | |
| --- | --- | --- | --- | --- | --- | --- | --- | --- | --- | --- | --- | --- | --- | --- | --- |
|  | |  | |  | | mi | | epc | | sepc (lv) | | sepc (all) | | sepc (nox) | |
| SE |  | ~ |  | SC |  | 4.475 |  | 2.224 |  | 2.224 |  | 5.299 |  | 5.299 |  |
| SE |  | ~ |  | RESIL |  | 4.459 |  | 1.576 |  | 1.576 |  | 4.732 |  | 4.732 |  |
| SE |  | ~ |  | LOT |  | 4.448 |  | 0.578 |  | 0.578 |  | 0.759 |  | 0.759 |  |
| SE |  | ~ |  | BU |  | 4.447 |  | -0.402 |  | -0.402 |  | -0.525 |  | -0.525 |  |
| SE |  | ~ |  | RSQ |  | 4.446 |  | -0.052 |  | -0.052 |  | -0.103 |  | -0.014 |  |
| SE |  | ~ |  | STS |  | 4.446 |  | -0.418 |  | -0.418 |  | -0.504 |  | -0.504 |  |
| RSQ |  | ~ |  | SE |  | 4.049 |  | -0.178 |  | -0.178 |  | -0.091 |  | -0.091 |  |
| PSS |  | ~ |  | SE |  | 3.243 |  | 0.341 |  | 0.341 |  | 0.211 |  | 0.211 |  |
| RSQ |  | ~ |  | RESIL |  | 3.089 |  | -0.063 |  | -0.063 |  | -0.097 |  | -0.097 |  |
| PSS |  | ~ |  | RESIL |  | 2.463 |  | 0.121 |  | 0.121 |  | 0.224 |  | 0.224 |  |
| RSQ |  | ~ |  | LOT |  | 2.235 |  | -0.161 |  | -0.161 |  | -0.108 |  | -0.108 |  |
| RSQ |  | ~ |  | SC |  | 2.173 |  | -0.054 |  | -0.054 |  | -0.066 |  | -0.066 |  |
| PSS |  | ~ |  | LOT |  | 1.952 |  | 0.337 |  | 0.337 |  | 0.274 |  | 0.274 |  |
| PSS |  | ~ |  | SC |  | 1.873 |  | 0.112 |  | 0.112 |  | 0.165 |  | 0.165 |  |
| SC |  | ~ |  | RESIL |  | 1.768 |  | 3.488 |  | 3.488 |  | 4.396 |  | 4.396 |  |
| SC |  | ~~ |  | STS |  | 1.760 |  | 9.924 |  | 9.924 |  | 0.331 |  | 0.331 |  |
| SC |  | ~ |  | RSQ |  | 1.760 |  | -0.078 |  | -0.078 |  | -0.064 |  | -0.009 |  |
| SC |  | ~ |  | LOT |  | 1.760 |  | 0.855 |  | 0.855 |  | 0.471 |  | 0.471 |  |
| SC |  | ~ |  | BU |  | 1.760 |  | -0.861 |  | -0.861 |  | -0.471 |  | -0.471 |  |
| SC |  | ~ |  | SE |  | 1.748 |  | -5.622 |  | -5.622 |  | -2.359 |  | -2.359 |  |
| RSQ |  | ~ |  | BU |  | 1.202 |  | 0.102 |  | 0.102 |  | 0.068 |  | 0.068 |  |
| PSS |  | ~ |  | BU |  | 1.167 |  | -0.237 |  | -0.237 |  | -0.191 |  | -0.191 |  |
| RSQ |  | ~ |  | CS |  | 0.645 |  | -0.056 |  | -0.056 |  | -0.038 |  | -0.038 |  |
| PSS |  | ~ |  | CS |  | 0.556 |  | 0.115 |  | 0.115 |  | 0.096 |  | 0.096 |  |
| RSQ |  | ~ |  | STS |  | 0.512 |  | 0.161 |  | 0.161 |  | 0.099 |  | 0.099 |  |
| PSS |  | ~ |  | STS |  | 0.181 |  | -0.137 |  | -0.137 |  | -0.102 |  | -0.102 |  |
| STS |  | ~ |  | SC |  | 0.105 |  | 0.021 |  | 0.021 |  | 0.041 |  | 0.041 |  |
| LOT |  | ~~ |  | STS |  | 0.073 |  | -0.251 |  | -0.251 |  | -0.015 |  | -0.015 |  |
| LOT |  | ~ |  | STS |  | 0.073 |  | -0.016 |  | -0.016 |  | -0.014 |  | -0.014 |  |
| LOT |  | ~ |  | BU |  | 0.072 |  | -0.049 |  | -0.049 |  | -0.048 |  | -0.048 |  |
| LOT |  | ~ |  | SE |  | 0.072 |  | 0.142 |  | 0.142 |  | 0.109 |  | 0.109 |  |
| LOT |  | ~ |  | SC |  | 0.072 |  | 0.083 |  | 0.083 |  | 0.150 |  | 0.150 |  |
| LOT |  | ~ |  | RESIL |  | 0.072 |  | 0.177 |  | 0.177 |  | 0.404 |  | 0.404 |  |
| STS |  | ~ |  | LOT |  | 0.036 |  | -0.010 |  | -0.010 |  | -0.011 |  | -0.011 |  |
| CS |  | ~ |  | SE |  | 0.019 |  | 0.080 |  | 0.080 |  | 0.059 |  | 0.059 |  |
| STS |  | ~~ |  | CS |  | 0.019 |  | -0.141 |  | -0.141 |  | -0.008 |  | -0.008 |  |
| STS |  | ~ |  | CS |  | 0.016 |  | -0.006 |  | -0.006 |  | -0.007 |  | -0.007 |  |
| CS |  | ~ |  | SC |  | 0.015 |  | 0.040 |  | 0.040 |  | 0.071 |  | 0.071 |  |
| CS |  | ~ |  | STS |  | 0.015 |  | -0.008 |  | -0.008 |  | -0.007 |  | -0.007 |  |
| STS |  | ~ |  | BU |  | 0.014 |  | 0.010 |  | 0.010 |  | 0.010 |  | 0.010 |  |
| STS |  | ~ |  | SE |  | 0.012 |  | -0.019 |  | -0.019 |  | -0.016 |  | -0.016 |  |
| STS |  | ~ |  | RESIL |  | 0.008 |  | -0.003 |  | -0.003 |  | -0.008 |  | -0.008 |  |
| CS |  | ~ |  | RESIL |  | 0.007 |  | 0.050 |  | 0.050 |  | 0.111 |  | 0.111 |  |
| CS |  | ~ |  | BU |  | 0.006 |  | -0.013 |  | -0.013 |  | -0.012 |  | -0.012 |  |
| CS |  | ~ |  | LOT |  | 0.003 |  | -0.023 |  | -0.023 |  | -0.022 |  | -0.022 |  |
| CS |  | ~ |  | RSQ |  | 0.003 |  | 0.002 |  | 0.002 |  | 0.003 |  | 4.167e  -4 |  |
| RSQ |  | ~~ |  | RSQ |  | 3.713e -29 |  | 3.713e -29 |  | 0.000 |  | 0.000 |  | 0.000 |  |
| PSS |  | ~~ |  | RSQ |  | 4.499e -31 |  | 4.499e -31 |  | 4.499e -31 |  |  |  | 4.499e -31 |  |
| PSS |  | ~~ |  | PSS |  | 4.417e -31 |  | 4.417e -31 |  | 0.000 |  | 0.000 |  | 0.000 |  |
| PSS |  | ~ |  | RSQ |  | 9.459e -32 |  | -2.097e -17 |  | -2.097e -17 |  | -2.541e -17 |  | -3.483e -18 |  |
| RSQ |  | ~ |  | PSS |  | 1.300e -32 |  | 9.790e -18 |  | 9.790e -18 |  | 8.079e -18 |  | 1.342e -18 |  |
|  | | | | | | | | | | | | | | | |

The modification indices table above does not provide evidence that some of the paths should be freeing.

It has to be noted that instead of using the default 'delta method' for testing mediation (which is known to be problematic because the sampling distribution of the indirect path product term is not normal), we used bootstrapping. Bootstrapping is a common workaround for the debate in the literature about estimates for indirect paths as it does not make strong assumptions about the distribution of the coefficient of interest (i.e., the sampling distributions of the mediated paths). As all indirect paths are significant, we can conclude that these is evidence for mediation.
