# Supplementary material for: Individual factors in the relationship between stress and resilience in mental health psychology practitioners during the COVID-19 pandemic
Source: J Health Psychol. 2021 Dec 7;27(11):2613–31. doi: 10.1177/13591053211059393 (PMC9483698; doi:10.1177/13591053211059393)
Supplement: sj-docx-2-hpq-10.1177_13591053211059393 – Supplemental material for Individual factors in the relationship between stress and resilience in mental health psychology practitioners during the COVID-19 pandemic [file sj-docx-2-hpq-10.1177_13591053211059393.docx]

**Determining sample size**

The sample size was calculated using G*Power version 3.1.9.6.

The full regression model for the present study is displayed below:

***RESIL = b_0_*** ***+ b_1_(AV) + b_2_(AP) + b_3_(LOT) + b_4_(SC) + b_5_(CS) + b_6_(BU) + b_7_(STS) + b_8_(SE)***

We believe, from previous research, that the R^2^ for the full-model with eight continuous predictor variables will be will be about 0.5. It is plausible to assume that each of these predictors will add about 0.1 (variance explained by special effect) to the R^2^ when it is added last to the model. The residual variance is defined as 1 – (R^2^ of the full-model), and in this case is 1 – 0.5 = 0.5.  The total number of variables (predictors) is 8 and the number being tested (df) is one.

We ran calculations with power equal to 0.7, 0.8 and 0.9 with steps of 0.01 Figure S1).

Figure S1. Required sample size depending on Power calculation

This gives us a range of sample sizes ranging from 132 (Power = 0.7) to 236 (Power = 0.95).
