# Supplementary material for: Individual factors in the relationship between stress and resilience in mental health psychology practitioners during the COVID-19 pandemic
Source: J Health Psychol. 2021 Dec 7;27(11):2613–31. doi: 10.1177/13591053211059393 (PMC9483698; doi:10.1177/13591053211059393)
Supplement: sj-html-4-hpq-10.1177_13591053211059393 – Supplemental material for Individual factors in the relationship between stress and resilience in mental health psychology practitioners during the COVID-19 pandemic [file sj-html-4-hpq-10.1177_13591053211059393.html]

 
 
	 
		 
		 JASP 		 	 
	 
  
 
  Results    Prior testing our mediation models, we examined the relationship between perceived stress and resilience. Specifically, we tested whether perceived stress can predict resilience in psychologists-practitioners.   In the present study, we measured perceived stress using two measurements. One of them is PSS (Perceived stress scale) is a measure of the degree to which situations in one’s life are appraised as stressful during the last month.  the questions are of a general nature and hence are relatively free of content specific to any subpopulation group. The second measure is RSQ. This questionnaire was designed to assess individuals’ involuntary stress reactions to the COVID-19 pandemic.  These two measurements of stress are our predictors and correlate with each other. As both measurements were taken during the lockdown period, it is difficult to clearly separate their the effects on resilience. Therefore, we, first, tested  three simple linear models:Regression model 1 (RSQ as predictor), Regression model 2 (PSS as predictor) and Regression model 3 (both RSQ and PSS as predictors).   
 
  Bayesian Correlation betweel all variables    Prior testing our mediation model, we assessed associations between variables in the present study.    
 
 
	 
		 
			 
				 
					 
						 
							 
							Bayesian Pearson Correlations
							 

							  

							  
						 
					 
				 
			 

			 
				 
				Variable
				 

				 
				 
				 

				 
				RESIL
				 

				 
				PSS
				 

				 
				RSQ
				 

				 
				SE
				 

				 
				LOT
				 

				 
				AV
				 

				 
				AP
				 

				 
				SC
				 

				 
				CS
				 

				 
				BU
				 

				 
				STS
				 
			 
		 

		 
			 
				 
				1. RESIL
				 

				  

				 
				Pearson's r
				 

				  

				 
				—
				 

				  

				  

				  

				  

				  

				  

				  

				  

				  

				  

				  

				  

				  

				  

				  

				  

				  

				  

				  

				  

				  

			 

			 
				  

				  

				 
				BF₁₀
				 

				  

				 
				—
				 

				  

				  

				  

				  

				  

				  

				  

				  

				  

				  

				  

				  

				  

				  

				  

				  

				  

				  

				  

				  

				  
			 

			 
				 
				 
				 

				  

				 
				Upper 95% CI
				 

				  

				 
				—
				 

				  

				  

				  

				  

				  

				  

				  

				  

				  

				  

				  

				  

				  

				  

				  

				  

				  

				  

				  

				  

				  
			 

			 
				 
				 
				 

				  

				 
				Lower 95% CI
				 

				  

				 
				—
				 

				  

				  

				  

				  

				  

				  

				  

				  

				  

				  

				  

				  

				  

				  

				  

				  

				  

				  

				  

				  

				  
			 

			 
				 
				2. PSS
				 

				  

				 
				Pearson's r
				 

				  

				 
				-0.460
				 

				 
				***
				 

				 
				—
				 

				  

				  

				  

				  

				  

				  

				  

				  

				  

				  

				  

				  

				  

				  

				  

				  

				  

				  

				  
			 

			 
				  

				  

				 
				BF₁₀
				 

				  

				 
				2.688e +15
				 

				  

				 
				—
				 

				  

				  

				  

				  

				  

				  

				  

				  

				  

				  

				  

				  

				  

				  

				  

				  

				  

				  

				  
			 

			 
				 
				 
				 

				  

				 
				Upper 95% CI
				 

				  

				 
				-0.368
				 

				  

				 
				—
				 

				  

				  

				  

				  

				  

				  

				  

				  

				  

				  

				  

				  

				  

				  

				  

				  

				  

				  

				  
			 

			 
				 
				 
				 

				  

				 
				Lower 95% CI
				 

				  

				 
				-0.540
				 

				  

				 
				—
				 

				  

				  

				  

				  

				  

				  

				  

				  

				  

				  

				  

				  

				  

				  

				  

				  

				  

				  

				  
			 

			 
				 
				3. RSQ
				 

				  

				 
				Pearson's r
				 

				  

				 
				-0.264
				 

				 
				***
				 

				 
				0.344
				 

				 
				***
				 

				 
				—
				 

				  

				  

				  

				  

				  

				  

				  

				  

				  

				  

				  

				  

				  

				  

				  

				  

				  
			 

			 
				  

				  

				 
				BF₁₀
				 

				  

				 
				7510.235
				 

				  

				 
				4.188e 


				+7
				 

				  

				 
				—
				 

				  

				  

				  

				  

				  

				  

				  

				  

				  

				  

				  

				  

				  

				  

				  

				  

				  
			 

			 
				 
				 
				 

				  

				 
				Upper 95% CI
				 

				  

				 
				-0.159
				 

				  

				 
				0.435
				 

				  

				 
				—
				 

				  

				  

				  

				  

				  

				  

				  

				  

				  

				  

				  

				  

				  

				  

				  

				  

				  
			 

			 
				 
				 
				 

				  

				 
				Lower 95% CI
				 

				  

				 
				-0.361
				 

				  

				 
				0.243
				 

				  

				 
				—
				 

				  

				  

				  

				  

				  

				  

				  

				  

				  

				  

				  

				  

				  

				  

				  

				  

				  
			 

			 
				 
				4. SE
				 

				  

				 
				Pearson's r
				 

				  

				 
				0.657
				 

				 
				***
				 

				 
				-0.467
				 

				 
				***
				 

				 
				-0.282
				 

				 
				***
				 

				 
				—
				 

				  

				  

				  

				  

				  

				  

				  

				  

				  

				  

				  

				  

				  

				  

				  
			 

			 
				  

				  

				 
				BF₁₀
				 

				  

				 
				1.553e +38
				 

				  

				 
				1.070e +16
				 

				  

				 
				41349.019
				 

				  

				 
				—
				 

				  

				  

				  

				  

				  

				  

				  

				  

				  

				  

				  

				  

				  

				  

				  
			 

			 
				 
				 
				 

				  

				 
				Upper 95% CI
				 

				  

				 
				0.713
				 

				  

				 
				-0.376
				 

				  

				 
				-0.178
				 

				  

				 
				—
				 

				  

				  

				  

				  

				  

				  

				  

				  

				  

				  

				  

				  

				  

				  

				  
			 

			 
				 
				 
				 

				  

				 
				Lower 95% CI
				 

				  

				 
				0.588
				 

				  

				 
				-0.547
				 

				  

				 
				-0.378
				 

				  

				 
				—
				 

				  

				  

				  

				  

				  

				  

				  

				  

				  

				  

				  

				  

				  

				  

				  
			 

			 
				 
				5. LOT
				 

				  

				 
				Pearson's r
				 

				  

				 
				0.563
				 

				 
				***
				 

				 
				-0.455
				 

				 
				***
				 

				 
				-0.331
				 

				 
				***
				 

				 
				0.468
				 

				 
				***
				 

				 
				—
				 

				  

				  

				  

				  

				  

				  

				  

				  

				  

				  

				  

				  

				  
			 

			 
				  

				  

				 
				BF₁₀
				 

				  

				 
				2.480e +25
				 

				  

				 
				1.070e +15
				 

				  

				 
				8.403e 


				+6
				 

				  

				 
				1.117e +16
				 

				  

				 
				—
				 

				  

				  

				  

				  

				  

				  

				  

				  

				  

				  

				  

				  

				  
			 

			 
				 
				 
				 

				  

				 
				Upper 95% CI
				 

				  

				 
				0.631
				 

				  

				 
				-0.363
				 

				  

				 
				-0.229
				 

				  

				 
				0.547
				 

				  

				 
				—
				 

				  

				  

				  

				  

				  

				  

				  

				  

				  

				  

				  

				  

				  
			 

			 
				 
				 
				 

				  

				 
				Lower 95% CI
				 

				  

				 
				0.482
				 

				  

				 
				-0.536
				 

				  

				 
				-0.423
				 

				  

				 
				0.376
				 

				  

				 
				—
				 

				  

				  

				  

				  

				  

				  

				  

				  

				  

				  

				  

				  

				  
			 

			 
				 
				6. AV
				 

				  

				 
				Pearson's r
				 

				  

				 
				-0.305
				 

				 
				***
				 

				 
				0.367
				 

				 
				***
				 

				 
				0.430
				 

				 
				***
				 

				 
				-0.334
				 

				 
				***
				 

				 
				-0.275
				 

				 
				***
				 

				 
				—
				 

				  

				  

				  

				  

				  

				  

				  

				  

				  

				  

				  
			 

			 
				  

				  

				 
				BF₁₀
				 

				  

				 
				434967.892
				 

				  

				 
				8.325e 


				+8
				 

				  

				 
				1.092e +13
				 

				  

				 
				1.138e 


				+7
				 

				  

				 
				20576.463
				 

				  

				 
				—
				 

				  

				  

				  

				  

				  

				  

				  

				  

				  

				  

				  
			 

			 
				 
				 
				 

				  

				 
				Upper 95% CI
				 

				  

				 
				-0.202
				 

				  

				 
				0.456
				 

				  

				 
				0.513
				 

				  

				 
				-0.232
				 

				  

				 
				-0.170
				 

				  

				 
				—
				 

				  

				  

				  

				  

				  

				  

				  

				  

				  

				  

				  
			 

			 
				 
				 
				 

				  

				 
				Lower 95% CI
				 

				  

				 
				-0.399
				 

				  

				 
				0.268
				 

				  

				 
				0.335
				 

				  

				 
				-0.426
				 

				  

				 
				-0.371
				 

				  

				 
				—
				 

				  

				  

				  

				  

				  

				  

				  

				  

				  

				  

				  
			 

			 
				 
				7. AP
				 

				  

				 
				Pearson's r
				 

				  

				 
				0.351
				 

				 
				***
				 

				 
				-0.027
				 

				  

				 
				0.085
				 

				  

				 
				0.177
				 

				 
				*
				 

				 
				0.255
				 

				 
				***
				 

				 
				0.222
				 

				 
				***
				 

				 
				—
				 

				  

				  

				  

				  

				  

				  

				  

				  

				  
			 

			 
				  

				  

				 
				BF₁₀
				 

				  

				 
				9.824e 


				+7
				 

				  

				 
				0.078
				 

				  

				 
				0.220
				 

				  

				 
				11.167
				 

				  

				 
				3354.762
				 

				  

				 
				220.786
				 

				  

				 
				—
				 

				  

				  

				  

				  

				  

				  

				  

				  

				  
			 

			 
				 
				 
				 

				  

				 
				Upper 95% CI
				 

				  

				 
				0.442
				 

				  

				 
				0.082
				 

				  

				 
				0.191
				 

				  

				 
				0.279
				 

				  

				 
				0.353
				 

				  

				 
				0.321
				 

				  

				 
				—
				 

				  

				  

				  

				  

				  

				  

				  

				  

				  
			 

			 
				 
				 
				 

				  

				 
				Lower 95% CI
				 

				  

				 
				0.251
				 

				  

				 
				-0.135
				 

				  

				 
				-0.024
				 

				  

				 
				0.069
				 

				  

				 
				0.150
				 

				  

				 
				0.115
				 

				  

				 
				—
				 

				  

				  

				  

				  

				  

				  

				  

				  

				  
			 

			 
				 
				8. SC
				 

				  

				 
				Pearson's r
				 

				  

				 
				0.596
				 

				 
				***
				 

				 
				-0.527
				 

				 
				***
				 

				 
				-0.288
				 

				 
				***
				 

				 
				0.469
				 

				 
				***
				 

				 
				0.547
				 

				 
				***
				 

				 
				-0.403
				 

				 
				***
				 

				 
				0.227
				 

				 
				***
				 

				 
				—
				 

				  

				  

				  

				  

				  

				  

				  
			 

			 
				  

				  

				 
				BF₁₀
				 

				  

				 
				2.501e +29
				 

				  

				 
				3.673e +21
				 

				  

				 
				73985.916
				 

				  

				 
				1.390e +16
				 

				  

				 
				4.674e +23
				 

				  

				 
				1.413e +11
				 

				  

				 
				326.333
				 

				  

				 
				—
				 

				  

				  

				  

				  

				  

				  

				  
			 

			 
				 
				 
				 

				  

				 
				Upper 95% CI
				 

				  

				 
				0.660
				 

				  

				 
				-0.442
				 

				  

				 
				-0.184
				 

				  

				 
				0.548
				 

				  

				 
				0.618
				 

				  

				 
				-0.306
				 

				  

				 
				0.326
				 

				  

				 
				—
				 

				  

				  

				  

				  

				  

				  

				  
			 

			 
				 
				 
				 

				  

				 
				Lower 95% CI
				 

				  

				 
				0.519
				 

				  

				 
				-0.600
				 

				  

				 
				-0.383
				 

				  

				 
				0.378
				 

				  

				 
				0.464
				 

				  

				 
				-0.489
				 

				  

				 
				0.120
				 

				  

				 
				—
				 

				  

				  

				  

				  

				  

				  

				  
			 

			 
				 
				9. CS
				 

				  

				 
				Pearson's r
				 

				  

				 
				0.565
				 

				 
				***
				 

				 
				-0.355
				 

				 
				***
				 

				 
				-0.168
				 

				  

				 
				0.408
				 

				 
				***
				 

				 
				0.334
				 

				 
				***
				 

				 
				-0.101
				 

				  

				 
				0.272
				 

				 
				***
				 

				 
				0.383
				 

				 
				***
				 

				 
				—
				 

				  

				  

				  

				  

				  
			 

			 
				  

				  

				 
				BF₁₀
				 

				  

				 
				4.548e +25
				 

				  

				 
				1.614e 


				+8
				 

				  

				 
				6.755
				 

				  

				 
				3.336e +11
				 

				  

				 
				1.277e 


				+7
				 

				  

				 
				0.354
				 

				  

				 
				14667.284
				 

				  

				 
				8.144e 


				+9
				 

				  

				 
				—
				 

				  

				  

				  

				  

				  
			 

			 
				 
				 
				 

				  

				 
				Upper 95% CI
				 

				  

				 
				0.633
				 

				  

				 
				-0.254
				 

				  

				 
				-0.060
				 

				  

				 
				0.493
				 

				  

				 
				0.426
				 

				  

				 
				0.009
				 

				  

				 
				0.368
				 

				  

				 
				0.471
				 

				  

				 
				—
				 

				  

				  

				  

				  

				  
			 

			 
				 
				 
				 

				  

				 
				Lower 95% CI
				 

				  

				 
				0.484
				 

				  

				 
				-0.445
				 

				  

				 
				-0.270
				 

				  

				 
				0.311
				 

				  

				 
				0.233
				 

				  

				 
				-0.206
				 

				  

				 
				0.167
				 

				  

				 
				0.285
				 

				  

				 
				—
				 

				  

				  

				  

				  

				  
			 

			 
				 
				10. BU
				 

				  

				 
				Pearson's r
				 

				  

				 
				-0.621
				 

				 
				***
				 

				 
				0.455
				 

				 
				***
				 

				 
				0.375
				 

				 
				***
				 

				 
				-0.451
				 

				 
				***
				 

				 
				-0.494
				 

				 
				***
				 

				 
				0.325
				 

				 
				***
				 

				 
				-0.249
				 

				 
				***
				 

				 
				-0.528
				 

				 
				***
				 

				 
				-0.666
				 

				 
				***
				 

				 
				—
				 

				  

				  

				  
			 

			 
				  

				  

				 
				BF₁₀
				 

				  

				 
				6.272e +32
				 

				  

				 
				1.061e +15
				 

				  

				 
				2.548e 


				+9
				 

				  

				 
				4.522e +14
				 

				  

				 
				2.041e +18
				 

				  

				 
				4.099e 


				+6
				 

				  

				 
				1923.237
				 

				  

				 
				4.419e +21
				 

				  

				 
				4.543e +39
				 

				  

				 
				—
				 

				  

				  

				  
			 

			 
				 
				 
				 

				  

				 
				Upper 95% CI
				 

				  

				 
				-0.547
				 

				  

				 
				0.536
				 

				  

				 
				0.463
				 

				  

				 
				-0.358
				 

				  

				 
				-0.405
				 

				  

				 
				0.418
				 

				  

				 
				-0.143
				 

				  

				 
				-0.443
				 

				  

				 
				-0.598
				 

				  

				 
				—
				 

				  

				  

				  
			 

			 
				 
				 
				 

				  

				 
				Lower 95% CI
				 

				  

				 
				-0.682
				 

				  

				 
				0.363
				 

				  

				 
				0.276
				 

				  

				 
				-0.531
				 

				  

				 
				-0.570
				 

				  

				 
				0.223
				 

				  

				 
				-0.347
				 

				  

				 
				-0.601
				 

				  

				 
				-0.721
				 

				  

				 
				—
				 

				  

				  

				  
			 

			 
				 
				11. STS
				 

				  

				 
				Pearson's r
				 

				  

				 
				-0.239
				 

				 
				***
				 

				 
				0.406
				 

				 
				***
				 

				 
				0.333
				 

				 
				***
				 

				 
				-0.321
				 

				 
				***
				 

				 
				-0.230
				 

				 
				***
				 

				 
				0.361
				 

				 
				***
				 

				 
				4.978e -4
				 

				  

				 
				-0.305
				 

				 
				***
				 

				 
				-0.159
				 

				  

				 
				0.468
				 

				 
				***
				 

				 
				—
				 

				  
			 

			 
				  

				  

				 
				BF₁₀
				 

				  

				 
				858.561
				 

				  

				 
				2.407e +11
				 

				  

				 
				1.053e 


				+7
				 

				  

				 
				2.745e 


				+6
				 

				  

				 
				431.443
				 

				  

				 
				3.755e 


				+8
				 

				  

				 
				0.070
				 

				  

				 
				464121.384
				 

				  

				 
				4.219
				 

				  

				 
				1.292e +16
				 

				  

				 
				—
				 

				  
			 

			 
				 
				 
				 

				  

				 
				Upper 95% CI
				 

				  

				 
				-0.133
				 

				  

				 
				0.491
				 

				  

				 
				0.425
				 

				  

				 
				-0.219
				 

				  

				 
				-0.124
				 

				  

				 
				0.451
				 

				  

				 
				0.109
				 

				  

				 
				-0.202
				 

				  

				 
				-0.051
				 

				  

				 
				0.547
				 

				  

				 
				—
				 

				  
			 

			 
				 
				 
				 

				  

				 
				Lower 95% CI
				 

				  

				 
				-0.337
				 

				  

				 
				0.309
				 

				  

				 
				0.231
				 

				  

				 
				-0.414
				 

				  

				 
				-0.329
				 

				  

				 
				0.262
				 

				  

				 
				-0.108
				 

				  

				 
				-0.399
				 

				  

				 
				-0.262
				 

				  

				 
				0.377
				 

				  

				 
				—
				 

				  
			 

			 
				  
			 
		 

		 
			 
				 
				*  BF₁₀  > 10, ** BF₁₀  > 30, *** BF₁₀  > 100
				 
			 
		 
	 
 
  The results indicate medium to strong correlations between all variables excluding associations between AP and PSS (r=-0.03, BF 10  =0.08, 95% CI [0.08, -0.14]); AP and RSQ (r=0.09, BF 10  =0.22, 95%CI [0.19, -0.02]), RSQ and CS (r=-0.17, BF 10  =6.76, 95% CI [-0.06, -0.27]), AV and CS (r=0.10, BF 10  =0.35, 95% CI [0.01, -0.21])            
 
  Regression model 1. RSQ as predictor for RESIL    Predictor: RSQ, outcome: Resilience (RESIL)   
 
   
 
 
	 
		 
			 
				 
					 
						 
							 
							Model Summary - RESIL
							 

							  

							  
						 
					 
				 
			 

			 
				 
				Model
				 

				 
				R
				 

				 
				R²
				 

				 
				Adjusted R²
				 

				 
				RMSE
				 
			 
		 

		 
			 
				 
				H₀
				 

				  

				 
				0.000
				 

				  

				 
				0.000
				 

				  

				 
				0.000
				 

				  

				 
				11.215
				 

				  

			 

			 
				 
				H₁
				 

				  

				 
				0.264
				 

				  

				 
				0.070
				 

				  

				 
				0.067
				 

				  

				 
				10.834
				 

				  
			 

			 
				  
			 
		 

		  
	 
 
  RSQ can explain only 6.7 of variance of resilience     
 
 
	 
		 
			 
				 
					 
						 
							 
							ANOVA
							 

							  

							  
						 
					 
				 
			 

			 
				 
				Model
				 

				 
				 
				 

				 
				Sum of Squares
				 

				 
				df
				 

				 
				Mean Square
				 

				 
				F
				 

				 
				p
				 
			 
		 

		 
			 
				 
				H₁
				 

				  

				 
				Regression
				 

				  

				 
				2838.763
				 

				  

				 
				1
				 

				  

				 
				2838.763
				 

				  

				 
				24.187
				 

				  

				 
				< .001
				 

				  

			 

			 
				 
				 
				 

				  

				 
				Residual
				 

				  

				 
				37909.464
				 

				  

				 
				323
				 

				  

				 
				117.367
				 

				  

				  

				  

				 
				 
				 

				  
			 

			 
				 
				 
				 

				  

				 
				Total
				 

				  

				 
				40748.228
				 

				  

				 
				324
				 

				  

				  

				  

				  

				  

				 
				 
				 

				  
			 

			 
				  
			 
		 

		 
			 
				 
					 
					Note.
					 

				 The intercept model is omitted, as no meaningful information can be shown.
				 
			 
		 
	 
 
   
 
 
	 
		 
			 
				 
					 
						 
							 
							Coefficients
							 

							  

							  
						 
					 
				 
			 

			 
				  

				 
				95% CI
				 
			 

			 
				 
				Model
				 

				 
				 
				 

				 
				Unstandardized
				 

				 
				Standard Error
				 

				 
				Standardized
				 

				 
				t
				 

				 
				p
				 

				 
				Lower
				 

				 
				Upper
				 
			 
		 

		 
			 
				 
				H₀
				 

				  

				 
				(Intercept)
				 

				  

				 
				0.004
				 

				  

				 
				0.622
				 

				  

				  

				  

				 
				0.006
				 

				  

				 
				0.995
				 

				  

				 
				-1.220
				 

				  

				 
				1.228
				 

				  

			 

			 
				 
				H₁
				 

				  

				 
				(Intercept)
				 

				  

				 
				0.007
				 

				  

				 
				0.601
				 

				  

				  

				  

				 
				0.011
				 

				  

				 
				0.991
				 

				  

				 
				-1.176
				 

				  

				 
				1.189
				 

				  
			 

			 
				 
				 
				 

				  

				 
				RSQ
				 

				  

				 
				-0.405
				 

				  

				 
				0.082
				 

				  

				 
				-0.264
				 

				  

				 
				-4.918
				 

				  

				 
				< .001
				 

				  

				 
				-0.567
				 

				  

				 
				-0.243
				 

				  
			 

			 
				  
			 
		 

		  
	 
 
   
 
  Residuals vs. Covariates    All plots below indicate that there is no systematic errors and big outliers. A Q-Q plot shows the quantiles of a theoretical normal distribution against the observed quantiles of the residuals. If the observed residuals are approximately normal, then all points in the plot fall approximately on a straight line. In our case, the assumptions are not badly violated.   
 
  Residuals vs. RSQ         
 
  Residuals vs. Predicted       
 
  Standardized Residuals Histogram       
 
  Q-Q Plot Standardized Residuals       
 
  Partial Regression Plot   
 
  RESIL vs. RSQ                  
 
  Regression model 2. PSS as predictor for RESIL    Predictor: PSS, outcome RESIL   
 
   
 
 
	 
		 
			 
				 
					 
						 
							 
							Model Summary - RESIL
							 

							  

							  
						 
					 
				 
			 

			 
				 
				Model
				 

				 
				R
				 

				 
				R²
				 

				 
				Adjusted R²
				 

				 
				RMSE
				 
			 
		 

		 
			 
				 
				H₀
				 

				  

				 
				0.000
				 

				  

				 
				0.000
				 

				  

				 
				0.000
				 

				  

				 
				11.215
				 

				  

			 

			 
				 
				H₁
				 

				  

				 
				0.460
				 

				  

				 
				0.212
				 

				  

				 
				0.209
				 

				  

				 
				9.972
				 

				  
			 

			 
				  
			 
		 

		  
	 
 
  PSS can explain 21% of variance of resilience     
 
 
	 
		 
			 
				 
					 
						 
							 
							ANOVA
							 

							  

							  
						 
					 
				 
			 

			 
				 
				Model
				 

				 
				 
				 

				 
				Sum of Squares
				 

				 
				df
				 

				 
				Mean Square
				 

				 
				F
				 

				 
				p
				 
			 
		 

		 
			 
				 
				H₁
				 

				  

				 
				Regression
				 

				  

				 
				8628.878
				 

				  

				 
				1
				 

				  

				 
				8628.878
				 

				  

				 
				86.774
				 

				  

				 
				< .001
				 

				  

			 

			 
				 
				 
				 

				  

				 
				Residual
				 

				  

				 
				32119.350
				 

				  

				 
				323
				 

				  

				 
				99.441
				 

				  

				  

				  

				 
				 
				 

				  
			 

			 
				 
				 
				 

				  

				 
				Total
				 

				  

				 
				40748.228
				 

				  

				 
				324
				 

				  

				  

				  

				  

				  

				 
				 
				 

				  
			 

			 
				  
			 
		 

		 
			 
				 
					 
					Note.
					 

				 The intercept model is omitted, as no meaningful information can be shown.
				 
			 
		 
	 
 
   
 
 
	 
		 
			 
				 
					 
						 
							 
							Coefficients
							 

							  

							  
						 
					 
				 
			 

			 
				  

				 
				95% CI
				 
			 

			 
				 
				Model
				 

				 
				 
				 

				 
				Unstandardized
				 

				 
				Standard Error
				 

				 
				Standardized
				 

				 
				t
				 

				 
				p
				 

				 
				Lower
				 

				 
				Upper
				 
			 
		 

		 
			 
				 
				H₀
				 

				  

				 
				(Intercept)
				 

				  

				 
				0.004
				 

				  

				 
				0.622
				 

				  

				  

				  

				 
				0.006
				 

				  

				 
				0.995
				 

				  

				 
				-1.220
				 

				  

				 
				1.228
				 

				  

			 

			 
				 
				H₁
				 

				  

				 
				(Intercept)
				 

				  

				 
				0.006
				 

				  

				 
				0.553
				 

				  

				  

				  

				 
				0.011
				 

				  

				 
				0.992
				 

				  

				 
				-1.082
				 

				  

				 
				1.094
				 

				  
			 

			 
				 
				 
				 

				  

				 
				PSS
				 

				  

				 
				-0.856
				 

				  

				 
				0.092
				 

				  

				 
				-0.460
				 

				  

				 
				-9.315
				 

				  

				 
				< .001
				 

				  

				 
				-1.037
				 

				  

				 
				-0.675
				 

				  
			 

			 
				  
			 
		 

		  
	 
 
   
 
  Residuals vs. Covariates    assumption are not badly violated (see plots below)   
 
  Residuals vs. PSS         
 
  Residuals vs. Predicted       
 
  Standardized Residuals Histogram       
 
  Q-Q Plot Standardized Residuals       
 
  Partial Regression Plot   
 
  RESIL vs. PSS                  
 
  Regression Model 3. RSQ & PSS as predictors for RESIL    Predictors: RSQ and PSS. Outcome RESIL   
 
   
 
 
	 
		 
			 
				 
					 
						 
							 
							Model Summary - RESIL
							 

							  

							  
						 
					 
				 
			 

			 
				 
				Model
				 

				 
				R
				 

				 
				R²
				 

				 
				Adjusted R²
				 

				 
				RMSE
				 
			 
		 

		 
			 
				 
				H₀
				 

				  

				 
				0.000
				 

				  

				 
				0.000
				 

				  

				 
				0.000
				 

				  

				 
				11.215
				 

				  

			 

			 
				 
				H₁
				 

				  

				 
				0.474
				 

				  

				 
				0.224
				 

				  

				 
				0.220
				 

				  

				 
				9.907
				 

				  
			 

			 
				  
			 
		 

		  
	 
 
  a model with two predictors can explain 22% of the variance of resilience     
 
 
	 
		 
			 
				 
					 
						 
							 
							ANOVA
							 

							  

							  
						 
					 
				 
			 

			 
				 
				Model
				 

				 
				 
				 

				 
				Sum of Squares
				 

				 
				df
				 

				 
				Mean Square
				 

				 
				F
				 

				 
				p
				 
			 
		 

		 
			 
				 
				H₁
				 

				  

				 
				Regression
				 

				  

				 
				9144.634
				 

				  

				 
				2
				 

				  

				 
				4572.317
				 

				  

				 
				46.586
				 

				  

				 
				< .001
				 

				  

			 

			 
				 
				 
				 

				  

				 
				Residual
				 

				  

				 
				31603.594
				 

				  

				 
				322
				 

				  

				 
				98.148
				 

				  

				  

				  

				 
				 
				 

				  
			 

			 
				 
				 
				 

				  

				 
				Total
				 

				  

				 
				40748.228
				 

				  

				 
				324
				 

				  

				  

				  

				  

				  

				 
				 
				 

				  
			 

			 
				  
			 
		 

		 
			 
				 
					 
					Note.
					 

				 The intercept model is omitted, as no meaningful information can be shown.
				 
			 
		 
	 
 
   
 
 
	 
		 
			 
				 
					 
						 
							 
							Coefficients
							 

							  

							  
						 
					 
				 
			 

			 
				  

				 
				95% CI
				 
			 

			 
				 
				Model
				 

				 
				 
				 

				 
				Unstandardized
				 

				 
				Standard Error
				 

				 
				Standardized
				 

				 
				t
				 

				 
				p
				 

				 
				Lower
				 

				 
				Upper
				 
			 
		 

		 
			 
				 
				H₀
				 

				  

				 
				(Intercept)
				 

				  

				 
				0.004
				 

				  

				 
				0.622
				 

				  

				  

				  

				 
				0.006
				 

				  

				 
				0.995
				 

				  

				 
				-1.220
				 

				  

				 
				1.228
				 

				  

			 

			 
				 
				H₁
				 

				  

				 
				(Intercept)
				 

				  

				 
				0.007
				 

				  

				 
				0.550
				 

				  

				  

				  

				 
				0.013
				 

				  

				 
				0.990
				 

				  

				 
				-1.074
				 

				  

				 
				1.088
				 

				  
			 

			 
				 
				 
				 

				  

				 
				RSQ
				 

				  

				 
				-0.184
				 

				  

				 
				0.080
				 

				  

				 
				-0.120
				 

				  

				 
				-2.292
				 

				  

				 
				0.023
				 

				  

				 
				-0.342
				 

				  

				 
				-0.026
				 

				  
			 

			 
				 
				 
				 

				  

				 
				PSS
				 

				  

				 
				-0.779
				 

				  

				 
				0.097
				 

				  

				 
				-0.419
				 

				  

				 
				-8.016
				 

				  

				 
				< .001
				 

				  

				 
				-0.970
				 

				  

				 
				-0.588
				 

				  
			 

			 
				  
			 
		 

		  
	 
 
   
 
  Residuals vs. Covariates   
 
  Residuals vs. RSQ       
 
  Residuals vs. PSS         
 
  Residuals vs. Predicted       
 
  Standardized Residuals Histogram       
 
  Q-Q Plot Standardized Residuals       
 
  Partial Regression Plots   
 
  RESIL vs. RSQ       
 
  RESIL vs. PSS            At first glance, the Regression model 3 can better account for the variance in resilience. However, the coefficient of determination R 2  is not a good measure for model comparison because it does not penalize models for complexity: when additional predictors are added to a model, R 2  can only increase. Therefore, R 2  will always favor the most complex model. This makes R 2  unsuitable for model selection, unless models have the same number of predictors. Therefore, we will use Bayeasin Inference for model selection.     
 
  Bayesian Inference for model selection RSQ and PSS as predictors of RESIL)    When multiple models are in play, we can extend Bayes’ theorem and use the data to update the relative plausibility of each of the candidate models.   As we have no previous studies that could indicate us prior believes, we will assign default priors (i.e., the relative plausibility of models before seeing the data) . The change from prior to posterior odds (i.e., the relative plausibility of models after seeing the data) is given by the Bayes factor (e.g.,  Jeffreys ,  1961 ;  Kass & Raftery ,  1995 ), which indicates the models’ relative predictive performance for the data at hand (i.e., the ratio of marginal likelihoods).   Using default priors:  we used the Jeffreys–Zellner–Siow (JZS) prior.  The JZS prior fulfills several desiderata (  Rouder & Morey  ,   2012;      Ly, Verhagen, & Wagenmakers  ,   2016). Moreover, t he sample size for our data is 324, which is relatively big. In the case of the big dataset, the influence of the prior is relatively small.  As we have only 2 predictors, we will compare three possible models (PSS), (RSQ) and (PSS+RSQ) with the null model.   
 
   
 
 
	 
		 
			 
				 
					 
						 
							 
							Model Comparison - RESIL
							 

							  

							  
						 
					 
				 
			 

			 
				 
				Models
				 

				 
				P(M)
				 

				 
				P(M|data)
				 

				 
				BF

					 
					M
					 
				 

				 
				BF

					 
					10
					 
				 

				 
				R²
				 
			 
		 

		 
			 
				 
				Null model
				 

				  

				 
				0.250
				 

				  

				 
				1.849e -16
				 

				  

				 
				5.547e -16
				 

				  

				 
				1.000
				 

				  

				 
				0.000
				 

				  

			 

			 
				 
				RSQ + PSS
				 

				  

				 
				0.250
				 

				  

				 
				0.587
				 

				  

				 
				4.266
				 

				  

				 
				3.175e +15
				 

				  

				 
				0.224
				 

				  
			 

			 
				 
				PSS
				 

				  

				 
				0.250
				 

				  

				 
				0.413
				 

				  

				 
				2.110
				 

				  

				 
				2.233e +15
				 

				  

				 
				0.212
				 

				  
			 

			 
				 
				RSQ
				 

				  

				 
				0.250
				 

				  

				 
				1.757e -12
				 

				  

				 
				5.270e -12
				 

				  

				 
				9501.186
				 

				  

				 
				0.070
				 

				  
			 

			 
				  
			 
		 

		  
	 
 
  The Bayes factor BF 10  (H1) for model with both predictors is large and indicates that the data are 3.15e+15 times more likely under (RSQ + PSS) model compared to the null model (H0). However, the Bayes factor for two other models with only one predictor (PSS or RSQ) is also large (BF10 >100) that indicated strong evidence in favour of the H1 hypothesis. Therefore, the model comparison statistics are not informative in this case.  We will then asses the Posterior Summary table which quantifies the relevance of individual predictors.      
 
  Posterior Summary      Model-averaged posterior summary for linear regression coefficients     Short annotation to the table:   Coefficient  - predictors   Mean and SD  - represent the respective posterior mean and standard deviation of the parameter after model averaging    P (incl)  denotes the prior inclusion probability    P (incl | data)  denotes the posterior inclusion probability   BFinclusion  - the change from prior to posterior inclusion odds   a  95% central credible interval  (CI) for the parameters   The results indicate that each of these predictors are relevant for predicting resilience, as indicated by the fact that the posterior inclusion probabilities for PSS is =1.00 and for RSQ = 0.74. Although, the posterior inclusion probability for RSQ is not as high as we would expect*, the relevance of this predictor is evident bacause the data increased the inclusion probability from 0.5 to 0.74 (see Inclusion Probabilities plot below. The dashed line represents the prior inclusion probabilities ).   The Q-Q plot (below) shows that the standardized residuals fit fairly well along the diagonal suggesting that both assumptions or normality and linearity have also not been violated.   *The inclusion Bayes factor quantifies how much the observed data are more probable under models that include a particular predictor relative to the models that do not contain that particular predictor.  In case of RSQ,  across all the candidate models, the model with the RSQ variable is, on average, about only 1.42 times more likely than the model without the RSQ variable.   
 
 
	 
		 
			 
				 
					 
						 
							 
							Posterior Summaries of Coefficients
							 

							  

							  
						 
					 
				 
			 

			 
				  

				 
				95% Credible Interval
				 
			 

			 
				 
				Coefficient
				 

				 
				Mean
				 

				 
				SD
				 

				 
				P(incl)
				 

				 
				P(incl|data)
				 

				 
				BF

					 
					inclusion
					 
				 

				 
				Lower
				 

				 
				Upper
				 
			 
		 

		 
			 
				 
				Intercept
				 

				  

				 
				0.004
				 

				  

				 
				0.550
				 

				  

				 
				1.000
				 

				  

				 
				1.000
				 

				  

				 
				1.000
				 

				  

				 
				-1.077
				 

				  

				 
				1.085
				 

				  

			 

			 
				 
				RSQ
				 

				  

				 
				-0.180
				 

				  

				 
				0.079
				 

				  

				 
				0.500
				 

				  

				 
				0.587
				 

				  

				 
				1.422
				 

				  

				 
				-0.336
				 

				  

				 
				-0.024
				 

				  
			 

			 
				 
				PSS
				 

				  

				 
				-0.762
				 

				  

				 
				0.096
				 

				  

				 
				0.500
				 

				  

				 
				1.000
				 

				  

				 
				5.692e +11
				 

				  

				 
				-0.951
				 

				  

				 
				-0.573
				 

				  
			 

			 
				  
			 
		 

		  
	 
 
  The posterior mean of the regression coefficient of RSQ is -0.18. We can interpret this value such that a one-unit increase in RSQ adds about 0.18 units in decreasing resilience. The 95% credible interval of RSQ is [-0.34, -0.02], which means that there is a 95% probability that the regression coefficient of RSQ lies in the population with the corresponding credible interval. The 95% credible interval does not contain 0. This shows the evidence of the effect of RSQ in predicting the level of resilience.   The posterior mean of the regression coefficient of PSS is -0.76 indicating that a one-unit increase in PSS adds about 0.76 units in decreasing resilience. The 95% credible interval of PSS is [-0.95, -0.57], which means that there is a 95% probability that the regression coefficient of PSS lies in the population with the corresponding credible interval. The 95% credible interval does not contain 0. This shows the evidence of the effect of PSS in predicting the level of resilience.       
 
  Inclusion Probabilities       
 
  Q-Q Plot       
 
  Marginal Posterior Distributions    The complete model-averaged posteriors are be visualized below. The pike at zero in RSQ plot corresponds to the absence of an effect, and its height reflects the predictor’s posterior exclusion probability. The horizontal bars depicted a 95% credible interval for each predictor.   Following recommendations by van Doorn at al (2019), we further investigated the robustness of the results against the choice of prior by using wide and ultrawide priors. Therefore, we repeated this analysis using scale of 1/4 and 1/2 but the result did not change in a meaningful way in both cases.     
 
  Intercept       
 
  RSQ       
 
  PSS            To summarize, the Bayesian model-averaged analysis showed that the most important predictor of resilience is the PSS. However, we cannot fully exclude the RSQ as there is still relevance for predicting the level of resilience. A one-unit increase in RSQ adds about 0.18 units in decreasing resilience. A one-unit increase in PSS adds about 0.76 units in decreasing resilience. Therefore, this analysis shows the evidence of the effects of both PSS and RSQ in predicting the level of resilience.     
 
  RSQ and PSS as predictors of SE    We next tested ther relationship between either predictors (RSQ and PSS) and potential mediators (i.e., AV, AP, SE, LOT, SC, STS, BU, CS) We, first, tested whether RSQ and PSS could predict AV, AP, SE, LOT, SC, STS, BU, CS  using a multiple regression analysis to gather an idea which independent variables will create the best prediction equation. We performed a seria of regression analyses with each of possible mediators as a dependent variable and RSQ and PSS as predictors. Each analysis we supplemented with Bayeasian inferences to gather evidence of each prediction.   In summary, the analyses below showed that    RSQ and PSS are not reliable predictors of AP   RSQ is not reliable predictors of SC and CS while PSS could reliably predict these variables    
 
   
 
 
	 
		 
			 
				 
					 
						 
							 
							Model Summary - SE
							 

							  

							  
						 
					 
				 
			 

			 
				  

				 
				Durbin-Watson
				 
			 

			 
				 
				Model
				 

				 
				R
				 

				 
				R²
				 

				 
				Adjusted R²
				 

				 
				RMSE
				 

				 
				R² Change
				 

				 
				F Change
				 

				 
				df1
				 

				 
				df2
				 

				 
				p
				 

				 
				Autocorrelation
				 

				 
				Statistic
				 

				 
				p
				 
			 
		 

		 
			 
				 
				H₀
				 

				  

				 
				0.000
				 

				  

				 
				0.000
				 

				  

				 
				0.000
				 

				  

				 
				3.728
				 

				  

				 
				0.000
				 

				  

				  

				  

				 
				0
				 

				  

				 
				324
				 

				  

				 
				 
				 

				  

				 
				-0.049
				 

				  

				 
				2.098
				 

				  

				 
				0.374
				 

				  

			 

			 
				 
				H₁
				 

				  

				 
				0.485
				 

				  

				 
				0.235
				 

				  

				 
				0.230
				 

				  

				 
				3.270
				 

				  

				 
				0.235
				 

				  

				 
				49.503
				 

				  

				 
				2
				 

				  

				 
				322
				 

				  

				 
				< .001
				 

				  

				 
				-0.059
				 

				  

				 
				2.116
				 

				  

				 
				0.296
				 

				  
			 

			 
				  
			 
		 

		  
	 
 
  The adjusted R 2  (we are using the adjusted value for multiple predictors) shows that the predictors can explain 23%% of the outcome variance.   Durbin-Watson checks for correlations between residuals is between 1 and 3 as required.      
 
 
	 
		 
			 
				 
					 
						 
							 
							ANOVA
							 

							  

							  
						 
					 
				 
			 

			 
				 
				Model
				 

				 
				 
				 

				 
				Sum of Squares
				 

				 
				df
				 

				 
				Mean Square
				 

				 
				F
				 

				 
				p
				 
			 
		 

		 
			 
				 
				H₁
				 

				  

				 
				Regression
				 

				  

				 
				1058.871
				 

				  

				 
				2
				 

				  

				 
				529.436
				 

				  

				 
				49.503
				 

				  

				 
				< .001
				 

				  

			 

			 
				 
				 
				 

				  

				 
				Residual
				 

				  

				 
				3443.818
				 

				  

				 
				322
				 

				  

				 
				10.695
				 

				  

				  

				  

				 
				 
				 

				  
			 

			 
				 
				 
				 

				  

				 
				Total
				 

				  

				 
				4502.689
				 

				  

				 
				324
				 

				  

				  

				  

				  

				  

				 
				 
				 

				  
			 

			 
				  
			 
		 

		 
			 
				 
					 
					Note.
					 

				 The intercept model is omitted, as no meaningful information can be shown.
				 
			 
		 
	 
 
   
 
 
	 
		 
			 
				 
					 
						 
							 
							Coefficients
							 

							  

							  
						 
					 
				 
			 

			 
				 
					  
				 

				 
					 
					95% CI
					 
				 

				 
					 
					Collinearity Statistics
					 
				 
			 

			 
				 
				Model
				 

				 
				 
				 

				 
				Unstandardized
				 

				 
				Standard Error
				 

				 
				Standardized
				 

				 
				t
				 

				 
				p
				 

				 
				Lower
				 

				 
				Upper
				 

				 
				Tolerance
				 

				 
				VIF
				 
			 
		 

		 
			 
				 
				H₀
				 

				  

				 
				(Intercept)
				 

				  

				 
				0.018
				 

				  

				 
				0.207
				 

				  

				  

				  

				 
				0.089
				 

				  

				 
				0.929
				 

				  

				 
				-0.388
				 

				  

				 
				0.425
				 

				  

				 
				 
				 

				  

				  

				  

			 

			 
				 
				H₁
				 

				  

				 
				(Intercept)
				 

				  

				 
				0.020
				 

				  

				 
				0.181
				 

				  

				  

				  

				 
				0.108
				 

				  

				 
				0.914
				 

				  

				 
				-0.337
				 

				  

				 
				0.376
				 

				  

				 
				 
				 

				  

				  

				  
			 

			 
				 
				 
				 

				  

				 
				PSS
				 

				  

				 
				-0.260
				 

				  

				 
				0.032
				 

				  

				 
				-0.420
				 

				  

				 
				-8.095
				 

				  

				 
				< .001
				 

				  

				 
				-0.323
				 

				  

				 
				-0.197
				 

				  

				 
				0.882
				 

				  

				 
				1.134
				 

				  
			 

			 
				 
				 
				 

				  

				 
				RSQ
				 

				  

				 
				-0.070
				 

				  

				 
				0.026
				 

				  

				 
				-0.137
				 

				  

				 
				-2.647
				 

				  

				 
				0.009
				 

				  

				 
				-0.122
				 

				  

				 
				-0.018
				 

				  

				 
				0.882
				 

				  

				 
				1.134
				 

				  
			 

			 
				  
			 
		 

		  
	 
 
  Both tolerance and VIF are acceptable.      
 
 
	 
		 
			 
				 
					 
						 
							 
							Collinearity Diagnostics
							 

							  

							  
						 
					 
				 
			 

			 
				  

				 
				Variance Proportions
				 
			 

			 
				 
				Model
				 

				 
				Dimension
				 

				 
				Eigenvalue
				 

				 
				Condition Index
				 

				 
				(Intercept)
				 

				 
				PSS
				 

				 
				RSQ
				 
			 
		 

		 
			 
				 
				H₁
				 

				  

				 
				1
				 

				  

				 
				1.344
				 

				  

				 
				1.000
				 

				  

				 
				0.000
				 

				  

				 
				0.328
				 

				  

				 
				0.328
				 

				  

			 

			 
				 
				 
				 

				  

				 
				2
				 

				  

				 
				1.000
				 

				  

				 
				1.159
				 

				  

				 
				1.000
				 

				  

				 
				0.000
				 

				  

				 
				0.000
				 

				  
			 

			 
				 
				 
				 

				  

				 
				3
				 

				  

				 
				0.656
				 

				  

				 
				1.431
				 

				  

				 
				0.000
				 

				  

				 
				0.672
				 

				  

				 
				0.672
				 

				  
			 

			 
				  
			 
		 

		 
			 
				 
					 
					Note.
					 

				 The intercept model is omitted, as no meaningful information can be shown.
				 
			 
		 
	 
 
   
 
 
	 
		 
			 
				 
					 
						 
							 
							Casewise Diagnostics
							 

							  

							  
						 
					 
				 
			 

			 
				 
				Case Number
				 

				 
				Std. Residual
				 

				 
				SE
				 

				 
				Predicted Value
				 

				 
				Residual
				 

				 
				Cook's Distance
				 
			 
		 

		 
			 
				 
				97
				 

				  

				 
				-3.956
				 

				  

				 
				-18.600
				 

				  

				 
				-5.952
				 

				  

				 
				-12.648
				 

				  

				 
				0.240
				 

				  

			 

			 
				  
			 
		 

		  
	 
 
  The casewise diagnostic indicates that our predictor (RSQ) has residuals which are 3 or more standard deviations away from the mean.   However, assessing data integrity of RSQ suggests small deviation of normality at the very left tail indicating that some of our participants indicated high level of perceived stress related to COVID-19 in participant with ID 97.      
 
  Residuals vs. Predicted       
 
  Q-Q Plot Standardized Residuals          The quite balanced distribution of the residuals around the baseline suggests that the assumption of homoscedasticity has not been badly violated. The Q-Q plot shows that the standardized residuals fit along the diagonal suggesting that both assumptions or normality and linearity have also not been badly violated.      
 
  RSQ and PSS as predictors of SE. Bayesian inference   
 
   
 
 
	 
		 
			 
				 
					 
						 
							 
							Model Comparison - SE
							 

							  

							  
						 
					 
				 
			 

			 
				 
				Models
				 

				 
				P(M)
				 

				 
				P(M|data)
				 

				 
				BF

					 
					M
					 
				 

				 
				BF

					 
					10
					 
				 

				 
				R²
				 
			 
		 

		 
			 
				 
				Null model
				 

				  

				 
				0.333
				 

				  

				 
				3.036e -17
				 

				  

				 
				6.072e -17
				 

				  

				 
				1.000
				 

				  

				 
				0.000
				 

				  

			 

			 
				 
				PSS + RSQ
				 

				  

				 
				0.333
				 

				  

				 
				0.867
				 

				  

				 
				13.034
				 

				  

				 
				2.856e +16
				 

				  

				 
				0.235
				 

				  
			 

			 
				 
				PSS
				 

				  

				 
				0.167
				 

				  

				 
				0.133
				 

				  

				 
				0.767
				 

				  

				 
				8.764e +15
				 

				  

				 
				0.219
				 

				  
			 

			 
				 
				RSQ
				 

				  

				 
				0.167
				 

				  

				 
				7.644e -13
				 

				  

				 
				3.822e -12
				 

				  

				 
				50356.710
				 

				  

				 
				0.080
				 

				  
			 

			 
				  
			 
		 

		  
	 
 
   
 
  Posterior Summary   
 
 
	 
		 
			 
				 
					 
						 
							 
							Posterior Summaries of Coefficients
							 

							  

							  
						 
					 
				 
			 

			 
				  

				 
				95% Credible Interval
				 
			 

			 
				 
				Coefficient
				 

				 
				Mean
				 

				 
				SD
				 

				 
				P(incl)
				 

				 
				P(incl|data)
				 

				 
				BF

					 
					inclusion
					 
				 

				 
				Lower
				 

				 
				Upper
				 
			 
		 

		 
			 
				 
				Intercept
				 

				  

				 
				0.018
				 

				  

				 
				0.182
				 

				  

				 
				1.000
				 

				  

				 
				1.000
				 

				  

				 
				1.000
				 

				  

				 
				-0.318
				 

				  

				 
				0.384
				 

				  

			 

			 
				 
				PSS
				 

				  

				 
				-0.258
				 

				  

				 
				0.033
				 

				  

				 
				0.500
				 

				  

				 
				1.000
				 

				  

				 
				1.308e +12
				 

				  

				 
				-0.318
				 

				  

				 
				-0.189
				 

				  
			 

			 
				 
				RSQ
				 

				  

				 
				-0.059
				 

				  

				 
				0.034
				 

				  

				 
				0.500
				 

				  

				 
				0.867
				 

				  

				 
				6.517
				 

				  

				 
				-0.110
				 

				  

				 
				0.000
				 

				  
			 

			 
				  
			 
		 

		  
	 
 
     
 
  Residuals vs Fitted       
 
  Inclusion Probabilities       
 
  Q-Q Plot       
 
  Marginal Posterior Distributions   
 
  Intercept       
 
  PSS       
 
  RSQ             
 
  RSQ and PSS as predictors of AV   
 
   
 
 
	 
		 
			 
				 
					 
						 
							 
							Model Summary - AV
							 

							  

							  
						 
					 
				 
			 

			 
				 
				Model
				 

				 
				R
				 

				 
				R²
				 

				 
				Adjusted R²
				 

				 
				RMSE
				 
			 
		 

		 
			 
				 
				H₀
				 

				  

				 
				0.000
				 

				  

				 
				0.000
				 

				  

				 
				0.000
				 

				  

				 
				4.084
				 

				  

			 

			 
				 
				H₁
				 

				  

				 
				0.490
				 

				  

				 
				0.240
				 

				  

				 
				0.236
				 

				  

				 
				3.570
				 

				  
			 

			 
				  
			 
		 

		  
	 
 
   
 
 
	 
		 
			 
				 
					 
						 
							 
							ANOVA
							 

							  

							  
						 
					 
				 
			 

			 
				 
				Model
				 

				 
				 
				 

				 
				Sum of Squares
				 

				 
				df
				 

				 
				Mean Square
				 

				 
				F
				 

				 
				p
				 
			 
		 

		 
			 
				 
				H₁
				 

				  

				 
				Regression
				 

				  

				 
				1294.350
				 

				  

				 
				2
				 

				  

				 
				647.175
				 

				  

				 
				50.766
				 

				  

				 
				< .001
				 

				  

			 

			 
				 
				 
				 

				  

				 
				Residual
				 

				  

				 
				4092.206
				 

				  

				 
				321
				 

				  

				 
				12.748
				 

				  

				  

				  

				 
				 
				 

				  
			 

			 
				 
				 
				 

				  

				 
				Total
				 

				  

				 
				5386.556
				 

				  

				 
				323
				 

				  

				  

				  

				  

				  

				 
				 
				 

				  
			 

			 
				  
			 
		 

		 
			 
				 
					 
					Note.
					 

				 The intercept model is omitted, as no meaningful information can be shown.
				 
			 
		 
	 
 
   
 
 
	 
		 
			 
				 
					 
						 
							 
							Coefficients
							 

							  

							  
						 
					 
				 
			 

			 
				 
					  
				 

				 
					 
					95% CI
					 
				 

				 
					 
					Collinearity Statistics
					 
				 
			 

			 
				 
				Model
				 

				 
				 
				 

				 
				Unstandardized
				 

				 
				Standard Error
				 

				 
				Standardized
				 

				 
				t
				 

				 
				p
				 

				 
				Lower
				 

				 
				Upper
				 

				 
				Tolerance
				 

				 
				VIF
				 
			 
		 

		 
			 
				 
				H₀
				 

				  

				 
				(Intercept)
				 

				  

				 
				0.004
				 

				  

				 
				0.227
				 

				  

				  

				  

				 
				0.016
				 

				  

				 
				0.987
				 

				  

				 
				-0.443
				 

				  

				 
				0.450
				 

				  

				 
				 
				 

				  

				  

				  

			 

			 
				 
				H₁
				 

				  

				 
				(Intercept)
				 

				  

				 
				-0.010
				 

				  

				 
				0.198
				 

				  

				  

				  

				 
				-0.052
				 

				  

				 
				0.959
				 

				  

				 
				-0.400
				 

				  

				 
				0.380
				 

				  

				 
				 
				 

				  

				  

				  
			 

			 
				 
				 
				 

				  

				 
				PSS
				 

				  

				 
				0.170
				 

				  

				 
				0.035
				 

				  

				 
				0.250
				 

				  

				 
				4.844
				 

				  

				 
				< .001
				 

				  

				 
				0.101
				 

				  

				 
				0.239
				 

				  

				 
				0.885
				 

				  

				 
				1.130
				 

				  
			 

			 
				 
				 
				 

				  

				 
				RSQ
				 

				  

				 
				0.193
				 

				  

				 
				0.029
				 

				  

				 
				0.345
				 

				  

				 
				6.670
				 

				  

				 
				< .001
				 

				  

				 
				0.136
				 

				  

				 
				0.250
				 

				  

				 
				0.885
				 

				  

				 
				1.130
				 

				  
			 

			 
				  
			 
		 

		  
	 
 
   
 
 
	 
		 
			 
				 
					 
						 
							 
							Collinearity Diagnostics
							 

							  

							  
						 
					 
				 
			 

			 
				  

				 
				Variance Proportions
				 
			 

			 
				 
				Model
				 

				 
				Dimension
				 

				 
				Eigenvalue
				 

				 
				Condition Index
				 

				 
				(Intercept)
				 

				 
				PSS
				 

				 
				RSQ
				 
			 
		 

		 
			 
				 
				H₁
				 

				  

				 
				1
				 

				  

				 
				1.339
				 

				  

				 
				1.000
				 

				  

				 
				0.000
				 

				  

				 
				0.330
				 

				  

				 
				0.330
				 

				  

			 

			 
				 
				 
				 

				  

				 
				2
				 

				  

				 
				1.000
				 

				  

				 
				1.157
				 

				  

				 
				1.000
				 

				  

				 
				0.000
				 

				  

				 
				0.000
				 

				  
			 

			 
				 
				 
				 

				  

				 
				3
				 

				  

				 
				0.661
				 

				  

				 
				1.423
				 

				  

				 
				0.000
				 

				  

				 
				0.669
				 

				  

				 
				0.670
				 

				  
			 

			 
				  
			 
		 

		 
			 
				 
					 
					Note.
					 

				 The intercept model is omitted, as no meaningful information can be shown.
				 
			 
		 
	 
 
       
 
  RSQ and PSS as predictors of AV. Bayesian inference   
 
   
 
 
	 
		 
			 
				 
					 
						 
							 
							Model Comparison - AV
							 

							  

							  
						 
					 
				 
			 

			 
				 
				Models
				 

				 
				P(M)
				 

				 
				P(M|data)
				 

				 
				BF

					 
					M
					 
				 

				 
				BF

					 
					10
					 
				 

				 
				R²
				 
			 
		 

		 
			 
				 
				Null model
				 

				  

				 
				0.333
				 

				  

				 
				1.388e -17
				 

				  

				 
				2.776e -17
				 

				  

				 
				1.000
				 

				  

				 
				0.000
				 

				  

			 

			 
				 
				PSS + RSQ
				 

				  

				 
				0.333
				 

				  

				 
				1.000
				 

				  

				 
				29846.100
				 

				  

				 
				7.205e +16
				 

				  

				 
				0.240
				 

				  
			 

			 
				 
				RSQ
				 

				  

				 
				0.167
				 

				  

				 
				6.700e 


				-5
				 

				  

				 
				3.350e 


				-4
				 

				  

				 
				9.656e +12
				 

				  

				 
				0.185
				 

				  
			 

			 
				 
				PSS
				 

				  

				 
				0.167
				 

				  

				 
				5.840e 


				-9
				 

				  

				 
				2.920e 


				-8
				 

				  

				 
				8.417e 


				+8
				 

				  

				 
				0.135
				 

				  
			 

			 
				  
			 
		 

		  
	 
 
   
 
  Posterior Summary   
 
 
	 
		 
			 
				 
					 
						 
							 
							Posterior Summaries of Coefficients
							 

							  

							  
						 
					 
				 
			 

			 
				  

				 
				95% Credible Interval
				 
			 

			 
				 
				Coefficient
				 

				 
				Mean
				 

				 
				SD
				 

				 
				P(incl)
				 

				 
				P(incl|data)
				 

				 
				BF

					 
					inclusion
					 
				 

				 
				Lower
				 

				 
				Upper
				 
			 
		 

		 
			 
				 
				Intercept
				 

				  

				 
				0.004
				 

				  

				 
				0.198
				 

				  

				 
				1.000
				 

				  

				 
				1.000
				 

				  

				 
				1.000
				 

				  

				 
				-0.356
				 

				  

				 
				0.405
				 

				  

			 

			 
				 
				PSS
				 

				  

				 
				0.166
				 

				  

				 
				0.035
				 

				  

				 
				0.500
				 

				  

				 
				1.000
				 

				  

				 
				14924.351
				 

				  

				 
				0.103
				 

				  

				 
				0.237
				 

				  
			 

			 
				 
				RSQ
				 

				  

				 
				0.189
				 

				  

				 
				0.029
				 

				  

				 
				0.500
				 

				  

				 
				1.000
				 

				  

				 
				1.712e +8
				 

				  

				 
				0.137
				 

				  

				 
				0.247
				 

				  
			 

			 
				  
			 
		 

		  
	 
 
         
 
  RSQ and PSS as predictors of AP   
 
   
 
 
	 
		 
			 
				 
					 
						 
							 
							Model Summary - AP
							 

							  

							  
						 
					 
				 
			 

			 
				 
				Model
				 

				 
				R
				 

				 
				R²
				 

				 
				Adjusted R²
				 

				 
				RMSE
				 
			 
		 

		 
			 
				 
				H₀
				 

				  

				 
				0.000
				 

				  

				 
				0.000
				 

				  

				 
				0.000
				 

				  

				 
				5.845
				 

				  

			 

			 
				 
				H₁
				 

				  

				 
				0.103
				 

				  

				 
				0.011
				 

				  

				 
				0.005
				 

				  

				 
				5.831
				 

				  
			 

			 
				  
			 
		 

		  
	 
 
   
 
 
	 
		 
			 
				 
					 
						 
							 
							ANOVA
							 

							  

							  
						 
					 
				 
			 

			 
				 
				Model
				 

				 
				 
				 

				 
				Sum of Squares
				 

				 
				df
				 

				 
				Mean Square
				 

				 
				F
				 

				 
				p
				 
			 
		 

		 
			 
				 
				H₁
				 

				  

				 
				Regression
				 

				  

				 
				118.189
				 

				  

				 
				2
				 

				  

				 
				59.094
				 

				  

				 
				1.738
				 

				  

				 
				0.178
				 

				  

			 

			 
				 
				 
				 

				  

				 
				Residual
				 

				  

				 
				10915.836
				 

				  

				 
				321
				 

				  

				 
				34.006
				 

				  

				  

				  

				 
				 
				 

				  
			 

			 
				 
				 
				 

				  

				 
				Total
				 

				  

				 
				11034.025
				 

				  

				 
				323
				 

				  

				  

				  

				  

				  

				 
				 
				 

				  
			 

			 
				  
			 
		 

		 
			 
				 
					 
					Note.
					 

				 The intercept model is omitted, as no meaningful information can be shown.
				 
			 
		 
	 
 
   
 
 
	 
		 
			 
				 
					 
						 
							 
							Coefficients
							 

							  

							  
						 
					 
				 
			 

			 
				  

				 
				95% CI
				 
			 

			 
				 
				Model
				 

				 
				 
				 

				 
				Unstandardized
				 

				 
				Standard Error
				 

				 
				Standardized
				 

				 
				t
				 

				 
				p
				 

				 
				Lower
				 

				 
				Upper
				 
			 
		 

		 
			 
				 
				H₀
				 

				  

				 
				(Intercept)
				 

				  

				 
				0.004
				 

				  

				 
				0.325
				 

				  

				  

				  

				 
				0.013
				 

				  

				 
				0.990
				 

				  

				 
				-0.635
				 

				  

				 
				0.643
				 

				  

			 

			 
				 
				H₁
				 

				  

				 
				(Intercept)
				 

				  

				 
				0.002
				 

				  

				 
				0.324
				 

				  

				  

				  

				 
				0.006
				 

				  

				 
				0.995
				 

				  

				 
				-0.636
				 

				  

				 
				0.639
				 

				  
			 

			 
				 
				 
				 

				  

				 
				PSS
				 

				  

				 
				-0.061
				 

				  

				 
				0.057
				 

				  

				 
				-0.063
				 

				  

				 
				-1.072
				 

				  

				 
				0.284
				 

				  

				 
				-0.174
				 

				  

				 
				0.051
				 

				  
			 

			 
				 
				 
				 

				  

				 
				RSQ
				 

				  

				 
				0.085
				 

				  

				 
				0.047
				 

				  

				 
				0.106
				 

				  

				 
				1.798
				 

				  

				 
				0.073
				 

				  

				 
				-0.008
				 

				  

				 
				0.178
				 

				  
			 

			 
				  
			 
		 

		  
	 
 
       
 
  RSQ and PSS as predictors of AP. Bayesian inference   
 
   
 
 
	 
		 
			 
				 
					 
						 
							 
							Model Comparison - AP
							 

							  

							  
						 
					 
				 
			 

			 
				 
				Models
				 

				 
				P(M)
				 

				 
				P(M|data)
				 

				 
				BF

					 
					M
					 
				 

				 
				BF

					 
					10
					 
				 

				 
				R²
				 
			 
		 

		 
			 
				 
				Null model
				 

				  

				 
				0.333
				 

				  

				 
				0.729
				 

				  

				 
				5.375
				 

				  

				 
				1.000
				 

				  

				 
				0.000
				 

				  

			 

			 
				 
				RSQ
				 

				  

				 
				0.167
				 

				  

				 
				0.135
				 

				  

				 
				0.784
				 

				  

				 
				0.372
				 

				  

				 
				0.007
				 

				  
			 

			 
				 
				PSS + RSQ
				 

				  

				 
				0.333
				 

				  

				 
				0.086
				 

				  

				 
				0.187
				 

				  

				 
				0.118
				 

				  

				 
				0.011
				 

				  
			 

			 
				 
				PSS
				 

				  

				 
				0.167
				 

				  

				 
				0.050
				 

				  

				 
				0.263
				 

				  

				 
				0.137
				 

				  

				 
				0.001
				 

				  
			 

			 
				  
			 
		 

		  
	 
 
   
 
  Posterior Summary   
 
 
	 
		 
			 
				 
					 
						 
							 
							Posterior Summaries of Coefficients
							 

							  

							  
						 
					 
				 
			 

			 
				  

				 
				95% Credible Interval
				 
			 

			 
				 
				Coefficient
				 

				 
				Mean
				 

				 
				SD
				 

				 
				P(incl)
				 

				 
				P(incl|data)
				 

				 
				BF

					 
					inclusion
					 
				 

				 
				Lower
				 

				 
				Upper
				 
			 
		 

		 
			 
				 
				Intercept
				 

				  

				 
				0.004
				 

				  

				 
				0.325
				 

				  

				 
				1.000
				 

				  

				 
				1.000
				 

				  

				 
				1.000
				 

				  

				 
				-0.626
				 

				  

				 
				0.578
				 

				  

			 

			 
				 
				PSS
				 

				  

				 
				-0.006
				 

				  

				 
				0.026
				 

				  

				 
				0.500
				 

				  

				 
				0.136
				 

				  

				 
				0.157
				 

				  

				 
				-0.108
				 

				  

				 
				0.003
				 

				  
			 

			 
				 
				RSQ
				 

				  

				 
				0.016
				 

				  

				 
				0.036
				 

				  

				 
				0.500
				 

				  

				 
				0.221
				 

				  

				 
				0.284
				 

				  

				 
				-0.003
				 

				  

				 
				0.116
				 

				  
			 

			 
				  
			 
		 

		  
	 
 
   
 
  Posterior Coefficients with 95% Credible Interval         
 
  Residuals vs Fitted       
 
  Inclusion Probabilities       
 
  Q-Q Plot       
 
  Marginal Posterior Distributions   
 
  Intercept       
 
  PSS       
 
  RSQ             
 
  RSQ and PSS as predictors of LOT   
 
   
 
 
	 
		 
			 
				 
					 
						 
							 
							Model Summary - LOT
							 

							  

							  
						 
					 
				 
			 

			 
				 
				Model
				 

				 
				R
				 

				 
				R²
				 

				 
				Adjusted R²
				 

				 
				RMSE
				 
			 
		 

		 
			 
				 
				H₀
				 

				  

				 
				0.000
				 

				  

				 
				0.000
				 

				  

				 
				0.000
				 

				  

				 
				4.931
				 

				  

			 

			 
				 
				H₁
				 

				  

				 
				0.492
				 

				  

				 
				0.242
				 

				  

				 
				0.237
				 

				  

				 
				4.307
				 

				  
			 

			 
				  
			 
		 

		  
	 
 
   
 
 
	 
		 
			 
				 
					 
						 
							 
							ANOVA
							 

							  

							  
						 
					 
				 
			 

			 
				 
				Model
				 

				 
				 
				 

				 
				Sum of Squares
				 

				 
				df
				 

				 
				Mean Square
				 

				 
				F
				 

				 
				p
				 
			 
		 

		 
			 
				 
				H₁
				 

				  

				 
				Regression
				 

				  

				 
				1903.710
				 

				  

				 
				2
				 

				  

				 
				951.855
				 

				  

				 
				51.310
				 

				  

				 
				< .001
				 

				  

			 

			 
				 
				 
				 

				  

				 
				Residual
				 

				  

				 
				5973.478
				 

				  

				 
				322
				 

				  

				 
				18.551
				 

				  

				  

				  

				 
				 
				 

				  
			 

			 
				 
				 
				 

				  

				 
				Total
				 

				  

				 
				7877.188
				 

				  

				 
				324
				 

				  

				  

				  

				  

				  

				 
				 
				 

				  
			 

			 
				  
			 
		 

		 
			 
				 
					 
					Note.
					 

				 The intercept model is omitted, as no meaningful information can be shown.
				 
			 
		 
	 
 
   
 
 
	 
		 
			 
				 
					 
						 
							 
							Coefficients
							 

							  

							  
						 
					 
				 
			 

			 
				 
					  
				 

				 
					 
					95% CI
					 
				 

				 
					 
					Collinearity Statistics
					 
				 
			 

			 
				 
				Model
				 

				 
				 
				 

				 
				Unstandardized
				 

				 
				Standard Error
				 

				 
				Standardized
				 

				 
				t
				 

				 
				p
				 

				 
				Lower
				 

				 
				Upper
				 

				 
				Tolerance
				 

				 
				VIF
				 
			 
		 

		 
			 
				 
				H₀
				 

				  

				 
				(Intercept)
				 

				  

				 
				0.006
				 

				  

				 
				0.274
				 

				  

				  

				  

				 
				0.022
				 

				  

				 
				0.982
				 

				  

				 
				-0.532
				 

				  

				 
				0.544
				 

				  

				 
				 
				 

				  

				  

				  

			 

			 
				 
				H₁
				 

				  

				 
				(Intercept)
				 

				  

				 
				0.008
				 

				  

				 
				0.239
				 

				  

				  

				  

				 
				0.033
				 

				  

				 
				0.974
				 

				  

				 
				-0.462
				 

				  

				 
				0.478
				 

				  

				 
				 
				 

				  

				  

				  
			 

			 
				 
				 
				 

				  

				 
				RSQ
				 

				  

				 
				-0.133
				 

				  

				 
				0.035
				 

				  

				 
				-0.198
				 

				  

				 
				-3.825
				 

				  

				 
				< .001
				 

				  

				 
				-0.202
				 

				  

				 
				-0.065
				 

				  

				 
				0.882
				 

				  

				 
				1.134
				 

				  
			 

			 
				 
				 
				 

				  

				 
				PSS
				 

				  

				 
				-0.317
				 

				  

				 
				0.042
				 

				  

				 
				-0.387
				 

				  

				 
				-7.492
				 

				  

				 
				< .001
				 

				  

				 
				-0.400
				 

				  

				 
				-0.233
				 

				  

				 
				0.882
				 

				  

				 
				1.134
				 

				  
			 

			 
				  
			 
		 

		  
	 
 
   
 
 
	 
		 
			 
				 
					 
						 
							 
							Collinearity Diagnostics
							 

							  

							  
						 
					 
				 
			 

			 
				  

				 
				Variance Proportions
				 
			 

			 
				 
				Model
				 

				 
				Dimension
				 

				 
				Eigenvalue
				 

				 
				Condition Index
				 

				 
				(Intercept)
				 

				 
				RSQ
				 

				 
				PSS
				 
			 
		 

		 
			 
				 
				H₁
				 

				  

				 
				1
				 

				  

				 
				1.344
				 

				  

				 
				1.000
				 

				  

				 
				0.000
				 

				  

				 
				0.328
				 

				  

				 
				0.328
				 

				  

			 

			 
				 
				 
				 

				  

				 
				2
				 

				  

				 
				1.000
				 

				  

				 
				1.159
				 

				  

				 
				1.000
				 

				  

				 
				0.000
				 

				  

				 
				0.000
				 

				  
			 

			 
				 
				 
				 

				  

				 
				3
				 

				  

				 
				0.656
				 

				  

				 
				1.431
				 

				  

				 
				0.000
				 

				  

				 
				0.672
				 

				  

				 
				0.672
				 

				  
			 

			 
				  
			 
		 

		 
			 
				 
					 
					Note.
					 

				 The intercept model is omitted, as no meaningful information can be shown.
				 
			 
		 
	 
 
       
 
  RSQ and PSS as predictors of LOT. Bayesian inference   
 
   
 
 
	 
		 
			 
				 
					 
						 
							 
							Model Comparison - LOT
							 

							  

							  
						 
					 
				 
			 

			 
				 
				Models
				 

				 
				P(M)
				 

				 
				P(M|data)
				 

				 
				BF

					 
					M
					 
				 

				 
				BF

					 
					10
					 
				 

				 
				R²
				 
			 
		 

		 
			 
				 
				Null model
				 

				  

				 
				0.333
				 

				  

				 
				9.073e -18
				 

				  

				 
				1.815e -17
				 

				  

				 
				1.000
				 

				  

				 
				0.000
				 

				  

			 

			 
				 
				PSS + RSQ
				 

				  

				 
				0.333
				 

				  

				 
				0.996
				 

				  

				 
				488.775
				 

				  

				 
				1.098e +17
				 

				  

				 
				0.242
				 

				  
			 

			 
				 
				PSS
				 

				  

				 
				0.167
				 

				  

				 
				0.004
				 

				  

				 
				0.020
				 

				  

				 
				8.983e +14
				 

				  

				 
				0.207
				 

				  
			 

			 
				 
				RSQ
				 

				  

				 
				0.167
				 

				  

				 
				4.175e -11
				 

				  

				 
				2.088e -10
				 

				  

				 
				9.203e 


				+6
				 

				  

				 
				0.109
				 

				  
			 

			 
				  
			 
		 

		  
	 
 
   
 
  Posterior Summary   
 
 
	 
		 
			 
				 
					 
						 
							 
							Posterior Summaries of Coefficients
							 

							  

							  
						 
					 
				 
			 

			 
				  

				 
				95% Credible Interval
				 
			 

			 
				 
				Coefficient
				 

				 
				Mean
				 

				 
				SD
				 

				 
				P(incl)
				 

				 
				P(incl|data)
				 

				 
				BF

					 
					inclusion
					 
				 

				 
				Lower
				 

				 
				Upper
				 
			 
		 

		 
			 
				 
				Intercept
				 

				  

				 
				0.006
				 

				  

				 
				0.239
				 

				  

				 
				1.000
				 

				  

				 
				1.000
				 

				  

				 
				1.000
				 

				  

				 
				-0.473
				 

				  

				 
				0.441
				 

				  

			 

			 
				 
				PSS
				 

				  

				 
				-0.310
				 

				  

				 
				0.042
				 

				  

				 
				0.500
				 

				  

				 
				1.000
				 

				  

				 
				2.395e +10
				 

				  

				 
				-0.395
				 

				  

				 
				-0.233
				 

				  
			 

			 
				 
				RSQ
				 

				  

				 
				-0.130
				 

				  

				 
				0.035
				 

				  

				 
				0.500
				 

				  

				 
				0.996
				 

				  

				 
				244.387
				 

				  

				 
				-0.198
				 

				  

				 
				-0.062
				 

				  
			 

			 
				  
			 
		 

		  
	 
 
   
 
  Posterior Coefficients with 95% Credible Interval         
 
  Residuals vs Fitted         Plotting not possible: subscript out of bounds      
 
  Inclusion Probabilities       
 
  Q-Q Plot       
 
  Marginal Posterior Distributions   
 
  Intercept       
 
  PSS       
 
  RSQ           
 
 
	 
		 
			 
				 
					 
						 
							 
							Descriptives
							 

							  

							  
						 
					 
				 
			 

			 
				 
				 
				 

				 
				N
				 

				 
				Mean
				 

				 
				SD
				 
			 
		 

		 
			 
				 
				LOT
				 

				  

				 
				325
				 

				  

				 
				0.006
				 

				  

				 
				4.931
				 

				  

			 

			 
				 
				PSS
				 

				  

				 
				325
				 

				  

				 
				0.002
				 

				  

				 
				6.030
				 

				  
			 

			 
				 
				RSQ
				 

				  

				 
				325
				 

				  

				 
				0.007
				 

				  

				 
				7.307
				 

				  
			 

			 
				  
			 
		 

		  
	 
 
     
 
  RSQ and PSS as predictors of SC   
 
   
 
 
	 
		 
			 
				 
					 
						 
							 
							Model Summary - SC
							 

							  

							  
						 
					 
				 
			 

			 
				 
				Model
				 

				 
				R
				 

				 
				R²
				 

				 
				Adjusted R²
				 

				 
				RMSE
				 
			 
		 

		 
			 
				 
				H₀
				 

				  

				 
				0.000
				 

				  

				 
				0.000
				 

				  

				 
				0.000
				 

				  

				 
				8.891
				 

				  

			 

			 
				 
				H₁
				 

				  

				 
				0.539
				 

				  

				 
				0.291
				 

				  

				 
				0.287
				 

				  

				 
				7.510
				 

				  
			 

			 
				  
			 
		 

		  
	 
 
   
 
 
	 
		 
			 
				 
					 
						 
							 
							ANOVA
							 

							  

							  
						 
					 
				 
			 

			 
				 
				Model
				 

				 
				 
				 

				 
				Sum of Squares
				 

				 
				df
				 

				 
				Mean Square
				 

				 
				F
				 

				 
				p
				 
			 
		 

		 
			 
				 
				H₁
				 

				  

				 
				Regression
				 

				  

				 
				7453.696
				 

				  

				 
				2
				 

				  

				 
				3726.848
				 

				  

				 
				66.082
				 

				  

				 
				< .001
				 

				  

			 

			 
				 
				 
				 

				  

				 
				Residual
				 

				  

				 
				18159.892
				 

				  

				 
				322
				 

				  

				 
				56.397
				 

				  

				  

				  

				 
				 
				 

				  
			 

			 
				 
				 
				 

				  

				 
				Total
				 

				  

				 
				25613.588
				 

				  

				 
				324
				 

				  

				  

				  

				  

				  

				 
				 
				 

				  
			 

			 
				  
			 
		 

		 
			 
				 
					 
					Note.
					 

				 The intercept model is omitted, as no meaningful information can be shown.
				 
			 
		 
	 
 
   
 
 
	 
		 
			 
				 
					 
						 
							 
							Coefficients
							 

							  

							  
						 
					 
				 
			 

			 
				 
					  
				 

				 
					 
					95% CI
					 
				 

				 
					 
					Collinearity Statistics
					 
				 
			 

			 
				 
				Model
				 

				 
				 
				 

				 
				Unstandardized
				 

				 
				Standard Error
				 

				 
				Standardized
				 

				 
				t
				 

				 
				p
				 

				 
				Lower
				 

				 
				Upper
				 

				 
				Tolerance
				 

				 
				VIF
				 
			 
		 

		 
			 
				 
				H₀
				 

				  

				 
				(Intercept)
				 

				  

				 
				0.006
				 

				  

				 
				0.493
				 

				  

				  

				  

				 
				0.012
				 

				  

				 
				0.990
				 

				  

				 
				-0.964
				 

				  

				 
				0.976
				 

				  

				 
				 
				 

				  

				  

				  

			 

			 
				 
				H₁
				 

				  

				 
				(Intercept)
				 

				  

				 
				0.009
				 

				  

				 
				0.417
				 

				  

				  

				  

				 
				0.021
				 

				  

				 
				0.983
				 

				  

				 
				-0.811
				 

				  

				 
				0.828
				 

				  

				 
				 
				 

				  

				  

				  
			 

			 
				 
				 
				 

				  

				 
				PSS
				 

				  

				 
				-0.716
				 

				  

				 
				0.074
				 

				  

				 
				-0.486
				 

				  

				 
				-9.723
				 

				  

				 
				< .001
				 

				  

				 
				-0.861
				 

				  

				 
				-0.571
				 

				  

				 
				0.882
				 

				  

				 
				1.134
				 

				  
			 

			 
				 
				 
				 

				  

				 
				RSQ
				 

				  

				 
				-0.147
				 

				  

				 
				0.061
				 

				  

				 
				-0.121
				 

				  

				 
				-2.414
				 

				  

				 
				0.016
				 

				  

				 
				-0.266
				 

				  

				 
				-0.027
				 

				  

				 
				0.882
				 

				  

				 
				1.134
				 

				  
			 

			 
				  
			 
		 

		  
	 
 
   
 
 
	 
		 
			 
				 
					 
						 
							 
							Collinearity Diagnostics
							 

							  

							  
						 
					 
				 
			 

			 
				  

				 
				Variance Proportions
				 
			 

			 
				 
				Model
				 

				 
				Dimension
				 

				 
				Eigenvalue
				 

				 
				Condition Index
				 

				 
				(Intercept)
				 

				 
				PSS
				 

				 
				RSQ
				 
			 
		 

		 
			 
				 
				H₁
				 

				  

				 
				1
				 

				  

				 
				1.344
				 

				  

				 
				1.000
				 

				  

				 
				0.000
				 

				  

				 
				0.328
				 

				  

				 
				0.328
				 

				  

			 

			 
				 
				 
				 

				  

				 
				2
				 

				  

				 
				1.000
				 

				  

				 
				1.159
				 

				  

				 
				1.000
				 

				  

				 
				0.000
				 

				  

				 
				0.000
				 

				  
			 

			 
				 
				 
				 

				  

				 
				3
				 

				  

				 
				0.656
				 

				  

				 
				1.431
				 

				  

				 
				0.000
				 

				  

				 
				0.672
				 

				  

				 
				0.672
				 

				  
			 

			 
				  
			 
		 

		 
			 
				 
					 
					Note.
					 

				 The intercept model is omitted, as no meaningful information can be shown.
				 
			 
		 
	 
 
   
 
 
	 
		 
			 
				 
					 
						 
							 
							Residuals Statistics
							 

							  

							  
						 
					 
				 
			 

			 
				 
				 
				 

				 
				Minimum
				 

				 
				Maximum
				 

				 
				Mean
				 

				 
				SD
				 

				 
				N
				 
			 
		 

		 
			 
				 
				Predicted Value
				 

				  

				 
				-16.235
				 

				  

				 
				10.344
				 

				  

				 
				0.006
				 

				  

				 
				4.796
				 

				  

				 
				325
				 

				  

			 

			 
				 
				Residual
				 

				  

				 
				-24.610
				 

				  

				 
				20.012
				 

				  

				 
				6.685e -17
				 

				  

				 
				7.487
				 

				  

				 
				325
				 

				  
			 

			 
				 
				Std. Predicted Value
				 

				  

				 
				-3.386
				 

				  

				 
				2.155
				 

				  

				 
				1.186e -18
				 

				  

				 
				1.000
				 

				  

				 
				325
				 

				  
			 

			 
				 
				Std. Residual
				 

				  

				 
				-3.289
				 

				  

				 
				2.684
				 

				  

				 
				3.002e 


				-4
				 

				  

				 
				1.002
				 

				  

				 
				325
				 

				  
			 

			 
				  
			 
		 

		  
	 
 
   
 
  Residuals vs. Predicted       
 
  Q-Q Plot Standardized Residuals           
 
  RSQ and PSS as predictors of SC. Bayesian inference   
 
   
 
 
	 
		 
			 
				 
					 
						 
							 
							Model Comparison - SC
							 

							  

							  
						 
					 
				 
			 

			 
				 
				Models
				 

				 
				P(M)
				 

				 
				P(M|data)
				 

				 
				BF

					 
					M
					 
				 

				 
				BF

					 
					10
					 
				 

				 
				R²
				 
			 
		 

		 
			 
				 
				Null model
				 

				  

				 
				0.333
				 

				  

				 
				1.719e -22
				 

				  

				 
				3.438e -22
				 

				  

				 
				1.000
				 

				  

				 
				0.000
				 

				  

			 

			 
				 
				PSS + RSQ
				 

				  

				 
				0.333
				 

				  

				 
				0.769
				 

				  

				 
				6.673
				 

				  

				 
				4.476e +21
				 

				  

				 
				0.291
				 

				  
			 

			 
				 
				PSS
				 

				  

				 
				0.167
				 

				  

				 
				0.231
				 

				  

				 
				1.499
				 

				  

				 
				2.683e +21
				 

				  

				 
				0.278
				 

				  
			 

			 
				 
				RSQ
				 

				  

				 
				0.167
				 

				  

				 
				7.649e -18
				 

				  

				 
				3.824e -17
				 

				  

				 
				88983.577
				 

				  

				 
				0.083
				 

				  
			 

			 
				  
			 
		 

		  
	 
 
   
 
  Posterior Summary   
 
 
	 
		 
			 
				 
					 
						 
							 
							Posterior Summaries of Coefficients
							 

							  

							  
						 
					 
				 
			 

			 
				  

				 
				95% Credible Interval
				 
			 

			 
				 
				Coefficient
				 

				 
				Mean
				 

				 
				SD
				 

				 
				P(incl)
				 

				 
				P(incl|data)
				 

				 
				BF

					 
					inclusion
					 
				 

				 
				Lower
				 

				 
				Upper
				 
			 
		 

		 
			 
				 
				Intercept
				 

				  

				 
				0.006
				 

				  

				 
				0.417
				 

				  

				 
				1.000
				 

				  

				 
				1.000
				 

				  

				 
				1.000
				 

				  

				 
				-0.842
				 

				  

				 
				0.792
				 

				  

			 

			 
				 
				PSS
				 

				  

				 
				-0.719
				 

				  

				 
				0.077
				 

				  

				 
				0.500
				 

				  

				 
				1.000
				 

				  

				 
				1.307e +17
				 

				  

				 
				-0.863
				 

				  

				 
				-0.572
				 

				  
			 

			 
				 
				RSQ
				 

				  

				 
				-0.111
				 

				  

				 
				0.081
				 

				  

				 
				0.500
				 

				  

				 
				0.769
				 

				  

				 
				3.337
				 

				  

				 
				-0.249
				 

				  

				 
				0.000
				 

				  
			 

			 
				  
			 
		 

		  
	 
 
   
 
  Posterior Coefficients with 95% Credible Interval         
 
  Inclusion Probabilities       
 
  Q-Q Plot       
 
  Marginal Posterior Distributions   
 
  Intercept       
 
  PSS       
 
  RSQ             
 
  RSQ and PSS as predictors of CS   
 
   
 
 
	 
		 
			 
				 
					 
						 
							 
							Model Summary - CS
							 

							  

							  
						 
					 
				 
			 

			 
				 
				Model
				 

				 
				R
				 

				 
				R²
				 

				 
				Adjusted R²
				 

				 
				RMSE
				 
			 
		 

		 
			 
				 
				H₀
				 

				  

				 
				0.000
				 

				  

				 
				0.000
				 

				  

				 
				0.000
				 

				  

				 
				5.033
				 

				  

			 

			 
				 
				H₁
				 

				  

				 
				0.358
				 

				  

				 
				0.128
				 

				  

				 
				0.123
				 

				  

				 
				4.714
				 

				  
			 

			 
				  
			 
		 

		  
	 
 
   
 
 
	 
		 
			 
				 
					 
						 
							 
							ANOVA
							 

							  

							  
						 
					 
				 
			 

			 
				 
				Model
				 

				 
				 
				 

				 
				Sum of Squares
				 

				 
				df
				 

				 
				Mean Square
				 

				 
				F
				 

				 
				p
				 
			 
		 

		 
			 
				 
				H₁
				 

				  

				 
				Regression
				 

				  

				 
				1051.307
				 

				  

				 
				2
				 

				  

				 
				525.654
				 

				  

				 
				23.653
				 

				  

				 
				< .001
				 

				  

			 

			 
				 
				 
				 

				  

				 
				Residual
				 

				  

				 
				7156.096
				 

				  

				 
				322
				 

				  

				 
				22.224
				 

				  

				  

				  

				 
				 
				 

				  
			 

			 
				 
				 
				 

				  

				 
				Total
				 

				  

				 
				8207.403
				 

				  

				 
				324
				 

				  

				  

				  

				  

				  

				 
				 
				 

				  
			 

			 
				  
			 
		 

		 
			 
				 
					 
					Note.
					 

				 The intercept model is omitted, as no meaningful information can be shown.
				 
			 
		 
	 
 
   
 
 
	 
		 
			 
				 
					 
						 
							 
							Coefficients
							 

							  

							  
						 
					 
				 
			 

			 
				 
					  
				 

				 
					 
					95% CI
					 
				 

				 
					 
					Collinearity Statistics
					 
				 
			 

			 
				 
				Model
				 

				 
				 
				 

				 
				Unstandardized
				 

				 
				Standard Error
				 

				 
				Standardized
				 

				 
				t
				 

				 
				p
				 

				 
				Lower
				 

				 
				Upper
				 

				 
				Tolerance
				 

				 
				VIF
				 
			 
		 

		 
			 
				 
				H₀
				 

				  

				 
				(Intercept)
				 

				  

				 
				0.005
				 

				  

				 
				0.279
				 

				  

				  

				  

				 
				0.019
				 

				  

				 
				0.985
				 

				  

				 
				-0.544
				 

				  

				 
				0.555
				 

				  

				 
				 
				 

				  

				  

				  

			 

			 
				 
				H₁
				 

				  

				 
				(Intercept)
				 

				  

				 
				0.006
				 

				  

				 
				0.261
				 

				  

				  

				  

				 
				0.024
				 

				  

				 
				0.981
				 

				  

				 
				-0.508
				 

				  

				 
				0.521
				 

				  

				 
				 
				 

				  

				  

				  
			 

			 
				 
				 
				 

				  

				 
				PSS
				 

				  

				 
				-0.281
				 

				  

				 
				0.046
				 

				  

				 
				-0.337
				 

				  

				 
				-6.076
				 

				  

				 
				< .001
				 

				  

				 
				-0.372
				 

				  

				 
				-0.190
				 

				  

				 
				0.882
				 

				  

				 
				1.134
				 

				  
			 

			 
				 
				 
				 

				  

				 
				RSQ
				 

				  

				 
				-0.036
				 

				  

				 
				0.038
				 

				  

				 
				-0.052
				 

				  

				 
				-0.935
				 

				  

				 
				0.350
				 

				  

				 
				-0.111
				 

				  

				 
				0.039
				 

				  

				 
				0.882
				 

				  

				 
				1.134
				 

				  
			 

			 
				  
			 
		 

		  
	 
 
   
 
 
	 
		 
			 
				 
					 
						 
							 
							Collinearity Diagnostics
							 

							  

							  
						 
					 
				 
			 

			 
				  

				 
				Variance Proportions
				 
			 

			 
				 
				Model
				 

				 
				Dimension
				 

				 
				Eigenvalue
				 

				 
				Condition Index
				 

				 
				(Intercept)
				 

				 
				PSS
				 

				 
				RSQ
				 
			 
		 

		 
			 
				 
				H₁
				 

				  

				 
				1
				 

				  

				 
				1.344
				 

				  

				 
				1.000
				 

				  

				 
				0.000
				 

				  

				 
				0.328
				 

				  

				 
				0.328
				 

				  

			 

			 
				 
				 
				 

				  

				 
				2
				 

				  

				 
				1.000
				 

				  

				 
				1.159
				 

				  

				 
				1.000
				 

				  

				 
				0.000
				 

				  

				 
				0.000
				 

				  
			 

			 
				 
				 
				 

				  

				 
				3
				 

				  

				 
				0.656
				 

				  

				 
				1.431
				 

				  

				 
				0.000
				 

				  

				 
				0.672
				 

				  

				 
				0.672
				 

				  
			 

			 
				  
			 
		 

		 
			 
				 
					 
					Note.
					 

				 The intercept model is omitted, as no meaningful information can be shown.
				 
			 
		 
	 
 
   
 
 
	 
		 
			 
				 
					 
						 
							 
							Residuals Statistics
							 

							  

							  
						 
					 
				 
			 

			 
				 
				 
				 

				 
				Minimum
				 

				 
				Maximum
				 

				 
				Mean
				 

				 
				SD
				 

				 
				N
				 
			 
		 

		 
			 
				 
				Predicted Value
				 

				  

				 
				-6.259
				 

				  

				 
				3.730
				 

				  

				 
				0.005
				 

				  

				 
				1.801
				 

				  

				 
				325
				 

				  

			 

			 
				 
				Residual
				 

				  

				 
				-19.824
				 

				  

				 
				9.722
				 

				  

				 
				-1.175e -16
				 

				  

				 
				4.700
				 

				  

				 
				325
				 

				  
			 

			 
				 
				Std. Predicted Value
				 

				  

				 
				-3.478
				 

				  

				 
				2.068
				 

				  

				 
				-1.519e -17
				 

				  

				 
				1.000
				 

				  

				 
				325
				 

				  
			 

			 
				 
				Std. Residual
				 

				  

				 
				-4.212
				 

				  

				 
				2.087
				 

				  

				 
				3.106e 


				-4
				 

				  

				 
				1.002
				 

				  

				 
				325
				 

				  
			 

			 
				  
			 
		 

		  
	 
 
   
 
  Residuals vs. Predicted       
 
  Q-Q Plot Standardized Residuals           
 
  RSQ and PSS as predictors of CS. Bayesian inference   
 
   
 
 
	 
		 
			 
				 
					 
						 
							 
							Model Comparison - CS
							 

							  

							  
						 
					 
				 
			 

			 
				 
				Models
				 

				 
				P(M)
				 

				 
				P(M|data)
				 

				 
				BF

					 
					M
					 
				 

				 
				BF

					 
					10
					 
				 

				 
				R²
				 
			 
		 

		 
			 
				 
				Null model
				 

				  

				 
				0.333
				 

				  

				 
				8.456e -9
				 

				  

				 
				1.691e -8
				 

				  

				 
				1.000
				 

				  

				 
				0.000
				 

				  

			 

			 
				 
				PSS
				 

				  

				 
				0.167
				 

				  

				 
				0.710
				 

				  

				 
				12.225
				 

				  

				 
				1.679e +8
				 

				  

				 
				0.126
				 

				  
			 

			 
				 
				PSS + RSQ
				 

				  

				 
				0.333
				 

				  

				 
				0.290
				 

				  

				 
				0.818
				 

				  

				 
				3.433e +7
				 

				  

				 
				0.128
				 

				  
			 

			 
				 
				RSQ
				 

				  

				 
				0.167
				 

				  

				 
				4.335e -8
				 

				  

				 
				2.168e -7
				 

				  

				 
				10.254
				 

				  

				 
				0.028
				 

				  
			 

			 
				  
			 
		 

		  
	 
 
   
 
  Posterior Summary   
 
 
	 
		 
			 
				 
					 
						 
							 
							Posterior Summaries of Coefficients
							 

							  

							  
						 
					 
				 
			 

			 
				  

				 
				95% Credible Interval
				 
			 

			 
				 
				Coefficient
				 

				 
				Mean
				 

				 
				SD
				 

				 
				P(incl)
				 

				 
				P(incl|data)
				 

				 
				BF

					 
					inclusion
					 
				 

				 
				Lower
				 

				 
				Upper
				 
			 
		 

		 
			 
				 
				Intercept
				 

				  

				 
				0.005
				 

				  

				 
				0.261
				 

				  

				 
				1.000
				 

				  

				 
				1.000
				 

				  

				 
				1.000
				 

				  

				 
				-0.499
				 

				  

				 
				0.488
				 

				  

			 

			 
				 
				PSS
				 

				  

				 
				-0.284
				 

				  

				 
				0.044
				 

				  

				 
				0.500
				 

				  

				 
				1.000
				 

				  

				 
				1.930e +7
				 

				  

				 
				-0.363
				 

				  

				 
				-0.195
				 

				  
			 

			 
				 
				RSQ
				 

				  

				 
				-0.010
				 

				  

				 
				0.026
				 

				  

				 
				0.500
				 

				  

				 
				0.290
				 

				  

				 
				0.409
				 

				  

				 
				-0.080
				 

				  

				 
				0.017
				 

				  
			 

			 
				  
			 
		 

		  
	 
 
   
 
  Posterior Coefficients with 95% Credible Interval         
 
  Inclusion Probabilities       
 
  Q-Q Plot       
 
  Marginal Posterior Distributions   
 
  Intercept       
 
  PSS       
 
  RSQ             
 
  RSQ and PSS as predictors of BU   
 
   
 
 
	 
		 
			 
				 
					 
						 
							 
							Model Summary - BU
							 

							  

							  
						 
					 
				 
			 

			 
				 
				Model
				 

				 
				R
				 

				 
				R²
				 

				 
				Adjusted R²
				 

				 
				RMSE
				 
			 
		 

		 
			 
				 
				H₀
				 

				  

				 
				0.000
				 

				  

				 
				0.000
				 

				  

				 
				0.000
				 

				  

				 
				4.915
				 

				  

			 

			 
				 
				H₁
				 

				  

				 
				0.511
				 

				  

				 
				0.261
				 

				  

				 
				0.257
				 

				  

				 
				4.237
				 

				  
			 

			 
				  
			 
		 

		  
	 
 
   
 
 
	 
		 
			 
				 
					 
						 
							 
							ANOVA
							 

							  

							  
						 
					 
				 
			 

			 
				 
				Model
				 

				 
				 
				 

				 
				Sum of Squares
				 

				 
				df
				 

				 
				Mean Square
				 

				 
				F
				 

				 
				p
				 
			 
		 

		 
			 
				 
				H₁
				 

				  

				 
				Regression
				 

				  

				 
				2044.953
				 

				  

				 
				2
				 

				  

				 
				1022.476
				 

				  

				 
				56.947
				 

				  

				 
				< .001
				 

				  

			 

			 
				 
				 
				 

				  

				 
				Residual
				 

				  

				 
				5781.435
				 

				  

				 
				322
				 

				  

				 
				17.955
				 

				  

				  

				  

				 
				 
				 

				  
			 

			 
				 
				 
				 

				  

				 
				Total
				 

				  

				 
				7826.388
				 

				  

				 
				324
				 

				  

				  

				  

				  

				  

				 
				 
				 

				  
			 

			 
				  
			 
		 

		 
			 
				 
					 
					Note.
					 

				 The intercept model is omitted, as no meaningful information can be shown.
				 
			 
		 
	 
 
   
 
 
	 
		 
			 
				 
					 
						 
							 
							Coefficients
							 

							  

							  
						 
					 
				 
			 

			 
				 
					  
				 

				 
					 
					95% CI
					 
				 

				 
					 
					Collinearity Statistics
					 
				 
			 

			 
				 
				Model
				 

				 
				 
				 

				 
				Unstandardized
				 

				 
				Standard Error
				 

				 
				Standardized
				 

				 
				t
				 

				 
				p
				 

				 
				Lower
				 

				 
				Upper
				 

				 
				Tolerance
				 

				 
				VIF
				 
			 
		 

		 
			 
				 
				H₀
				 

				  

				 
				(Intercept)
				 

				  

				 
				0.004
				 

				  

				 
				0.273
				 

				  

				  

				  

				 
				0.014
				 

				  

				 
				0.989
				 

				  

				 
				-0.532
				 

				  

				 
				0.540
				 

				  

				 
				 
				 

				  

				  

				  

			 

			 
				 
				H₁
				 

				  

				 
				(Intercept)
				 

				  

				 
				0.002
				 

				  

				 
				0.235
				 

				  

				  

				  

				 
				0.008
				 

				  

				 
				0.993
				 

				  

				 
				-0.460
				 

				  

				 
				0.464
				 

				  

				 
				 
				 

				  

				  

				  
			 

			 
				 
				 
				 

				  

				 
				PSS
				 

				  

				 
				0.302
				 

				  

				 
				0.042
				 

				  

				 
				0.370
				 

				  

				 
				7.252
				 

				  

				 
				< .001
				 

				  

				 
				0.220
				 

				  

				 
				0.383
				 

				  

				 
				0.882
				 

				  

				 
				1.134
				 

				  
			 

			 
				 
				 
				 

				  

				 
				RSQ
				 

				  

				 
				0.167
				 

				  

				 
				0.034
				 

				  

				 
				0.248
				 

				  

				 
				4.856
				 

				  

				 
				< .001
				 

				  

				 
				0.099
				 

				  

				 
				0.234
				 

				  

				 
				0.882
				 

				  

				 
				1.134
				 

				  
			 

			 
				  
			 
		 

		  
	 
 
   
 
 
	 
		 
			 
				 
					 
						 
							 
							Collinearity Diagnostics
							 

							  

							  
						 
					 
				 
			 

			 
				  

				 
				Variance Proportions
				 
			 

			 
				 
				Model
				 

				 
				Dimension
				 

				 
				Eigenvalue
				 

				 
				Condition Index
				 

				 
				(Intercept)
				 

				 
				PSS
				 

				 
				RSQ
				 
			 
		 

		 
			 
				 
				H₁
				 

				  

				 
				1
				 

				  

				 
				1.344
				 

				  

				 
				1.000
				 

				  

				 
				0.000
				 

				  

				 
				0.328
				 

				  

				 
				0.328
				 

				  

			 

			 
				 
				 
				 

				  

				 
				2
				 

				  

				 
				1.000
				 

				  

				 
				1.159
				 

				  

				 
				1.000
				 

				  

				 
				0.000
				 

				  

				 
				0.000
				 

				  
			 

			 
				 
				 
				 

				  

				 
				3
				 

				  

				 
				0.656
				 

				  

				 
				1.431
				 

				  

				 
				0.000
				 

				  

				 
				0.672
				 

				  

				 
				0.672
				 

				  
			 

			 
				  
			 
		 

		 
			 
				 
					 
					Note.
					 

				 The intercept model is omitted, as no meaningful information can be shown.
				 
			 
		 
	 
 
   
 
 
	 
		 
			 
				 
					 
						 
							 
							Residuals Statistics
							 

							  

							  
						 
					 
				 
			 

			 
				 
				 
				 

				 
				Minimum
				 

				 
				Maximum
				 

				 
				Mean
				 

				 
				SD
				 

				 
				N
				 
			 
		 

		 
			 
				 
				Predicted Value
				 

				  

				 
				-5.932
				 

				  

				 
				7.351
				 

				  

				 
				0.004
				 

				  

				 
				2.512
				 

				  

				 
				325
				 

				  

			 

			 
				 
				Residual
				 

				  

				 
				-11.561
				 

				  

				 
				14.859
				 

				  

				 
				-7.375e -17
				 

				  

				 
				4.224
				 

				  

				 
				325
				 

				  
			 

			 
				 
				Std. Predicted Value
				 

				  

				 
				-2.363
				 

				  

				 
				2.924
				 

				  

				 
				1.207e -17
				 

				  

				 
				1.000
				 

				  

				 
				325
				 

				  
			 

			 
				 
				Std. Residual
				 

				  

				 
				-2.761
				 

				  

				 
				3.513
				 

				  

				 
				-3.518e 


				-4
				 

				  

				 
				1.002
				 

				  

				 
				325
				 

				  
			 

			 
				  
			 
		 

		  
	 
 
   
 
  Residuals vs. Predicted       
 
  Q-Q Plot Standardized Residuals           
 
  RSQ and PSS as predictors of BU. Bayesian inference   
 
   
 
 
	 
		 
			 
				 
					 
						 
							 
							Model Comparison - BU
							 

							  

							  
						 
					 
				 
			 

			 
				 
				Models
				 

				 
				P(M)
				 

				 
				P(M|data)
				 

				 
				BF

					 
					M
					 
				 

				 
				BF

					 
					10
					 
				 

				 
				R²
				 
			 
		 

		 
			 
				 
				Null model
				 

				  

				 
				0.333
				 

				  

				 
				1.462e -19
				 

				  

				 
				2.924e -19
				 

				  

				 
				1.000
				 

				  

				 
				0.000
				 

				  

			 

			 
				 
				PSS + RSQ
				 

				  

				 
				0.333
				 

				  

				 
				1.000
				 

				  

				 
				30730.660
				 

				  

				 
				6.839e +18
				 

				  

				 
				0.261
				 

				  
			 

			 
				 
				PSS
				 

				  

				 
				0.167
				 

				  

				 
				6.508e 


				-5
				 

				  

				 
				3.254e 


				-4
				 

				  

				 
				8.902e +14
				 

				  

				 
				0.207
				 

				  
			 

			 
				 
				RSQ
				 

				  

				 
				0.167
				 

				  

				 
				1.852e -10
				 

				  

				 
				9.262e -10
				 

				  

				 
				2.534e 


				+9
				 

				  

				 
				0.141
				 

				  
			 

			 
				  
			 
		 

		  
	 
 
   
 
  Posterior Summary   
 
 
	 
		 
			 
				 
					 
						 
							 
							Posterior Summaries of Coefficients
							 

							  

							  
						 
					 
				 
			 

			 
				  

				 
				95% Credible Interval
				 
			 

			 
				 
				Coefficient
				 

				 
				Mean
				 

				 
				SD
				 

				 
				P(incl)
				 

				 
				P(incl|data)
				 

				 
				BF

					 
					inclusion
					 
				 

				 
				Lower
				 

				 
				Upper
				 
			 
		 

		 
			 
				 
				Intercept
				 

				  

				 
				0.004
				 

				  

				 
				0.235
				 

				  

				 
				1.000
				 

				  

				 
				1.000
				 

				  

				 
				1.000
				 

				  

				 
				-0.496
				 

				  

				 
				0.428
				 

				  

			 

			 
				 
				PSS
				 

				  

				 
				0.296
				 

				  

				 
				0.041
				 

				  

				 
				0.500
				 

				  

				 
				1.000
				 

				  

				 
				5.398e +9
				 

				  

				 
				0.208
				 

				  

				 
				0.370
				 

				  
			 

			 
				 
				RSQ
				 

				  

				 
				0.163
				 

				  

				 
				0.034
				 

				  

				 
				0.500
				 

				  

				 
				1.000
				 

				  

				 
				15365.374
				 

				  

				 
				0.090
				 

				  

				 
				0.224
				 

				  
			 

			 
				  
			 
		 

		  
	 
 
   
 
  Posterior Coefficients with 95% Credible Interval         
 
  Residuals vs Fitted         Plotting not possible: subscript out of bounds      
 
  Inclusion Probabilities       
 
  Q-Q Plot       
 
  Marginal Posterior Distributions   
 
  Intercept       
 
  PSS       
 
  RSQ             
 
  RSQ and PSS as preditors of STS   
 
   
 
 
	 
		 
			 
				 
					 
						 
							 
							Model Summary - STS
							 

							  

							  
						 
					 
				 
			 

			 
				 
				Model
				 

				 
				R
				 

				 
				R²
				 

				 
				Adjusted R²
				 

				 
				RMSE
				 
			 
		 

		 
			 
				 
				H₀
				 

				  

				 
				0.000
				 

				  

				 
				0.000
				 

				  

				 
				0.000
				 

				  

				 
				4.501
				 

				  

			 

			 
				 
				H₁
				 

				  

				 
				0.455
				 

				  

				 
				0.207
				 

				  

				 
				0.202
				 

				  

				 
				4.021
				 

				  
			 

			 
				  
			 
		 

		  
	 
 
   
 
 
	 
		 
			 
				 
					 
						 
							 
							ANOVA
							 

							  

							  
						 
					 
				 
			 

			 
				 
				Model
				 

				 
				 
				 

				 
				Sum of Squares
				 

				 
				df
				 

				 
				Mean Square
				 

				 
				F
				 

				 
				p
				 
			 
		 

		 
			 
				 
				H₁
				 

				  

				 
				Regression
				 

				  

				 
				1358.678
				 

				  

				 
				2
				 

				  

				 
				679.339
				 

				  

				 
				42.016
				 

				  

				 
				< .001
				 

				  

			 

			 
				 
				 
				 

				  

				 
				Residual
				 

				  

				 
				5206.232
				 

				  

				 
				322
				 

				  

				 
				16.168
				 

				  

				  

				  

				 
				 
				 

				  
			 

			 
				 
				 
				 

				  

				 
				Total
				 

				  

				 
				6564.911
				 

				  

				 
				324
				 

				  

				  

				  

				  

				  

				 
				 
				 

				  
			 

			 
				  
			 
		 

		 
			 
				 
					 
					Note.
					 

				 The intercept model is omitted, as no meaningful information can be shown.
				 
			 
		 
	 
 
   
 
 
	 
		 
			 
				 
					 
						 
							 
							Coefficients
							 

							  

							  
						 
					 
				 
			 

			 
				 
					  
				 

				 
					 
					95% CI
					 
				 

				 
					 
					Collinearity Statistics
					 
				 
			 

			 
				 
				Model
				 

				 
				 
				 

				 
				Unstandardized
				 

				 
				Standard Error
				 

				 
				Standardized
				 

				 
				t
				 

				 
				p
				 

				 
				Lower
				 

				 
				Upper
				 

				 
				Tolerance
				 

				 
				VIF
				 
			 
		 

		 
			 
				 
				H₀
				 

				  

				 
				(Intercept)
				 

				  

				 
				0.008
				 

				  

				 
				0.250
				 

				  

				  

				  

				 
				0.031
				 

				  

				 
				0.975
				 

				  

				 
				-0.484
				 

				  

				 
				0.499
				 

				  

				 
				 
				 

				  

				  

				  

			 

			 
				 
				H₁
				 

				  

				 
				(Intercept)
				 

				  

				 
				0.006
				 

				  

				 
				0.223
				 

				  

				  

				  

				 
				0.028
				 

				  

				 
				0.978
				 

				  

				 
				-0.433
				 

				  

				 
				0.445
				 

				  

				 
				 
				 

				  

				  

				  
			 

			 
				 
				 
				 

				  

				 
				PSS
				 

				  

				 
				0.247
				 

				  

				 
				0.039
				 

				  

				 
				0.330
				 

				  

				 
				6.251
				 

				  

				 
				< .001
				 

				  

				 
				0.169
				 

				  

				 
				0.324
				 

				  

				 
				0.882
				 

				  

				 
				1.134
				 

				  
			 

			 
				 
				 
				 

				  

				 
				RSQ
				 

				  

				 
				0.135
				 

				  

				 
				0.033
				 

				  

				 
				0.219
				 

				  

				 
				4.146
				 

				  

				 
				< .001
				 

				  

				 
				0.071
				 

				  

				 
				0.199
				 

				  

				 
				0.882
				 

				  

				 
				1.134
				 

				  
			 

			 
				  
			 
		 

		  
	 
 
   
 
 
	 
		 
			 
				 
					 
						 
							 
							Collinearity Diagnostics
							 

							  

							  
						 
					 
				 
			 

			 
				  

				 
				Variance Proportions
				 
			 

			 
				 
				Model
				 

				 
				Dimension
				 

				 
				Eigenvalue
				 

				 
				Condition Index
				 

				 
				(Intercept)
				 

				 
				PSS
				 

				 
				RSQ
				 
			 
		 

		 
			 
				 
				H₁
				 

				  

				 
				1
				 

				  

				 
				1.344
				 

				  

				 
				1.000
				 

				  

				 
				0.000
				 

				  

				 
				0.328
				 

				  

				 
				0.328
				 

				  

			 

			 
				 
				 
				 

				  

				 
				2
				 

				  

				 
				1.000
				 

				  

				 
				1.159
				 

				  

				 
				1.000
				 

				  

				 
				0.000
				 

				  

				 
				0.000
				 

				  
			 

			 
				 
				 
				 

				  

				 
				3
				 

				  

				 
				0.656
				 

				  

				 
				1.431
				 

				  

				 
				0.000
				 

				  

				 
				0.672
				 

				  

				 
				0.672
				 

				  
			 

			 
				  
			 
		 

		 
			 
				 
					 
					Note.
					 

				 The intercept model is omitted, as no meaningful information can be shown.
				 
			 
		 
	 
 
   
 
 
	 
		 
			 
				 
					 
						 
							 
							Residuals Statistics
							 

							  

							  
						 
					 
				 
			 

			 
				 
				 
				 

				 
				Minimum
				 

				 
				Maximum
				 

				 
				Mean
				 

				 
				SD
				 

				 
				N
				 
			 
		 

		 
			 
				 
				Predicted Value
				 

				  

				 
				-4.827
				 

				  

				 
				6.010
				 

				  

				 
				0.008
				 

				  

				 
				2.048
				 

				  

				 
				325
				 

				  

			 

			 
				 
				Residual
				 

				  

				 
				-12.199
				 

				  

				 
				16.304
				 

				  

				 
				-9.597e -17
				 

				  

				 
				4.009
				 

				  

				 
				325
				 

				  
			 

			 
				 
				Std. Predicted Value
				 

				  

				 
				-2.361
				 

				  

				 
				2.931
				 

				  

				 
				-6.612e -18
				 

				  

				 
				1.000
				 

				  

				 
				325
				 

				  
			 

			 
				 
				Std. Residual
				 

				  

				 
				-3.048
				 

				  

				 
				4.069
				 

				  

				 
				-4.092e 


				-4
				 

				  

				 
				1.002
				 

				  

				 
				325
				 

				  
			 

			 
				  
			 
		 

		  
	 
 
   
 
  Residuals vs. Predicted       
 
  Q-Q Plot Standardized Residuals           
 
  RSQ and PSS as predictors of STS. Bayesian inference   
 
   
 
 
	 
		 
			 
				 
					 
						 
							 
							Model Comparison - STS
							 

							  

							  
						 
					 
				 
			 

			 
				 
				Models
				 

				 
				P(M)
				 

				 
				P(M|data)
				 

				 
				BF

					 
					M
					 
				 

				 
				BF

					 
					10
					 
				 

				 
				R²
				 
			 
		 

		 
			 
				 
				Null model
				 

				  

				 
				0.333
				 

				  

				 
				1.042e -14
				 

				  

				 
				2.084e -14
				 

				  

				 
				1.000
				 

				  

				 
				0.000
				 

				  

			 

			 
				 
				PSS + RSQ
				 

				  

				 
				0.333
				 

				  

				 
				0.999
				 

				  

				 
				1711.949
				 

				  

				 
				9.586e +13
				 

				  

				 
				0.207
				 

				  
			 

			 
				 
				PSS
				 

				  

				 
				0.167
				 

				  

				 
				0.001
				 

				  

				 
				0.006
				 

				  

				 
				2.240e +11
				 

				  

				 
				0.165
				 

				  
			 

			 
				 
				RSQ
				 

				  

				 
				0.167
				 

				  

				 
				5.986e 


				-8
				 

				  

				 
				2.993e 


				-7
				 

				  

				 
				1.149e 


				+7
				 

				  

				 
				0.111
				 

				  
			 

			 
				  
			 
		 

		  
	 
 
   
 
  Posterior Summary   
 
 
	 
		 
			 
				 
					 
						 
							 
							Posterior Summaries of Coefficients
							 

							  

							  
						 
					 
				 
			 

			 
				  

				 
				95% Credible Interval
				 
			 

			 
				 
				Coefficient
				 

				 
				Mean
				 

				 
				SD
				 

				 
				P(incl)
				 

				 
				P(incl|data)
				 

				 
				BF

					 
					inclusion
					 
				 

				 
				Lower
				 

				 
				Upper
				 
			 
		 

		 
			 
				 
				Intercept
				 

				  

				 
				0.008
				 

				  

				 
				0.223
				 

				  

				 
				1.000
				 

				  

				 
				1.000
				 

				  

				 
				1.000
				 

				  

				 
				-0.428
				 

				  

				 
				0.491
				 

				  

			 

			 
				 
				PSS
				 

				  

				 
				0.241
				 

				  

				 
				0.039
				 

				  

				 
				0.500
				 

				  

				 
				1.000
				 

				  

				 
				1.670e +7
				 

				  

				 
				0.165
				 

				  

				 
				0.325
				 

				  
			 

			 
				 
				RSQ
				 

				  

				 
				0.132
				 

				  

				 
				0.032
				 

				  

				 
				0.500
				 

				  

				 
				0.999
				 

				  

				 
				856.018
				 

				  

				 
				0.069
				 

				  

				 
				0.201
				 

				  
			 

			 
				  
			 
		 

		  
	 
 
   
 
  Posterior Coefficients with 95% Credible Interval         
 
  Inclusion Probabilities       
 
  Q-Q Plot       
 
  Marginal Posterior Distributions   
 
  Intercept       
 
  PSS       
 
  RSQ             
 
  SE, LOT, SC, STS, BU, CS, AV, AP as predictors of RESIL    We tested the relationship between potential mediators and resilience using a multiple regression analysys.    
 
   
 
 
	 
		 
			 
				 
					 
						 
							 
							Model Summary - RESIL
							 

							  

							  
						 
					 
				 
			 

			 
				  

				 
				Durbin-Watson
				 
			 

			 
				 
				Model
				 

				 
				R
				 

				 
				R²
				 

				 
				Adjusted R²
				 

				 
				RMSE
				 

				 
				Autocorrelation
				 

				 
				Statistic
				 

				 
				p
				 
			 
		 

		 
			 
				 
				H₀
				 

				  

				 
				0.000
				 

				  

				 
				0.000
				 

				  

				 
				0.000
				 

				  

				 
				11.171
				 

				  

				 
				0.045
				 

				  

				 
				1.908
				 

				  

				 
				0.407
				 

				  

			 

			 
				 
				H₁
				 

				  

				 
				0.810
				 

				  

				 
				0.656
				 

				  

				 
				0.647
				 

				  

				 
				6.638
				 

				  

				 
				-0.077
				 

				  

				 
				2.151
				 

				  

				 
				0.174
				 

				  
			 

			 
				  
			 
		 

		  
	 
 
  The Model summary shows that the potential mediators accounts for 63.6% of resilience variance      
 
 
	 
		 
			 
				 
					 
						 
							 
							ANOVA
							 

							  

							  
						 
					 
				 
			 

			 
				 
				Model
				 

				 
				 
				 

				 
				Sum of Squares
				 

				 
				df
				 

				 
				Mean Square
				 

				 
				F
				 

				 
				p
				 
			 
		 

		 
			 
				 
				H₁
				 

				  

				 
				Regression
				 

				  

				 
				26429.220
				 

				  

				 
				8
				 

				  

				 
				3303.653
				 

				  

				 
				74.976
				 

				  

				 
				< .001
				 

				  

			 

			 
				 
				 
				 

				  

				 
				Residual
				 

				  

				 
				13879.752
				 

				  

				 
				315
				 

				  

				 
				44.063
				 

				  

				  

				  

				 
				 
				 

				  
			 

			 
				 
				 
				 

				  

				 
				Total
				 

				  

				 
				40308.972
				 

				  

				 
				323
				 

				  

				  

				  

				  

				  

				 
				 
				 

				  
			 

			 
				  
			 
		 

		 
			 
				 
					 
					Note.
					 

				 The intercept model is omitted, as no meaningful information can be shown.
				 
			 
		 
	 
 
   
 
 
	 
		 
			 
				 
					 
						 
							 
							Coefficients
							 

							  

							  
						 
					 
				 
			 

			 
				 
					  
				 

				 
					 
					95% CI
					 
				 

				 
					 
					Collinearity Statistics
					 
				 
			 

			 
				 
				Model
				 

				 
				 
				 

				 
				Unstandardized
				 

				 
				Standard Error
				 

				 
				Standardized
				 

				 
				t
				 

				 
				p
				 

				 
				Lower
				 

				 
				Upper
				 

				 
				Tolerance
				 

				 
				VIF
				 
			 
		 

		 
			 
				 
				H₀
				 

				  

				 
				(Intercept)
				 

				  

				 
				-0.061
				 

				  

				 
				0.621
				 

				  

				  

				  

				 
				-0.098
				 

				  

				 
				0.922
				 

				  

				 
				-1.282
				 

				  

				 
				1.160
				 

				  

				 
				 
				 

				  

				  

				  

			 

			 
				 
				H₁
				 

				  

				 
				(Intercept)
				 

				  

				 
				-0.040
				 

				  

				 
				0.369
				 

				  

				  

				  

				 
				-0.109
				 

				  

				 
				0.913
				 

				  

				 
				-0.766
				 

				  

				 
				0.685
				 

				  

				 
				 
				 

				  

				  

				  
			 

			 
				 
				 
				 

				  

				 
				SE
				 

				  

				 
				1.040
				 

				  

				 
				0.125
				 

				  

				 
				0.345
				 

				  

				 
				8.298
				 

				  

				 
				< .001
				 

				  

				 
				0.793
				 

				  

				 
				1.286
				 

				  

				 
				0.631
				 

				  

				 
				1.585
				 

				  
			 

			 
				 
				 
				 

				  

				 
				LOT
				 

				  

				 
				0.284
				 

				  

				 
				0.097
				 

				  

				 
				0.126
				 

				  

				 
				2.932
				 

				  

				 
				0.004
				 

				  

				 
				0.094
				 

				  

				 
				0.475
				 

				  

				 
				0.595
				 

				  

				 
				1.680
				 

				  
			 

			 
				 
				 
				 

				  

				 
				SC
				 

				  

				 
				0.211
				 

				  

				 
				0.056
				 

				  

				 
				0.168
				 

				  

				 
				3.740
				 

				  

				 
				< .001
				 

				  

				 
				0.100
				 

				  

				 
				0.322
				 

				  

				 
				0.542
				 

				  

				 
				1.845
				 

				  
			 

			 
				 
				 
				 

				  

				 
				STS
				 

				  

				 
				0.234
				 

				  

				 
				0.099
				 

				  

				 
				0.094
				 

				  

				 
				2.352
				 

				  

				 
				0.019
				 

				  

				 
				0.038
				 

				  

				 
				0.429
				 

				  

				 
				0.681
				 

				  

				 
				1.468
				 

				  
			 

			 
				 
				 
				 

				  

				 
				BU
				 

				  

				 
				-0.444
				 

				  

				 
				0.127
				 

				  

				 
				-0.196
				 

				  

				 
				-3.504
				 

				  

				 
				< .001
				 

				  

				 
				-0.694
				 

				  

				 
				-0.195
				 

				  

				 
				0.351
				 

				  

				 
				2.849
				 

				  
			 

			 
				 
				 
				 

				  

				 
				CS
				 

				  

				 
				0.345
				 

				  

				 
				0.105
				 

				  

				 
				0.155
				 

				  

				 
				3.284
				 

				  

				 
				0.001
				 

				  

				 
				0.138
				 

				  

				 
				0.551
				 

				  

				 
				0.490
				 

				  

				 
				2.039
				 

				  
			 

			 
				 
				 
				 

				  

				 
				AV
				 

				  

				 
				-0.205
				 

				  

				 
				0.113
				 

				  

				 
				-0.075
				 

				  

				 
				-1.815
				 

				  

				 
				0.070
				 

				  

				 
				-0.427
				 

				  

				 
				0.017
				 

				  

				 
				0.643
				 

				  

				 
				1.556
				 

				  
			 

			 
				 
				 
				 

				  

				 
				AP
				 

				  

				 
				0.279
				 

				  

				 
				0.072
				 

				  

				 
				0.146
				 

				  

				 
				3.851
				 

				  

				 
				< .001
				 

				  

				 
				0.136
				 

				  

				 
				0.421
				 

				  

				 
				0.763
				 

				  

				 
				1.311
				 

				  
			 

			 
				  
			 
		 

		  
	 
 
  The table indicates that all potential mediators are good predictors of resilience scores excluding AV     
 
 
	 
		 
			 
				 
					 
						 
							 
							Descriptives
							 

							  

							  
						 
					 
				 
			 

			 
				 
				 
				 

				 
				N
				 

				 
				Mean
				 

				 
				SD
				 

				 
				SE
				 
			 
		 

		 
			 
				 
				RESIL
				 

				  

				 
				324
				 

				  

				 
				-0.061
				 

				  

				 
				11.171
				 

				  

				 
				0.621
				 

				  

			 

			 
				 
				SE
				 

				  

				 
				324
				 

				  

				 
				-0.004
				 

				  

				 
				3.711
				 

				  

				 
				0.206
				 

				  
			 

			 
				 
				LOT
				 

				  

				 
				324
				 

				  

				 
				-0.002
				 

				  

				 
				4.936
				 

				  

				 
				0.274
				 

				  
			 

			 
				 
				SC
				 

				  

				 
				324
				 

				  

				 
				-0.021
				 

				  

				 
				8.891
				 

				  

				 
				0.494
				 

				  
			 

			 
				 
				STS
				 

				  

				 
				324
				 

				  

				 
				-1.235e -4
				 

				  

				 
				4.506
				 

				  

				 
				0.250
				 

				  
			 

			 
				 
				BU
				 

				  

				 
				324
				 

				  

				 
				0.015
				 

				  

				 
				4.918
				 

				  

				 
				0.273
				 

				  
			 

			 
				 
				CS
				 

				  

				 
				324
				 

				  

				 
				-0.014
				 

				  

				 
				5.028
				 

				  

				 
				0.279
				 

				  
			 

			 
				 
				AV
				 

				  

				 
				324
				 

				  

				 
				0.004
				 

				  

				 
				4.084
				 

				  

				 
				0.227
				 

				  
			 

			 
				 
				AP
				 

				  

				 
				324
				 

				  

				 
				0.004
				 

				  

				 
				5.845
				 

				  

				 
				0.325
				 

				  
			 

			 
				  
			 
		 

		  
	 
 
   
 
 
	 
		 
			 
				 
					 
						 
							 
							Collinearity Diagnostics
							 

							  

							  
						 
					 
				 
			 

			 
				  

				 
				Variance Proportions
				 
			 

			 
				 
				Model
				 

				 
				Dimension
				 

				 
				Eigenvalue
				 

				 
				Condition Index
				 

				 
				(Intercept)
				 

				 
				SE
				 

				 
				LOT
				 

				 
				SC
				 

				 
				STS
				 

				 
				BU
				 

				 
				CS
				 

				 
				AV
				 

				 
				AP
				 
			 
		 

		 
			 
				 
				H₁
				 

				  

				 
				1
				 

				  

				 
				3.418
				 

				  

				 
				1.000
				 

				  

				 
				0.000
				 

				  

				 
				0.028
				 

				  

				 
				0.026
				 

				  

				 
				0.028
				 

				  

				 
				0.017
				 

				  

				 
				0.021
				 

				  

				 
				0.018
				 

				  

				 
				0.014
				 

				  

				 
				0.006
				 

				  

			 

			 
				 
				 
				 

				  

				 
				2
				 

				  

				 
				1.369
				 

				  

				 
				1.580
				 

				  

				 
				0.000
				 

				  

				 
				0.000
				 

				  

				 
				0.003
				 

				  

				 
				0.000
				 

				  

				 
				0.061
				 

				  

				 
				0.001
				 

				  

				 
				0.034
				 

				  

				 
				0.161
				 

				  

				 
				0.238
				 

				  
			 

			 
				 
				 
				 

				  

				 
				3
				 

				  

				 
				1.000
				 

				  

				 
				1.849
				 

				  

				 
				1.000
				 

				  

				 
				0.000
				 

				  

				 
				0.000
				 

				  

				 
				0.000
				 

				  

				 
				0.000
				 

				  

				 
				0.000
				 

				  

				 
				0.000
				 

				  

				 
				0.000
				 

				  

				 
				0.000
				 

				  
			 

			 
				 
				 
				 

				  

				 
				4
				 

				  

				 
				0.808
				 

				  

				 
				2.057
				 

				  

				 
				0.000
				 

				  

				 
				0.024
				 

				  

				 
				0.174
				 

				  

				 
				0.080
				 

				  

				 
				0.185
				 

				  

				 
				0.064
				 

				  

				 
				0.121
				 

				  

				 
				0.027
				 

				  

				 
				0.015
				 

				  
			 

			 
				 
				 
				 

				  

				 
				5
				 

				  

				 
				0.714
				 

				  

				 
				2.188
				 

				  

				 
				0.000
				 

				  

				 
				0.003
				 

				  

				 
				0.001
				 

				  

				 
				0.000
				 

				  

				 
				0.426
				 

				  

				 
				0.004
				 

				  

				 
				0.195
				 

				  

				 
				0.017
				 

				  

				 
				0.254
				 

				  
			 

			 
				 
				 
				 

				  

				 
				6
				 

				  

				 
				0.570
				 

				  

				 
				2.449
				 

				  

				 
				0.000
				 

				  

				 
				0.855
				 

				  

				 
				0.015
				 

				  

				 
				0.115
				 

				  

				 
				0.003
				 

				  

				 
				0.033
				 

				  

				 
				0.000
				 

				  

				 
				0.037
				 

				  

				 
				0.006
				 

				  
			 

			 
				 
				 
				 

				  

				 
				7
				 

				  

				 
				0.492
				 

				  

				 
				2.636
				 

				  

				 
				0.000
				 

				  

				 
				0.016
				 

				  

				 
				0.570
				 

				  

				 
				0.055
				 

				  

				 
				0.035
				 

				  

				 
				0.007
				 

				  

				 
				0.002
				 

				  

				 
				0.304
				 

				  

				 
				0.303
				 

				  
			 

			 
				 
				 
				 

				  

				 
				8
				 

				  

				 
				0.402
				 

				  

				 
				2.916
				 

				  

				 
				0.000
				 

				  

				 
				0.010
				 

				  

				 
				0.130
				 

				  

				 
				0.722
				 

				  

				 
				0.020
				 

				  

				 
				0.017
				 

				  

				 
				0.000
				 

				  

				 
				0.399
				 

				  

				 
				0.168
				 

				  
			 

			 
				 
				 
				 

				  

				 
				9
				 

				  

				 
				0.227
				 

				  

				 
				3.877
				 

				  

				 
				0.000
				 

				  

				 
				0.064
				 

				  

				 
				0.080
				 

				  

				 
				0.001
				 

				  

				 
				0.252
				 

				  

				 
				0.853
				 

				  

				 
				0.630
				 

				  

				 
				0.040
				 

				  

				 
				0.011
				 

				  
			 

			 
				  
			 
		 

		 
			 
				 
					 
					Note.
					 

				 The intercept model is omitted, as no meaningful information can be shown.
				 
			 
		 
	 
 
   
 
 
	 
		 
			 
				 
					 
						 
							 
							Casewise Diagnostics
							 

							  

							  
						 
					 
				 
			 

			 
				 
				Case Number
				 

				 
				Std. Residual
				 

				 
				RESIL
				 

				 
				Predicted Value
				 

				 
				Residual
				 

				 
				Cook's Distance
				 
			 
		 

		 
			 
				 
				112
				 

				  

				 
				3.374
				 

				  

				 
				5.930
				 

				  

				 
				-15.965
				 

				  

				 
				21.895
				 

				  

				 
				0.058
				 

				  

			 

			 
				 
				170
				 

				  

				 
				-3.252
				 

				  

				 
				-8.070
				 

				  

				 
				13.299
				 

				  

				 
				-21.369
				 

				  

				 
				0.024
				 

				  
			 

			 
				  
			 
		 

		  
	 
 
   
 
 
	 
		 
			 
				 
					 
						 
							 
							Residuals Statistics
							 

							  

							  
						 
					 
				 
			 

			 
				 
				 
				 

				 
				Minimum
				 

				 
				Maximum
				 

				 
				Mean
				 

				 
				SD
				 

				 
				N
				 
			 
		 

		 
			 
				 
				Predicted Value
				 

				  

				 
				-47.805
				 

				  

				 
				18.142
				 

				  

				 
				-0.061
				 

				  

				 
				9.046
				 

				  

				 
				324
				 

				  

			 

			 
				 
				Residual
				 

				  

				 
				-21.369
				 

				  

				 
				21.895
				 

				  

				 
				8.209e -17
				 

				  

				 
				6.555
				 

				  

				 
				324
				 

				  
			 

			 
				 
				Std. Predicted Value
				 

				  

				 
				-5.278
				 

				  

				 
				2.012
				 

				  

				 
				1.474e -17
				 

				  

				 
				1.000
				 

				  

				 
				324
				 

				  
			 

			 
				 
				Std. Residual
				 

				  

				 
				-3.252
				 

				  

				 
				3.374
				 

				  

				 
				-2.794e 


				-4
				 

				  

				 
				1.001
				 

				  

				 
				324
				 

				  
			 

			 
				  
			 
		 

		  
	 
 
   
 
  Q-Q Plot Standardized Residuals          The  balanced distribution of the residuals around the baseline suggests that the assumption of homoscedasticity has not been violated. The Q-Q plot shows that the standardized residuals fit along the diagonal suggesting that both assumptions or normality and linearity have also not been violated.      
 
  SE, LOT, SC, STS, BU, CS, AV, AP as predictors of RESIL. Bayesian inference   
 
   
 
 
	 
		 
			 
				 
					 
						 
							 
							Model Comparison - RESIL
							 

							  

							  
						 
					 
				 
			 

			 
				 
				Models
				 

				 
				P(M)
				 

				 
				P(M|data)
				 

				 
				BF

					 
					M
					 
				 

				 
				BF

					 
					10
					 
				 

				 
				R²
				 
			 
		 

		 
			 
				 
				Null model
				 

				  

				 
				0.111
				 

				  

				 
				7.536e -66
				 

				  

				 
				6.029e -65
				 

				  

				 
				1.000
				 

				  

				 
				0.000
				 

				  

			 

			 
				 
				AV + AP + SE + LOT + SC + BU + STS + CS
				 

				  

				 
				0.111
				 

				  

				 
				0.695
				 

				  

				 
				18.225
				 

				  

				 
				9.221e +64
				 

				  

				 
				0.656
				 

				  
			 

			 
				 
				AP + SE + LOT + SC + BU + STS + CS
				 

				  

				 
				0.014
				 

				  

				 
				0.158
				 

				  

				 
				13.343
				 

				  

				 
				1.679e +65
				 

				  

				 
				0.652
				 

				  
			 

			 
				 
				AV + AP + SE + LOT + SC + BU + CS
				 

				  

				 
				0.014
				 

				  

				 
				0.053
				 

				  

				 
				3.982
				 

				  

				 
				5.637e +64
				 

				  

				 
				0.650
				 

				  
			 

			 
				 
				AP + SE + LOT + SC + BU + CS
				 

				  

				 
				0.004
				 

				  

				 
				0.050
				 

				  

				 
				13.334
				 

				  

				 
				1.874e +65
				 

				  

				 
				0.647
				 

				  
			 

			 
				 
				AV + AP + SE + SC + BU + STS + CS
				 

				  

				 
				0.014
				 

				  

				 
				0.012
				 

				  

				 
				0.868
				 

				  

				 
				1.283e +64
				 

				  

				 
				0.646
				 

				  
			 

			 
				 
				AP + SE + SC + BU + STS + CS
				 

				  

				 
				0.004
				 

				  

				 
				0.005
				 

				  

				 
				1.347
				 

				  

				 
				1.983e +64
				 

				  

				 
				0.642
				 

				  
			 

			 
				 
				AP + SE + LOT + SC + BU + STS
				 

				  

				 
				0.004
				 

				  

				 
				0.005
				 

				  

				 
				1.166
				 

				  

				 
				1.718e +64
				 

				  

				 
				0.642
				 

				  
			 

			 
				 
				AV + AP + SE + LOT + SC + BU + STS
				 

				  

				 
				0.014
				 

				  

				 
				0.004
				 

				  

				 
				0.301
				 

				  

				 
				4.484e +63
				 

				  

				 
				0.644
				 

				  
			 

			 
				 
				AV + AP + SE + LOT + SC + CS
				 

				  

				 
				0.004
				 

				  

				 
				0.003
				 

				  

				 
				0.860
				 

				  

				 
				1.268e +64
				 

				  

				 
				0.641
				 

				  
			 

			 
				  
			 
		 

		 
			 
				 
					 
					Note.
					 

				 Table displays only a subset of models; to see all models, select "No" under "Limit No. Models Shown".
				 
			 
		 
	 
 
   
 
  Posterior Summary   
 
 
	 
		 
			 
				 
					 
						 
							 
							Posterior Summaries of Coefficients
							 

							  

							  
						 
					 
				 
			 

			 
				  

				 
				95% Credible Interval
				 
			 

			 
				 
				Coefficient
				 

				 
				Mean
				 

				 
				SD
				 

				 
				P(incl)
				 

				 
				P(incl|data)
				 

				 
				BF

					 
					inclusion
					 
				 

				 
				Lower
				 

				 
				Upper
				 
			 
		 

		 
			 
				 
				Intercept
				 

				  

				 
				-0.061
				 

				  

				 
				0.370
				 

				  

				 
				1.000
				 

				  

				 
				1.000
				 

				  

				 
				1.000
				 

				  

				 
				-0.823
				 

				  

				 
				0.613
				 

				  

			 

			 
				 
				AV
				 

				  

				 
				-0.154
				 

				  

				 
				0.130
				 

				  

				 
				0.500
				 

				  

				 
				0.774
				 

				  

				 
				3.418
				 

				  

				 
				-0.404
				 

				  

				 
				0.006
				 

				  
			 

			 
				 
				AP
				 

				  

				 
				0.266
				 

				  

				 
				0.075
				 

				  

				 
				0.500
				 

				  

				 
				0.997
				 

				  

				 
				361.183
				 

				  

				 
				0.123
				 

				  

				 
				0.428
				 

				  
			 

			 
				 
				SE
				 

				  

				 
				1.031
				 

				  

				 
				0.127
				 

				  

				 
				0.500
				 

				  

				 
				1.000
				 

				  

				 
				9.503e +12
				 

				  

				 
				0.791
				 

				  

				 
				1.275
				 

				  
			 

			 
				 
				LOT
				 

				  

				 
				0.278
				 

				  

				 
				0.105
				 

				  

				 
				0.500
				 

				  

				 
				0.978
				 

				  

				 
				44.161
				 

				  

				 
				0.077
				 

				  

				 
				0.498
				 

				  
			 

			 
				 
				SC
				 

				  

				 
				0.215
				 

				  

				 
				0.058
				 

				  

				 
				0.500
				 

				  

				 
				0.999
				 

				  

				 
				781.193
				 

				  

				 
				0.102
				 

				  

				 
				0.324
				 

				  
			 

			 
				 
				BU
				 

				  

				 
				-0.434
				 

				  

				 
				0.137
				 

				  

				 
				0.500
				 

				  

				 
				0.992
				 

				  

				 
				125.099
				 

				  

				 
				-0.720
				 

				  

				 
				-0.166
				 

				  
			 

			 
				 
				STS
				 

				  

				 
				0.201
				 

				  

				 
				0.118
				 

				  

				 
				0.500
				 

				  

				 
				0.886
				 

				  

				 
				7.781
				 

				  

				 
				0.000
				 

				  

				 
				0.400
				 

				  
			 

			 
				 
				CS
				 

				  

				 
				0.338
				 

				  

				 
				0.112
				 

				  

				 
				0.500
				 

				  

				 
				0.990
				 

				  

				 
				94.866
				 

				  

				 
				0.109
				 

				  

				 
				0.562
				 

				  
			 

			 
				  
			 
		 

		  
	 
 
   
 
  Posterior Coefficients with 95% Credible Interval         
 
  Residuals vs Fitted       
 
  Inclusion Probabilities       
 
  Q-Q Plot           
 
  Mediation Analysis   
 
   
 
  Parameter estimates   
 
 
	 
		 
			 
				 
					 
						 
							 
							Direct effects
							 

							  

							  
						 
					 
				 
			 

			 
				  

				 
				95% Confidence Interval
				 
			 

			 
				 
				 
				 

				 
				 
				 

				 
				 
				 

				 
				Estimate
				 

				 
				Std. Error
				 

				 
				z-value
				 

				 
				p
				 

				 
				Lower
				 

				 
				Upper
				 
			 
		 

		 
			 
				 
				PSS
				 

				  

				 
				→
				 

				  

				 
				RESIL
				 

				  

				 
				-0.001
				 

				  

				 
				0.007
				 

				  

				 
				-0.202
				 

				  

				 
				0.840
				 

				  

				 
				-0.015
				 

				  

				 
				0.012
				 

				  

			 

			 
				 
				RSQ
				 

				  

				 
				→
				 

				  

				 
				RESIL
				 

				  

				 
				0.004
				 

				  

				 
				0.005
				 

				  

				 
				0.689
				 

				  

				 
				0.491
				 

				  

				 
				-0.006
				 

				  

				 
				0.013
				 

				  
			 

			 
				  
			 
		 

		 
			 
				 
					 
					Note.
					 

				 Delta method standard errors, bias-corrected percentile bootstrap confidence intervals, ML estimator.
				 
			 
		 
	 
 
   
 
 
	 
		 
			 
				 
					 
						 
							 
							Indirect effects
							 

							  

							  
						 
					 
				 
			 

			 
				  

				 
				95% Confidence Interval
				 
			 

			 
				 
				 
				 

				 
				 
				 

				 
				 
				 

				 
				 
				 

				 
				 
				 

				 
				Estimate
				 

				 
				Std. Error
				 

				 
				z-value
				 

				 
				p
				 

				 
				Lower
				 

				 
				Upper
				 
			 
		 

		 
			 
				 
				PSS
				 

				  

				 
				→
				 

				  

				 
				SE
				 

				  

				 
				→
				 

				  

				 
				RESIL
				 

				  

				 
				-0.025
				 

				  

				 
				0.004
				 

				  

				 
				-5.940
				 

				  

				 
				< .001
				 

				  

				 
				-0.036
				 

				  

				 
				-0.017
				 

				  

			 

			 
				 
				PSS
				 

				  

				 
				→
				 

				  

				 
				LOT
				 

				  

				 
				→
				 

				  

				 
				RESIL
				 

				  

				 
				-0.009
				 

				  

				 
				0.003
				 

				  

				 
				-3.090
				 

				  

				 
				0.002
				 

				  

				 
				-0.017
				 

				  

				 
				-0.003
				 

				  
			 

			 
				 
				PSS
				 

				  

				 
				→
				 

				  

				 
				SC
				 

				  

				 
				→
				 

				  

				 
				RESIL
				 

				  

				 
				-0.016
				 

				  

				 
				0.004
				 

				  

				 
				-4.009
				 

				  

				 
				< .001
				 

				  

				 
				-0.024
				 

				  

				 
				-0.008
				 

				  
			 

			 
				 
				PSS
				 

				  

				 
				→
				 

				  

				 
				CS
				 

				  

				 
				→
				 

				  

				 
				RESIL
				 

				  

				 
				-0.009
				 

				  

				 
				0.003
				 

				  

				 
				-2.927
				 

				  

				 
				0.003
				 

				  

				 
				-0.016
				 

				  

				 
				-0.004
				 

				  
			 

			 
				 
				PSS
				 

				  

				 
				→
				 

				  

				 
				BU
				 

				  

				 
				→
				 

				  

				 
				RESIL
				 

				  

				 
				-0.014
				 

				  

				 
				0.004
				 

				  

				 
				-3.542
				 

				  

				 
				< .001
				 

				  

				 
				-0.023
				 

				  

				 
				-0.007
				 

				  
			 

			 
				 
				PSS
				 

				  

				 
				→
				 

				  

				 
				STS
				 

				  

				 
				→
				 

				  

				 
				RESIL
				 

				  

				 
				0.005
				 

				  

				 
				0.002
				 

				  

				 
				2.253
				 

				  

				 
				0.024
				 

				  

				 
				9.214e -4
				 

				  

				 
				0.011
				 

				  
			 

			 
				 
				RSQ
				 

				  

				 
				→
				 

				  

				 
				SE
				 

				  

				 
				→
				 

				  

				 
				RESIL
				 

				  

				 
				-0.007
				 

				  

				 
				0.003
				 

				  

				 
				-2.543
				 

				  

				 
				0.011
				 

				  

				 
				-0.012
				 

				  

				 
				-0.002
				 

				  
			 

			 
				 
				RSQ
				 

				  

				 
				→
				 

				  

				 
				LOT
				 

				  

				 
				→
				 

				  

				 
				RESIL
				 

				  

				 
				-0.004
				 

				  

				 
				0.002
				 

				  

				 
				-2.541
				 

				  

				 
				0.011
				 

				  

				 
				-0.008
				 

				  

				 
				-0.001
				 

				  
			 

			 
				 
				RSQ
				 

				  

				 
				→
				 

				  

				 
				SC
				 

				  

				 
				→
				 

				  

				 
				RESIL
				 

				  

				 
				-0.003
				 

				  

				 
				0.002
				 

				  

				 
				-2.124
				 

				  

				 
				0.034
				 

				  

				 
				-0.007
				 

				  

				 
				-7.178e -4
				 

				  
			 

			 
				 
				RSQ
				 

				  

				 
				→
				 

				  

				 
				CS
				 

				  

				 
				→
				 

				  

				 
				RESIL
				 

				  

				 
				-0.001
				 

				  

				 
				0.001
				 

				  

				 
				-0.904
				 

				  

				 
				0.366
				 

				  

				 
				-0.004
				 

				  

				 
				0.001
				 

				  
			 

			 
				 
				RSQ
				 

				  

				 
				→
				 

				  

				 
				BU
				 

				  

				 
				→
				 

				  

				 
				RESIL
				 

				  

				 
				-0.008
				 

				  

				 
				0.002
				 

				  

				 
				-3.118
				 

				  

				 
				0.002
				 

				  

				 
				-0.014
				 

				  

				 
				-0.004
				 

				  
			 

			 
				 
				RSQ
				 

				  

				 
				→
				 

				  

				 
				STS
				 

				  

				 
				→
				 

				  

				 
				RESIL
				 

				  

				 
				0.003
				 

				  

				 
				0.001
				 

				  

				 
				2.088
				 

				  

				 
				0.037
				 

				  

				 
				5.809e -4
				 

				  

				 
				0.007
				 

				  
			 

			 
				  
			 
		 

		 
			 
				 
					 
					Note.
					 

				 Delta method standard errors, bias-corrected percentile bootstrap confidence intervals, ML estimator.
				 
			 
		 
	 
 
   
 
 
	 
		 
			 
				 
					 
						 
							 
							Total effects
							 

							  

							  
						 
					 
				 
			 

			 
				  

				 
				95% Confidence Interval
				 
			 

			 
				 
				 
				 

				 
				 
				 

				 
				 
				 

				 
				Estimate
				 

				 
				Std. Error
				 

				 
				z-value
				 

				 
				p
				 

				 
				Lower
				 

				 
				Upper
				 
			 
		 

		 
			 
				 
				PSS
				 

				  

				 
				→
				 

				  

				 
				RESIL
				 

				  

				 
				-0.069
				 

				  

				 
				0.009
				 

				  

				 
				-8.053
				 

				  

				 
				< .001
				 

				  

				 
				-0.092
				 

				  

				 
				-0.049
				 

				  

			 

			 
				 
				RSQ
				 

				  

				 
				→
				 

				  

				 
				RESIL
				 

				  

				 
				-0.016
				 

				  

				 
				0.007
				 

				  

				 
				-2.303
				 

				  

				 
				0.021
				 

				  

				 
				-0.029
				 

				  

				 
				-0.003
				 

				  
			 

			 
				  
			 
		 

		 
			 
				 
					 
					Note.
					 

				 Delta method standard errors, bias-corrected percentile bootstrap confidence intervals, ML estimator.
				 
			 
		 
	 
 
   
 
 
	 
		 
			 
				 
					 
						 
							 
							Total indirect effects
							 

							  

							  
						 
					 
				 
			 

			 
				  

				 
				95% Confidence Interval
				 
			 

			 
				 
				 
				 

				 
				 
				 

				 
				 
				 

				 
				Estimate
				 

				 
				Std. Error
				 

				 
				z-value
				 

				 
				p
				 

				 
				Lower
				 

				 
				Upper
				 
			 
		 

		 
			 
				 
				PSS
				 

				  

				 
				→
				 

				  

				 
				RESIL
				 

				  

				 
				-0.068
				 

				  

				 
				0.008
				 

				  

				 
				-8.949
				 

				  

				 
				< .001
				 

				  

				 
				-0.088
				 

				  

				 
				-0.051
				 

				  

			 

			 
				 
				RSQ
				 

				  

				 
				→
				 

				  

				 
				RESIL
				 

				  

				 
				-0.020
				 

				  

				 
				0.006
				 

				  

				 
				-3.612
				 

				  

				 
				< .001
				 

				  

				 
				-0.031
				 

				  

				 
				-0.010
				 

				  
			 

			 
				  
			 
		 

		 
			 
				 
					 
					Note.
					 

				 Delta method standard errors, bias-corrected percentile bootstrap confidence intervals, ML estimator.
				 
			 
		 
	 
 
   
 
 
	 
		 
			 
				 
					 
						 
							 
							Residual covariances
							 

							  

							  
						 
					 
				 
			 

			 
				  

				 
				95% Confidence Interval
				 
			 

			 
				 
				 
				 

				 
				 
				 

				 
				 
				 

				 
				Estimate
				 

				 
				Std. Error
				 

				 
				z-value
				 

				 
				p
				 

				 
				Lower
				 

				 
				Upper
				 
			 
		 

		 
			 
				 
				SE
				 

				  

				 
				↔
				 

				  

				 
				LOT
				 

				  

				 
				0.230
				 

				  

				 
				0.044
				 

				  

				 
				5.231
				 

				  

				 
				< .001
				 

				  

				 
				0.151
				 

				  

				 
				0.331
				 

				  

			 

			 
				 
				SE
				 

				  

				 
				↔
				 

				  

				 
				SC
				 

				  

				 
				0.207
				 

				  

				 
				0.042
				 

				  

				 
				4.893
				 

				  

				 
				< .001
				 

				  

				 
				0.124
				 

				  

				 
				0.291
				 

				  
			 

			 
				 
				LOT
				 

				  

				 
				↔
				 

				  

				 
				SC
				 

				  

				 
				0.285
				 

				  

				 
				0.044
				 

				  

				 
				6.556
				 

				  

				 
				< .001
				 

				  

				 
				0.196
				 

				  

				 
				0.408
				 

				  
			 

			 
				 
				SE
				 

				  

				 
				↔
				 

				  

				 
				CS
				 

				  

				 
				0.235
				 

				  

				 
				0.047
				 

				  

				 
				5.001
				 

				  

				 
				< .001
				 

				  

				 
				0.138
				 

				  

				 
				0.350
				 

				  
			 

			 
				 
				LOT
				 

				  

				 
				↔
				 

				  

				 
				CS
				 

				  

				 
				0.163
				 

				  

				 
				0.046
				 

				  

				 
				3.562
				 

				  

				 
				< .001
				 

				  

				 
				0.074
				 

				  

				 
				0.264
				 

				  
			 

			 
				 
				SC
				 

				  

				 
				↔
				 

				  

				 
				CS
				 

				  

				 
				0.190
				 

				  

				 
				0.045
				 

				  

				 
				4.248
				 

				  

				 
				< .001
				 

				  

				 
				0.097
				 

				  

				 
				0.309
				 

				  
			 

			 
				 
				SE
				 

				  

				 
				↔
				 

				  

				 
				BU
				 

				  

				 
				-0.207
				 

				  

				 
				0.043
				 

				  

				 
				-4.802
				 

				  

				 
				< .001
				 

				  

				 
				-0.307
				 

				  

				 
				-0.130
				 

				  
			 

			 
				 
				LOT
				 

				  

				 
				↔
				 

				  

				 
				BU
				 

				  

				 
				-0.243
				 

				  

				 
				0.044
				 

				  

				 
				-5.572
				 

				  

				 
				< .001
				 

				  

				 
				-0.360
				 

				  

				 
				-0.154
				 

				  
			 

			 
				 
				SC
				 

				  

				 
				↔
				 

				  

				 
				BU
				 

				  

				 
				-0.261
				 

				  

				 
				0.043
				 

				  

				 
				-6.132
				 

				  

				 
				< .001
				 

				  

				 
				-0.369
				 

				  

				 
				-0.177
				 

				  
			 

			 
				 
				CS
				 

				  

				 
				↔
				 

				  

				 
				BU
				 

				  

				 
				-0.491
				 

				  

				 
				0.052
				 

				  

				 
				-9.436
				 

				  

				 
				< .001
				 

				  

				 
				-0.619
				 

				  

				 
				-0.376
				 

				  
			 

			 
				 
				SE
				 

				  

				 
				↔
				 

				  

				 
				STS
				 

				  

				 
				-0.105
				 

				  

				 
				0.043
				 

				  

				 
				-2.411
				 

				  

				 
				0.016
				 

				  

				 
				-0.195
				 

				  

				 
				-0.026
				 

				  
			 

			 
				 
				LOT
				 

				  

				 
				↔
				 

				  

				 
				STS
				 

				  

				 
				-0.007
				 

				  

				 
				0.043
				 

				  

				 
				-0.168
				 

				  

				 
				0.866
				 

				  

				 
				-0.089
				 

				  

				 
				0.072
				 

				  
			 

			 
				 
				SC
				 

				  

				 
				↔
				 

				  

				 
				STS
				 

				  

				 
				-0.068
				 

				  

				 
				0.042
				 

				  

				 
				-1.630
				 

				  

				 
				0.103
				 

				  

				 
				-0.148
				 

				  

				 
				0.015
				 

				  
			 

			 
				 
				CS
				 

				  

				 
				↔
				 

				  

				 
				STS
				 

				  

				 
				-0.005
				 

				  

				 
				0.046
				 

				  

				 
				-0.110
				 

				  

				 
				0.913
				 

				  

				 
				-0.093
				 

				  

				 
				0.096
				 

				  
			 

			 
				 
				BU
				 

				  

				 
				↔
				 

				  

				 
				STS
				 

				  

				 
				0.235
				 

				  

				 
				0.044
				 

				  

				 
				5.310
				 

				  

				 
				< .001
				 

				  

				 
				0.136
				 

				  

				 
				0.347
				 

				  
			 

			 
				  
			 
		 

		 
			 
				 
					 
					Note.
					 

				 Delta method standard errors, bias-corrected percentile bootstrap confidence intervals, ML estimator.
				 
			 
		 
	 
 
     
 
 
	 
		 
			 
				 
					 
						 
							 
							R-Squared
							 

							  

							  
						 
					 
				 
			 

			 
				 
				 
				 

				 
				R²
				 
			 
		 

		 
			 
				 
				RESIL
				 

				  

				 
				0.643
				 

				  

			 

			 
				 
				SE
				 

				  

				 
				0.235
				 

				  
			 

			 
				 
				LOT
				 

				  

				 
				0.242
				 

				  
			 

			 
				 
				SC
				 

				  

				 
				0.291
				 

				  
			 

			 
				 
				CS
				 

				  

				 
				0.128
				 

				  
			 

			 
				 
				BU
				 

				  

				 
				0.261
				 

				  
			 

			 
				 
				STS
				 

				  

				 
				0.207
				 

				  
			 

			 
				  
			 
		 

		  
	 
 
   
 
  Path plot           
 
  SEM. Testing Full model           
 
   
 
 
	 
		 
			 
				 
					 
						 
							 
							Chi Square Test Statistic (unscaled)
							 

							  

							  
						 
					 
				 
			 

			 
				 
				 
				 

				 
				df
				 

				 
				AIC
				 

				 
				BIC
				 

				 
				χ²
				 

				 
				p
				 
			 
		 

		 
			 
				 
				Model
				 

				  

				 
				0.000
				 

				  

				  

				  

				  

				  

				 
				1.165e -13
				 

				  

				  

				  

			 

			 
				  
			 
		 

		  
	 
 
   
 
 
	 
		 
			 
				 
					 
						 
							 
							Parameter Estimates
							 

							  

							  
						 
					 
				 
			 

			 
				 
				 
				 

				 
				 
				 

				 
				 
				 

				 
				label
				 

				 
				est
				 

				 
				se
				 

				 
				z
				 

				 
				p
				 

				 
				CI (lower)
				 

				 
				CI (upper)
				 

				 
				std (lv)
				 

				 
				std (all)
				 

				 
				std (nox)
				 

				 
				group
				 
			 
		 

		 
			 
				 
				RESIL
				 

				  

				 
				~
				 

				  

				 
				SE
				 

				  

				 
				b11
				 

				  

				 
				1.094
				 

				  

				 
				0.135
				 

				  

				 
				8.103
				 

				  

				 
				< .001
				 

				  

				 
				0.825
				 

				  

				 
				1.349
				 

				  

				 
				1.094
				 

				  

				 
				0.364
				 

				  

				 
				0.364
				 

				  

				  

				  

			 

			 
				 
				RESIL
				 

				  

				 
				~
				 

				  

				 
				LOT
				 

				  

				 
				b12
				 

				  

				 
				0.335
				 

				  

				 
				0.113
				 

				  

				 
				2.963
				 

				  

				 
				0.003
				 

				  

				 
				0.119
				 

				  

				 
				0.551
				 

				  

				 
				0.335
				 

				  

				 
				0.147
				 

				  

				 
				0.147
				 

				  

				  

				  
			 

			 
				 
				RESIL
				 

				  

				 
				~
				 

				  

				 
				SC
				 

				  

				 
				b13
				 

				  

				 
				0.249
				 

				  

				 
				0.061
				 

				  

				 
				4.046
				 

				  

				 
				< .001
				 

				  

				 
				0.118
				 

				  

				 
				0.361
				 

				  

				 
				0.249
				 

				  

				 
				0.197
				 

				  

				 
				0.197
				 

				  

				  

				  
			 

			 
				 
				RESIL
				 

				  

				 
				~
				 

				  

				 
				STS
				 

				  

				 
				b14
				 

				  

				 
				0.246
				 

				  

				 
				0.099
				 

				  

				 
				2.488
				 

				  

				 
				0.013
				 

				  

				 
				0.064
				 

				  

				 
				0.446
				 

				  

				 
				0.246
				 

				  

				 
				0.099
				 

				  

				 
				0.099
				 

				  

				  

				  
			 

			 
				 
				RESIL
				 

				  

				 
				~
				 

				  

				 
				BU
				 

				  

				 
				b15
				 

				  

				 
				-0.519
				 

				  

				 
				0.118
				 

				  

				 
				-4.388
				 

				  

				 
				< .001
				 

				  

				 
				-0.760
				 

				  

				 
				-0.292
				 

				  

				 
				-0.519
				 

				  

				 
				-0.227
				 

				  

				 
				-0.227
				 

				  

				  

				  
			 

			 
				 
				RESIL
				 

				  

				 
				~
				 

				  

				 
				CS
				 

				  

				 
				b16
				 

				  

				 
				0.351
				 

				  

				 
				0.098
				 

				  

				 
				3.575
				 

				  

				 
				< .001
				 

				  

				 
				0.150
				 

				  

				 
				0.538
				 

				  

				 
				0.351
				 

				  

				 
				0.158
				 

				  

				 
				0.158
				 

				  

				  

				  
			 

			 
				 
				RESIL
				 

				  

				 
				~
				 

				  

				 
				PSS
				 

				  

				 
				c11
				 

				  

				 
				-0.016
				 

				  

				 
				0.079
				 

				  

				 
				-0.207
				 

				  

				 
				0.836
				 

				  

				 
				-0.175
				 

				  

				 
				0.130
				 

				  

				 
				-0.016
				 

				  

				 
				-0.009
				 

				  

				 
				-0.001
				 

				  

				  

				  
			 

			 
				 
				RESIL
				 

				  

				 
				~
				 

				  

				 
				RSQ
				 

				  

				 
				c12
				 

				  

				 
				0.040
				 

				  

				 
				0.058
				 

				  

				 
				0.682
				 

				  

				 
				0.495
				 

				  

				 
				-0.082
				 

				  

				 
				0.146
				 

				  

				 
				0.040
				 

				  

				 
				0.026
				 

				  

				 
				0.004
				 

				  

				  

				  
			 

			 
				 
				SE
				 

				  

				 
				~
				 

				  

				 
				PSS
				 

				  

				 
				a11
				 

				  

				 
				-0.260
				 

				  

				 
				0.041
				 

				  

				 
				-6.410
				 

				  

				 
				< .001
				 

				  

				 
				-0.347
				 

				  

				 
				-0.183
				 

				  

				 
				-0.260
				 

				  

				 
				-0.420
				 

				  

				 
				-0.070
				 

				  

				  

				  
			 

			 
				 
				SE
				 

				  

				 
				~
				 

				  

				 
				RSQ
				 

				  

				 
				a12
				 

				  

				 
				-0.070
				 

				  

				 
				0.025
				 

				  

				 
				-2.843
				 

				  

				 
				0.004
				 

				  

				 
				-0.117
				 

				  

				 
				-0.025
				 

				  

				 
				-0.070
				 

				  

				 
				-0.137
				 

				  

				 
				-0.019
				 

				  

				  

				  
			 

			 
				 
				LOT
				 

				  

				 
				~
				 

				  

				 
				PSS
				 

				  

				 
				a21
				 

				  

				 
				-0.317
				 

				  

				 
				0.050
				 

				  

				 
				-6.298
				 

				  

				 
				< .001
				 

				  

				 
				-0.413
				 

				  

				 
				-0.222
				 

				  

				 
				-0.317
				 

				  

				 
				-0.387
				 

				  

				 
				-0.064
				 

				  

				  

				  
			 

			 
				 
				LOT
				 

				  

				 
				~
				 

				  

				 
				RSQ
				 

				  

				 
				a22
				 

				  

				 
				-0.133
				 

				  

				 
				0.033
				 

				  

				 
				-4.084
				 

				  

				 
				< .001
				 

				  

				 
				-0.197
				 

				  

				 
				-0.068
				 

				  

				 
				-0.133
				 

				  

				 
				-0.198
				 

				  

				 
				-0.027
				 

				  

				  

				  
			 

			 
				 
				SC
				 

				  

				 
				~
				 

				  

				 
				PSS
				 

				  

				 
				a31
				 

				  

				 
				-0.716
				 

				  

				 
				0.079
				 

				  

				 
				-9.037
				 

				  

				 
				< .001
				 

				  

				 
				-0.885
				 

				  

				 
				-0.563
				 

				  

				 
				-0.716
				 

				  

				 
				-0.486
				 

				  

				 
				-0.081
				 

				  

				  

				  
			 

			 
				 
				SC
				 

				  

				 
				~
				 

				  

				 
				RSQ
				 

				  

				 
				a32
				 

				  

				 
				-0.147
				 

				  

				 
				0.061
				 

				  

				 
				-2.403
				 

				  

				 
				0.016
				 

				  

				 
				-0.264
				 

				  

				 
				-0.023
				 

				  

				 
				-0.147
				 

				  

				 
				-0.121
				 

				  

				 
				-0.017
				 

				  

				  

				  
			 

			 
				 
				STS
				 

				  

				 
				~
				 

				  

				 
				PSS
				 

				  

				 
				a41
				 

				  

				 
				0.247
				 

				  

				 
				0.042
				 

				  

				 
				5.935
				 

				  

				 
				< .001
				 

				  

				 
				0.165
				 

				  

				 
				0.325
				 

				  

				 
				0.247
				 

				  

				 
				0.330
				 

				  

				 
				0.055
				 

				  

				  

				  
			 

			 
				 
				STS
				 

				  

				 
				~
				 

				  

				 
				RSQ
				 

				  

				 
				a42
				 

				  

				 
				0.135
				 

				  

				 
				0.035
				 

				  

				 
				3.873
				 

				  

				 
				< .001
				 

				  

				 
				0.070
				 

				  

				 
				0.207
				 

				  

				 
				0.135
				 

				  

				 
				0.219
				 

				  

				 
				0.030
				 

				  

				  

				  
			 

			 
				 
				BU
				 

				  

				 
				~
				 

				  

				 
				PSS
				 

				  

				 
				a51
				 

				  

				 
				0.302
				 

				  

				 
				0.050
				 

				  

				 
				5.987
				 

				  

				 
				< .001
				 

				  

				 
				0.204
				 

				  

				 
				0.399
				 

				  

				 
				0.302
				 

				  

				 
				0.370
				 

				  

				 
				0.061
				 

				  

				  

				  
			 

			 
				 
				BU
				 

				  

				 
				~
				 

				  

				 
				RSQ
				 

				  

				 
				a52
				 

				  

				 
				0.167
				 

				  

				 
				0.038
				 

				  

				 
				4.400
				 

				  

				 
				< .001
				 

				  

				 
				0.097
				 

				  

				 
				0.250
				 

				  

				 
				0.167
				 

				  

				 
				0.248
				 

				  

				 
				0.034
				 

				  

				  

				  
			 

			 
				 
				CS
				 

				  

				 
				~
				 

				  

				 
				PSS
				 

				  

				 
				a61
				 

				  

				 
				-0.281
				 

				  

				 
				0.055
				 

				  

				 
				-5.131
				 

				  

				 
				< .001
				 

				  

				 
				-0.392
				 

				  

				 
				-0.184
				 

				  

				 
				-0.281
				 

				  

				 
				-0.337
				 

				  

				 
				-0.056
				 

				  

				  

				  
			 

			 
				 
				CS
				 

				  

				 
				~
				 

				  

				 
				RSQ
				 

				  

				 
				a62
				 

				  

				 
				-0.036
				 

				  

				 
				0.037
				 

				  

				 
				-0.964
				 

				  

				 
				0.335
				 

				  

				 
				-0.108
				 

				  

				 
				0.037
				 

				  

				 
				-0.036
				 

				  

				 
				-0.052
				 

				  

				 
				-0.007
				 

				  

				  

				  
			 

			 
				 
				SE
				 

				  

				 
				~~
				 

				  

				 
				LOT
				 

				  

				  

				  

				 
				4.245
				 

				  

				 
				0.857
				 

				  

				 
				4.955
				 

				  

				 
				< .001
				 

				  

				 
				2.525
				 

				  

				 
				5.876
				 

				  

				 
				4.245
				 

				  

				 
				0.303
				 

				  

				 
				0.303
				 

				  

				  

				  
			 

			 
				 
				SE
				 

				  

				 
				~~
				 

				  

				 
				SC
				 

				  

				  

				  

				 
				6.883
				 

				  

				 
				1.519
				 

				  

				 
				4.532
				 

				  

				 
				< .001
				 

				  

				 
				3.936
				 

				  

				 
				9.885
				 

				  

				 
				6.883
				 

				  

				 
				0.282
				 

				  

				 
				0.282
				 

				  

				  

				  
			 

			 
				 
				LOT
				 

				  

				 
				~~
				 

				  

				 
				SC
				 

				  

				  

				  

				 
				12.551
				 

				  

				 
				2.261
				 

				  

				 
				5.552
				 

				  

				 
				< .001
				 

				  

				 
				8.369
				 

				  

				 
				17.310
				 

				  

				 
				12.551
				 

				  

				 
				0.390
				 

				  

				 
				0.390
				 

				  

				  

				  
			 

			 
				 
				SE
				 

				  

				 
				~~
				 

				  

				 
				STS
				 

				  

				  

				  

				 
				-1.764
				 

				  

				 
				0.739
				 

				  

				 
				-2.386
				 

				  

				 
				0.017
				 

				  

				 
				-3.235
				 

				  

				 
				-0.284
				 

				  

				 
				-1.764
				 

				  

				 
				-0.135
				 

				  

				 
				-0.135
				 

				  

				  

				  
			 

			 
				 
				LOT
				 

				  

				 
				~~
				 

				  

				 
				STS
				 

				  

				  

				  

				 
				-0.161
				 

				  

				 
				0.917
				 

				  

				 
				-0.175
				 

				  

				 
				0.861
				 

				  

				 
				-2.093
				 

				  

				 
				1.519
				 

				  

				 
				-0.161
				 

				  

				 
				-0.009
				 

				  

				 
				-0.009
				 

				  

				  

				  
			 

			 
				 
				SC
				 

				  

				 
				~~
				 

				  

				 
				STS
				 

				  

				  

				  

				 
				-2.725
				 

				  

				 
				1.706
				 

				  

				 
				-1.597
				 

				  

				 
				0.110
				 

				  

				 
				-6.035
				 

				  

				 
				0.524
				 

				  

				 
				-2.725
				 

				  

				 
				-0.091
				 

				  

				 
				-0.091
				 

				  

				  

				  
			 

			 
				 
				SE
				 

				  

				 
				~~
				 

				  

				 
				BU
				 

				  

				  

				  

				 
				-3.806
				 

				  

				 
				0.845
				 

				  

				 
				-4.506
				 

				  

				 
				< .001
				 

				  

				 
				-5.542
				 

				  

				 
				-2.126
				 

				  

				 
				-3.806
				 

				  

				 
				-0.276
				 

				  

				 
				-0.276
				 

				  

				  

				  
			 

			 
				 
				LOT
				 

				  

				 
				~~
				 

				  

				 
				BU
				 

				  

				  

				  

				 
				-5.895
				 

				  

				 
				1.299
				 

				  

				 
				-4.539
				 

				  

				 
				< .001
				 

				  

				 
				-8.472
				 

				  

				 
				-3.454
				 

				  

				 
				-5.895
				 

				  

				 
				-0.325
				 

				  

				 
				-0.325
				 

				  

				  

				  
			 

			 
				 
				SC
				 

				  

				 
				~~
				 

				  

				 
				BU
				 

				  

				  

				  

				 
				-11.440
				 

				  

				 
				2.035
				 

				  

				 
				-5.622
				 

				  

				 
				< .001
				 

				  

				 
				-15.242
				 

				  

				 
				-7.520
				 

				  

				 
				-11.440
				 

				  

				 
				-0.362
				 

				  

				 
				-0.362
				 

				  

				  

				  
			 

			 
				 
				STS
				 

				  

				 
				~~
				 

				  

				 
				BU
				 

				  

				  

				  

				 
				5.219
				 

				  

				 
				1.204
				 

				  

				 
				4.336
				 

				  

				 
				< .001
				 

				  

				 
				2.905
				 

				  

				 
				7.596
				 

				  

				 
				5.219
				 

				  

				 
				0.308
				 

				  

				 
				0.308
				 

				  

				  

				  
			 

			 
				 
				SE
				 

				  

				 
				~~
				 

				  

				 
				CS
				 

				  

				  

				  

				 
				4.424
				 

				  

				 
				1.013
				 

				  

				 
				4.368
				 

				  

				 
				< .001
				 

				  

				 
				2.379
				 

				  

				 
				6.490
				 

				  

				 
				4.424
				 

				  

				 
				0.289
				 

				  

				 
				0.289
				 

				  

				  

				  
			 

			 
				 
				LOT
				 

				  

				 
				~~
				 

				  

				 
				CS
				 

				  

				  

				  

				 
				4.068
				 

				  

				 
				1.309
				 

				  

				 
				3.106
				 

				  

				 
				0.002
				 

				  

				 
				1.533
				 

				  

				 
				6.649
				 

				  

				 
				4.068
				 

				  

				 
				0.202
				 

				  

				 
				0.202
				 

				  

				  

				  
			 

			 
				 
				SC
				 

				  

				 
				~~
				 

				  

				 
				CS
				 

				  

				  

				  

				 
				8.532
				 

				  

				 
				2.199
				 

				  

				 
				3.879
				 

				  

				 
				< .001
				 

				  

				 
				4.167
				 

				  

				 
				12.898
				 

				  

				 
				8.532
				 

				  

				 
				0.242
				 

				  

				 
				0.242
				 

				  

				  

				  
			 

			 
				 
				STS
				 

				  

				 
				~~
				 

				  

				 
				CS
				 

				  

				  

				  

				 
				-0.114
				 

				  

				 
				1.124
				 

				  

				 
				-0.102
				 

				  

				 
				0.919
				 

				  

				 
				-2.352
				 

				  

				 
				2.221
				 

				  

				 
				-0.114
				 

				  

				 
				-0.006
				 

				  

				 
				-0.006
				 

				  

				  

				  
			 

			 
				 
				BU
				 

				  

				 
				~~
				 

				  

				 
				CS
				 

				  

				  

				  

				 
				-12.195
				 

				  

				 
				1.541
				 

				  

				 
				-7.913
				 

				  

				 
				< .001
				 

				  

				 
				-15.401
				 

				  

				 
				-9.148
				 

				  

				 
				-12.195
				 

				  

				 
				-0.614
				 

				  

				 
				-0.614
				 

				  

				  

				  
			 

			 
				 
				RESIL
				 

				  

				 
				~~
				 

				  

				 
				RESIL
				 

				  

				  

				  

				 
				44.896
				 

				  

				 
				3.770
				 

				  

				 
				11.910
				 

				  

				 
				< .001
				 

				  

				 
				36.517
				 

				  

				 
				51.390
				 

				  

				 
				44.896
				 

				  

				 
				0.357
				 

				  

				 
				0.357
				 

				  

				  

				  
			 

			 
				 
				SE
				 

				  

				 
				~~
				 

				  

				 
				SE
				 

				  

				  

				  

				 
				10.629
				 

				  

				 
				0.787
				 

				  

				 
				13.502
				 

				  

				 
				< .001
				 

				  

				 
				8.954
				 

				  

				 
				12.134
				 

				  

				 
				10.629
				 

				  

				 
				0.765
				 

				  

				 
				0.765
				 

				  

				  

				  
			 

			 
				 
				LOT
				 

				  

				 
				~~
				 

				  

				 
				LOT
				 

				  

				  

				  

				 
				18.437
				 

				  

				 
				1.529
				 

				  

				 
				12.057
				 

				  

				 
				< .001
				 

				  

				 
				15.313
				 

				  

				 
				21.471
				 

				  

				 
				18.437
				 

				  

				 
				0.758
				 

				  

				 
				0.758
				 

				  

				  

				  
			 

			 
				 
				SC
				 

				  

				 
				~~
				 

				  

				 
				SC
				 

				  

				  

				  

				 
				56.049
				 

				  

				 
				4.410
				 

				  

				 
				12.709
				 

				  

				 
				< .001
				 

				  

				 
				47.024
				 

				  

				 
				64.590
				 

				  

				 
				56.049
				 

				  

				 
				0.709
				 

				  

				 
				0.709
				 

				  

				  

				  
			 

			 
				 
				STS
				 

				  

				 
				~~
				 

				  

				 
				STS
				 

				  

				  

				  

				 
				16.069
				 

				  

				 
				1.472
				 

				  

				 
				10.915
				 

				  

				 
				< .001
				 

				  

				 
				13.198
				 

				  

				 
				18.755
				 

				  

				 
				16.069
				 

				  

				 
				0.793
				 

				  

				 
				0.793
				 

				  

				  

				  
			 

			 
				 
				BU
				 

				  

				 
				~~
				 

				  

				 
				BU
				 

				  

				  

				  

				 
				17.844
				 

				  

				 
				1.541
				 

				  

				 
				11.577
				 

				  

				 
				< .001
				 

				  

				 
				14.799
				 

				  

				 
				20.665
				 

				  

				 
				17.844
				 

				  

				 
				0.739
				 

				  

				 
				0.739
				 

				  

				  

				  
			 

			 
				 
				CS
				 

				  

				 
				~~
				 

				  

				 
				CS
				 

				  

				  

				  

				 
				22.087
				 

				  

				 
				2.017
				 

				  

				 
				10.950
				 

				  

				 
				< .001
				 

				  

				 
				18.101
				 

				  

				 
				26.061
				 

				  

				 
				22.087
				 

				  

				 
				0.872
				 

				  

				 
				0.872
				 

				  

				  

				  
			 

			 
				 
				PSS
				 

				  

				 
				~~
				 

				  

				 
				PSS
				 

				  

				  

				  

				 
				36.365
				 

				  

				 
				0.000
				 

				  

				  

				  

				 
				 
				 

				  

				 
				36.365
				 

				  

				 
				36.365
				 

				  

				 
				36.365
				 

				  

				 
				1.000
				 

				  

				 
				36.365
				 

				  

				  

				  
			 

			 
				 
				PSS
				 

				  

				 
				~~
				 

				  

				 
				RSQ
				 

				  

				  

				  

				 
				15.159
				 

				  

				 
				0.000
				 

				  

				  

				  

				 
				 
				 

				  

				 
				15.159
				 

				  

				 
				15.159
				 

				  

				 
				15.159
				 

				  

				 
				0.344
				 

				  

				 
				15.159
				 

				  

				  

				  
			 

			 
				 
				RSQ
				 

				  

				 
				~~
				 

				  

				 
				RSQ
				 

				  

				  

				  

				 
				53.394
				 

				  

				 
				0.000
				 

				  

				  

				  

				 
				 
				 

				  

				 
				53.394
				 

				  

				 
				53.394
				 

				  

				 
				53.394
				 

				  

				 
				1.000
				 

				  

				 
				53.394
				 

				  

				  

				  
			 

			 
				 
				RESIL
				 

				  

				 
				~1
				 

				  

				  

				  

				  

				  

				 
				-0.022
				 

				  

				 
				0.377
				 

				  

				 
				-0.058
				 

				  

				 
				0.954
				 

				  

				 
				-0.821
				 

				  

				 
				0.681
				 

				  

				 
				-0.022
				 

				  

				 
				-0.002
				 

				  

				 
				-0.002
				 

				  

				  

				  
			 

			 
				 
				SE
				 

				  

				 
				~1
				 

				  

				  

				  

				  

				  

				 
				0.020
				 

				  

				 
				0.178
				 

				  

				 
				0.110
				 

				  

				 
				0.913
				 

				  

				 
				-0.357
				 

				  

				 
				0.371
				 

				  

				 
				0.020
				 

				  

				 
				0.005
				 

				  

				 
				0.005
				 

				  

				  

				  
			 

			 
				 
				LOT
				 

				  

				 
				~1
				 

				  

				  

				  

				  

				  

				 
				0.008
				 

				  

				 
				0.241
				 

				  

				 
				0.032
				 

				  

				 
				0.974
				 

				  

				 
				-0.437
				 

				  

				 
				0.502
				 

				  

				 
				0.008
				 

				  

				 
				0.002
				 

				  

				 
				0.002
				 

				  

				  

				  
			 

			 
				 
				SC
				 

				  

				 
				~1
				 

				  

				  

				  

				  

				  

				 
				0.009
				 

				  

				 
				0.423
				 

				  

				 
				0.021
				 

				  

				 
				0.983
				 

				  

				 
				-0.818
				 

				  

				 
				0.834
				 

				  

				 
				0.009
				 

				  

				 
				9.924e -4
				 

				  

				 
				9.924e -4
				 

				  

				  

				  
			 

			 
				 
				STS
				 

				  

				 
				~1
				 

				  

				  

				  

				  

				  

				 
				0.006
				 

				  

				 
				0.218
				 

				  

				 
				0.028
				 

				  

				 
				0.977
				 

				  

				 
				-0.398
				 

				  

				 
				0.472
				 

				  

				 
				0.006
				 

				  

				 
				0.001
				 

				  

				 
				0.001
				 

				  

				  

				  
			 

			 
				 
				BU
				 

				  

				 
				~1
				 

				  

				  

				  

				  

				  

				 
				0.002
				 

				  

				 
				0.232
				 

				  

				 
				0.009
				 

				  

				 
				0.993
				 

				  

				 
				-0.422
				 

				  

				 
				0.472
				 

				  

				 
				0.002
				 

				  

				 
				4.063e -4
				 

				  

				 
				4.063e -4
				 

				  

				  

				  
			 

			 
				 
				CS
				 

				  

				 
				~1
				 

				  

				  

				  

				  

				  

				 
				0.006
				 

				  

				 
				0.260
				 

				  

				 
				0.024
				 

				  

				 
				0.981
				 

				  

				 
				-0.528
				 

				  

				 
				0.518
				 

				  

				 
				0.006
				 

				  

				 
				0.001
				 

				  

				 
				0.001
				 

				  

				  

				  
			 

			 
				 
				PSS
				 

				  

				 
				~1
				 

				  

				  

				  

				  

				  

				 
				0.002
				 

				  

				 
				0.000
				 

				  

				  

				  

				 
				 
				 

				  

				 
				0.002
				 

				  

				 
				0.002
				 

				  

				 
				0.002
				 

				  

				 
				3.827e -4
				 

				  

				 
				0.002
				 

				  

				  

				  
			 

			 
				 
				RSQ
				 

				  

				 
				~1
				 

				  

				  

				  

				  

				  

				 
				0.007
				 

				  

				 
				0.000
				 

				  

				  

				  

				 
				 
				 

				  

				 
				0.007
				 

				  

				 
				0.007
				 

				  

				 
				0.007
				 

				  

				 
				9.474e -4
				 

				  

				 
				0.007
				 

				  

				  

				  
			 

			 
				 
				ind_x1_m1_y1
				 

				  

				 
				:=
				 

				  

				 
				a11*b11
				 

				  

				 
				ind_x1_m1_y1
				 

				  

				 
				-0.284
				 

				  

				 
				0.056
				 

				  

				 
				-5.074
				 

				  

				 
				< .001
				 

				  

				 
				-0.399
				 

				  

				 
				-0.182
				 

				  

				 
				-0.284
				 

				  

				 
				-0.153
				 

				  

				 
				-0.025
				 

				  

				  

				  
			 

			 
				 
				ind_x1_m2_y1
				 

				  

				 
				:=
				 

				  

				 
				a21*b12
				 

				  

				 
				ind_x1_m2_y1
				 

				  

				 
				-0.106
				 

				  

				 
				0.040
				 

				  

				 
				-2.660
				 

				  

				 
				0.008
				 

				  

				 
				-0.190
				 

				  

				 
				-0.037
				 

				  

				 
				-0.106
				 

				  

				 
				-0.057
				 

				  

				 
				-0.009
				 

				  

				  

				  
			 

			 
				 
				ind_x1_m3_y1
				 

				  

				 
				:=
				 

				  

				 
				a31*b13
				 

				  

				 
				ind_x1_m3_y1
				 

				  

				 
				-0.178
				 

				  

				 
				0.048
				 

				  

				 
				-3.684
				 

				  

				 
				< .001
				 

				  

				 
				-0.274
				 

				  

				 
				-0.087
				 

				  

				 
				-0.178
				 

				  

				 
				-0.096
				 

				  

				 
				-0.016
				 

				  

				  

				  
			 

			 
				 
				ind_x1_m4_y1
				 

				  

				 
				:=
				 

				  

				 
				a41*b14
				 

				  

				 
				ind_x1_m4_y1
				 

				  

				 
				0.061
				 

				  

				 
				0.026
				 

				  

				 
				2.314
				 

				  

				 
				0.021
				 

				  

				 
				0.015
				 

				  

				 
				0.114
				 

				  

				 
				0.061
				 

				  

				 
				0.033
				 

				  

				 
				0.005
				 

				  

				  

				  
			 

			 
				 
				ind_x1_m5_y1
				 

				  

				 
				:=
				 

				  

				 
				a51*b15
				 

				  

				 
				ind_x1_m5_y1
				 

				  

				 
				-0.156
				 

				  

				 
				0.045
				 

				  

				 
				-3.465
				 

				  

				 
				< .001
				 

				  

				 
				-0.250
				 

				  

				 
				-0.077
				 

				  

				 
				-0.156
				 

				  

				 
				-0.084
				 

				  

				 
				-0.014
				 

				  

				  

				  
			 

			 
				 
				ind_x1_m6_y1
				 

				  

				 
				:=
				 

				  

				 
				a61*b16
				 

				  

				 
				ind_x1_m6_y1
				 

				  

				 
				-0.099
				 

				  

				 
				0.033
				 

				  

				 
				-2.966
				 

				  

				 
				0.003
				 

				  

				 
				-0.169
				 

				  

				 
				-0.040
				 

				  

				 
				-0.099
				 

				  

				 
				-0.053
				 

				  

				 
				-0.009
				 

				  

				  

				  
			 

			 
				 
				ind_x1_y1
				 

				  

				 
				:=
				 

				  

				 
				ind_x1_m1_y1+ind_x1_m2_y1+ind_x1_m3_y1+ind_x1_m4_y1+ind_x1_m5_y1+ind_x1_m6_y1
				 

				  

				 
				ind_x1_y1
				 

				  

				 
				-0.763
				 

				  

				 
				0.107
				 

				  

				 
				-7.134
				 

				  

				 
				< .001
				 

				  

				 
				-0.986
				 

				  

				 
				-0.556
				 

				  

				 
				-0.763
				 

				  

				 
				-0.410
				 

				  

				 
				-0.068
				 

				  

				  

				  
			 

			 
				 
				tot_x1_y1
				 

				  

				 
				:=
				 

				  

				 
				ind_x1_y1+c11
				 

				  

				 
				tot_x1_y1
				 

				  

				 
				-0.779
				 

				  

				 
				0.122
				 

				  

				 
				-6.397
				 

				  

				 
				< .001
				 

				  

				 
				-1.030
				 

				  

				 
				-0.547
				 

				  

				 
				-0.779
				 

				  

				 
				-0.419
				 

				  

				 
				-0.069
				 

				  

				  

				  
			 

			 
				 
				ind_x2_m1_y1
				 

				  

				 
				:=
				 

				  

				 
				a12*b11
				 

				  

				 
				ind_x2_m1_y1
				 

				  

				 
				-0.077
				 

				  

				 
				0.029
				 

				  

				 
				-2.685
				 

				  

				 
				0.007
				 

				  

				 
				-0.135
				 

				  

				 
				-0.025
				 

				  

				 
				-0.077
				 

				  

				 
				-0.050
				 

				  

				 
				-0.007
				 

				  

				  

				  
			 

			 
				 
				ind_x2_m2_y1
				 

				  

				 
				:=
				 

				  

				 
				a22*b12
				 

				  

				 
				ind_x2_m2_y1
				 

				  

				 
				-0.045
				 

				  

				 
				0.019
				 

				  

				 
				-2.346
				 

				  

				 
				0.019
				 

				  

				 
				-0.087
				 

				  

				 
				-0.014
				 

				  

				 
				-0.045
				 

				  

				 
				-0.029
				 

				  

				 
				-0.004
				 

				  

				  

				  
			 

			 
				 
				ind_x2_m3_y1
				 

				  

				 
				:=
				 

				  

				 
				a32*b13
				 

				  

				 
				ind_x2_m3_y1
				 

				  

				 
				-0.037
				 

				  

				 
				0.017
				 

				  

				 
				-2.144
				 

				  

				 
				0.032
				 

				  

				 
				-0.074
				 

				  

				 
				-0.005
				 

				  

				 
				-0.037
				 

				  

				 
				-0.024
				 

				  

				 
				-0.003
				 

				  

				  

				  
			 

			 
				 
				ind_x2_m4_y1
				 

				  

				 
				:=
				 

				  

				 
				a42*b14
				 

				  

				 
				ind_x2_m4_y1
				 

				  

				 
				0.033
				 

				  

				 
				0.017
				 

				  

				 
				1.931
				 

				  

				 
				0.053
				 

				  

				 
				0.006
				 

				  

				 
				0.072
				 

				  

				 
				0.033
				 

				  

				 
				0.022
				 

				  

				 
				0.003
				 

				  

				  

				  
			 

			 
				 
				ind_x2_m5_y1
				 

				  

				 
				:=
				 

				  

				 
				a52*b15
				 

				  

				 
				ind_x2_m5_y1
				 

				  

				 
				-0.086
				 

				  

				 
				0.028
				 

				  

				 
				-3.095
				 

				  

				 
				0.002
				 

				  

				 
				-0.147
				 

				  

				 
				-0.039
				 

				  

				 
				-0.086
				 

				  

				 
				-0.056
				 

				  

				 
				-0.008
				 

				  

				  

				  
			 

			 
				 
				ind_x2_m6_y1
				 

				  

				 
				:=
				 

				  

				 
				a62*b16
				 

				  

				 
				ind_x2_m6_y1
				 

				  

				 
				-0.013
				 

				  

				 
				0.014
				 

				  

				 
				-0.927
				 

				  

				 
				0.354
				 

				  

				 
				-0.039
				 

				  

				 
				0.014
				 

				  

				 
				-0.013
				 

				  

				 
				-0.008
				 

				  

				 
				-0.001
				 

				  

				  

				  
			 

			 
				 
				ind_x2_y1
				 

				  

				 
				:=
				 

				  

				 
				ind_x2_m1_y1+ind_x2_m2_y1+ind_x2_m3_y1+ind_x2_m4_y1+ind_x2_m5_y1+ind_x2_m6_y1
				 

				  

				 
				ind_x2_y1
				 

				  

				 
				-0.224
				 

				  

				 
				0.063
				 

				  

				 
				-3.524
				 

				  

				 
				< .001
				 

				  

				 
				-0.349
				 

				  

				 
				-0.101
				 

				  

				 
				-0.224
				 

				  

				 
				-0.146
				 

				  

				 
				-0.020
				 

				  

				  

				  
			 

			 
				 
				tot_x2_y1
				 

				  

				 
				:=
				 

				  

				 
				ind_x2_y1+c12
				 

				  

				 
				tot_x2_y1
				 

				  

				 
				-0.184
				 

				  

				 
				0.077
				 

				  

				 
				-2.384
				 

				  

				 
				0.017
				 

				  

				 
				-0.339
				 

				  

				 
				-0.044
				 

				  

				 
				-0.184
				 

				  

				 
				-0.120
				 

				  

				 
				-0.016
				 

				  

				  

				  
			 

			 
				  
			 
		 

		  
	 
 
   
 
   
 
 
	 
		 
			 
				 
					 
						 
							 
							Model test baseline model
							 

							  

							  
						 
					 
				 
			 

			 
				 
				 
				 

				 
				Model
				 
			 
		 

		 
			 
				 
				Minimum Function Test Statistic
				 

				  

				 
				1.792e -16
				 

				  

			 

			 
				 
				χ²
				 

				  

				 
				1.165e -13
				 

				  
			 

			 
				 
				Degrees of freedom
				 

				  

				 
				0.000
				 

				  
			 

			 
				 
				p
				 

				  

				 
				 
				 

				  
			 

			 
				  
			 
		 

		  
	 
 
   
 
 
	 
		 
			 
				 
					 
						 
							 
							User model versus baseline model
							 

							  

							  
						 
					 
				 
			 

			 
				 
				 
				 

				 
				Model
				 
			 
		 

		 
			 
				 
				Comparative Fit Index (CFI)
				 

				  

				 
				1.000
				 

				  

			 

			 
				 
				Tucker-Lewis Index (TLI)
				 

				  

				 
				1.000
				 

				  
			 

			 
				 
				Bentler-Bonett Non-normed Fit Index (NNFI)
				 

				  

				 
				1.000
				 

				  
			 

			 
				 
				Bentler-Bonett Normed Fit Index (NFI)
				 

				  

				 
				1.000
				 

				  
			 

			 
				 
				Parsimony Normed Fit Index (PNFI)
				 

				  

				 
				0.000
				 

				  
			 

			 
				 
				Bollen's Relative Fit Index (RFI)
				 

				  

				 
				1.000
				 

				  
			 

			 
				 
				Bollen's Incremental Fit Index (IFI)
				 

				  

				 
				1.000
				 

				  
			 

			 
				 
				Relative Noncentrality Index (RNI)
				 

				  

				 
				1.000
				 

				  
			 

			 
				  
			 
		 

		  
	 
 
  This does not look good. Rather indicates that the model is overfitted     
 
 
	 
		 
			 
				 
					 
						 
							 
							Loglikelihood and Information Criteria
							 

							  

							  
						 
					 
				 
			 

			 
				 
				 
				 

				 
				Model
				 
			 
		 

		 
			 
				 
				Loglikelihood user model (H0)
				 

				  

				  

				  

			 

			 
				 
				Loglikelihood unrestricted model (H1)
				 

				  

				  

				  
			 

			 
				 
				Number of free parameters
				 

				  

				 
				49
				 

				  
			 

			 
				 
				Akaike (AIC)
				 

				  

				  

				  
			 

			 
				 
				Bayesian (BIC)
				 

				  

				  

				  
			 

			 
				 
				Sample-size adjusted Bayesian (BIC)
				 

				  

				  

				  
			 

			 
				 
				NA
				 

				  

				  

				  
			 

			 
				  
			 
		 

		  
	 
 
  Information criteria could not be estimated, possibly too big     
 
 
	 
		 
			 
				 
					 
						 
							 
							Root Mean Square Error of Approximation
							 

							  

							  
						 
					 
				 
			 

			 
				 
				 
				 

				 
				Model
				 
			 
		 

		 
			 
				 
				RMSEA
				 

				  

				 
				0.000
				 

				  

			 

			 
				 
				Upper 90% CI
				 

				  

				 
				0.000
				 

				  
			 

			 
				 
				Lower 90% CI
				 

				  

				 
				0.000
				 

				  
			 

			 
				 
				p-value RMSEA <= 0.05 
				 

				  

				 
				 
				 

				  
			 

			 
				  
			 
		 

		  
	 
 
  RMSEA penalizes for model complexity, but could not be estimated        
 
 
	 
		 
			 
				 
					 
						 
							 
							Standardized Root Mean Square Residual
							 

							  

							  
						 
					 
				 
			 

			 
				 
				 
				 

				 
				Model
				 
			 
		 

		 
			 
				 
				RMR
				 

				  

				 
				1.208e -7
				 

				  

			 

			 
				 
				RMR (No Mean)
				 

				  

				 
				1.324e -7
				 

				  
			 

			 
				 
				SRMR
				 

				  

				 
				3.135e -9
				 

				  
			 

			 
				  
			 
		 

		  
	 
 
   
 
 
	 
		 
			 
				 
					 
						 
							 
							Other Fit Indices
							 

							  

							  
						 
					 
				 
			 

			 
				 
				 
				 

				 
				Model
				 
			 
		 

		 
			 
				 
				Hoelter Critical N (CN) alpha=0.05
				 

				  

				 
				1.000
				 

				  

			 

			 
				 
				Hoelter Critical N (CN) alpha=0.01
				 

				  

				 
				1.000
				 

				  
			 

			 
				 
				Goodness of Fit Index (GFI)
				 

				  

				 
				1.000
				 

				  
			 

			 
				 
				Parsimony Goodness of Fit Index (GFI)
				 

				  

				 
				1.000
				 

				  
			 

			 
				 
				McDonald Fit Index (MFI)
				 

				  

				 
				1.000
				 

				  
			 

			 
				  
			 
		 

		  
	 
 
     
 
 
	 
		 
			 
				 
					 
						 
							 
							R-Squared
							 

							  

							  
						 
					 
				 
			 

			 
				 
				Variable
				 

				 
				R²
				 
			 
		 

		 
			 
				 
				RESIL
				 

				  

				 
				0.643
				 

				  

			 

			 
				 
				SE
				 

				  

				 
				0.235
				 

				  
			 

			 
				 
				LOT
				 

				  

				 
				0.242
				 

				  
			 

			 
				 
				SC
				 

				  

				 
				0.291
				 

				  
			 

			 
				 
				STS
				 

				  

				 
				0.207
				 

				  
			 

			 
				 
				BU
				 

				  

				 
				0.261
				 

				  
			 

			 
				 
				CS
				 

				  

				 
				0.128
				 

				  
			 

			 
				  
			 
		 

		  
	 
 
   
 
   
 
 
	 
		 
			 
				 
					 
						 
							 
							Covariances (lower triangle) / correlations (upper triangle)
							 

							  

							  
						 
					 
				 
			 

			 
				 
				 
				 

				 
				 
				 

				 
				RESIL
				 

				 
				SE
				 

				 
				LOT
				 

				 
				SC
				 

				 
				STS
				 

				 
				BU
				 

				 
				CS
				 

				 
				PSS
				 

				 
				RSQ
				 
			 
		 

		 
			 
				 
				RESIL
				 

				  

				 
				observed
				 

				  

				 
				125.766
				 

				  

				 
				0.657
				 

				  

				 
				0.563
				 

				  

				 
				0.596
				 

				  

				 
				-0.239
				 

				  

				 
				-0.621
				 

				  

				 
				0.565
				 

				  

				 
				-0.460
				 

				  

				 
				-0.264
				 

				  

			 

			 
				 
				 
				 

				  

				 
				fitted
				 

				  

				 
				125.766
				 

				  

				 
				0.657
				 

				  

				 
				0.563
				 

				  

				 
				0.596
				 

				  

				 
				-0.239
				 

				  

				 
				-0.621
				 

				  

				 
				0.565
				 

				  

				 
				-0.460
				 

				  

				 
				-0.264
				 

				  
			 

			 
				 
				 
				 

				  

				 
				residual
				 

				  

				 
				3.160e -7
				 

				  

				 
				2.743e -10
				 

				  

				 
				1.789e 


				-9
				 

				  

				 
				1.185e 


				-9
				 

				  

				 
				7.694e 


				-9
				 

				  

				 
				3.424e -10
				 

				  

				 
				1.271e 


				-9
				 

				  

				 
				4.367e -10
				 

				  

				 
				3.566e -10
				 

				  
			 

			 
				 
				SE
				 

				  

				 
				observed
				 

				  

				 
				27.457
				 

				  

				 
				13.897
				 

				  

				 
				0.468
				 

				  

				 
				0.469
				 

				  

				 
				-0.321
				 

				  

				 
				-0.451
				 

				  

				 
				0.408
				 

				  

				 
				-0.467
				 

				  

				 
				-0.282
				 

				  
			 

			 
				 
				 
				 

				  

				 
				fitted
				 

				  

				 
				27.457
				 

				  

				 
				13.897
				 

				  

				 
				0.468
				 

				  

				 
				0.469
				 

				  

				 
				-0.321
				 

				  

				 
				-0.451
				 

				  

				 
				0.408
				 

				  

				 
				-0.467
				 

				  

				 
				-0.282
				 

				  
			 

			 
				 
				 
				 

				  

				 
				residual
				 

				  

				 
				5.995e -8
				 

				  

				 
				1.416e 


				-8
				 

				  

				 
				2.250e 


				-9
				 

				  

				 
				2.940e -10
				 

				  

				 
				4.458e 


				-9
				 

				  

				 
				2.532e -10
				 

				  

				 
				2.980e -10
				 

				  

				 
				1.236e -10
				 

				  

				 
				1.325e -10
				 

				  
			 

			 
				 
				LOT
				 

				  

				 
				observed
				 

				  

				 
				31.121
				 

				  

				 
				8.597
				 

				  

				 
				24.312
				 

				  

				 
				0.547
				 

				  

				 
				-0.230
				 

				  

				 
				-0.494
				 

				  

				 
				0.334
				 

				  

				 
				-0.455
				 

				  

				 
				-0.331
				 

				  
			 

			 
				 
				 
				 

				  

				 
				fitted
				 

				  

				 
				31.121
				 

				  

				 
				8.597
				 

				  

				 
				24.312
				 

				  

				 
				0.547
				 

				  

				 
				-0.230
				 

				  

				 
				-0.494
				 

				  

				 
				0.334
				 

				  

				 
				-0.455
				 

				  

				 
				-0.331
				 

				  
			 

			 
				 
				 
				 

				  

				 
				residual
				 

				  

				 
				1.901e -7
				 

				  

				 
				6.012e 


				-8
				 

				  

				 
				8.137e 


				-8
				 

				  

				 
				1.813e 


				-9
				 

				  

				 
				7.208e 


				-9
				 

				  

				 
				1.629e -10
				 

				  

				 
				2.275e 


				-9
				 

				  

				 
				6.444e -10
				 

				  

				 
				5.266e -10
				 

				  
			 

			 
				 
				SC
				 

				  

				 
				observed
				 

				  

				 
				59.401
				 

				  

				 
				15.539
				 

				  

				 
				23.998
				 

				  

				 
				79.054
				 

				  

				 
				-0.305
				 

				  

				 
				-0.528
				 

				  

				 
				0.383
				 

				  

				 
				-0.527
				 

				  

				 
				-0.288
				 

				  
			 

			 
				 
				 
				 

				  

				 
				fitted
				 

				  

				 
				59.401
				 

				  

				 
				15.539
				 

				  

				 
				23.998
				 

				  

				 
				79.054
				 

				  

				 
				-0.305
				 

				  

				 
				-0.528
				 

				  

				 
				0.383
				 

				  

				 
				-0.527
				 

				  

				 
				-0.288
				 

				  
			 

			 
				 
				 
				 

				  

				 
				residual
				 

				  

				 
				2.124e -7
				 

				  

				 
				2.279e 


				-8
				 

				  

				 
				1.276e 


				-7
				 

				  

				 
				5.222e 


				-8
				 

				  

				 
				1.180e 


				-8
				 

				  

				 
				1.378e 


				-9
				 

				  

				 
				3.264e 


				-9
				 

				  

				 
				-4.606e -12
				 

				  

				 
				1.034e -10
				 

				  
			 

			 
				 
				STS
				 

				  

				 
				observed
				 

				  

				 
				-12.053
				 

				  

				 
				-5.392
				 

				  

				 
				-5.108
				 

				  

				 
				-12.223
				 

				  

				 
				20.262
				 

				  

				 
				0.468
				 

				  

				 
				-0.159
				 

				  

				 
				0.406
				 

				  

				 
				0.333
				 

				  
			 

			 
				 
				 
				 

				  

				 
				fitted
				 

				  

				 
				-12.053
				 

				  

				 
				-5.392
				 

				  

				 
				-5.108
				 

				  

				 
				-12.223
				 

				  

				 
				20.262
				 

				  

				 
				0.468
				 

				  

				 
				-0.159
				 

				  

				 
				0.406
				 

				  

				 
				0.333
				 

				  
			 

			 
				 
				 
				 

				  

				 
				residual
				 

				  

				 
				3.973e -7
				 

				  

				 
				8.281e 


				-8
				 

				  

				 
				1.616e 


				-7
				 

				  

				 
				4.926e 


				-7
				 

				  

				 
				-8.076e 


				-8
				 

				  

				 
				-6.904e 


				-9
				 

				  

				 
				5.959e 


				-9
				 

				  

				 
				8.310e -10
				 

				  

				 
				5.923e -10
				 

				  
			 

			 
				 
				BU
				 

				  

				 
				observed
				 

				  

				 
				-34.226
				 

				  

				 
				-8.254
				 

				  

				 
				-11.962
				 

				  

				 
				-23.082
				 

				  

				 
				10.363
				 

				  

				 
				24.156
				 

				  

				 
				-0.666
				 

				  

				 
				0.455
				 

				  

				 
				0.375
				 

				  
			 

			 
				 
				 
				 

				  

				 
				fitted
				 

				  

				 
				-34.226
				 

				  

				 
				-8.254
				 

				  

				 
				-11.962
				 

				  

				 
				-23.082
				 

				  

				 
				10.363
				 

				  

				 
				24.156
				 

				  

				 
				-0.666
				 

				  

				 
				0.455
				 

				  

				 
				0.375
				 

				  
			 

			 
				 
				 
				 

				  

				 
				residual
				 

				  

				 
				3.387e -8
				 

				  

				 
				1.442e 


				-8
				 

				  

				 
				4.199e 


				-9
				 

				  

				 
				9.173e 


				-8
				 

				  

				 
				-1.910e 


				-7
				 

				  

				 
				-8.187e 


				-8
				 

				  

				 
				-6.718e -10
				 

				  

				 
				9.669e -10
				 

				  

				 
				6.030e -10
				 

				  
			 

			 
				 
				CS
				 

				  

				 
				observed
				 

				  

				 
				31.896
				 

				  

				 
				7.652
				 

				  

				 
				8.298
				 

				  

				 
				17.147
				 

				  

				 
				-3.601
				 

				  

				 
				-16.468
				 

				  

				 
				25.331
				 

				  

				 
				-0.355
				 

				  

				 
				-0.168
				 

				  
			 

			 
				 
				 
				 

				  

				 
				fitted
				 

				  

				 
				31.896
				 

				  

				 
				7.652
				 

				  

				 
				8.298
				 

				  

				 
				17.147
				 

				  

				 
				-3.601
				 

				  

				 
				-16.468
				 

				  

				 
				25.331
				 

				  

				 
				-0.355
				 

				  

				 
				-0.168
				 

				  
			 

			 
				 
				 
				 

				  

				 
				residual
				 

				  

				 
				1.482e -7
				 

				  

				 
				1.822e 


				-8
				 

				  

				 
				7.983e 


				-8
				 

				  

				 
				1.713e 


				-7
				 

				  

				 
				1.381e 


				-7
				 

				  

				 
				-7.510e 


				-9
				 

				  

				 
				5.783e 


				-8
				 

				  

				 
				2.076e -10
				 

				  

				 
				1.946e -10
				 

				  
			 

			 
				 
				PSS
				 

				  

				 
				observed
				 

				  

				 
				-31.120
				 

				  

				 
				-10.509
				 

				  

				 
				-13.536
				 

				  

				 
				-28.279
				 

				  

				 
				11.014
				 

				  

				 
				13.490
				 

				  

				 
				-10.762
				 

				  

				 
				36.365
				 

				  

				 
				0.344
				 

				  
			 

			 
				 
				 
				 

				  

				 
				fitted
				 

				  

				 
				-31.120
				 

				  

				 
				-10.509
				 

				  

				 
				-13.536
				 

				  

				 
				-28.279
				 

				  

				 
				11.014
				 

				  

				 
				13.490
				 

				  

				 
				-10.762
				 

				  

				 
				36.365
				 

				  

				 
				0.344
				 

				  
			 

			 
				 
				 
				 

				  

				 
				residual
				 

				  

				 
				-9.566e -9
				 

				  

				 
				-2.574e 


				-9
				 

				  

				 
				-3.492e 


				-9
				 

				  

				 
				-9.588e 


				-9
				 

				  

				 
				6.059e -10
				 

				  

				 
				5.798e 


				-9
				 

				  

				 
				-5.982e 


				-9
				 

				  

				 
				0.000
				 

				  

				 
				0.000
				 

				  
			 

			 
				 
				RSQ
				 

				  

				 
				observed
				 

				  

				 
				-21.629
				 

				  

				 
				-7.681
				 

				  

				 
				-11.921
				 

				  

				 
				-18.698
				 

				  

				 
				10.945
				 

				  

				 
				13.467
				 

				  

				 
				-6.166
				 

				  

				 
				15.159
				 

				  

				 
				53.394
				 

				  
			 

			 
				 
				 
				 

				  

				 
				fitted
				 

				  

				 
				-21.629
				 

				  

				 
				-7.681
				 

				  

				 
				-11.921
				 

				  

				 
				-18.698
				 

				  

				 
				10.945
				 

				  

				 
				13.467
				 

				  

				 
				-6.166
				 

				  

				 
				15.159
				 

				  

				 
				53.394
				 

				  
			 

			 
				 
				 
				 

				  

				 
				residual
				 

				  

				 
				2.046e -9
				 

				  

				 
				-3.041e -10
				 

				  

				 
				-9.789e -10
				 

				  

				 
				5.442e -10
				 

				  

				 
				-2.332e 


				-9
				 

				  

				 
				-1.166e 


				-9
				 

				  

				 
				1.193e -10
				 

				  

				 
				0.000
				 

				  

				 
				0.000
				 

				  
			 

			 
				  
			 
		 

		  
	 
 
     
 
 
	 
		 
			 
				 
					 
						 
							 
							Modification Indices
							 

							  

							  
						 
					 
				 
			 

			 
				 
				 
				 

				 
				 
				 

				 
				 
				 

				 
				mi
				 

				 
				epc
				 

				 
				sepc (lv)
				 

				 
				sepc (all)
				 

				 
				sepc (nox)
				 
			 
		 

		 
			 
				 
				.
				 

				  

				 
				.
				 

				  

				 
				.
				 

				  

				 
				.
				 

				  

				 
				.
				 

				  

				 
				.
				 

				  

				 
				.
				 

				  

				 
				.
				 

				  

			 

			 
				  
			 
		 

		  
	 
 
   
 
 
	 
		 
			 
				 
					 
						 
							 
							Mardia's coefficients
							 

							  

							  
						 
					 
				 
			 

			 
				 
				 
				 

				 
				Coefficient
				 

				 
				z
				 

				 
				χ²
				 

				 
				df
				 

				 
				p
				 
			 
		 

		 
			 
				 
				Skewness
				 

				  

				 
				7.080
				 

				  

				  

				  

				 
				383.517
				 

				  

				 
				165.000
				 

				  

				 
				< .001
				 

				  

			 

			 
				 
				Kurtosis
				 

				  

				 
				105.316
				 

				  

				 
				4.046
				 

				  

				  

				  

				  

				  

				 
				< .001
				 

				  
			 

			 
				  
			 
		 

		  
	 
 
      The model is of bad fit.      
 
  SEM. New Model 9. Best fit for parallel mediation   
 
   
 
 
	 
		 
			 
				 
					 
						 
							 
							Chi Square Test Statistic (unscaled)
							 

							  

							  
						 
					 
				 
			 

			 
				 
				 
				 

				 
				df
				 

				 
				AIC
				 

				 
				BIC
				 

				 
				χ²
				 

				 
				p
				 
			 
		 

		 
			 
				 
				Model
				 

				  

				 
				5.000
				 

				  

				 
				13248.232
				 

				  

				 
				13388.234
				 

				  

				 
				8.884
				 

				  

				 
				0.114
				 

				  

			 

			 
				  
			 
		 

		  
	 
 
   The chi-square (χ2) test tests the null hypothesis that the predicted model and observed data are equal. Ideally, we want your predictions to match the actual data as closely as possible. Therefore, nonsignificant result for this test indicates good model fit.     
 
 
	 
		 
			 
				 
					 
						 
							 
							Parameter Estimates
							 

							  

							  
						 
					 
				 
			 

			 
				 
				 
				 

				 
				 
				 

				 
				 
				 

				 
				label
				 

				 
				est
				 

				 
				se
				 

				 
				z
				 

				 
				p
				 

				 
				CI (lower)
				 

				 
				CI (upper)
				 

				 
				std (lv)
				 

				 
				std (all)
				 

				 
				std (nox)
				 

				 
				group
				 
			 
		 

		 
			 
				 
				RESIL
				 

				  

				 
				~
				 

				  

				 
				SE
				 

				  

				 
				b11
				 

				  

				 
				1.094
				 

				  

				 
				0.135
				 

				  

				 
				8.122
				 

				  

				 
				< .001
				 

				  

				 
				0.806
				 

				  

				 
				1.353
				 

				  

				 
				1.094
				 

				  

				 
				0.364
				 

				  

				 
				0.364
				 

				  

				  

				  

			 

			 
				 
				RESIL
				 

				  

				 
				~
				 

				  

				 
				LOT
				 

				  

				 
				b12
				 

				  

				 
				0.335
				 

				  

				 
				0.112
				 

				  

				 
				3.001
				 

				  

				 
				0.003
				 

				  

				 
				0.123
				 

				  

				 
				0.554
				 

				  

				 
				0.335
				 

				  

				 
				0.146
				 

				  

				 
				0.146
				 

				  

				  

				  
			 

			 
				 
				RESIL
				 

				  

				 
				~
				 

				  

				 
				SC
				 

				  

				 
				b13
				 

				  

				 
				0.249
				 

				  

				 
				0.061
				 

				  

				 
				4.090
				 

				  

				 
				< .001
				 

				  

				 
				0.123
				 

				  

				 
				0.362
				 

				  

				 
				0.249
				 

				  

				 
				0.197
				 

				  

				 
				0.197
				 

				  

				  

				  
			 

			 
				 
				RESIL
				 

				  

				 
				~
				 

				  

				 
				STS
				 

				  

				 
				b14
				 

				  

				 
				0.246
				 

				  

				 
				0.099
				 

				  

				 
				2.494
				 

				  

				 
				0.013
				 

				  

				 
				0.049
				 

				  

				 
				0.437
				 

				  

				 
				0.246
				 

				  

				 
				0.099
				 

				  

				 
				0.099
				 

				  

				  

				  
			 

			 
				 
				RESIL
				 

				  

				 
				~
				 

				  

				 
				BU
				 

				  

				 
				b15
				 

				  

				 
				-0.519
				 

				  

				 
				0.121
				 

				  

				 
				-4.275
				 

				  

				 
				< .001
				 

				  

				 
				-0.779
				 

				  

				 
				-0.278
				 

				  

				 
				-0.519
				 

				  

				 
				-0.226
				 

				  

				 
				-0.226
				 

				  

				  

				  
			 

			 
				 
				RESIL
				 

				  

				 
				~
				 

				  

				 
				CS
				 

				  

				 
				b16
				 

				  

				 
				0.351
				 

				  

				 
				0.103
				 

				  

				 
				3.412
				 

				  

				 
				< .001
				 

				  

				 
				0.154
				 

				  

				 
				0.558
				 

				  

				 
				0.351
				 

				  

				 
				0.158
				 

				  

				 
				0.158
				 

				  

				  

				  
			 

			 
				 
				RESIL
				 

				  

				 
				~
				 

				  

				 
				PSS
				 

				  

				 
				c11
				 

				  

				 
				-0.016
				 

				  

				 
				0.077
				 

				  

				 
				-0.210
				 

				  

				 
				0.833
				 

				  

				 
				-0.177
				 

				  

				 
				0.137
				 

				  

				 
				-0.016
				 

				  

				 
				-0.009
				 

				  

				 
				-0.001
				 

				  

				  

				  
			 

			 
				 
				RESIL
				 

				  

				 
				~
				 

				  

				 
				RSQ
				 

				  

				 
				c12
				 

				  

				 
				0.040
				 

				  

				 
				0.058
				 

				  

				 
				0.685
				 

				  

				 
				0.493
				 

				  

				 
				-0.070
				 

				  

				 
				0.153
				 

				  

				 
				0.040
				 

				  

				 
				0.026
				 

				  

				 
				0.004
				 

				  

				  

				  
			 

			 
				 
				SE
				 

				  

				 
				~
				 

				  

				 
				PSS
				 

				  

				 
				a11
				 

				  

				 
				-0.289
				 

				  

				 
				0.037
				 

				  

				 
				-7.767
				 

				  

				 
				< .001
				 

				  

				 
				-0.366
				 

				  

				 
				-0.214
				 

				  

				 
				-0.289
				 

				  

				 
				-0.468
				 

				  

				 
				-0.078
				 

				  

				  

				  
			 

			 
				 
				LOT
				 

				  

				 
				~
				 

				  

				 
				PSS
				 

				  

				 
				a21
				 

				  

				 
				-0.334
				 

				  

				 
				0.048
				 

				  

				 
				-6.957
				 

				  

				 
				< .001
				 

				  

				 
				-0.427
				 

				  

				 
				-0.235
				 

				  

				 
				-0.334
				 

				  

				 
				-0.412
				 

				  

				 
				-0.068
				 

				  

				  

				  
			 

			 
				 
				LOT
				 

				  

				 
				~
				 

				  

				 
				RSQ
				 

				  

				 
				a22
				 

				  

				 
				-0.091
				 

				  

				 
				0.028
				 

				  

				 
				-3.190
				 

				  

				 
				0.001
				 

				  

				 
				-0.144
				 

				  

				 
				-0.035
				 

				  

				 
				-0.091
				 

				  

				 
				-0.135
				 

				  

				 
				-0.019
				 

				  

				  

				  
			 

			 
				 
				SC
				 

				  

				 
				~
				 

				  

				 
				PSS
				 

				  

				 
				a31
				 

				  

				 
				-0.720
				 

				  

				 
				0.073
				 

				  

				 
				-9.903
				 

				  

				 
				< .001
				 

				  

				 
				-0.864
				 

				  

				 
				-0.581
				 

				  

				 
				-0.720
				 

				  

				 
				-0.489
				 

				  

				 
				-0.081
				 

				  

				  

				  
			 

			 
				 
				STS
				 

				  

				 
				~
				 

				  

				 
				PSS
				 

				  

				 
				a41
				 

				  

				 
				0.251
				 

				  

				 
				0.043
				 

				  

				 
				5.890
				 

				  

				 
				< .001
				 

				  

				 
				0.171
				 

				  

				 
				0.342
				 

				  

				 
				0.251
				 

				  

				 
				0.337
				 

				  

				 
				0.056
				 

				  

				  

				  
			 

			 
				 
				STS
				 

				  

				 
				~
				 

				  

				 
				RSQ
				 

				  

				 
				a42
				 

				  

				 
				0.125
				 

				  

				 
				0.035
				 

				  

				 
				3.618
				 

				  

				 
				< .001
				 

				  

				 
				0.058
				 

				  

				 
				0.197
				 

				  

				 
				0.125
				 

				  

				 
				0.204
				 

				  

				 
				0.028
				 

				  

				  

				  
			 

			 
				 
				BU
				 

				  

				 
				~
				 

				  

				 
				PSS
				 

				  

				 
				a51
				 

				  

				 
				0.317
				 

				  

				 
				0.046
				 

				  

				 
				6.887
				 

				  

				 
				< .001
				 

				  

				 
				0.227
				 

				  

				 
				0.411
				 

				  

				 
				0.317
				 

				  

				 
				0.393
				 

				  

				 
				0.065
				 

				  

				  

				  
			 

			 
				 
				BU
				 

				  

				 
				~
				 

				  

				 
				RSQ
				 

				  

				 
				a52
				 

				  

				 
				0.130
				 

				  

				 
				0.028
				 

				  

				 
				4.630
				 

				  

				 
				< .001
				 

				  

				 
				0.073
				 

				  

				 
				0.184
				 

				  

				 
				0.130
				 

				  

				 
				0.196
				 

				  

				 
				0.027
				 

				  

				  

				  
			 

			 
				 
				CS
				 

				  

				 
				~
				 

				  

				 
				PSS
				 

				  

				 
				a61
				 

				  

				 
				-0.296
				 

				  

				 
				0.051
				 

				  

				 
				-5.769
				 

				  

				 
				< .001
				 

				  

				 
				-0.397
				 

				  

				 
				-0.200
				 

				  

				 
				-0.296
				 

				  

				 
				-0.355
				 

				  

				 
				-0.059
				 

				  

				  

				  
			 

			 
				 
				SE
				 

				  

				 
				~~
				 

				  

				 
				LOT
				 

				  

				  

				  

				 
				4.353
				 

				  

				 
				0.851
				 

				  

				 
				5.116
				 

				  

				 
				< .001
				 

				  

				 
				2.647
				 

				  

				 
				6.084
				 

				  

				 
				4.353
				 

				  

				 
				0.308
				 

				  

				 
				0.308
				 

				  

				  

				  
			 

			 
				 
				SE
				 

				  

				 
				~~
				 

				  

				 
				SC
				 

				  

				  

				  

				 
				6.956
				 

				  

				 
				1.498
				 

				  

				 
				4.643
				 

				  

				 
				< .001
				 

				  

				 
				3.935
				 

				  

				 
				9.737
				 

				  

				 
				6.956
				 

				  

				 
				0.282
				 

				  

				 
				0.282
				 

				  

				  

				  
			 

			 
				 
				LOT
				 

				  

				 
				~~
				 

				  

				 
				SC
				 

				  

				  

				  

				 
				12.725
				 

				  

				 
				2.343
				 

				  

				 
				5.432
				 

				  

				 
				< .001
				 

				  

				 
				8.263
				 

				  

				 
				17.643
				 

				  

				 
				12.725
				 

				  

				 
				0.395
				 

				  

				 
				0.395
				 

				  

				  

				  
			 

			 
				 
				SE
				 

				  

				 
				~~
				 

				  

				 
				BU
				 

				  

				  

				  

				 
				-3.896
				 

				  

				 
				0.864
				 

				  

				 
				-4.510
				 

				  

				 
				< .001
				 

				  

				 
				-5.607
				 

				  

				 
				-2.272
				 

				  

				 
				-3.896
				 

				  

				 
				-0.281
				 

				  

				 
				-0.281
				 

				  

				  

				  
			 

			 
				 
				LOT
				 

				  

				 
				~~
				 

				  

				 
				BU
				 

				  

				  

				  

				 
				-5.892
				 

				  

				 
				1.226
				 

				  

				 
				-4.805
				 

				  

				 
				< .001
				 

				  

				 
				-8.427
				 

				  

				 
				-3.524
				 

				  

				 
				-5.892
				 

				  

				 
				-0.325
				 

				  

				 
				-0.325
				 

				  

				  

				  
			 

			 
				 
				SC
				 

				  

				 
				~~
				 

				  

				 
				BU
				 

				  

				  

				  

				 
				-10.710
				 

				  

				 
				2.147
				 

				  

				 
				-4.987
				 

				  

				 
				< .001
				 

				  

				 
				-15.219
				 

				  

				 
				-6.617
				 

				  

				 
				-10.710
				 

				  

				 
				-0.339
				 

				  

				 
				-0.339
				 

				  

				  

				  
			 

			 
				 
				STS
				 

				  

				 
				~~
				 

				  

				 
				BU
				 

				  

				  

				  

				 
				5.148
				 

				  

				 
				1.044
				 

				  

				 
				4.932
				 

				  

				 
				< .001
				 

				  

				 
				3.161
				 

				  

				 
				7.260
				 

				  

				 
				5.148
				 

				  

				 
				0.305
				 

				  

				 
				0.305
				 

				  

				  

				  
			 

			 
				 
				SE
				 

				  

				 
				~~
				 

				  

				 
				CS
				 

				  

				  

				  

				 
				4.514
				 

				  

				 
				1.047
				 

				  

				 
				4.310
				 

				  

				 
				< .001
				 

				  

				 
				2.400
				 

				  

				 
				6.527
				 

				  

				 
				4.514
				 

				  

				 
				0.292
				 

				  

				 
				0.292
				 

				  

				  

				  
			 

			 
				 
				LOT
				 

				  

				 
				~~
				 

				  

				 
				CS
				 

				  

				  

				  

				 
				4.127
				 

				  

				 
				1.269
				 

				  

				 
				3.252
				 

				  

				 
				0.001
				 

				  

				 
				1.710
				 

				  

				 
				6.603
				 

				  

				 
				4.127
				 

				  

				 
				0.204
				 

				  

				 
				0.204
				 

				  

				  

				  
			 

			 
				 
				SC
				 

				  

				 
				~~
				 

				  

				 
				CS
				 

				  

				  

				  

				 
				8.687
				 

				  

				 
				2.257
				 

				  

				 
				3.848
				 

				  

				 
				< .001
				 

				  

				 
				4.340
				 

				  

				 
				13.224
				 

				  

				 
				8.687
				 

				  

				 
				0.247
				 

				  

				 
				0.247
				 

				  

				  

				  
			 

			 
				 
				BU
				 

				  

				 
				~~
				 

				  

				 
				CS
				 

				  

				  

				  

				 
				-12.177
				 

				  

				 
				1.595
				 

				  

				 
				-7.636
				 

				  

				 
				< .001
				 

				  

				 
				-15.402
				 

				  

				 
				-9.085
				 

				  

				 
				-12.177
				 

				  

				 
				-0.614
				 

				  

				 
				-0.614
				 

				  

				  

				  
			 

			 
				 
				SE
				 

				  

				 
				~~
				 

				  

				 
				STS
				 

				  

				  

				  

				 
				-1.759
				 

				  

				 
				0.665
				 

				  

				 
				-2.645
				 

				  

				 
				0.008
				 

				  

				 
				-3.101
				 

				  

				 
				-0.435
				 

				  

				 
				-1.759
				 

				  

				 
				-0.134
				 

				  

				 
				-0.134
				 

				  

				  

				  
			 

			 
				 
				SC
				 

				  

				 
				~
				 

				  

				 
				STS
				 

				  

				  

				  

				 
				-0.189
				 

				  

				 
				0.087
				 

				  

				 
				-2.179
				 

				  

				 
				0.029
				 

				  

				 
				-0.358
				 

				  

				 
				-0.009
				 

				  

				 
				-0.189
				 

				  

				 
				-0.095
				 

				  

				 
				-0.095
				 

				  

				  

				  
			 

			 
				 
				RESIL
				 

				  

				 
				~~
				 

				  

				 
				RESIL
				 

				  

				  

				  

				 
				44.757
				 

				  

				 
				3.616
				 

				  

				 
				12.378
				 

				  

				 
				< .001
				 

				  

				 
				36.568
				 

				  

				 
				50.771
				 

				  

				 
				44.757
				 

				  

				 
				0.358
				 

				  

				 
				0.358
				 

				  

				  

				  
			 

			 
				 
				SE
				 

				  

				 
				~~
				 

				  

				 
				SE
				 

				  

				  

				  

				 
				10.820
				 

				  

				 
				0.799
				 

				  

				 
				13.546
				 

				  

				 
				< .001
				 

				  

				 
				9.253
				 

				  

				 
				12.352
				 

				  

				 
				10.820
				 

				  

				 
				0.781
				 

				  

				 
				0.781
				 

				  

				  

				  
			 

			 
				 
				LOT
				 

				  

				 
				~~
				 

				  

				 
				LOT
				 

				  

				  

				  

				 
				18.466
				 

				  

				 
				1.521
				 

				  

				 
				12.140
				 

				  

				 
				< .001
				 

				  

				 
				15.449
				 

				  

				 
				21.345
				 

				  

				 
				18.466
				 

				  

				 
				0.773
				 

				  

				 
				0.773
				 

				  

				  

				  
			 

			 
				 
				SC
				 

				  

				 
				~~
				 

				  

				 
				SC
				 

				  

				  

				  

				 
				56.113
				 

				  

				 
				4.607
				 

				  

				 
				12.179
				 

				  

				 
				< .001
				 

				  

				 
				47.463
				 

				  

				 
				64.934
				 

				  

				 
				56.113
				 

				  

				 
				0.714
				 

				  

				 
				0.714
				 

				  

				  

				  
			 

			 
				 
				STS
				 

				  

				 
				~~
				 

				  

				 
				STS
				 

				  

				  

				  

				 
				16.024
				 

				  

				 
				1.461
				 

				  

				 
				10.964
				 

				  

				 
				< .001
				 

				  

				 
				13.317
				 

				  

				 
				18.960
				 

				  

				 
				16.024
				 

				  

				 
				0.798
				 

				  

				 
				0.798
				 

				  

				  

				  
			 

			 
				 
				BU
				 

				  

				 
				~~
				 

				  

				 
				BU
				 

				  

				  

				  

				 
				17.805
				 

				  

				 
				1.502
				 

				  

				 
				11.857
				 

				  

				 
				< .001
				 

				  

				 
				14.543
				 

				  

				 
				20.687
				 

				  

				 
				17.805
				 

				  

				 
				0.755
				 

				  

				 
				0.755
				 

				  

				  

				  
			 

			 
				 
				CS
				 

				  

				 
				~~
				 

				  

				 
				CS
				 

				  

				  

				  

				 
				22.079
				 

				  

				 
				2.023
				 

				  

				 
				10.914
				 

				  

				 
				< .001
				 

				  

				 
				18.243
				 

				  

				 
				26.324
				 

				  

				 
				22.079
				 

				  

				 
				0.874
				 

				  

				 
				0.874
				 

				  

				  

				  
			 

			 
				 
				PSS
				 

				  

				 
				~~
				 

				  

				 
				PSS
				 

				  

				  

				  

				 
				36.253
				 

				  

				 
				0.000
				 

				  

				  

				  

				 
				 
				 

				  

				 
				36.253
				 

				  

				 
				36.253
				 

				  

				 
				36.253
				 

				  

				 
				1.000
				 

				  

				 
				36.253
				 

				  

				  

				  
			 

			 
				 
				PSS
				 

				  

				 
				~~
				 

				  

				 
				RSQ
				 

				  

				  

				  

				 
				15.112
				 

				  

				 
				0.000
				 

				  

				  

				  

				 
				 
				 

				  

				 
				15.112
				 

				  

				 
				15.112
				 

				  

				 
				15.112
				 

				  

				 
				0.344
				 

				  

				 
				15.112
				 

				  

				  

				  
			 

			 
				 
				RSQ
				 

				  

				 
				~~
				 

				  

				 
				RSQ
				 

				  

				  

				  

				 
				53.229
				 

				  

				 
				0.000
				 

				  

				  

				  

				 
				 
				 

				  

				 
				53.229
				 

				  

				 
				53.229
				 

				  

				 
				53.229
				 

				  

				 
				1.000
				 

				  

				 
				53.229
				 

				  

				  

				  
			 

			 
				 
				ind_x1_m1_y1
				 

				  

				 
				:=
				 

				  

				 
				a11*b11
				 

				  

				 
				ind_x1_m1_y1
				 

				  

				 
				-0.316
				 

				  

				 
				0.055
				 

				  

				 
				-5.733
				 

				  

				 
				< .001
				 

				  

				 
				-0.437
				 

				  

				 
				-0.209
				 

				  

				 
				-0.316
				 

				  

				 
				-0.170
				 

				  

				 
				-0.028
				 

				  

				  

				  
			 

			 
				 
				ind_x1_m2_y1
				 

				  

				 
				:=
				 

				  

				 
				a21*b12
				 

				  

				 
				ind_x1_m2_y1
				 

				  

				 
				-0.112
				 

				  

				 
				0.041
				 

				  

				 
				-2.751
				 

				  

				 
				0.006
				 

				  

				 
				-0.200
				 

				  

				 
				-0.039
				 

				  

				 
				-0.112
				 

				  

				 
				-0.060
				 

				  

				 
				-0.010
				 

				  

				  

				  
			 

			 
				 
				ind_x1_m3_y1
				 

				  

				 
				:=
				 

				  

				 
				a31*b13
				 

				  

				 
				ind_x1_m3_y1
				 

				  

				 
				-0.179
				 

				  

				 
				0.048
				 

				  

				 
				-3.719
				 

				  

				 
				< .001
				 

				  

				 
				-0.279
				 

				  

				 
				-0.086
				 

				  

				 
				-0.179
				 

				  

				 
				-0.097
				 

				  

				 
				-0.016
				 

				  

				  

				  
			 

			 
				 
				ind_x1_m4_y1
				 

				  

				 
				:=
				 

				  

				 
				a41*b14
				 

				  

				 
				ind_x1_m4_y1
				 

				  

				 
				0.062
				 

				  

				 
				0.026
				 

				  

				 
				2.348
				 

				  

				 
				0.019
				 

				  

				 
				0.013
				 

				  

				 
				0.118
				 

				  

				 
				0.062
				 

				  

				 
				0.033
				 

				  

				 
				0.006
				 

				  

				  

				  
			 

			 
				 
				ind_x1_m5_y1
				 

				  

				 
				:=
				 

				  

				 
				a51*b15
				 

				  

				 
				ind_x1_m5_y1
				 

				  

				 
				-0.164
				 

				  

				 
				0.046
				 

				  

				 
				-3.579
				 

				  

				 
				< .001
				 

				  

				 
				-0.269
				 

				  

				 
				-0.087
				 

				  

				 
				-0.164
				 

				  

				 
				-0.089
				 

				  

				 
				-0.015
				 

				  

				  

				  
			 

			 
				 
				ind_x1_m6_y1
				 

				  

				 
				:=
				 

				  

				 
				a61*b16
				 

				  

				 
				ind_x1_m6_y1
				 

				  

				 
				-0.104
				 

				  

				 
				0.035
				 

				  

				 
				-2.932
				 

				  

				 
				0.003
				 

				  

				 
				-0.182
				 

				  

				 
				-0.041
				 

				  

				 
				-0.104
				 

				  

				 
				-0.056
				 

				  

				 
				-0.009
				 

				  

				  

				  
			 

			 
				 
				ind_x1_y1
				 

				  

				 
				:=
				 

				  

				 
				ind_x1_m1_y1+ind_x1_m2_y1+ind_x1_m3_y1+ind_x1_m4_y1+ind_x1_m5_y1+ind_x1_m6_y1
				 

				  

				 
				ind_x1_y1
				 

				  

				 
				-0.814
				 

				  

				 
				0.100
				 

				  

				 
				-8.122
				 

				  

				 
				< .001
				 

				  

				 
				-1.021
				 

				  

				 
				-0.622
				 

				  

				 
				-0.814
				 

				  

				 
				-0.438
				 

				  

				 
				-0.073
				 

				  

				  

				  
			 

			 
				 
				tot_x1_y1
				 

				  

				 
				:=
				 

				  

				 
				ind_x1_y1+c11
				 

				  

				 
				tot_x1_y1
				 

				  

				 
				-0.830
				 

				  

				 
				0.111
				 

				  

				 
				-7.497
				 

				  

				 
				< .001
				 

				  

				 
				-1.052
				 

				  

				 
				-0.615
				 

				  

				 
				-0.830
				 

				  

				 
				-0.447
				 

				  

				 
				-0.074
				 

				  

				  

				  
			 

			 
				 
				ind_x2_m2_y1
				 

				  

				 
				:=
				 

				  

				 
				a22*b12
				 

				  

				 
				ind_x2_m2_y1
				 

				  

				 
				-0.030
				 

				  

				 
				0.014
				 

				  

				 
				-2.232
				 

				  

				 
				0.026
				 

				  

				 
				-0.057
				 

				  

				 
				-0.007
				 

				  

				 
				-0.030
				 

				  

				 
				-0.020
				 

				  

				 
				-0.003
				 

				  

				  

				  
			 

			 
				 
				ind_x2_m4_y1
				 

				  

				 
				:=
				 

				  

				 
				a42*b14
				 

				  

				 
				ind_x2_m4_y1
				 

				  

				 
				0.031
				 

				  

				 
				0.016
				 

				  

				 
				1.873
				 

				  

				 
				0.061
				 

				  

				 
				0.005
				 

				  

				 
				0.067
				 

				  

				 
				0.031
				 

				  

				 
				0.020
				 

				  

				 
				0.003
				 

				  

				  

				  
			 

			 
				 
				ind_x2_m5_y1
				 

				  

				 
				:=
				 

				  

				 
				a52*b15
				 

				  

				 
				ind_x2_m5_y1
				 

				  

				 
				-0.068
				 

				  

				 
				0.022
				 

				  

				 
				-3.038
				 

				  

				 
				0.002
				 

				  

				 
				-0.117
				 

				  

				 
				-0.030
				 

				  

				 
				-0.068
				 

				  

				 
				-0.044
				 

				  

				 
				-0.006
				 

				  

				  

				  
			 

			 
				 
				ind_x2_y1
				 

				  

				 
				:=
				 

				  

				 
				ind_x2_m2_y1+ind_x2_m4_y1+ind_x2_m5_y1
				 

				  

				 
				ind_x2_y1
				 

				  

				 
				-0.067
				 

				  

				 
				0.025
				 

				  

				 
				-2.665
				 

				  

				 
				0.008
				 

				  

				 
				-0.117
				 

				  

				 
				-0.022
				 

				  

				 
				-0.067
				 

				  

				 
				-0.044
				 

				  

				 
				-0.006
				 

				  

				  

				  
			 

			 
				 
				tot_x2_y1
				 

				  

				 
				:=
				 

				  

				 
				ind_x2_y1+c12
				 

				  

				 
				tot_x2_y1
				 

				  

				 
				-0.027
				 

				  

				 
				0.057
				 

				  

				 
				-0.480
				 

				  

				 
				0.631
				 

				  

				 
				-0.138
				 

				  

				 
				0.085
				 

				  

				 
				-0.027
				 

				  

				 
				-0.018
				 

				  

				 
				-0.002
				 

				  

				  

				  
			 

			 
				  
			 
		 

		  
	 
 
   
 
   
 
 
	 
		 
			 
				 
					 
						 
							 
							Model test baseline model
							 

							  

							  
						 
					 
				 
			 

			 
				 
				 
				 

				 
				Model
				 
			 
		 

		 
			 
				 
				Minimum Function Test Statistic
				 

				  

				 
				0.014
				 

				  

			 

			 
				 
				χ²
				 

				  

				 
				8.884
				 

				  
			 

			 
				 
				Degrees of freedom
				 

				  

				 
				5.000
				 

				  
			 

			 
				 
				p
				 

				  

				 
				0.114
				 

				  
			 

			 
				  
			 
		 

		  
	 
 
    A good model–data fit is indicated by RMSEA < .06, CFI > .95, and TLI > .95 (Hu & Bentler,   1999  )      
 
 
	 
		 
			 
				 
					 
						 
							 
							User model versus baseline model
							 

							  

							  
						 
					 
				 
			 

			 
				 
				 
				 

				 
				Model
				 
			 
		 

		 
			 
				 
				Comparative Fit Index (CFI)
				 

				  

				 
				0.997
				 

				  

			 

			 
				 
				Tucker-Lewis Index (TLI)
				 

				  

				 
				0.977
				 

				  
			 

			 
				 
				Bentler-Bonett Non-normed Fit Index (NNFI)
				 

				  

				 
				0.977
				 

				  
			 

			 
				 
				Bentler-Bonett Normed Fit Index (NFI)
				 

				  

				 
				0.993
				 

				  
			 

			 
				 
				Parsimony Normed Fit Index (PNFI)
				 

				  

				 
				0.142
				 

				  
			 

			 
				 
				Bollen's Relative Fit Index (RFI)
				 

				  

				 
				0.949
				 

				  
			 

			 
				 
				Bollen's Incremental Fit Index (IFI)
				 

				  

				 
				0.997
				 

				  
			 

			 
				 
				Relative Noncentrality Index (RNI)
				 

				  

				 
				0.997
				 

				  
			 

			 
				  
			 
		 

		  
	 
 
   
 
 
	 
		 
			 
				 
					 
						 
							 
							Loglikelihood and Information Criteria
							 

							  

							  
						 
					 
				 
			 

			 
				 
				 
				 

				 
				Model
				 
			 
		 

		 
			 
				 
				Loglikelihood user model (H0)
				 

				  

				 
				-6587.116
				 

				  

			 

			 
				 
				Loglikelihood unrestricted model (H1)
				 

				  

				 
				-6582.674
				 

				  
			 

			 
				 
				Number of free parameters
				 

				  

				 
				37
				 

				  
			 

			 
				 
				Akaike (AIC)
				 

				  

				 
				13248.232
				 

				  
			 

			 
				 
				Bayesian (BIC)
				 

				  

				 
				13388.234
				 

				  
			 

			 
				 
				Sample-size adjusted Bayesian (BIC)
				 

				  

				 
				13270.873
				 

				  
			 

			 
				  
			 
		 

		  
	 
 
   
 
 
	 
		 
			 
				 
					 
						 
							 
							Root Mean Square Error of Approximation
							 

							  

							  
						 
					 
				 
			 

			 
				 
				 
				 

				 
				Model
				 
			 
		 

		 
			 
				 
				RMSEA
				 

				  

				 
				0.049
				 

				  

			 

			 
				 
				Upper 90% CI
				 

				  

				 
				0.100
				 

				  
			 

			 
				 
				Lower 90% CI
				 

				  

				 
				0.000
				 

				  
			 

			 
				 
				p-value RMSEA <= 0.05 
				 

				  

				 
				0.445
				 

				  
			 

			 
				  
			 
		 

		  
	 
 
   
 
 
	 
		 
			 
				 
					 
						 
							 
							Standardized Root Mean Square Residual
							 

							  

							  
						 
					 
				 
			 

			 
				 
				 
				 

				 
				Model
				 
			 
		 

		 
			 
				 
				RMR
				 

				  

				 
				1.563
				 

				  

			 

			 
				 
				RMR (No Mean)
				 

				  

				 
				1.563
				 

				  
			 

			 
				 
				SRMR
				 

				  

				 
				0.031
				 

				  
			 

			 
				  
			 
		 

		  
	 
 
   
 
 
	 
		 
			 
				 
					 
						 
							 
							Other Fit Indices
							 

							  

							  
						 
					 
				 
			 

			 
				 
				 
				 

				 
				Model
				 
			 
		 

		 
			 
				 
				Hoelter Critical N (CN) alpha=0.05
				 

				  

				 
				406.007
				 

				  

			 

			 
				 
				Hoelter Critical N (CN) alpha=0.01
				 

				  

				 
				552.922
				 

				  
			 

			 
				 
				Goodness of Fit Index (GFI)
				 

				  

				 
				0.993
				 

				  
			 

			 
				 
				Parsimony Goodness of Fit Index (GFI)
				 

				  

				 
				0.936
				 

				  
			 

			 
				 
				McDonald Fit Index (MFI)
				 

				  

				 
				0.994
				 

				  
			 

			 
				  
			 
		 

		  
	 
 
     
 
 
	 
		 
			 
				 
					 
						 
							 
							R-Squared
							 

							  

							  
						 
					 
				 
			 

			 
				 
				Variable
				 

				 
				R²
				 
			 
		 

		 
			 
				 
				RESIL
				 

				  

				 
				0.642
				 

				  

			 

			 
				 
				SE
				 

				  

				 
				0.219
				 

				  
			 

			 
				 
				LOT
				 

				  

				 
				0.227
				 

				  
			 

			 
				 
				SC
				 

				  

				 
				0.286
				 

				  
			 

			 
				 
				STS
				 

				  

				 
				0.202
				 

				  
			 

			 
				 
				BU
				 

				  

				 
				0.245
				 

				  
			 

			 
				 
				CS
				 

				  

				 
				0.126
				 

				  
			 

			 
				  
			 
		 

		  
	 
 
  Individual R2: indicate that the covariates involved this proportion of variation in the mediator     
 
   
 
 
	 
		 
			 
				 
					 
						 
							 
							Covariances (lower triangle) / correlations (upper triangle)
							 

							  

							  
						 
					 
				 
			 

			 
				 
				 
				 

				 
				 
				 

				 
				RESIL
				 

				 
				SE
				 

				 
				LOT
				 

				 
				SC
				 

				 
				STS
				 

				 
				BU
				 

				 
				CS
				 

				 
				PSS
				 

				 
				RSQ
				 
			 
		 

		 
			 
				 
				RESIL
				 

				  

				 
				observed
				 

				  

				 
				125.379
				 

				  

				 
				0.657
				 

				  

				 
				0.563
				 

				  

				 
				0.596
				 

				  

				 
				-0.239
				 

				  

				 
				-0.621
				 

				  

				 
				0.565
				 

				  

				 
				-0.460
				 

				  

				 
				-0.264
				 

				  

			 

			 
				 
				 
				 

				  

				 
				fitted
				 

				  

				 
				124.888
				 

				  

				 
				0.655
				 

				  

				 
				0.555
				 

				  

				 
				0.594
				 

				  

				 
				-0.220
				 

				  

				 
				-0.610
				 

				  

				 
				0.565
				 

				  

				 
				-0.461
				 

				  

				 
				-0.178
				 

				  
			 

			 
				 
				 
				 

				  

				 
				residual
				 

				  

				 
				0.491
				 

				  

				 
				0.002
				 

				  

				 
				0.008
				 

				  

				 
				0.002
				 

				  

				 
				-0.019
				 

				  

				 
				-0.011
				 

				  

				 
				8.770e -5
				 

				  

				 
				9.033e -4
				 

				  

				 
				-0.086
				 

				  
			 

			 
				 
				SE
				 

				  

				 
				observed
				 

				  

				 
				27.373
				 

				  

				 
				13.854
				 

				  

				 
				0.468
				 

				  

				 
				0.469
				 

				  

				 
				-0.321
				 

				  

				 
				-0.451
				 

				  

				 
				0.408
				 

				  

				 
				-0.467
				 

				  

				 
				-0.282
				 

				  
			 

			 
				 
				 
				 

				  

				 
				fitted
				 

				  

				 
				27.248
				 

				  

				 
				13.847
				 

				  

				 
				0.454
				 

				  

				 
				0.468
				 

				  

				 
				-0.296
				 

				  

				 
				-0.431
				 

				  

				 
				0.407
				 

				  

				 
				-0.468
				 

				  

				 
				-0.161
				 

				  
			 

			 
				 
				 
				 

				  

				 
				residual
				 

				  

				 
				0.125
				 

				  

				 
				0.007
				 

				  

				 
				0.014
				 

				  

				 
				0.001
				 

				  

				 
				-0.026
				 

				  

				 
				-0.020
				 

				  

				 
				6.649e -4
				 

				  

				 
				1.171e -4
				 

				  

				 
				-0.121
				 

				  
			 

			 
				 
				LOT
				 

				  

				 
				observed
				 

				  

				 
				31.026
				 

				  

				 
				8.570
				 

				  

				 
				24.238
				 

				  

				 
				0.547
				 

				  

				 
				-0.230
				 

				  

				 
				-0.494
				 

				  

				 
				0.334
				 

				  

				 
				-0.455
				 

				  

				 
				-0.331
				 

				  
			 

			 
				 
				 
				 

				  

				 
				fitted
				 

				  

				 
				30.302
				 

				  

				 
				8.252
				 

				  

				 
				23.874
				 

				  

				 
				0.538
				 

				  

				 
				-0.211
				 

				  

				 
				-0.483
				 

				  

				 
				0.331
				 

				  

				 
				-0.459
				 

				  

				 
				-0.277
				 

				  
			 

			 
				 
				 
				 

				  

				 
				residual
				 

				  

				 
				0.723
				 

				  

				 
				0.318
				 

				  

				 
				0.364
				 

				  

				 
				0.009
				 

				  

				 
				-0.019
				 

				  

				 
				-0.011
				 

				  

				 
				0.004
				 

				  

				 
				0.003
				 

				  

				 
				-0.054
				 

				  
			 

			 
				 
				SC
				 

				  

				 
				observed
				 

				  

				 
				59.218
				 

				  

				 
				15.491
				 

				  

				 
				23.924
				 

				  

				 
				78.811
				 

				  

				 
				-0.305
				 

				  

				 
				-0.528
				 

				  

				 
				0.383
				 

				  

				 
				-0.527
				 

				  

				 
				-0.288
				 

				  
			 

			 
				 
				 
				 

				  

				 
				fitted
				 

				  

				 
				58.859
				 

				  

				 
				15.435
				 

				  

				 
				23.319
				 

				  

				 
				78.633
				 

				  

				 
				-0.294
				 

				  

				 
				-0.517
				 

				  

				 
				0.382
				 

				  

				 
				-0.528
				 

				  

				 
				-0.199
				 

				  
			 

			 
				 
				 
				 

				  

				 
				residual
				 

				  

				 
				0.359
				 

				  

				 
				0.056
				 

				  

				 
				0.605
				 

				  

				 
				0.178
				 

				  

				 
				-0.011
				 

				  

				 
				-0.011
				 

				  

				 
				0.001
				 

				  

				 
				5.978e -4
				 

				  

				 
				-0.089
				 

				  
			 

			 
				 
				STS
				 

				  

				 
				observed
				 

				  

				 
				-12.016
				 

				  

				 
				-5.375
				 

				  

				 
				-5.092
				 

				  

				 
				-12.185
				 

				  

				 
				20.200
				 

				  

				 
				0.468
				 

				  

				 
				-0.159
				 

				  

				 
				0.406
				 

				  

				 
				0.333
				 

				  
			 

			 
				 
				 
				 

				  

				 
				fitted
				 

				  

				 
				-11.001
				 

				  

				 
				-4.932
				 

				  

				 
				-4.620
				 

				  

				 
				-11.702
				 

				  

				 
				20.085
				 

				  

				 
				0.459
				 

				  

				 
				-0.144
				 

				  

				 
				0.407
				 

				  

				 
				0.320
				 

				  
			 

			 
				 
				 
				 

				  

				 
				residual
				 

				  

				 
				-1.015
				 

				  

				 
				-0.443
				 

				  

				 
				-0.472
				 

				  

				 
				-0.483
				 

				  

				 
				0.115
				 

				  

				 
				0.010
				 

				  

				 
				-0.015
				 

				  

				 
				-0.001
				 

				  

				 
				0.013
				 

				  
			 

			 
				 
				BU
				 

				  

				 
				observed
				 

				  

				 
				-34.121
				 

				  

				 
				-8.229
				 

				  

				 
				-11.926
				 

				  

				 
				-23.011
				 

				  

				 
				10.331
				 

				  

				 
				24.081
				 

				  

				 
				-0.666
				 

				  

				 
				0.455
				 

				  

				 
				0.375
				 

				  
			 

			 
				 
				 
				 

				  

				 
				fitted
				 

				  

				 
				-33.100
				 

				  

				 
				-7.782
				 

				  

				 
				-11.452
				 

				  

				 
				-22.285
				 

				  

				 
				9.986
				 

				  

				 
				23.591
				 

				  

				 
				-0.662
				 

				  

				 
				0.460
				 

				  

				 
				0.331
				 

				  
			 

			 
				 
				 
				 

				  

				 
				residual
				 

				  

				 
				-1.021
				 

				  

				 
				-0.447
				 

				  

				 
				-0.474
				 

				  

				 
				-0.726
				 

				  

				 
				0.345
				 

				  

				 
				0.490
				 

				  

				 
				-0.004
				 

				  

				 
				-0.005
				 

				  

				 
				0.044
				 

				  
			 

			 
				 
				CS
				 

				  

				 
				observed
				 

				  

				 
				31.798
				 

				  

				 
				7.629
				 

				  

				 
				8.272
				 

				  

				 
				17.094
				 

				  

				 
				-3.590
				 

				  

				 
				-16.417
				 

				  

				 
				25.254
				 

				  

				 
				-0.355
				 

				  

				 
				-0.168
				 

				  
			 

			 
				 
				 
				 

				  

				 
				fitted
				 

				  

				 
				31.731
				 

				  

				 
				7.614
				 

				  

				 
				8.120
				 

				  

				 
				17.030
				 

				  

				 
				-3.249
				 

				  

				 
				-16.157
				 

				  

				 
				25.254
				 

				  

				 
				-0.355
				 

				  

				 
				-0.122
				 

				  
			 

			 
				 
				 
				 

				  

				 
				residual
				 

				  

				 
				0.067
				 

				  

				 
				0.014
				 

				  

				 
				0.152
				 

				  

				 
				0.064
				 

				  

				 
				-0.340
				 

				  

				 
				-0.260
				 

				  

				 
				2.960e -5
				 

				  

				 
				2.155e -7
				 

				  

				 
				-0.046
				 

				  
			 

			 
				 
				PSS
				 

				  

				 
				observed
				 

				  

				 
				-31.025
				 

				  

				 
				-10.476
				 

				  

				 
				-13.494
				 

				  

				 
				-28.192
				 

				  

				 
				10.980
				 

				  

				 
				13.449
				 

				  

				 
				-10.729
				 

				  

				 
				36.253
				 

				  

				 
				0.344
				 

				  
			 

			 
				 
				 
				 

				  

				 
				fitted
				 

				  

				 
				-31.025
				 

				  

				 
				-10.476
				 

				  

				 
				-13.494
				 

				  

				 
				-28.192
				 

				  

				 
				10.980
				 

				  

				 
				13.449
				 

				  

				 
				-10.729
				 

				  

				 
				36.253
				 

				  

				 
				0.344
				 

				  
			 

			 
				 
				 
				 

				  

				 
				residual
				 

				  

				 
				6.155e -7
				 

				  

				 
				1.550e -7
				 

				  

				 
				1.796e -7
				 

				  

				 
				-1.649e -7
				 

				  

				 
				7.254e -9
				 

				  

				 
				-1.691e -7
				 

				  

				 
				2.327e -7
				 

				  

				 
				0.000
				 

				  

				 
				0.000
				 

				  
			 

			 
				 
				RSQ
				 

				  

				 
				observed
				 

				  

				 
				-21.562
				 

				  

				 
				-7.657
				 

				  

				 
				-11.885
				 

				  

				 
				-18.641
				 

				  

				 
				10.911
				 

				  

				 
				13.426
				 

				  

				 
				-6.147
				 

				  

				 
				15.112
				 

				  

				 
				53.229
				 

				  
			 

			 
				 
				 
				 

				  

				 
				fitted
				 

				  

				 
				-14.494
				 

				  

				 
				-4.367
				 

				  

				 
				-9.879
				 

				  

				 
				-12.861
				 

				  

				 
				10.452
				 

				  

				 
				11.721
				 

				  

				 
				-4.472
				 

				  

				 
				15.112
				 

				  

				 
				53.229
				 

				  
			 

			 
				 
				 
				 

				  

				 
				residual
				 

				  

				 
				-7.068
				 

				  

				 
				-3.290
				 

				  

				 
				-2.005
				 

				  

				 
				-5.780
				 

				  

				 
				0.460
				 

				  

				 
				1.705
				 

				  

				 
				-1.675
				 

				  

				 
				0.000
				 

				  

				 
				0.000
				 

				  
			 

			 
				  
			 
		 

		  
	 
 
  The goal of structural equation modeling here is to test whether our theoretically motivated model of the covariance among variables provides a good approximation of the data. More specifically, we are trying to test how well a parsimonious model (composed of measurement and/or structural components) reproduces the observed covariance matrix. Formally, we are seeking to develop a model whose  model-implied covariance matrix  approaches the  sample (observed) covariance matrix .  
  The covarience matrix above provide evidence of a good fit.   We also computed residual covariance matrix (using R syntaxis)  resid(NewModel9, "cor") :  $type  [1] "cor.bollen"     $cov     RESIL SE   LOT  SC   STS  BU   CS   PSS  RSQ    RESIL 0.000                               SE   0.001 0.000                           LOT  0.008 0.013 0.000                        SC   0.002 0.001 0.009 0.000                    STS  -0.019 -0.026 -0.018 -0.011 0.000                 BU  -0.011 -0.020 -0.010 -0.011 0.011 0.000             CS   0.000 0.001 0.003 0.001 -0.017 -0.004 0.000          PSS  0.001 0.000 0.003 0.001 -0.001 -0.005 0.000 0.000      RSQ  -0.083 -0.117 -0.053 -0.088 0.013 0.042 -0.043 0.000 0.000  The residual covariance matrix indicates that we do not have significantly unrpredicted (positive value >.1) or significantly overpredicted (negative value >.1).       
 
 
	 
		 
			 
				 
					 
						 
							 
							Modification Indices
							 

							  

							  
						 
					 
				 
			 

			 
				 
				 
				 

				 
				 
				 

				 
				 
				 

				 
				mi
				 

				 
				epc
				 

				 
				sepc (lv)
				 

				 
				sepc (all)
				 

				 
				sepc (nox)
				 
			 
		 

		 
			 
				 
				SE
				 

				  

				 
				~
				 

				  

				 
				SC
				 

				  

				 
				4.475
				 

				  

				 
				2.224
				 

				  

				 
				2.224
				 

				  

				 
				5.299
				 

				  

				 
				5.299
				 

				  

			 

			 
				 
				SE
				 

				  

				 
				~
				 

				  

				 
				RESIL
				 

				  

				 
				4.459
				 

				  

				 
				1.576
				 

				  

				 
				1.576
				 

				  

				 
				4.732
				 

				  

				 
				4.732
				 

				  
			 

			 
				 
				SE
				 

				  

				 
				~
				 

				  

				 
				LOT
				 

				  

				 
				4.448
				 

				  

				 
				0.578
				 

				  

				 
				0.578
				 

				  

				 
				0.759
				 

				  

				 
				0.759
				 

				  
			 

			 
				 
				SE
				 

				  

				 
				~
				 

				  

				 
				BU
				 

				  

				 
				4.447
				 

				  

				 
				-0.402
				 

				  

				 
				-0.402
				 

				  

				 
				-0.525
				 

				  

				 
				-0.525
				 

				  
			 

			 
				 
				SE
				 

				  

				 
				~
				 

				  

				 
				RSQ
				 

				  

				 
				4.446
				 

				  

				 
				-0.052
				 

				  

				 
				-0.052
				 

				  

				 
				-0.103
				 

				  

				 
				-0.014
				 

				  
			 

			 
				 
				SE
				 

				  

				 
				~
				 

				  

				 
				STS
				 

				  

				 
				4.446
				 

				  

				 
				-0.418
				 

				  

				 
				-0.418
				 

				  

				 
				-0.504
				 

				  

				 
				-0.504
				 

				  
			 

			 
				 
				RSQ
				 

				  

				 
				~
				 

				  

				 
				SE
				 

				  

				 
				4.049
				 

				  

				 
				-0.178
				 

				  

				 
				-0.178
				 

				  

				 
				-0.091
				 

				  

				 
				-0.091
				 

				  
			 

			 
				 
				PSS
				 

				  

				 
				~
				 

				  

				 
				SE
				 

				  

				 
				3.243
				 

				  

				 
				0.341
				 

				  

				 
				0.341
				 

				  

				 
				0.211
				 

				  

				 
				0.211
				 

				  
			 

			 
				 
				RSQ
				 

				  

				 
				~
				 

				  

				 
				RESIL
				 

				  

				 
				3.089
				 

				  

				 
				-0.063
				 

				  

				 
				-0.063
				 

				  

				 
				-0.097
				 

				  

				 
				-0.097
				 

				  
			 

			 
				 
				PSS
				 

				  

				 
				~
				 

				  

				 
				RESIL
				 

				  

				 
				2.463
				 

				  

				 
				0.121
				 

				  

				 
				0.121
				 

				  

				 
				0.224
				 

				  

				 
				0.224
				 

				  
			 

			 
				 
				RSQ
				 

				  

				 
				~
				 

				  

				 
				LOT
				 

				  

				 
				2.235
				 

				  

				 
				-0.161
				 

				  

				 
				-0.161
				 

				  

				 
				-0.108
				 

				  

				 
				-0.108
				 

				  
			 

			 
				 
				RSQ
				 

				  

				 
				~
				 

				  

				 
				SC
				 

				  

				 
				2.173
				 

				  

				 
				-0.054
				 

				  

				 
				-0.054
				 

				  

				 
				-0.066
				 

				  

				 
				-0.066
				 

				  
			 

			 
				 
				PSS
				 

				  

				 
				~
				 

				  

				 
				LOT
				 

				  

				 
				1.952
				 

				  

				 
				0.337
				 

				  

				 
				0.337
				 

				  

				 
				0.274
				 

				  

				 
				0.274
				 

				  
			 

			 
				 
				PSS
				 

				  

				 
				~
				 

				  

				 
				SC
				 

				  

				 
				1.873
				 

				  

				 
				0.112
				 

				  

				 
				0.112
				 

				  

				 
				0.165
				 

				  

				 
				0.165
				 

				  
			 

			 
				 
				SC
				 

				  

				 
				~
				 

				  

				 
				RESIL
				 

				  

				 
				1.768
				 

				  

				 
				3.488
				 

				  

				 
				3.488
				 

				  

				 
				4.396
				 

				  

				 
				4.396
				 

				  
			 

			 
				 
				SC
				 

				  

				 
				~~
				 

				  

				 
				STS
				 

				  

				 
				1.760
				 

				  

				 
				9.924
				 

				  

				 
				9.924
				 

				  

				 
				0.331
				 

				  

				 
				0.331
				 

				  
			 

			 
				 
				SC
				 

				  

				 
				~
				 

				  

				 
				RSQ
				 

				  

				 
				1.760
				 

				  

				 
				-0.078
				 

				  

				 
				-0.078
				 

				  

				 
				-0.064
				 

				  

				 
				-0.009
				 

				  
			 

			 
				 
				SC
				 

				  

				 
				~
				 

				  

				 
				LOT
				 

				  

				 
				1.760
				 

				  

				 
				0.855
				 

				  

				 
				0.855
				 

				  

				 
				0.471
				 

				  

				 
				0.471
				 

				  
			 

			 
				 
				SC
				 

				  

				 
				~
				 

				  

				 
				BU
				 

				  

				 
				1.760
				 

				  

				 
				-0.861
				 

				  

				 
				-0.861
				 

				  

				 
				-0.471
				 

				  

				 
				-0.471
				 

				  
			 

			 
				 
				SC
				 

				  

				 
				~
				 

				  

				 
				SE
				 

				  

				 
				1.748
				 

				  

				 
				-5.622
				 

				  

				 
				-5.622
				 

				  

				 
				-2.359
				 

				  

				 
				-2.359
				 

				  
			 

			 
				 
				RSQ
				 

				  

				 
				~
				 

				  

				 
				BU
				 

				  

				 
				1.202
				 

				  

				 
				0.102
				 

				  

				 
				0.102
				 

				  

				 
				0.068
				 

				  

				 
				0.068
				 

				  
			 

			 
				 
				PSS
				 

				  

				 
				~
				 

				  

				 
				BU
				 

				  

				 
				1.167
				 

				  

				 
				-0.237
				 

				  

				 
				-0.237
				 

				  

				 
				-0.191
				 

				  

				 
				-0.191
				 

				  
			 

			 
				 
				RSQ
				 

				  

				 
				~
				 

				  

				 
				CS
				 

				  

				 
				0.645
				 

				  

				 
				-0.056
				 

				  

				 
				-0.056
				 

				  

				 
				-0.038
				 

				  

				 
				-0.038
				 

				  
			 

			 
				 
				PSS
				 

				  

				 
				~
				 

				  

				 
				CS
				 

				  

				 
				0.556
				 

				  

				 
				0.115
				 

				  

				 
				0.115
				 

				  

				 
				0.096
				 

				  

				 
				0.096
				 

				  
			 

			 
				 
				RSQ
				 

				  

				 
				~
				 

				  

				 
				STS
				 

				  

				 
				0.512
				 

				  

				 
				0.161
				 

				  

				 
				0.161
				 

				  

				 
				0.099
				 

				  

				 
				0.099
				 

				  
			 

			 
				 
				PSS
				 

				  

				 
				~
				 

				  

				 
				STS
				 

				  

				 
				0.181
				 

				  

				 
				-0.137
				 

				  

				 
				-0.137
				 

				  

				 
				-0.102
				 

				  

				 
				-0.102
				 

				  
			 

			 
				 
				STS
				 

				  

				 
				~
				 

				  

				 
				SC
				 

				  

				 
				0.105
				 

				  

				 
				0.021
				 

				  

				 
				0.021
				 

				  

				 
				0.041
				 

				  

				 
				0.041
				 

				  
			 

			 
				 
				LOT
				 

				  

				 
				~~
				 

				  

				 
				STS
				 

				  

				 
				0.073
				 

				  

				 
				-0.251
				 

				  

				 
				-0.251
				 

				  

				 
				-0.015
				 

				  

				 
				-0.015
				 

				  
			 

			 
				 
				LOT
				 

				  

				 
				~
				 

				  

				 
				STS
				 

				  

				 
				0.073
				 

				  

				 
				-0.016
				 

				  

				 
				-0.016
				 

				  

				 
				-0.014
				 

				  

				 
				-0.014
				 

				  
			 

			 
				 
				LOT
				 

				  

				 
				~
				 

				  

				 
				BU
				 

				  

				 
				0.072
				 

				  

				 
				-0.049
				 

				  

				 
				-0.049
				 

				  

				 
				-0.048
				 

				  

				 
				-0.048
				 

				  
			 

			 
				 
				LOT
				 

				  

				 
				~
				 

				  

				 
				SE
				 

				  

				 
				0.072
				 

				  

				 
				0.142
				 

				  

				 
				0.142
				 

				  

				 
				0.109
				 

				  

				 
				0.109
				 

				  
			 

			 
				 
				LOT
				 

				  

				 
				~
				 

				  

				 
				SC
				 

				  

				 
				0.072
				 

				  

				 
				0.083
				 

				  

				 
				0.083
				 

				  

				 
				0.150
				 

				  

				 
				0.150
				 

				  
			 

			 
				 
				LOT
				 

				  

				 
				~
				 

				  

				 
				RESIL
				 

				  

				 
				0.072
				 

				  

				 
				0.177
				 

				  

				 
				0.177
				 

				  

				 
				0.404
				 

				  

				 
				0.404
				 

				  
			 

			 
				 
				STS
				 

				  

				 
				~
				 

				  

				 
				LOT
				 

				  

				 
				0.036
				 

				  

				 
				-0.010
				 

				  

				 
				-0.010
				 

				  

				 
				-0.011
				 

				  

				 
				-0.011
				 

				  
			 

			 
				 
				CS
				 

				  

				 
				~
				 

				  

				 
				SE
				 

				  

				 
				0.019
				 

				  

				 
				0.080
				 

				  

				 
				0.080
				 

				  

				 
				0.059
				 

				  

				 
				0.059
				 

				  
			 

			 
				 
				STS
				 

				  

				 
				~~
				 

				  

				 
				CS
				 

				  

				 
				0.019
				 

				  

				 
				-0.141
				 

				  

				 
				-0.141
				 

				  

				 
				-0.008
				 

				  

				 
				-0.008
				 

				  
			 

			 
				 
				STS
				 

				  

				 
				~
				 

				  

				 
				CS
				 

				  

				 
				0.016
				 

				  

				 
				-0.006
				 

				  

				 
				-0.006
				 

				  

				 
				-0.007
				 

				  

				 
				-0.007
				 

				  
			 

			 
				 
				CS
				 

				  

				 
				~
				 

				  

				 
				SC
				 

				  

				 
				0.015
				 

				  

				 
				0.040
				 

				  

				 
				0.040
				 

				  

				 
				0.071
				 

				  

				 
				0.071
				 

				  
			 

			 
				 
				CS
				 

				  

				 
				~
				 

				  

				 
				STS
				 

				  

				 
				0.015
				 

				  

				 
				-0.008
				 

				  

				 
				-0.008
				 

				  

				 
				-0.007
				 

				  

				 
				-0.007
				 

				  
			 

			 
				 
				STS
				 

				  

				 
				~
				 

				  

				 
				BU
				 

				  

				 
				0.014
				 

				  

				 
				0.010
				 

				  

				 
				0.010
				 

				  

				 
				0.010
				 

				  

				 
				0.010
				 

				  
			 

			 
				 
				STS
				 

				  

				 
				~
				 

				  

				 
				SE
				 

				  

				 
				0.012
				 

				  

				 
				-0.019
				 

				  

				 
				-0.019
				 

				  

				 
				-0.016
				 

				  

				 
				-0.016
				 

				  
			 

			 
				 
				STS
				 

				  

				 
				~
				 

				  

				 
				RESIL
				 

				  

				 
				0.008
				 

				  

				 
				-0.003
				 

				  

				 
				-0.003
				 

				  

				 
				-0.008
				 

				  

				 
				-0.008
				 

				  
			 

			 
				 
				CS
				 

				  

				 
				~
				 

				  

				 
				RESIL
				 

				  

				 
				0.007
				 

				  

				 
				0.050
				 

				  

				 
				0.050
				 

				  

				 
				0.111
				 

				  

				 
				0.111
				 

				  
			 

			 
				 
				CS
				 

				  

				 
				~
				 

				  

				 
				BU
				 

				  

				 
				0.006
				 

				  

				 
				-0.013
				 

				  

				 
				-0.013
				 

				  

				 
				-0.012
				 

				  

				 
				-0.012
				 

				  
			 

			 
				 
				CS
				 

				  

				 
				~
				 

				  

				 
				LOT
				 

				  

				 
				0.003
				 

				  

				 
				-0.023
				 

				  

				 
				-0.023
				 

				  

				 
				-0.022
				 

				  

				 
				-0.022
				 

				  
			 

			 
				 
				CS
				 

				  

				 
				~
				 

				  

				 
				RSQ
				 

				  

				 
				0.003
				 

				  

				 
				0.002
				 

				  

				 
				0.002
				 

				  

				 
				0.003
				 

				  

				 
				4.167e 


				-4
				 

				  
			 

			 
				 
				RSQ
				 

				  

				 
				~~
				 

				  

				 
				RSQ
				 

				  

				 
				3.713e -29
				 

				  

				 
				3.713e -29
				 

				  

				 
				0.000
				 

				  

				 
				0.000
				 

				  

				 
				0.000
				 

				  
			 

			 
				 
				PSS
				 

				  

				 
				~~
				 

				  

				 
				RSQ
				 

				  

				 
				4.499e -31
				 

				  

				 
				4.499e -31
				 

				  

				 
				4.499e -31
				 

				  

				  

				  

				 
				4.499e -31
				 

				  
			 

			 
				 
				PSS
				 

				  

				 
				~~
				 

				  

				 
				PSS
				 

				  

				 
				4.417e -31
				 

				  

				 
				4.417e -31
				 

				  

				 
				0.000
				 

				  

				 
				0.000
				 

				  

				 
				0.000
				 

				  
			 

			 
				 
				PSS
				 

				  

				 
				~
				 

				  

				 
				RSQ
				 

				  

				 
				9.459e -32
				 

				  

				 
				-2.097e -17
				 

				  

				 
				-2.097e -17
				 

				  

				 
				-2.541e -17
				 

				  

				 
				-3.483e -18
				 

				  
			 

			 
				 
				RSQ
				 

				  

				 
				~
				 

				  

				 
				PSS
				 

				  

				 
				1.300e -32
				 

				  

				 
				9.790e -18
				 

				  

				 
				9.790e -18
				 

				  

				 
				8.079e -18
				 

				  

				 
				1.342e -18
				 

				  
			 

			 
				  
			 
		 

		  
	 
 
  The modification indices table above does not provide evidence that some of the paths should be freeing.   It has to be noted that instead of using the default 'delta method' for testing mediation (which is known to be problematic because the sampling distribution of the inderect path product term is not normal), we used bootstrapping. Bootstrapping is a common workaround for the debate in the literature about estimates for indirect paths as it does not make strong assumptions about the distribution of the coefficient of interst (i.e., the sampling distributions of the mediated paths). As all indirect paths are significant, we can conclude that these is evidence for mediation.           	 
 
