# Supplementary material for: Individual factors in the relationship between stress and resilience in mental health psychology practitioners during the COVID-19 pandemic
Source: J Health Psychol. 2021 Dec 7;27(11):2613–31. doi: 10.1177/13591053211059393 (PMC9483698; doi:10.1177/13591053211059393)
Supplement: sj-jasp-3-hpq-10.1177_13591053211059393 – Supplemental material for Individual factors in the relationship between stress and resilience in mental health psychology practitioners during the COVID-19 pandemic [file sj-jasp-3-hpq-10.1177_13591053211059393.jasp › index.html]

JASP 


# Results

Prior testing our mediation models, we examined the relationship between perceived stress and resilience. Specifically, we tested whether perceived stress can predict resilience in psychologists-practitioners.

In the present study, we measured perceived stress using two measurements. One of them is PSS (Perceived stress scale) is a measure of the degree to which situations in one’s life are appraised as stressful during the last month.  the questions are of a general nature and hence are relatively free of content specific to any subpopulation group. The second measure is RSQ. This questionnaire was designed to assess individuals’ involuntary stress reactions to the COVID-19 pandemic.

These two measurements of stress are our predictors and correlate with each other. As both measurements were taken during the lockdown period, it is difficult to clearly separate their the effects on resilience. Therefore, we, first, tested three simple linear models:Regression model 1 (RSQ as predictor), Regression model 2 (PSS as predictor) and Regression model 3 (both RSQ and PSS as predictors).

## Bayesian Correlation between all variables

Prior testing our mediation model, we assessed associations between variables in the present study.

| Bayesian Pearson Correlations | | | | | | | | | | | | | | | | | | | | | | | | | |
| --- | --- | --- | --- | --- | --- | --- | --- | --- | --- | --- | --- | --- | --- | --- | --- | --- | --- | --- | --- | --- | --- | --- | --- | --- | --- |
| Variable | |  | | RESIL | | PSS | | RSQ | | SE | | LOT | | AV | | AP | | SC | | CS | | BU | | STS | |
| 1. RESIL |  | Pearson's r |  | — |  |  |  |  |  |  |  |  |  |  |  |  |  |  |  |  |  |  |  |  |  |
|  |  | BF₁₀ |  | — |  |  |  |  |  |  |  |  |  |  |  |  |  |  |  |  |  |  |  |  |  |
|  |  | Upper 95% CI |  | — |  |  |  |  |  |  |  |  |  |  |  |  |  |  |  |  |  |  |  |  |  |
|  |  | Lower 95% CI |  | — |  |  |  |  |  |  |  |  |  |  |  |  |  |  |  |  |  |  |  |  |  |
| 2. PSS |  | Pearson's r |  | -0.460 | \*\*\* | — |  |  |  |  |  |  |  |  |  |  |  |  |  |  |  |  |  |  |  |
|  |  | BF₁₀ |  | 2.688e +15 |  | — |  |  |  |  |  |  |  |  |  |  |  |  |  |  |  |  |  |  |  |
|  |  | Upper 95% CI |  | -0.368 |  | — |  |  |  |  |  |  |  |  |  |  |  |  |  |  |  |  |  |  |  |
|  |  | Lower 95% CI |  | -0.540 |  | — |  |  |  |  |  |  |  |  |  |  |  |  |  |  |  |  |  |  |  |
| 3. RSQ |  | Pearson's r |  | -0.264 | \*\*\* | 0.344 | \*\*\* | — |  |  |  |  |  |  |  |  |  |  |  |  |  |  |  |  |  |
|  |  | BF₁₀ |  | 7510.235 |  | 4.188e  +7 |  | — |  |  |  |  |  |  |  |  |  |  |  |  |  |  |  |  |  |
|  |  | Upper 95% CI |  | -0.159 |  | 0.435 |  | — |  |  |  |  |  |  |  |  |  |  |  |  |  |  |  |  |  |
|  |  | Lower 95% CI |  | -0.361 |  | 0.243 |  | — |  |  |  |  |  |  |  |  |  |  |  |  |  |  |  |  |  |
| 4. SE |  | Pearson's r |  | 0.657 | \*\*\* | -0.467 | \*\*\* | -0.282 | \*\*\* | — |  |  |  |  |  |  |  |  |  |  |  |  |  |  |  |
|  |  | BF₁₀ |  | 1.553e +38 |  | 1.070e +16 |  | 41349.019 |  | — |  |  |  |  |  |  |  |  |  |  |  |  |  |  |  |
|  |  | Upper 95% CI |  | 0.713 |  | -0.376 |  | -0.178 |  | — |  |  |  |  |  |  |  |  |  |  |  |  |  |  |  |
|  |  | Lower 95% CI |  | 0.588 |  | -0.547 |  | -0.378 |  | — |  |  |  |  |  |  |  |  |  |  |  |  |  |  |  |
| 5. LOT |  | Pearson's r |  | 0.563 | \*\*\* | -0.455 | \*\*\* | -0.331 | \*\*\* | 0.468 | \*\*\* | — |  |  |  |  |  |  |  |  |  |  |  |  |  |
|  |  | BF₁₀ |  | 2.480e +25 |  | 1.070e +15 |  | 8.403e  +6 |  | 1.117e +16 |  | — |  |  |  |  |  |  |  |  |  |  |  |  |  |
|  |  | Upper 95% CI |  | 0.631 |  | -0.363 |  | -0.229 |  | 0.547 |  | — |  |  |  |  |  |  |  |  |  |  |  |  |  |
|  |  | Lower 95% CI |  | 0.482 |  | -0.536 |  | -0.423 |  | 0.376 |  | — |  |  |  |  |  |  |  |  |  |  |  |  |  |
| 6. AV |  | Pearson's r |  | -0.305 | \*\*\* | 0.367 | \*\*\* | 0.430 | \*\*\* | -0.334 | \*\*\* | -0.275 | \*\*\* | — |  |  |  |  |  |  |  |  |  |  |  |
|  |  | BF₁₀ |  | 434967.892 |  | 8.325e  +8 |  | 1.092e +13 |  | 1.138e  +7 |  | 20576.463 |  | — |  |  |  |  |  |  |  |  |  |  |  |
|  |  | Upper 95% CI |  | -0.202 |  | 0.456 |  | 0.513 |  | -0.232 |  | -0.170 |  | — |  |  |  |  |  |  |  |  |  |  |  |
|  |  | Lower 95% CI |  | -0.399 |  | 0.268 |  | 0.335 |  | -0.426 |  | -0.371 |  | — |  |  |  |  |  |  |  |  |  |  |  |
| 7. AP |  | Pearson's r |  | 0.351 | \*\*\* | -0.027 |  | 0.085 |  | 0.177 | \* | 0.255 | \*\*\* | 0.222 | \*\*\* | — |  |  |  |  |  |  |  |  |  |
|  |  | BF₁₀ |  | 9.824e  +7 |  | 0.078 |  | 0.220 |  | 11.167 |  | 3354.762 |  | 220.786 |  | — |  |  |  |  |  |  |  |  |  |
|  |  | Upper 95% CI |  | 0.442 |  | 0.082 |  | 0.191 |  | 0.279 |  | 0.353 |  | 0.321 |  | — |  |  |  |  |  |  |  |  |  |
|  |  | Lower 95% CI |  | 0.251 |  | -0.135 |  | -0.024 |  | 0.069 |  | 0.150 |  | 0.115 |  | — |  |  |  |  |  |  |  |  |  |
| 8. SC |  | Pearson's r |  | 0.596 | \*\*\* | -0.527 | \*\*\* | -0.288 | \*\*\* | 0.469 | \*\*\* | 0.547 | \*\*\* | -0.403 | \*\*\* | 0.227 | \*\*\* | — |  |  |  |  |  |  |  |
|  |  | BF₁₀ |  | 2.501e +29 |  | 3.673e +21 |  | 73985.916 |  | 1.390e +16 |  | 4.674e +23 |  | 1.413e +11 |  | 326.333 |  | — |  |  |  |  |  |  |  |
|  |  | Upper 95% CI |  | 0.660 |  | -0.442 |  | -0.184 |  | 0.548 |  | 0.618 |  | -0.306 |  | 0.326 |  | — |  |  |  |  |  |  |  |
|  |  | Lower 95% CI |  | 0.519 |  | -0.600 |  | -0.383 |  | 0.378 |  | 0.464 |  | -0.489 |  | 0.120 |  | — |  |  |  |  |  |  |  |
| 9. CS |  | Pearson's r |  | 0.565 | \*\*\* | -0.355 | \*\*\* | -0.168 |  | 0.408 | \*\*\* | 0.334 | \*\*\* | -0.101 |  | 0.272 | \*\*\* | 0.383 | \*\*\* | — |  |  |  |  |  |
|  |  | BF₁₀ |  | 4.548e +25 |  | 1.614e  +8 |  | 6.755 |  | 3.336e +11 |  | 1.277e  +7 |  | 0.354 |  | 14667.284 |  | 8.144e  +9 |  | — |  |  |  |  |  |
|  |  | Upper 95% CI |  | 0.633 |  | -0.254 |  | -0.060 |  | 0.493 |  | 0.426 |  | 0.009 |  | 0.368 |  | 0.471 |  | — |  |  |  |  |  |
|  |  | Lower 95% CI |  | 0.484 |  | -0.445 |  | -0.270 |  | 0.311 |  | 0.233 |  | -0.206 |  | 0.167 |  | 0.285 |  | — |  |  |  |  |  |
| 10. BU |  | Pearson's r |  | -0.621 | \*\*\* | 0.455 | \*\*\* | 0.375 | \*\*\* | -0.451 | \*\*\* | -0.494 | \*\*\* | 0.325 | \*\*\* | -0.249 | \*\*\* | -0.528 | \*\*\* | -0.666 | \*\*\* | — |  |  |  |
|  |  | BF₁₀ |  | 6.272e +32 |  | 1.061e +15 |  | 2.548e  +9 |  | 4.522e +14 |  | 2.041e +18 |  | 4.099e  +6 |  | 1923.237 |  | 4.419e +21 |  | 4.543e +39 |  | — |  |  |  |
|  |  | Upper 95% CI |  | -0.547 |  | 0.536 |  | 0.463 |  | -0.358 |  | -0.405 |  | 0.418 |  | -0.143 |  | -0.443 |  | -0.598 |  | — |  |  |  |
|  |  | Lower 95% CI |  | -0.682 |  | 0.363 |  | 0.276 |  | -0.531 |  | -0.570 |  | 0.223 |  | -0.347 |  | -0.601 |  | -0.721 |  | — |  |  |  |
| 11. STS |  | Pearson's r |  | -0.239 | \*\*\* | 0.406 | \*\*\* | 0.333 | \*\*\* | -0.321 | \*\*\* | -0.230 | \*\*\* | 0.361 | \*\*\* | 4.978e -4 |  | -0.305 | \*\*\* | -0.159 |  | 0.468 | \*\*\* | — |  |
|  |  | BF₁₀ |  | 858.561 |  | 2.407e +11 |  | 1.053e  +7 |  | 2.745e  +6 |  | 431.443 |  | 3.755e  +8 |  | 0.070 |  | 464121.384 |  | 4.219 |  | 1.292e +16 |  | — |  |
|  |  | Upper 95% CI |  | -0.133 |  | 0.491 |  | 0.425 |  | -0.219 |  | -0.124 |  | 0.451 |  | 0.109 |  | -0.202 |  | -0.051 |  | 0.547 |  | — |  |
|  |  | Lower 95% CI |  | -0.337 |  | 0.309 |  | 0.231 |  | -0.414 |  | -0.329 |  | 0.262 |  | -0.108 |  | -0.399 |  | -0.262 |  | 0.377 |  | — |  |
|  | | | | | | | | | | | | | | | | | | | | | | | | | |
|  |  |  |  |  |  |  |  |  |  |  |  |  |  |  |  |  |  |  |  |  |  |  |  |  |  |
| --- | --- | --- | --- | --- | --- | --- | --- | --- | --- | --- | --- | --- | --- | --- | --- | --- | --- | --- | --- | --- | --- | --- | --- | --- | --- |
| \*  BF₁₀ > 10, \*\* BF₁₀ > 30, \*\*\* BF₁₀ > 100 | | | | | | | | | | | | | | | | | | | | | | | | | |

The results indicate medium to strong correlations between all variables excluding associations between AP and PSS (r=-0.03, BF10 =0.08, 95% CI [0.08, -0.14]); AP and RSQ (r=0.09, BF10 =0.22, 95%CI [0.19, -0.02]), RSQ and CS (r=-0.17, BF10 =6.76, 95% CI [-0.06, -0.27]), AV and CS (r=0.10, BF10 =0.35, 95% CI [0.01, -0.21])

## Regression model 1. RSQ as predictor for RESIL

Predictor: RSQ, outcome: Resilience (RESIL)

| Model Summary - RESIL | | | | | | | | | |
| --- | --- | --- | --- | --- | --- | --- | --- | --- | --- |
| Model | | R | | R² | | Adjusted R² | | RMSE | |
| H₀ |  | 0.000 |  | 0.000 |  | 0.000 |  | 11.215 |  |
| H₁ |  | 0.264 |  | 0.070 |  | 0.067 |  | 10.834 |  |
|  | | | | | | | | | |

RSQ can explain only 6.7 of variance of resilience

| ANOVA | | | | | | | | | | | | | |
| --- | --- | --- | --- | --- | --- | --- | --- | --- | --- | --- | --- | --- | --- |
| Model | |  | | Sum of Squares | | df | | Mean Square | | F | | p | |
| H₁ |  | Regression |  | 2838.763 |  | 1 |  | 2838.763 |  | 24.187 |  | < .001 |  |
|  |  | Residual |  | 37909.464 |  | 323 |  | 117.367 |  |  |  |  |  |
|  |  | Total |  | 40748.228 |  | 324 |  |  |  |  |  |  |  |
|  | | | | | | | | | | | | | |
|  |  |  |  |  |  |  |  |  |  |  |  |  |  |
| --- | --- | --- | --- | --- | --- | --- | --- | --- | --- | --- | --- | --- | --- |
| *Note.*  The intercept model is omitted, as no meaningful information can be shown. | | | | | | | | | | | | | |

| Coefficients | | | | | | | | | | | | | | | | | |
| --- | --- | --- | --- | --- | --- | --- | --- | --- | --- | --- | --- | --- | --- | --- | --- | --- | --- |
|  | | | | | | | | | | | | | | 95% CI | | | |
| Model | |  | | Unstandardized | | Standard Error | | Standardized | | t | | p | | Lower | | Upper | |
| H₀ |  | (Intercept) |  | 0.004 |  | 0.622 |  |  |  | 0.006 |  | 0.995 |  | -1.220 |  | 1.228 |  |
| H₁ |  | (Intercept) |  | 0.007 |  | 0.601 |  |  |  | 0.011 |  | 0.991 |  | -1.176 |  | 1.189 |  |
|  |  | RSQ |  | -0.405 |  | 0.082 |  | -0.264 |  | -4.918 |  | < .001 |  | -0.567 |  | -0.243 |  |
|  | | | | | | | | | | | | | | | | | |

### Residuals vs. Covariates

All plots below indicate that there is no systematic errors and big outliers. A Q-Q plot shows the quantiles of a theoretical normal distribution against the observed quantiles of the residuals. If the observed residuals are approximately normal, then all points in the plot fall approximately on a straight line. In our case, the assumptions are not badly violated.

#### Residuals vs. RSQ

### Residuals vs. Predicted

### Standardized Residuals Histogram

### Q-Q Plot Standardized Residuals

### Partial Regression Plot

#### RESIL vs. RSQ

## Regression model 2. PSS as predictor for RESIL

Predictor: PSS, outcome RESIL

| Model Summary - RESIL | | | | | | | | | |
| --- | --- | --- | --- | --- | --- | --- | --- | --- | --- |
| Model | | R | | R² | | Adjusted R² | | RMSE | |
| H₀ |  | 0.000 |  | 0.000 |  | 0.000 |  | 11.215 |  |
| H₁ |  | 0.460 |  | 0.212 |  | 0.209 |  | 9.972 |  |
|  | | | | | | | | | |

PSS can explain 21% of variance of resilience

| ANOVA | | | | | | | | | | | | | |
| --- | --- | --- | --- | --- | --- | --- | --- | --- | --- | --- | --- | --- | --- |
| Model | |  | | Sum of Squares | | df | | Mean Square | | F | | p | |
| H₁ |  | Regression |  | 8628.878 |  | 1 |  | 8628.878 |  | 86.774 |  | < .001 |  |
|  |  | Residual |  | 32119.350 |  | 323 |  | 99.441 |  |  |  |  |  |
|  |  | Total |  | 40748.228 |  | 324 |  |  |  |  |  |  |  |
|  | | | | | | | | | | | | | |
|  |  |  |  |  |  |  |  |  |  |  |  |  |  |
| --- | --- | --- | --- | --- | --- | --- | --- | --- | --- | --- | --- | --- | --- |
| *Note.*  The intercept model is omitted, as no meaningful information can be shown. | | | | | | | | | | | | | |

| Coefficients | | | | | | | | | | | | | | | | | |
| --- | --- | --- | --- | --- | --- | --- | --- | --- | --- | --- | --- | --- | --- | --- | --- | --- | --- |
|  | | | | | | | | | | | | | | 95% CI | | | |
| Model | |  | | Unstandardized | | Standard Error | | Standardized | | t | | p | | Lower | | Upper | |
| H₀ |  | (Intercept) |  | 0.004 |  | 0.622 |  |  |  | 0.006 |  | 0.995 |  | -1.220 |  | 1.228 |  |
| H₁ |  | (Intercept) |  | 0.006 |  | 0.553 |  |  |  | 0.011 |  | 0.992 |  | -1.082 |  | 1.094 |  |
|  |  | PSS |  | -0.856 |  | 0.092 |  | -0.460 |  | -9.315 |  | < .001 |  | -1.037 |  | -0.675 |  |
|  | | | | | | | | | | | | | | | | | |

### Residuals vs. Covariates

assumption are not badly violated (see plots below)

#### Residuals vs. PSS

### Residuals vs. Predicted

### Standardized Residuals Histogram

### Q-Q Plot Standardized Residuals

### Partial Regression Plot

#### RESIL vs. PSS

## Regression Model 3. RSQ & PSS as predictors for RESIL

Predictors: RSQ and PSS. Outcome RESIL

| Model Summary - RESIL | | | | | | | | | |
| --- | --- | --- | --- | --- | --- | --- | --- | --- | --- |
| Model | | R | | R² | | Adjusted R² | | RMSE | |
| H₀ |  | 0.000 |  | 0.000 |  | 0.000 |  | 11.215 |  |
| H₁ |  | 0.474 |  | 0.224 |  | 0.220 |  | 9.907 |  |
|  | | | | | | | | | |

a model with two predictors can explain 22% of the variance of resilience

| ANOVA | | | | | | | | | | | | | |
| --- | --- | --- | --- | --- | --- | --- | --- | --- | --- | --- | --- | --- | --- |
| Model | |  | | Sum of Squares | | df | | Mean Square | | F | | p | |
| H₁ |  | Regression |  | 9144.634 |  | 2 |  | 4572.317 |  | 46.586 |  | < .001 |  |
|  |  | Residual |  | 31603.594 |  | 322 |  | 98.148 |  |  |  |  |  |
|  |  | Total |  | 40748.228 |  | 324 |  |  |  |  |  |  |  |
|  | | | | | | | | | | | | | |
|  |  |  |  |  |  |  |  |  |  |  |  |  |  |
| --- | --- | --- | --- | --- | --- | --- | --- | --- | --- | --- | --- | --- | --- |
| *Note.*  The intercept model is omitted, as no meaningful information can be shown. | | | | | | | | | | | | | |

| Coefficients | | | | | | | | | | | | | | | | | |
| --- | --- | --- | --- | --- | --- | --- | --- | --- | --- | --- | --- | --- | --- | --- | --- | --- | --- |
|  | | | | | | | | | | | | | | 95% CI | | | |
| Model | |  | | Unstandardized | | Standard Error | | Standardized | | t | | p | | Lower | | Upper | |
| H₀ |  | (Intercept) |  | 0.004 |  | 0.622 |  |  |  | 0.006 |  | 0.995 |  | -1.220 |  | 1.228 |  |
| H₁ |  | (Intercept) |  | 0.007 |  | 0.550 |  |  |  | 0.013 |  | 0.990 |  | -1.074 |  | 1.088 |  |
|  |  | RSQ |  | -0.184 |  | 0.080 |  | -0.120 |  | -2.292 |  | 0.023 |  | -0.342 |  | -0.026 |  |
|  |  | PSS |  | -0.779 |  | 0.097 |  | -0.419 |  | -8.016 |  | < .001 |  | -0.970 |  | -0.588 |  |
|  | | | | | | | | | | | | | | | | | |

### Residuals vs. Covariates

#### Residuals vs. RSQ

#### Residuals vs. PSS

### Residuals vs. Predicted

### Standardized Residuals Histogram

### Q-Q Plot Standardized Residuals

### Partial Regression Plots

#### RESIL vs. RSQ

#### RESIL vs. PSS

At first glance, the Regression model 3 can better account for the variance in resilience. However, the coefficient of determination R2 is not a good measure for model comparison because it does not penalize models for complexity: when additional predictors are added to a model, R2 can only increase. Therefore, R2 will always favor the most complex model. This makes R2 unsuitable for model selection, unless models have the same number of predictors. Therefore, we will use Bayeasin Inference for model selection.

## Bayesian Inference for model selection RSQ and PSS as predictors of RESIL)

When multiple models are in play, we can extend Bayes’ theorem and use the data to update the relative plausibility of each of the candidate models.

As we have no previous studies that could indicate us prior believes, we will assign default priors (i.e., the relative plausibility of models before seeing the data) . The change from prior to posterior odds (i.e., the relative plausibility of models after seeing the data) is given by the Bayes factor (e.g., Jeffreys, 1961; Kass & Raftery, 1995), which indicates the models’ relative predictive performance for the data at hand (i.e., the ratio of marginal likelihoods).

 Using default priors:  we used the Jeffreys–Zellner–Siow (JZS) prior. The JZS prior fulfills several desiderata (Rouder & Morey, 2012;  Ly, Verhagen, & Wagenmakers, 2016). Moreover, the sample size for our data is 324, which is relatively big. In the case of the big dataset, the influence of the prior is relatively small.

As we have only 2 predictors, we will compare three possible models (PSS), (RSQ) and (PSS+RSQ) with the null model.

| Model Comparison - RESIL | | | | | | | | | | | |
| --- | --- | --- | --- | --- | --- | --- | --- | --- | --- | --- | --- |
| Models | | P(M) | | P(M|data) | | BF M | | BF 10 | | R² | |
| Null model |  | 0.250 |  | 1.849e -16 |  | 5.547e -16 |  | 1.000 |  | 0.000 |  |
| RSQ + PSS |  | 0.250 |  | 0.587 |  | 4.266 |  | 3.175e +15 |  | 0.224 |  |
| PSS |  | 0.250 |  | 0.413 |  | 2.110 |  | 2.233e +15 |  | 0.212 |  |
| RSQ |  | 0.250 |  | 1.757e -12 |  | 5.270e -12 |  | 9501.186 |  | 0.070 |  |
|  | | | | | | | | | | | |

The Bayes factor BF10 (H1) for model with both predictors is large and indicates that the data are 3.15e+15 times more likely under (RSQ + PSS) model compared to the null model (H0). However, the Bayes factor for two other models with only one predictor (PSS or RSQ) is also large (BF10 >100) that indicated strong evidence in favour of the H1 hypothesis. Therefore, the model comparison statistics are not informative in this case.

We will then asses the Posterior Summary table which quantifies the relevance of individual predictors.

### Posterior Summary

**Model-averaged posterior summary for linear regression coefficients**

Short annotation to the table:

**Coefficient** - predictors

**Mean and SD** - represent the respective posterior mean and standard deviation of the parameter after model averaging

**P (incl)** denotes the prior inclusion probability

**P (incl | data)** denotes the posterior inclusion probability

**BFinclusion** - the change from prior to posterior inclusion odds

a **95% central credible interval** (CI) for the parameters

The results indicate that each of these predictors are relevant for predicting resilience, as indicated by the fact that the posterior inclusion probabilities for PSS is =1.00 and for RSQ = 0.74. Although, the posterior inclusion probability for RSQ is not as high as we would expect\*, the relevance of this predictor is evident bacause the data increased the inclusion probability from 0.5 to 0.74 (see Inclusion Probabilities plot below. The dashed line represents the prior inclusion probabilities ).

The Q-Q plot (below) shows that the standardized residuals fit fairly well along the diagonal suggesting that both assumptions or normality and linearity have also not been violated.

\*The inclusion Bayes factor quantifies how much the observed data are more probable under models that include a particular predictor relative to the models that do not contain that particular predictor.  In case of RSQ, across all the candidate models, the model with the RSQ variable is, on average, about only 1.42 times more likely than the model without the RSQ variable.

| Posterior Summaries of Coefficients | | | | | | | | | | | | | | | |
| --- | --- | --- | --- | --- | --- | --- | --- | --- | --- | --- | --- | --- | --- | --- | --- |
|  | | | | | | | | | | | | 95% Credible Interval | | | |
| Coefficient | | Mean | | SD | | P(incl) | | P(incl|data) | | BF inclusion | | Lower | | Upper | |
| Intercept |  | 0.004 |  | 0.550 |  | 1.000 |  | 1.000 |  | 1.000 |  | -1.077 |  | 1.085 |  |
| RSQ |  | -0.180 |  | 0.079 |  | 0.500 |  | 0.587 |  | 1.422 |  | -0.336 |  | -0.024 |  |
| PSS |  | -0.762 |  | 0.096 |  | 0.500 |  | 1.000 |  | 5.692e +11 |  | -0.951 |  | -0.573 |  |
|  | | | | | | | | | | | | | | | |

The posterior mean of the regression coefficient of RSQ is -0.18. We can interpret this value such that a one-unit increase in RSQ adds about 0.18 units in decreasing resilience. The 95% credible interval of RSQ is [-0.34, -0.02], which means that there is a 95% probability that the regression coefficient of RSQ lies in the population with the corresponding credible interval. The 95% credible interval does not contain 0. This shows the evidence of the effect of RSQ in predicting the level of resilience.

The posterior mean of the regression coefficient of PSS is -0.76 indicating that a one-unit increase in PSS adds about 0.76 units in decreasing resilience. The 95% credible interval of PSS is [-0.95, -0.57], which means that there is a 95% probability that the regression coefficient of PSS lies in the population with the corresponding credible interval. The 95% credible interval does not contain 0. This shows the evidence of the effect of PSS in predicting the level of resilience.

### Inclusion Probabilities

### Q-Q Plot

### Marginal Posterior Distributions

The complete model-averaged posteriors are be visualized below. The pike at zero in RSQ plot corresponds to the absence of an effect, and its height reflects the predictor’s posterior exclusion probability. The horizontal bars depicted a 95% credible interval for each predictor.

Following recommendations by van Doorn at al (2019), we further investigated the robustness of the results against the choice of prior by using wide and ultrawide priors. Therefore, we repeated this analysis using scale of 1/4 and 1/2 but the result did not change in a meaningful way in both cases.

#### Intercept

#### RSQ

#### PSS

To summarize, the Bayesian model-averaged analysis showed that the most important predictor of resilience is the PSS. However, we cannot fully exclude the RSQ as there is still relevance for predicting the level of resilience. A one-unit increase in RSQ adds about 0.18 units in decreasing resilience. A one-unit increase in PSS adds about 0.76 units in decreasing resilience. Therefore, this analysis shows the evidence of the effects of both PSS and RSQ in predicting the level of resilience.

## RSQ and PSS as predictors of SE

We next tested ther relationship between either predictors (RSQ and PSS) and potential mediators (i.e., AV, AP, SE, LOT, SC, STS, BU, CS) We, first, tested whether RSQ and PSS could predict AV, AP, SE, LOT, SC, STS, BU, CS using a multiple regression analysis to gather an idea which independent variables will create the best prediction equation. We performed a seria of regression analyses with each of possible mediators as a dependent variable and RSQ and PSS as predictors. Each analysis we supplemented with Bayeasian inferences to gather evidence of each prediction.

In summary, the analyses below showed that

1. RSQ and PSS are not reliable predictors of AP
2. RSQ is not reliable predictors of SC and CS while PSS could reliably predict these variables

| Model Summary - SE | | | | | | | | | | | | | | | | | | | | | | | | | |
| --- | --- | --- | --- | --- | --- | --- | --- | --- | --- | --- | --- | --- | --- | --- | --- | --- | --- | --- | --- | --- | --- | --- | --- | --- | --- |
|  | | | | | | | | | | | | | | | | | | | | Durbin-Watson | | | | | |
| Model | | R | | R² | | Adjusted R² | | RMSE | | R² Change | | F Change | | df1 | | df2 | | p | | Autocorrelation | | Statistic | | p | |
| H₀ |  | 0.000 |  | 0.000 |  | 0.000 |  | 3.728 |  | 0.000 |  |  |  | 0 |  | 324 |  |  |  | -0.049 |  | 2.098 |  | 0.374 |  |
| H₁ |  | 0.485 |  | 0.235 |  | 0.230 |  | 3.270 |  | 0.235 |  | 49.503 |  | 2 |  | 322 |  | < .001 |  | -0.059 |  | 2.116 |  | 0.296 |  |
|  | | | | | | | | | | | | | | | | | | | | | | | | | |

The adjusted R2 (we are using the adjusted value for multiple predictors) shows that the predictors can explain 23%% of the outcome variance.

Durbin-Watson checks for correlations between residuals is between 1 and 3 as required.

| ANOVA | | | | | | | | | | | | | |
| --- | --- | --- | --- | --- | --- | --- | --- | --- | --- | --- | --- | --- | --- |
| Model | |  | | Sum of Squares | | df | | Mean Square | | F | | p | |
| H₁ |  | Regression |  | 1058.871 |  | 2 |  | 529.436 |  | 49.503 |  | < .001 |  |
|  |  | Residual |  | 3443.818 |  | 322 |  | 10.695 |  |  |  |  |  |
|  |  | Total |  | 4502.689 |  | 324 |  |  |  |  |  |  |  |
|  | | | | | | | | | | | | | |
|  |  |  |  |  |  |  |  |  |  |  |  |  |  |
| --- | --- | --- | --- | --- | --- | --- | --- | --- | --- | --- | --- | --- | --- |
| *Note.*  The intercept model is omitted, as no meaningful information can be shown. | | | | | | | | | | | | | |

| Coefficients | | | | | | | | | | | | | | | | | | | | | |
| --- | --- | --- | --- | --- | --- | --- | --- | --- | --- | --- | --- | --- | --- | --- | --- | --- | --- | --- | --- | --- | --- |
|  | | | | | | | | | | | | | | 95% CI | | | | Collinearity Statistics | | | |
| Model | |  | | Unstandardized | | Standard Error | | Standardized | | t | | p | | Lower | | Upper | | Tolerance | | VIF | |
| H₀ |  | (Intercept) |  | 0.018 |  | 0.207 |  |  |  | 0.089 |  | 0.929 |  | -0.388 |  | 0.425 |  |  |  |  |  |
| H₁ |  | (Intercept) |  | 0.020 |  | 0.181 |  |  |  | 0.108 |  | 0.914 |  | -0.337 |  | 0.376 |  |  |  |  |  |
|  |  | PSS |  | -0.260 |  | 0.032 |  | -0.420 |  | -8.095 |  | < .001 |  | -0.323 |  | -0.197 |  | 0.882 |  | 1.134 |  |
|  |  | RSQ |  | -0.070 |  | 0.026 |  | -0.137 |  | -2.647 |  | 0.009 |  | -0.122 |  | -0.018 |  | 0.882 |  | 1.134 |  |
|  | | | | | | | | | | | | | | | | | | | | | |

Both tolerance and VIF are acceptable.

| Collinearity Diagnostics | | | | | | | | | | | | | |
| --- | --- | --- | --- | --- | --- | --- | --- | --- | --- | --- | --- | --- | --- |
|  | | | | | | | | Variance Proportions | | | | | |
| Model | | Dimension | | Eigenvalue | | Condition Index | | (Intercept) | | PSS | | RSQ | |
| H₁ |  | 1 |  | 1.344 |  | 1.000 |  | 0.000 |  | 0.328 |  | 0.328 |  |
|  |  | 2 |  | 1.000 |  | 1.159 |  | 1.000 |  | 0.000 |  | 0.000 |  |
|  |  | 3 |  | 0.656 |  | 1.431 |  | 0.000 |  | 0.672 |  | 0.672 |  |
|  | | | | | | | | | | | | | |
|  |  |  |  |  |  |  |  |  |  |  |  |  |  |
| --- | --- | --- | --- | --- | --- | --- | --- | --- | --- | --- | --- | --- | --- |
| *Note.*  The intercept model is omitted, as no meaningful information can be shown. | | | | | | | | | | | | | |

| Casewise Diagnostics | | | | | | | | | | | |
| --- | --- | --- | --- | --- | --- | --- | --- | --- | --- | --- | --- |
| Case Number | | Std. Residual | | SE | | Predicted Value | | Residual | | Cook's Distance | |
| 97 |  | -3.956 |  | -18.600 |  | -5.952 |  | -12.648 |  | 0.240 |  |
|  | | | | | | | | | | | |

The casewise diagnostic indicates that our predictor (RSQ) has residuals which are 3 or more standard deviations away from the mean.

However, assessing data integrity of RSQ suggests small deviation of normality at the very left tail indicating that some of our participants indicated high level of perceived stress related to COVID-19 in participant with ID 97.

### Residuals vs. Predicted

### Q-Q Plot Standardized Residuals

The quite balanced distribution of the residuals around the baseline suggests that the assumption of homoscedasticity has not been badly violated. The Q-Q plot shows that the standardized residuals fit along the diagonal suggesting that both assumptions or normality and linearity have also not been badly violated.

## RSQ and PSS as predictors of SE. Bayesian inference

| Model Comparison - SE | | | | | | | | | | | |
| --- | --- | --- | --- | --- | --- | --- | --- | --- | --- | --- | --- |
| Models | | P(M) | | P(M|data) | | BF M | | BF 10 | | R² | |
| Null model |  | 0.333 |  | 3.036e -17 |  | 6.072e -17 |  | 1.000 |  | 0.000 |  |
| PSS + RSQ |  | 0.333 |  | 0.867 |  | 13.034 |  | 2.856e +16 |  | 0.235 |  |
| PSS |  | 0.167 |  | 0.133 |  | 0.767 |  | 8.764e +15 |  | 0.219 |  |
| RSQ |  | 0.167 |  | 7.644e -13 |  | 3.822e -12 |  | 50356.710 |  | 0.080 |  |
|  | | | | | | | | | | | |

### Posterior Summary

| Posterior Summaries of Coefficients | | | | | | | | | | | | | | | |
| --- | --- | --- | --- | --- | --- | --- | --- | --- | --- | --- | --- | --- | --- | --- | --- |
|  | | | | | | | | | | | | 95% Credible Interval | | | |
| Coefficient | | Mean | | SD | | P(incl) | | P(incl|data) | | BF inclusion | | Lower | | Upper | |
| Intercept |  | 0.018 |  | 0.182 |  | 1.000 |  | 1.000 |  | 1.000 |  | -0.318 |  | 0.384 |  |
| PSS |  | -0.258 |  | 0.033 |  | 0.500 |  | 1.000 |  | 1.308e +12 |  | -0.318 |  | -0.189 |  |
| RSQ |  | -0.059 |  | 0.034 |  | 0.500 |  | 0.867 |  | 6.517 |  | -0.110 |  | 0.000 |  |
|  | | | | | | | | | | | | | | | |

### Residuals vs Fitted

### Inclusion Probabilities

### Q-Q Plot

### Marginal Posterior Distributions

#### Intercept

#### PSS

#### RSQ

## RSQ and PSS as predictors of AV

| Model Summary - AV | | | | | | | | | |
| --- | --- | --- | --- | --- | --- | --- | --- | --- | --- |
| Model | | R | | R² | | Adjusted R² | | RMSE | |
| H₀ |  | 0.000 |  | 0.000 |  | 0.000 |  | 4.084 |  |
| H₁ |  | 0.490 |  | 0.240 |  | 0.236 |  | 3.570 |  |
|  | | | | | | | | | |

| ANOVA | | | | | | | | | | | | | |
| --- | --- | --- | --- | --- | --- | --- | --- | --- | --- | --- | --- | --- | --- |
| Model | |  | | Sum of Squares | | df | | Mean Square | | F | | p | |
| H₁ |  | Regression |  | 1294.350 |  | 2 |  | 647.175 |  | 50.766 |  | < .001 |  |
|  |  | Residual |  | 4092.206 |  | 321 |  | 12.748 |  |  |  |  |  |
|  |  | Total |  | 5386.556 |  | 323 |  |  |  |  |  |  |  |
|  | | | | | | | | | | | | | |
|  |  |  |  |  |  |  |  |  |  |  |  |  |  |
| --- | --- | --- | --- | --- | --- | --- | --- | --- | --- | --- | --- | --- | --- |
| *Note.*  The intercept model is omitted, as no meaningful information can be shown. | | | | | | | | | | | | | |

| Coefficients | | | | | | | | | | | | | | | | | | | | | |
| --- | --- | --- | --- | --- | --- | --- | --- | --- | --- | --- | --- | --- | --- | --- | --- | --- | --- | --- | --- | --- | --- |
|  | | | | | | | | | | | | | | 95% CI | | | | Collinearity Statistics | | | |
| Model | |  | | Unstandardized | | Standard Error | | Standardized | | t | | p | | Lower | | Upper | | Tolerance | | VIF | |
| H₀ |  | (Intercept) |  | 0.004 |  | 0.227 |  |  |  | 0.016 |  | 0.987 |  | -0.443 |  | 0.450 |  |  |  |  |  |
| H₁ |  | (Intercept) |  | -0.010 |  | 0.198 |  |  |  | -0.052 |  | 0.959 |  | -0.400 |  | 0.380 |  |  |  |  |  |
|  |  | PSS |  | 0.170 |  | 0.035 |  | 0.250 |  | 4.844 |  | < .001 |  | 0.101 |  | 0.239 |  | 0.885 |  | 1.130 |  |
|  |  | RSQ |  | 0.193 |  | 0.029 |  | 0.345 |  | 6.670 |  | < .001 |  | 0.136 |  | 0.250 |  | 0.885 |  | 1.130 |  |
|  | | | | | | | | | | | | | | | | | | | | | |

| Collinearity Diagnostics | | | | | | | | | | | | | |
| --- | --- | --- | --- | --- | --- | --- | --- | --- | --- | --- | --- | --- | --- |
|  | | | | | | | | Variance Proportions | | | | | |
| Model | | Dimension | | Eigenvalue | | Condition Index | | (Intercept) | | PSS | | RSQ | |
| H₁ |  | 1 |  | 1.339 |  | 1.000 |  | 0.000 |  | 0.330 |  | 0.330 |  |
|  |  | 2 |  | 1.000 |  | 1.157 |  | 1.000 |  | 0.000 |  | 0.000 |  |
|  |  | 3 |  | 0.661 |  | 1.423 |  | 0.000 |  | 0.669 |  | 0.670 |  |
|  | | | | | | | | | | | | | |
|  |  |  |  |  |  |  |  |  |  |  |  |  |  |
| --- | --- | --- | --- | --- | --- | --- | --- | --- | --- | --- | --- | --- | --- |
| *Note.*  The intercept model is omitted, as no meaningful information can be shown. | | | | | | | | | | | | | |

## RSQ and PSS as predictors of AV. Bayesian inference

| Model Comparison - AV | | | | | | | | | | | |
| --- | --- | --- | --- | --- | --- | --- | --- | --- | --- | --- | --- |
| Models | | P(M) | | P(M|data) | | BF M | | BF 10 | | R² | |
| Null model |  | 0.333 |  | 1.388e -17 |  | 2.776e -17 |  | 1.000 |  | 0.000 |  |
| PSS + RSQ |  | 0.333 |  | 1.000 |  | 29846.100 |  | 7.205e +16 |  | 0.240 |  |
| RSQ |  | 0.167 |  | 6.700e  -5 |  | 3.350e  -4 |  | 9.656e +12 |  | 0.185 |  |
| PSS |  | 0.167 |  | 5.840e  -9 |  | 2.920e  -8 |  | 8.417e  +8 |  | 0.135 |  |
|  | | | | | | | | | | | |

### Posterior Summary

| Posterior Summaries of Coefficients | | | | | | | | | | | | | | | |
| --- | --- | --- | --- | --- | --- | --- | --- | --- | --- | --- | --- | --- | --- | --- | --- |
|  | | | | | | | | | | | | 95% Credible Interval | | | |
| Coefficient | | Mean | | SD | | P(incl) | | P(incl|data) | | BF inclusion | | Lower | | Upper | |
| Intercept |  | 0.004 |  | 0.198 |  | 1.000 |  | 1.000 |  | 1.000 |  | -0.356 |  | 0.405 |  |
| PSS |  | 0.166 |  | 0.035 |  | 0.500 |  | 1.000 |  | 14924.351 |  | 0.103 |  | 0.237 |  |
| RSQ |  | 0.189 |  | 0.029 |  | 0.500 |  | 1.000 |  | 1.712e +8 |  | 0.137 |  | 0.247 |  |
|  | | | | | | | | | | | | | | | |

## RSQ and PSS as predictors of AP

| Model Summary - AP | | | | | | | | | |
| --- | --- | --- | --- | --- | --- | --- | --- | --- | --- |
| Model | | R | | R² | | Adjusted R² | | RMSE | |
| H₀ |  | 0.000 |  | 0.000 |  | 0.000 |  | 5.845 |  |
| H₁ |  | 0.103 |  | 0.011 |  | 0.005 |  | 5.831 |  |
|  | | | | | | | | | |

| ANOVA | | | | | | | | | | | | | |
| --- | --- | --- | --- | --- | --- | --- | --- | --- | --- | --- | --- | --- | --- |
| Model | |  | | Sum of Squares | | df | | Mean Square | | F | | p | |
| H₁ |  | Regression |  | 118.189 |  | 2 |  | 59.094 |  | 1.738 |  | 0.178 |  |
|  |  | Residual |  | 10915.836 |  | 321 |  | 34.006 |  |  |  |  |  |
|  |  | Total |  | 11034.025 |  | 323 |  |  |  |  |  |  |  |
|  | | | | | | | | | | | | | |
|  |  |  |  |  |  |  |  |  |  |  |  |  |  |
| --- | --- | --- | --- | --- | --- | --- | --- | --- | --- | --- | --- | --- | --- |
| *Note.*  The intercept model is omitted, as no meaningful information can be shown. | | | | | | | | | | | | | |

| Coefficients | | | | | | | | | | | | | | | | | |
| --- | --- | --- | --- | --- | --- | --- | --- | --- | --- | --- | --- | --- | --- | --- | --- | --- | --- |
|  | | | | | | | | | | | | | | 95% CI | | | |
| Model | |  | | Unstandardized | | Standard Error | | Standardized | | t | | p | | Lower | | Upper | |
| H₀ |  | (Intercept) |  | 0.004 |  | 0.325 |  |  |  | 0.013 |  | 0.990 |  | -0.635 |  | 0.643 |  |
| H₁ |  | (Intercept) |  | 0.002 |  | 0.324 |  |  |  | 0.006 |  | 0.995 |  | -0.636 |  | 0.639 |  |
|  |  | PSS |  | -0.061 |  | 0.057 |  | -0.063 |  | -1.072 |  | 0.284 |  | -0.174 |  | 0.051 |  |
|  |  | RSQ |  | 0.085 |  | 0.047 |  | 0.106 |  | 1.798 |  | 0.073 |  | -0.008 |  | 0.178 |  |
|  | | | | | | | | | | | | | | | | | |

## RSQ and PSS as predictors of AP. Bayesian inference

| Model Comparison - AP | | | | | | | | | | | |
| --- | --- | --- | --- | --- | --- | --- | --- | --- | --- | --- | --- |
| Models | | P(M) | | P(M|data) | | BF M | | BF 10 | | R² | |
| Null model |  | 0.333 |  | 0.729 |  | 5.375 |  | 1.000 |  | 0.000 |  |
| RSQ |  | 0.167 |  | 0.135 |  | 0.784 |  | 0.372 |  | 0.007 |  |
| PSS + RSQ |  | 0.333 |  | 0.086 |  | 0.187 |  | 0.118 |  | 0.011 |  |
| PSS |  | 0.167 |  | 0.050 |  | 0.263 |  | 0.137 |  | 0.001 |  |
|  | | | | | | | | | | | |

### Posterior Summary

| Posterior Summaries of Coefficients | | | | | | | | | | | | | | | |
| --- | --- | --- | --- | --- | --- | --- | --- | --- | --- | --- | --- | --- | --- | --- | --- |
|  | | | | | | | | | | | | 95% Credible Interval | | | |
| Coefficient | | Mean | | SD | | P(incl) | | P(incl|data) | | BF inclusion | | Lower | | Upper | |
| Intercept |  | 0.004 |  | 0.325 |  | 1.000 |  | 1.000 |  | 1.000 |  | -0.626 |  | 0.578 |  |
| PSS |  | -0.006 |  | 0.026 |  | 0.500 |  | 0.136 |  | 0.157 |  | -0.108 |  | 0.003 |  |
| RSQ |  | 0.016 |  | 0.036 |  | 0.500 |  | 0.221 |  | 0.284 |  | -0.003 |  | 0.116 |  |
|  | | | | | | | | | | | | | | | |

#### Posterior Coefficients with 95% Credible Interval

### Residuals vs Fitted

### Inclusion Probabilities

### Q-Q Plot

### Marginal Posterior Distributions

#### Intercept

#### PSS

#### RSQ

## RSQ and PSS as predictors of LOT

| Model Summary - LOT | | | | | | | | | |
| --- | --- | --- | --- | --- | --- | --- | --- | --- | --- |
| Model | | R | | R² | | Adjusted R² | | RMSE | |
| H₀ |  | 0.000 |  | 0.000 |  | 0.000 |  | 4.931 |  |
| H₁ |  | 0.492 |  | 0.242 |  | 0.237 |  | 4.307 |  |
|  | | | | | | | | | |

| ANOVA | | | | | | | | | | | | | |
| --- | --- | --- | --- | --- | --- | --- | --- | --- | --- | --- | --- | --- | --- |
| Model | |  | | Sum of Squares | | df | | Mean Square | | F | | p | |
| H₁ |  | Regression |  | 1903.710 |  | 2 |  | 951.855 |  | 51.310 |  | < .001 |  |
|  |  | Residual |  | 5973.478 |  | 322 |  | 18.551 |  |  |  |  |  |
|  |  | Total |  | 7877.188 |  | 324 |  |  |  |  |  |  |  |
|  | | | | | | | | | | | | | |
|  |  |  |  |  |  |  |  |  |  |  |  |  |  |
| --- | --- | --- | --- | --- | --- | --- | --- | --- | --- | --- | --- | --- | --- |
| *Note.*  The intercept model is omitted, as no meaningful information can be shown. | | | | | | | | | | | | | |

| Coefficients | | | | | | | | | | | | | | | | | | | | | |
| --- | --- | --- | --- | --- | --- | --- | --- | --- | --- | --- | --- | --- | --- | --- | --- | --- | --- | --- | --- | --- | --- |
|  | | | | | | | | | | | | | | 95% CI | | | | Collinearity Statistics | | | |
| Model | |  | | Unstandardized | | Standard Error | | Standardized | | t | | p | | Lower | | Upper | | Tolerance | | VIF | |
| H₀ |  | (Intercept) |  | 0.006 |  | 0.274 |  |  |  | 0.022 |  | 0.982 |  | -0.532 |  | 0.544 |  |  |  |  |  |
| H₁ |  | (Intercept) |  | 0.008 |  | 0.239 |  |  |  | 0.033 |  | 0.974 |  | -0.462 |  | 0.478 |  |  |  |  |  |
|  |  | RSQ |  | -0.133 |  | 0.035 |  | -0.198 |  | -3.825 |  | < .001 |  | -0.202 |  | -0.065 |  | 0.882 |  | 1.134 |  |
|  |  | PSS |  | -0.317 |  | 0.042 |  | -0.387 |  | -7.492 |  | < .001 |  | -0.400 |  | -0.233 |  | 0.882 |  | 1.134 |  |
|  | | | | | | | | | | | | | | | | | | | | | |

| Collinearity Diagnostics | | | | | | | | | | | | | |
| --- | --- | --- | --- | --- | --- | --- | --- | --- | --- | --- | --- | --- | --- |
|  | | | | | | | | Variance Proportions | | | | | |
| Model | | Dimension | | Eigenvalue | | Condition Index | | (Intercept) | | RSQ | | PSS | |
| H₁ |  | 1 |  | 1.344 |  | 1.000 |  | 0.000 |  | 0.328 |  | 0.328 |  |
|  |  | 2 |  | 1.000 |  | 1.159 |  | 1.000 |  | 0.000 |  | 0.000 |  |
|  |  | 3 |  | 0.656 |  | 1.431 |  | 0.000 |  | 0.672 |  | 0.672 |  |
|  | | | | | | | | | | | | | |
|  |  |  |  |  |  |  |  |  |  |  |  |  |  |
| --- | --- | --- | --- | --- | --- | --- | --- | --- | --- | --- | --- | --- | --- |
| *Note.*  The intercept model is omitted, as no meaningful information can be shown. | | | | | | | | | | | | | |

## RSQ and PSS as predictors of LOT. Bayesian inference

| Model Comparison - LOT | | | | | | | | | | | |
| --- | --- | --- | --- | --- | --- | --- | --- | --- | --- | --- | --- |
| Models | | P(M) | | P(M|data) | | BF M | | BF 10 | | R² | |
| Null model |  | 0.333 |  | 9.073e -18 |  | 1.815e -17 |  | 1.000 |  | 0.000 |  |
| PSS + RSQ |  | 0.333 |  | 0.996 |  | 488.775 |  | 1.098e +17 |  | 0.242 |  |
| PSS |  | 0.167 |  | 0.004 |  | 0.020 |  | 8.983e +14 |  | 0.207 |  |
| RSQ |  | 0.167 |  | 4.175e -11 |  | 2.088e -10 |  | 9.203e  +6 |  | 0.109 |  |
|  | | | | | | | | | | | |

### Posterior Summary

| Posterior Summaries of Coefficients | | | | | | | | | | | | | | | |
| --- | --- | --- | --- | --- | --- | --- | --- | --- | --- | --- | --- | --- | --- | --- | --- |
|  | | | | | | | | | | | | 95% Credible Interval | | | |
| Coefficient | | Mean | | SD | | P(incl) | | P(incl|data) | | BF inclusion | | Lower | | Upper | |
| Intercept |  | 0.006 |  | 0.239 |  | 1.000 |  | 1.000 |  | 1.000 |  | -0.473 |  | 0.441 |  |
| PSS |  | -0.310 |  | 0.042 |  | 0.500 |  | 1.000 |  | 2.395e +10 |  | -0.395 |  | -0.233 |  |
| RSQ |  | -0.130 |  | 0.035 |  | 0.500 |  | 0.996 |  | 244.387 |  | -0.198 |  | -0.062 |  |
|  | | | | | | | | | | | | | | | |

#### Posterior Coefficients with 95% Credible Interval

### Residuals vs Fitted

Plotting not possible: subscript out of bounds

### Inclusion Probabilities

### Q-Q Plot

### Marginal Posterior Distributions

#### Intercept

#### PSS

#### RSQ

| Descriptives | | | | | | | |
| --- | --- | --- | --- | --- | --- | --- | --- |
|  | | N | | Mean | | SD | |
| LOT |  | 325 |  | 0.006 |  | 4.931 |  |
| PSS |  | 325 |  | 0.002 |  | 6.030 |  |
| RSQ |  | 325 |  | 0.007 |  | 7.307 |  |
|  | | | | | | | |

## RSQ and PSS as predictors of SC

| Model Summary - SC | | | | | | | | | |
| --- | --- | --- | --- | --- | --- | --- | --- | --- | --- |
| Model | | R | | R² | | Adjusted R² | | RMSE | |
| H₀ |  | 0.000 |  | 0.000 |  | 0.000 |  | 8.891 |  |
| H₁ |  | 0.539 |  | 0.291 |  | 0.287 |  | 7.510 |  |
|  | | | | | | | | | |

| ANOVA | | | | | | | | | | | | | |
| --- | --- | --- | --- | --- | --- | --- | --- | --- | --- | --- | --- | --- | --- |
| Model | |  | | Sum of Squares | | df | | Mean Square | | F | | p | |
| H₁ |  | Regression |  | 7453.696 |  | 2 |  | 3726.848 |  | 66.082 |  | < .001 |  |
|  |  | Residual |  | 18159.892 |  | 322 |  | 56.397 |  |  |  |  |  |
|  |  | Total |  | 25613.588 |  | 324 |  |  |  |  |  |  |  |
|  | | | | | | | | | | | | | |
|  |  |  |  |  |  |  |  |  |  |  |  |  |  |
| --- | --- | --- | --- | --- | --- | --- | --- | --- | --- | --- | --- | --- | --- |
| *Note.*  The intercept model is omitted, as no meaningful information can be shown. | | | | | | | | | | | | | |

| Coefficients | | | | | | | | | | | | | | | | | | | | | |
| --- | --- | --- | --- | --- | --- | --- | --- | --- | --- | --- | --- | --- | --- | --- | --- | --- | --- | --- | --- | --- | --- |
|  | | | | | | | | | | | | | | 95% CI | | | | Collinearity Statistics | | | |
| Model | |  | | Unstandardized | | Standard Error | | Standardized | | t | | p | | Lower | | Upper | | Tolerance | | VIF | |
| H₀ |  | (Intercept) |  | 0.006 |  | 0.493 |  |  |  | 0.012 |  | 0.990 |  | -0.964 |  | 0.976 |  |  |  |  |  |
| H₁ |  | (Intercept) |  | 0.009 |  | 0.417 |  |  |  | 0.021 |  | 0.983 |  | -0.811 |  | 0.828 |  |  |  |  |  |
|  |  | PSS |  | -0.716 |  | 0.074 |  | -0.486 |  | -9.723 |  | < .001 |  | -0.861 |  | -0.571 |  | 0.882 |  | 1.134 |  |
|  |  | RSQ |  | -0.147 |  | 0.061 |  | -0.121 |  | -2.414 |  | 0.016 |  | -0.266 |  | -0.027 |  | 0.882 |  | 1.134 |  |
|  | | | | | | | | | | | | | | | | | | | | | |

| Collinearity Diagnostics | | | | | | | | | | | | | |
| --- | --- | --- | --- | --- | --- | --- | --- | --- | --- | --- | --- | --- | --- |
|  | | | | | | | | Variance Proportions | | | | | |
| Model | | Dimension | | Eigenvalue | | Condition Index | | (Intercept) | | PSS | | RSQ | |
| H₁ |  | 1 |  | 1.344 |  | 1.000 |  | 0.000 |  | 0.328 |  | 0.328 |  |
|  |  | 2 |  | 1.000 |  | 1.159 |  | 1.000 |  | 0.000 |  | 0.000 |  |
|  |  | 3 |  | 0.656 |  | 1.431 |  | 0.000 |  | 0.672 |  | 0.672 |  |
|  | | | | | | | | | | | | | |
|  |  |  |  |  |  |  |  |  |  |  |  |  |  |
| --- | --- | --- | --- | --- | --- | --- | --- | --- | --- | --- | --- | --- | --- |
| *Note.*  The intercept model is omitted, as no meaningful information can be shown. | | | | | | | | | | | | | |

| Residuals Statistics | | | | | | | | | | | |
| --- | --- | --- | --- | --- | --- | --- | --- | --- | --- | --- | --- |
|  | | Minimum | | Maximum | | Mean | | SD | | N | |
| Predicted Value |  | -16.235 |  | 10.344 |  | 0.006 |  | 4.796 |  | 325 |  |
| Residual |  | -24.610 |  | 20.012 |  | 6.685e -17 |  | 7.487 |  | 325 |  |
| Std. Predicted Value |  | -3.386 |  | 2.155 |  | 1.186e -18 |  | 1.000 |  | 325 |  |
| Std. Residual |  | -3.289 |  | 2.684 |  | 3.002e  -4 |  | 1.002 |  | 325 |  |
|  | | | | | | | | | | | |

### Residuals vs. Predicted

### Q-Q Plot Standardized Residuals

## RSQ and PSS as predictors of SC. Bayesian inference

| Model Comparison - SC | | | | | | | | | | | |
| --- | --- | --- | --- | --- | --- | --- | --- | --- | --- | --- | --- |
| Models | | P(M) | | P(M|data) | | BF M | | BF 10 | | R² | |
| Null model |  | 0.333 |  | 1.719e -22 |  | 3.438e -22 |  | 1.000 |  | 0.000 |  |
| PSS + RSQ |  | 0.333 |  | 0.769 |  | 6.673 |  | 4.476e +21 |  | 0.291 |  |
| PSS |  | 0.167 |  | 0.231 |  | 1.499 |  | 2.683e +21 |  | 0.278 |  |
| RSQ |  | 0.167 |  | 7.649e -18 |  | 3.824e -17 |  | 88983.577 |  | 0.083 |  |
|  | | | | | | | | | | | |

### Posterior Summary

| Posterior Summaries of Coefficients | | | | | | | | | | | | | | | |
| --- | --- | --- | --- | --- | --- | --- | --- | --- | --- | --- | --- | --- | --- | --- | --- |
|  | | | | | | | | | | | | 95% Credible Interval | | | |
| Coefficient | | Mean | | SD | | P(incl) | | P(incl|data) | | BF inclusion | | Lower | | Upper | |
| Intercept |  | 0.006 |  | 0.417 |  | 1.000 |  | 1.000 |  | 1.000 |  | -0.842 |  | 0.792 |  |
| PSS |  | -0.719 |  | 0.077 |  | 0.500 |  | 1.000 |  | 1.307e +17 |  | -0.863 |  | -0.572 |  |
| RSQ |  | -0.111 |  | 0.081 |  | 0.500 |  | 0.769 |  | 3.337 |  | -0.249 |  | 0.000 |  |
|  | | | | | | | | | | | | | | | |

#### Posterior Coefficients with 95% Credible Interval

### Inclusion Probabilities

### Q-Q Plot

### Marginal Posterior Distributions

#### Intercept

#### PSS

#### RSQ

## RSQ and PSS as predictors of CS

| Model Summary - CS | | | | | | | | | |
| --- | --- | --- | --- | --- | --- | --- | --- | --- | --- |
| Model | | R | | R² | | Adjusted R² | | RMSE | |
| H₀ |  | 0.000 |  | 0.000 |  | 0.000 |  | 5.033 |  |
| H₁ |  | 0.358 |  | 0.128 |  | 0.123 |  | 4.714 |  |
|  | | | | | | | | | |

| ANOVA | | | | | | | | | | | | | |
| --- | --- | --- | --- | --- | --- | --- | --- | --- | --- | --- | --- | --- | --- |
| Model | |  | | Sum of Squares | | df | | Mean Square | | F | | p | |
| H₁ |  | Regression |  | 1051.307 |  | 2 |  | 525.654 |  | 23.653 |  | < .001 |  |
|  |  | Residual |  | 7156.096 |  | 322 |  | 22.224 |  |  |  |  |  |
|  |  | Total |  | 8207.403 |  | 324 |  |  |  |  |  |  |  |
|  | | | | | | | | | | | | | |
|  |  |  |  |  |  |  |  |  |  |  |  |  |  |
| --- | --- | --- | --- | --- | --- | --- | --- | --- | --- | --- | --- | --- | --- |
| *Note.*  The intercept model is omitted, as no meaningful information can be shown. | | | | | | | | | | | | | |

| Coefficients | | | | | | | | | | | | | | | | | | | | | |
| --- | --- | --- | --- | --- | --- | --- | --- | --- | --- | --- | --- | --- | --- | --- | --- | --- | --- | --- | --- | --- | --- |
|  | | | | | | | | | | | | | | 95% CI | | | | Collinearity Statistics | | | |
| Model | |  | | Unstandardized | | Standard Error | | Standardized | | t | | p | | Lower | | Upper | | Tolerance | | VIF | |
| H₀ |  | (Intercept) |  | 0.005 |  | 0.279 |  |  |  | 0.019 |  | 0.985 |  | -0.544 |  | 0.555 |  |  |  |  |  |
| H₁ |  | (Intercept) |  | 0.006 |  | 0.261 |  |  |  | 0.024 |  | 0.981 |  | -0.508 |  | 0.521 |  |  |  |  |  |
|  |  | PSS |  | -0.281 |  | 0.046 |  | -0.337 |  | -6.076 |  | < .001 |  | -0.372 |  | -0.190 |  | 0.882 |  | 1.134 |  |
|  |  | RSQ |  | -0.036 |  | 0.038 |  | -0.052 |  | -0.935 |  | 0.350 |  | -0.111 |  | 0.039 |  | 0.882 |  | 1.134 |  |
|  | | | | | | | | | | | | | | | | | | | | | |

| Collinearity Diagnostics | | | | | | | | | | | | | |
| --- | --- | --- | --- | --- | --- | --- | --- | --- | --- | --- | --- | --- | --- |
|  | | | | | | | | Variance Proportions | | | | | |
| Model | | Dimension | | Eigenvalue | | Condition Index | | (Intercept) | | PSS | | RSQ | |
| H₁ |  | 1 |  | 1.344 |  | 1.000 |  | 0.000 |  | 0.328 |  | 0.328 |  |
|  |  | 2 |  | 1.000 |  | 1.159 |  | 1.000 |  | 0.000 |  | 0.000 |  |
|  |  | 3 |  | 0.656 |  | 1.431 |  | 0.000 |  | 0.672 |  | 0.672 |  |
|  | | | | | | | | | | | | | |
|  |  |  |  |  |  |  |  |  |  |  |  |  |  |
| --- | --- | --- | --- | --- | --- | --- | --- | --- | --- | --- | --- | --- | --- |
| *Note.*  The intercept model is omitted, as no meaningful information can be shown. | | | | | | | | | | | | | |

| Residuals Statistics | | | | | | | | | | | |
| --- | --- | --- | --- | --- | --- | --- | --- | --- | --- | --- | --- |
|  | | Minimum | | Maximum | | Mean | | SD | | N | |
| Predicted Value |  | -6.259 |  | 3.730 |  | 0.005 |  | 1.801 |  | 325 |  |
| Residual |  | -19.824 |  | 9.722 |  | -1.175e -16 |  | 4.700 |  | 325 |  |
| Std. Predicted Value |  | -3.478 |  | 2.068 |  | -1.519e -17 |  | 1.000 |  | 325 |  |
| Std. Residual |  | -4.212 |  | 2.087 |  | 3.106e  -4 |  | 1.002 |  | 325 |  |
|  | | | | | | | | | | | |

### Residuals vs. Predicted

### Q-Q Plot Standardized Residuals

## RSQ and PSS as predictors of CS. Bayesian inference

| Model Comparison - CS | | | | | | | | | | | |
| --- | --- | --- | --- | --- | --- | --- | --- | --- | --- | --- | --- |
| Models | | P(M) | | P(M|data) | | BF M | | BF 10 | | R² | |
| Null model |  | 0.333 |  | 8.456e -9 |  | 1.691e -8 |  | 1.000 |  | 0.000 |  |
| PSS |  | 0.167 |  | 0.710 |  | 12.225 |  | 1.679e +8 |  | 0.126 |  |
| PSS + RSQ |  | 0.333 |  | 0.290 |  | 0.818 |  | 3.433e +7 |  | 0.128 |  |
| RSQ |  | 0.167 |  | 4.335e -8 |  | 2.168e -7 |  | 10.254 |  | 0.028 |  |
|  | | | | | | | | | | | |

### Posterior Summary

| Posterior Summaries of Coefficients | | | | | | | | | | | | | | | |
| --- | --- | --- | --- | --- | --- | --- | --- | --- | --- | --- | --- | --- | --- | --- | --- |
|  | | | | | | | | | | | | 95% Credible Interval | | | |
| Coefficient | | Mean | | SD | | P(incl) | | P(incl|data) | | BF inclusion | | Lower | | Upper | |
| Intercept |  | 0.005 |  | 0.261 |  | 1.000 |  | 1.000 |  | 1.000 |  | -0.499 |  | 0.488 |  |
| PSS |  | -0.284 |  | 0.044 |  | 0.500 |  | 1.000 |  | 1.930e +7 |  | -0.363 |  | -0.195 |  |
| RSQ |  | -0.010 |  | 0.026 |  | 0.500 |  | 0.290 |  | 0.409 |  | -0.080 |  | 0.017 |  |
|  | | | | | | | | | | | | | | | |

#### Posterior Coefficients with 95% Credible Interval

### Inclusion Probabilities

### Q-Q Plot

### Marginal Posterior Distributions

#### Intercept

#### PSS

#### RSQ

## RSQ and PSS as predictors of BU

| Model Summary - BU | | | | | | | | | |
| --- | --- | --- | --- | --- | --- | --- | --- | --- | --- |
| Model | | R | | R² | | Adjusted R² | | RMSE | |
| H₀ |  | 0.000 |  | 0.000 |  | 0.000 |  | 4.915 |  |
| H₁ |  | 0.511 |  | 0.261 |  | 0.257 |  | 4.237 |  |
|  | | | | | | | | | |

| ANOVA | | | | | | | | | | | | | |
| --- | --- | --- | --- | --- | --- | --- | --- | --- | --- | --- | --- | --- | --- |
| Model | |  | | Sum of Squares | | df | | Mean Square | | F | | p | |
| H₁ |  | Regression |  | 2044.953 |  | 2 |  | 1022.476 |  | 56.947 |  | < .001 |  |
|  |  | Residual |  | 5781.435 |  | 322 |  | 17.955 |  |  |  |  |  |
|  |  | Total |  | 7826.388 |  | 324 |  |  |  |  |  |  |  |
|  | | | | | | | | | | | | | |
|  |  |  |  |  |  |  |  |  |  |  |  |  |  |
| --- | --- | --- | --- | --- | --- | --- | --- | --- | --- | --- | --- | --- | --- |
| *Note.*  The intercept model is omitted, as no meaningful information can be shown. | | | | | | | | | | | | | |

| Coefficients | | | | | | | | | | | | | | | | | | | | | |
| --- | --- | --- | --- | --- | --- | --- | --- | --- | --- | --- | --- | --- | --- | --- | --- | --- | --- | --- | --- | --- | --- |
|  | | | | | | | | | | | | | | 95% CI | | | | Collinearity Statistics | | | |
| Model | |  | | Unstandardized | | Standard Error | | Standardized | | t | | p | | Lower | | Upper | | Tolerance | | VIF | |
| H₀ |  | (Intercept) |  | 0.004 |  | 0.273 |  |  |  | 0.014 |  | 0.989 |  | -0.532 |  | 0.540 |  |  |  |  |  |
| H₁ |  | (Intercept) |  | 0.002 |  | 0.235 |  |  |  | 0.008 |  | 0.993 |  | -0.460 |  | 0.464 |  |  |  |  |  |
|  |  | PSS |  | 0.302 |  | 0.042 |  | 0.370 |  | 7.252 |  | < .001 |  | 0.220 |  | 0.383 |  | 0.882 |  | 1.134 |  |
|  |  | RSQ |  | 0.167 |  | 0.034 |  | 0.248 |  | 4.856 |  | < .001 |  | 0.099 |  | 0.234 |  | 0.882 |  | 1.134 |  |
|  | | | | | | | | | | | | | | | | | | | | | |

| Collinearity Diagnostics | | | | | | | | | | | | | |
| --- | --- | --- | --- | --- | --- | --- | --- | --- | --- | --- | --- | --- | --- |
|  | | | | | | | | Variance Proportions | | | | | |
| Model | | Dimension | | Eigenvalue | | Condition Index | | (Intercept) | | PSS | | RSQ | |
| H₁ |  | 1 |  | 1.344 |  | 1.000 |  | 0.000 |  | 0.328 |  | 0.328 |  |
|  |  | 2 |  | 1.000 |  | 1.159 |  | 1.000 |  | 0.000 |  | 0.000 |  |
|  |  | 3 |  | 0.656 |  | 1.431 |  | 0.000 |  | 0.672 |  | 0.672 |  |
|  | | | | | | | | | | | | | |
|  |  |  |  |  |  |  |  |  |  |  |  |  |  |
| --- | --- | --- | --- | --- | --- | --- | --- | --- | --- | --- | --- | --- | --- |
| *Note.*  The intercept model is omitted, as no meaningful information can be shown. | | | | | | | | | | | | | |

| Residuals Statistics | | | | | | | | | | | |
| --- | --- | --- | --- | --- | --- | --- | --- | --- | --- | --- | --- |
|  | | Minimum | | Maximum | | Mean | | SD | | N | |
| Predicted Value |  | -5.932 |  | 7.351 |  | 0.004 |  | 2.512 |  | 325 |  |
| Residual |  | -11.561 |  | 14.859 |  | -7.375e -17 |  | 4.224 |  | 325 |  |
| Std. Predicted Value |  | -2.363 |  | 2.924 |  | 1.207e -17 |  | 1.000 |  | 325 |  |
| Std. Residual |  | -2.761 |  | 3.513 |  | -3.518e  -4 |  | 1.002 |  | 325 |  |
|  | | | | | | | | | | | |

### Residuals vs. Predicted

### Q-Q Plot Standardized Residuals

## RSQ and PSS as predictors of BU. Bayesian inference

| Model Comparison - BU | | | | | | | | | | | |
| --- | --- | --- | --- | --- | --- | --- | --- | --- | --- | --- | --- |
| Models | | P(M) | | P(M|data) | | BF M | | BF 10 | | R² | |
| Null model |  | 0.333 |  | 1.462e -19 |  | 2.924e -19 |  | 1.000 |  | 0.000 |  |
| PSS + RSQ |  | 0.333 |  | 1.000 |  | 30730.660 |  | 6.839e +18 |  | 0.261 |  |
| PSS |  | 0.167 |  | 6.508e  -5 |  | 3.254e  -4 |  | 8.902e +14 |  | 0.207 |  |
| RSQ |  | 0.167 |  | 1.852e -10 |  | 9.262e -10 |  | 2.534e  +9 |  | 0.141 |  |
|  | | | | | | | | | | | |

### Posterior Summary

| Posterior Summaries of Coefficients | | | | | | | | | | | | | | | |
| --- | --- | --- | --- | --- | --- | --- | --- | --- | --- | --- | --- | --- | --- | --- | --- |
|  | | | | | | | | | | | | 95% Credible Interval | | | |
| Coefficient | | Mean | | SD | | P(incl) | | P(incl|data) | | BF inclusion | | Lower | | Upper | |
| Intercept |  | 0.004 |  | 0.235 |  | 1.000 |  | 1.000 |  | 1.000 |  | -0.496 |  | 0.428 |  |
| PSS |  | 0.296 |  | 0.041 |  | 0.500 |  | 1.000 |  | 5.398e +9 |  | 0.208 |  | 0.370 |  |
| RSQ |  | 0.163 |  | 0.034 |  | 0.500 |  | 1.000 |  | 15365.374 |  | 0.090 |  | 0.224 |  |
|  | | | | | | | | | | | | | | | |

#### Posterior Coefficients with 95% Credible Interval

### Residuals vs Fitted

Plotting not possible: subscript out of bounds

### Inclusion Probabilities

### Q-Q Plot

### Marginal Posterior Distributions

#### Intercept

#### PSS

#### RSQ

## RSQ and PSS as preditors of STS

| Model Summary - STS | | | | | | | | | |
| --- | --- | --- | --- | --- | --- | --- | --- | --- | --- |
| Model | | R | | R² | | Adjusted R² | | RMSE | |
| H₀ |  | 0.000 |  | 0.000 |  | 0.000 |  | 4.501 |  |
| H₁ |  | 0.455 |  | 0.207 |  | 0.202 |  | 4.021 |  |
|  | | | | | | | | | |

| ANOVA | | | | | | | | | | | | | |
| --- | --- | --- | --- | --- | --- | --- | --- | --- | --- | --- | --- | --- | --- |
| Model | |  | | Sum of Squares | | df | | Mean Square | | F | | p | |
| H₁ |  | Regression |  | 1358.678 |  | 2 |  | 679.339 |  | 42.016 |  | < .001 |  |
|  |  | Residual |  | 5206.232 |  | 322 |  | 16.168 |  |  |  |  |  |
|  |  | Total |  | 6564.911 |  | 324 |  |  |  |  |  |  |  |
|  | | | | | | | | | | | | | |
|  |  |  |  |  |  |  |  |  |  |  |  |  |  |
| --- | --- | --- | --- | --- | --- | --- | --- | --- | --- | --- | --- | --- | --- |
| *Note.*  The intercept model is omitted, as no meaningful information can be shown. | | | | | | | | | | | | | |

| Coefficients | | | | | | | | | | | | | | | | | | | | | |
| --- | --- | --- | --- | --- | --- | --- | --- | --- | --- | --- | --- | --- | --- | --- | --- | --- | --- | --- | --- | --- | --- |
|  | | | | | | | | | | | | | | 95% CI | | | | Collinearity Statistics | | | |
| Model | |  | | Unstandardized | | Standard Error | | Standardized | | t | | p | | Lower | | Upper | | Tolerance | | VIF | |
| H₀ |  | (Intercept) |  | 0.008 |  | 0.250 |  |  |  | 0.031 |  | 0.975 |  | -0.484 |  | 0.499 |  |  |  |  |  |
| H₁ |  | (Intercept) |  | 0.006 |  | 0.223 |  |  |  | 0.028 |  | 0.978 |  | -0.433 |  | 0.445 |  |  |  |  |  |
|  |  | PSS |  | 0.247 |  | 0.039 |  | 0.330 |  | 6.251 |  | < .001 |  | 0.169 |  | 0.324 |  | 0.882 |  | 1.134 |  |
|  |  | RSQ |  | 0.135 |  | 0.033 |  | 0.219 |  | 4.146 |  | < .001 |  | 0.071 |  | 0.199 |  | 0.882 |  | 1.134 |  |
|  | | | | | | | | | | | | | | | | | | | | | |

| Collinearity Diagnostics | | | | | | | | | | | | | |
| --- | --- | --- | --- | --- | --- | --- | --- | --- | --- | --- | --- | --- | --- |
|  | | | | | | | | Variance Proportions | | | | | |
| Model | | Dimension | | Eigenvalue | | Condition Index | | (Intercept) | | PSS | | RSQ | |
| H₁ |  | 1 |  | 1.344 |  | 1.000 |  | 0.000 |  | 0.328 |  | 0.328 |  |
|  |  | 2 |  | 1.000 |  | 1.159 |  | 1.000 |  | 0.000 |  | 0.000 |  |
|  |  | 3 |  | 0.656 |  | 1.431 |  | 0.000 |  | 0.672 |  | 0.672 |  |
|  | | | | | | | | | | | | | |
|  |  |  |  |  |  |  |  |  |  |  |  |  |  |
| --- | --- | --- | --- | --- | --- | --- | --- | --- | --- | --- | --- | --- | --- |
| *Note.*  The intercept model is omitted, as no meaningful information can be shown. | | | | | | | | | | | | | |

| Residuals Statistics | | | | | | | | | | | |
| --- | --- | --- | --- | --- | --- | --- | --- | --- | --- | --- | --- |
|  | | Minimum | | Maximum | | Mean | | SD | | N | |
| Predicted Value |  | -4.827 |  | 6.010 |  | 0.008 |  | 2.048 |  | 325 |  |
| Residual |  | -12.199 |  | 16.304 |  | -9.597e -17 |  | 4.009 |  | 325 |  |
| Std. Predicted Value |  | -2.361 |  | 2.931 |  | -6.612e -18 |  | 1.000 |  | 325 |  |
| Std. Residual |  | -3.048 |  | 4.069 |  | -4.092e  -4 |  | 1.002 |  | 325 |  |
|  | | | | | | | | | | | |

### Residuals vs. Predicted

### Q-Q Plot Standardized Residuals

## RSQ and PSS as predictors of STS. Bayesian inference

| Model Comparison - STS | | | | | | | | | | | |
| --- | --- | --- | --- | --- | --- | --- | --- | --- | --- | --- | --- |
| Models | | P(M) | | P(M|data) | | BF M | | BF 10 | | R² | |
| Null model |  | 0.333 |  | 1.042e -14 |  | 2.084e -14 |  | 1.000 |  | 0.000 |  |
| PSS + RSQ |  | 0.333 |  | 0.999 |  | 1711.949 |  | 9.586e +13 |  | 0.207 |  |
| PSS |  | 0.167 |  | 0.001 |  | 0.006 |  | 2.240e +11 |  | 0.165 |  |
| RSQ |  | 0.167 |  | 5.986e  -8 |  | 2.993e  -7 |  | 1.149e  +7 |  | 0.111 |  |
|  | | | | | | | | | | | |

### Posterior Summary

| Posterior Summaries of Coefficients | | | | | | | | | | | | | | | |
| --- | --- | --- | --- | --- | --- | --- | --- | --- | --- | --- | --- | --- | --- | --- | --- |
|  | | | | | | | | | | | | 95% Credible Interval | | | |
| Coefficient | | Mean | | SD | | P(incl) | | P(incl|data) | | BF inclusion | | Lower | | Upper | |
| Intercept |  | 0.008 |  | 0.223 |  | 1.000 |  | 1.000 |  | 1.000 |  | -0.428 |  | 0.491 |  |
| PSS |  | 0.241 |  | 0.039 |  | 0.500 |  | 1.000 |  | 1.670e +7 |  | 0.165 |  | 0.325 |  |
| RSQ |  | 0.132 |  | 0.032 |  | 0.500 |  | 0.999 |  | 856.018 |  | 0.069 |  | 0.201 |  |
|  | | | | | | | | | | | | | | | |

#### Posterior Coefficients with 95% Credible Interval

### Inclusion Probabilities

### Q-Q Plot

### Marginal Posterior Distributions

#### Intercept

#### PSS

#### RSQ

## SE, LOT, SC, STS, BU, CS, AV, AP as predictors of RESIL

We tested the relationship between potential mediators and resilience using a multiple regression analysys.

| Model Summary - RESIL | | | | | | | | | | | | | | | |
| --- | --- | --- | --- | --- | --- | --- | --- | --- | --- | --- | --- | --- | --- | --- | --- |
|  | | | | | | | | | | Durbin-Watson | | | | | |
| Model | | R | | R² | | Adjusted R² | | RMSE | | Autocorrelation | | Statistic | | p | |
| H₀ |  | 0.000 |  | 0.000 |  | 0.000 |  | 11.171 |  | 0.045 |  | 1.908 |  | 0.407 |  |
| H₁ |  | 0.810 |  | 0.656 |  | 0.647 |  | 6.638 |  | -0.077 |  | 2.151 |  | 0.174 |  |
|  | | | | | | | | | | | | | | | |

The Model summary shows that the potential mediators accounts for 63.6% of resilience variance

| ANOVA | | | | | | | | | | | | | |
| --- | --- | --- | --- | --- | --- | --- | --- | --- | --- | --- | --- | --- | --- |
| Model | |  | | Sum of Squares | | df | | Mean Square | | F | | p | |
| H₁ |  | Regression |  | 26429.220 |  | 8 |  | 3303.653 |  | 74.976 |  | < .001 |  |
|  |  | Residual |  | 13879.752 |  | 315 |  | 44.063 |  |  |  |  |  |
|  |  | Total |  | 40308.972 |  | 323 |  |  |  |  |  |  |  |
|  | | | | | | | | | | | | | |
|  |  |  |  |  |  |  |  |  |  |  |  |  |  |
| --- | --- | --- | --- | --- | --- | --- | --- | --- | --- | --- | --- | --- | --- |
| *Note.*  The intercept model is omitted, as no meaningful information can be shown. | | | | | | | | | | | | | |

| Coefficients | | | | | | | | | | | | | | | | | | | | | |
| --- | --- | --- | --- | --- | --- | --- | --- | --- | --- | --- | --- | --- | --- | --- | --- | --- | --- | --- | --- | --- | --- |
|  | | | | | | | | | | | | | | 95% CI | | | | Collinearity Statistics | | | |
| Model | |  | | Unstandardized | | Standard Error | | Standardized | | t | | p | | Lower | | Upper | | Tolerance | | VIF | |
| H₀ |  | (Intercept) |  | -0.061 |  | 0.621 |  |  |  | -0.098 |  | 0.922 |  | -1.282 |  | 1.160 |  |  |  |  |  |
| H₁ |  | (Intercept) |  | -0.040 |  | 0.369 |  |  |  | -0.109 |  | 0.913 |  | -0.766 |  | 0.685 |  |  |  |  |  |
|  |  | SE |  | 1.040 |  | 0.125 |  | 0.345 |  | 8.298 |  | < .001 |  | 0.793 |  | 1.286 |  | 0.631 |  | 1.585 |  |
|  |  | LOT |  | 0.284 |  | 0.097 |  | 0.126 |  | 2.932 |  | 0.004 |  | 0.094 |  | 0.475 |  | 0.595 |  | 1.680 |  |
|  |  | SC |  | 0.211 |  | 0.056 |  | 0.168 |  | 3.740 |  | < .001 |  | 0.100 |  | 0.322 |  | 0.542 |  | 1.845 |  |
|  |  | STS |  | 0.234 |  | 0.099 |  | 0.094 |  | 2.352 |  | 0.019 |  | 0.038 |  | 0.429 |  | 0.681 |  | 1.468 |  |
|  |  | BU |  | -0.444 |  | 0.127 |  | -0.196 |  | -3.504 |  | < .001 |  | -0.694 |  | -0.195 |  | 0.351 |  | 2.849 |  |
|  |  | CS |  | 0.345 |  | 0.105 |  | 0.155 |  | 3.284 |  | 0.001 |  | 0.138 |  | 0.551 |  | 0.490 |  | 2.039 |  |
|  |  | AV |  | -0.205 |  | 0.113 |  | -0.075 |  | -1.815 |  | 0.070 |  | -0.427 |  | 0.017 |  | 0.643 |  | 1.556 |  |
|  |  | AP |  | 0.279 |  | 0.072 |  | 0.146 |  | 3.851 |  | < .001 |  | 0.136 |  | 0.421 |  | 0.763 |  | 1.311 |  |
|  | | | | | | | | | | | | | | | | | | | | | |

The table indicates that all potential mediators are good predictors of resilience scores excluding AV

| Descriptives | | | | | | | | | |
| --- | --- | --- | --- | --- | --- | --- | --- | --- | --- |
|  | | N | | Mean | | SD | | SE | |
| RESIL |  | 324 |  | -0.061 |  | 11.171 |  | 0.621 |  |
| SE |  | 324 |  | -0.004 |  | 3.711 |  | 0.206 |  |
| LOT |  | 324 |  | -0.002 |  | 4.936 |  | 0.274 |  |
| SC |  | 324 |  | -0.021 |  | 8.891 |  | 0.494 |  |
| STS |  | 324 |  | -1.235e -4 |  | 4.506 |  | 0.250 |  |
| BU |  | 324 |  | 0.015 |  | 4.918 |  | 0.273 |  |
| CS |  | 324 |  | -0.014 |  | 5.028 |  | 0.279 |  |
| AV |  | 324 |  | 0.004 |  | 4.084 |  | 0.227 |  |
| AP |  | 324 |  | 0.004 |  | 5.845 |  | 0.325 |  |
|  | | | | | | | | | |

| Collinearity Diagnostics | | | | | | | | | | | | | | | | | | | | | | | | | |
| --- | --- | --- | --- | --- | --- | --- | --- | --- | --- | --- | --- | --- | --- | --- | --- | --- | --- | --- | --- | --- | --- | --- | --- | --- | --- |
|  | | | | | | | | Variance Proportions | | | | | | | | | | | | | | | | | |
| Model | | Dimension | | Eigenvalue | | Condition Index | | (Intercept) | | SE | | LOT | | SC | | STS | | BU | | CS | | AV | | AP | |
| H₁ |  | 1 |  | 3.418 |  | 1.000 |  | 0.000 |  | 0.028 |  | 0.026 |  | 0.028 |  | 0.017 |  | 0.021 |  | 0.018 |  | 0.014 |  | 0.006 |  |
|  |  | 2 |  | 1.369 |  | 1.580 |  | 0.000 |  | 0.000 |  | 0.003 |  | 0.000 |  | 0.061 |  | 0.001 |  | 0.034 |  | 0.161 |  | 0.238 |  |
|  |  | 3 |  | 1.000 |  | 1.849 |  | 1.000 |  | 0.000 |  | 0.000 |  | 0.000 |  | 0.000 |  | 0.000 |  | 0.000 |  | 0.000 |  | 0.000 |  |
|  |  | 4 |  | 0.808 |  | 2.057 |  | 0.000 |  | 0.024 |  | 0.174 |  | 0.080 |  | 0.185 |  | 0.064 |  | 0.121 |  | 0.027 |  | 0.015 |  |
|  |  | 5 |  | 0.714 |  | 2.188 |  | 0.000 |  | 0.003 |  | 0.001 |  | 0.000 |  | 0.426 |  | 0.004 |  | 0.195 |  | 0.017 |  | 0.254 |  |
|  |  | 6 |  | 0.570 |  | 2.449 |  | 0.000 |  | 0.855 |  | 0.015 |  | 0.115 |  | 0.003 |  | 0.033 |  | 0.000 |  | 0.037 |  | 0.006 |  |
|  |  | 7 |  | 0.492 |  | 2.636 |  | 0.000 |  | 0.016 |  | 0.570 |  | 0.055 |  | 0.035 |  | 0.007 |  | 0.002 |  | 0.304 |  | 0.303 |  |
|  |  | 8 |  | 0.402 |  | 2.916 |  | 0.000 |  | 0.010 |  | 0.130 |  | 0.722 |  | 0.020 |  | 0.017 |  | 0.000 |  | 0.399 |  | 0.168 |  |
|  |  | 9 |  | 0.227 |  | 3.877 |  | 0.000 |  | 0.064 |  | 0.080 |  | 0.001 |  | 0.252 |  | 0.853 |  | 0.630 |  | 0.040 |  | 0.011 |  |
|  | | | | | | | | | | | | | | | | | | | | | | | | | |
|  |  |  |  |  |  |  |  |  |  |  |  |  |  |  |  |  |  |  |  |  |  |  |  |  |  |
| --- | --- | --- | --- | --- | --- | --- | --- | --- | --- | --- | --- | --- | --- | --- | --- | --- | --- | --- | --- | --- | --- | --- | --- | --- | --- |
| *Note.*  The intercept model is omitted, as no meaningful information can be shown. | | | | | | | | | | | | | | | | | | | | | | | | | |

| Casewise Diagnostics | | | | | | | | | | | |
| --- | --- | --- | --- | --- | --- | --- | --- | --- | --- | --- | --- |
| Case Number | | Std. Residual | | RESIL | | Predicted Value | | Residual | | Cook's Distance | |
| 112 |  | 3.374 |  | 5.930 |  | -15.965 |  | 21.895 |  | 0.058 |  |
| 170 |  | -3.252 |  | -8.070 |  | 13.299 |  | -21.369 |  | 0.024 |  |
|  | | | | | | | | | | | |

| Residuals Statistics | | | | | | | | | | | |
| --- | --- | --- | --- | --- | --- | --- | --- | --- | --- | --- | --- |
|  | | Minimum | | Maximum | | Mean | | SD | | N | |
| Predicted Value |  | -47.805 |  | 18.142 |  | -0.061 |  | 9.046 |  | 324 |  |
| Residual |  | -21.369 |  | 21.895 |  | 8.209e -17 |  | 6.555 |  | 324 |  |
| Std. Predicted Value |  | -5.278 |  | 2.012 |  | 1.474e -17 |  | 1.000 |  | 324 |  |
| Std. Residual |  | -3.252 |  | 3.374 |  | -2.794e  -4 |  | 1.001 |  | 324 |  |
|  | | | | | | | | | | | |

### Q-Q Plot Standardized Residuals

The balanced distribution of the residuals around the baseline suggests that the assumption of homoscedasticity has not been violated. The Q-Q plot shows that the standardized residuals fit along the diagonal suggesting that both assumptions or normality and linearity have also not been violated.

## SE, LOT, SC, STS, BU, CS, AV, AP as predictors of RESIL. Bayesian inference

| Model Comparison - RESIL | | | | | | | | | | | |
| --- | --- | --- | --- | --- | --- | --- | --- | --- | --- | --- | --- |
| Models | | P(M) | | P(M|data) | | BF M | | BF 10 | | R² | |
| Null model |  | 0.111 |  | 7.536e -66 |  | 6.029e -65 |  | 1.000 |  | 0.000 |  |
| AV + AP + SE + LOT + SC + BU + STS + CS |  | 0.111 |  | 0.695 |  | 18.225 |  | 9.221e +64 |  | 0.656 |  |
| AP + SE + LOT + SC + BU + STS + CS |  | 0.014 |  | 0.158 |  | 13.343 |  | 1.679e +65 |  | 0.652 |  |
| AV + AP + SE + LOT + SC + BU + CS |  | 0.014 |  | 0.053 |  | 3.982 |  | 5.637e +64 |  | 0.650 |  |
| AP + SE + LOT + SC + BU + CS |  | 0.004 |  | 0.050 |  | 13.334 |  | 1.874e +65 |  | 0.647 |  |
| AV + AP + SE + SC + BU + STS + CS |  | 0.014 |  | 0.012 |  | 0.868 |  | 1.283e +64 |  | 0.646 |  |
| AP + SE + SC + BU + STS + CS |  | 0.004 |  | 0.005 |  | 1.347 |  | 1.983e +64 |  | 0.642 |  |
| AP + SE + LOT + SC + BU + STS |  | 0.004 |  | 0.005 |  | 1.166 |  | 1.718e +64 |  | 0.642 |  |
| AV + AP + SE + LOT + SC + BU + STS |  | 0.014 |  | 0.004 |  | 0.301 |  | 4.484e +63 |  | 0.644 |  |
| AV + AP + SE + LOT + SC + CS |  | 0.004 |  | 0.003 |  | 0.860 |  | 1.268e +64 |  | 0.641 |  |
|  | | | | | | | | | | | |
|  |  |  |  |  |  |  |  |  |  |  |  |
| --- | --- | --- | --- | --- | --- | --- | --- | --- | --- | --- | --- |
| *Note.*  Table displays only a subset of models; to see all models, select "No" under "Limit No. Models Shown". | | | | | | | | | | | |

### Posterior Summary

| Posterior Summaries of Coefficients | | | | | | | | | | | | | | | |
| --- | --- | --- | --- | --- | --- | --- | --- | --- | --- | --- | --- | --- | --- | --- | --- |
|  | | | | | | | | | | | | 95% Credible Interval | | | |
| Coefficient | | Mean | | SD | | P(incl) | | P(incl|data) | | BF inclusion | | Lower | | Upper | |
| Intercept |  | -0.061 |  | 0.370 |  | 1.000 |  | 1.000 |  | 1.000 |  | -0.823 |  | 0.613 |  |
| AV |  | -0.154 |  | 0.130 |  | 0.500 |  | 0.774 |  | 3.418 |  | -0.404 |  | 0.006 |  |
| AP |  | 0.266 |  | 0.075 |  | 0.500 |  | 0.997 |  | 361.183 |  | 0.123 |  | 0.428 |  |
| SE |  | 1.031 |  | 0.127 |  | 0.500 |  | 1.000 |  | 9.503e +12 |  | 0.791 |  | 1.275 |  |
| LOT |  | 0.278 |  | 0.105 |  | 0.500 |  | 0.978 |  | 44.161 |  | 0.077 |  | 0.498 |  |
| SC |  | 0.215 |  | 0.058 |  | 0.500 |  | 0.999 |  | 781.193 |  | 0.102 |  | 0.324 |  |
| BU |  | -0.434 |  | 0.137 |  | 0.500 |  | 0.992 |  | 125.099 |  | -0.720 |  | -0.166 |  |
| STS |  | 0.201 |  | 0.118 |  | 0.500 |  | 0.886 |  | 7.781 |  | 0.000 |  | 0.400 |  |
| CS |  | 0.338 |  | 0.112 |  | 0.500 |  | 0.990 |  | 94.866 |  | 0.109 |  | 0.562 |  |
|  | | | | | | | | | | | | | | | |

#### Posterior Coefficients with 95% Credible Interval

### Residuals vs Fitted

### Inclusion Probabilities

### Q-Q Plot

## Mediation Analysis

### Parameter estimates

| Direct effects | | | | | | | | | | | | | | | | | |
| --- | --- | --- | --- | --- | --- | --- | --- | --- | --- | --- | --- | --- | --- | --- | --- | --- | --- |
|  | | | | | | | | | | | | | | 95% Confidence Interval | | | |
|  | |  | |  | | Estimate | | Std. Error | | z-value | | p | | Lower | | Upper | |
| PSS |  | → |  | RESIL |  | -0.016 |  | 0.081 |  | -0.202 |  | 0.840 |  | -0.183 |  | 0.142 |  |
| RSQ |  | → |  | RESIL |  | 0.040 |  | 0.058 |  | 0.689 |  | 0.491 |  | -0.070 |  | 0.150 |  |
|  | | | | | | | | | | | | | | | | | |
|  |  |  |  |  |  |  |  |  |  |  |  |  |  |  |  |  |  |
| --- | --- | --- | --- | --- | --- | --- | --- | --- | --- | --- | --- | --- | --- | --- | --- | --- | --- |
| *Note.*  Delta method standard errors, bias-corrected percentile bootstrap confidence intervals, ML estimator. | | | | | | | | | | | | | | | | | |

| Indirect effects | | | | | | | | | | | | | | | | | | | | | |
| --- | --- | --- | --- | --- | --- | --- | --- | --- | --- | --- | --- | --- | --- | --- | --- | --- | --- | --- | --- | --- | --- |
|  | | | | | | | | | | | | | | | | | | 95% Confidence Interval | | | |
|  | |  | |  | |  | |  | | Estimate | | Std. Error | | z-value | | p | | Lower | | Upper | |
| PSS |  | → |  | SE |  | → |  | RESIL |  | -0.284 |  | 0.048 |  | -5.940 |  | < .001 |  | -0.410 |  | -0.187 |  |
| PSS |  | → |  | LOT |  | → |  | RESIL |  | -0.106 |  | 0.034 |  | -3.090 |  | 0.002 |  | -0.198 |  | -0.042 |  |
| PSS |  | → |  | SC |  | → |  | RESIL |  | -0.178 |  | 0.044 |  | -4.009 |  | < .001 |  | -0.285 |  | -0.094 |  |
| PSS |  | → |  | CS |  | → |  | RESIL |  | -0.099 |  | 0.034 |  | -2.927 |  | 0.003 |  | -0.184 |  | -0.044 |  |
| PSS |  | → |  | BU |  | → |  | RESIL |  | -0.156 |  | 0.044 |  | -3.542 |  | < .001 |  | -0.255 |  | -0.077 |  |
| PSS |  | → |  | STS |  | → |  | RESIL |  | 0.061 |  | 0.027 |  | 2.253 |  | 0.024 |  | 0.014 |  | 0.117 |  |
| RSQ |  | → |  | SE |  | → |  | RESIL |  | -0.077 |  | 0.030 |  | -2.543 |  | 0.011 |  | -0.140 |  | -0.025 |  |
| RSQ |  | → |  | LOT |  | → |  | RESIL |  | -0.045 |  | 0.018 |  | -2.541 |  | 0.011 |  | -0.095 |  | -0.017 |  |
| RSQ |  | → |  | SC |  | → |  | RESIL |  | -0.037 |  | 0.017 |  | -2.124 |  | 0.034 |  | -0.081 |  | -0.010 |  |
| RSQ |  | → |  | CS |  | → |  | RESIL |  | -0.013 |  | 0.014 |  | -0.904 |  | 0.366 |  | -0.042 |  | 0.009 |  |
| RSQ |  | → |  | BU |  | → |  | RESIL |  | -0.086 |  | 0.028 |  | -3.118 |  | 0.002 |  | -0.151 |  | -0.043 |  |
| RSQ |  | → |  | STS |  | → |  | RESIL |  | 0.033 |  | 0.016 |  | 2.088 |  | 0.037 |  | 0.006 |  | 0.074 |  |
|  | | | | | | | | | | | | | | | | | | | | | |
|  |  |  |  |  |  |  |  |  |  |  |  |  |  |  |  |  |  |  |  |  |  |
| --- | --- | --- | --- | --- | --- | --- | --- | --- | --- | --- | --- | --- | --- | --- | --- | --- | --- | --- | --- | --- | --- |
| *Note.*  Delta method standard errors, bias-corrected percentile bootstrap confidence intervals, ML estimator. | | | | | | | | | | | | | | | | | | | | | |

| Total effects | | | | | | | | | | | | | | | | | |
| --- | --- | --- | --- | --- | --- | --- | --- | --- | --- | --- | --- | --- | --- | --- | --- | --- | --- |
|  | | | | | | | | | | | | | | 95% Confidence Interval | | | |
|  | |  | |  | | Estimate | | Std. Error | | z-value | | p | | Lower | | Upper | |
| PSS |  | → |  | RESIL |  | -0.779 |  | 0.097 |  | -8.053 |  | < .001 |  | -1.044 |  | -0.541 |  |
| RSQ |  | → |  | RESIL |  | -0.184 |  | 0.080 |  | -2.303 |  | 0.021 |  | -0.334 |  | -0.037 |  |
|  | | | | | | | | | | | | | | | | | |
|  |  |  |  |  |  |  |  |  |  |  |  |  |  |  |  |  |  |
| --- | --- | --- | --- | --- | --- | --- | --- | --- | --- | --- | --- | --- | --- | --- | --- | --- | --- |
| *Note.*  Delta method standard errors, bias-corrected percentile bootstrap confidence intervals, ML estimator. | | | | | | | | | | | | | | | | | |

| Total indirect effects | | | | | | | | | | | | | | | | | |
| --- | --- | --- | --- | --- | --- | --- | --- | --- | --- | --- | --- | --- | --- | --- | --- | --- | --- |
|  | | | | | | | | | | | | | | 95% Confidence Interval | | | |
|  | |  | |  | | Estimate | | Std. Error | | z-value | | p | | Lower | | Upper | |
| PSS |  | → |  | RESIL |  | -0.763 |  | 0.085 |  | -8.949 |  | < .001 |  | -1.007 |  | -0.557 |  |
| RSQ |  | → |  | RESIL |  | -0.224 |  | 0.062 |  | -3.612 |  | < .001 |  | -0.359 |  | -0.119 |  |
|  | | | | | | | | | | | | | | | | | |
|  |  |  |  |  |  |  |  |  |  |  |  |  |  |  |  |  |  |
| --- | --- | --- | --- | --- | --- | --- | --- | --- | --- | --- | --- | --- | --- | --- | --- | --- | --- |
| *Note.*  Delta method standard errors, bias-corrected percentile bootstrap confidence intervals, ML estimator. | | | | | | | | | | | | | | | | | |

| Residual covariances | | | | | | | | | | | | | | | | | |
| --- | --- | --- | --- | --- | --- | --- | --- | --- | --- | --- | --- | --- | --- | --- | --- | --- | --- |
|  | | | | | | | | | | | | | | 95% Confidence Interval | | | |
|  | |  | |  | | Estimate | | Std. Error | | z-value | | p | | Lower | | Upper | |
| SE |  | ↔ |  | LOT |  | 4.232 |  | 0.809 |  | 5.231 |  | < .001 |  | 2.623 |  | 6.169 |  |
| SE |  | ↔ |  | SC |  | 6.862 |  | 1.402 |  | 4.893 |  | < .001 |  | 4.029 |  | 9.880 |  |
| LOT |  | ↔ |  | SC |  | 12.512 |  | 1.908 |  | 6.556 |  | < .001 |  | 8.818 |  | 18.113 |  |
| SE |  | ↔ |  | CS |  | 4.411 |  | 0.882 |  | 5.001 |  | < .001 |  | 2.669 |  | 6.537 |  |
| LOT |  | ↔ |  | CS |  | 4.055 |  | 1.138 |  | 3.562 |  | < .001 |  | 1.853 |  | 6.724 |  |
| SC |  | ↔ |  | CS |  | 8.505 |  | 2.002 |  | 4.248 |  | < .001 |  | 4.605 |  | 13.172 |  |
| SE |  | ↔ |  | BU |  | -3.794 |  | 0.790 |  | -4.802 |  | < .001 |  | -5.708 |  | -2.364 |  |
| LOT |  | ↔ |  | BU |  | -5.877 |  | 1.055 |  | -5.572 |  | < .001 |  | -8.757 |  | -3.865 |  |
| SC |  | ↔ |  | BU |  | -11.405 |  | 1.860 |  | -6.132 |  | < .001 |  | -16.312 |  | -7.624 |  |
| CS |  | ↔ |  | BU |  | -12.158 |  | 1.288 |  | -9.436 |  | < .001 |  | -15.951 |  | -9.582 |  |
| SE |  | ↔ |  | STS |  | -1.758 |  | 0.729 |  | -2.411 |  | 0.016 |  | -3.187 |  | -0.326 |  |
| LOT |  | ↔ |  | STS |  | -0.160 |  | 0.952 |  | -0.168 |  | 0.866 |  | -2.003 |  | 1.698 |  |
| SC |  | ↔ |  | STS |  | -2.717 |  | 1.666 |  | -1.630 |  | 0.103 |  | -6.091 |  | 0.639 |  |
| CS |  | ↔ |  | STS |  | -0.114 |  | 1.042 |  | -0.110 |  | 0.913 |  | -2.182 |  | 2.091 |  |
| BU |  | ↔ |  | STS |  | 5.203 |  | 0.980 |  | 5.310 |  | < .001 |  | 3.050 |  | 7.517 |  |
|  | | | | | | | | | | | | | | | | | |
|  |  |  |  |  |  |  |  |  |  |  |  |  |  |  |  |  |  |
| --- | --- | --- | --- | --- | --- | --- | --- | --- | --- | --- | --- | --- | --- | --- | --- | --- | --- |
| *Note.*  Delta method standard errors, bias-corrected percentile bootstrap confidence intervals, ML estimator. | | | | | | | | | | | | | | | | | |

| R-Squared | | | |
| --- | --- | --- | --- |
|  | | R² | |
| RESIL |  | 0.643 |  |
| SE |  | 0.235 |  |
| LOT |  | 0.242 |  |
| SC |  | 0.291 |  |
| CS |  | 0.128 |  |
| BU |  | 0.261 |  |
| STS |  | 0.207 |  |
|  | | | |

### Path plot

## SEM. Testing Full model

| Chi Square Test Statistic (unscaled) | | | | | | | | | | | |
| --- | --- | --- | --- | --- | --- | --- | --- | --- | --- | --- | --- |
|  | | df | | AIC | | BIC | | χ² | | p | |
| Model |  | 0.000 |  |  |  |  |  | 1.165e -13 |  |  |  |
|  | | | | | | | | | | | |

| Parameter Estimates | | | | | | | | | | | | | | | | | | | | | | | | | | | |
| --- | --- | --- | --- | --- | --- | --- | --- | --- | --- | --- | --- | --- | --- | --- | --- | --- | --- | --- | --- | --- | --- | --- | --- | --- | --- | --- | --- |
|  | |  | |  | | label | | est | | se | | z | | p | | CI (lower) | | CI (upper) | | std (lv) | | std (all) | | std (nox) | | group | |
| RESIL |  | ~ |  | SE |  | b11 |  | 1.094 |  | 0.135 |  | 8.103 |  | < .001 |  | 0.825 |  | 1.349 |  | 1.094 |  | 0.364 |  | 0.364 |  |  |  |
| RESIL |  | ~ |  | LOT |  | b12 |  | 0.335 |  | 0.113 |  | 2.963 |  | 0.003 |  | 0.119 |  | 0.551 |  | 0.335 |  | 0.147 |  | 0.147 |  |  |  |
| RESIL |  | ~ |  | SC |  | b13 |  | 0.249 |  | 0.061 |  | 4.046 |  | < .001 |  | 0.118 |  | 0.361 |  | 0.249 |  | 0.197 |  | 0.197 |  |  |  |
| RESIL |  | ~ |  | STS |  | b14 |  | 0.246 |  | 0.099 |  | 2.488 |  | 0.013 |  | 0.064 |  | 0.446 |  | 0.246 |  | 0.099 |  | 0.099 |  |  |  |
| RESIL |  | ~ |  | BU |  | b15 |  | -0.519 |  | 0.118 |  | -4.388 |  | < .001 |  | -0.760 |  | -0.292 |  | -0.519 |  | -0.227 |  | -0.227 |  |  |  |
| RESIL |  | ~ |  | CS |  | b16 |  | 0.351 |  | 0.098 |  | 3.575 |  | < .001 |  | 0.150 |  | 0.538 |  | 0.351 |  | 0.158 |  | 0.158 |  |  |  |
| RESIL |  | ~ |  | PSS |  | c11 |  | -0.016 |  | 0.079 |  | -0.207 |  | 0.836 |  | -0.175 |  | 0.130 |  | -0.016 |  | -0.009 |  | -0.001 |  |  |  |
| RESIL |  | ~ |  | RSQ |  | c12 |  | 0.040 |  | 0.058 |  | 0.682 |  | 0.495 |  | -0.082 |  | 0.146 |  | 0.040 |  | 0.026 |  | 0.004 |  |  |  |
| SE |  | ~ |  | PSS |  | a11 |  | -0.260 |  | 0.041 |  | -6.410 |  | < .001 |  | -0.347 |  | -0.183 |  | -0.260 |  | -0.420 |  | -0.070 |  |  |  |
| SE |  | ~ |  | RSQ |  | a12 |  | -0.070 |  | 0.025 |  | -2.843 |  | 0.004 |  | -0.117 |  | -0.025 |  | -0.070 |  | -0.137 |  | -0.019 |  |  |  |
| LOT |  | ~ |  | PSS |  | a21 |  | -0.317 |  | 0.050 |  | -6.298 |  | < .001 |  | -0.413 |  | -0.222 |  | -0.317 |  | -0.387 |  | -0.064 |  |  |  |
| LOT |  | ~ |  | RSQ |  | a22 |  | -0.133 |  | 0.033 |  | -4.084 |  | < .001 |  | -0.197 |  | -0.068 |  | -0.133 |  | -0.198 |  | -0.027 |  |  |  |
| SC |  | ~ |  | PSS |  | a31 |  | -0.716 |  | 0.079 |  | -9.037 |  | < .001 |  | -0.885 |  | -0.563 |  | -0.716 |  | -0.486 |  | -0.081 |  |  |  |
| SC |  | ~ |  | RSQ |  | a32 |  | -0.147 |  | 0.061 |  | -2.403 |  | 0.016 |  | -0.264 |  | -0.023 |  | -0.147 |  | -0.121 |  | -0.017 |  |  |  |
| STS |  | ~ |  | PSS |  | a41 |  | 0.247 |  | 0.042 |  | 5.935 |  | < .001 |  | 0.165 |  | 0.325 |  | 0.247 |  | 0.330 |  | 0.055 |  |  |  |
| STS |  | ~ |  | RSQ |  | a42 |  | 0.135 |  | 0.035 |  | 3.873 |  | < .001 |  | 0.070 |  | 0.207 |  | 0.135 |  | 0.219 |  | 0.030 |  |  |  |
| BU |  | ~ |  | PSS |  | a51 |  | 0.302 |  | 0.050 |  | 5.987 |  | < .001 |  | 0.204 |  | 0.399 |  | 0.302 |  | 0.370 |  | 0.061 |  |  |  |
| BU |  | ~ |  | RSQ |  | a52 |  | 0.167 |  | 0.038 |  | 4.400 |  | < .001 |  | 0.097 |  | 0.250 |  | 0.167 |  | 0.248 |  | 0.034 |  |  |  |
| CS |  | ~ |  | PSS |  | a61 |  | -0.281 |  | 0.055 |  | -5.131 |  | < .001 |  | -0.392 |  | -0.184 |  | -0.281 |  | -0.337 |  | -0.056 |  |  |  |
| CS |  | ~ |  | RSQ |  | a62 |  | -0.036 |  | 0.037 |  | -0.964 |  | 0.335 |  | -0.108 |  | 0.037 |  | -0.036 |  | -0.052 |  | -0.007 |  |  |  |
| SE |  | ~~ |  | LOT |  |  |  | 4.245 |  | 0.857 |  | 4.955 |  | < .001 |  | 2.525 |  | 5.876 |  | 4.245 |  | 0.303 |  | 0.303 |  |  |  |
| SE |  | ~~ |  | SC |  |  |  | 6.883 |  | 1.519 |  | 4.532 |  | < .001 |  | 3.936 |  | 9.885 |  | 6.883 |  | 0.282 |  | 0.282 |  |  |  |
| LOT |  | ~~ |  | SC |  |  |  | 12.551 |  | 2.261 |  | 5.552 |  | < .001 |  | 8.369 |  | 17.310 |  | 12.551 |  | 0.390 |  | 0.390 |  |  |  |
| SE |  | ~~ |  | STS |  |  |  | -1.764 |  | 0.739 |  | -2.386 |  | 0.017 |  | -3.235 |  | -0.284 |  | -1.764 |  | -0.135 |  | -0.135 |  |  |  |
| LOT |  | ~~ |  | STS |  |  |  | -0.161 |  | 0.917 |  | -0.175 |  | 0.861 |  | -2.093 |  | 1.519 |  | -0.161 |  | -0.009 |  | -0.009 |  |  |  |
| SC |  | ~~ |  | STS |  |  |  | -2.725 |  | 1.706 |  | -1.597 |  | 0.110 |  | -6.035 |  | 0.524 |  | -2.725 |  | -0.091 |  | -0.091 |  |  |  |
| SE |  | ~~ |  | BU |  |  |  | -3.806 |  | 0.845 |  | -4.506 |  | < .001 |  | -5.542 |  | -2.126 |  | -3.806 |  | -0.276 |  | -0.276 |  |  |  |
| LOT |  | ~~ |  | BU |  |  |  | -5.895 |  | 1.299 |  | -4.539 |  | < .001 |  | -8.472 |  | -3.454 |  | -5.895 |  | -0.325 |  | -0.325 |  |  |  |
| SC |  | ~~ |  | BU |  |  |  | -11.440 |  | 2.035 |  | -5.622 |  | < .001 |  | -15.242 |  | -7.520 |  | -11.440 |  | -0.362 |  | -0.362 |  |  |  |
| STS |  | ~~ |  | BU |  |  |  | 5.219 |  | 1.204 |  | 4.336 |  | < .001 |  | 2.905 |  | 7.596 |  | 5.219 |  | 0.308 |  | 0.308 |  |  |  |
| SE |  | ~~ |  | CS |  |  |  | 4.424 |  | 1.013 |  | 4.368 |  | < .001 |  | 2.379 |  | 6.490 |  | 4.424 |  | 0.289 |  | 0.289 |  |  |  |
| LOT |  | ~~ |  | CS |  |  |  | 4.068 |  | 1.309 |  | 3.106 |  | 0.002 |  | 1.533 |  | 6.649 |  | 4.068 |  | 0.202 |  | 0.202 |  |  |  |
| SC |  | ~~ |  | CS |  |  |  | 8.532 |  | 2.199 |  | 3.879 |  | < .001 |  | 4.167 |  | 12.898 |  | 8.532 |  | 0.242 |  | 0.242 |  |  |  |
| STS |  | ~~ |  | CS |  |  |  | -0.114 |  | 1.124 |  | -0.102 |  | 0.919 |  | -2.352 |  | 2.221 |  | -0.114 |  | -0.006 |  | -0.006 |  |  |  |
| BU |  | ~~ |  | CS |  |  |  | -12.195 |  | 1.541 |  | -7.913 |  | < .001 |  | -15.401 |  | -9.148 |  | -12.195 |  | -0.614 |  | -0.614 |  |  |  |
| RESIL |  | ~~ |  | RESIL |  |  |  | 44.896 |  | 3.770 |  | 11.910 |  | < .001 |  | 36.517 |  | 51.390 |  | 44.896 |  | 0.357 |  | 0.357 |  |  |  |
| SE |  | ~~ |  | SE |  |  |  | 10.629 |  | 0.787 |  | 13.502 |  | < .001 |  | 8.954 |  | 12.134 |  | 10.629 |  | 0.765 |  | 0.765 |  |  |  |
| LOT |  | ~~ |  | LOT |  |  |  | 18.437 |  | 1.529 |  | 12.057 |  | < .001 |  | 15.313 |  | 21.471 |  | 18.437 |  | 0.758 |  | 0.758 |  |  |  |
| SC |  | ~~ |  | SC |  |  |  | 56.049 |  | 4.410 |  | 12.709 |  | < .001 |  | 47.024 |  | 64.590 |  | 56.049 |  | 0.709 |  | 0.709 |  |  |  |
| STS |  | ~~ |  | STS |  |  |  | 16.069 |  | 1.472 |  | 10.915 |  | < .001 |  | 13.198 |  | 18.755 |  | 16.069 |  | 0.793 |  | 0.793 |  |  |  |
| BU |  | ~~ |  | BU |  |  |  | 17.844 |  | 1.541 |  | 11.577 |  | < .001 |  | 14.799 |  | 20.665 |  | 17.844 |  | 0.739 |  | 0.739 |  |  |  |
| CS |  | ~~ |  | CS |  |  |  | 22.087 |  | 2.017 |  | 10.950 |  | < .001 |  | 18.101 |  | 26.061 |  | 22.087 |  | 0.872 |  | 0.872 |  |  |  |
| PSS |  | ~~ |  | PSS |  |  |  | 36.365 |  | 0.000 |  |  |  |  |  | 36.365 |  | 36.365 |  | 36.365 |  | 1.000 |  | 36.365 |  |  |  |
| PSS |  | ~~ |  | RSQ |  |  |  | 15.159 |  | 0.000 |  |  |  |  |  | 15.159 |  | 15.159 |  | 15.159 |  | 0.344 |  | 15.159 |  |  |  |
| RSQ |  | ~~ |  | RSQ |  |  |  | 53.394 |  | 0.000 |  |  |  |  |  | 53.394 |  | 53.394 |  | 53.394 |  | 1.000 |  | 53.394 |  |  |  |
| RESIL |  | ~1 |  |  |  |  |  | -0.022 |  | 0.377 |  | -0.058 |  | 0.954 |  | -0.821 |  | 0.681 |  | -0.022 |  | -0.002 |  | -0.002 |  |  |  |
| SE |  | ~1 |  |  |  |  |  | 0.020 |  | 0.178 |  | 0.110 |  | 0.913 |  | -0.357 |  | 0.371 |  | 0.020 |  | 0.005 |  | 0.005 |  |  |  |
| LOT |  | ~1 |  |  |  |  |  | 0.008 |  | 0.241 |  | 0.032 |  | 0.974 |  | -0.437 |  | 0.502 |  | 0.008 |  | 0.002 |  | 0.002 |  |  |  |
| SC |  | ~1 |  |  |  |  |  | 0.009 |  | 0.423 |  | 0.021 |  | 0.983 |  | -0.818 |  | 0.834 |  | 0.009 |  | 9.924e -4 |  | 9.924e -4 |  |  |  |
| STS |  | ~1 |  |  |  |  |  | 0.006 |  | 0.218 |  | 0.028 |  | 0.977 |  | -0.398 |  | 0.472 |  | 0.006 |  | 0.001 |  | 0.001 |  |  |  |
| BU |  | ~1 |  |  |  |  |  | 0.002 |  | 0.232 |  | 0.009 |  | 0.993 |  | -0.422 |  | 0.472 |  | 0.002 |  | 4.063e -4 |  | 4.063e -4 |  |  |  |
| CS |  | ~1 |  |  |  |  |  | 0.006 |  | 0.260 |  | 0.024 |  | 0.981 |  | -0.528 |  | 0.518 |  | 0.006 |  | 0.001 |  | 0.001 |  |  |  |
| PSS |  | ~1 |  |  |  |  |  | 0.002 |  | 0.000 |  |  |  |  |  | 0.002 |  | 0.002 |  | 0.002 |  | 3.827e -4 |  | 0.002 |  |  |  |
| RSQ |  | ~1 |  |  |  |  |  | 0.007 |  | 0.000 |  |  |  |  |  | 0.007 |  | 0.007 |  | 0.007 |  | 9.474e -4 |  | 0.007 |  |  |  |
| ind\_x1\_m1\_y1 |  | := |  | a11\*b11 |  | ind\_x1\_m1\_y1 |  | -0.284 |  | 0.056 |  | -5.074 |  | < .001 |  | -0.399 |  | -0.182 |  | -0.284 |  | -0.153 |  | -0.025 |  |  |  |
| ind\_x1\_m2\_y1 |  | := |  | a21\*b12 |  | ind\_x1\_m2\_y1 |  | -0.106 |  | 0.040 |  | -2.660 |  | 0.008 |  | -0.190 |  | -0.037 |  | -0.106 |  | -0.057 |  | -0.009 |  |  |  |
| ind\_x1\_m3\_y1 |  | := |  | a31\*b13 |  | ind\_x1\_m3\_y1 |  | -0.178 |  | 0.048 |  | -3.684 |  | < .001 |  | -0.274 |  | -0.087 |  | -0.178 |  | -0.096 |  | -0.016 |  |  |  |
| ind\_x1\_m4\_y1 |  | := |  | a41\*b14 |  | ind\_x1\_m4\_y1 |  | 0.061 |  | 0.026 |  | 2.314 |  | 0.021 |  | 0.015 |  | 0.114 |  | 0.061 |  | 0.033 |  | 0.005 |  |  |  |
| ind\_x1\_m5\_y1 |  | := |  | a51\*b15 |  | ind\_x1\_m5\_y1 |  | -0.156 |  | 0.045 |  | -3.465 |  | < .001 |  | -0.250 |  | -0.077 |  | -0.156 |  | -0.084 |  | -0.014 |  |  |  |
| ind\_x1\_m6\_y1 |  | := |  | a61\*b16 |  | ind\_x1\_m6\_y1 |  | -0.099 |  | 0.033 |  | -2.966 |  | 0.003 |  | -0.169 |  | -0.040 |  | -0.099 |  | -0.053 |  | -0.009 |  |  |  |
| ind\_x1\_y1 |  | := |  | ind\_x1\_m1\_y1+ind\_x1\_m2\_y1+ind\_x1\_m3\_y1+ind\_x1\_m4\_y1+ind\_x1\_m5\_y1+ind\_x1\_m6\_y1 |  | ind\_x1\_y1 |  | -0.763 |  | 0.107 |  | -7.134 |  | < .001 |  | -0.986 |  | -0.556 |  | -0.763 |  | -0.410 |  | -0.068 |  |  |  |
| tot\_x1\_y1 |  | := |  | ind\_x1\_y1+c11 |  | tot\_x1\_y1 |  | -0.779 |  | 0.122 |  | -6.397 |  | < .001 |  | -1.030 |  | -0.547 |  | -0.779 |  | -0.419 |  | -0.069 |  |  |  |
| ind\_x2\_m1\_y1 |  | := |  | a12\*b11 |  | ind\_x2\_m1\_y1 |  | -0.077 |  | 0.029 |  | -2.685 |  | 0.007 |  | -0.135 |  | -0.025 |  | -0.077 |  | -0.050 |  | -0.007 |  |  |  |
| ind\_x2\_m2\_y1 |  | := |  | a22\*b12 |  | ind\_x2\_m2\_y1 |  | -0.045 |  | 0.019 |  | -2.346 |  | 0.019 |  | -0.087 |  | -0.014 |  | -0.045 |  | -0.029 |  | -0.004 |  |  |  |
| ind\_x2\_m3\_y1 |  | := |  | a32\*b13 |  | ind\_x2\_m3\_y1 |  | -0.037 |  | 0.017 |  | -2.144 |  | 0.032 |  | -0.074 |  | -0.005 |  | -0.037 |  | -0.024 |  | -0.003 |  |  |  |
| ind\_x2\_m4\_y1 |  | := |  | a42\*b14 |  | ind\_x2\_m4\_y1 |  | 0.033 |  | 0.017 |  | 1.931 |  | 0.053 |  | 0.006 |  | 0.072 |  | 0.033 |  | 0.022 |  | 0.003 |  |  |  |
| ind\_x2\_m5\_y1 |  | := |  | a52\*b15 |  | ind\_x2\_m5\_y1 |  | -0.086 |  | 0.028 |  | -3.095 |  | 0.002 |  | -0.147 |  | -0.039 |  | -0.086 |  | -0.056 |  | -0.008 |  |  |  |
| ind\_x2\_m6\_y1 |  | := |  | a62\*b16 |  | ind\_x2\_m6\_y1 |  | -0.013 |  | 0.014 |  | -0.927 |  | 0.354 |  | -0.039 |  | 0.014 |  | -0.013 |  | -0.008 |  | -0.001 |  |  |  |
| ind\_x2\_y1 |  | := |  | ind\_x2\_m1\_y1+ind\_x2\_m2\_y1+ind\_x2\_m3\_y1+ind\_x2\_m4\_y1+ind\_x2\_m5\_y1+ind\_x2\_m6\_y1 |  | ind\_x2\_y1 |  | -0.224 |  | 0.063 |  | -3.524 |  | < .001 |  | -0.349 |  | -0.101 |  | -0.224 |  | -0.146 |  | -0.020 |  |  |  |
| tot\_x2\_y1 |  | := |  | ind\_x2\_y1+c12 |  | tot\_x2\_y1 |  | -0.184 |  | 0.077 |  | -2.384 |  | 0.017 |  | -0.339 |  | -0.044 |  | -0.184 |  | -0.120 |  | -0.016 |  |  |  |
|  | | | | | | | | | | | | | | | | | | | | | | | | | | | |

| Model test baseline model | | | |
| --- | --- | --- | --- |
|  | | Model | |
| Minimum Function Test Statistic |  | 1.792e -16 |  |
| χ² |  | 1.165e -13 |  |
| Degrees of freedom |  | 0.000 |  |
| p |  |  |  |
|  | | | |

| User model versus baseline model | | | |
| --- | --- | --- | --- |
|  | | Model | |
| Comparative Fit Index (CFI) |  | 1.000 |  |
| Tucker-Lewis Index (TLI) |  | 1.000 |  |
| Bentler-Bonett Non-normed Fit Index (NNFI) |  | 1.000 |  |
| Bentler-Bonett Normed Fit Index (NFI) |  | 1.000 |  |
| Parsimony Normed Fit Index (PNFI) |  | 0.000 |  |
| Bollen's Relative Fit Index (RFI) |  | 1.000 |  |
| Bollen's Incremental Fit Index (IFI) |  | 1.000 |  |
| Relative Noncentrality Index (RNI) |  | 1.000 |  |
|  | | | |

This does not look good. Rather indicates that the model is overfitted

| Loglikelihood and Information Criteria | | | |
| --- | --- | --- | --- |
|  | | Model | |
| Loglikelihood user model (H0) |  |  |  |
| Loglikelihood unrestricted model (H1) |  |  |  |
| Number of free parameters |  | 49 |  |
| Akaike (AIC) |  |  |  |
| Bayesian (BIC) |  |  |  |
| Sample-size adjusted Bayesian (BIC) |  |  |  |
| NA |  |  |  |
|  | | | |

Information criteria could not be estimated, possibly too big

| Root Mean Square Error of Approximation | | | |
| --- | --- | --- | --- |
|  | | Model | |
| RMSEA |  | 0.000 |  |
| Upper 90% CI |  | 0.000 |  |
| Lower 90% CI |  | 0.000 |  |
| p-value RMSEA <= 0.05 |  |  |  |
|  | | | |

RMSEA penalizes for model complexity, but could not be estimated

| Standardized Root Mean Square Residual | | | |
| --- | --- | --- | --- |
|  | | Model | |
| RMR |  | 1.208e -7 |  |
| RMR (No Mean) |  | 1.324e -7 |  |
| SRMR |  | 3.135e -9 |  |
|  | | | |

| Other Fit Indices | | | |
| --- | --- | --- | --- |
|  | | Model | |
| Hoelter Critical N (CN) alpha=0.05 |  | 1.000 |  |
| Hoelter Critical N (CN) alpha=0.01 |  | 1.000 |  |
| Goodness of Fit Index (GFI) |  | 1.000 |  |
| Parsimony Goodness of Fit Index (GFI) |  | 1.000 |  |
| McDonald Fit Index (MFI) |  | 1.000 |  |
|  | | | |

| R-Squared | | | |
| --- | --- | --- | --- |
| Variable | | R² | |
| RESIL |  | 0.643 |  |
| SE |  | 0.235 |  |
| LOT |  | 0.242 |  |
| SC |  | 0.291 |  |
| STS |  | 0.207 |  |
| BU |  | 0.261 |  |
| CS |  | 0.128 |  |
|  | | | |

| Covariances (lower triangle) / correlations (upper triangle) | | | | | | | | | | | | | | | | | | | | | |
| --- | --- | --- | --- | --- | --- | --- | --- | --- | --- | --- | --- | --- | --- | --- | --- | --- | --- | --- | --- | --- | --- |
|  | |  | | RESIL | | SE | | LOT | | SC | | STS | | BU | | CS | | PSS | | RSQ | |
| RESIL |  | observed |  | 125.766 |  | 0.657 |  | 0.563 |  | 0.596 |  | -0.239 |  | -0.621 |  | 0.565 |  | -0.460 |  | -0.264 |  |
|  |  | fitted |  | 125.766 |  | 0.657 |  | 0.563 |  | 0.596 |  | -0.239 |  | -0.621 |  | 0.565 |  | -0.460 |  | -0.264 |  |
|  |  | residual |  | 3.160e -7 |  | 2.743e -10 |  | 1.789e  -9 |  | 1.185e  -9 |  | 7.694e  -9 |  | 3.424e -10 |  | 1.271e  -9 |  | 4.367e -10 |  | 3.566e -10 |  |
| SE |  | observed |  | 27.457 |  | 13.897 |  | 0.468 |  | 0.469 |  | -0.321 |  | -0.451 |  | 0.408 |  | -0.467 |  | -0.282 |  |
|  |  | fitted |  | 27.457 |  | 13.897 |  | 0.468 |  | 0.469 |  | -0.321 |  | -0.451 |  | 0.408 |  | -0.467 |  | -0.282 |  |
|  |  | residual |  | 5.995e -8 |  | 1.416e  -8 |  | 2.250e  -9 |  | 2.940e -10 |  | 4.458e  -9 |  | 2.532e -10 |  | 2.980e -10 |  | 1.236e -10 |  | 1.325e -10 |  |
| LOT |  | observed |  | 31.121 |  | 8.597 |  | 24.312 |  | 0.547 |  | -0.230 |  | -0.494 |  | 0.334 |  | -0.455 |  | -0.331 |  |
|  |  | fitted |  | 31.121 |  | 8.597 |  | 24.312 |  | 0.547 |  | -0.230 |  | -0.494 |  | 0.334 |  | -0.455 |  | -0.331 |  |
|  |  | residual |  | 1.901e -7 |  | 6.012e  -8 |  | 8.137e  -8 |  | 1.813e  -9 |  | 7.208e  -9 |  | 1.629e -10 |  | 2.275e  -9 |  | 6.444e -10 |  | 5.266e -10 |  |
| SC |  | observed |  | 59.401 |  | 15.539 |  | 23.998 |  | 79.054 |  | -0.305 |  | -0.528 |  | 0.383 |  | -0.527 |  | -0.288 |  |
|  |  | fitted |  | 59.401 |  | 15.539 |  | 23.998 |  | 79.054 |  | -0.305 |  | -0.528 |  | 0.383 |  | -0.527 |  | -0.288 |  |
|  |  | residual |  | 2.124e -7 |  | 2.279e  -8 |  | 1.276e  -7 |  | 5.222e  -8 |  | 1.180e  -8 |  | 1.378e  -9 |  | 3.264e  -9 |  | -4.606e -12 |  | 1.034e -10 |  |
| STS |  | observed |  | -12.053 |  | -5.392 |  | -5.108 |  | -12.223 |  | 20.262 |  | 0.468 |  | -0.159 |  | 0.406 |  | 0.333 |  |
|  |  | fitted |  | -12.053 |  | -5.392 |  | -5.108 |  | -12.223 |  | 20.262 |  | 0.468 |  | -0.159 |  | 0.406 |  | 0.333 |  |
|  |  | residual |  | 3.973e -7 |  | 8.281e  -8 |  | 1.616e  -7 |  | 4.926e  -7 |  | -8.076e  -8 |  | -6.904e  -9 |  | 5.959e  -9 |  | 8.310e -10 |  | 5.923e -10 |  |
| BU |  | observed |  | -34.226 |  | -8.254 |  | -11.962 |  | -23.082 |  | 10.363 |  | 24.156 |  | -0.666 |  | 0.455 |  | 0.375 |  |
|  |  | fitted |  | -34.226 |  | -8.254 |  | -11.962 |  | -23.082 |  | 10.363 |  | 24.156 |  | -0.666 |  | 0.455 |  | 0.375 |  |
|  |  | residual |  | 3.387e -8 |  | 1.442e  -8 |  | 4.199e  -9 |  | 9.173e  -8 |  | -1.910e  -7 |  | -8.187e  -8 |  | -6.718e -10 |  | 9.669e -10 |  | 6.030e -10 |  |
| CS |  | observed |  | 31.896 |  | 7.652 |  | 8.298 |  | 17.147 |  | -3.601 |  | -16.468 |  | 25.331 |  | -0.355 |  | -0.168 |  |
|  |  | fitted |  | 31.896 |  | 7.652 |  | 8.298 |  | 17.147 |  | -3.601 |  | -16.468 |  | 25.331 |  | -0.355 |  | -0.168 |  |
|  |  | residual |  | 1.482e -7 |  | 1.822e  -8 |  | 7.983e  -8 |  | 1.713e  -7 |  | 1.381e  -7 |  | -7.510e  -9 |  | 5.783e  -8 |  | 2.076e -10 |  | 1.946e -10 |  |
| PSS |  | observed |  | -31.120 |  | -10.509 |  | -13.536 |  | -28.279 |  | 11.014 |  | 13.490 |  | -10.762 |  | 36.365 |  | 0.344 |  |
|  |  | fitted |  | -31.120 |  | -10.509 |  | -13.536 |  | -28.279 |  | 11.014 |  | 13.490 |  | -10.762 |  | 36.365 |  | 0.344 |  |
|  |  | residual |  | -9.566e -9 |  | -2.574e  -9 |  | -3.492e  -9 |  | -9.588e  -9 |  | 6.059e -10 |  | 5.798e  -9 |  | -5.982e  -9 |  | 0.000 |  | 0.000 |  |
| RSQ |  | observed |  | -21.629 |  | -7.681 |  | -11.921 |  | -18.698 |  | 10.945 |  | 13.467 |  | -6.166 |  | 15.159 |  | 53.394 |  |
|  |  | fitted |  | -21.629 |  | -7.681 |  | -11.921 |  | -18.698 |  | 10.945 |  | 13.467 |  | -6.166 |  | 15.159 |  | 53.394 |  |
|  |  | residual |  | 2.046e -9 |  | -3.041e -10 |  | -9.789e -10 |  | 5.442e -10 |  | -2.332e  -9 |  | -1.166e  -9 |  | 1.193e -10 |  | 0.000 |  | 0.000 |  |
|  | | | | | | | | | | | | | | | | | | | | | |

| Modification Indices | | | | | | | | | | | | | | | |
| --- | --- | --- | --- | --- | --- | --- | --- | --- | --- | --- | --- | --- | --- | --- | --- |
|  | |  | |  | | mi | | epc | | sepc (lv) | | sepc (all) | | sepc (nox) | |
| . |  | . |  | . |  | . |  | . |  | . |  | . |  | . |  |
|  | | | | | | | | | | | | | | | |

| Mardia's coefficients | | | | | | | | | | | |
| --- | --- | --- | --- | --- | --- | --- | --- | --- | --- | --- | --- |
|  | | Coefficient | | z | | χ² | | df | | p | |
| Skewness |  | 7.080 |  |  |  | 383.517 |  | 165.000 |  | < .001 |  |
| Kurtosis |  | 105.316 |  | 4.046 |  |  |  |  |  | < .001 |  |
|  | | | | | | | | | | | |

The model is of bad fit.

## SEM. New Model 9. Best fit for parallel mediation

| Chi Square Test Statistic (unscaled) | | | | | | | | | | | |
| --- | --- | --- | --- | --- | --- | --- | --- | --- | --- | --- | --- |
|  | | df | | AIC | | BIC | | χ² | | p | |
| Model |  | 5.000 |  | 13248.232 |  | 13388.234 |  | 8.884 |  | 0.114 |  |
|  | | | | | | | | | | | |

The chi-square (χ2) test tests the null hypothesis that the predicted model and observed data are equal. Ideally, we want your predictions to match the actual data as closely as possible. Therefore, nonsignificant result for this test indicates good model fit.

| Parameter Estimates | | | | | | | | | | | | | | | | | | | | | | | | | | | |
| --- | --- | --- | --- | --- | --- | --- | --- | --- | --- | --- | --- | --- | --- | --- | --- | --- | --- | --- | --- | --- | --- | --- | --- | --- | --- | --- | --- |
|  | |  | |  | | label | | est | | se | | z | | p | | CI (lower) | | CI (upper) | | std (lv) | | std (all) | | std (nox) | | group | |
| RESIL |  | ~ |  | SE |  | b11 |  | 1.094 |  | 0.135 |  | 8.122 |  | < .001 |  | 0.806 |  | 1.353 |  | 1.094 |  | 0.364 |  | 0.364 |  |  |  |
| RESIL |  | ~ |  | LOT |  | b12 |  | 0.335 |  | 0.112 |  | 3.001 |  | 0.003 |  | 0.123 |  | 0.554 |  | 0.335 |  | 0.146 |  | 0.146 |  |  |  |
| RESIL |  | ~ |  | SC |  | b13 |  | 0.249 |  | 0.061 |  | 4.090 |  | < .001 |  | 0.123 |  | 0.362 |  | 0.249 |  | 0.197 |  | 0.197 |  |  |  |
| RESIL |  | ~ |  | STS |  | b14 |  | 0.246 |  | 0.099 |  | 2.494 |  | 0.013 |  | 0.049 |  | 0.437 |  | 0.246 |  | 0.099 |  | 0.099 |  |  |  |
| RESIL |  | ~ |  | BU |  | b15 |  | -0.519 |  | 0.121 |  | -4.275 |  | < .001 |  | -0.779 |  | -0.278 |  | -0.519 |  | -0.226 |  | -0.226 |  |  |  |
| RESIL |  | ~ |  | CS |  | b16 |  | 0.351 |  | 0.103 |  | 3.412 |  | < .001 |  | 0.154 |  | 0.558 |  | 0.351 |  | 0.158 |  | 0.158 |  |  |  |
| RESIL |  | ~ |  | PSS |  | c11 |  | -0.016 |  | 0.077 |  | -0.210 |  | 0.833 |  | -0.177 |  | 0.137 |  | -0.016 |  | -0.009 |  | -0.001 |  |  |  |
| RESIL |  | ~ |  | RSQ |  | c12 |  | 0.040 |  | 0.058 |  | 0.685 |  | 0.493 |  | -0.070 |  | 0.153 |  | 0.040 |  | 0.026 |  | 0.004 |  |  |  |
| SE |  | ~ |  | PSS |  | a11 |  | -0.289 |  | 0.037 |  | -7.767 |  | < .001 |  | -0.366 |  | -0.214 |  | -0.289 |  | -0.468 |  | -0.078 |  |  |  |
| LOT |  | ~ |  | PSS |  | a21 |  | -0.334 |  | 0.048 |  | -6.957 |  | < .001 |  | -0.427 |  | -0.235 |  | -0.334 |  | -0.412 |  | -0.068 |  |  |  |
| LOT |  | ~ |  | RSQ |  | a22 |  | -0.091 |  | 0.028 |  | -3.190 |  | 0.001 |  | -0.144 |  | -0.035 |  | -0.091 |  | -0.135 |  | -0.019 |  |  |  |
| SC |  | ~ |  | PSS |  | a31 |  | -0.720 |  | 0.073 |  | -9.903 |  | < .001 |  | -0.864 |  | -0.581 |  | -0.720 |  | -0.489 |  | -0.081 |  |  |  |
| STS |  | ~ |  | PSS |  | a41 |  | 0.251 |  | 0.043 |  | 5.890 |  | < .001 |  | 0.171 |  | 0.342 |  | 0.251 |  | 0.337 |  | 0.056 |  |  |  |
| STS |  | ~ |  | RSQ |  | a42 |  | 0.125 |  | 0.035 |  | 3.618 |  | < .001 |  | 0.058 |  | 0.197 |  | 0.125 |  | 0.204 |  | 0.028 |  |  |  |
| BU |  | ~ |  | PSS |  | a51 |  | 0.317 |  | 0.046 |  | 6.887 |  | < .001 |  | 0.227 |  | 0.411 |  | 0.317 |  | 0.393 |  | 0.065 |  |  |  |
| BU |  | ~ |  | RSQ |  | a52 |  | 0.130 |  | 0.028 |  | 4.630 |  | < .001 |  | 0.073 |  | 0.184 |  | 0.130 |  | 0.196 |  | 0.027 |  |  |  |
| CS |  | ~ |  | PSS |  | a61 |  | -0.296 |  | 0.051 |  | -5.769 |  | < .001 |  | -0.397 |  | -0.200 |  | -0.296 |  | -0.355 |  | -0.059 |  |  |  |
| SE |  | ~~ |  | LOT |  |  |  | 4.353 |  | 0.851 |  | 5.116 |  | < .001 |  | 2.647 |  | 6.084 |  | 4.353 |  | 0.308 |  | 0.308 |  |  |  |
| SE |  | ~~ |  | SC |  |  |  | 6.956 |  | 1.498 |  | 4.643 |  | < .001 |  | 3.935 |  | 9.737 |  | 6.956 |  | 0.282 |  | 0.282 |  |  |  |
| LOT |  | ~~ |  | SC |  |  |  | 12.725 |  | 2.343 |  | 5.432 |  | < .001 |  | 8.263 |  | 17.643 |  | 12.725 |  | 0.395 |  | 0.395 |  |  |  |
| SE |  | ~~ |  | BU |  |  |  | -3.896 |  | 0.864 |  | -4.510 |  | < .001 |  | -5.607 |  | -2.272 |  | -3.896 |  | -0.281 |  | -0.281 |  |  |  |
| LOT |  | ~~ |  | BU |  |  |  | -5.892 |  | 1.226 |  | -4.805 |  | < .001 |  | -8.427 |  | -3.524 |  | -5.892 |  | -0.325 |  | -0.325 |  |  |  |
| SC |  | ~~ |  | BU |  |  |  | -10.710 |  | 2.147 |  | -4.987 |  | < .001 |  | -15.219 |  | -6.617 |  | -10.710 |  | -0.339 |  | -0.339 |  |  |  |
| STS |  | ~~ |  | BU |  |  |  | 5.148 |  | 1.044 |  | 4.932 |  | < .001 |  | 3.161 |  | 7.260 |  | 5.148 |  | 0.305 |  | 0.305 |  |  |  |
| SE |  | ~~ |  | CS |  |  |  | 4.514 |  | 1.047 |  | 4.310 |  | < .001 |  | 2.400 |  | 6.527 |  | 4.514 |  | 0.292 |  | 0.292 |  |  |  |
| LOT |  | ~~ |  | CS |  |  |  | 4.127 |  | 1.269 |  | 3.252 |  | 0.001 |  | 1.710 |  | 6.603 |  | 4.127 |  | 0.204 |  | 0.204 |  |  |  |
| SC |  | ~~ |  | CS |  |  |  | 8.687 |  | 2.257 |  | 3.848 |  | < .001 |  | 4.340 |  | 13.224 |  | 8.687 |  | 0.247 |  | 0.247 |  |  |  |
| BU |  | ~~ |  | CS |  |  |  | -12.177 |  | 1.595 |  | -7.636 |  | < .001 |  | -15.402 |  | -9.085 |  | -12.177 |  | -0.614 |  | -0.614 |  |  |  |
| SE |  | ~~ |  | STS |  |  |  | -1.759 |  | 0.665 |  | -2.645 |  | 0.008 |  | -3.101 |  | -0.435 |  | -1.759 |  | -0.134 |  | -0.134 |  |  |  |
| SC |  | ~ |  | STS |  |  |  | -0.189 |  | 0.087 |  | -2.179 |  | 0.029 |  | -0.358 |  | -0.009 |  | -0.189 |  | -0.095 |  | -0.095 |  |  |  |
| RESIL |  | ~~ |  | RESIL |  |  |  | 44.757 |  | 3.616 |  | 12.378 |  | < .001 |  | 36.568 |  | 50.771 |  | 44.757 |  | 0.358 |  | 0.358 |  |  |  |
| SE |  | ~~ |  | SE |  |  |  | 10.820 |  | 0.799 |  | 13.546 |  | < .001 |  | 9.253 |  | 12.352 |  | 10.820 |  | 0.781 |  | 0.781 |  |  |  |
| LOT |  | ~~ |  | LOT |  |  |  | 18.466 |  | 1.521 |  | 12.140 |  | < .001 |  | 15.449 |  | 21.345 |  | 18.466 |  | 0.773 |  | 0.773 |  |  |  |
| SC |  | ~~ |  | SC |  |  |  | 56.113 |  | 4.607 |  | 12.179 |  | < .001 |  | 47.463 |  | 64.934 |  | 56.113 |  | 0.714 |  | 0.714 |  |  |  |
| STS |  | ~~ |  | STS |  |  |  | 16.024 |  | 1.461 |  | 10.964 |  | < .001 |  | 13.317 |  | 18.960 |  | 16.024 |  | 0.798 |  | 0.798 |  |  |  |
| BU |  | ~~ |  | BU |  |  |  | 17.805 |  | 1.502 |  | 11.857 |  | < .001 |  | 14.543 |  | 20.687 |  | 17.805 |  | 0.755 |  | 0.755 |  |  |  |
| CS |  | ~~ |  | CS |  |  |  | 22.079 |  | 2.023 |  | 10.914 |  | < .001 |  | 18.243 |  | 26.324 |  | 22.079 |  | 0.874 |  | 0.874 |  |  |  |
| PSS |  | ~~ |  | PSS |  |  |  | 36.253 |  | 0.000 |  |  |  |  |  | 36.253 |  | 36.253 |  | 36.253 |  | 1.000 |  | 36.253 |  |  |  |
| PSS |  | ~~ |  | RSQ |  |  |  | 15.112 |  | 0.000 |  |  |  |  |  | 15.112 |  | 15.112 |  | 15.112 |  | 0.344 |  | 15.112 |  |  |  |
| RSQ |  | ~~ |  | RSQ |  |  |  | 53.229 |  | 0.000 |  |  |  |  |  | 53.229 |  | 53.229 |  | 53.229 |  | 1.000 |  | 53.229 |  |  |  |
| ind\_x1\_m1\_y1 |  | := |  | a11\*b11 |  | ind\_x1\_m1\_y1 |  | -0.316 |  | 0.055 |  | -5.733 |  | < .001 |  | -0.437 |  | -0.209 |  | -0.316 |  | -0.170 |  | -0.028 |  |  |  |
| ind\_x1\_m2\_y1 |  | := |  | a21\*b12 |  | ind\_x1\_m2\_y1 |  | -0.112 |  | 0.041 |  | -2.751 |  | 0.006 |  | -0.200 |  | -0.039 |  | -0.112 |  | -0.060 |  | -0.010 |  |  |  |
| ind\_x1\_m3\_y1 |  | := |  | a31\*b13 |  | ind\_x1\_m3\_y1 |  | -0.179 |  | 0.048 |  | -3.719 |  | < .001 |  | -0.279 |  | -0.086 |  | -0.179 |  | -0.097 |  | -0.016 |  |  |  |
| ind\_x1\_m4\_y1 |  | := |  | a41\*b14 |  | ind\_x1\_m4\_y1 |  | 0.062 |  | 0.026 |  | 2.348 |  | 0.019 |  | 0.013 |  | 0.118 |  | 0.062 |  | 0.033 |  | 0.006 |  |  |  |
| ind\_x1\_m5\_y1 |  | := |  | a51\*b15 |  | ind\_x1\_m5\_y1 |  | -0.164 |  | 0.046 |  | -3.579 |  | < .001 |  | -0.269 |  | -0.087 |  | -0.164 |  | -0.089 |  | -0.015 |  |  |  |
| ind\_x1\_m6\_y1 |  | := |  | a61\*b16 |  | ind\_x1\_m6\_y1 |  | -0.104 |  | 0.035 |  | -2.932 |  | 0.003 |  | -0.182 |  | -0.041 |  | -0.104 |  | -0.056 |  | -0.009 |  |  |  |
| ind\_x1\_y1 |  | := |  | ind\_x1\_m1\_y1+ind\_x1\_m2\_y1+ind\_x1\_m3\_y1+ind\_x1\_m4\_y1+ind\_x1\_m5\_y1+ind\_x1\_m6\_y1 |  | ind\_x1\_y1 |  | -0.814 |  | 0.100 |  | -8.122 |  | < .001 |  | -1.021 |  | -0.622 |  | -0.814 |  | -0.438 |  | -0.073 |  |  |  |
| tot\_x1\_y1 |  | := |  | ind\_x1\_y1+c11 |  | tot\_x1\_y1 |  | -0.830 |  | 0.111 |  | -7.497 |  | < .001 |  | -1.052 |  | -0.615 |  | -0.830 |  | -0.447 |  | -0.074 |  |  |  |
| ind\_x2\_m2\_y1 |  | := |  | a22\*b12 |  | ind\_x2\_m2\_y1 |  | -0.030 |  | 0.014 |  | -2.232 |  | 0.026 |  | -0.057 |  | -0.007 |  | -0.030 |  | -0.020 |  | -0.003 |  |  |  |
| ind\_x2\_m4\_y1 |  | := |  | a42\*b14 |  | ind\_x2\_m4\_y1 |  | 0.031 |  | 0.016 |  | 1.873 |  | 0.061 |  | 0.005 |  | 0.067 |  | 0.031 |  | 0.020 |  | 0.003 |  |  |  |
| ind\_x2\_m5\_y1 |  | := |  | a52\*b15 |  | ind\_x2\_m5\_y1 |  | -0.068 |  | 0.022 |  | -3.038 |  | 0.002 |  | -0.117 |  | -0.030 |  | -0.068 |  | -0.044 |  | -0.006 |  |  |  |
| ind\_x2\_y1 |  | := |  | ind\_x2\_m2\_y1+ind\_x2\_m4\_y1+ind\_x2\_m5\_y1 |  | ind\_x2\_y1 |  | -0.067 |  | 0.025 |  | -2.665 |  | 0.008 |  | -0.117 |  | -0.022 |  | -0.067 |  | -0.044 |  | -0.006 |  |  |  |
| tot\_x2\_y1 |  | := |  | ind\_x2\_y1+c12 |  | tot\_x2\_y1 |  | -0.027 |  | 0.057 |  | -0.480 |  | 0.631 |  | -0.138 |  | 0.085 |  | -0.027 |  | -0.018 |  | -0.002 |  |  |  |
|  | | | | | | | | | | | | | | | | | | | | | | | | | | | |

| Model test baseline model | | | |
| --- | --- | --- | --- |
|  | | Model | |
| Minimum Function Test Statistic |  | 0.014 |  |
| χ² |  | 8.884 |  |
| Degrees of freedom |  | 5.000 |  |
| p |  | 0.114 |  |
|  | | | |

A good model–data fit is indicated by RMSEA < .06, CFI > .95, and TLI > .95 (Hu & Bentler, 1999)

| User model versus baseline model | | | |
| --- | --- | --- | --- |
|  | | Model | |
| Comparative Fit Index (CFI) |  | 0.997 |  |
| Tucker-Lewis Index (TLI) |  | 0.977 |  |
| Bentler-Bonett Non-normed Fit Index (NNFI) |  | 0.977 |  |
| Bentler-Bonett Normed Fit Index (NFI) |  | 0.993 |  |
| Parsimony Normed Fit Index (PNFI) |  | 0.142 |  |
| Bollen's Relative Fit Index (RFI) |  | 0.949 |  |
| Bollen's Incremental Fit Index (IFI) |  | 0.997 |  |
| Relative Noncentrality Index (RNI) |  | 0.997 |  |
|  | | | |

| Loglikelihood and Information Criteria | | | |
| --- | --- | --- | --- |
|  | | Model | |
| Loglikelihood user model (H0) |  | -6587.116 |  |
| Loglikelihood unrestricted model (H1) |  | -6582.674 |  |
| Number of free parameters |  | 37 |  |
| Akaike (AIC) |  | 13248.232 |  |
| Bayesian (BIC) |  | 13388.234 |  |
| Sample-size adjusted Bayesian (BIC) |  | 13270.873 |  |
|  | | | |

| Root Mean Square Error of Approximation | | | |
| --- | --- | --- | --- |
|  | | Model | |
| RMSEA |  | 0.049 |  |
| Upper 90% CI |  | 0.100 |  |
| Lower 90% CI |  | 0.000 |  |
| p-value RMSEA <= 0.05 |  | 0.445 |  |
|  | | | |

| Standardized Root Mean Square Residual | | | |
| --- | --- | --- | --- |
|  | | Model | |
| RMR |  | 1.563 |  |
| RMR (No Mean) |  | 1.563 |  |
| SRMR |  | 0.031 |  |
|  | | | |

| Other Fit Indices | | | |
| --- | --- | --- | --- |
|  | | Model | |
| Hoelter Critical N (CN) alpha=0.05 |  | 406.007 |  |
| Hoelter Critical N (CN) alpha=0.01 |  | 552.922 |  |
| Goodness of Fit Index (GFI) |  | 0.993 |  |
| Parsimony Goodness of Fit Index (GFI) |  | 0.936 |  |
| McDonald Fit Index (MFI) |  | 0.994 |  |
|  | | | |

| R-Squared | | | |
| --- | --- | --- | --- |
| Variable | | R² | |
| RESIL |  | 0.642 |  |
| SE |  | 0.219 |  |
| LOT |  | 0.227 |  |
| SC |  | 0.286 |  |
| STS |  | 0.202 |  |
| BU |  | 0.245 |  |
| CS |  | 0.126 |  |
|  | | | |

Individual R2: indicate that the covariates involved this proportion of variation in the mediator

| Covariances (lower triangle) / correlations (upper triangle) | | | | | | | | | | | | | | | | | | | | | |
| --- | --- | --- | --- | --- | --- | --- | --- | --- | --- | --- | --- | --- | --- | --- | --- | --- | --- | --- | --- | --- | --- |
|  | |  | | RESIL | | SE | | LOT | | SC | | STS | | BU | | CS | | PSS | | RSQ | |
| RESIL |  | observed |  | 125.379 |  | 0.657 |  | 0.563 |  | 0.596 |  | -0.239 |  | -0.621 |  | 0.565 |  | -0.460 |  | -0.264 |  |
|  |  | fitted |  | 124.888 |  | 0.655 |  | 0.555 |  | 0.594 |  | -0.220 |  | -0.610 |  | 0.565 |  | -0.461 |  | -0.178 |  |
|  |  | residual |  | 0.491 |  | 0.002 |  | 0.008 |  | 0.002 |  | -0.019 |  | -0.011 |  | 8.770e -5 |  | 9.033e -4 |  | -0.086 |  |
| SE |  | observed |  | 27.373 |  | 13.854 |  | 0.468 |  | 0.469 |  | -0.321 |  | -0.451 |  | 0.408 |  | -0.467 |  | -0.282 |  |
|  |  | fitted |  | 27.248 |  | 13.847 |  | 0.454 |  | 0.468 |  | -0.296 |  | -0.431 |  | 0.407 |  | -0.468 |  | -0.161 |  |
|  |  | residual |  | 0.125 |  | 0.007 |  | 0.014 |  | 0.001 |  | -0.026 |  | -0.020 |  | 6.649e -4 |  | 1.171e -4 |  | -0.121 |  |
| LOT |  | observed |  | 31.026 |  | 8.570 |  | 24.238 |  | 0.547 |  | -0.230 |  | -0.494 |  | 0.334 |  | -0.455 |  | -0.331 |  |
|  |  | fitted |  | 30.302 |  | 8.252 |  | 23.874 |  | 0.538 |  | -0.211 |  | -0.483 |  | 0.331 |  | -0.459 |  | -0.277 |  |
|  |  | residual |  | 0.723 |  | 0.318 |  | 0.364 |  | 0.009 |  | -0.019 |  | -0.011 |  | 0.004 |  | 0.003 |  | -0.054 |  |
| SC |  | observed |  | 59.218 |  | 15.491 |  | 23.924 |  | 78.811 |  | -0.305 |  | -0.528 |  | 0.383 |  | -0.527 |  | -0.288 |  |
|  |  | fitted |  | 58.859 |  | 15.435 |  | 23.319 |  | 78.633 |  | -0.294 |  | -0.517 |  | 0.382 |  | -0.528 |  | -0.199 |  |
|  |  | residual |  | 0.359 |  | 0.056 |  | 0.605 |  | 0.178 |  | -0.011 |  | -0.011 |  | 0.001 |  | 5.978e -4 |  | -0.089 |  |
| STS |  | observed |  | -12.016 |  | -5.375 |  | -5.092 |  | -12.185 |  | 20.200 |  | 0.468 |  | -0.159 |  | 0.406 |  | 0.333 |  |
|  |  | fitted |  | -11.001 |  | -4.932 |  | -4.620 |  | -11.702 |  | 20.085 |  | 0.459 |  | -0.144 |  | 0.407 |  | 0.320 |  |
|  |  | residual |  | -1.015 |  | -0.443 |  | -0.472 |  | -0.483 |  | 0.115 |  | 0.010 |  | -0.015 |  | -0.001 |  | 0.013 |  |
| BU |  | observed |  | -34.121 |  | -8.229 |  | -11.926 |  | -23.011 |  | 10.331 |  | 24.081 |  | -0.666 |  | 0.455 |  | 0.375 |  |
|  |  | fitted |  | -33.100 |  | -7.782 |  | -11.452 |  | -22.285 |  | 9.986 |  | 23.591 |  | -0.662 |  | 0.460 |  | 0.331 |  |
|  |  | residual |  | -1.021 |  | -0.447 |  | -0.474 |  | -0.726 |  | 0.345 |  | 0.490 |  | -0.004 |  | -0.005 |  | 0.044 |  |
| CS |  | observed |  | 31.798 |  | 7.629 |  | 8.272 |  | 17.094 |  | -3.590 |  | -16.417 |  | 25.254 |  | -0.355 |  | -0.168 |  |
|  |  | fitted |  | 31.731 |  | 7.614 |  | 8.120 |  | 17.030 |  | -3.249 |  | -16.157 |  | 25.254 |  | -0.355 |  | -0.122 |  |
|  |  | residual |  | 0.067 |  | 0.014 |  | 0.152 |  | 0.064 |  | -0.340 |  | -0.260 |  | 2.960e -5 |  | 2.155e -7 |  | -0.046 |  |
| PSS |  | observed |  | -31.025 |  | -10.476 |  | -13.494 |  | -28.192 |  | 10.980 |  | 13.449 |  | -10.729 |  | 36.253 |  | 0.344 |  |
|  |  | fitted |  | -31.025 |  | -10.476 |  | -13.494 |  | -28.192 |  | 10.980 |  | 13.449 |  | -10.729 |  | 36.253 |  | 0.344 |  |
|  |  | residual |  | 6.155e -7 |  | 1.550e -7 |  | 1.796e -7 |  | -1.649e -7 |  | 7.254e -9 |  | -1.691e -7 |  | 2.327e -7 |  | 0.000 |  | 0.000 |  |
| RSQ |  | observed |  | -21.562 |  | -7.657 |  | -11.885 |  | -18.641 |  | 10.911 |  | 13.426 |  | -6.147 |  | 15.112 |  | 53.229 |  |
|  |  | fitted |  | -14.494 |  | -4.367 |  | -9.879 |  | -12.861 |  | 10.452 |  | 11.721 |  | -4.472 |  | 15.112 |  | 53.229 |  |
|  |  | residual |  | -7.068 |  | -3.290 |  | -2.005 |  | -5.780 |  | 0.460 |  | 1.705 |  | -1.675 |  | 0.000 |  | 0.000 |  |
|  | | | | | | | | | | | | | | | | | | | | | |

The goal of structural equation modeling here is to test whether our theoretically motivated model of the covariance among variables provides a good approximation of the data. More specifically, we are trying to test how well a parsimonious model (composed of measurement and/or structural components) reproduces the observed covariance matrix. Formally, we are seeking to develop a model whose *model-implied covariance matrix* approaches the *sample (observed) covariance matrix*.

```

```

The covarience matrix above provide evidence of a good fit.

We also computed residual covariance matrix (using R syntaxis) resid(NewModel9, "cor"):

$type

[1] "cor.bollen"

$cov

   RESIL SE   LOT  SC   STS  BU   CS   PSS  RSQ

RESIL 0.000

SE   0.001 0.000

LOT  0.008 0.013 0.000

SC   0.002 0.001 0.009 0.000

STS  -0.019 -0.026 -0.018 -0.011 0.000

BU  -0.011 -0.020 -0.010 -0.011 0.011 0.000

CS   0.000 0.001 0.003 0.001 -0.017 -0.004 0.000

PSS  0.001 0.000 0.003 0.001 -0.001 -0.005 0.000 0.000

RSQ  -0.083 -0.117 -0.053 -0.088 0.013 0.042 -0.043 0.000 0.000

The residual covariance matrix indicates that we do not have significantly unrpredicted (positive value >.1) or significantly overpredicted (negative value >.1).

| Modification Indices | | | | | | | | | | | | | | | |
| --- | --- | --- | --- | --- | --- | --- | --- | --- | --- | --- | --- | --- | --- | --- | --- |
|  | |  | |  | | mi | | epc | | sepc (lv) | | sepc (all) | | sepc (nox) | |
| SE |  | ~ |  | SC |  | 4.475 |  | 2.224 |  | 2.224 |  | 5.299 |  | 5.299 |  |
| SE |  | ~ |  | RESIL |  | 4.459 |  | 1.576 |  | 1.576 |  | 4.732 |  | 4.732 |  |
| SE |  | ~ |  | LOT |  | 4.448 |  | 0.578 |  | 0.578 |  | 0.759 |  | 0.759 |  |
| SE |  | ~ |  | BU |  | 4.447 |  | -0.402 |  | -0.402 |  | -0.525 |  | -0.525 |  |
| SE |  | ~ |  | RSQ |  | 4.446 |  | -0.052 |  | -0.052 |  | -0.103 |  | -0.014 |  |
| SE |  | ~ |  | STS |  | 4.446 |  | -0.418 |  | -0.418 |  | -0.504 |  | -0.504 |  |
| RSQ |  | ~ |  | SE |  | 4.049 |  | -0.178 |  | -0.178 |  | -0.091 |  | -0.091 |  |
| PSS |  | ~ |  | SE |  | 3.243 |  | 0.341 |  | 0.341 |  | 0.211 |  | 0.211 |  |
| RSQ |  | ~ |  | RESIL |  | 3.089 |  | -0.063 |  | -0.063 |  | -0.097 |  | -0.097 |  |
| PSS |  | ~ |  | RESIL |  | 2.463 |  | 0.121 |  | 0.121 |  | 0.224 |  | 0.224 |  |
| RSQ |  | ~ |  | LOT |  | 2.235 |  | -0.161 |  | -0.161 |  | -0.108 |  | -0.108 |  |
| RSQ |  | ~ |  | SC |  | 2.173 |  | -0.054 |  | -0.054 |  | -0.066 |  | -0.066 |  |
| PSS |  | ~ |  | LOT |  | 1.952 |  | 0.337 |  | 0.337 |  | 0.274 |  | 0.274 |  |
| PSS |  | ~ |  | SC |  | 1.873 |  | 0.112 |  | 0.112 |  | 0.165 |  | 0.165 |  |
| SC |  | ~ |  | RESIL |  | 1.768 |  | 3.488 |  | 3.488 |  | 4.396 |  | 4.396 |  |
| SC |  | ~~ |  | STS |  | 1.760 |  | 9.924 |  | 9.924 |  | 0.331 |  | 0.331 |  |
| SC |  | ~ |  | RSQ |  | 1.760 |  | -0.078 |  | -0.078 |  | -0.064 |  | -0.009 |  |
| SC |  | ~ |  | LOT |  | 1.760 |  | 0.855 |  | 0.855 |  | 0.471 |  | 0.471 |  |
| SC |  | ~ |  | BU |  | 1.760 |  | -0.861 |  | -0.861 |  | -0.471 |  | -0.471 |  |
| SC |  | ~ |  | SE |  | 1.748 |  | -5.622 |  | -5.622 |  | -2.359 |  | -2.359 |  |
| RSQ |  | ~ |  | BU |  | 1.202 |  | 0.102 |  | 0.102 |  | 0.068 |  | 0.068 |  |
| PSS |  | ~ |  | BU |  | 1.167 |  | -0.237 |  | -0.237 |  | -0.191 |  | -0.191 |  |
| RSQ |  | ~ |  | CS |  | 0.645 |  | -0.056 |  | -0.056 |  | -0.038 |  | -0.038 |  |
| PSS |  | ~ |  | CS |  | 0.556 |  | 0.115 |  | 0.115 |  | 0.096 |  | 0.096 |  |
| RSQ |  | ~ |  | STS |  | 0.512 |  | 0.161 |  | 0.161 |  | 0.099 |  | 0.099 |  |
| PSS |  | ~ |  | STS |  | 0.181 |  | -0.137 |  | -0.137 |  | -0.102 |  | -0.102 |  |
| STS |  | ~ |  | SC |  | 0.105 |  | 0.021 |  | 0.021 |  | 0.041 |  | 0.041 |  |
| LOT |  | ~~ |  | STS |  | 0.073 |  | -0.251 |  | -0.251 |  | -0.015 |  | -0.015 |  |
| LOT |  | ~ |  | STS |  | 0.073 |  | -0.016 |  | -0.016 |  | -0.014 |  | -0.014 |  |
| LOT |  | ~ |  | BU |  | 0.072 |  | -0.049 |  | -0.049 |  | -0.048 |  | -0.048 |  |
| LOT |  | ~ |  | SE |  | 0.072 |  | 0.142 |  | 0.142 |  | 0.109 |  | 0.109 |  |
| LOT |  | ~ |  | SC |  | 0.072 |  | 0.083 |  | 0.083 |  | 0.150 |  | 0.150 |  |
| LOT |  | ~ |  | RESIL |  | 0.072 |  | 0.177 |  | 0.177 |  | 0.404 |  | 0.404 |  |
| STS |  | ~ |  | LOT |  | 0.036 |  | -0.010 |  | -0.010 |  | -0.011 |  | -0.011 |  |
| CS |  | ~ |  | SE |  | 0.019 |  | 0.080 |  | 0.080 |  | 0.059 |  | 0.059 |  |
| STS |  | ~~ |  | CS |  | 0.019 |  | -0.141 |  | -0.141 |  | -0.008 |  | -0.008 |  |
| STS |  | ~ |  | CS |  | 0.016 |  | -0.006 |  | -0.006 |  | -0.007 |  | -0.007 |  |
| CS |  | ~ |  | SC |  | 0.015 |  | 0.040 |  | 0.040 |  | 0.071 |  | 0.071 |  |
| CS |  | ~ |  | STS |  | 0.015 |  | -0.008 |  | -0.008 |  | -0.007 |  | -0.007 |  |
| STS |  | ~ |  | BU |  | 0.014 |  | 0.010 |  | 0.010 |  | 0.010 |  | 0.010 |  |
| STS |  | ~ |  | SE |  | 0.012 |  | -0.019 |  | -0.019 |  | -0.016 |  | -0.016 |  |
| STS |  | ~ |  | RESIL |  | 0.008 |  | -0.003 |  | -0.003 |  | -0.008 |  | -0.008 |  |
| CS |  | ~ |  | RESIL |  | 0.007 |  | 0.050 |  | 0.050 |  | 0.111 |  | 0.111 |  |
| CS |  | ~ |  | BU |  | 0.006 |  | -0.013 |  | -0.013 |  | -0.012 |  | -0.012 |  |
| CS |  | ~ |  | LOT |  | 0.003 |  | -0.023 |  | -0.023 |  | -0.022 |  | -0.022 |  |
| CS |  | ~ |  | RSQ |  | 0.003 |  | 0.002 |  | 0.002 |  | 0.003 |  | 4.167e  -4 |  |
| RSQ |  | ~~ |  | RSQ |  | 3.713e -29 |  | 3.713e -29 |  | 0.000 |  | 0.000 |  | 0.000 |  |
| PSS |  | ~~ |  | RSQ |  | 4.499e -31 |  | 4.499e -31 |  | 4.499e -31 |  |  |  | 4.499e -31 |  |
| PSS |  | ~~ |  | PSS |  | 4.417e -31 |  | 4.417e -31 |  | 0.000 |  | 0.000 |  | 0.000 |  |
| PSS |  | ~ |  | RSQ |  | 9.459e -32 |  | -2.097e -17 |  | -2.097e -17 |  | -2.541e -17 |  | -3.483e -18 |  |
| RSQ |  | ~ |  | PSS |  | 1.300e -32 |  | 9.790e -18 |  | 9.790e -18 |  | 8.079e -18 |  | 1.342e -18 |  |
|  | | | | | | | | | | | | | | | |

The modification indices table above does not provide evidence that some of the paths should be freeing.

It has to be noted that instead of using the default 'delta method' for testing mediation (which is known to be problematic because the sampling distribution of the inderect path product term is not normal), we used bootstrapping. Bootstrapping is a common workaround for the debate in the literature about estimates for indirect paths as it does not make strong assumptions about the distribution of the coefficient of interst (i.e., the sampling distributions of the mediated paths). As all indirect paths are significant, we can conclude that these is evidence for mediation.
