# Supplementary figures and images for: Individual factors in the relationship between stress and resilience in mental health psychology practitioners during the COVID-19 pandemic
Source: J Health Psychol. 2021 Dec 7;27(11):2613–31. doi: 10.1177/13591053211059393 (PMC9483698; doi:10.1177/13591053211059393)

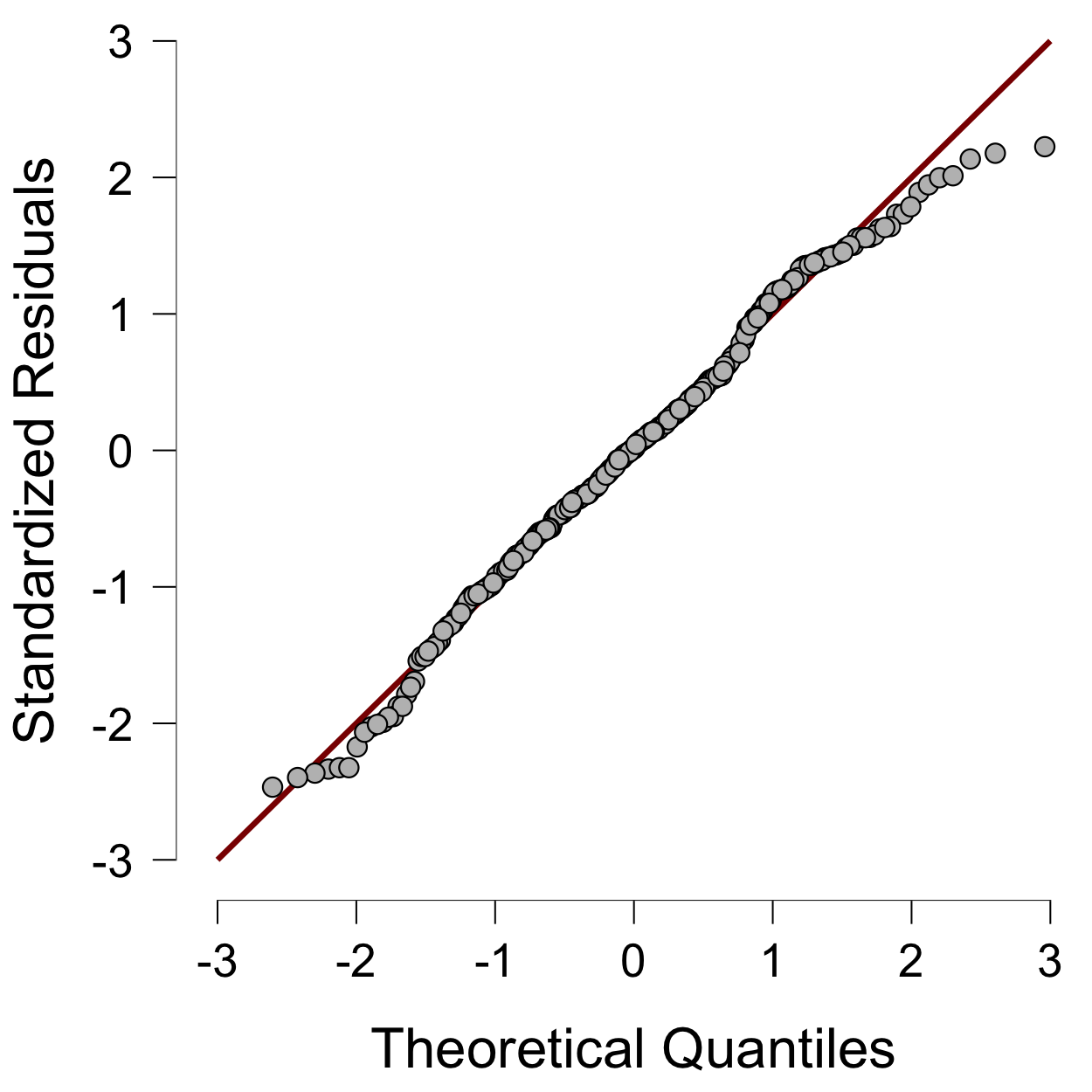

Supplement: sj-jasp-3-hpq-10.1177_13591053211059393 – Supplemental material for Individual factors in the relationship between stress and resilience in mental health psychology practitioners during the COVID-19 pandemic [file sj-jasp-3-hpq-10.1177_13591053211059393.jasp › resources/6/_111_t1602546316217.png]

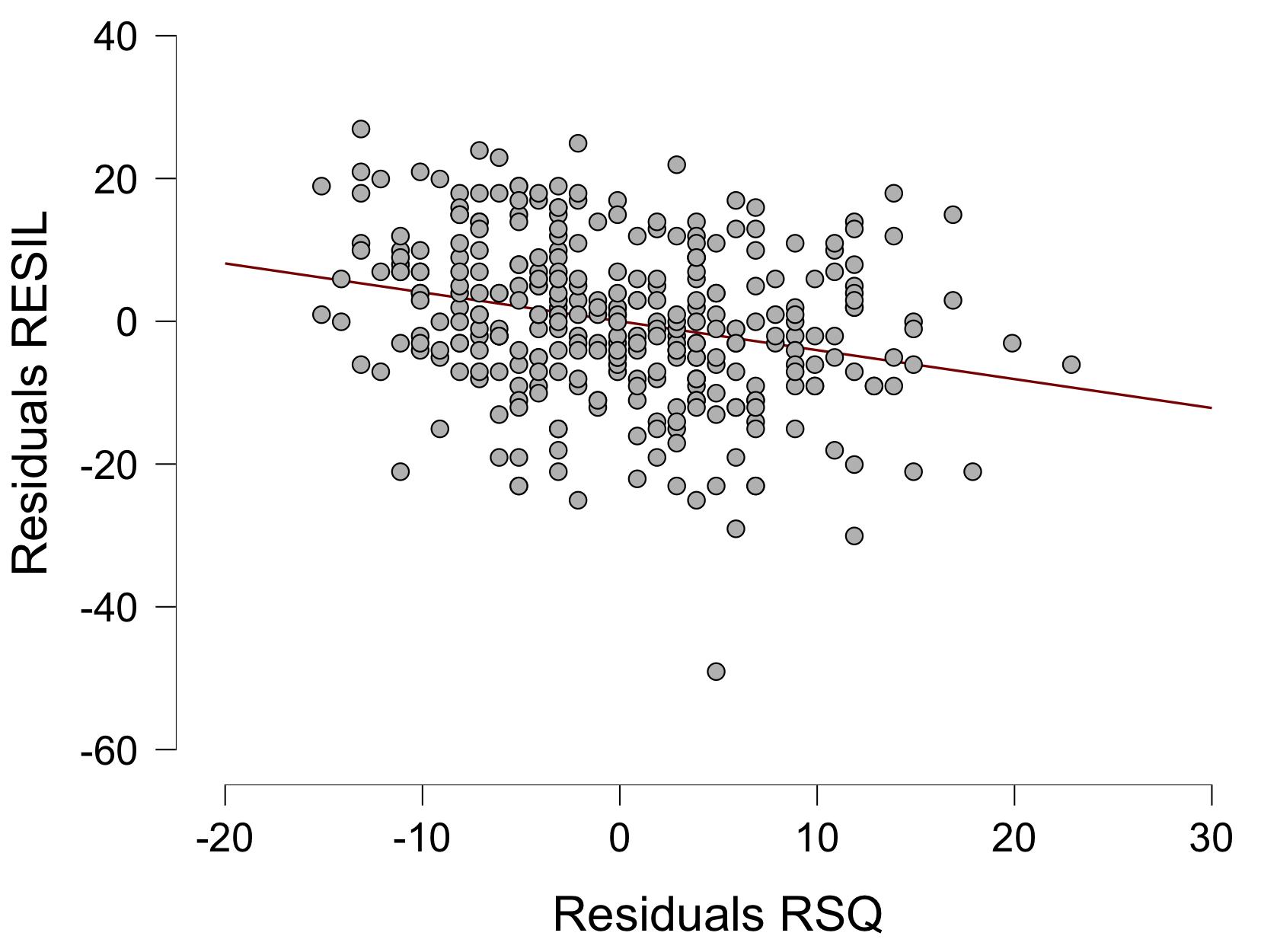

Supplement: sj-jasp-3-hpq-10.1177_13591053211059393 – Supplemental material for Individual factors in the relationship between stress and resilience in mental health psychology practitioners during the COVID-19 pandemic [file sj-jasp-3-hpq-10.1177_13591053211059393.jasp › resources/6/_112_t1602546316406.png]

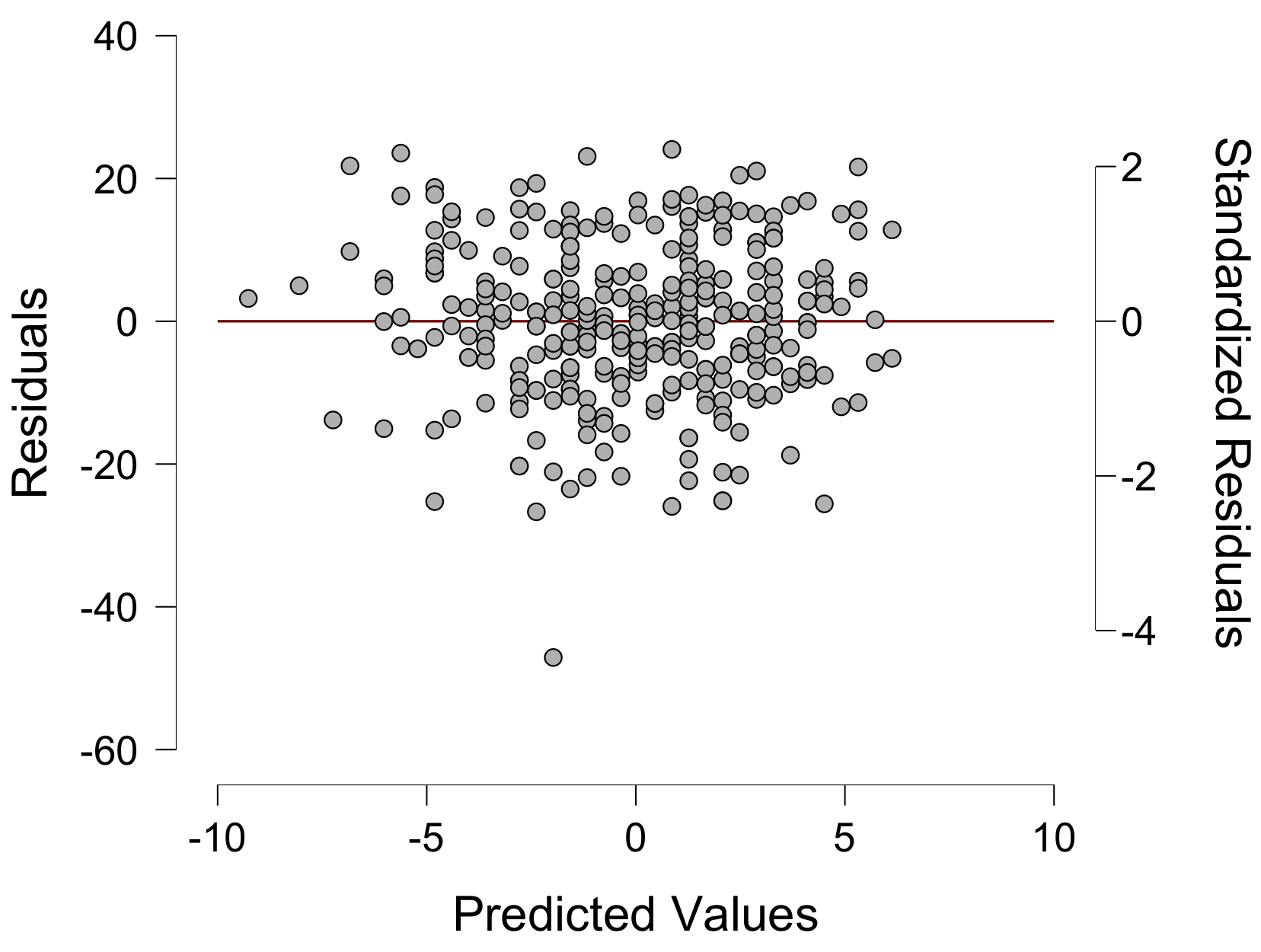

Supplement: sj-jasp-3-hpq-10.1177_13591053211059393 – Supplemental material for Individual factors in the relationship between stress and resilience in mental health psychology practitioners during the COVID-19 pandemic [file sj-jasp-3-hpq-10.1177_13591053211059393.jasp › resources/6/_109_t1602546315753.png]

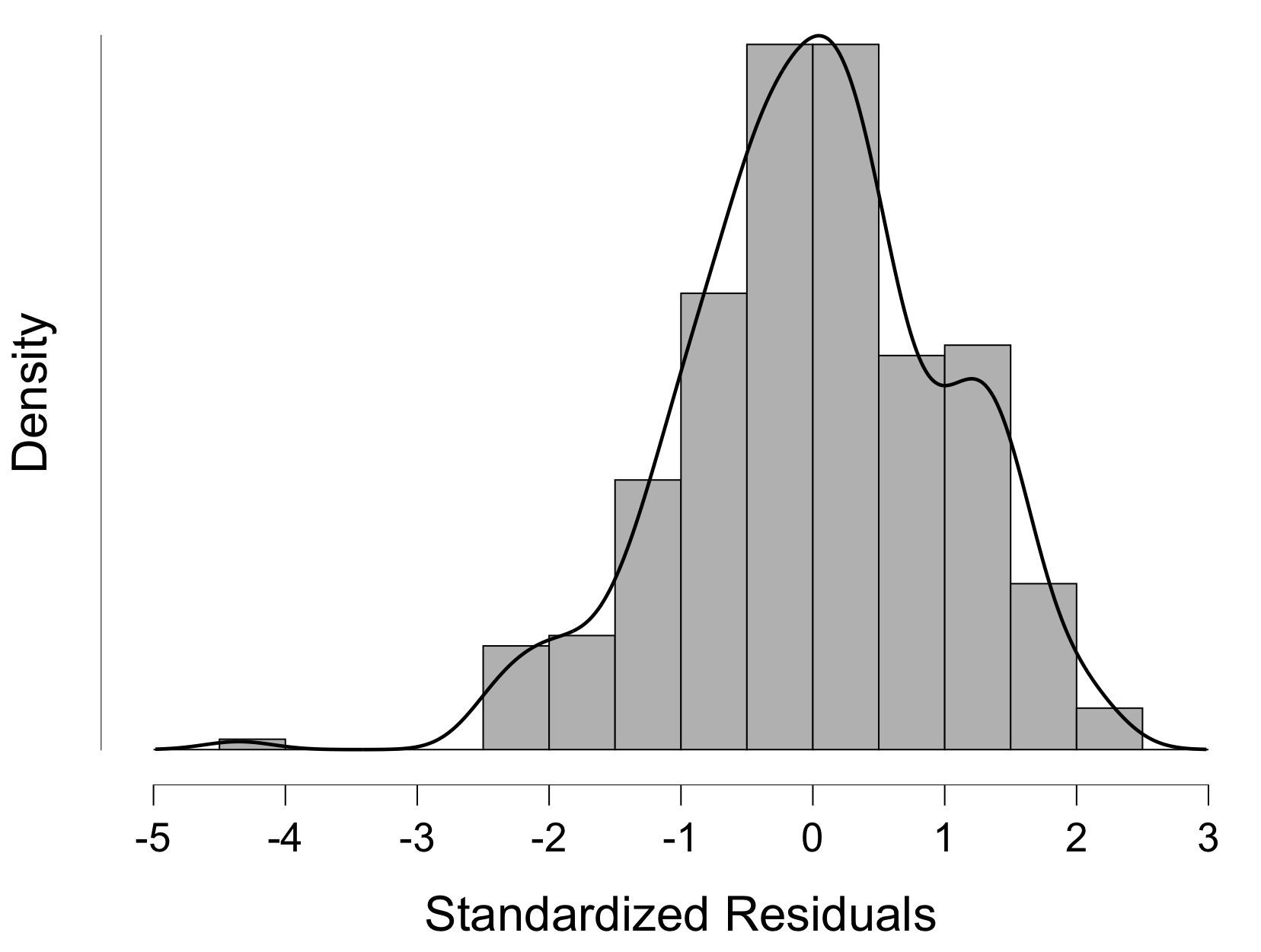

Supplement: sj-jasp-3-hpq-10.1177_13591053211059393 – Supplemental material for Individual factors in the relationship between stress and resilience in mental health psychology practitioners during the COVID-19 pandemic [file sj-jasp-3-hpq-10.1177_13591053211059393.jasp › resources/6/_110_t1602546315977.png]

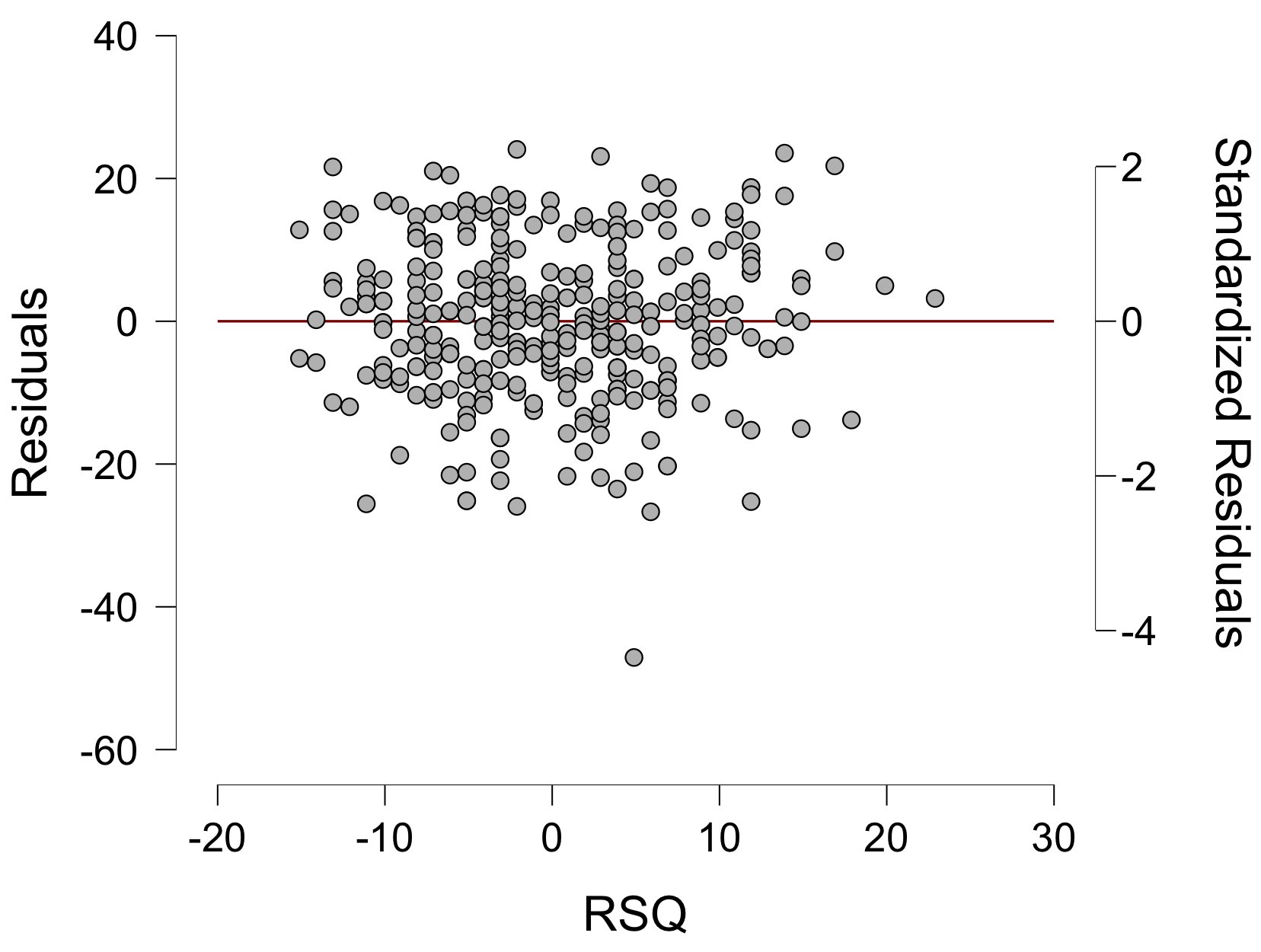

Supplement: sj-jasp-3-hpq-10.1177_13591053211059393 – Supplemental material for Individual factors in the relationship between stress and resilience in mental health psychology practitioners during the COVID-19 pandemic [file sj-jasp-3-hpq-10.1177_13591053211059393.jasp › resources/6/_108_t1602546315469.png]

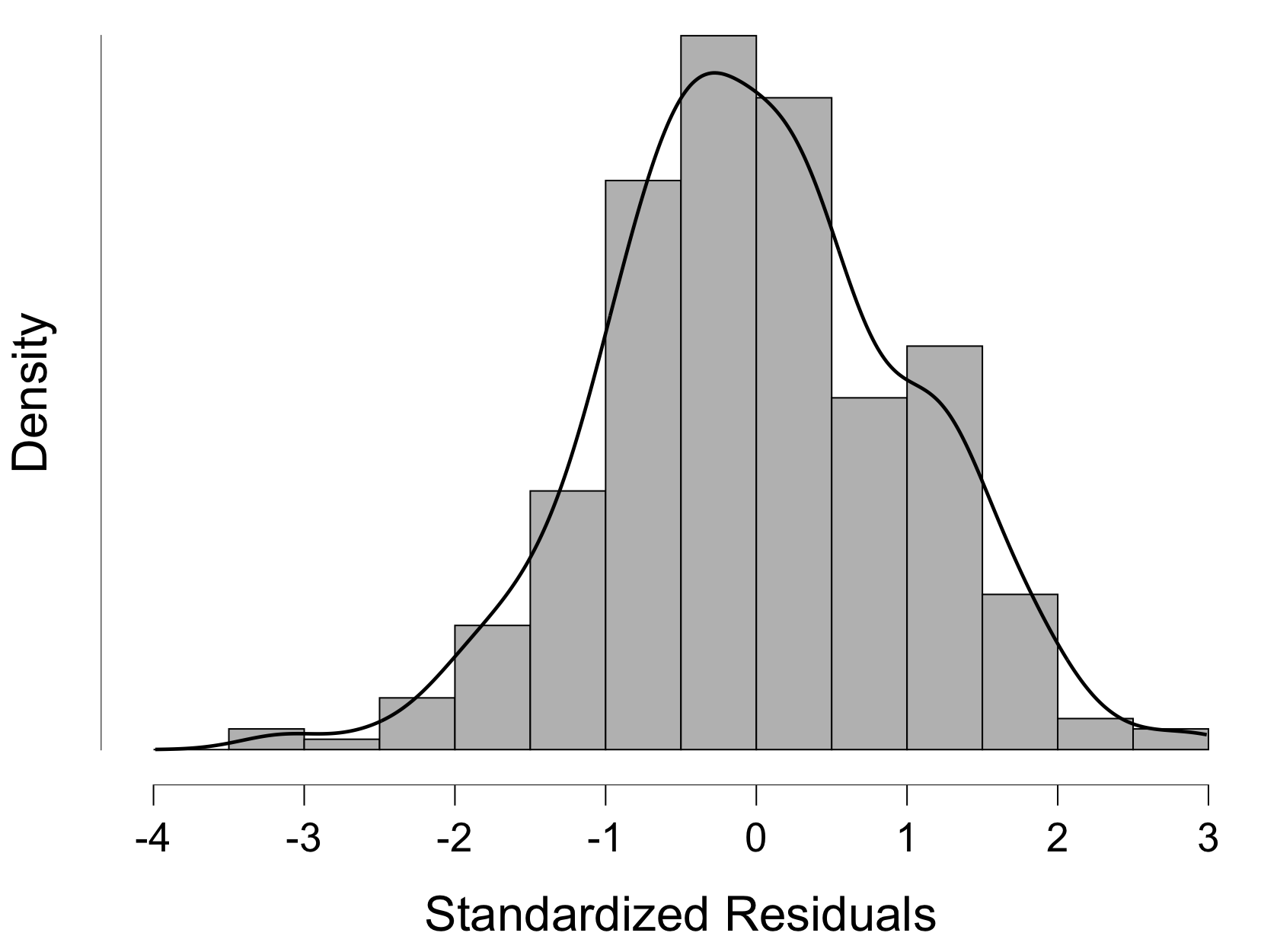

Supplement: sj-jasp-3-hpq-10.1177_13591053211059393 – Supplemental material for Individual factors in the relationship between stress and resilience in mental health psychology practitioners during the COVID-19 pandemic [file sj-jasp-3-hpq-10.1177_13591053211059393.jasp › resources/10/_105_t1602546153159.png]

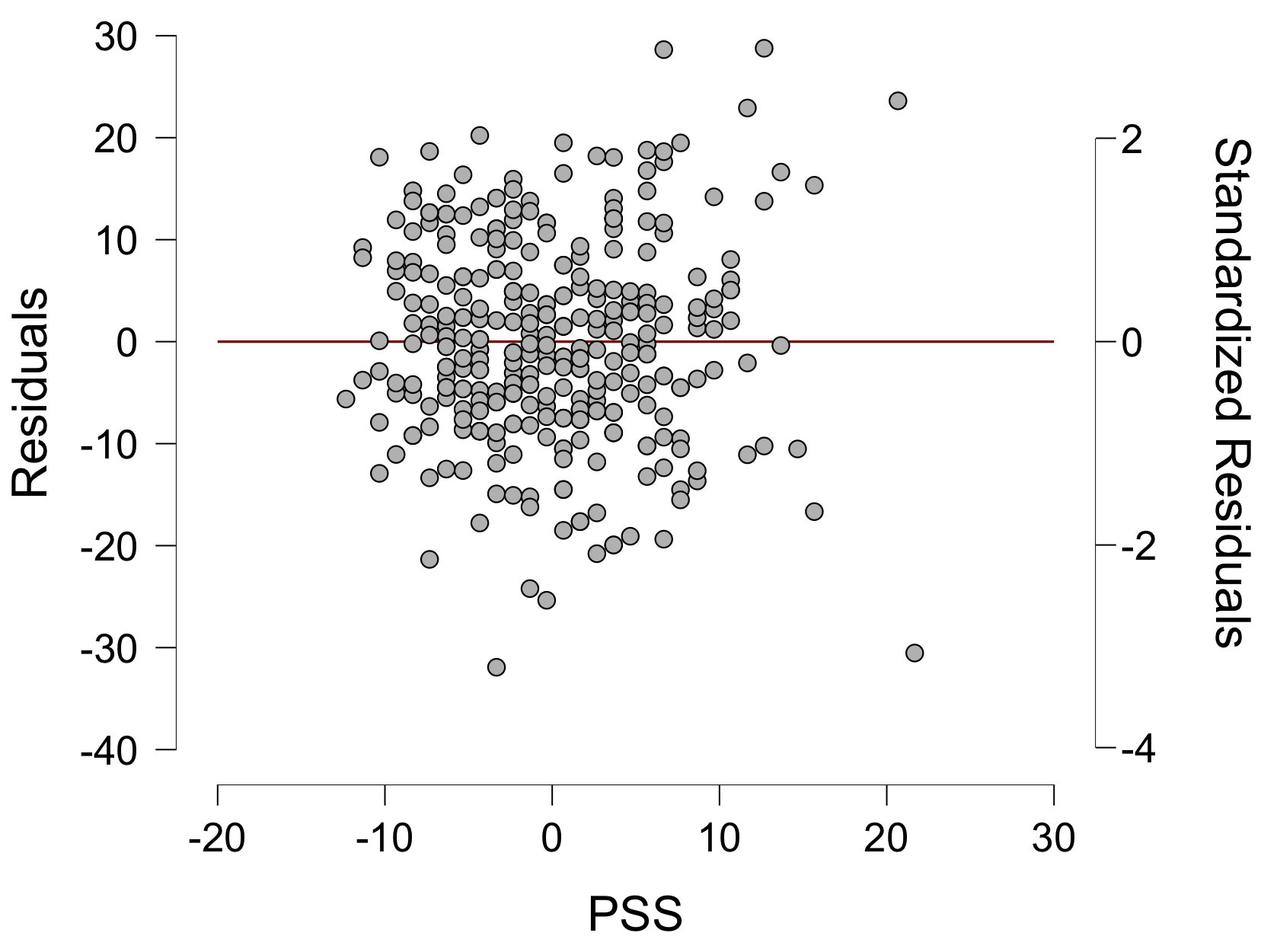

Supplement: sj-jasp-3-hpq-10.1177_13591053211059393 – Supplemental material for Individual factors in the relationship between stress and resilience in mental health psychology practitioners during the COVID-19 pandemic [file sj-jasp-3-hpq-10.1177_13591053211059393.jasp › resources/10/_103_t1602546151314.png]

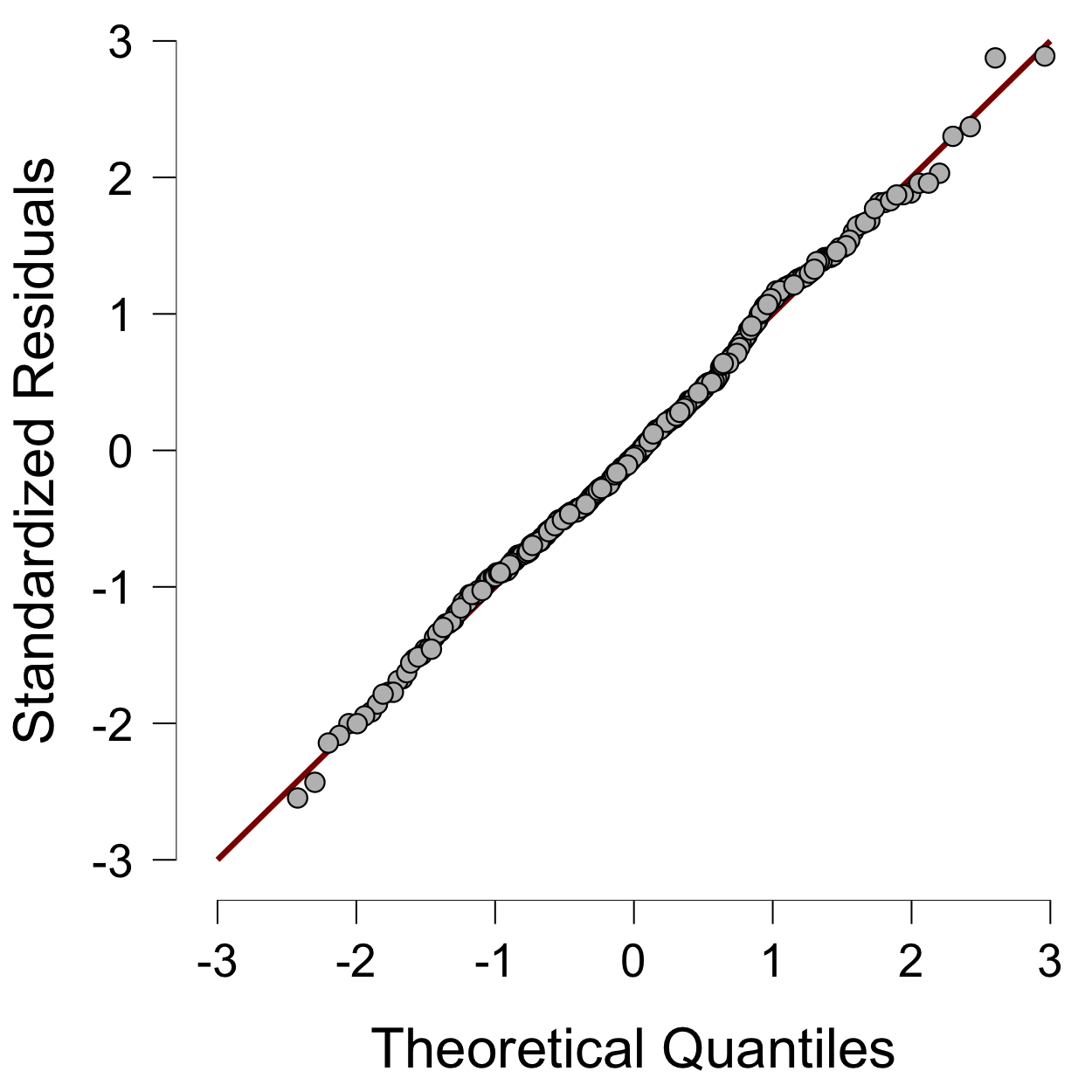

Supplement: sj-jasp-3-hpq-10.1177_13591053211059393 – Supplemental material for Individual factors in the relationship between stress and resilience in mental health psychology practitioners during the COVID-19 pandemic [file sj-jasp-3-hpq-10.1177_13591053211059393.jasp › resources/10/_106_t1602546154839.png]

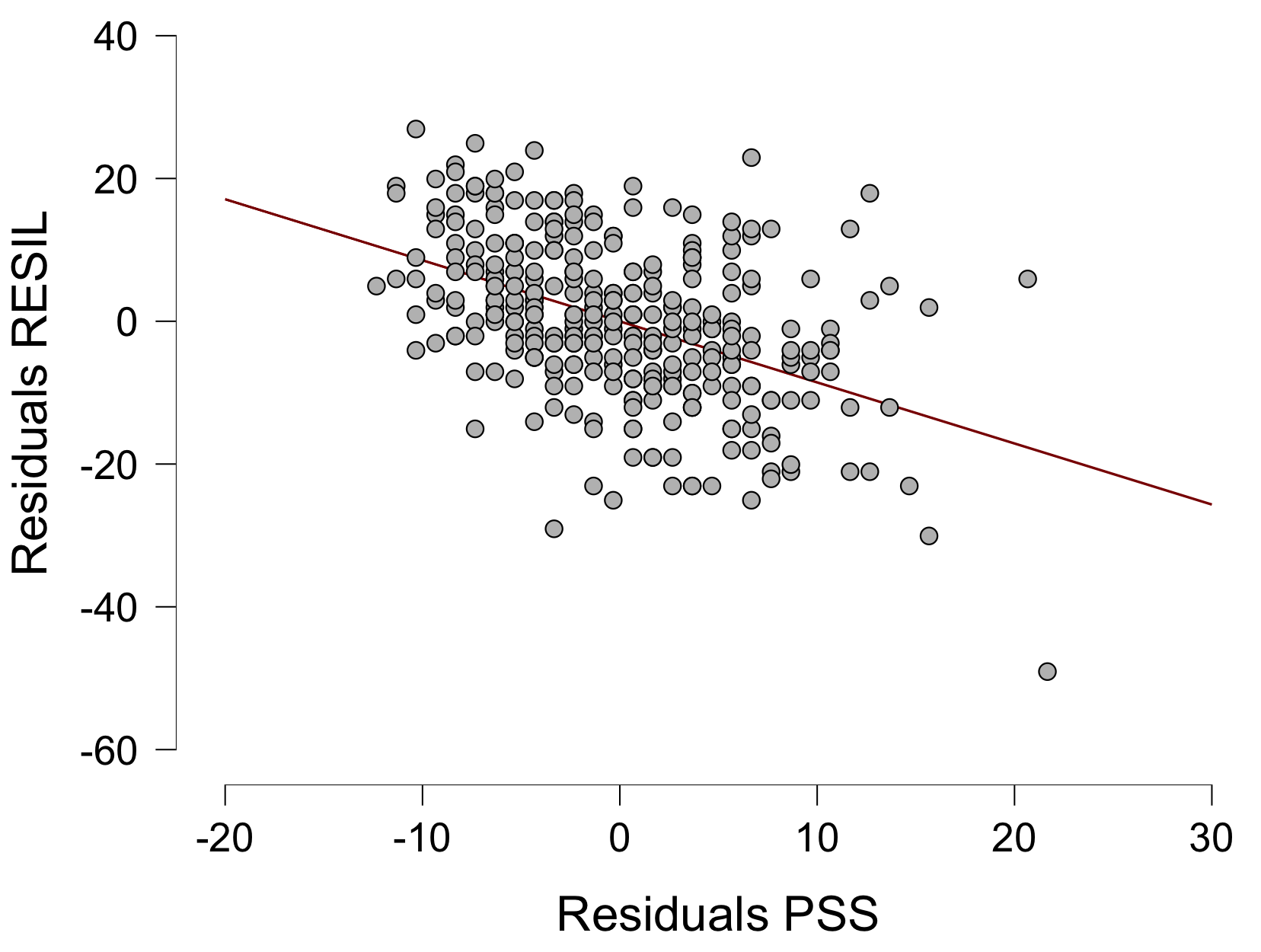

Supplement: sj-jasp-3-hpq-10.1177_13591053211059393 – Supplemental material for Individual factors in the relationship between stress and resilience in mental health psychology practitioners during the COVID-19 pandemic [file sj-jasp-3-hpq-10.1177_13591053211059393.jasp › resources/10/_107_t1602546155849.png]

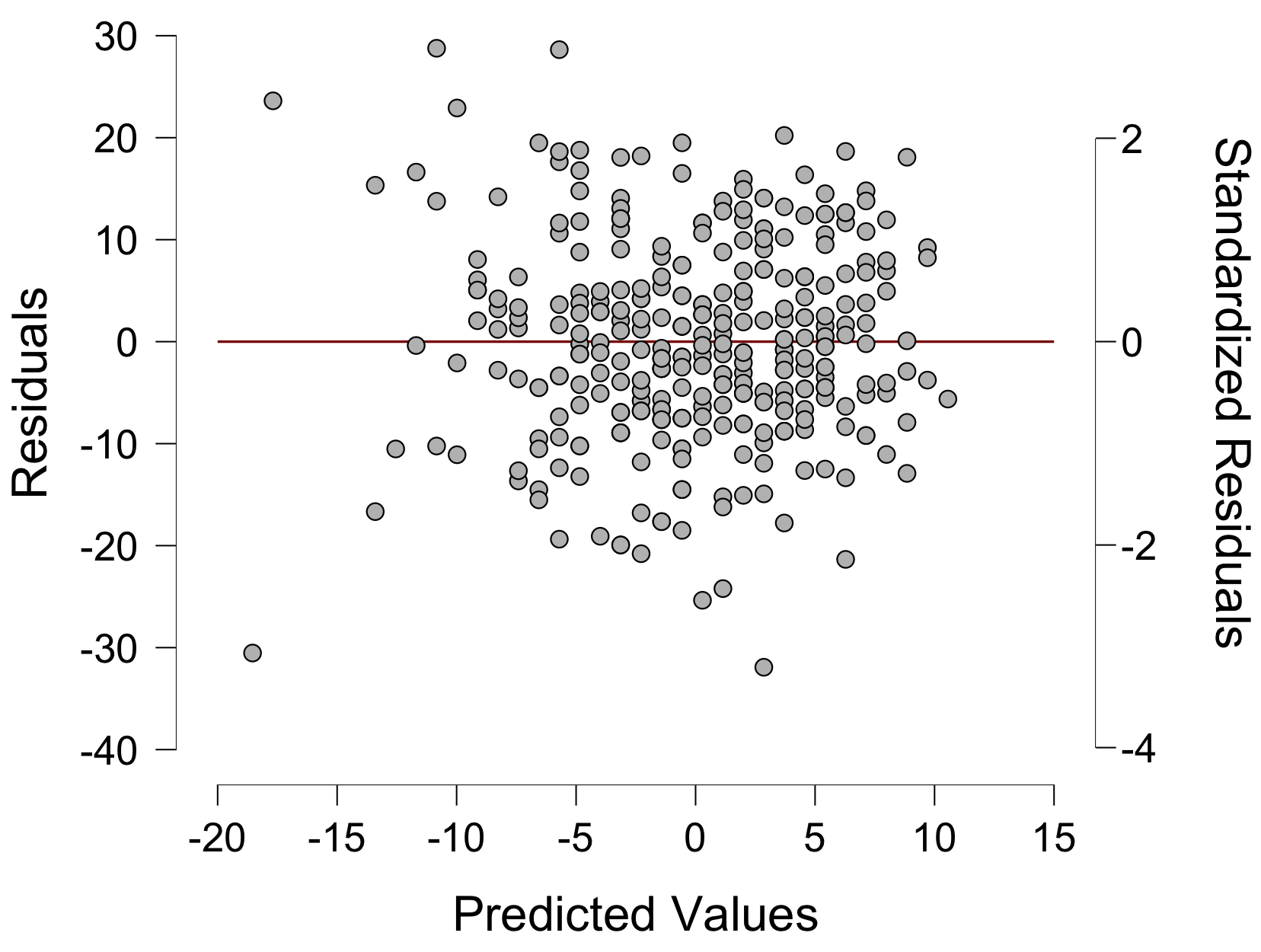

Supplement: sj-jasp-3-hpq-10.1177_13591053211059393 – Supplemental material for Individual factors in the relationship between stress and resilience in mental health psychology practitioners during the COVID-19 pandemic [file sj-jasp-3-hpq-10.1177_13591053211059393.jasp › resources/10/_104_t1602546152254.png]

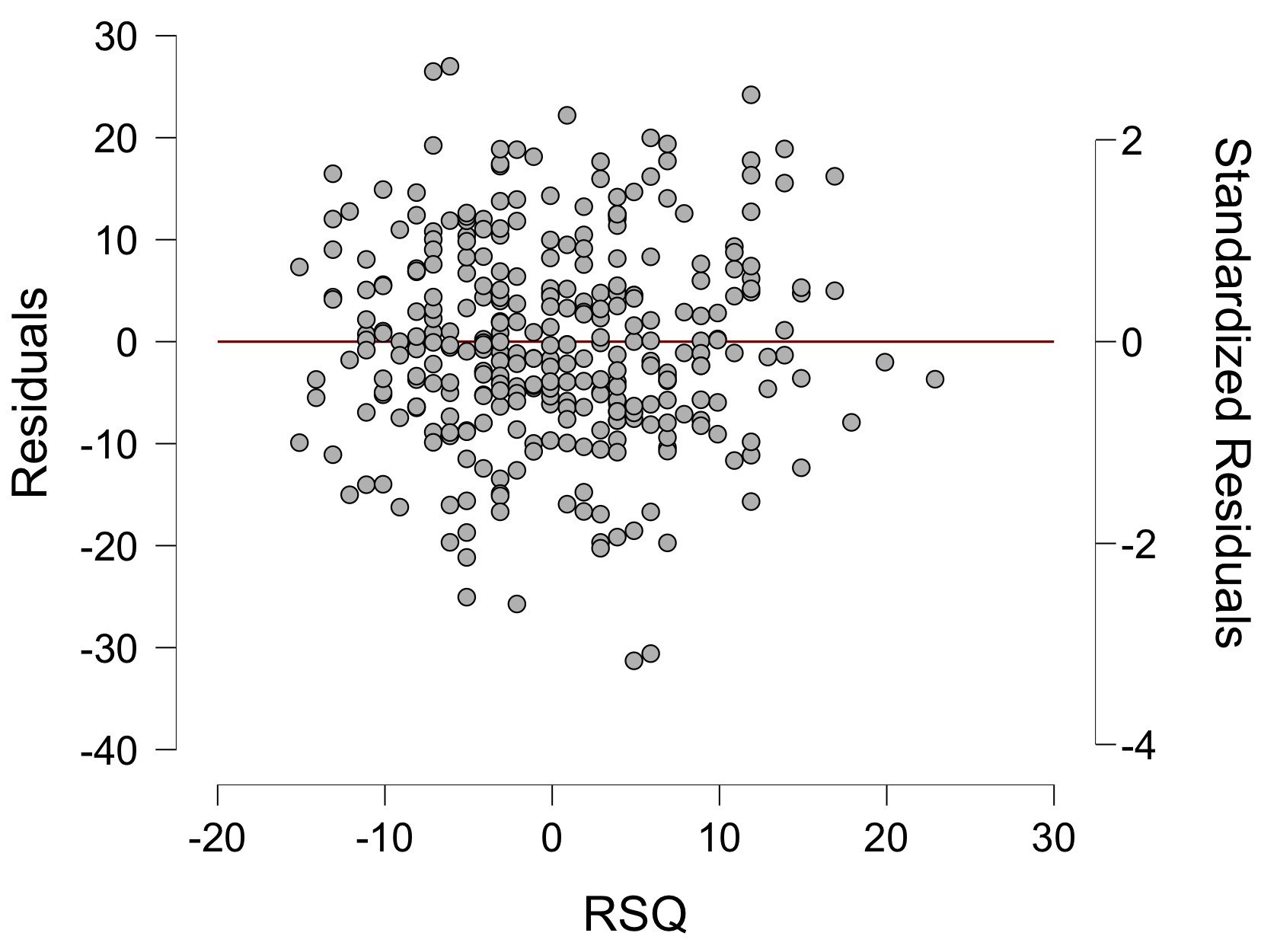

Supplement: sj-jasp-3-hpq-10.1177_13591053211059393 – Supplemental material for Individual factors in the relationship between stress and resilience in mental health psychology practitioners during the COVID-19 pandemic [file sj-jasp-3-hpq-10.1177_13591053211059393.jasp › resources/11/_113_t1602546809571.png]

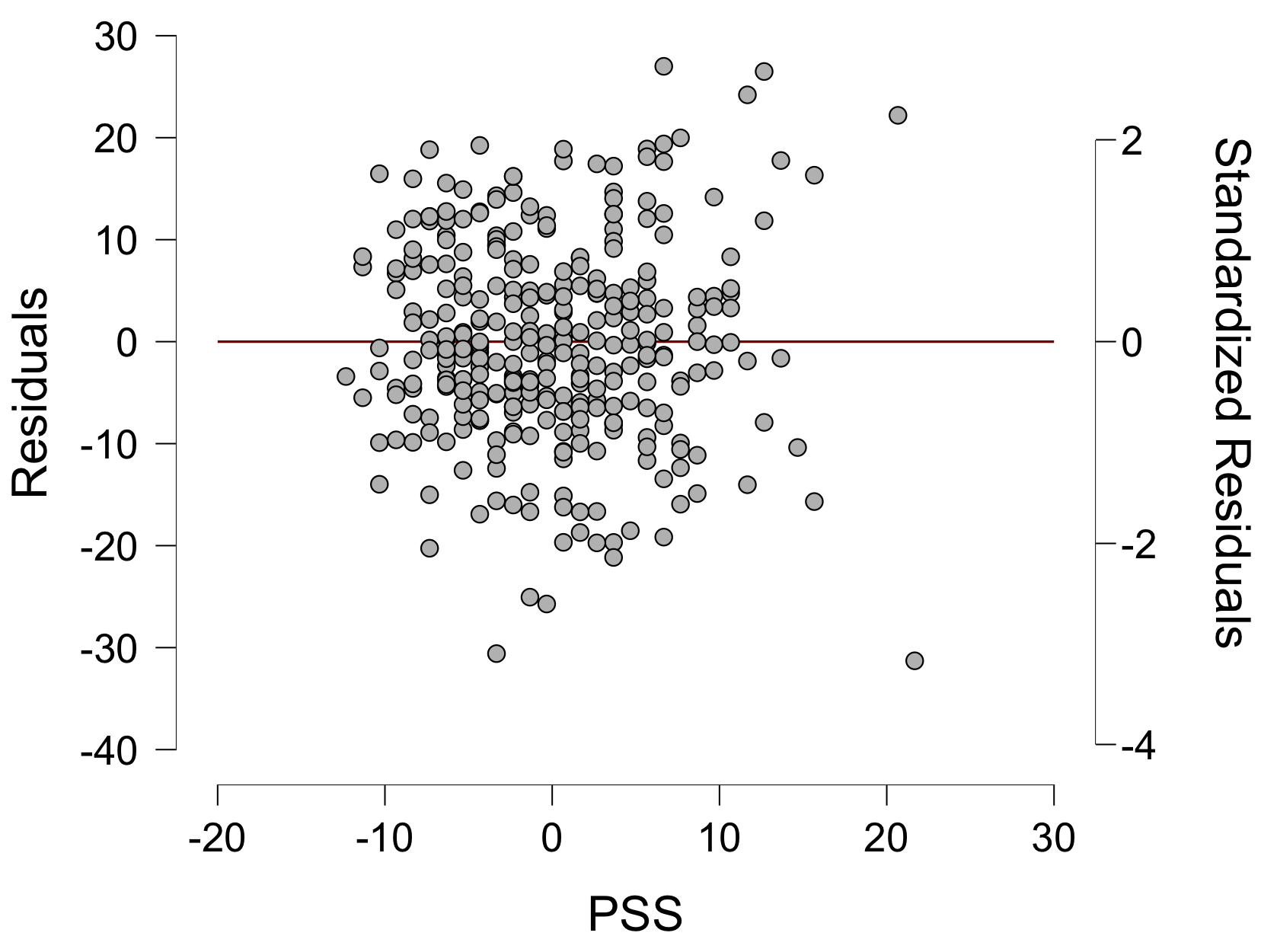

Supplement: sj-jasp-3-hpq-10.1177_13591053211059393 – Supplemental material for Individual factors in the relationship between stress and resilience in mental health psychology practitioners during the COVID-19 pandemic [file sj-jasp-3-hpq-10.1177_13591053211059393.jasp › resources/11/_114_t1602546809861.png]

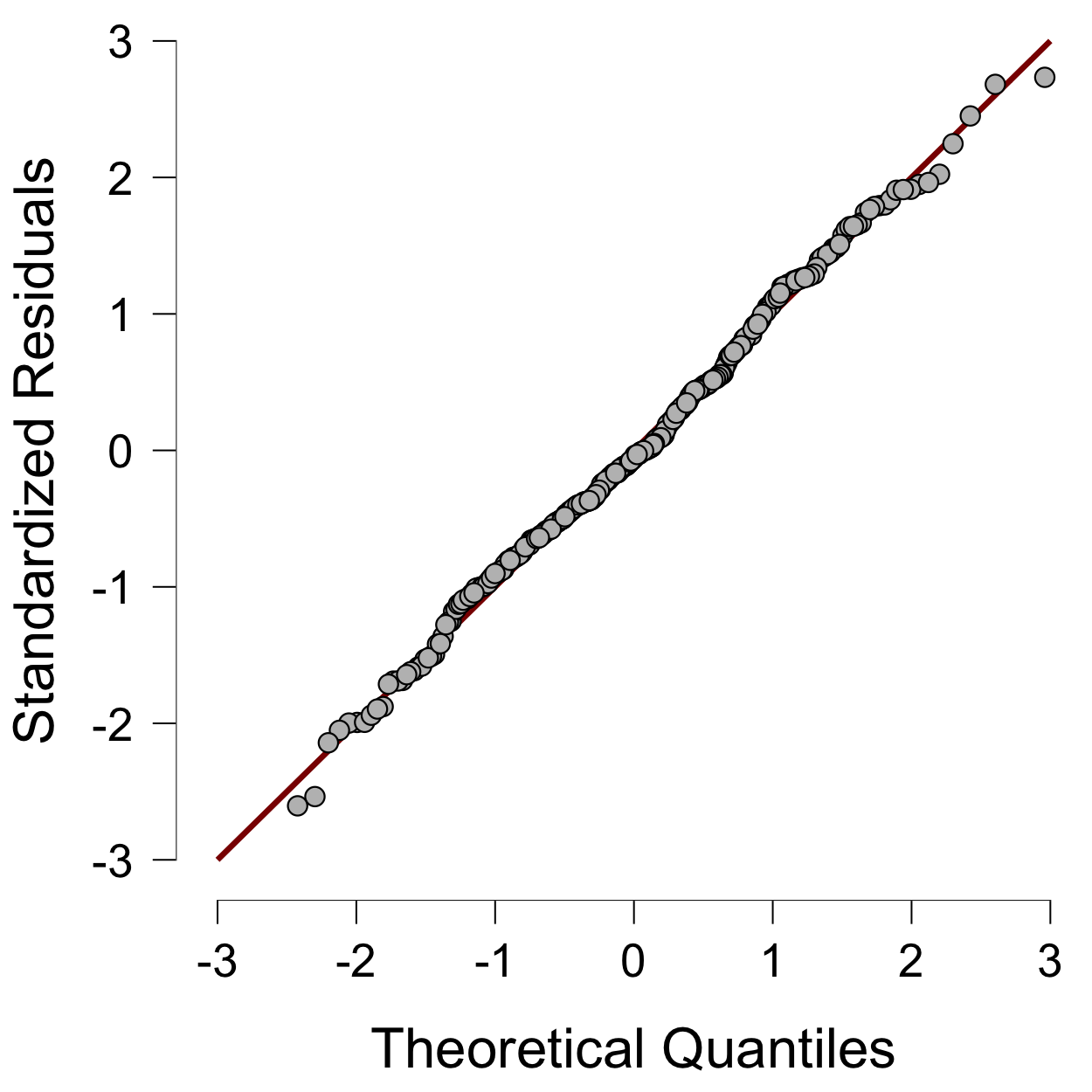

Supplement: sj-jasp-3-hpq-10.1177_13591053211059393 – Supplemental material for Individual factors in the relationship between stress and resilience in mental health psychology practitioners during the COVID-19 pandemic [file sj-jasp-3-hpq-10.1177_13591053211059393.jasp › resources/11/_2_t1602546812374.png]

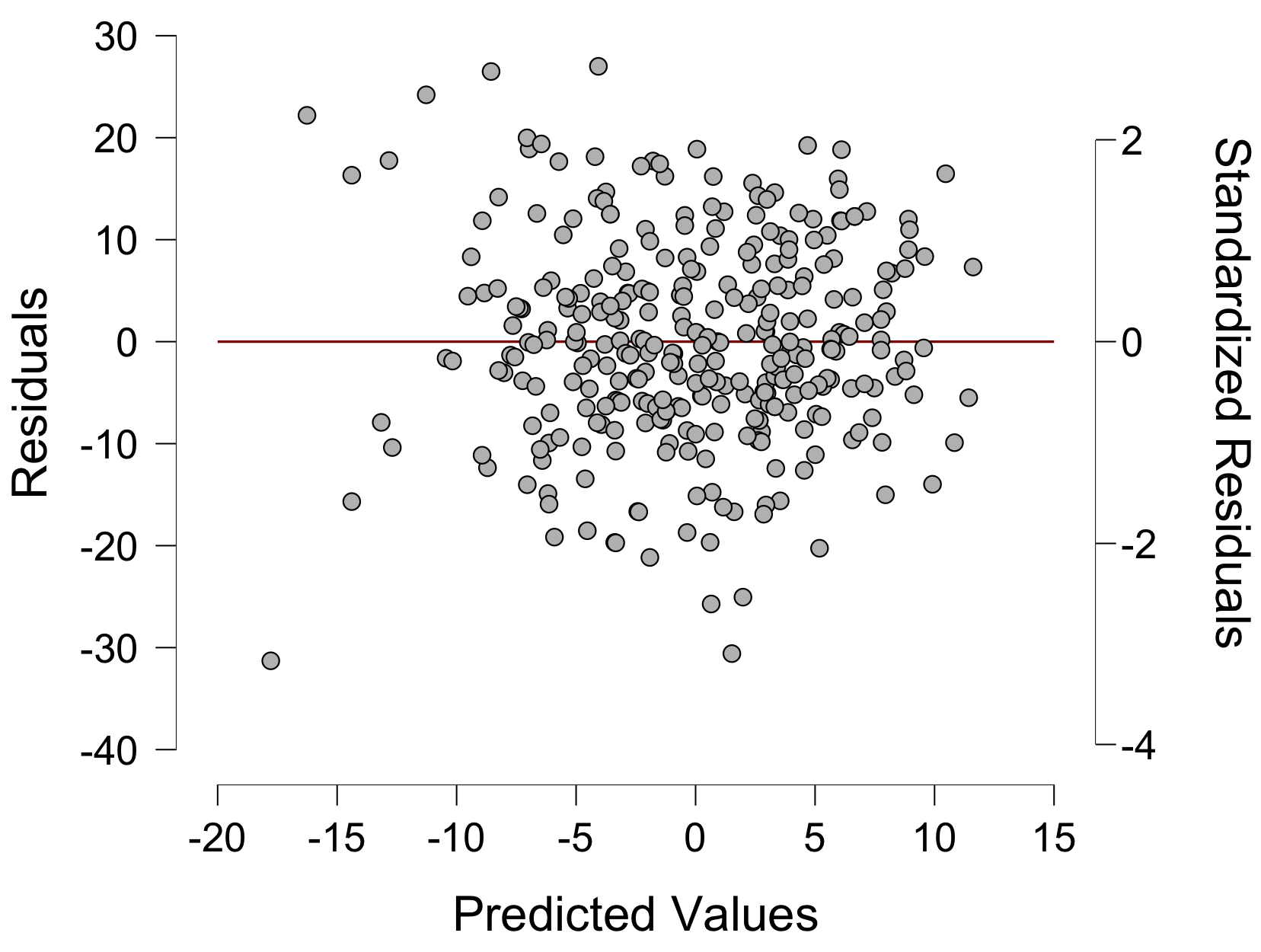

Supplement: sj-jasp-3-hpq-10.1177_13591053211059393 – Supplemental material for Individual factors in the relationship between stress and resilience in mental health psychology practitioners during the COVID-19 pandemic [file sj-jasp-3-hpq-10.1177_13591053211059393.jasp › resources/11/_0_t1602546811014.png]

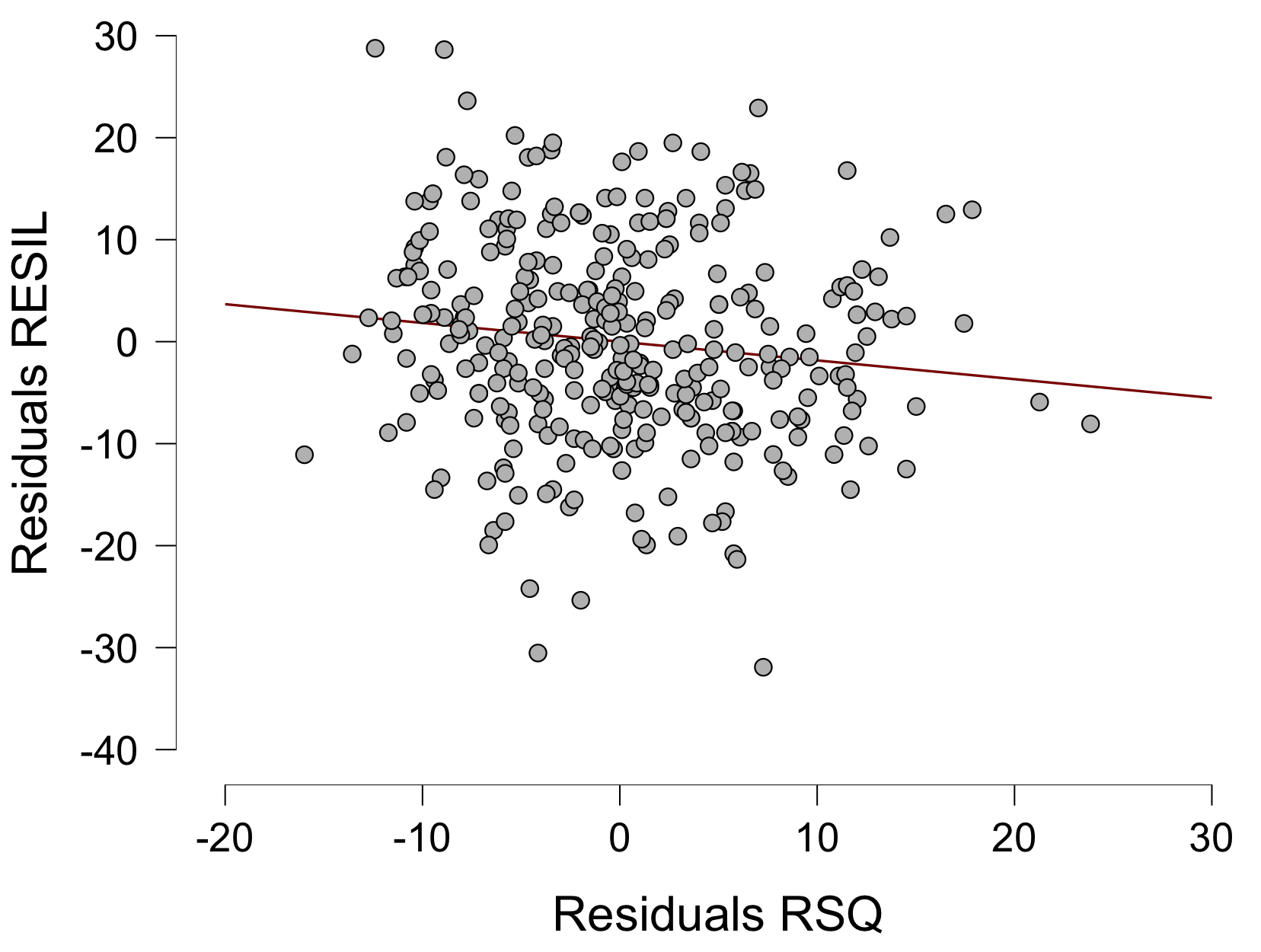

Supplement: sj-jasp-3-hpq-10.1177_13591053211059393 – Supplemental material for Individual factors in the relationship between stress and resilience in mental health psychology practitioners during the COVID-19 pandemic [file sj-jasp-3-hpq-10.1177_13591053211059393.jasp › resources/11/_0_t1602546816349.png]

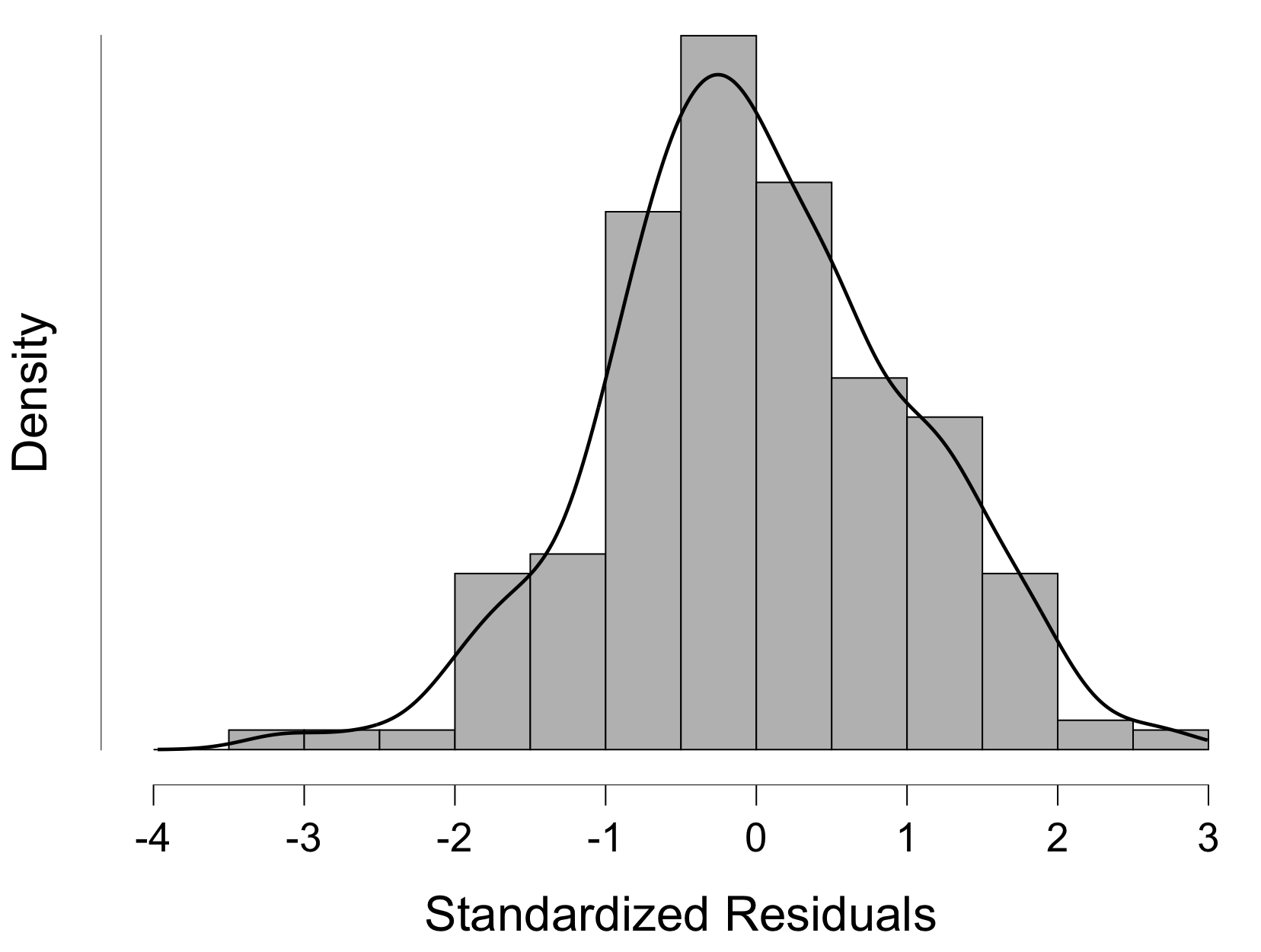

Supplement: sj-jasp-3-hpq-10.1177_13591053211059393 – Supplemental material for Individual factors in the relationship between stress and resilience in mental health psychology practitioners during the COVID-19 pandemic [file sj-jasp-3-hpq-10.1177_13591053211059393.jasp › resources/11/_1_t1602546811398.png]

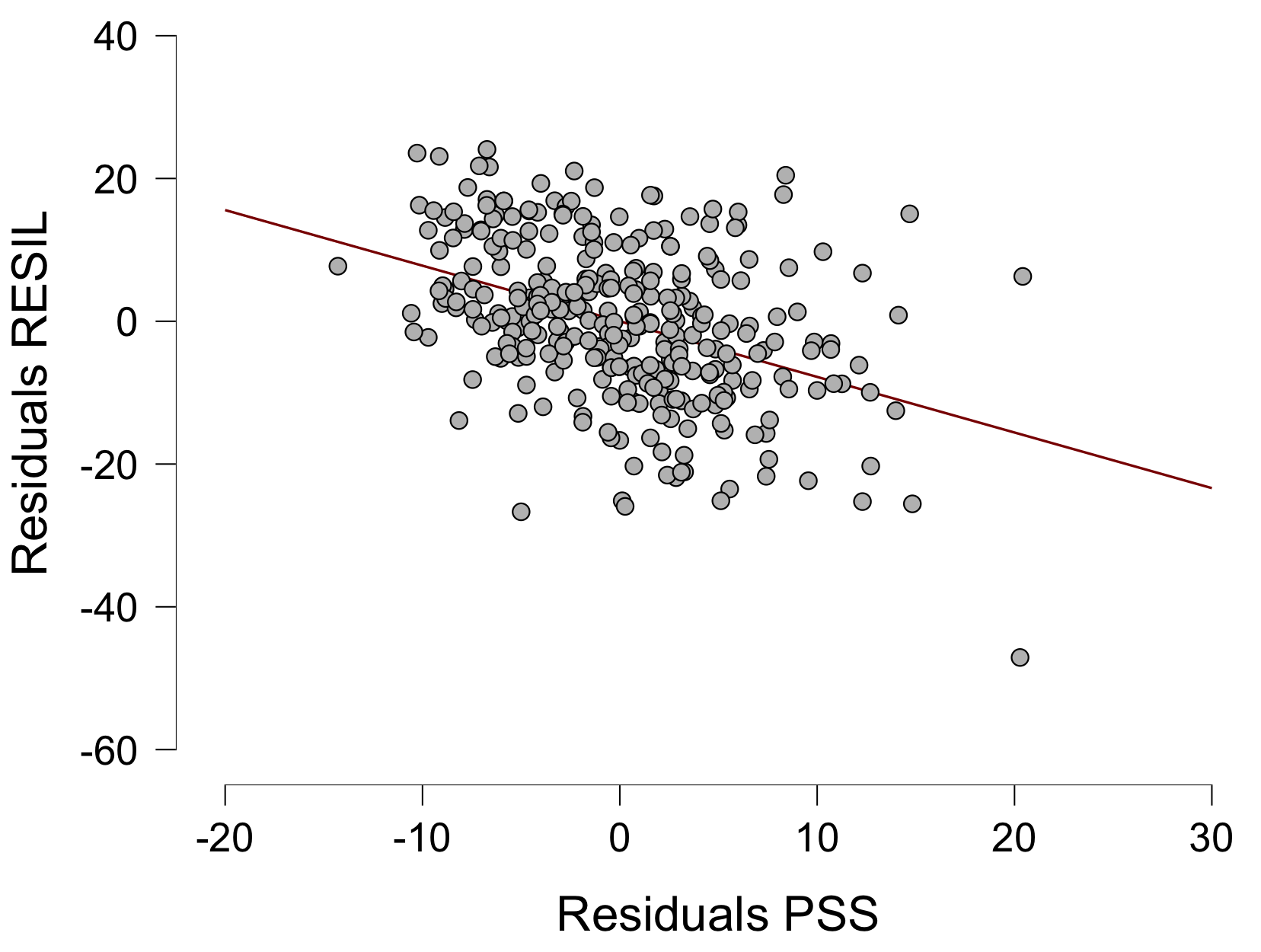

Supplement: sj-jasp-3-hpq-10.1177_13591053211059393 – Supplemental material for Individual factors in the relationship between stress and resilience in mental health psychology practitioners during the COVID-19 pandemic [file sj-jasp-3-hpq-10.1177_13591053211059393.jasp › resources/11/_1_t1602546816975.png]

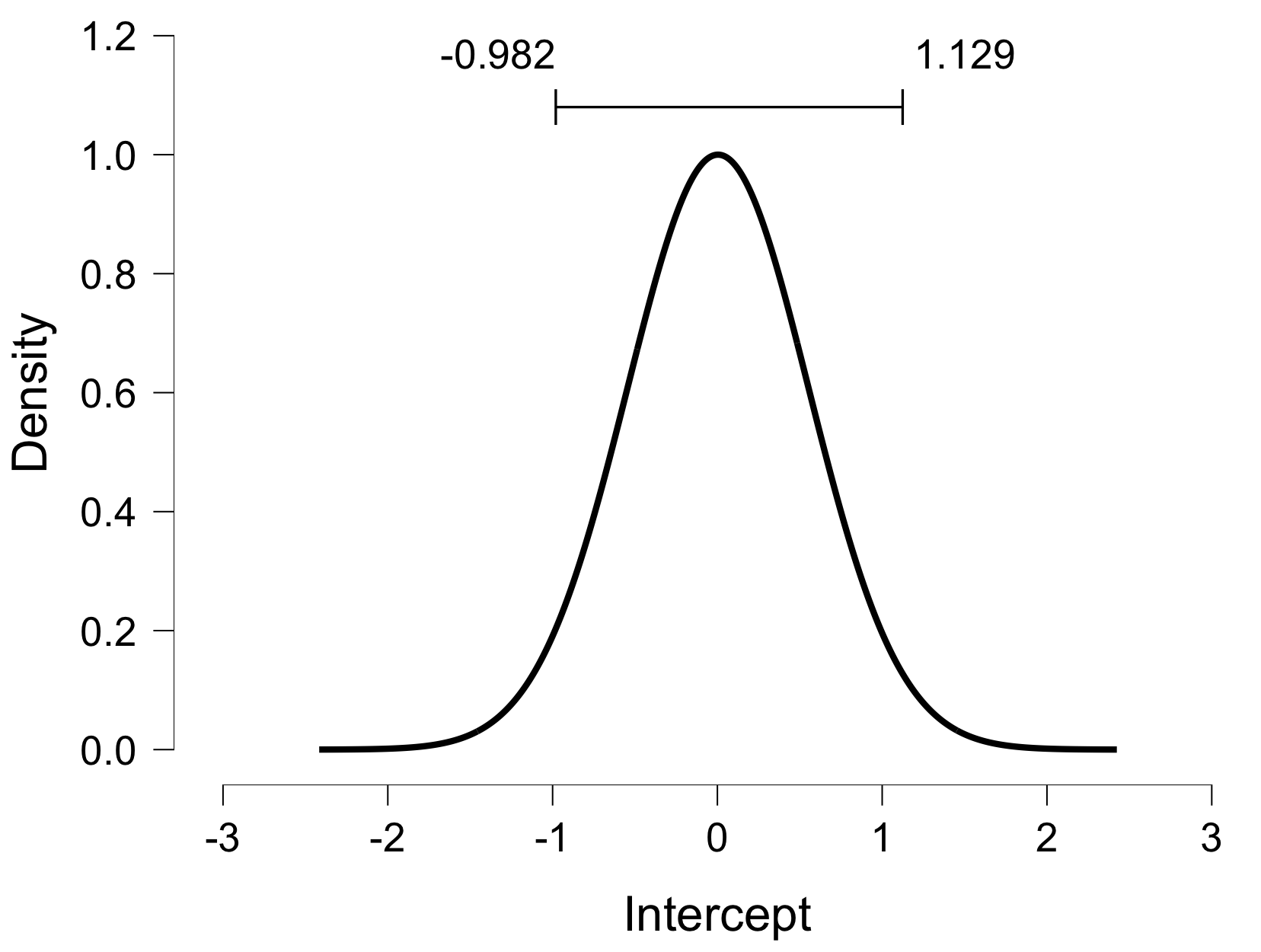

Supplement: sj-jasp-3-hpq-10.1177_13591053211059393 – Supplemental material for Individual factors in the relationship between stress and resilience in mental health psychology practitioners during the COVID-19 pandemic [file sj-jasp-3-hpq-10.1177_13591053211059393.jasp › resources/7/_55_t1602553993708.png]

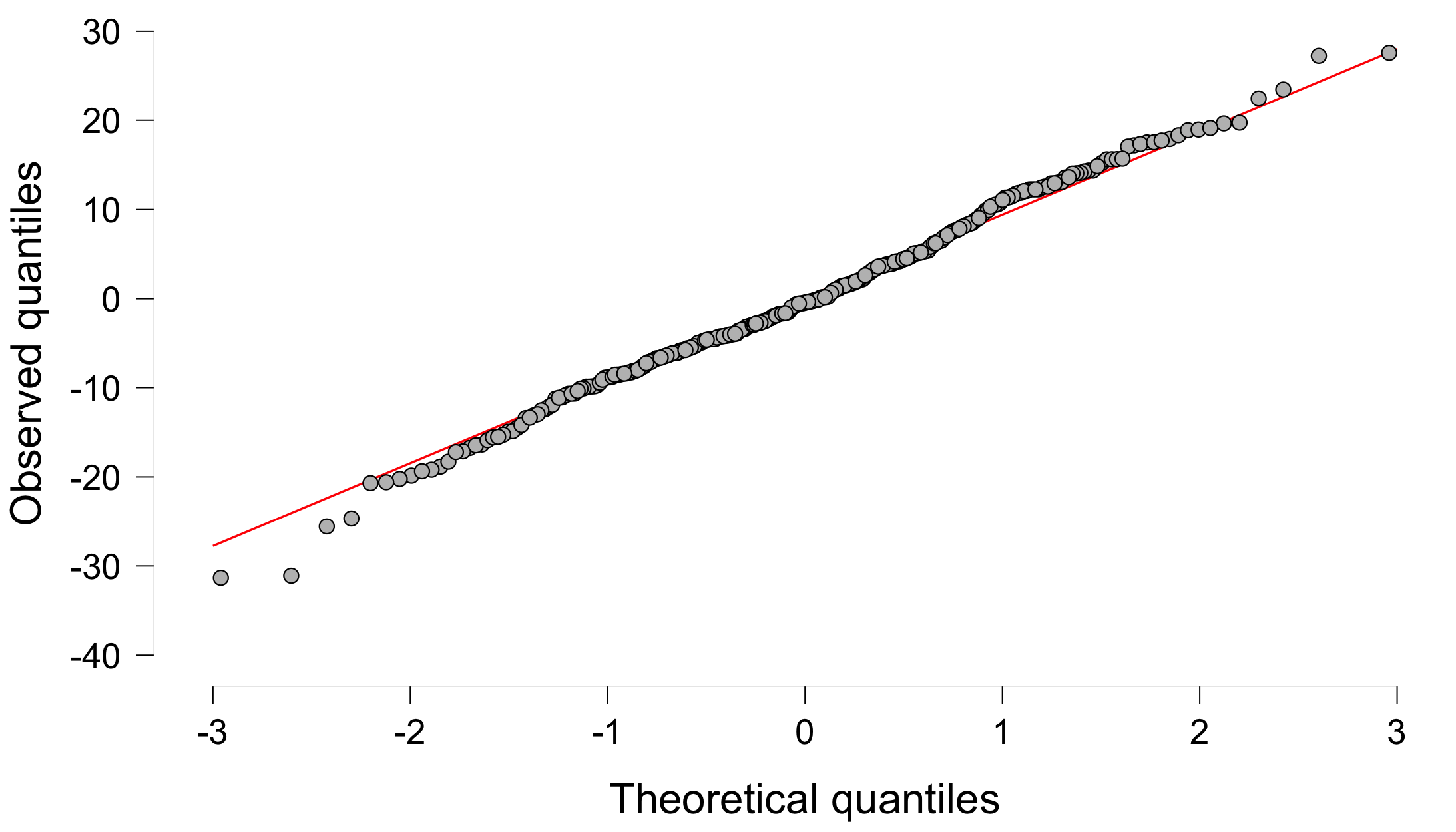

Supplement: sj-jasp-3-hpq-10.1177_13591053211059393 – Supplemental material for Individual factors in the relationship between stress and resilience in mental health psychology practitioners during the COVID-19 pandemic [file sj-jasp-3-hpq-10.1177_13591053211059393.jasp › resources/7/_58_t1602554726378.png]

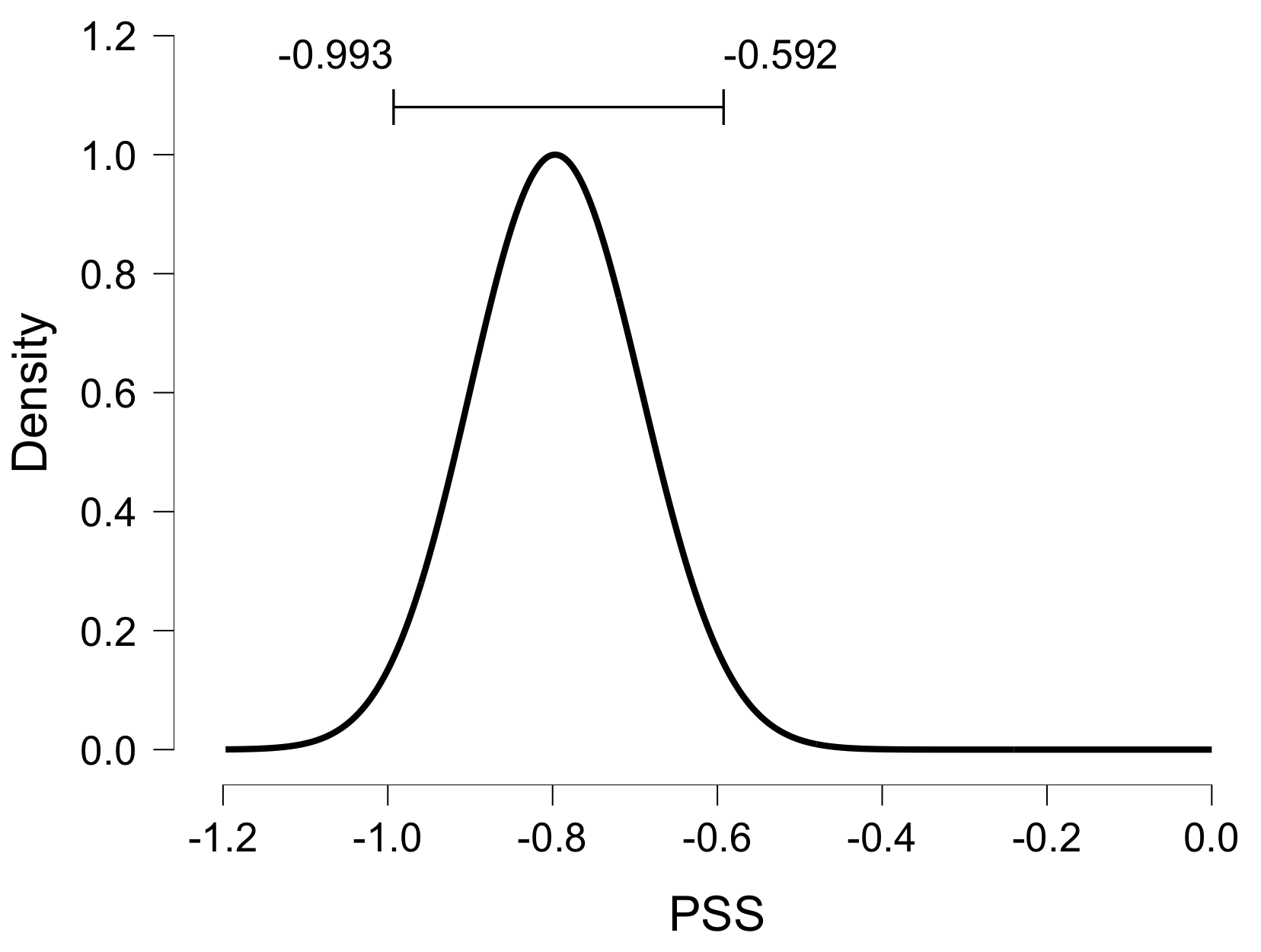

Supplement: sj-jasp-3-hpq-10.1177_13591053211059393 – Supplemental material for Individual factors in the relationship between stress and resilience in mental health psychology practitioners during the COVID-19 pandemic [file sj-jasp-3-hpq-10.1177_13591053211059393.jasp › resources/7/_57_t1602553994436.png]

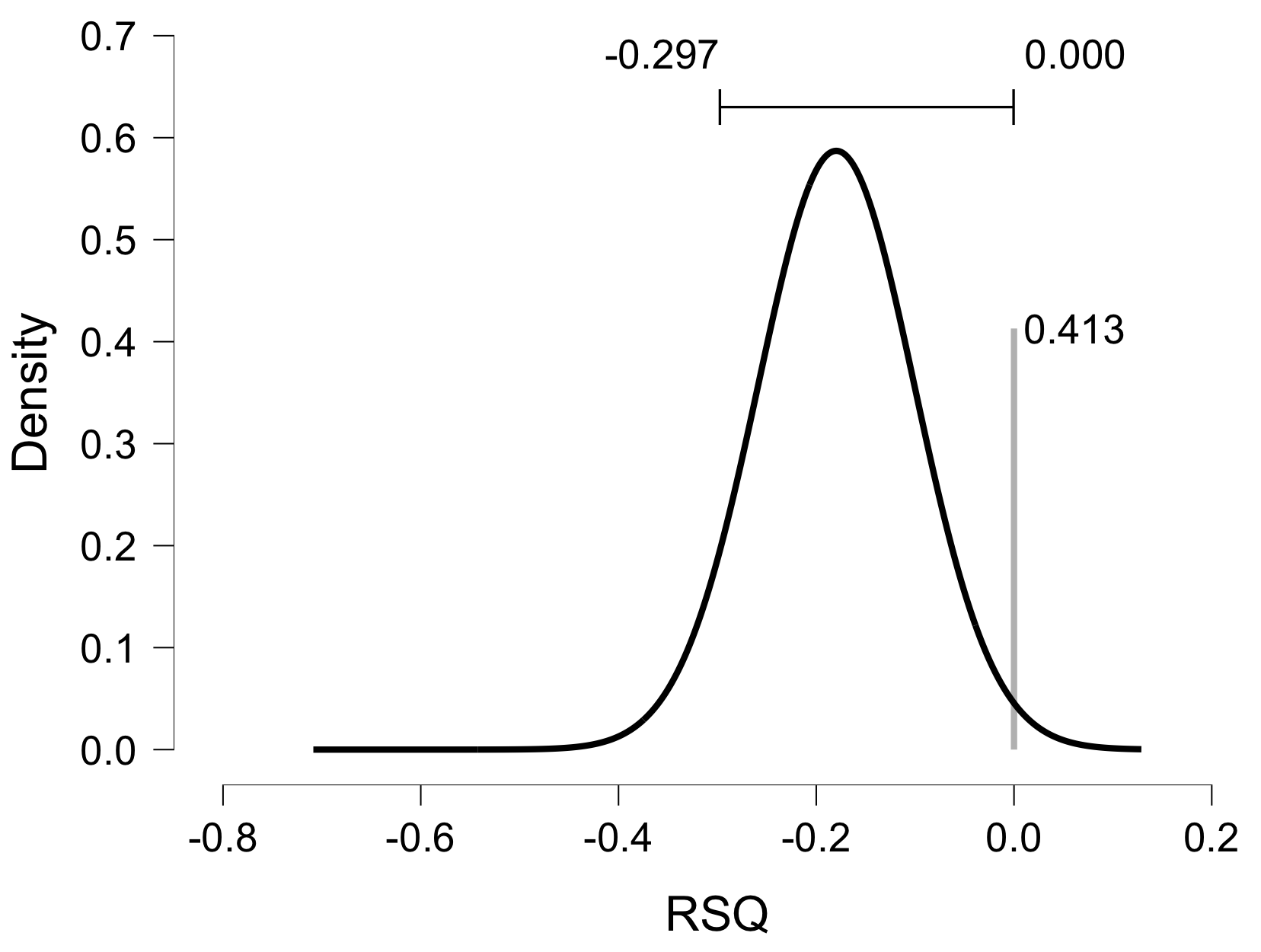

Supplement: sj-jasp-3-hpq-10.1177_13591053211059393 – Supplemental material for Individual factors in the relationship between stress and resilience in mental health psychology practitioners during the COVID-19 pandemic [file sj-jasp-3-hpq-10.1177_13591053211059393.jasp › resources/7/_56_t1602553994215.png]

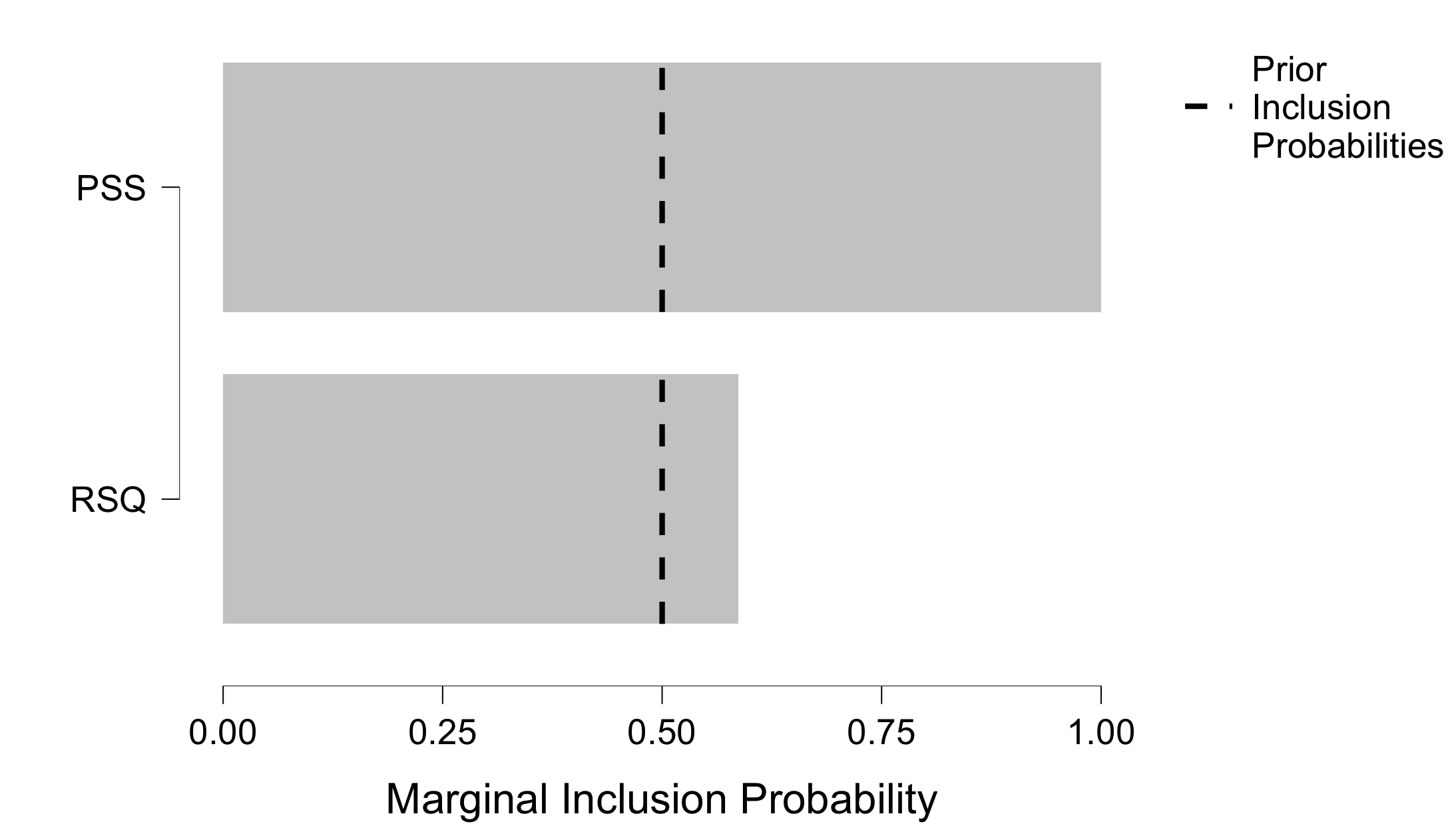

Supplement: sj-jasp-3-hpq-10.1177_13591053211059393 – Supplemental material for Individual factors in the relationship between stress and resilience in mental health psychology practitioners during the COVID-19 pandemic [file sj-jasp-3-hpq-10.1177_13591053211059393.jasp › resources/7/_54_t1602553993447.png]

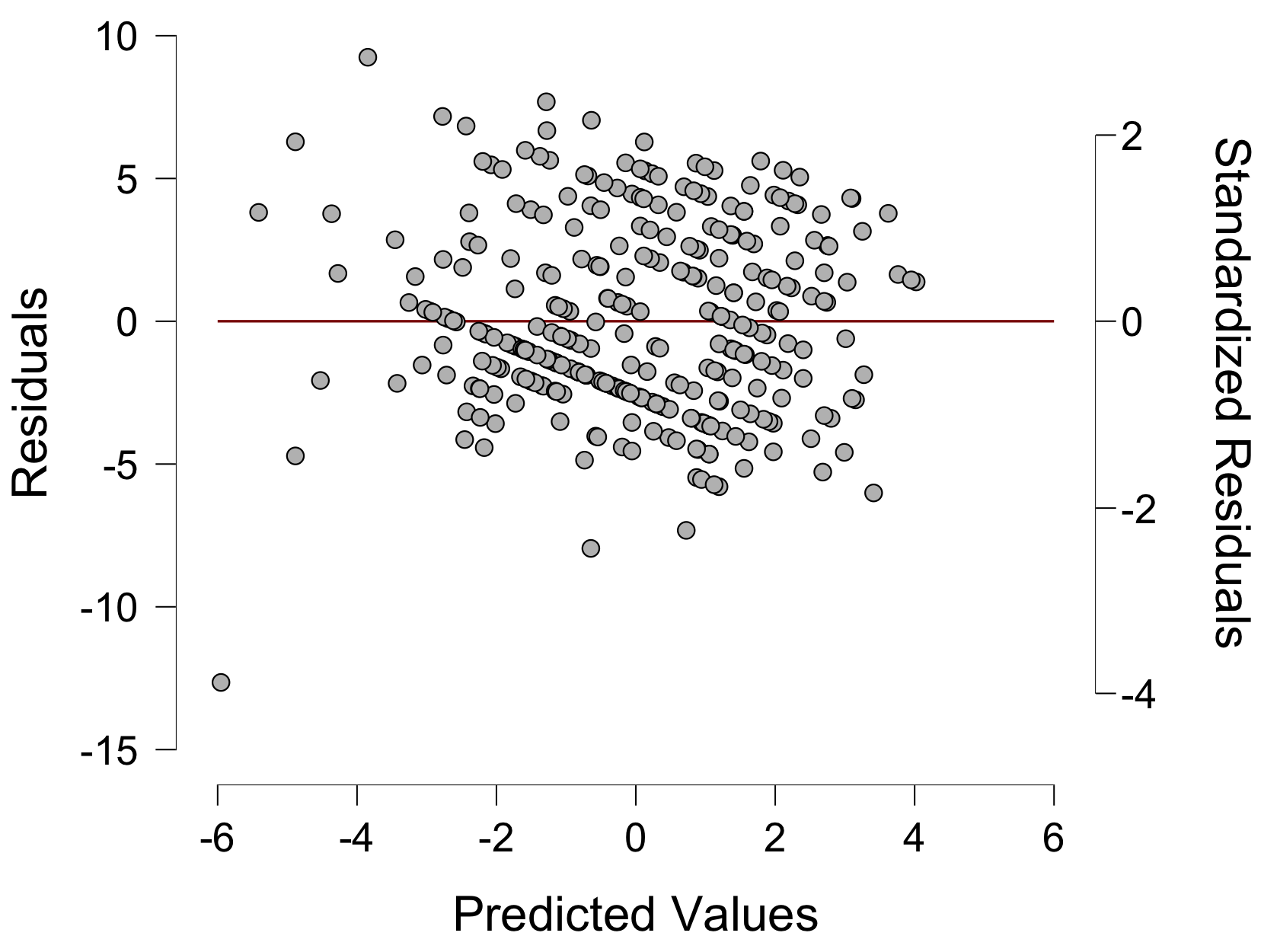

Supplement: sj-jasp-3-hpq-10.1177_13591053211059393 – Supplemental material for Individual factors in the relationship between stress and resilience in mental health psychology practitioners during the COVID-19 pandemic [file sj-jasp-3-hpq-10.1177_13591053211059393.jasp › resources/18/_9_t1603024353316.png]

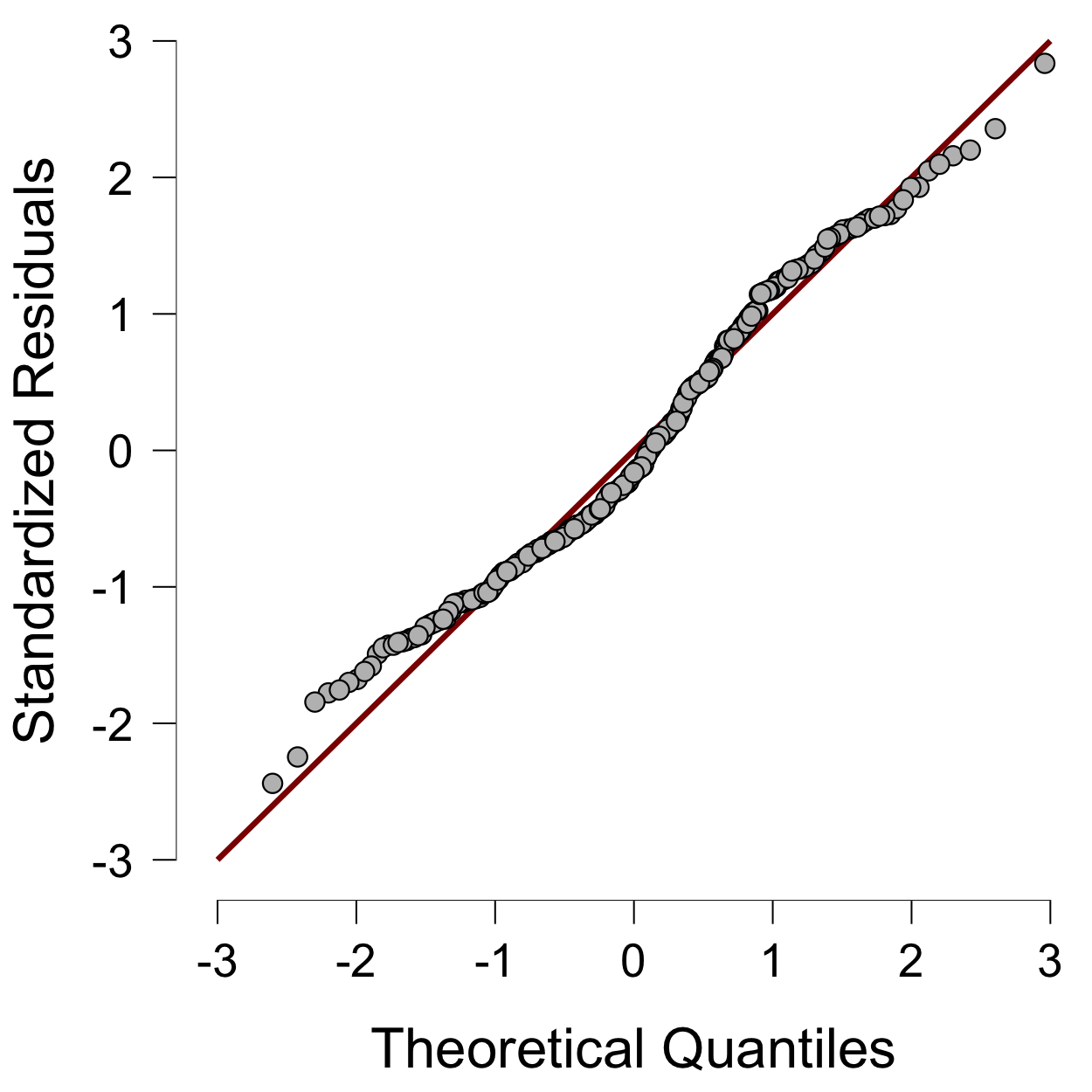

Supplement: sj-jasp-3-hpq-10.1177_13591053211059393 – Supplemental material for Individual factors in the relationship between stress and resilience in mental health psychology practitioners during the COVID-19 pandemic [file sj-jasp-3-hpq-10.1177_13591053211059393.jasp › resources/18/_10_t1603024353606.png]

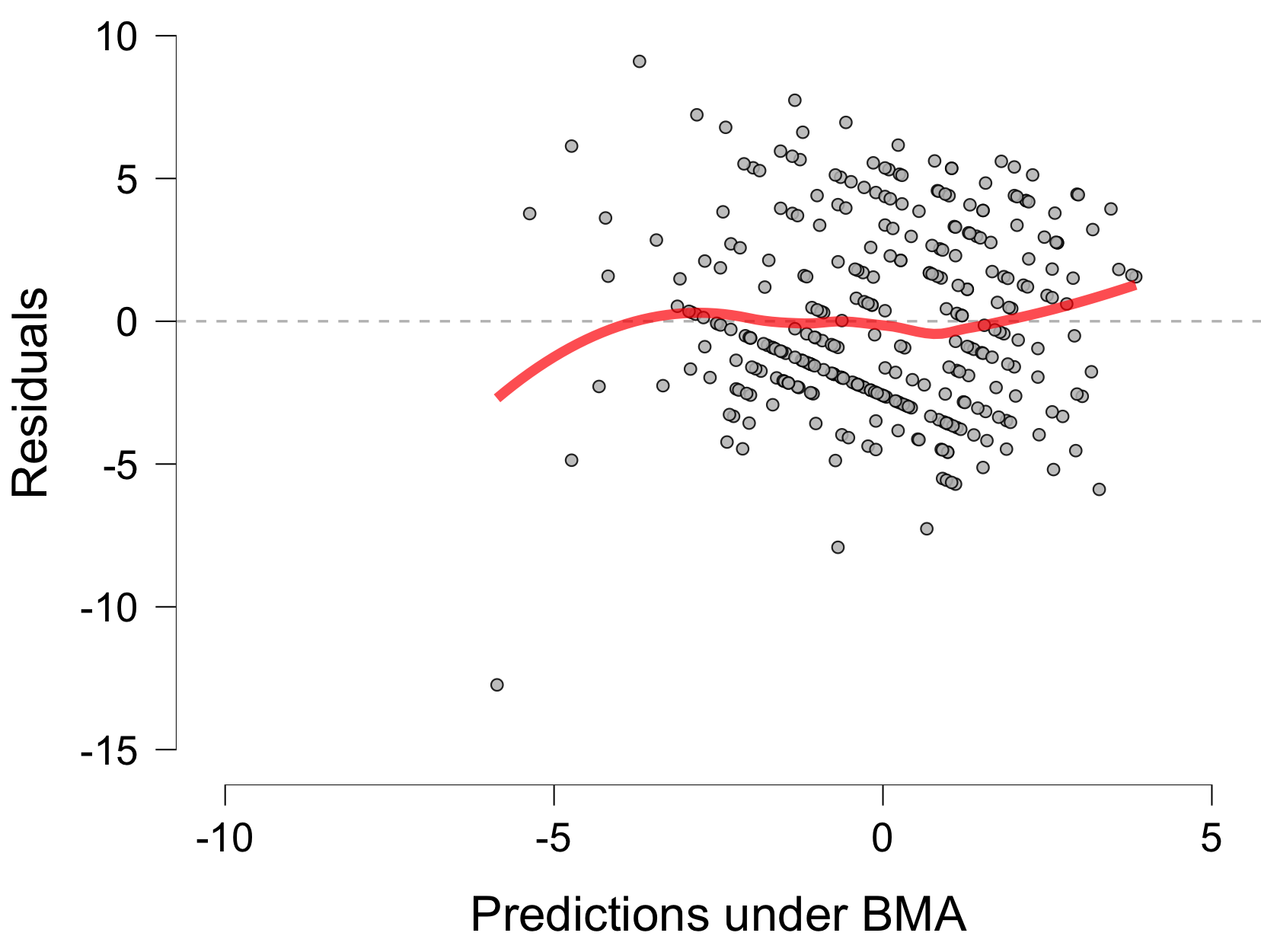

Supplement: sj-jasp-3-hpq-10.1177_13591053211059393 – Supplemental material for Individual factors in the relationship between stress and resilience in mental health psychology practitioners during the COVID-19 pandemic [file sj-jasp-3-hpq-10.1177_13591053211059393.jasp › resources/40/_15_t1603024493342.png]

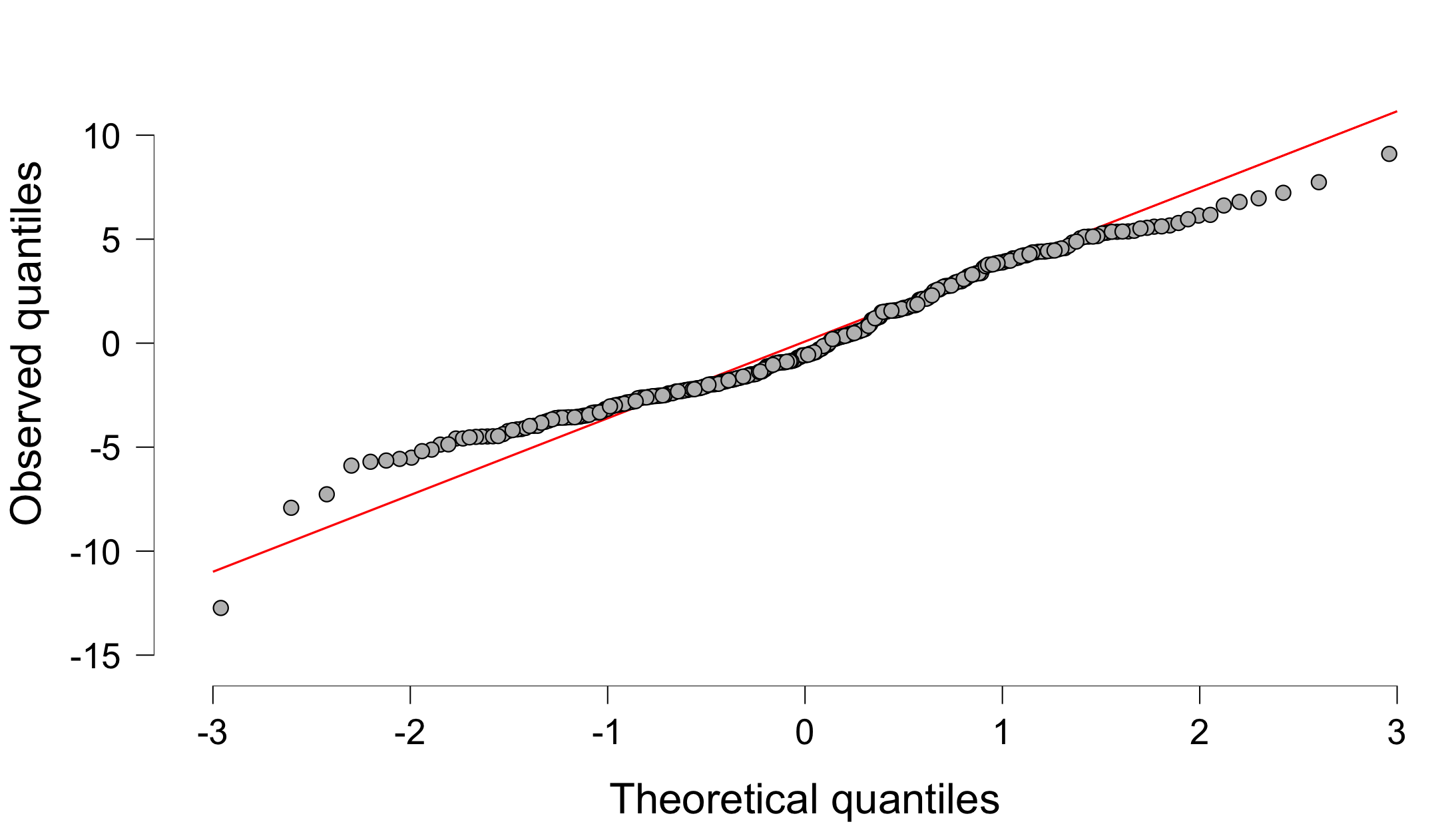

Supplement: sj-jasp-3-hpq-10.1177_13591053211059393 – Supplemental material for Individual factors in the relationship between stress and resilience in mental health psychology practitioners during the COVID-19 pandemic [file sj-jasp-3-hpq-10.1177_13591053211059393.jasp › resources/40/_16_t1603024493581.png]

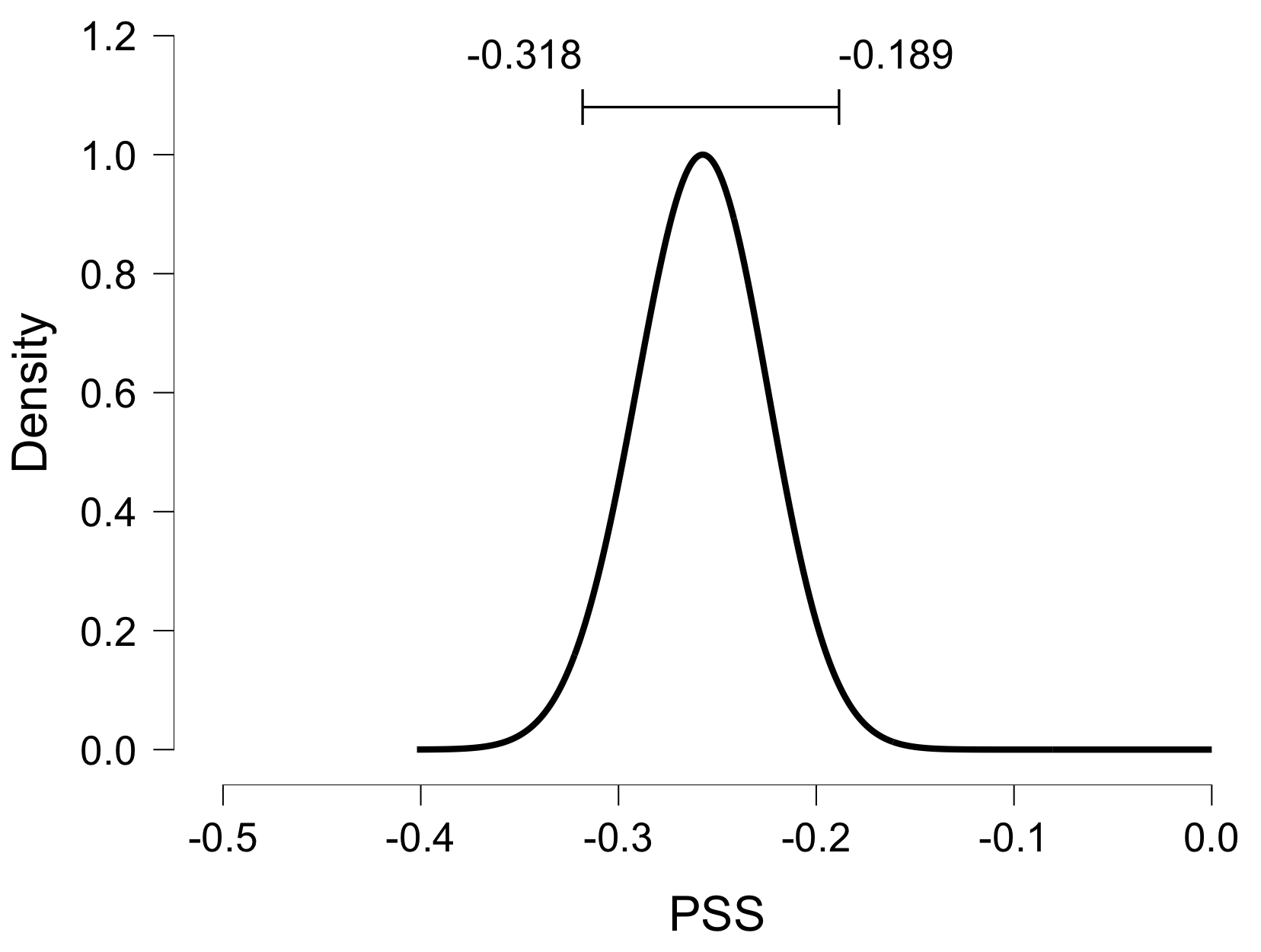

Supplement: sj-jasp-3-hpq-10.1177_13591053211059393 – Supplemental material for Individual factors in the relationship between stress and resilience in mental health psychology practitioners during the COVID-19 pandemic [file sj-jasp-3-hpq-10.1177_13591053211059393.jasp › resources/40/_18_t1603024494051.png]

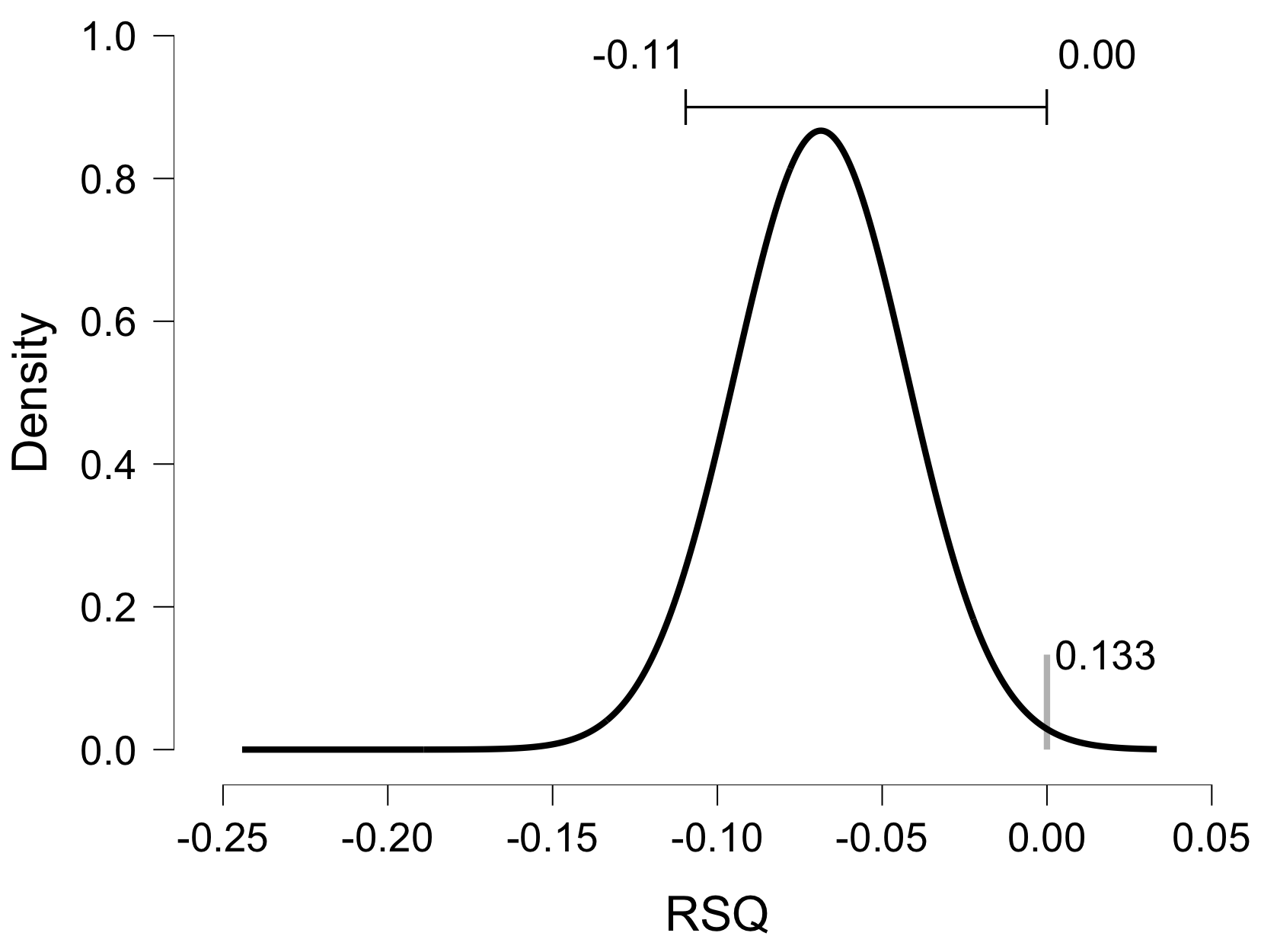

Supplement: sj-jasp-3-hpq-10.1177_13591053211059393 – Supplemental material for Individual factors in the relationship between stress and resilience in mental health psychology practitioners during the COVID-19 pandemic [file sj-jasp-3-hpq-10.1177_13591053211059393.jasp › resources/40/_19_t1603024494300.png]

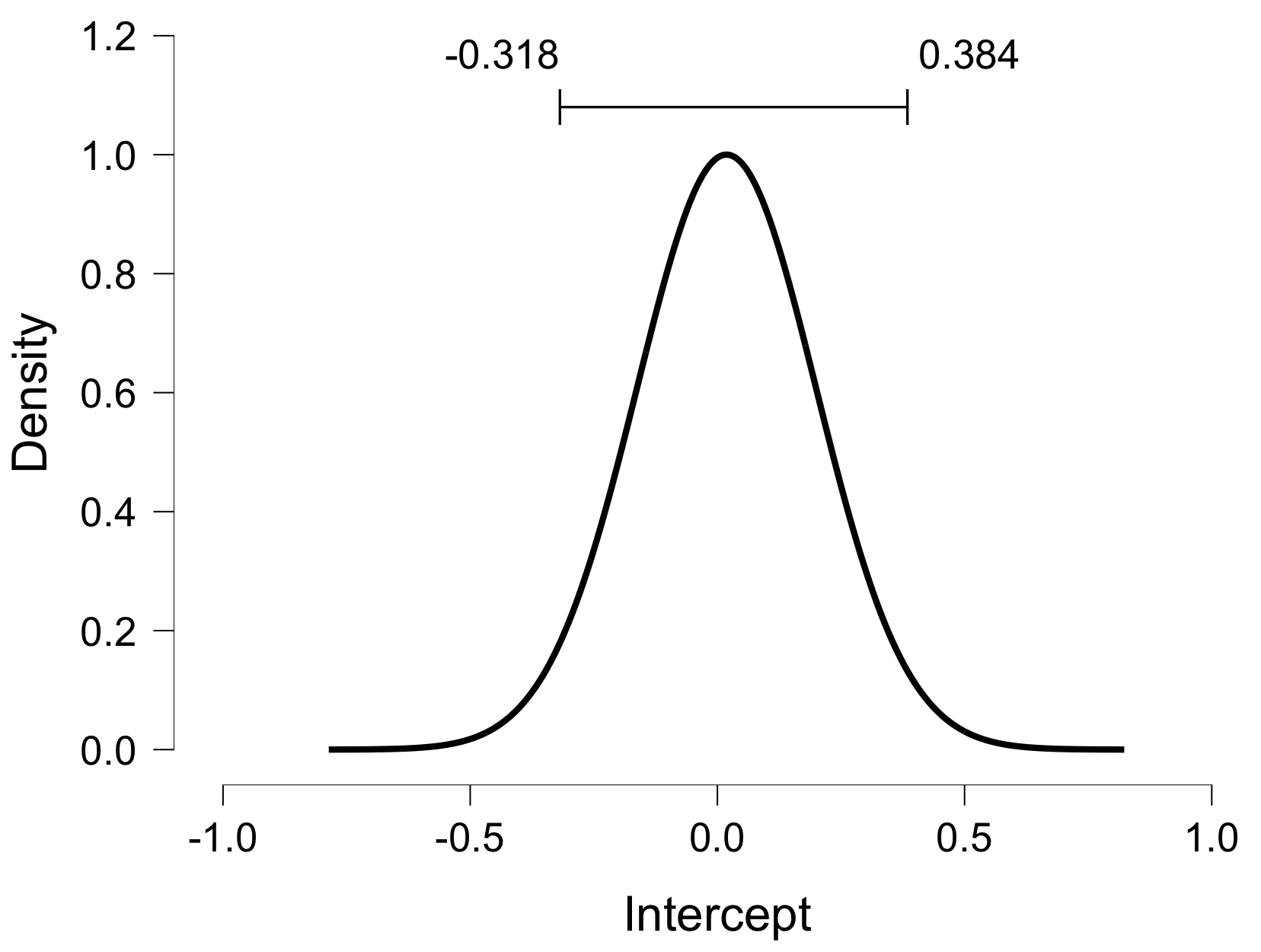

Supplement: sj-jasp-3-hpq-10.1177_13591053211059393 – Supplemental material for Individual factors in the relationship between stress and resilience in mental health psychology practitioners during the COVID-19 pandemic [file sj-jasp-3-hpq-10.1177_13591053211059393.jasp › resources/40/_17_t1603024493796.png]

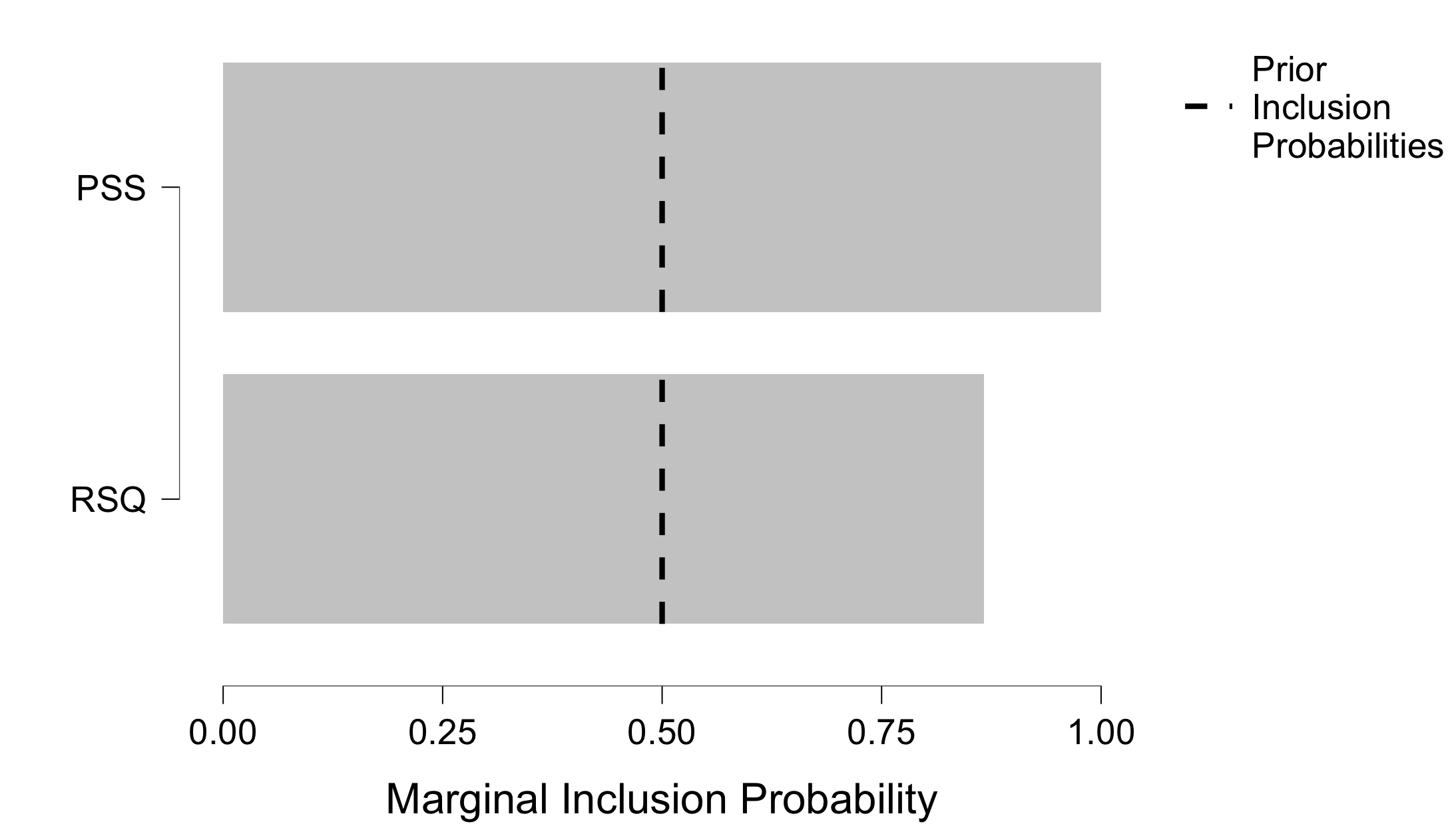

Supplement: sj-jasp-3-hpq-10.1177_13591053211059393 – Supplemental material for Individual factors in the relationship between stress and resilience in mental health psychology practitioners during the COVID-19 pandemic [file sj-jasp-3-hpq-10.1177_13591053211059393.jasp › resources/40/_11_t1603024489025.png]

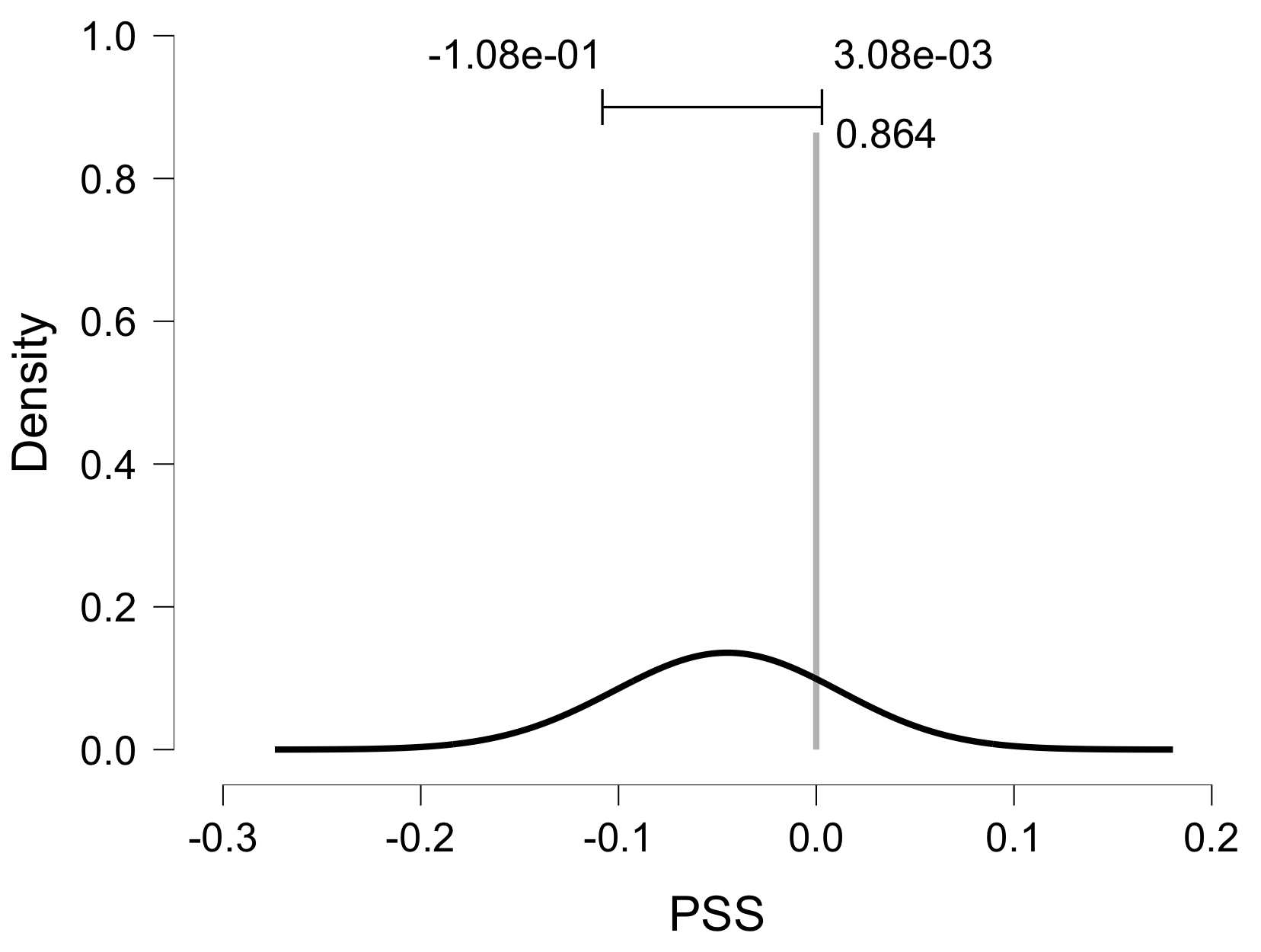

Supplement: sj-jasp-3-hpq-10.1177_13591053211059393 – Supplemental material for Individual factors in the relationship between stress and resilience in mental health psychology practitioners during the COVID-19 pandemic [file sj-jasp-3-hpq-10.1177_13591053211059393.jasp › resources/45/_3_t1603025151405.png]

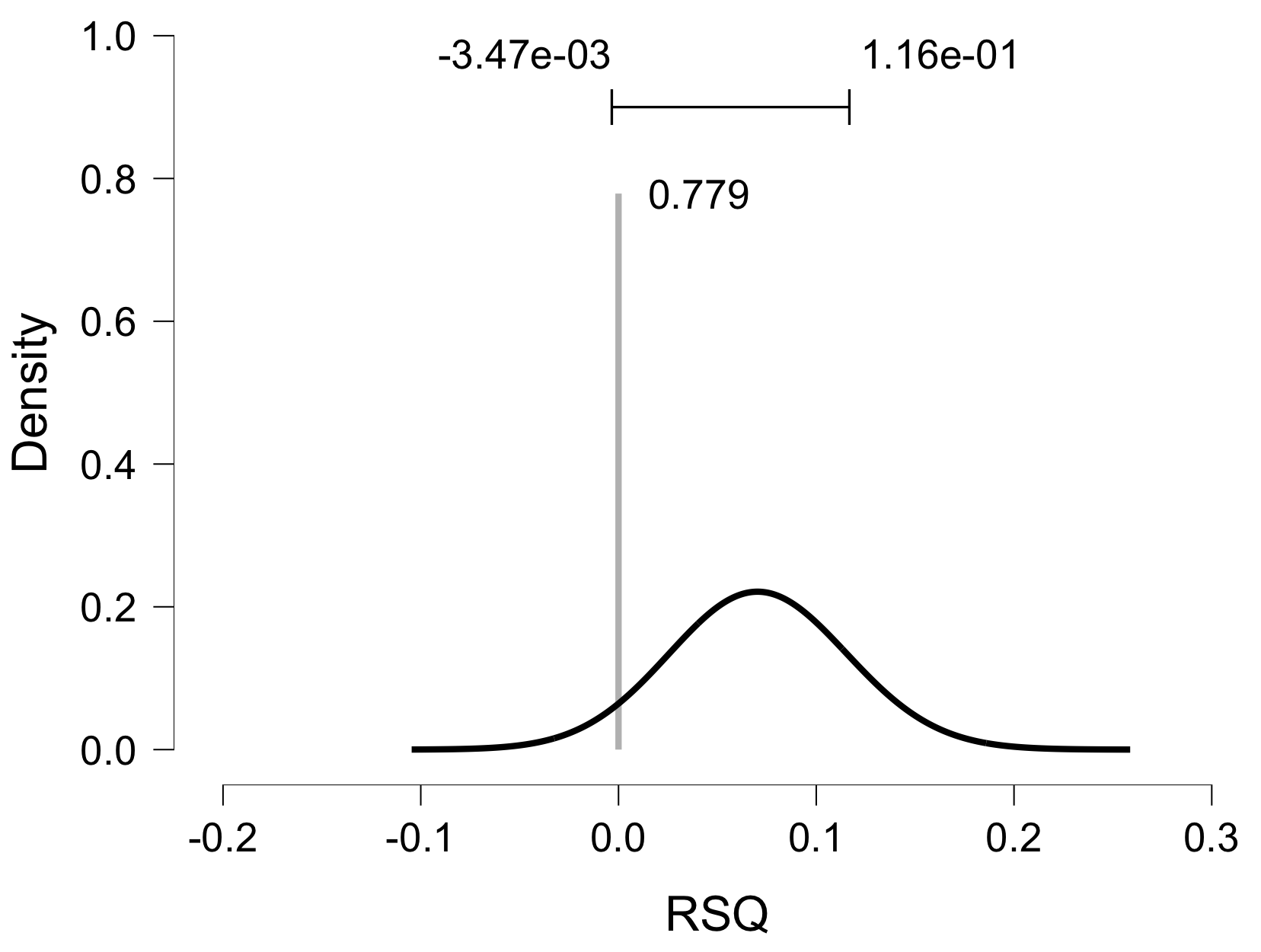

Supplement: sj-jasp-3-hpq-10.1177_13591053211059393 – Supplemental material for Individual factors in the relationship between stress and resilience in mental health psychology practitioners during the COVID-19 pandemic [file sj-jasp-3-hpq-10.1177_13591053211059393.jasp › resources/45/_4_t1603025151733.png]

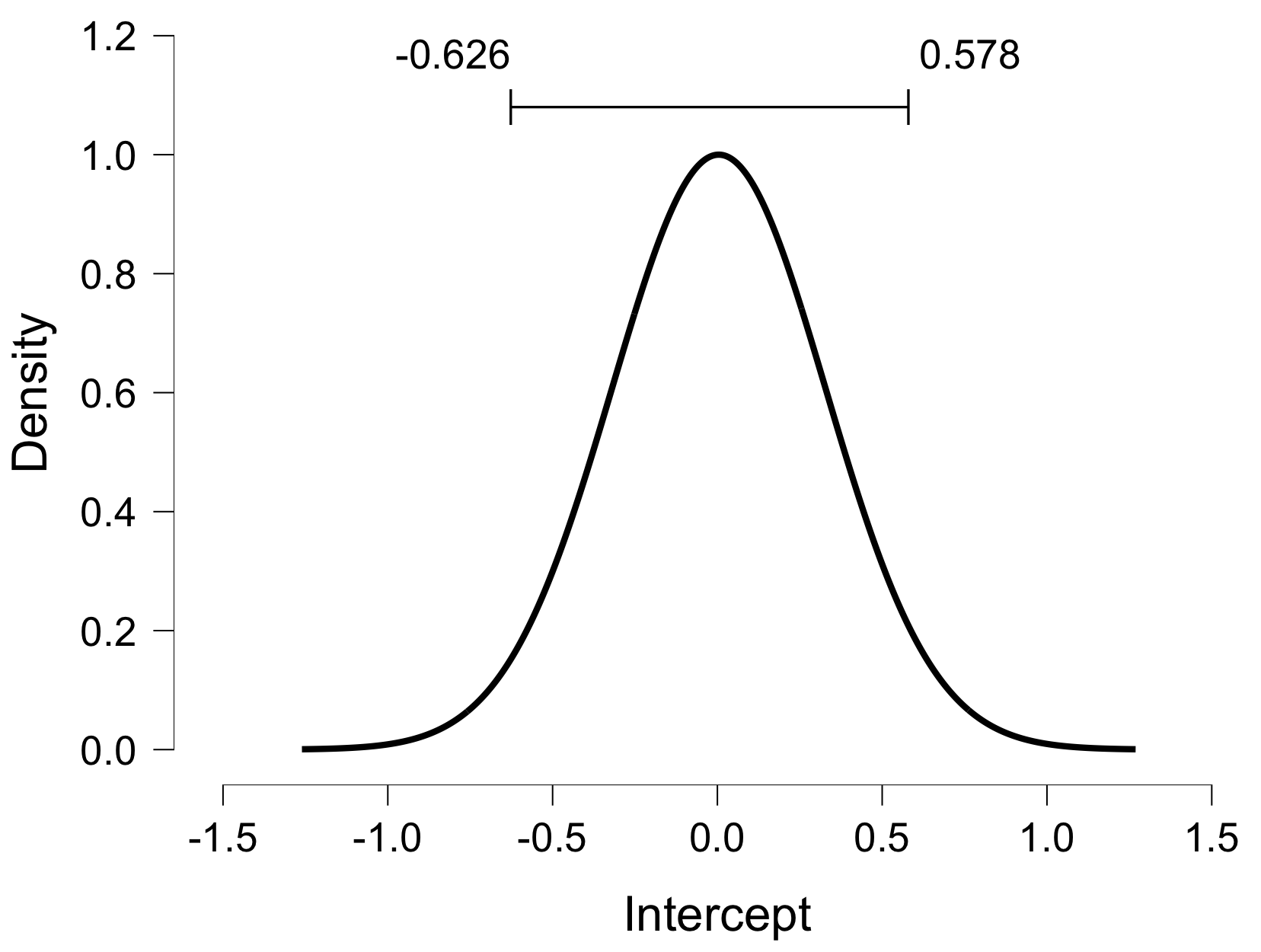

Supplement: sj-jasp-3-hpq-10.1177_13591053211059393 – Supplemental material for Individual factors in the relationship between stress and resilience in mental health psychology practitioners during the COVID-19 pandemic [file sj-jasp-3-hpq-10.1177_13591053211059393.jasp › resources/45/_2_t1603025151009.png]

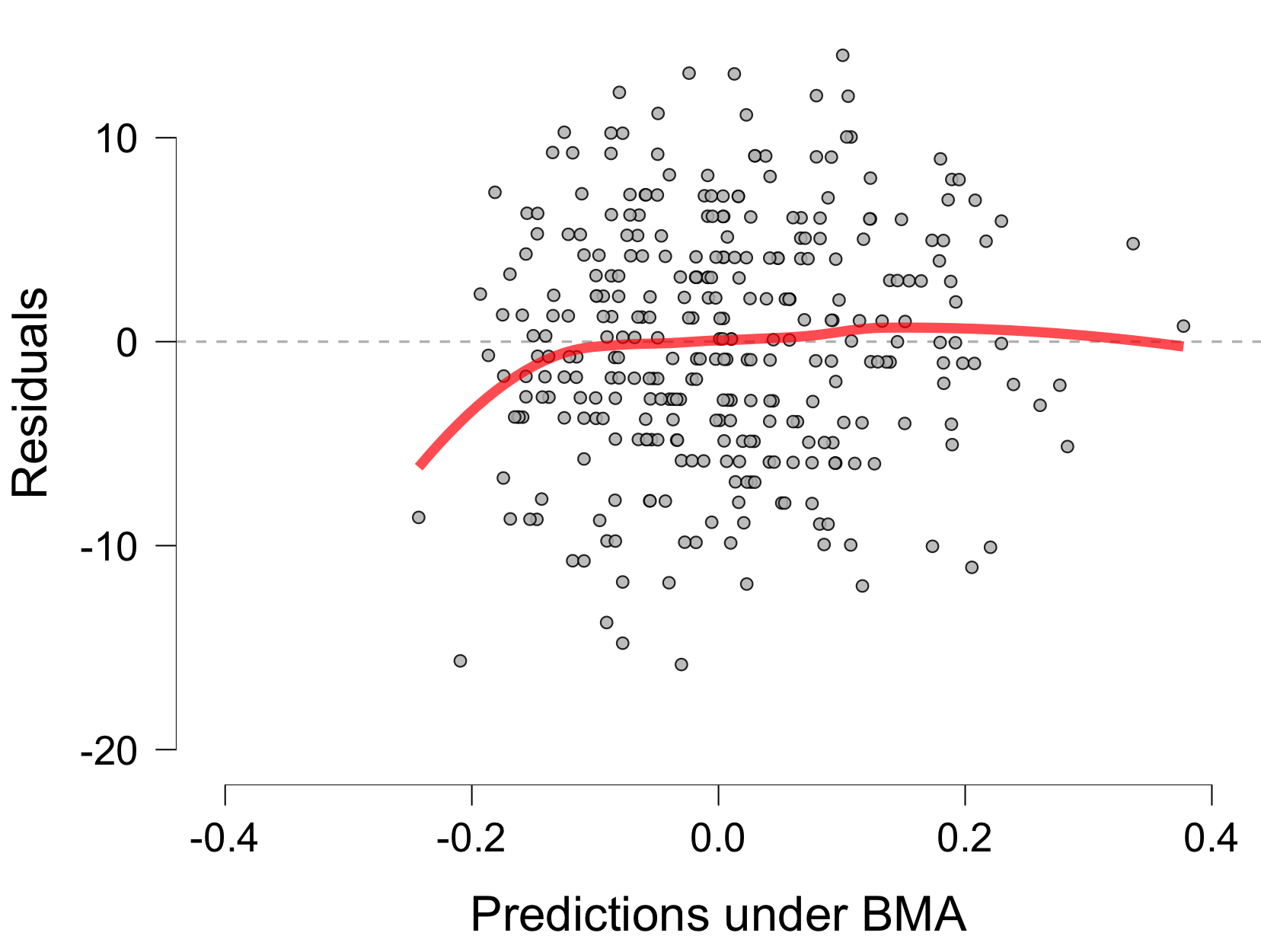

Supplement: sj-jasp-3-hpq-10.1177_13591053211059393 – Supplemental material for Individual factors in the relationship between stress and resilience in mental health psychology practitioners during the COVID-19 pandemic [file sj-jasp-3-hpq-10.1177_13591053211059393.jasp › resources/45/_21_t1603025142651.png]

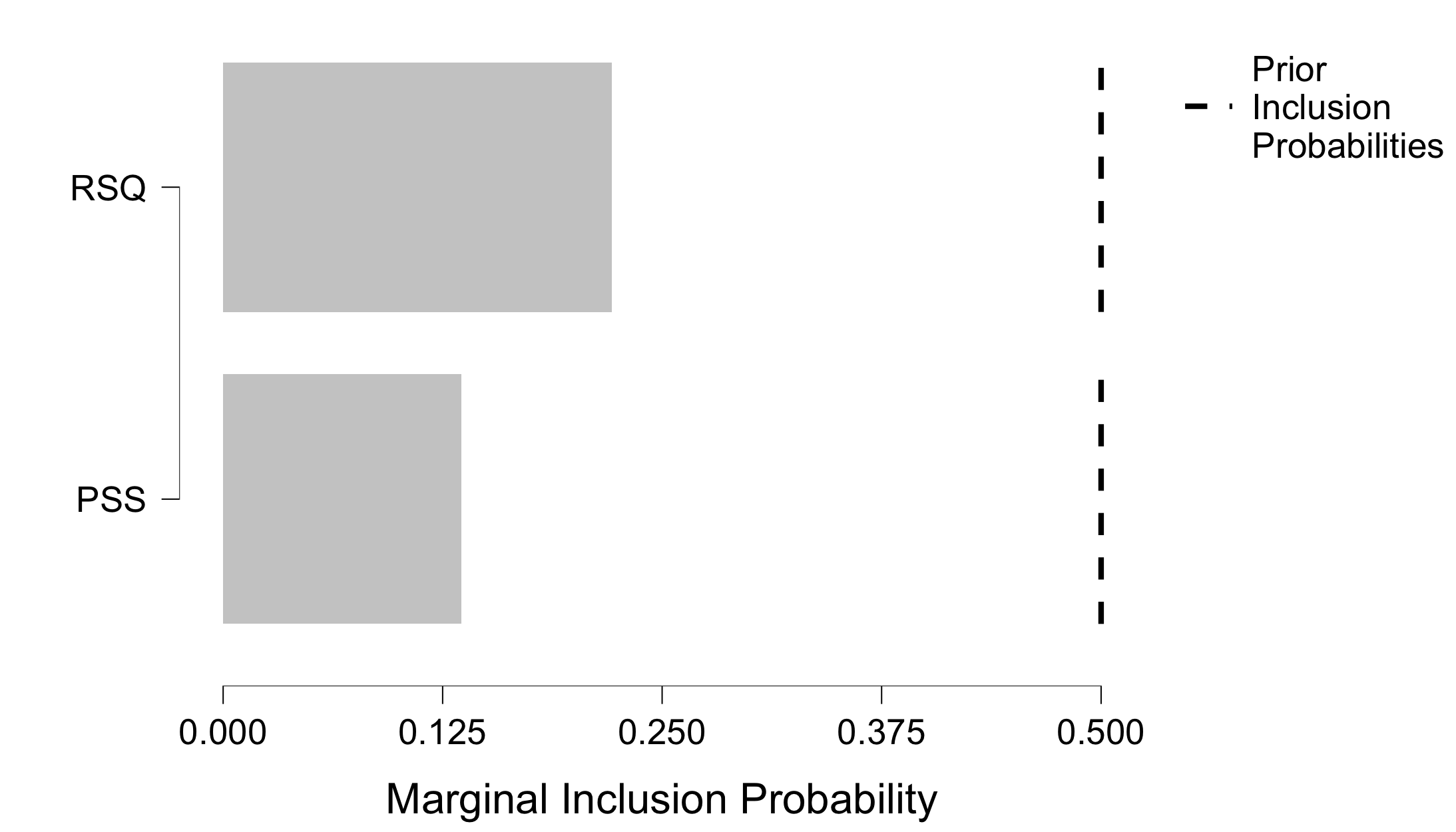

Supplement: sj-jasp-3-hpq-10.1177_13591053211059393 – Supplemental material for Individual factors in the relationship between stress and resilience in mental health psychology practitioners during the COVID-19 pandemic [file sj-jasp-3-hpq-10.1177_13591053211059393.jasp › resources/45/_0_t1603025150257.png]

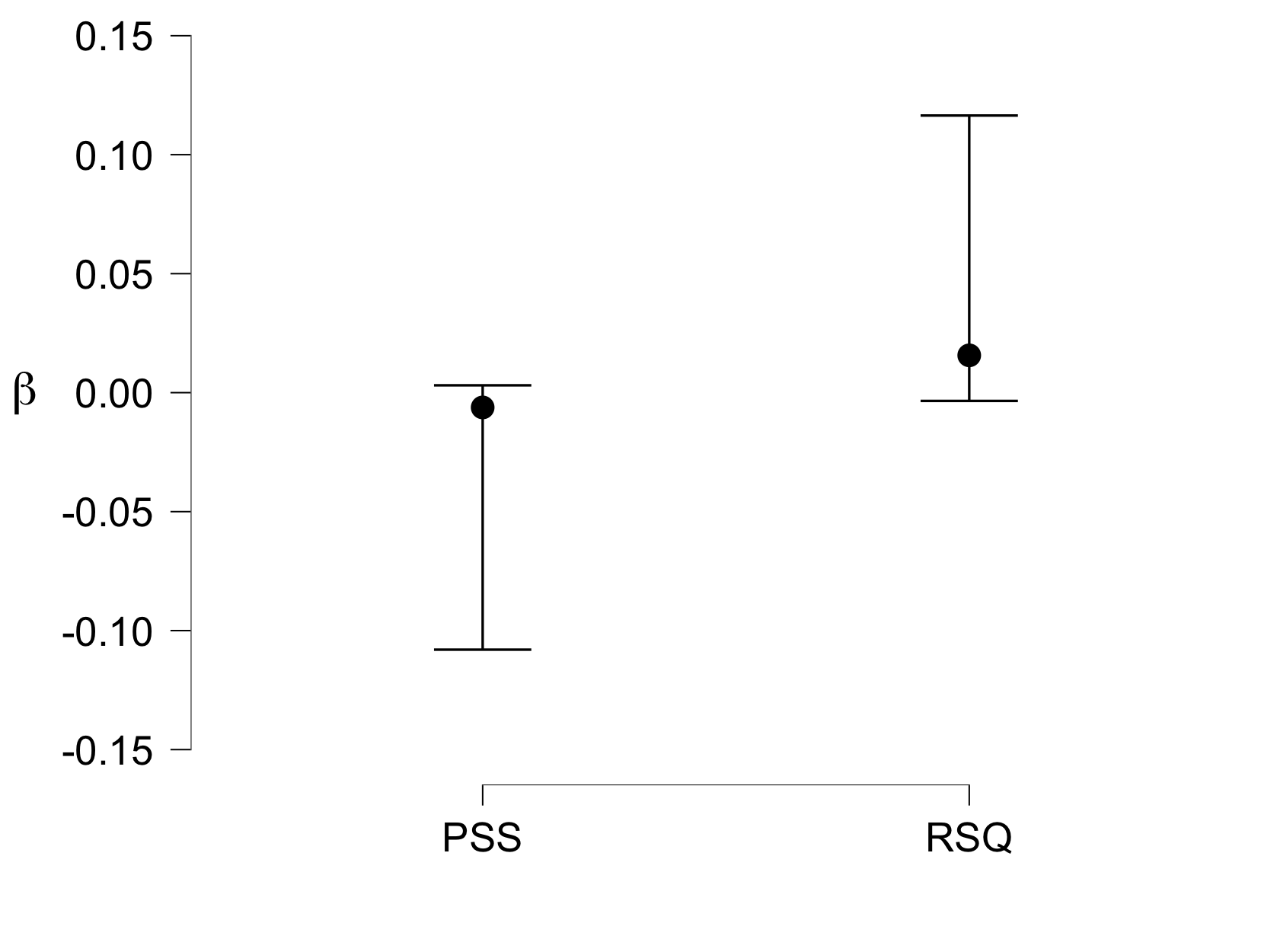

Supplement: sj-jasp-3-hpq-10.1177_13591053211059393 – Supplemental material for Individual factors in the relationship between stress and resilience in mental health psychology practitioners during the COVID-19 pandemic [file sj-jasp-3-hpq-10.1177_13591053211059393.jasp › resources/45/_0_t1603029202592.png]

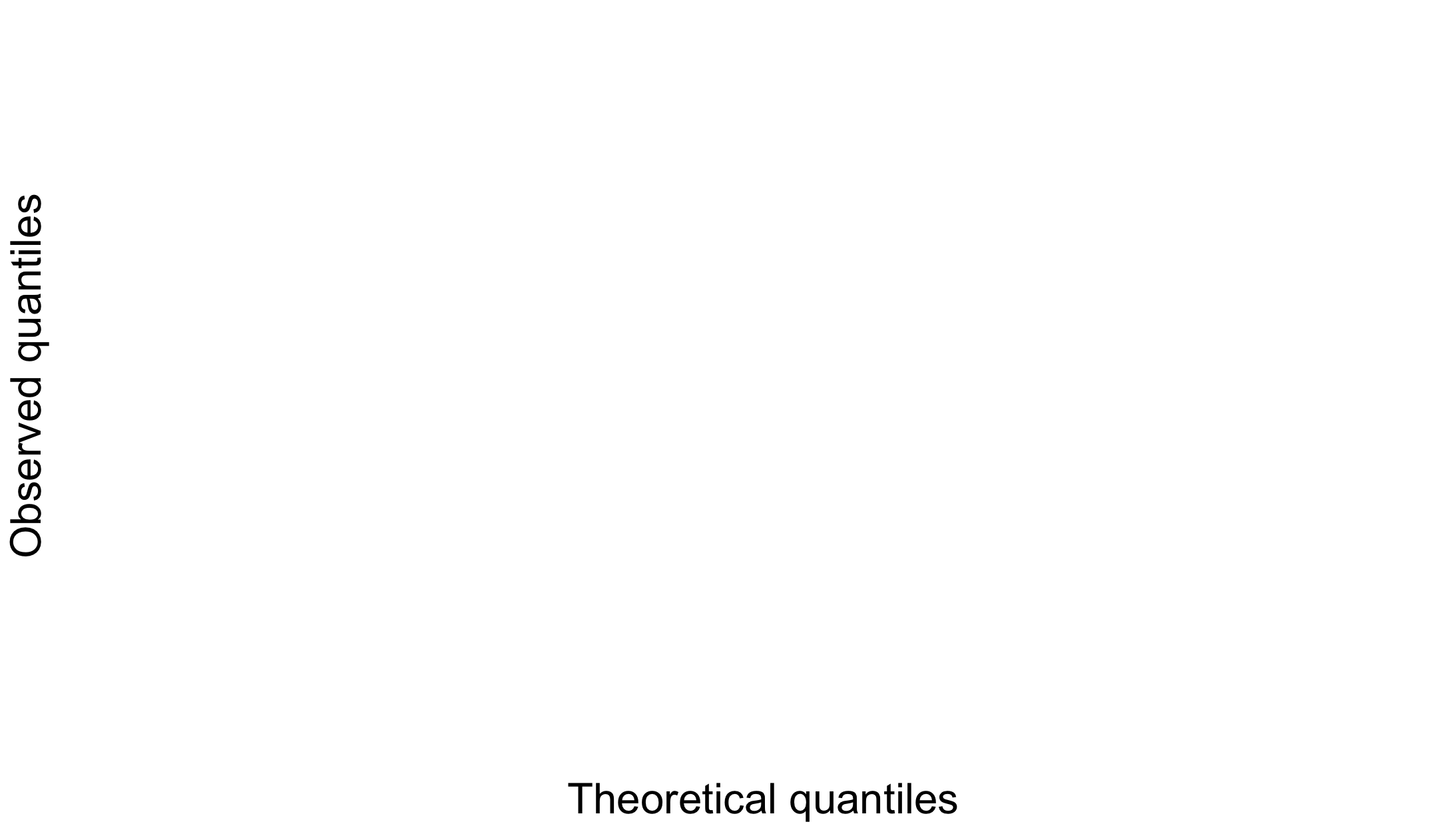

Supplement: sj-jasp-3-hpq-10.1177_13591053211059393 – Supplemental material for Individual factors in the relationship between stress and resilience in mental health psychology practitioners during the COVID-19 pandemic [file sj-jasp-3-hpq-10.1177_13591053211059393.jasp › resources/45/_1_t1603025150787.png]

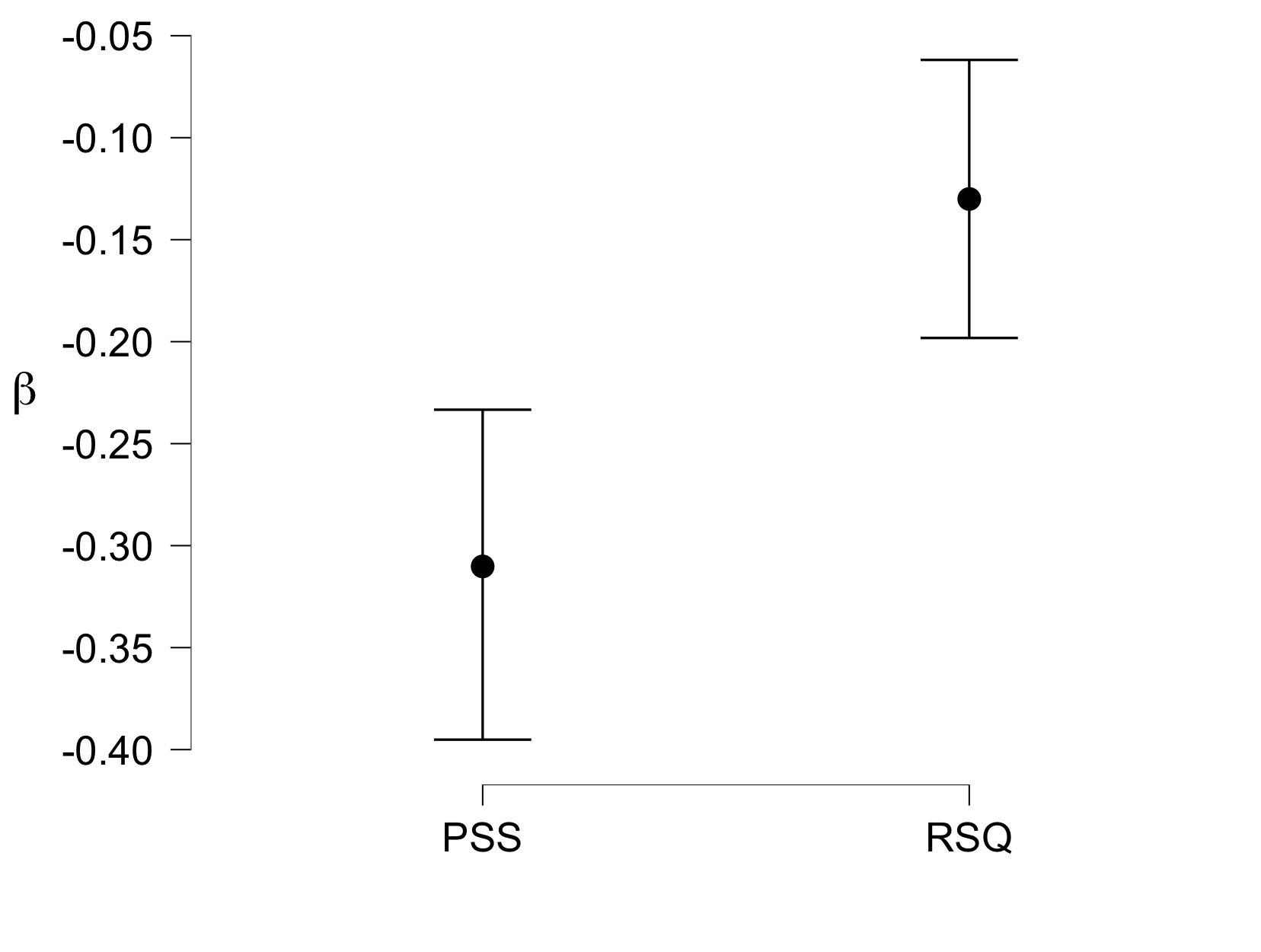

Supplement: sj-jasp-3-hpq-10.1177_13591053211059393 – Supplemental material for Individual factors in the relationship between stress and resilience in mental health psychology practitioners during the COVID-19 pandemic [file sj-jasp-3-hpq-10.1177_13591053211059393.jasp › resources/41/_2_t1603030925435.png]

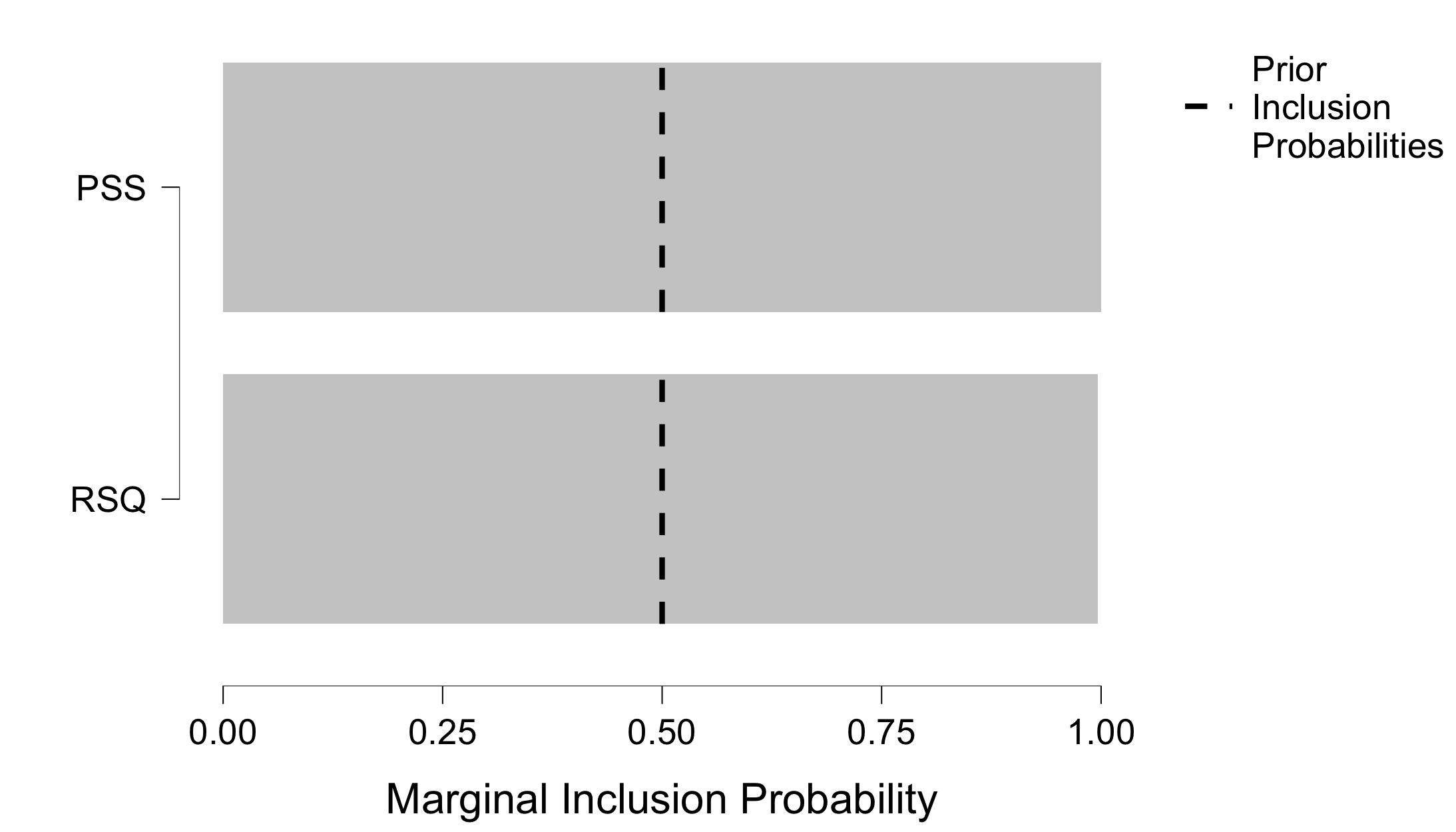

Supplement: sj-jasp-3-hpq-10.1177_13591053211059393 – Supplemental material for Individual factors in the relationship between stress and resilience in mental health psychology practitioners during the COVID-19 pandemic [file sj-jasp-3-hpq-10.1177_13591053211059393.jasp › resources/41/_3_t1603030929818.png]

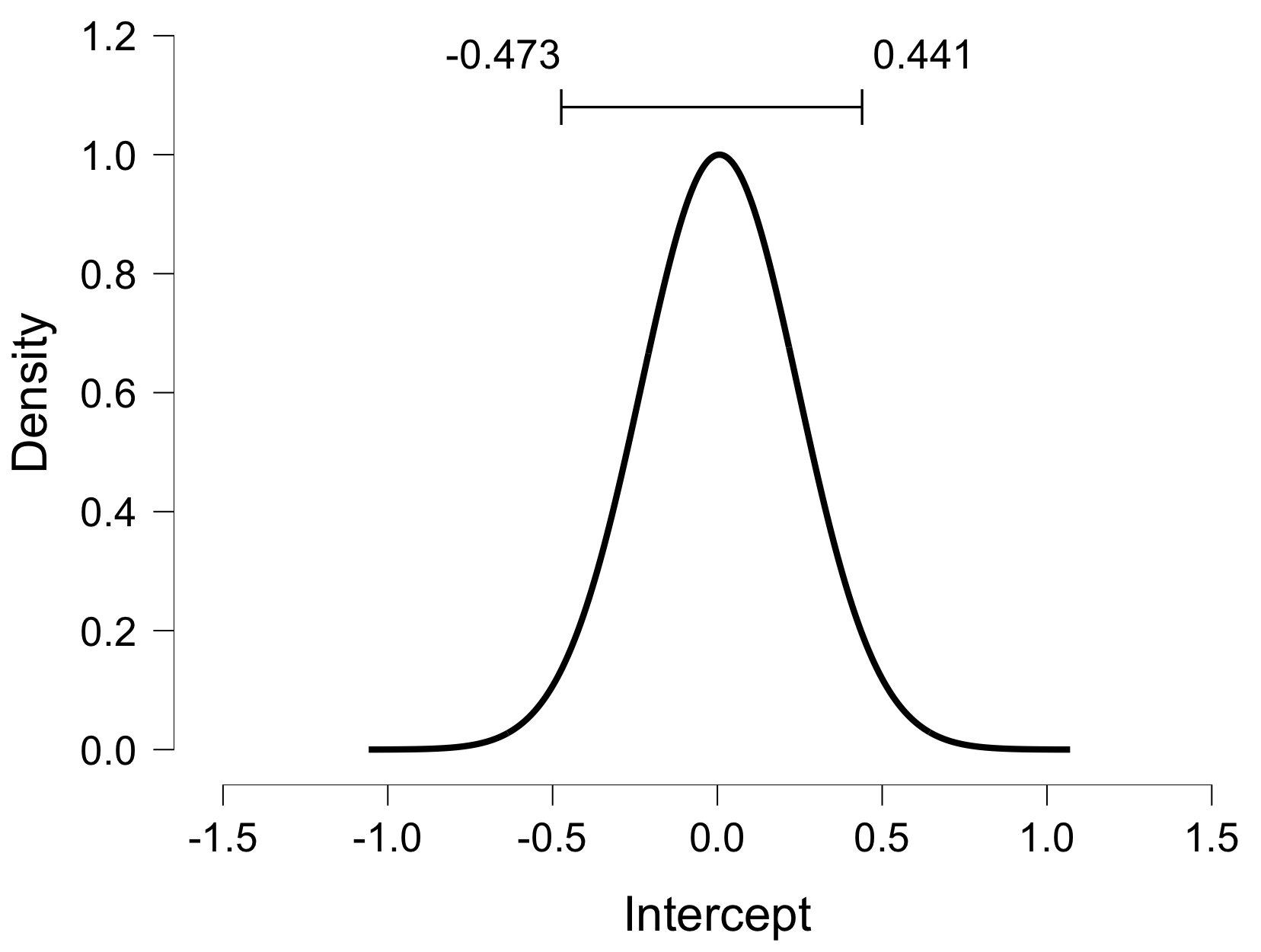

Supplement: sj-jasp-3-hpq-10.1177_13591053211059393 – Supplemental material for Individual factors in the relationship between stress and resilience in mental health psychology practitioners during the COVID-19 pandemic [file sj-jasp-3-hpq-10.1177_13591053211059393.jasp › resources/41/_1_t1603030939020.png]

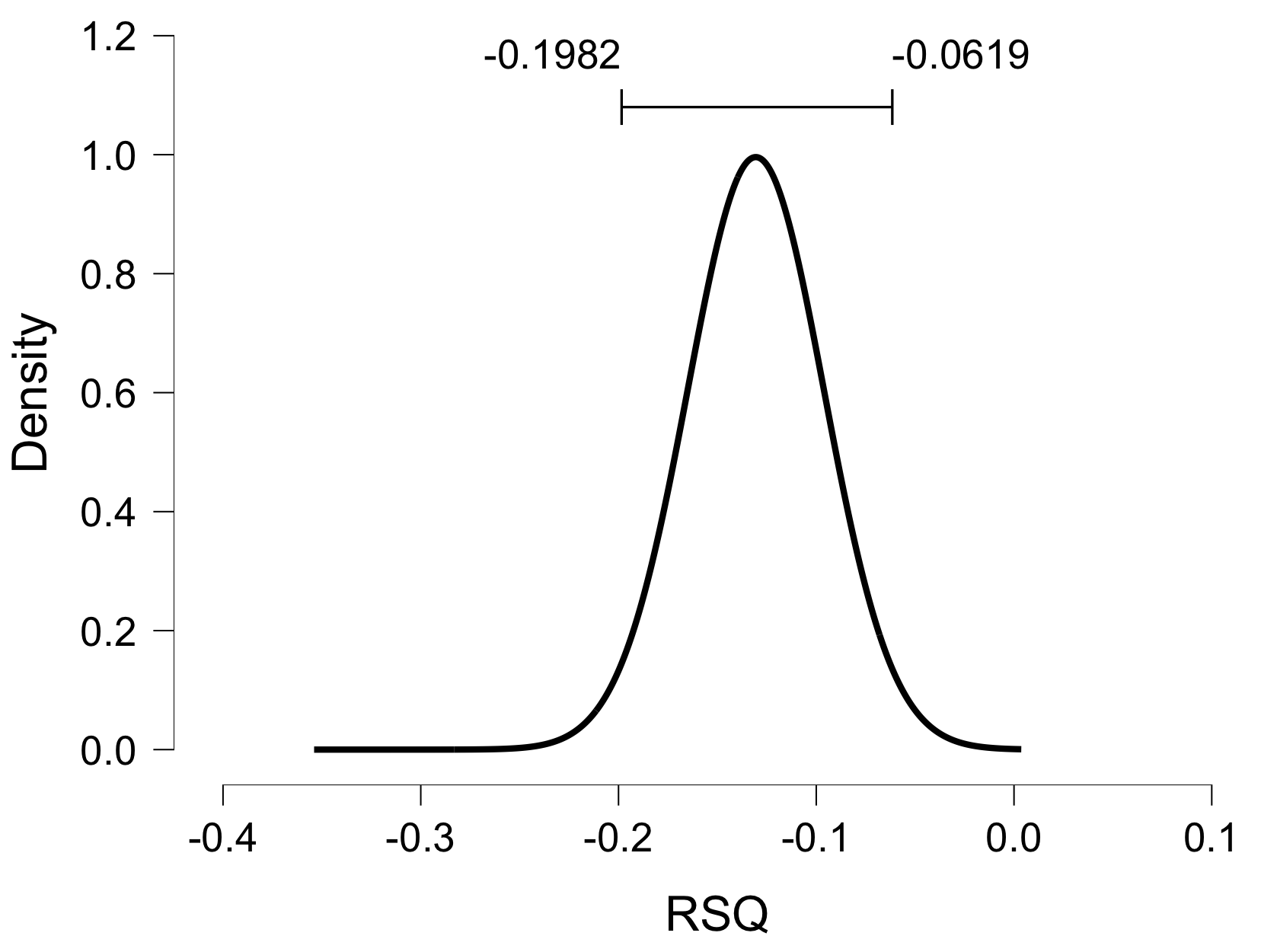

Supplement: sj-jasp-3-hpq-10.1177_13591053211059393 – Supplemental material for Individual factors in the relationship between stress and resilience in mental health psychology practitioners during the COVID-19 pandemic [file sj-jasp-3-hpq-10.1177_13591053211059393.jasp › resources/41/_3_t1603030939911.png]

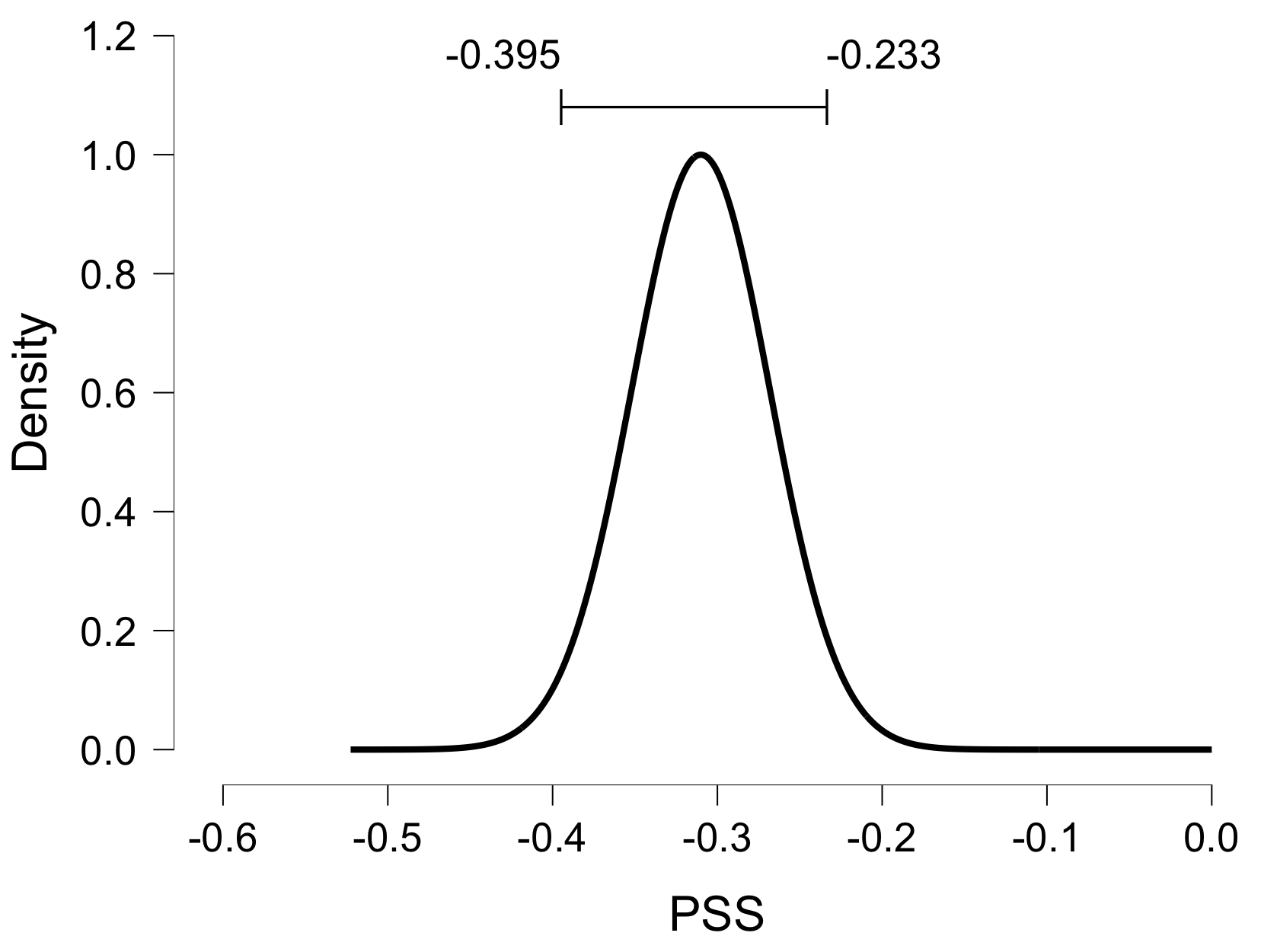

Supplement: sj-jasp-3-hpq-10.1177_13591053211059393 – Supplemental material for Individual factors in the relationship between stress and resilience in mental health psychology practitioners during the COVID-19 pandemic [file sj-jasp-3-hpq-10.1177_13591053211059393.jasp › resources/41/_2_t1603030939663.png]

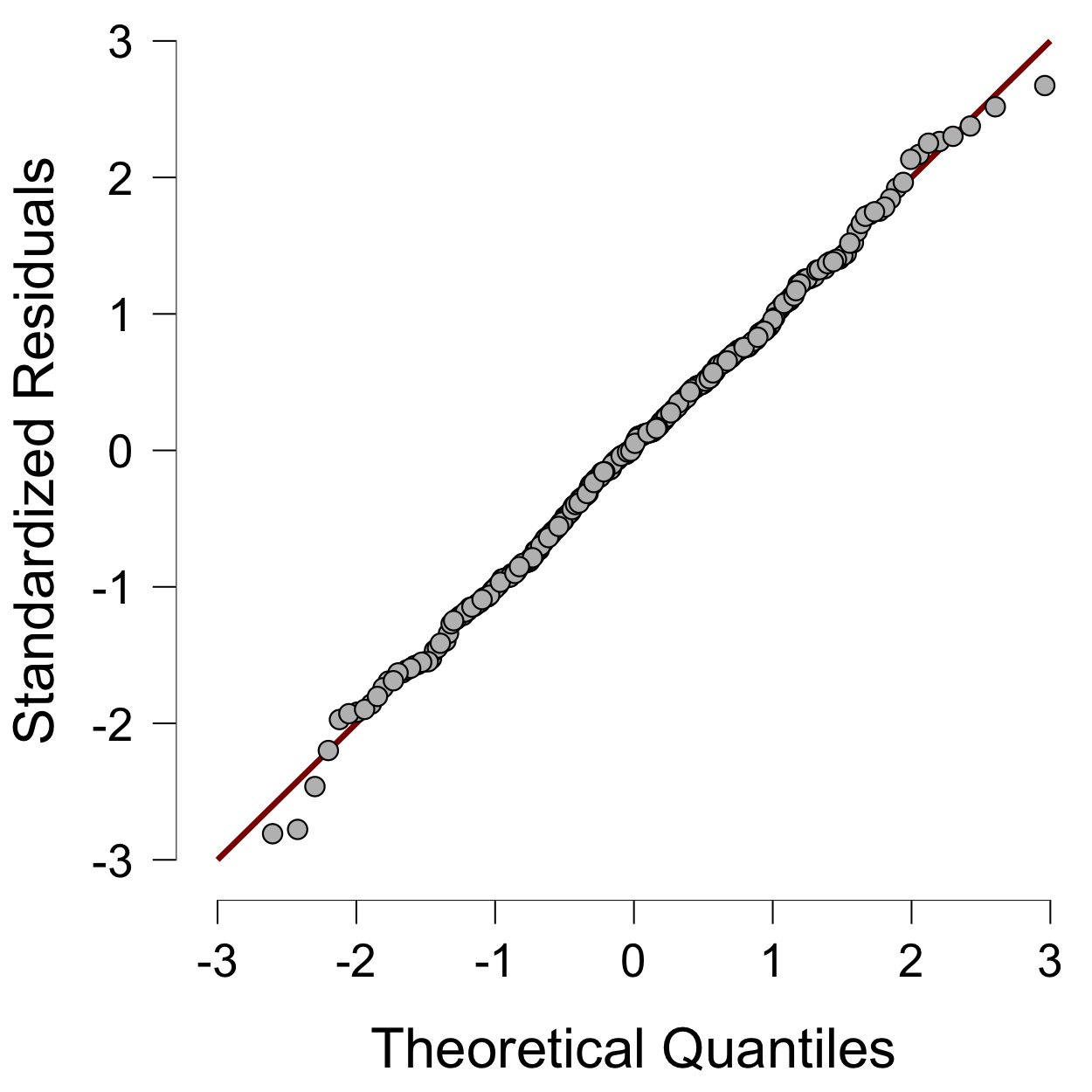

Supplement: sj-jasp-3-hpq-10.1177_13591053211059393 – Supplemental material for Individual factors in the relationship between stress and resilience in mental health psychology practitioners during the COVID-19 pandemic [file sj-jasp-3-hpq-10.1177_13591053211059393.jasp › resources/46/_6_t1603025257998.png]

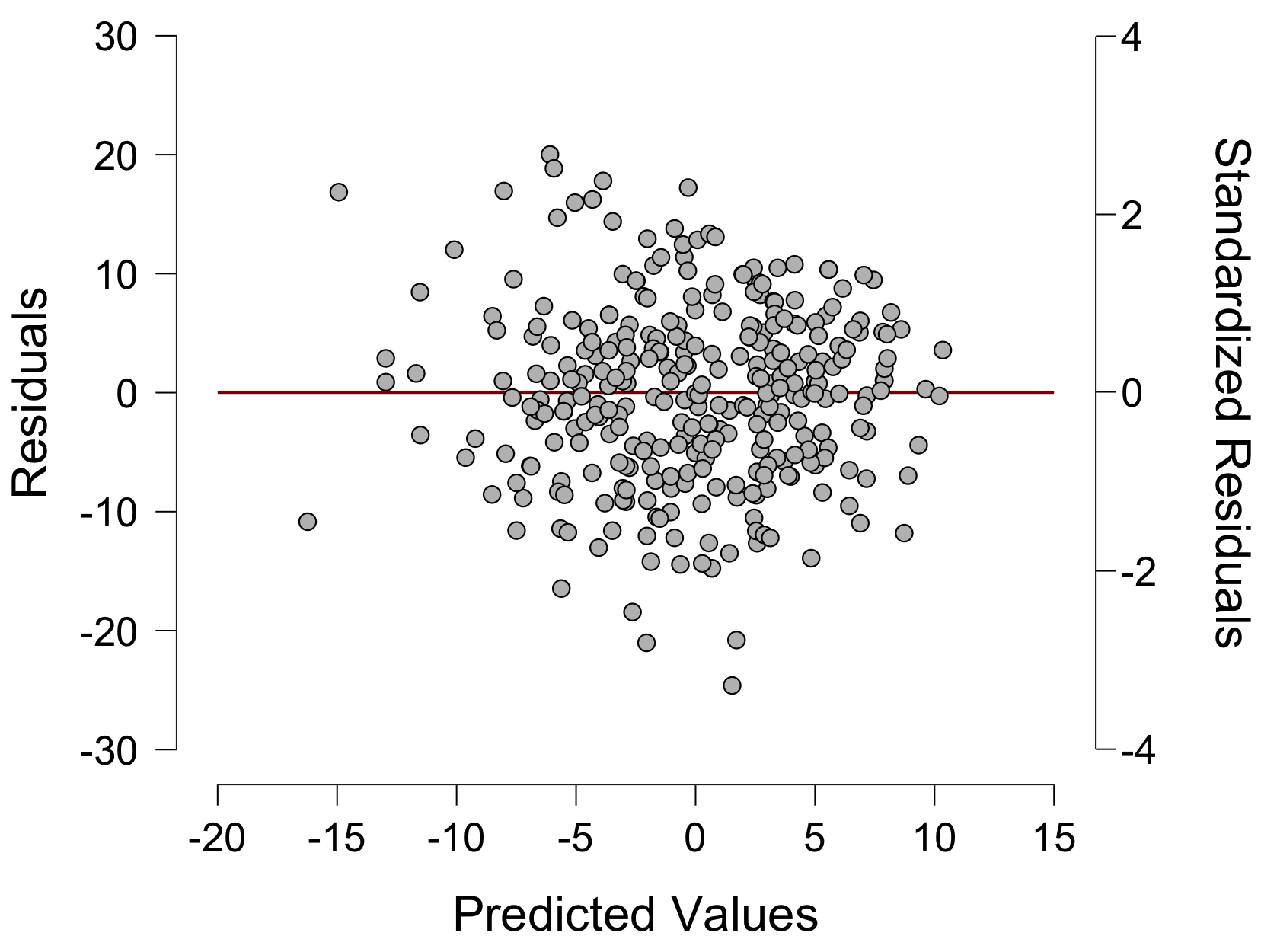

Supplement: sj-jasp-3-hpq-10.1177_13591053211059393 – Supplemental material for Individual factors in the relationship between stress and resilience in mental health psychology practitioners during the COVID-19 pandemic [file sj-jasp-3-hpq-10.1177_13591053211059393.jasp › resources/46/_5_t1603025257062.png]

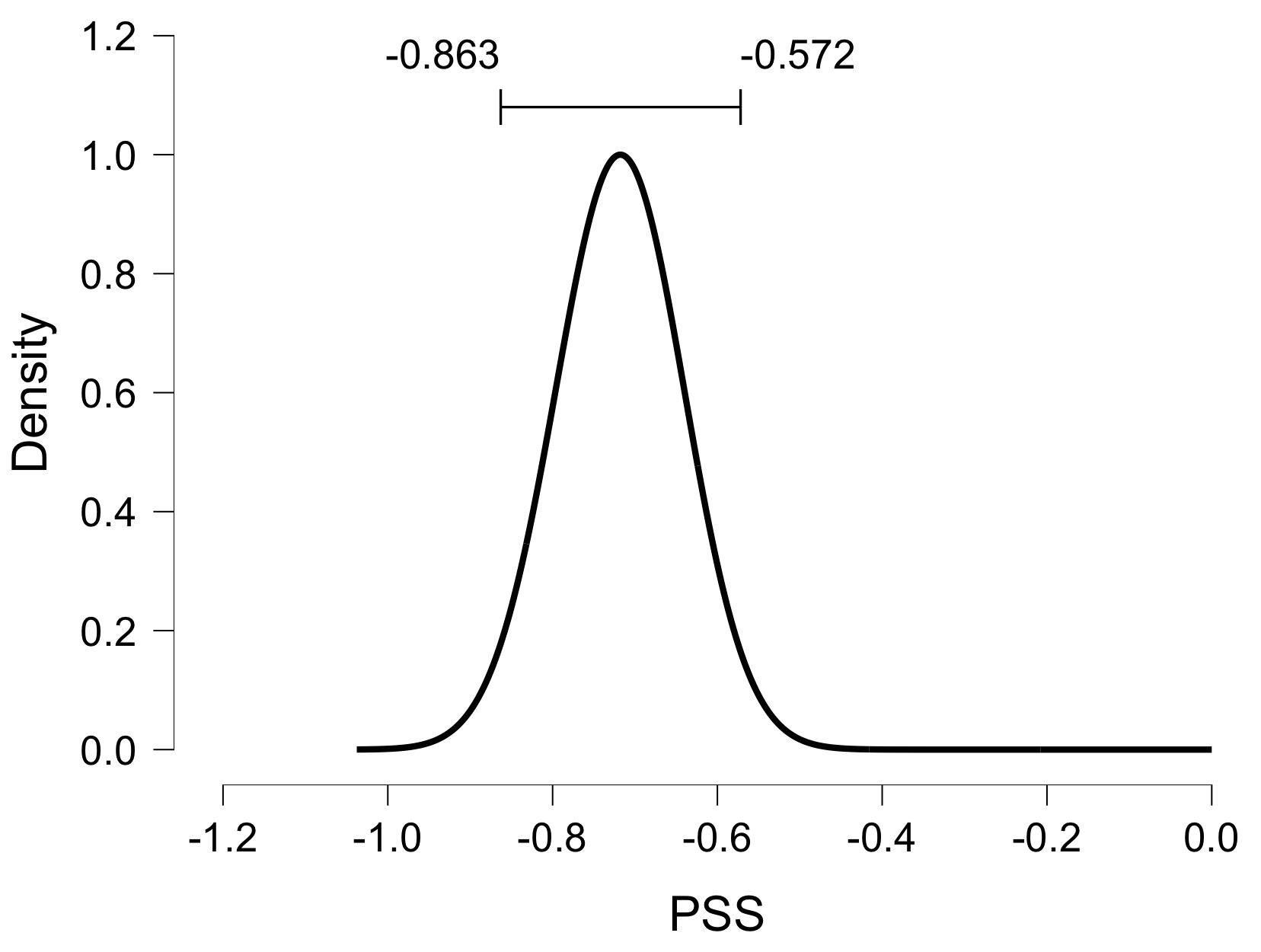

Supplement: sj-jasp-3-hpq-10.1177_13591053211059393 – Supplemental material for Individual factors in the relationship between stress and resilience in mental health psychology practitioners during the COVID-19 pandemic [file sj-jasp-3-hpq-10.1177_13591053211059393.jasp › resources/47/_2_t1603025313903.png]

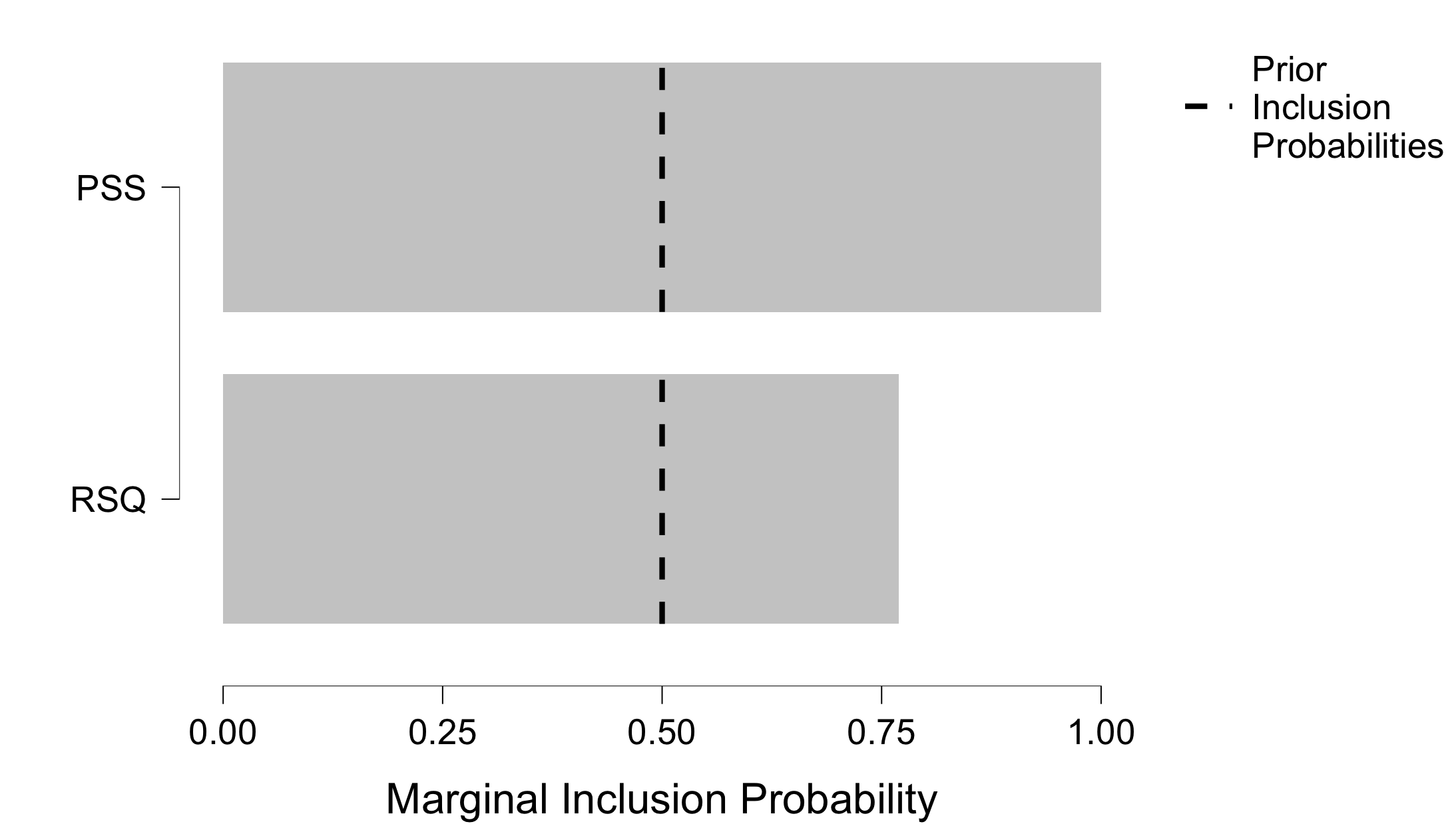

Supplement: sj-jasp-3-hpq-10.1177_13591053211059393 – Supplemental material for Individual factors in the relationship between stress and resilience in mental health psychology practitioners during the COVID-19 pandemic [file sj-jasp-3-hpq-10.1177_13591053211059393.jasp › resources/47/_8_t1603025306085.png]

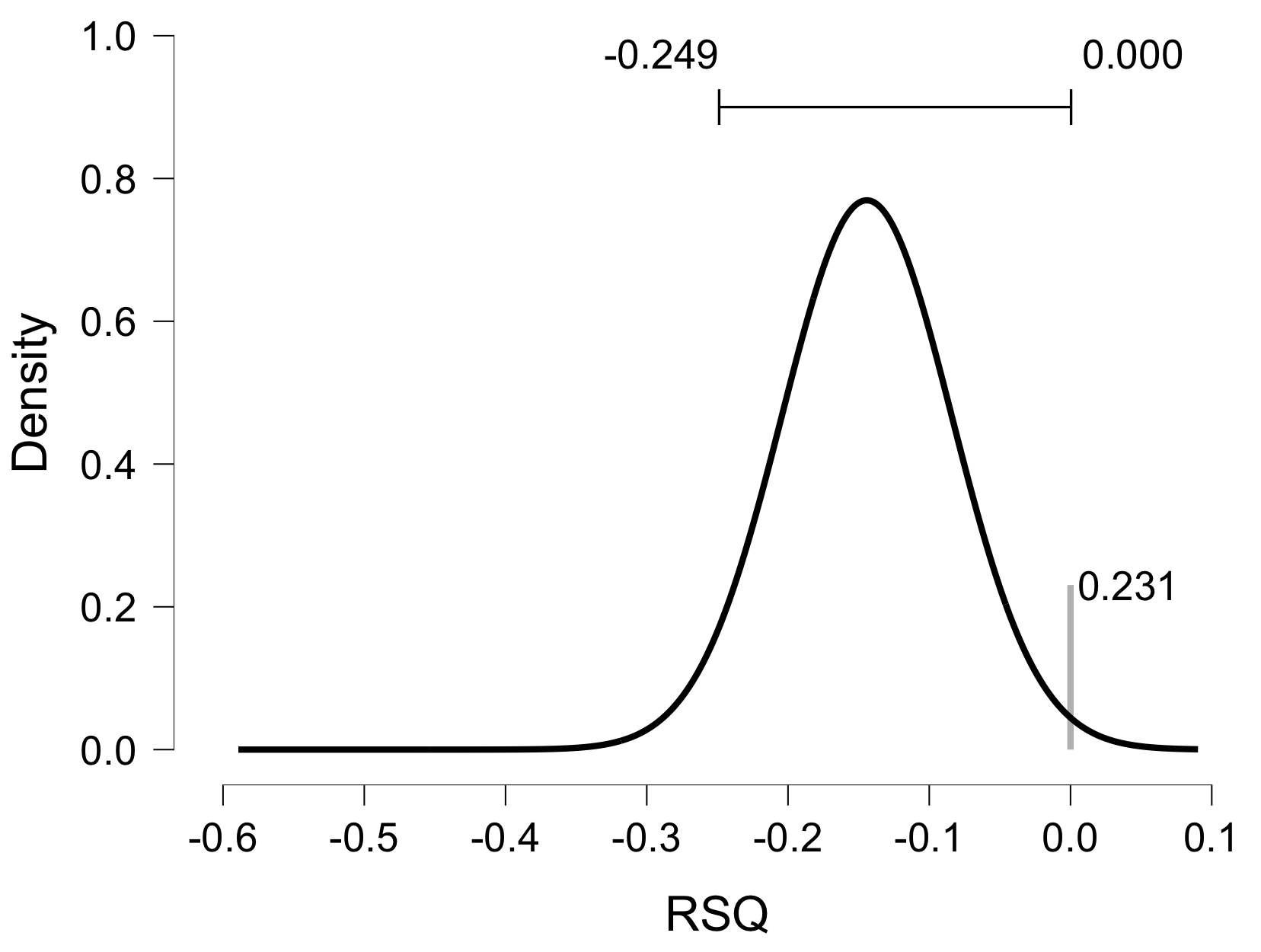

Supplement: sj-jasp-3-hpq-10.1177_13591053211059393 – Supplemental material for Individual factors in the relationship between stress and resilience in mental health psychology practitioners during the COVID-19 pandemic [file sj-jasp-3-hpq-10.1177_13591053211059393.jasp › resources/47/_3_t1603025314161.png]

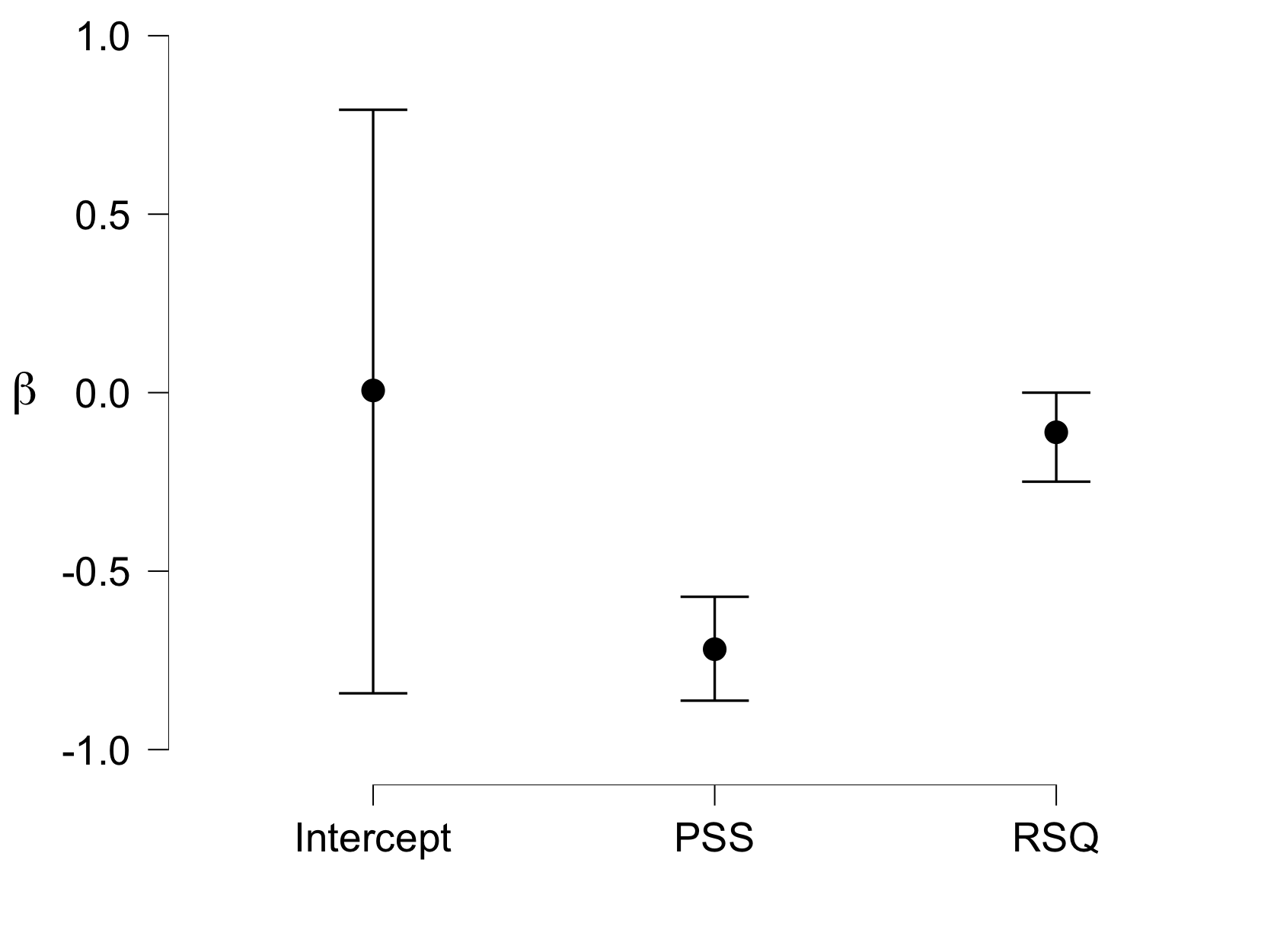

Supplement: sj-jasp-3-hpq-10.1177_13591053211059393 – Supplemental material for Individual factors in the relationship between stress and resilience in mental health psychology practitioners during the COVID-19 pandemic [file sj-jasp-3-hpq-10.1177_13591053211059393.jasp › resources/47/_7_t1603025299842.png]

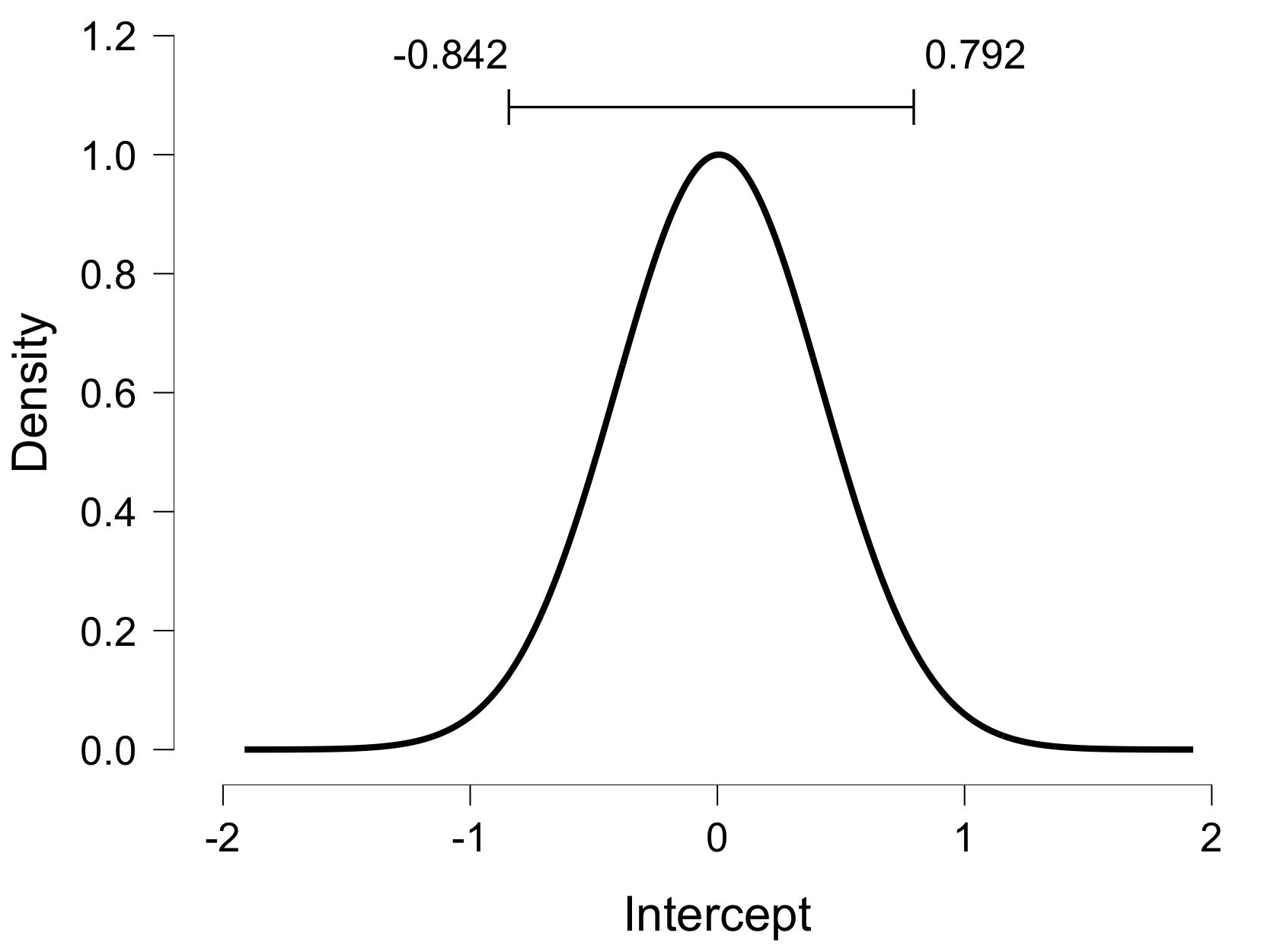

Supplement: sj-jasp-3-hpq-10.1177_13591053211059393 – Supplemental material for Individual factors in the relationship between stress and resilience in mental health psychology practitioners during the COVID-19 pandemic [file sj-jasp-3-hpq-10.1177_13591053211059393.jasp › resources/47/_1_t1603025313272.png]

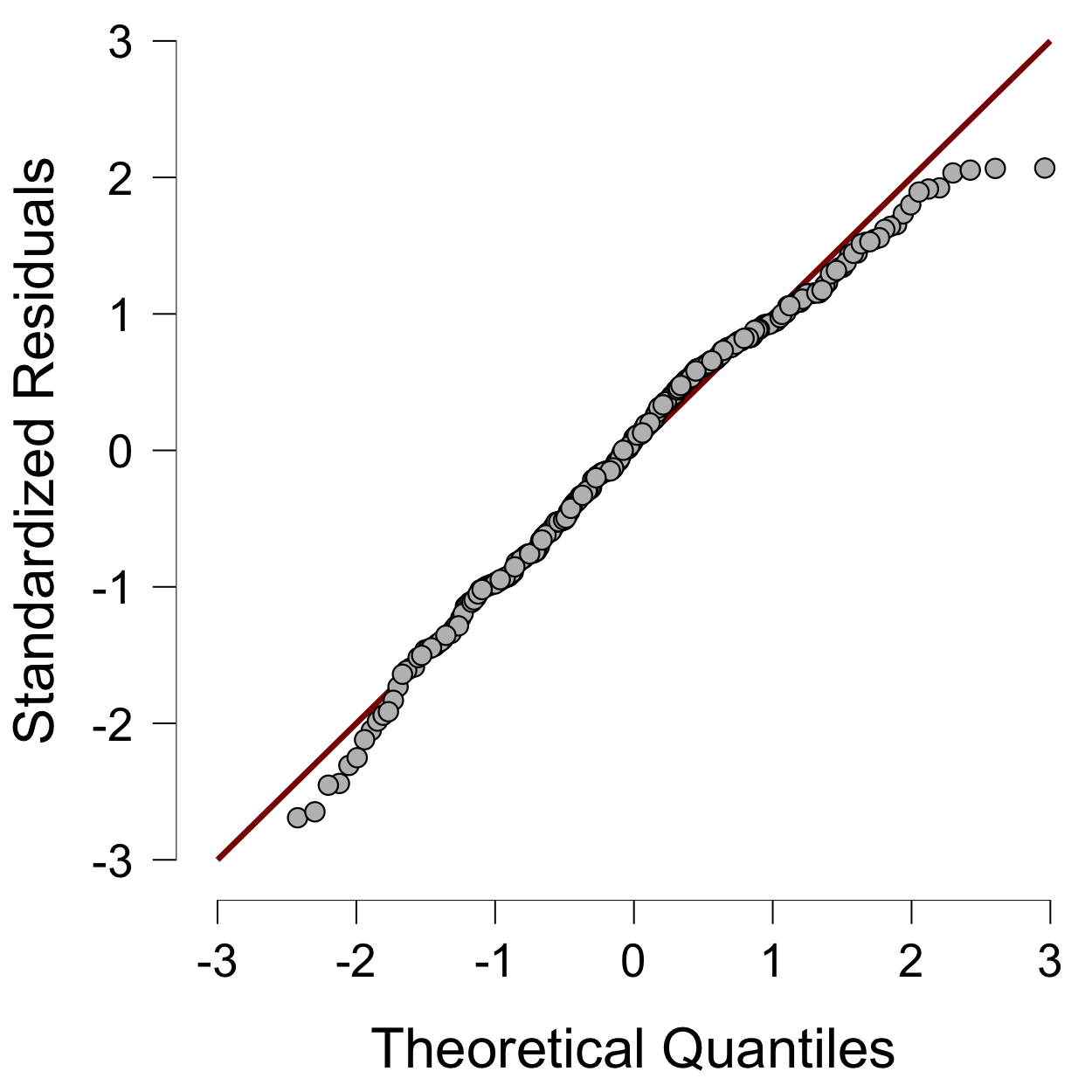

Supplement: sj-jasp-3-hpq-10.1177_13591053211059393 – Supplemental material for Individual factors in the relationship between stress and resilience in mental health psychology practitioners during the COVID-19 pandemic [file sj-jasp-3-hpq-10.1177_13591053211059393.jasp › resources/48/_1_t1603025496684.png]

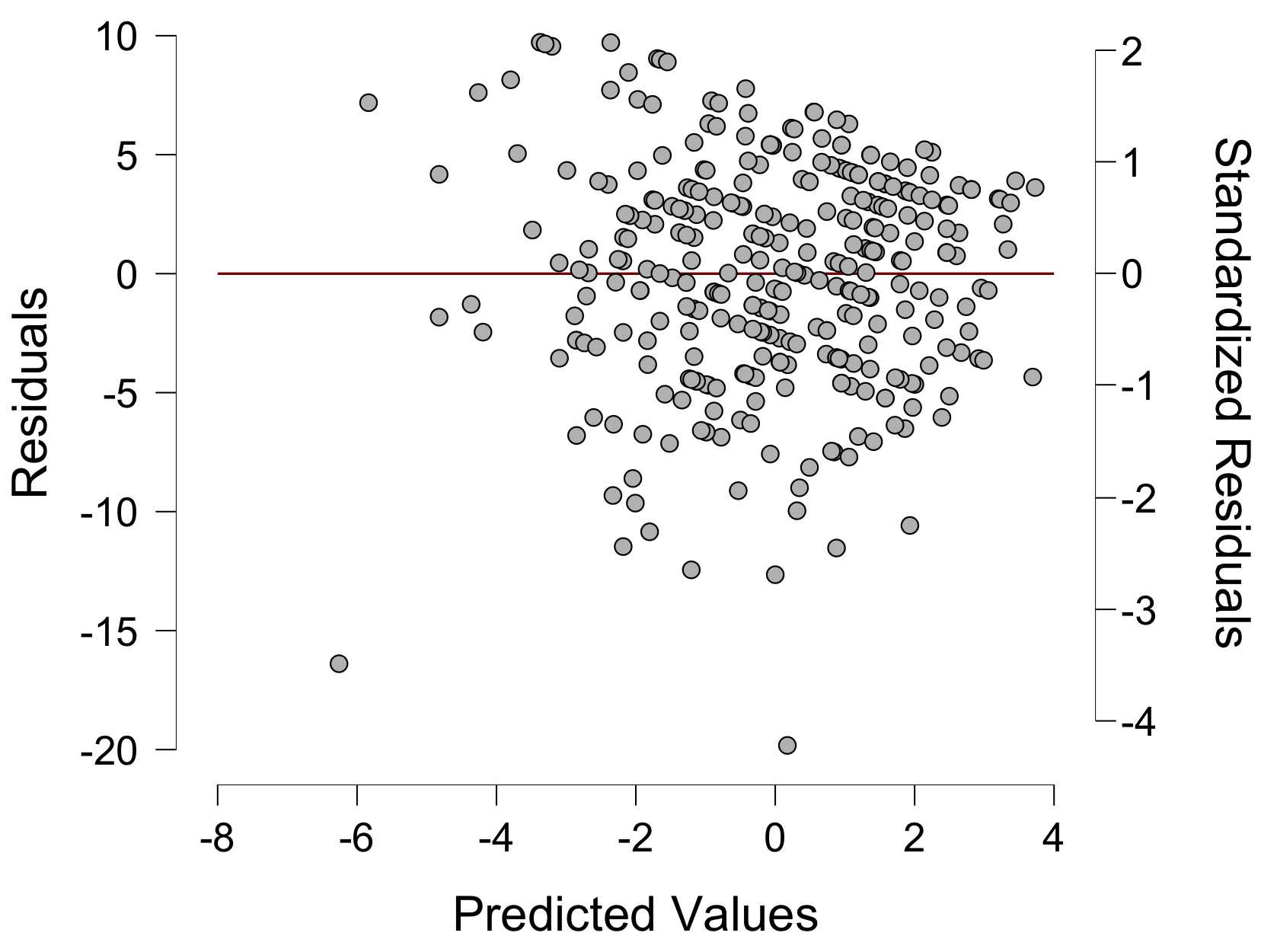

Supplement: sj-jasp-3-hpq-10.1177_13591053211059393 – Supplemental material for Individual factors in the relationship between stress and resilience in mental health psychology practitioners during the COVID-19 pandemic [file sj-jasp-3-hpq-10.1177_13591053211059393.jasp › resources/48/_0_t1603025495778.png]

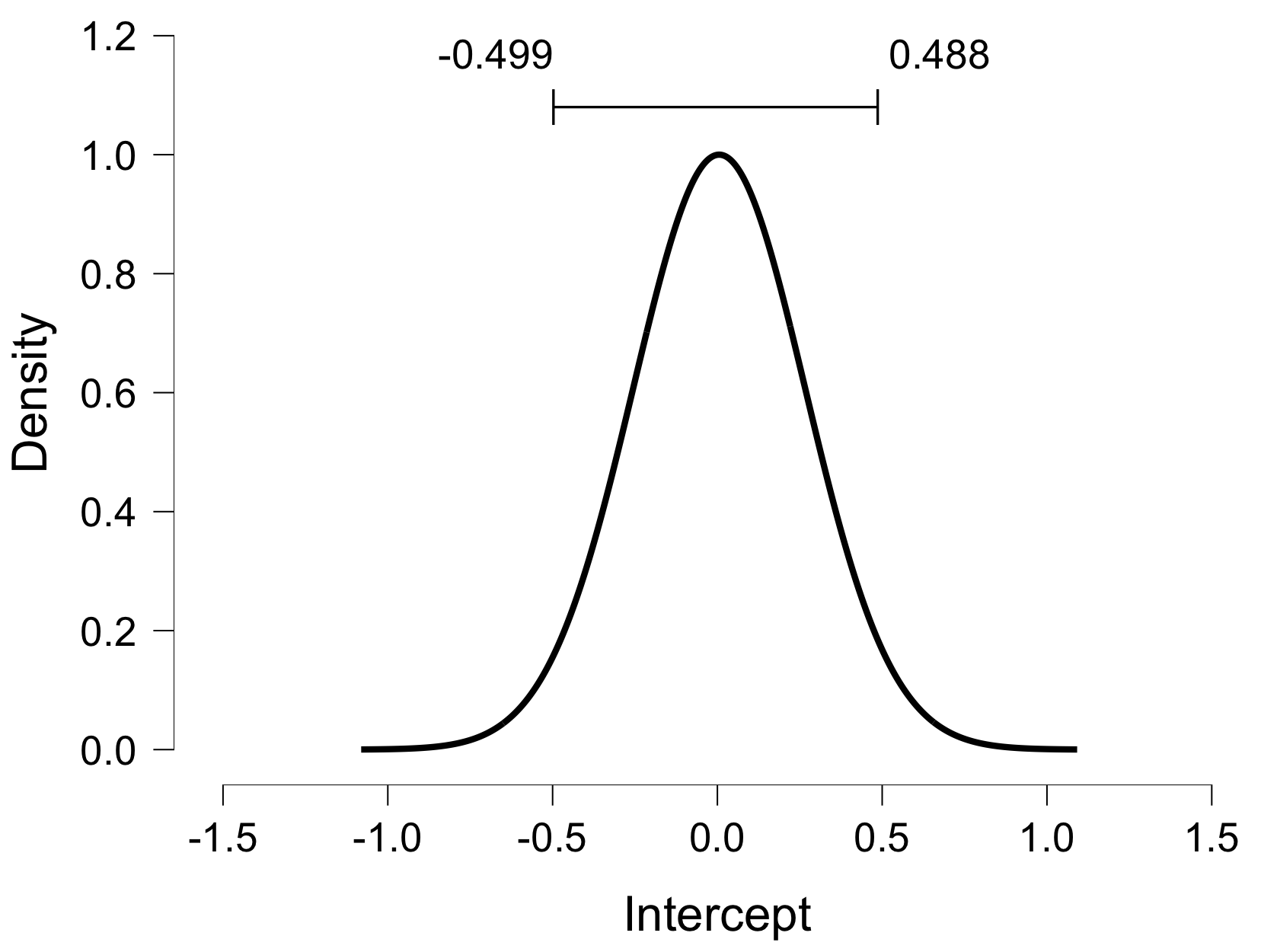

Supplement: sj-jasp-3-hpq-10.1177_13591053211059393 – Supplemental material for Individual factors in the relationship between stress and resilience in mental health psychology practitioners during the COVID-19 pandemic [file sj-jasp-3-hpq-10.1177_13591053211059393.jasp › resources/49/_1_t1603025587534.png]

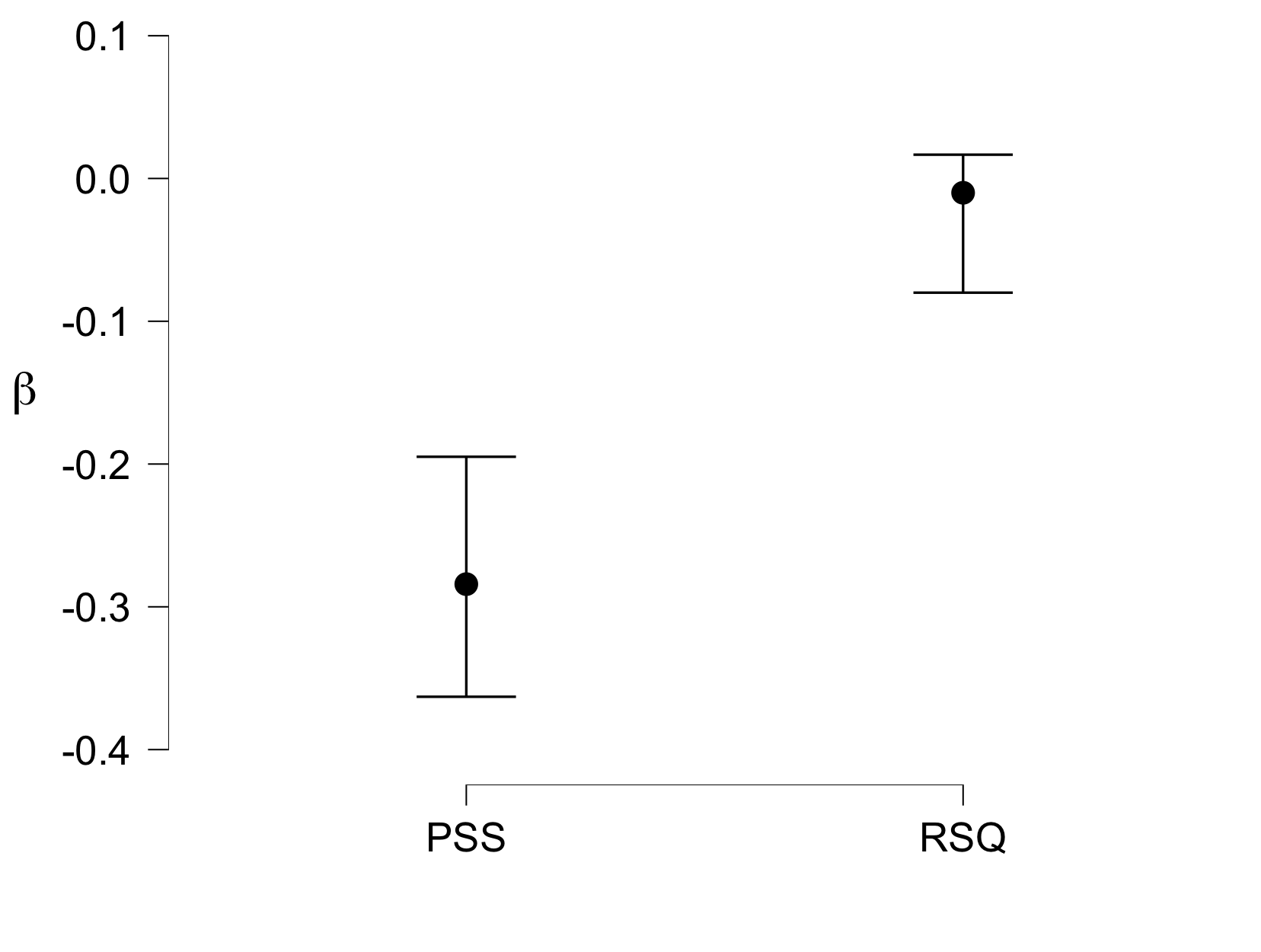

Supplement: sj-jasp-3-hpq-10.1177_13591053211059393 – Supplemental material for Individual factors in the relationship between stress and resilience in mental health psychology practitioners during the COVID-19 pandemic [file sj-jasp-3-hpq-10.1177_13591053211059393.jasp › resources/49/_4_t1603025597592.png]

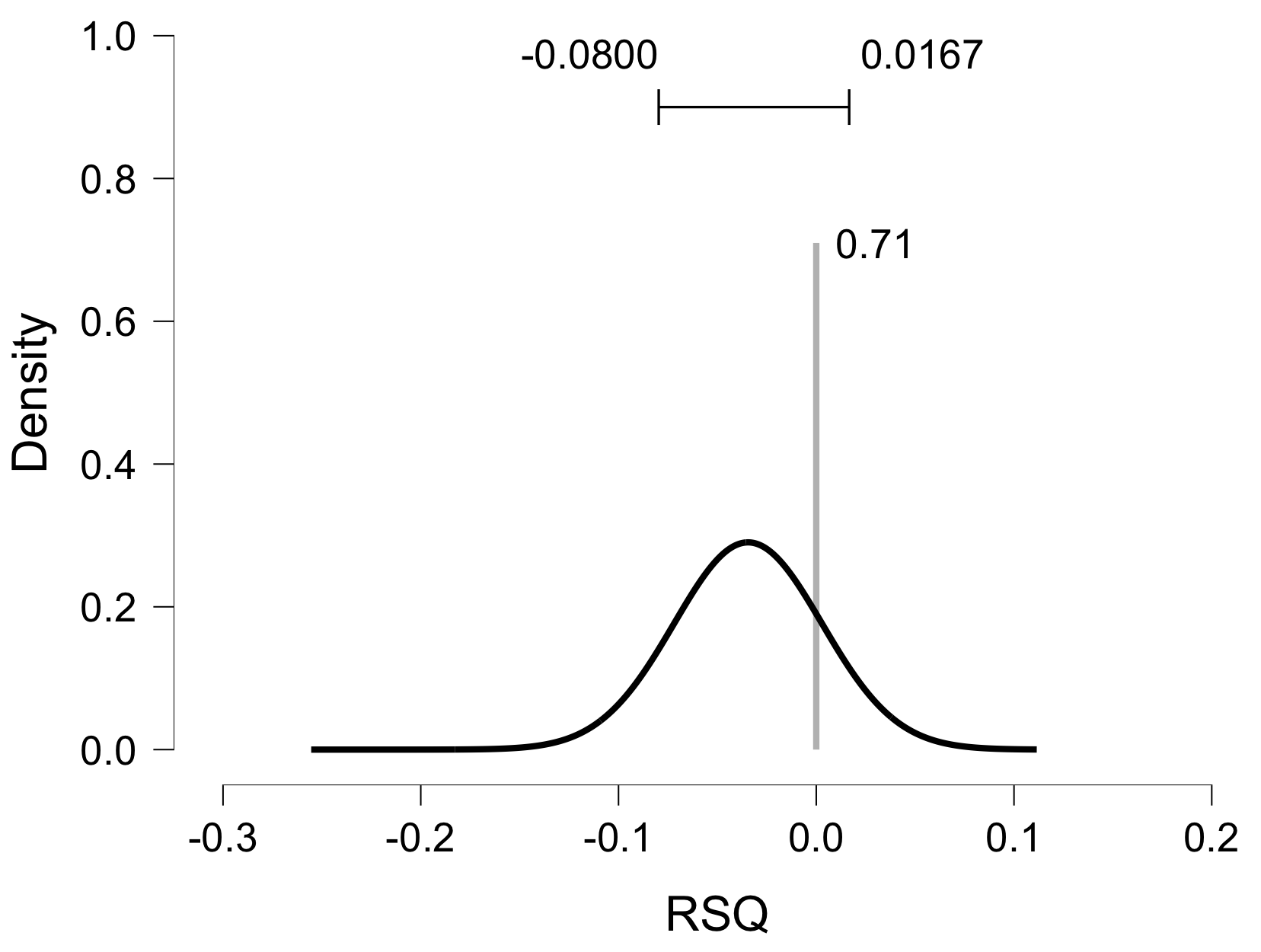

Supplement: sj-jasp-3-hpq-10.1177_13591053211059393 – Supplemental material for Individual factors in the relationship between stress and resilience in mental health psychology practitioners during the COVID-19 pandemic [file sj-jasp-3-hpq-10.1177_13591053211059393.jasp › resources/49/_3_t1603025588066.png]

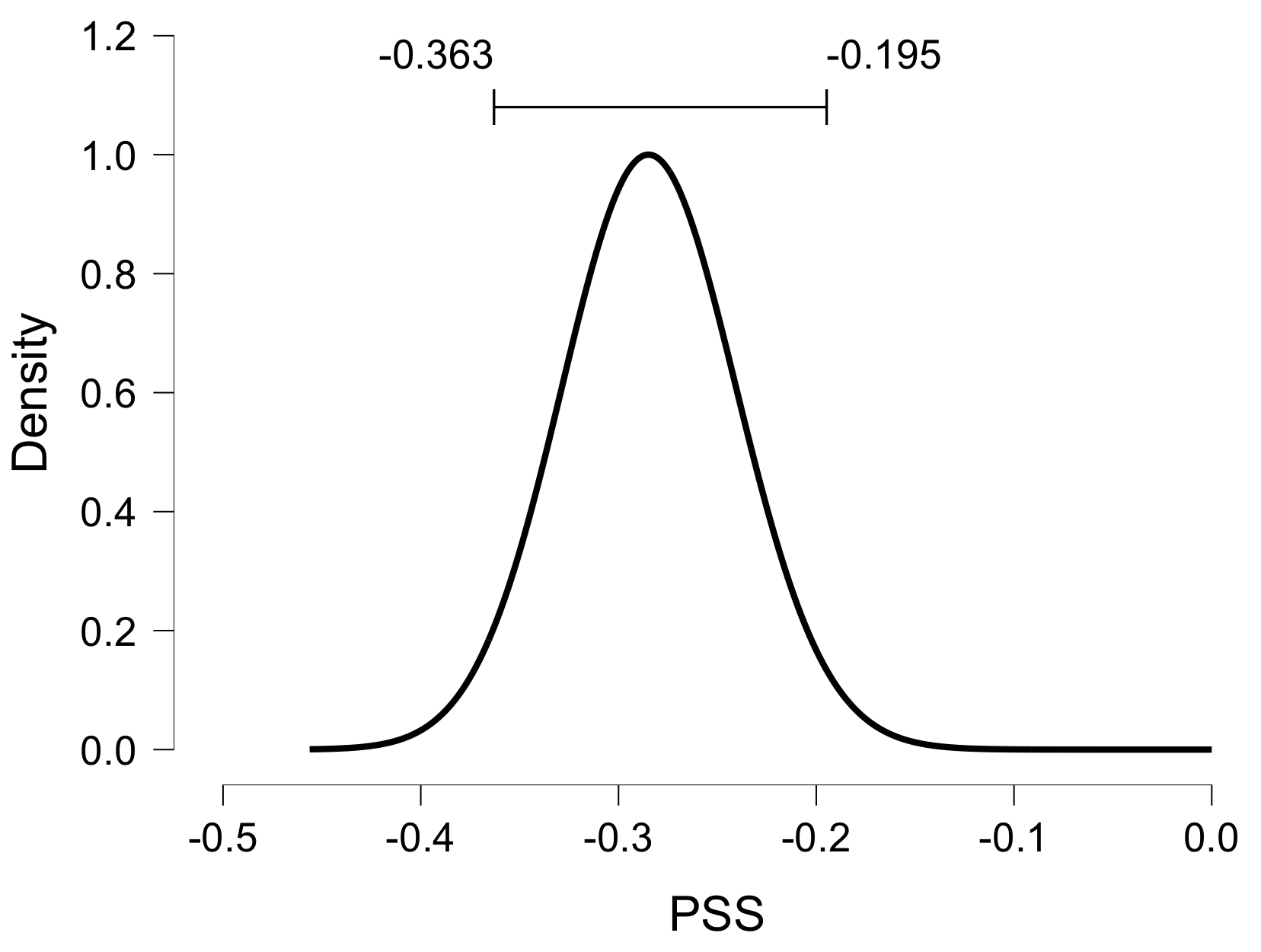

Supplement: sj-jasp-3-hpq-10.1177_13591053211059393 – Supplemental material for Individual factors in the relationship between stress and resilience in mental health psychology practitioners during the COVID-19 pandemic [file sj-jasp-3-hpq-10.1177_13591053211059393.jasp › resources/49/_2_t1603025587844.png]

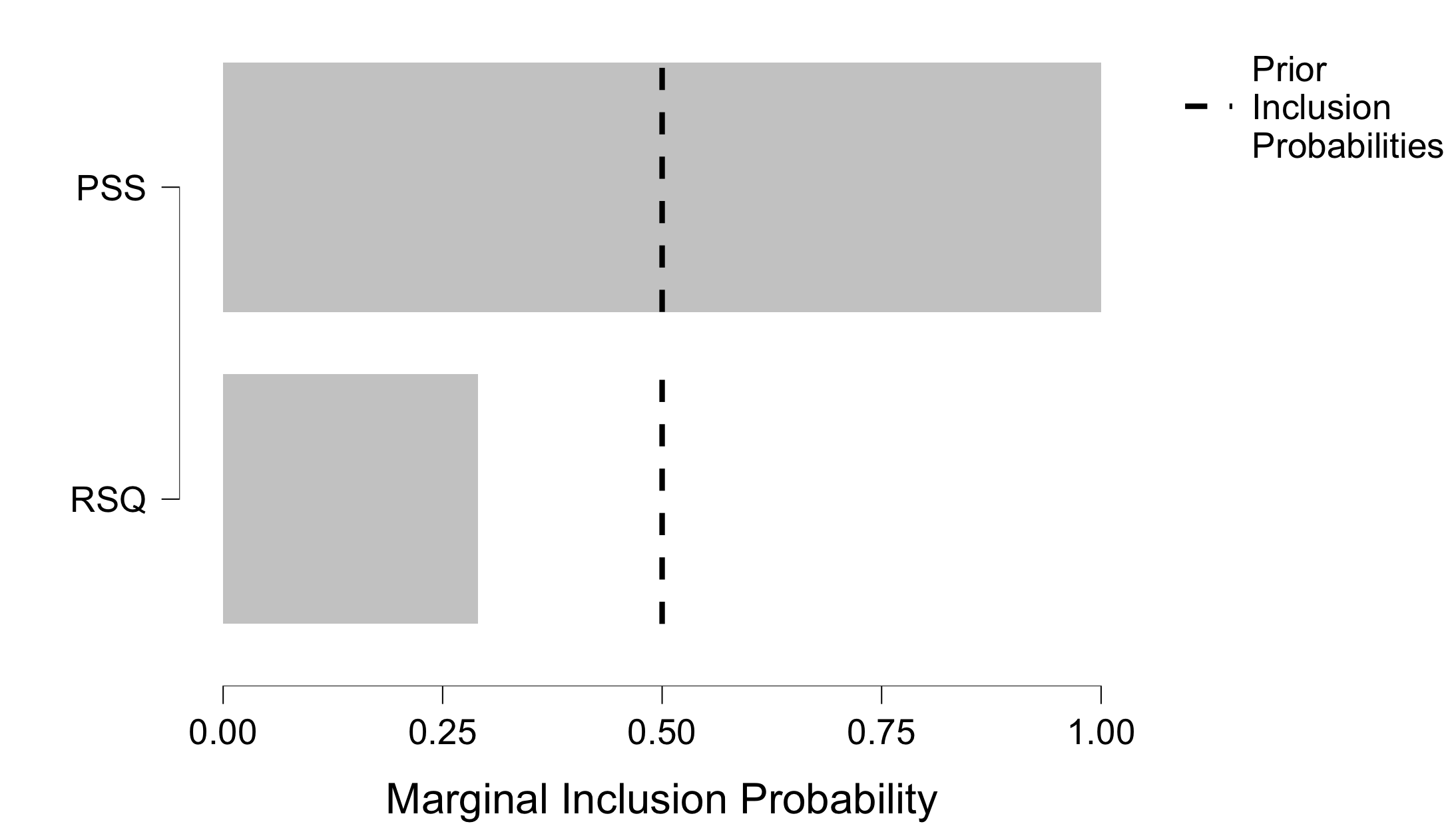

Supplement: sj-jasp-3-hpq-10.1177_13591053211059393 – Supplemental material for Individual factors in the relationship between stress and resilience in mental health psychology practitioners during the COVID-19 pandemic [file sj-jasp-3-hpq-10.1177_13591053211059393.jasp › resources/49/_3_t1603025580886.png]

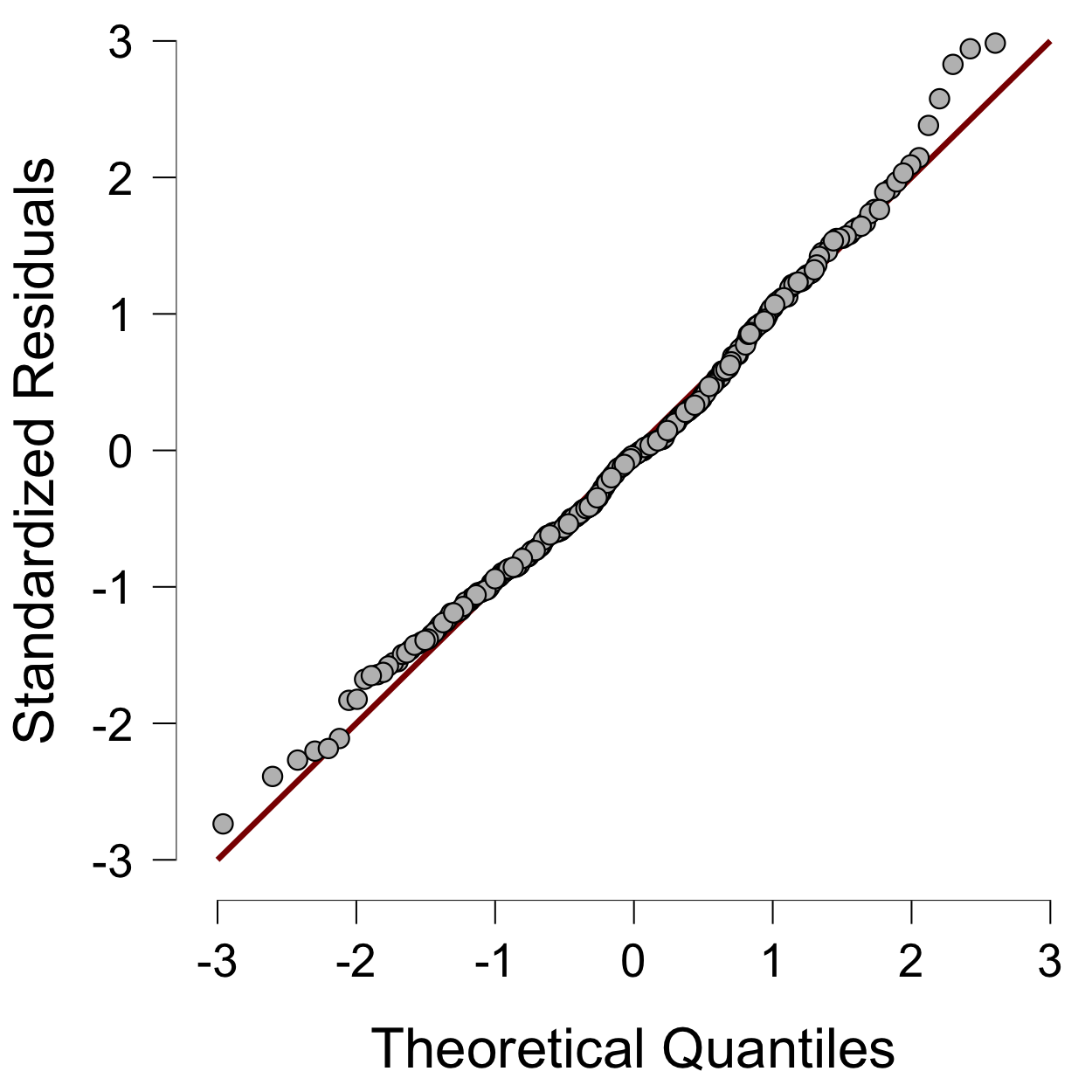

Supplement: sj-jasp-3-hpq-10.1177_13591053211059393 – Supplemental material for Individual factors in the relationship between stress and resilience in mental health psychology practitioners during the COVID-19 pandemic [file sj-jasp-3-hpq-10.1177_13591053211059393.jasp › resources/50/_6_t1603025714037.png]

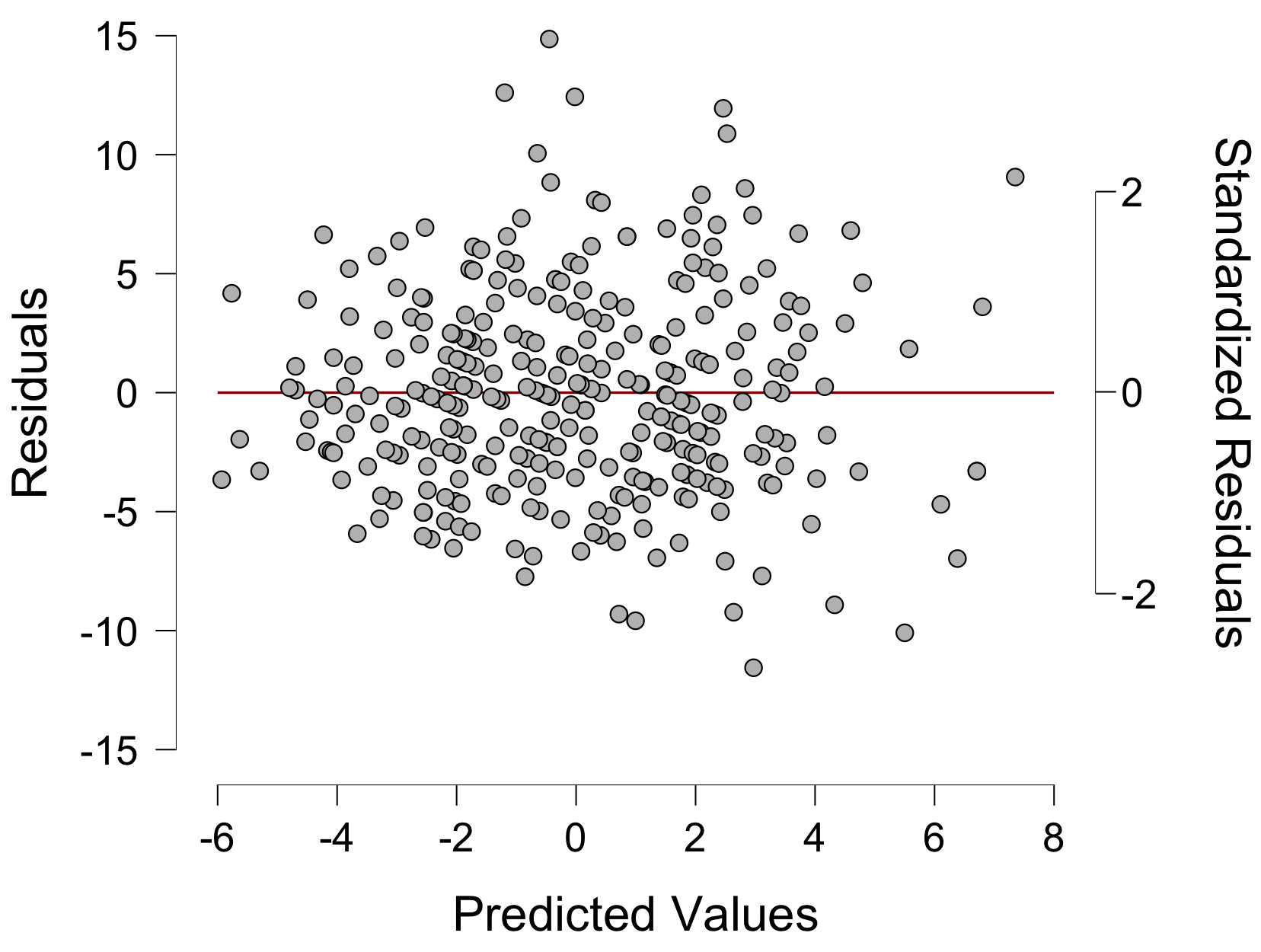

Supplement: sj-jasp-3-hpq-10.1177_13591053211059393 – Supplemental material for Individual factors in the relationship between stress and resilience in mental health psychology practitioners during the COVID-19 pandemic [file sj-jasp-3-hpq-10.1177_13591053211059393.jasp › resources/50/_5_t1603025712693.png]

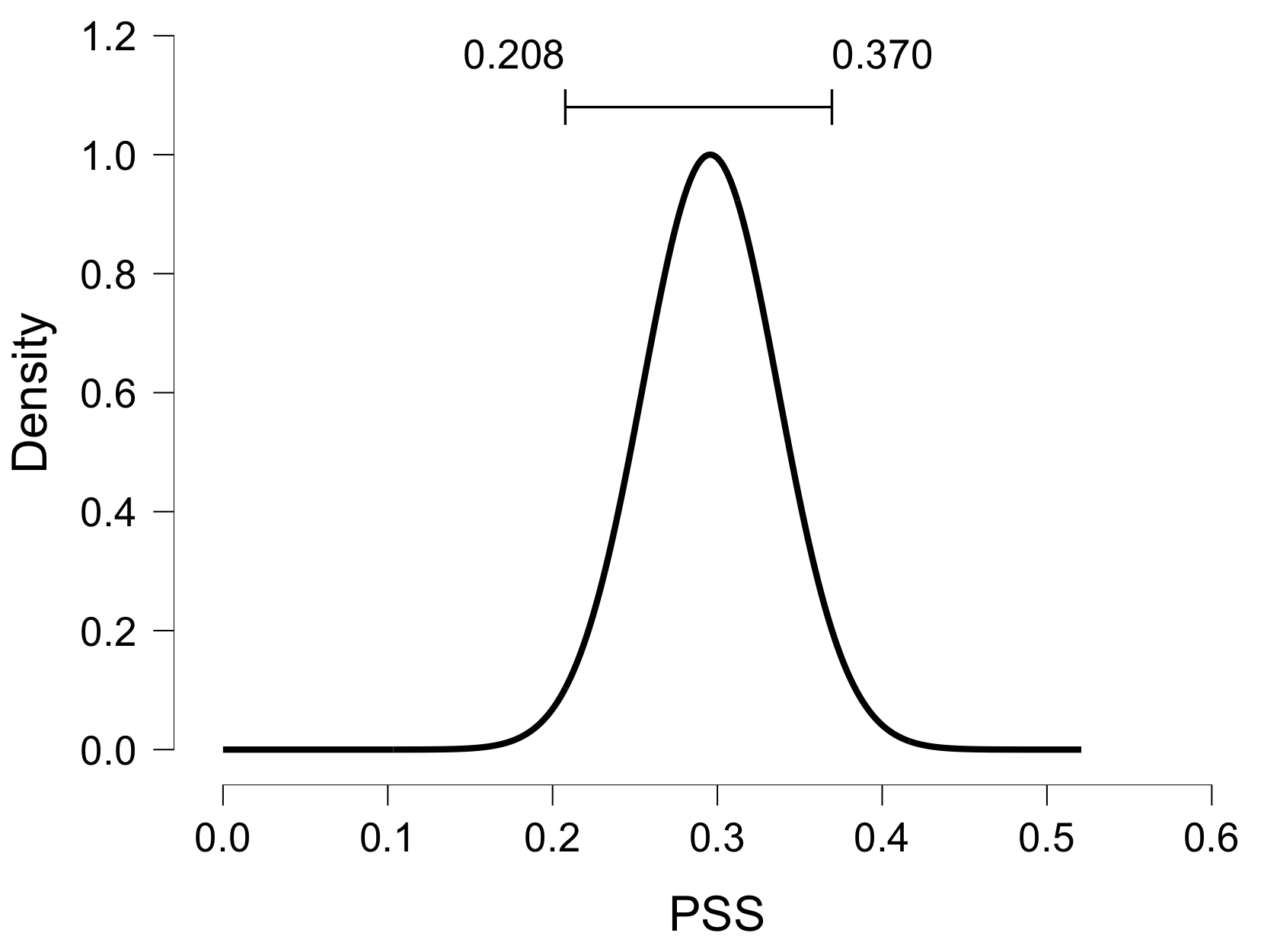

Supplement: sj-jasp-3-hpq-10.1177_13591053211059393 – Supplemental material for Individual factors in the relationship between stress and resilience in mental health psychology practitioners during the COVID-19 pandemic [file sj-jasp-3-hpq-10.1177_13591053211059393.jasp › resources/51/_2_t1603025773030.png]

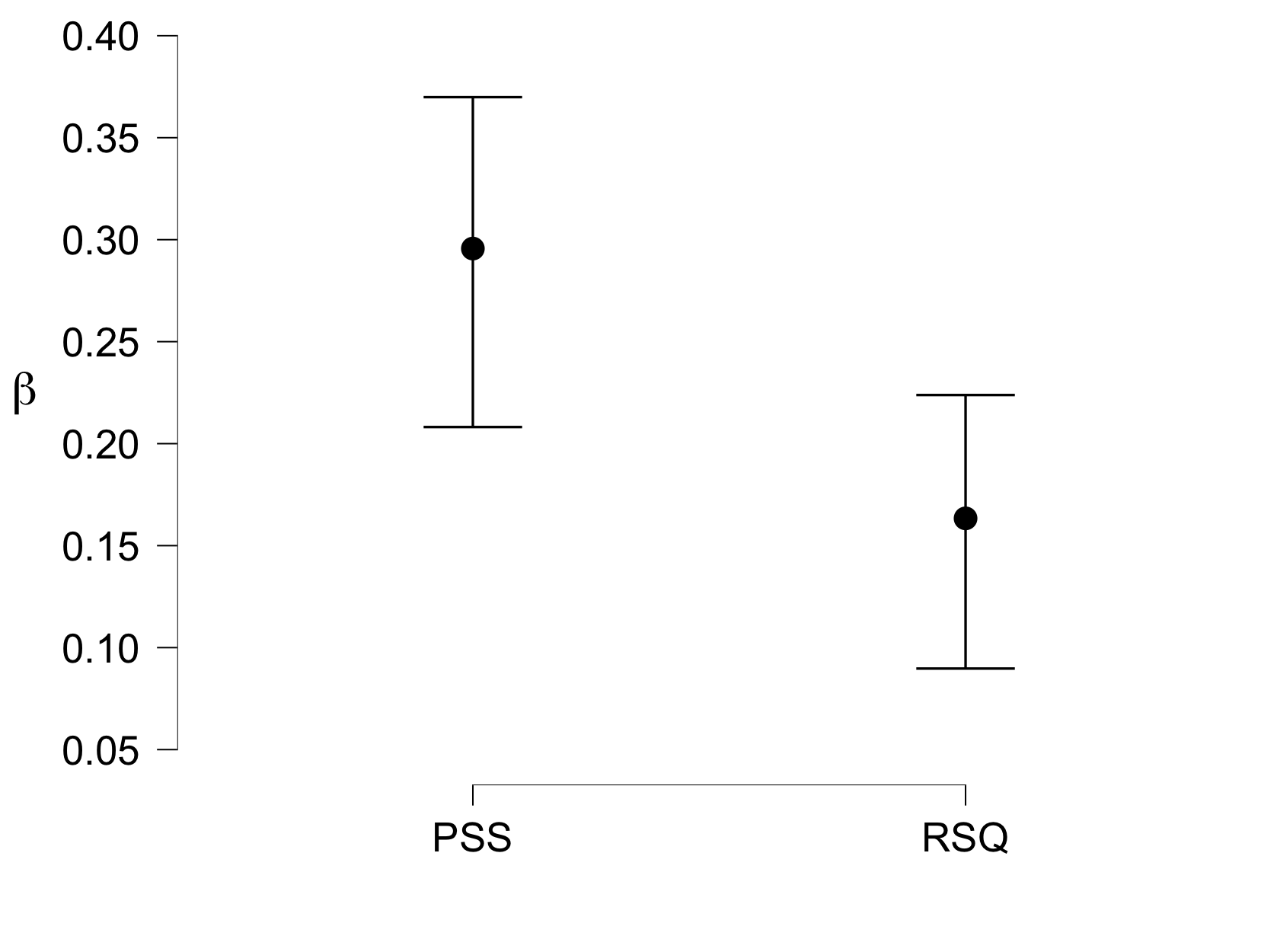

Supplement: sj-jasp-3-hpq-10.1177_13591053211059393 – Supplemental material for Individual factors in the relationship between stress and resilience in mental health psychology practitioners during the COVID-19 pandemic [file sj-jasp-3-hpq-10.1177_13591053211059393.jasp › resources/51/_8_t1603025763177.png]

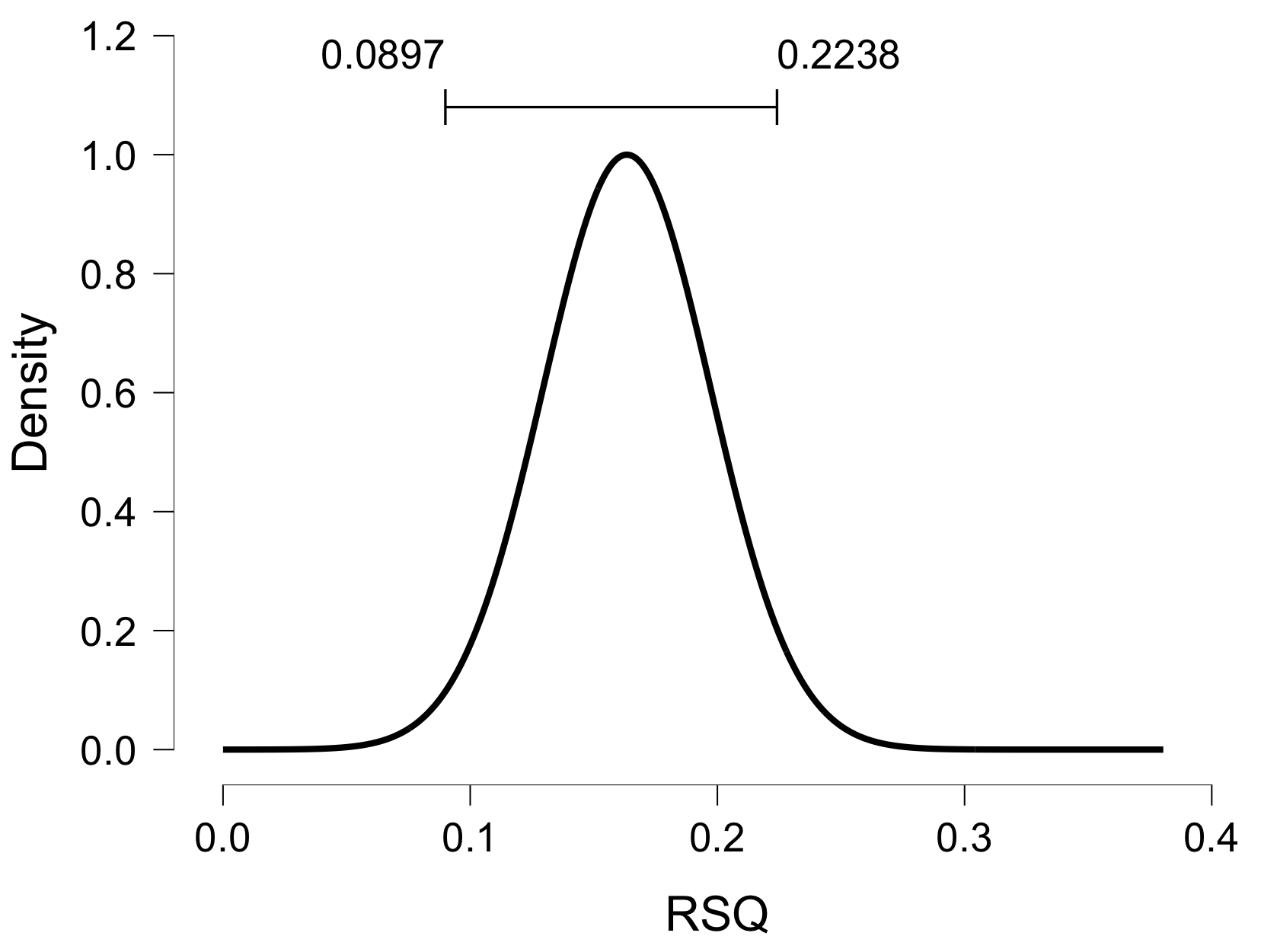

Supplement: sj-jasp-3-hpq-10.1177_13591053211059393 – Supplemental material for Individual factors in the relationship between stress and resilience in mental health psychology practitioners during the COVID-19 pandemic [file sj-jasp-3-hpq-10.1177_13591053211059393.jasp › resources/51/_3_t1603025773280.png]

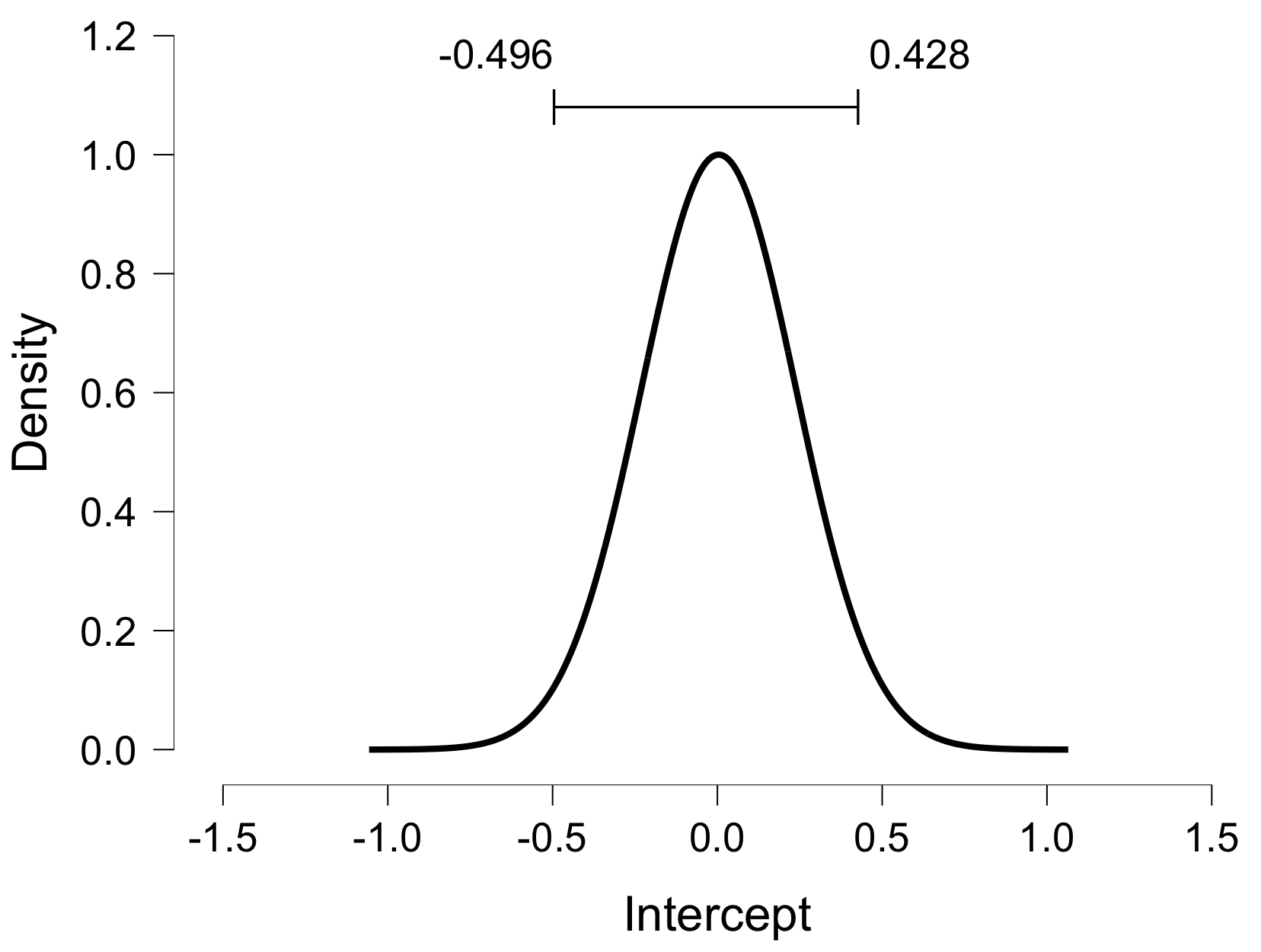

Supplement: sj-jasp-3-hpq-10.1177_13591053211059393 – Supplemental material for Individual factors in the relationship between stress and resilience in mental health psychology practitioners during the COVID-19 pandemic [file sj-jasp-3-hpq-10.1177_13591053211059393.jasp › resources/51/_1_t1603025772380.png]

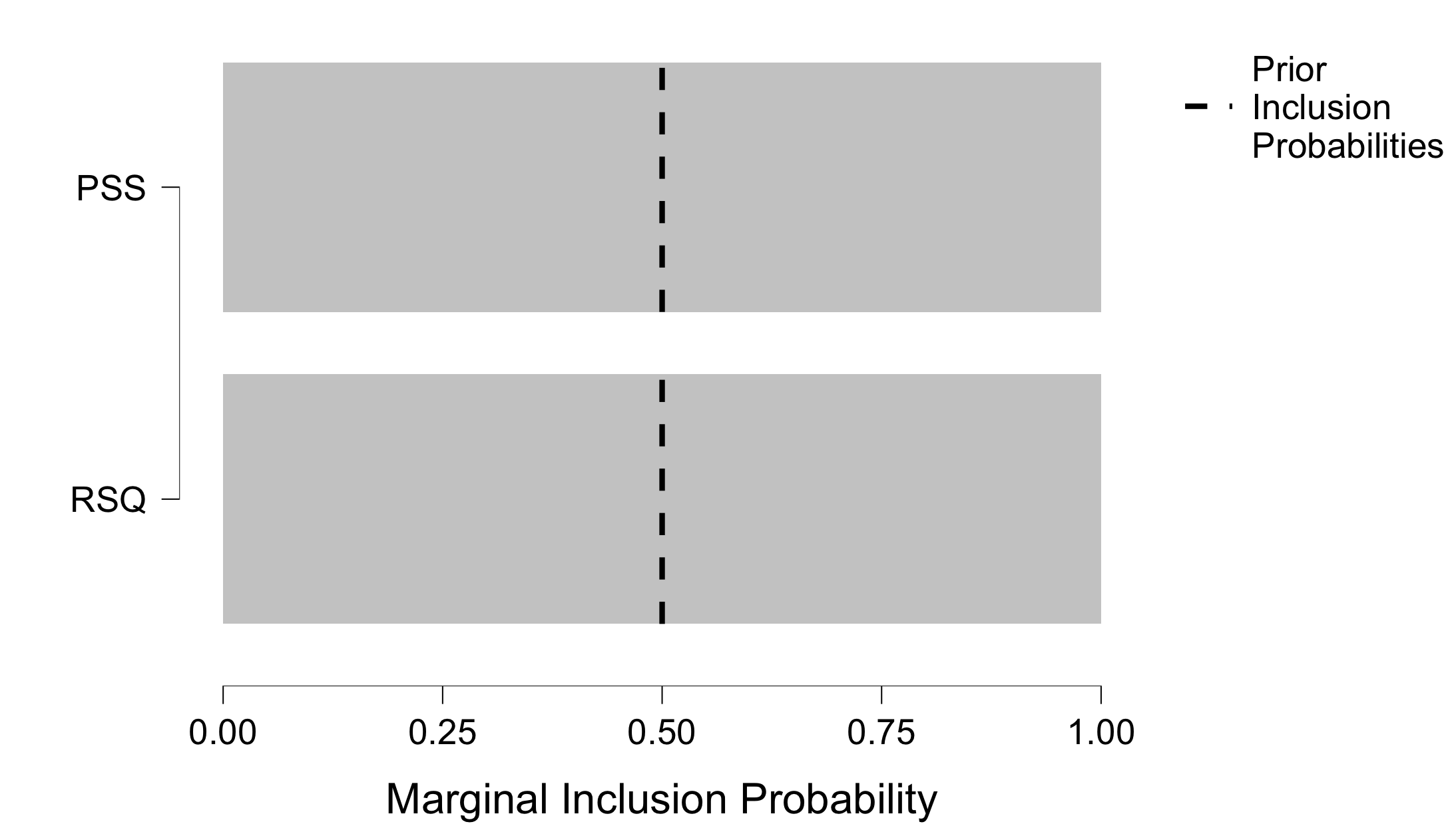

Supplement: sj-jasp-3-hpq-10.1177_13591053211059393 – Supplemental material for Individual factors in the relationship between stress and resilience in mental health psychology practitioners during the COVID-19 pandemic [file sj-jasp-3-hpq-10.1177_13591053211059393.jasp › resources/51/_9_t1603025768457.png]

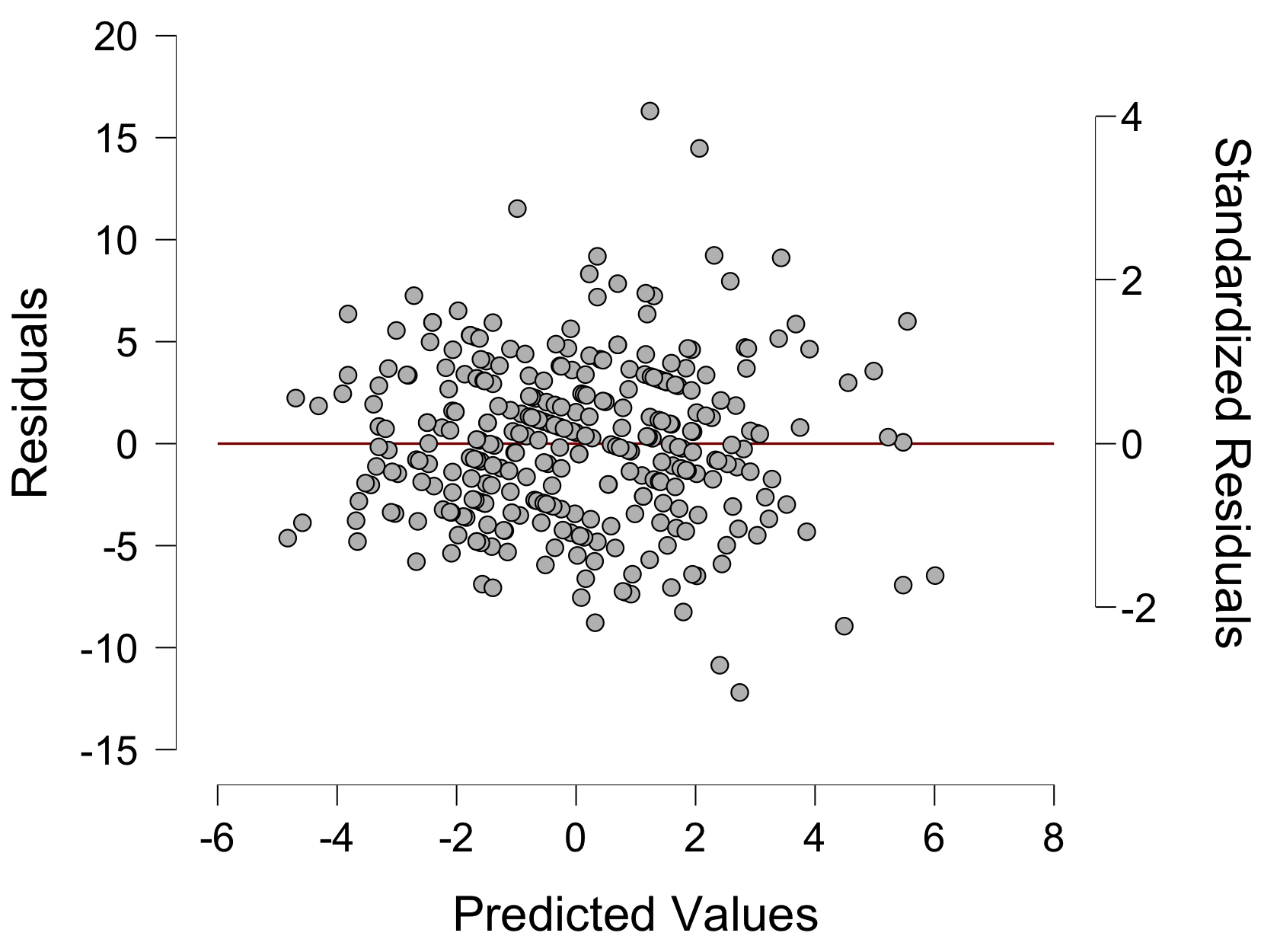

Supplement: sj-jasp-3-hpq-10.1177_13591053211059393 – Supplemental material for Individual factors in the relationship between stress and resilience in mental health psychology practitioners during the COVID-19 pandemic [file sj-jasp-3-hpq-10.1177_13591053211059393.jasp › resources/52/_0_t1603025883633.png]

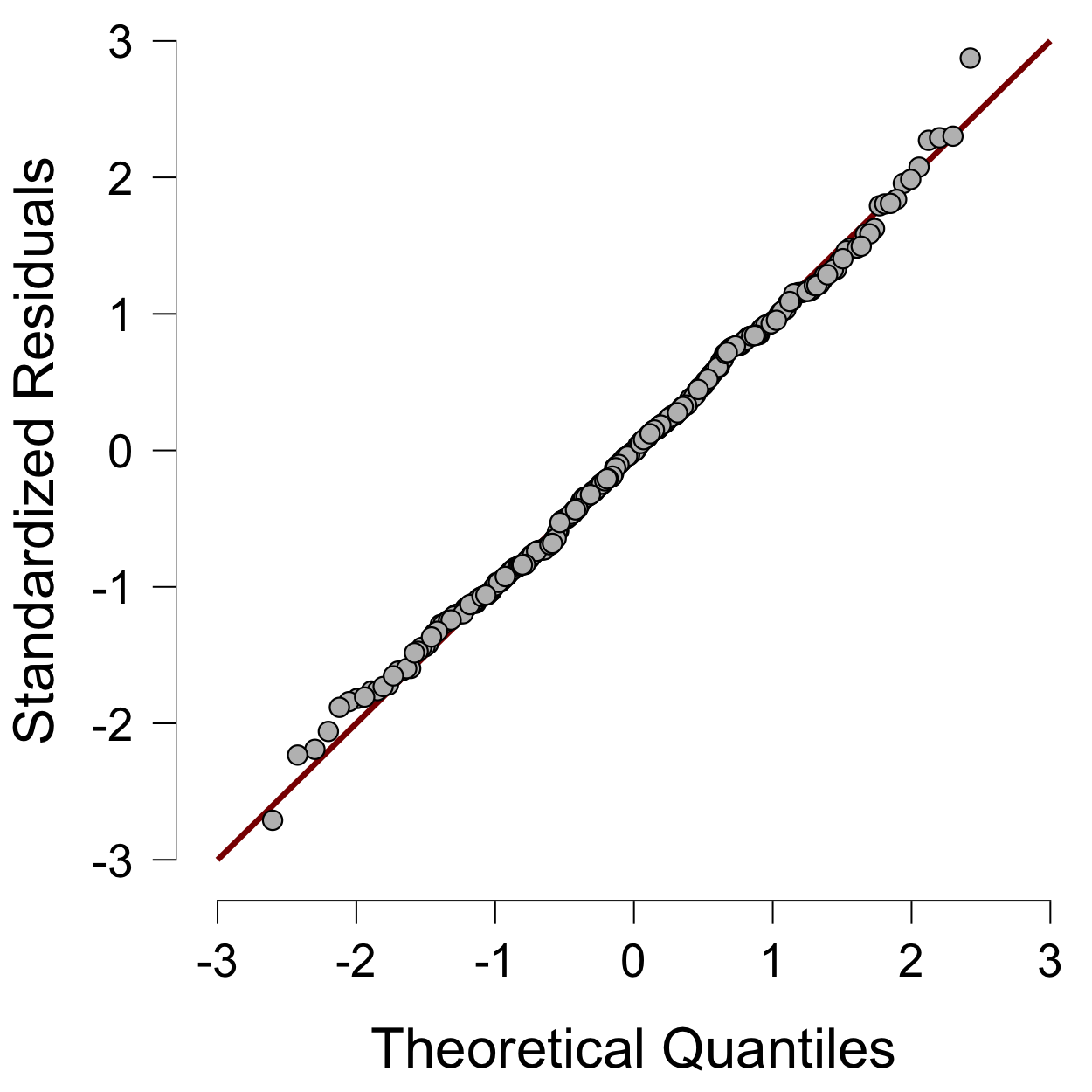

Supplement: sj-jasp-3-hpq-10.1177_13591053211059393 – Supplemental material for Individual factors in the relationship between stress and resilience in mental health psychology practitioners during the COVID-19 pandemic [file sj-jasp-3-hpq-10.1177_13591053211059393.jasp › resources/52/_1_t1603025884721.png]

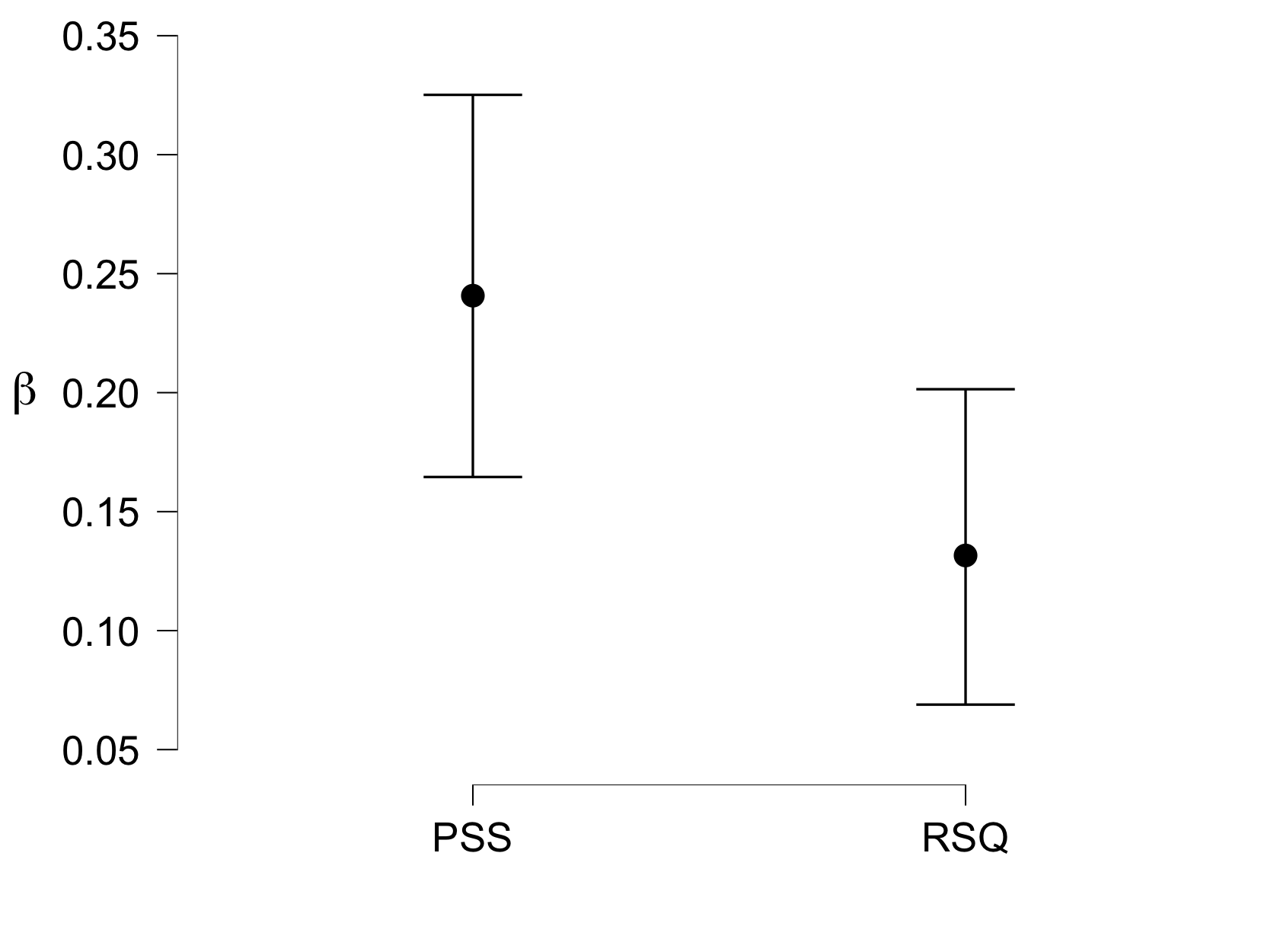

Supplement: sj-jasp-3-hpq-10.1177_13591053211059393 – Supplemental material for Individual factors in the relationship between stress and resilience in mental health psychology practitioners during the COVID-19 pandemic [file sj-jasp-3-hpq-10.1177_13591053211059393.jasp › resources/53/_4_t1603025955667.png]

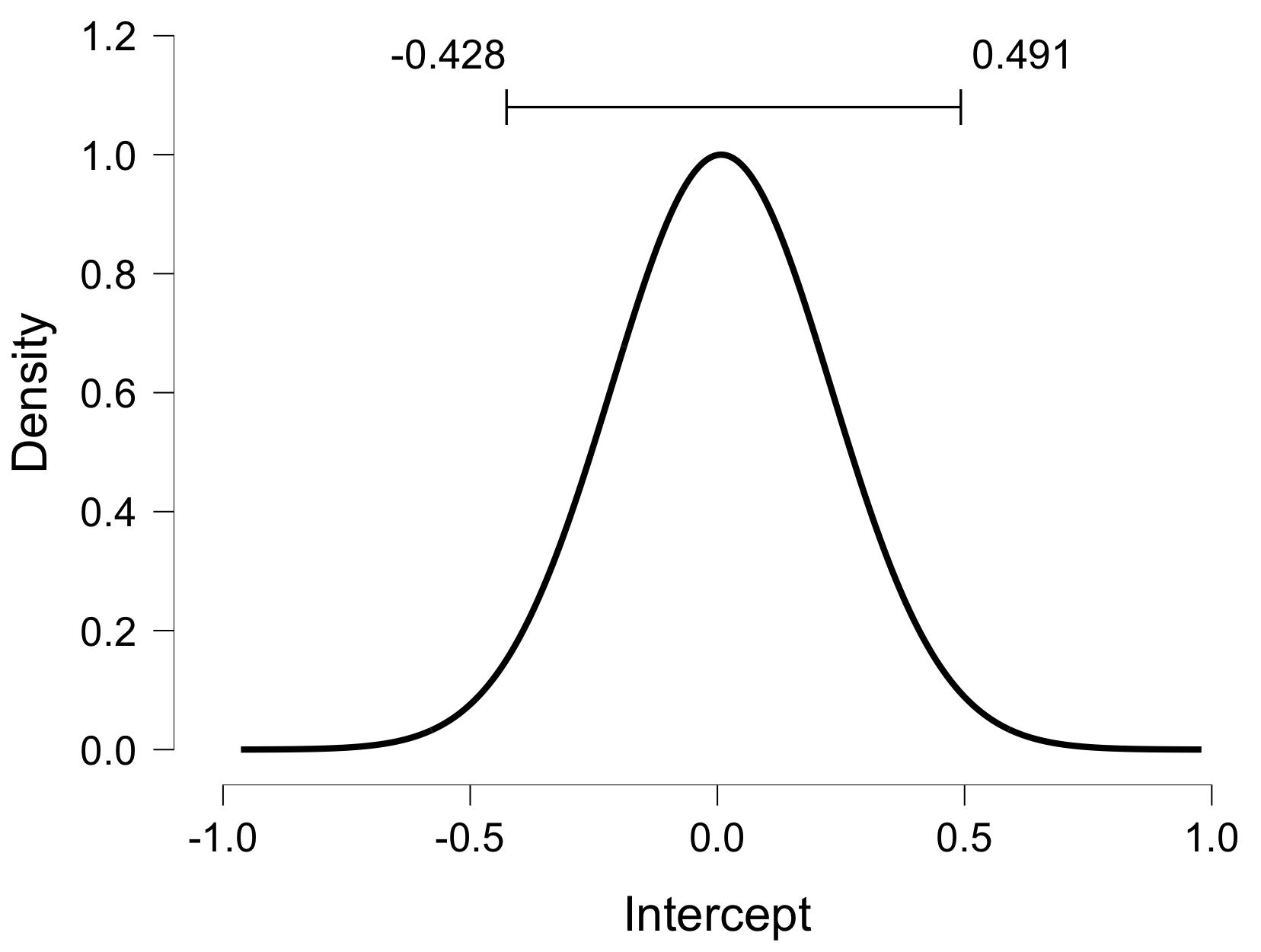

Supplement: sj-jasp-3-hpq-10.1177_13591053211059393 – Supplemental material for Individual factors in the relationship between stress and resilience in mental health psychology practitioners during the COVID-19 pandemic [file sj-jasp-3-hpq-10.1177_13591053211059393.jasp › resources/53/_1_t1603025965702.png]

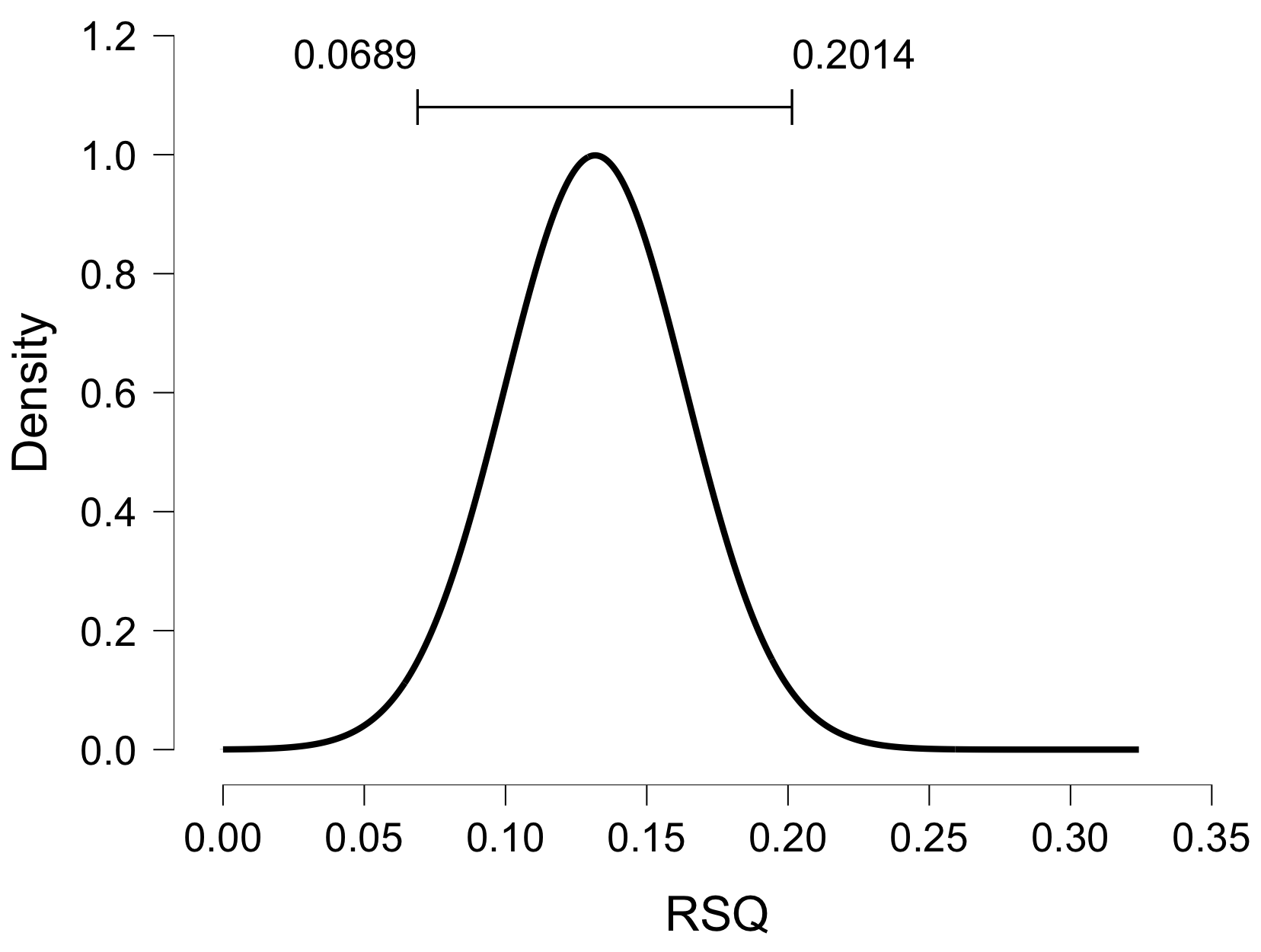

Supplement: sj-jasp-3-hpq-10.1177_13591053211059393 – Supplemental material for Individual factors in the relationship between stress and resilience in mental health psychology practitioners during the COVID-19 pandemic [file sj-jasp-3-hpq-10.1177_13591053211059393.jasp › resources/53/_3_t1603025966306.png]

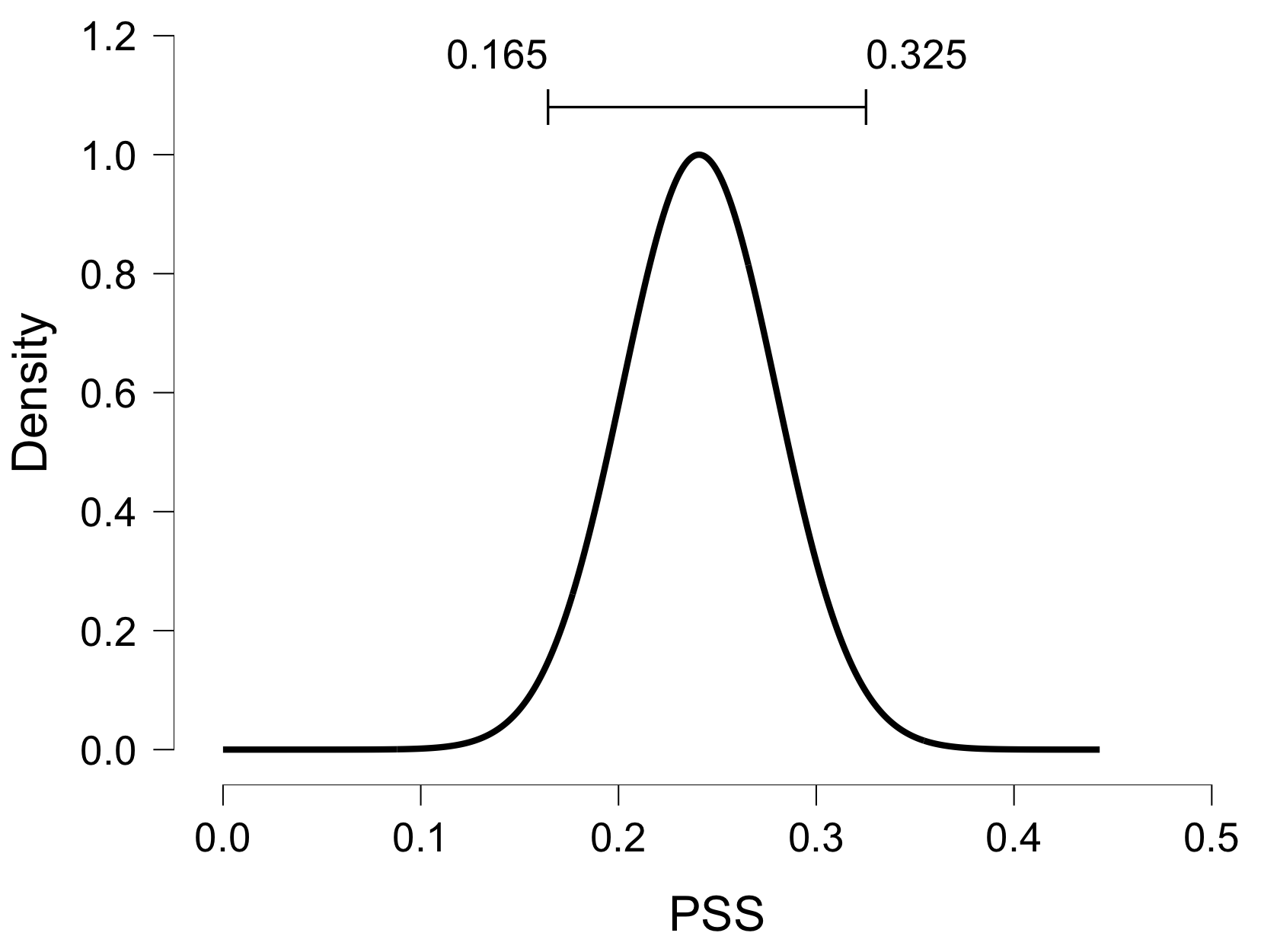

Supplement: sj-jasp-3-hpq-10.1177_13591053211059393 – Supplemental material for Individual factors in the relationship between stress and resilience in mental health psychology practitioners during the COVID-19 pandemic [file sj-jasp-3-hpq-10.1177_13591053211059393.jasp › resources/53/_2_t1603025966044.png]

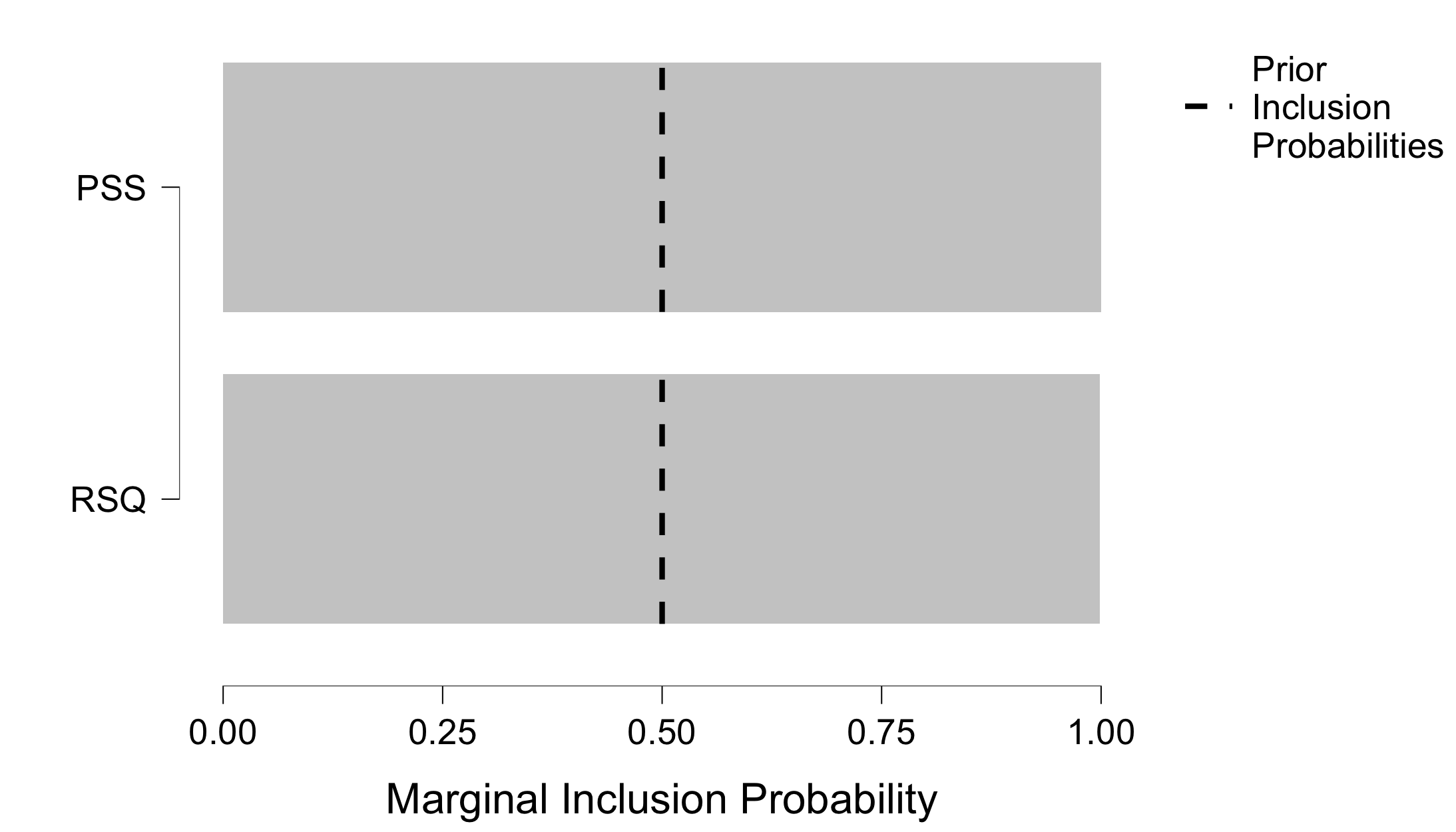

Supplement: sj-jasp-3-hpq-10.1177_13591053211059393 – Supplemental material for Individual factors in the relationship between stress and resilience in mental health psychology practitioners during the COVID-19 pandemic [file sj-jasp-3-hpq-10.1177_13591053211059393.jasp › resources/53/_5_t1603025958091.png]

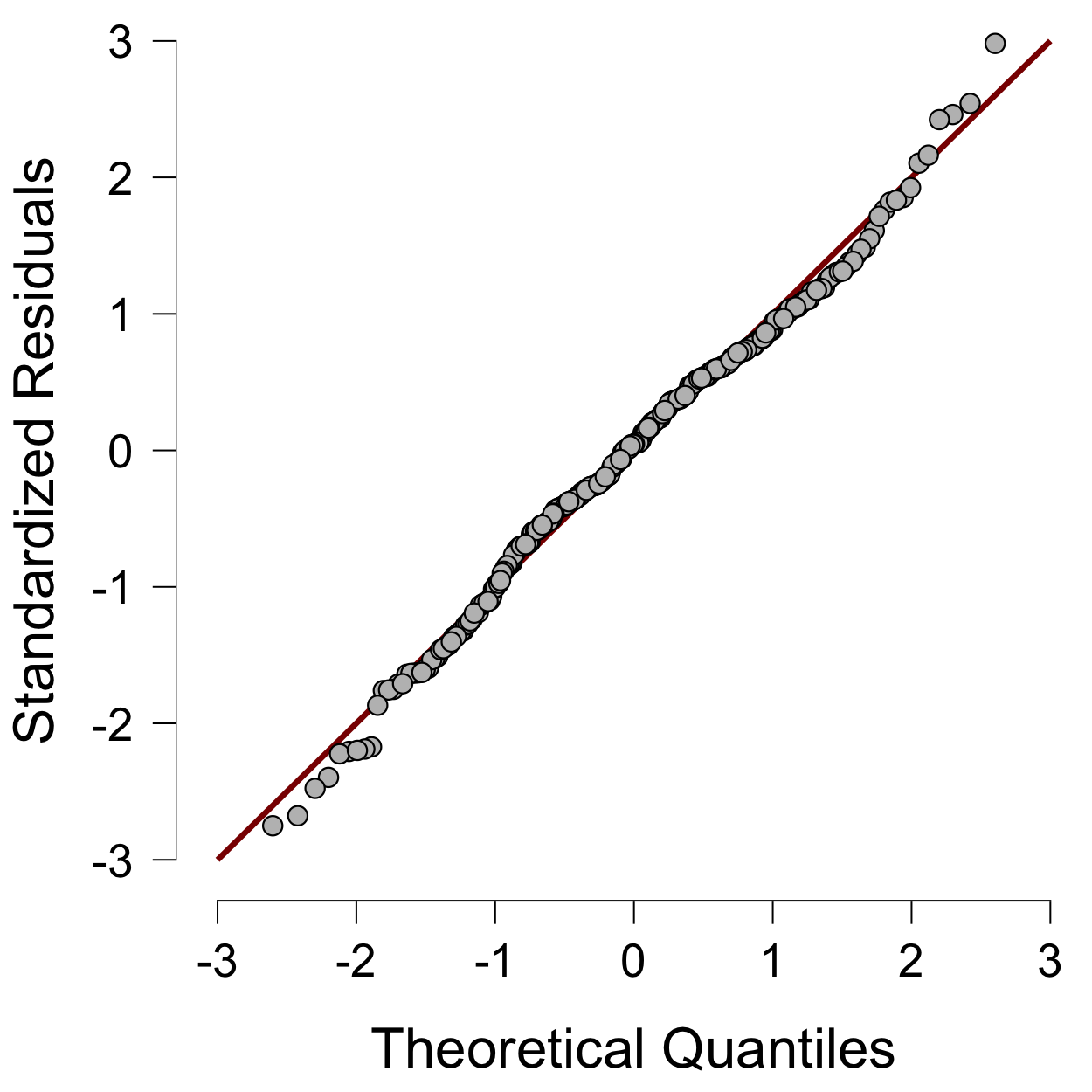

Supplement: sj-jasp-3-hpq-10.1177_13591053211059393 – Supplemental material for Individual factors in the relationship between stress and resilience in mental health psychology practitioners during the COVID-19 pandemic [file sj-jasp-3-hpq-10.1177_13591053211059393.jasp › resources/21/_3_t1603037069722.png]

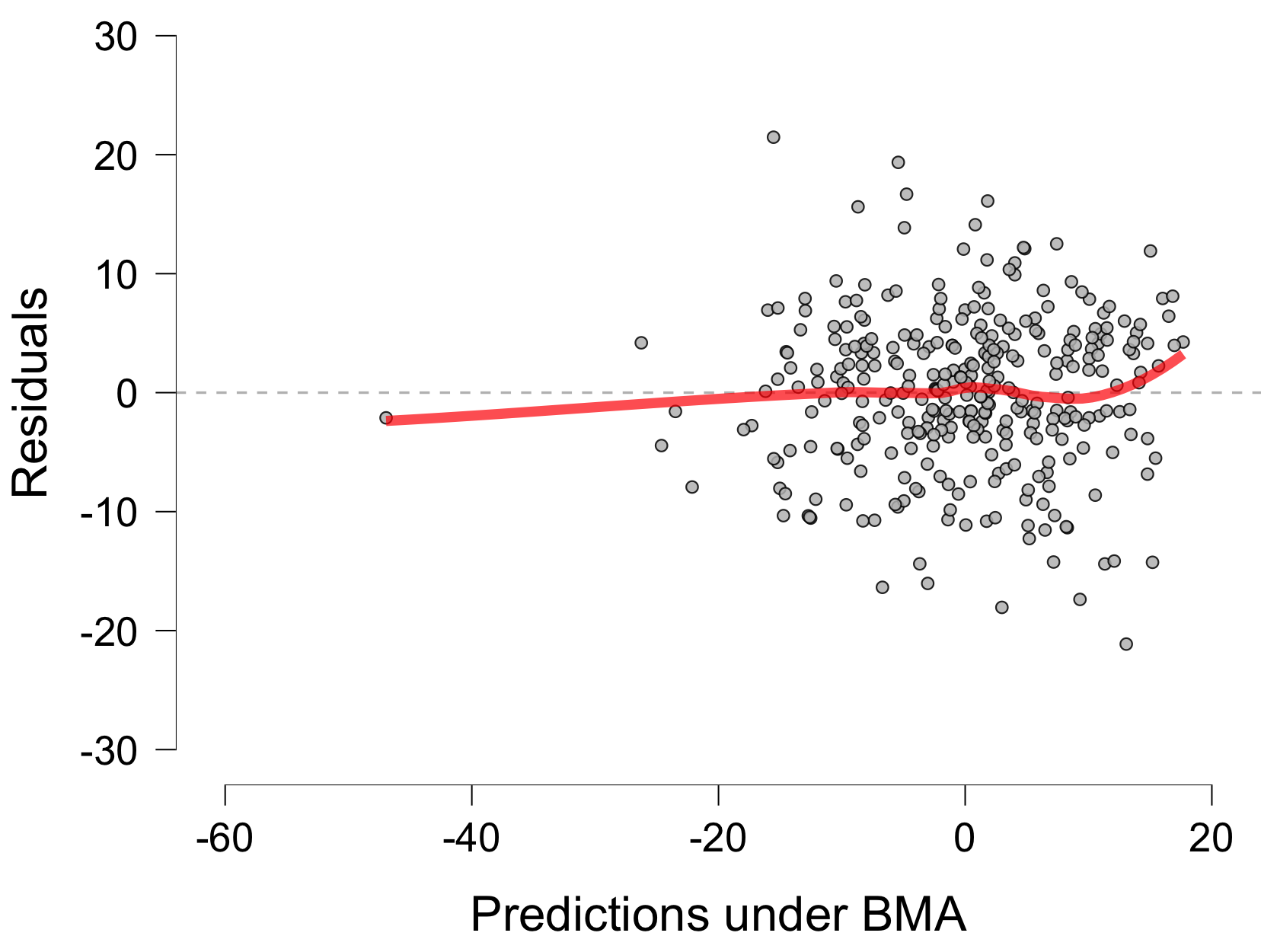

Supplement: sj-jasp-3-hpq-10.1177_13591053211059393 – Supplemental material for Individual factors in the relationship between stress and resilience in mental health psychology practitioners during the COVID-19 pandemic [file sj-jasp-3-hpq-10.1177_13591053211059393.jasp › resources/54/_7_t1603039162482.png]

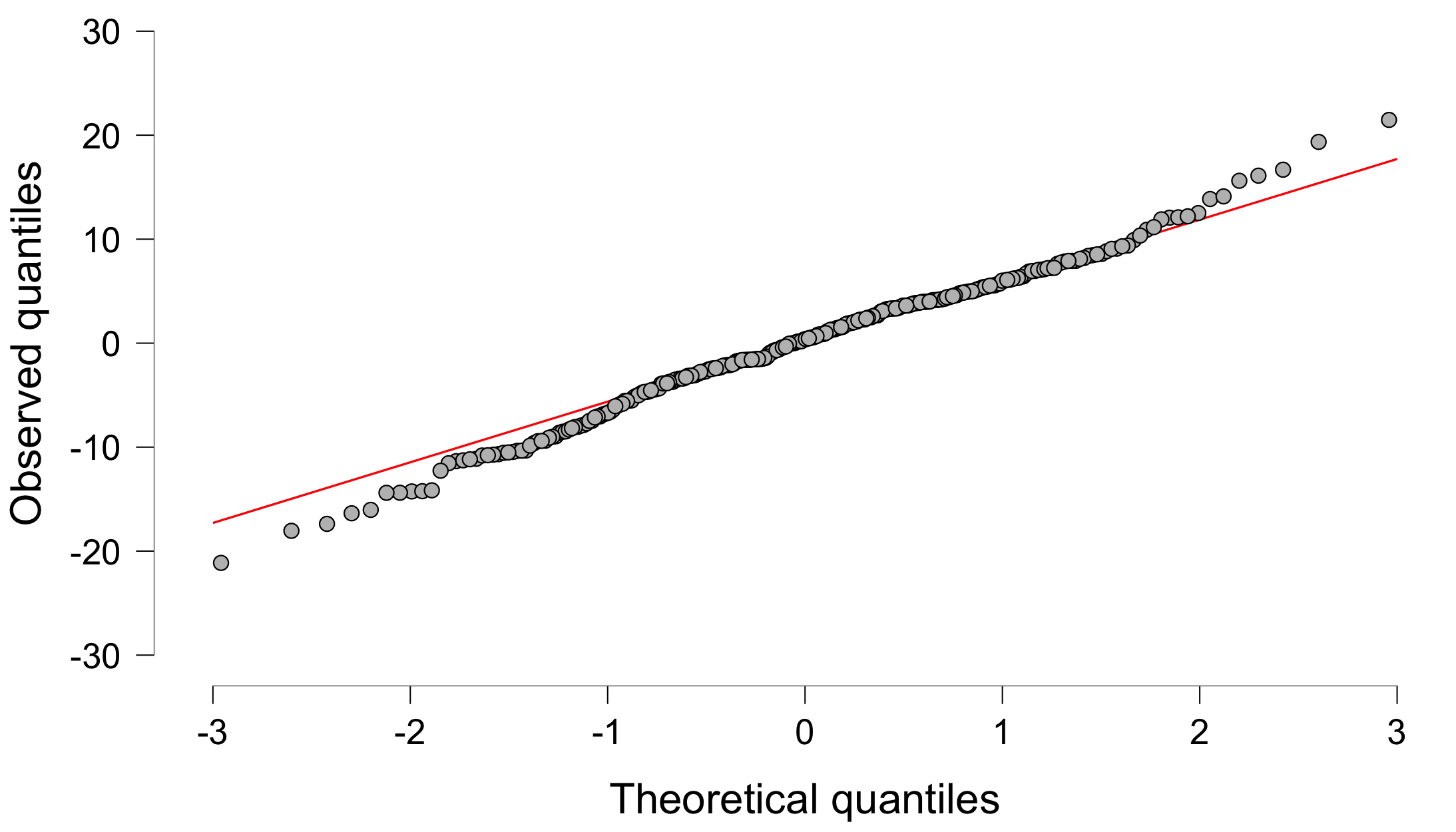

Supplement: sj-jasp-3-hpq-10.1177_13591053211059393 – Supplemental material for Individual factors in the relationship between stress and resilience in mental health psychology practitioners during the COVID-19 pandemic [file sj-jasp-3-hpq-10.1177_13591053211059393.jasp › resources/54/_8_t1603039162703.png]

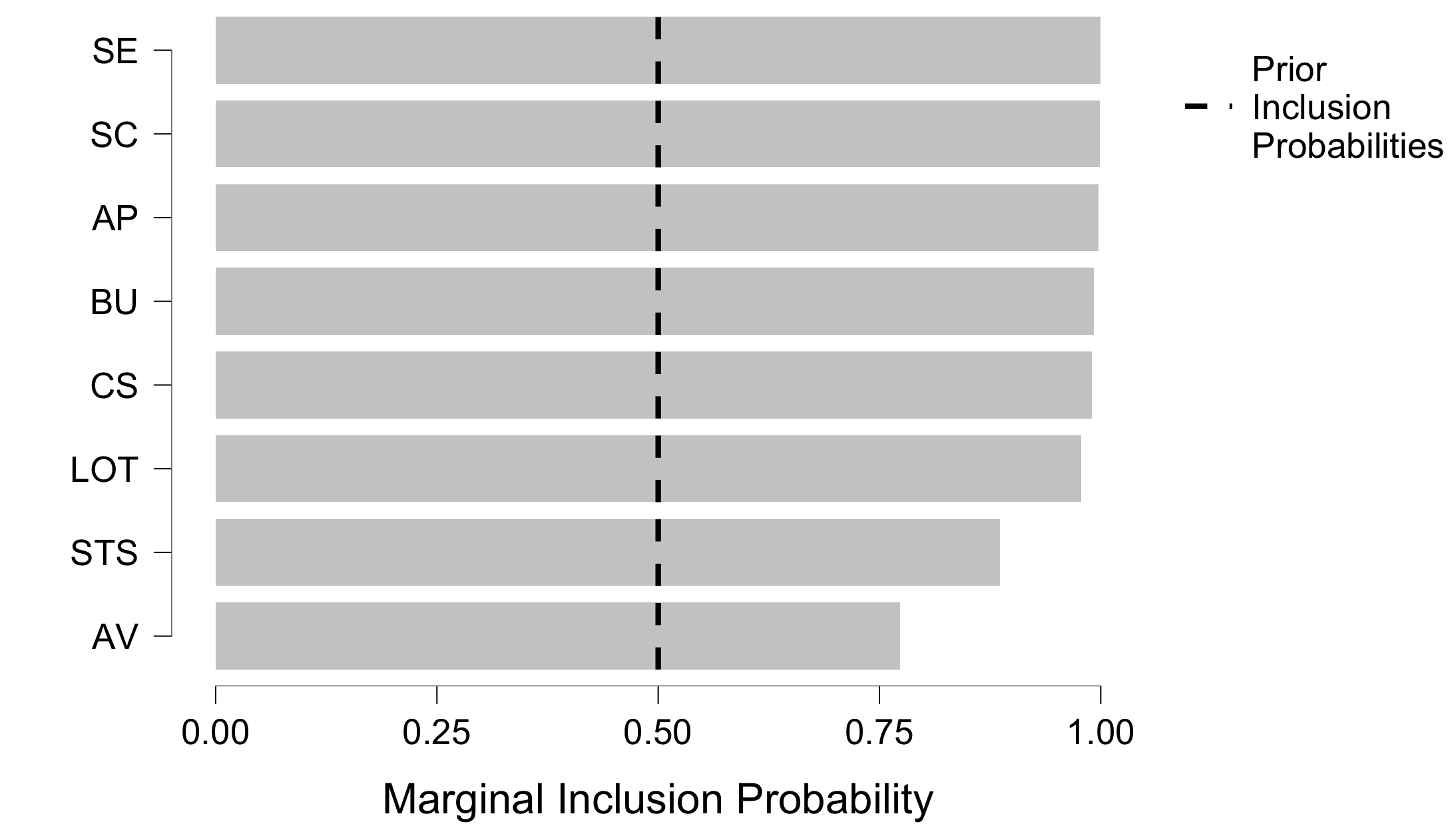

Supplement: sj-jasp-3-hpq-10.1177_13591053211059393 – Supplemental material for Individual factors in the relationship between stress and resilience in mental health psychology practitioners during the COVID-19 pandemic [file sj-jasp-3-hpq-10.1177_13591053211059393.jasp › resources/54/_9_t1603039171250.png]

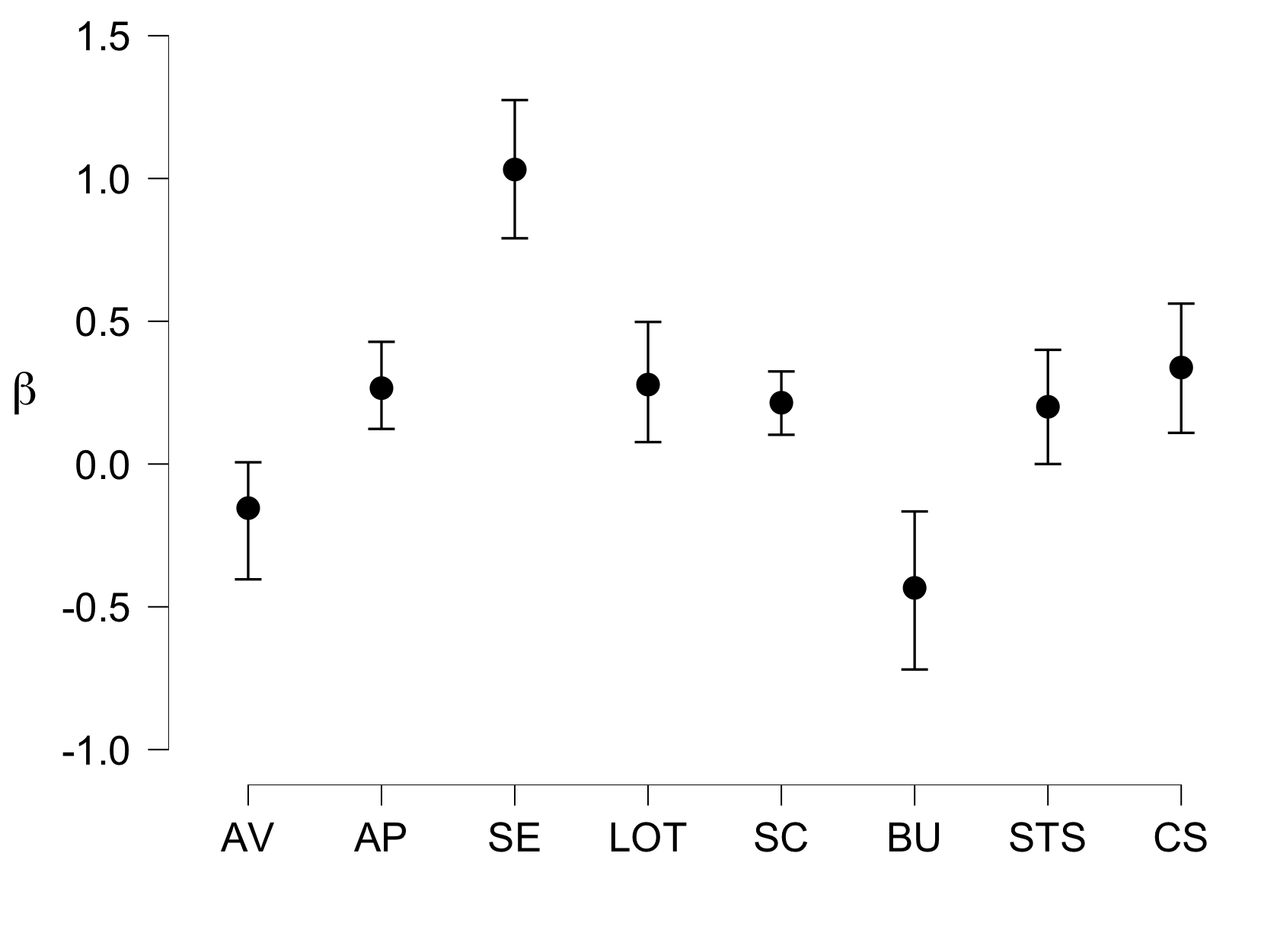

Supplement: sj-jasp-3-hpq-10.1177_13591053211059393 – Supplemental material for Individual factors in the relationship between stress and resilience in mental health psychology practitioners during the COVID-19 pandemic [file sj-jasp-3-hpq-10.1177_13591053211059393.jasp › resources/54/_6_t1603039162251.png]

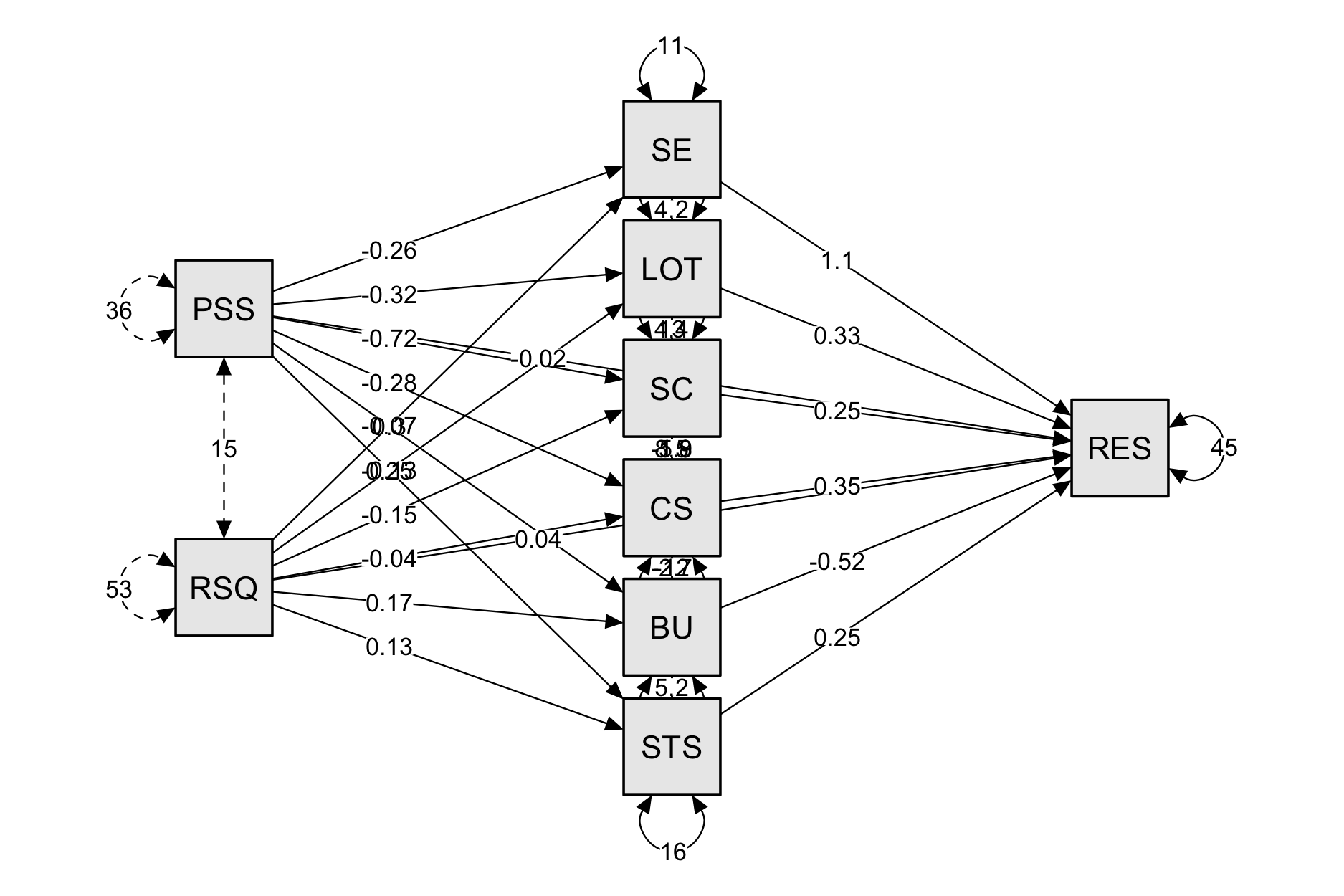

Supplement: sj-jasp-3-hpq-10.1177_13591053211059393 – Supplemental material for Individual factors in the relationship between stress and resilience in mental health psychology practitioners during the COVID-19 pandemic [file sj-jasp-3-hpq-10.1177_13591053211059393.jasp › resources/55/_0_t1611566660063.png]
