# Supplementary material for: Individual factors in the relationship between stress and resilience in mental health psychology practitioners during the COVID-19 pandemic
Source: J Health Psychol. 2021 Dec 7;27(11):2613–31. doi: 10.1177/13591053211059393 (PMC9483698; doi:10.1177/13591053211059393)
Supplement: sj-pdf-5-hpq-10.1177_13591053211059393 – Supplemental material for Individual factors in the relationship between stress and resilience in mental health psychology practitioners during the COVID-19 pandemic [file sj-pdf-5-hpq-10.1177_13591053211059393.pdf]

We submitted the following five documents. Their purpose is to offer the reviewers and readers more details about the analyses we performed and offered them the opportunity to replicate the findings of our study.

1. Data_analysis_score.text
2. Mediation_data_analysis.jasp
3. Mediation_data_analysis.html
4. Power.Sample size.docx
5. Supplementary material

- The text file provides raw data
- The html file provides all listed analyses. However, we can supplement this with JASP file. We created the html file based on this JASP.
- Document 4 and 5 are word files and provide additional information on the analyses we conducted.

We are happy to provide more information if requested by the reviewers.
